# HPW-Catalyzed environmentally benign approach to imidazo[1,2-*a*]pyridines

**DOI:** 10.3762/bjoc.20.55

**Published:** 2024-03-19

**Authors:** Luan A Martinho, Carlos Kleber Z Andrade

**Affiliations:** 1 Instituto de Química, Laboratório de Química Metodológica e Orgânica Sintética (LaQMOS), Universidade de Brasília, 70904-970, Brasília, DF, Brazil

**Keywords:** GBB-3CR, imidazo[1,2-*a*]pyridine, microwave, phosphotungstic acid

## Abstract

The imidazo[1,2-*a*]pyridine moiety is present in drugs with several biological activities. The most direct way of obtaining this nucleus is the Groebke–Blackburn–Bienaymé three-component reaction (GBB-3CR) between aminopyridines, aldehydes, and isocyanides under both Lewis and Brønsted acid catalysis. However, several catalysts for this reaction have major drawbacks such as being expensive, extremely dangerous, strong oxidizing, and even explosive. In this scenario, heteropolyacids emerge as greener and safer alternatives due to their very strong Brønsted acidity. In particular, phosphotungstic acid (HPW) is an economical and green attractive catalyst for being cheap, non-toxic, and is known for its chemical and thermal stability. Herein, we report a straightforward approach to the GBB-3CR using HPW as catalyst in ethanol under microwave (μw) heating. This convenient environmentally benign methodology is broad in scope, provides the heterobicyclic products in high yields (up to 99%), with a low catalyst loading (2 mol %) in only 30 minutes, and allows the successful use of aliphatic aldehydes, substrates not so frequently explored with most usual catalysts for this reaction. Furthermore, the aforementioned advantages make this methodology very attractive and superior to the existing ones.

## Introduction

**Figure 1:**
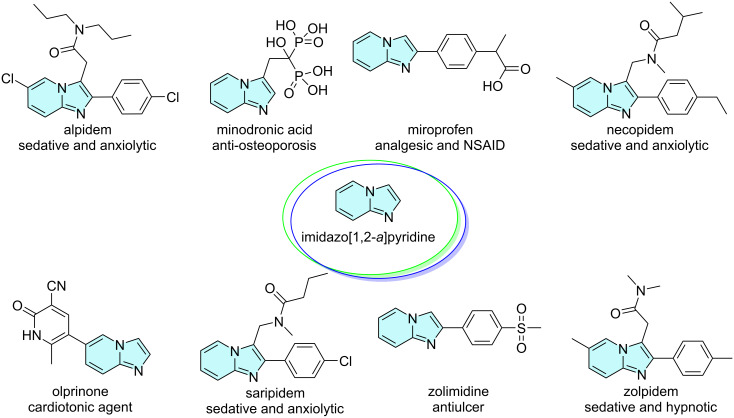
Selected examples of commercial drugs containing the imidazo[1,2-*a*]pyridine core [13].

Imidazo[1,2-*a*]pyridine is a privileged structure that plays an important role in organic synthesis and in the pharmaceutical industry. This scaffold is present in drugs with several biological activities, such as antiviral [1], anticonvulsant [2], antibacterial [3], antipyretic [4], antituberculosis [5], anticancer [6], anthelmintic [5], antifungal [7], analgesic [8], antiulcer [9], antiprotozoal [10], antitumor [11], and anti-inflammatory [12]. Examples of commercial drugs are depicted in Figure 1 [13] and include alpidem (anxiety disorders), minodronic acid (osteoporosis), miroprofen (non-steroidal anti-inflammatory drug, NSAID), necopidem (insomnia), olprinone (acute heart failure), saripidem (type A GABA receptor agonist), zolimidine (gastroesophageal reflux disease), and zolpidem (insomnia).

Some recent synthetic approaches to imidazo[1,2-*a*]pyridine scaffolds include synthetic pathways of transition metal-catalyzed reactions [14], cyclization [15], condensation [16], heteroannular [17], and photocatalytic reactions [18]. These approaches usually involve non-trivial reaction conditions and the employment of relatively complex starting materials [19]. A more efficient way of obtaining this nucleus is through the Groebke–Blackburn–Bienaymé three-component reaction (GBB-3CR) between amidines (aminoazoles), aldehydes, and isocyanides under both Lewis and Brønsted acid catalysis [20–22]. Multicomponent reactions (MCRs) provide one-pot reactions, simple synthetic procedures, less waste being produced, fewer purification steps, and a high atom economy [23].

The GBB three-component reaction is carried out in the presence of Lewis or Brønsted acid catalysis to increase the reactivity of the imine formation [24]. The most common catalysts are those derived from triflate salts such as Sc(OTf)_3_ [25], Yb(OTf)_3_ [26], In(OTf)_3_ [27] and Gd(OTf)_3_ [28], and inorganic Brønsted or Lewis acids like HClO_4_ [29], ZrCl_4_ [30], InCl_3_ [31], BiCl_3_ [32], RuCl_3_ [33], NH_4_Cl [34], HCl [35], LaCl_3_·7H_2_O [36], and ZnCl_2_ [37], or organic acids such as *p*-toluenesulfonic acid (PTSA) [38], TFA [39], and AcOH [40], etc. Nonetheless, most of these acids have major drawbacks such as being expensive, dangerous, strong oxidizing, or even potentially explosive, with long reaction times being required.

**Figure 2:**
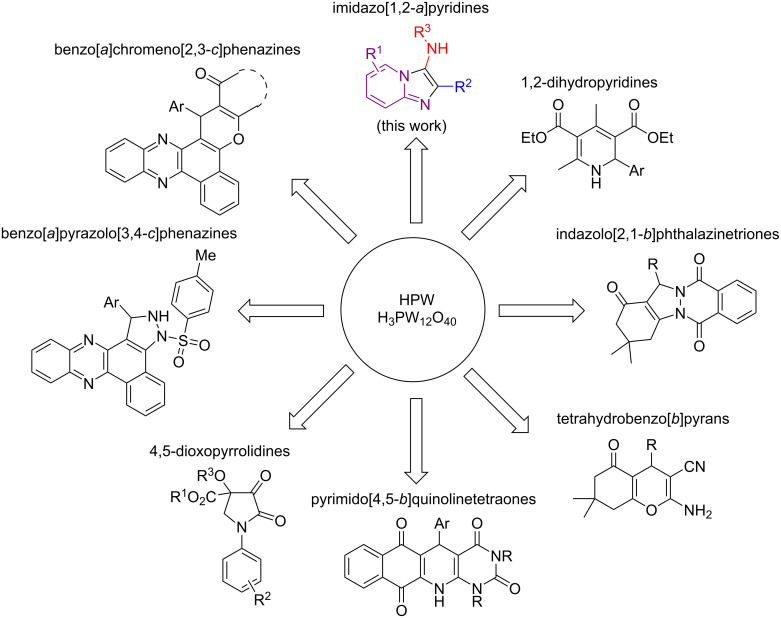
Examples of application of HPW as catalyst in the synthesis of heterocyclic compounds through multicomponent reaction approaches.

In contrast, heteropolyacids (HPAs) have been reported as greener catalysts not only for being a safer alternative to toxic catalysts [41] but also for providing easy recovery and reuse linked to high selectivity [42]. An example of HPA is phosphotungstic acid, H_3_PW_12_O_40_ (HPW), an inexpensive, non-toxic, and green catalyst with greater chemical and thermal stability in comparison to other heteropolyacids [43]. HPW has been shown to catalyze MCRs in the synthesis of heterocyclic compounds with high efficiency and chemoselectivity (Figure 2), including functionalized benzo[*c*]chromeno[2,3-*a*]phenazine [44], pyrazolo-fused benzophenazines [45], 4,5-dioxopyrrolidines [46], 1,2-dihydropyridine (1,2-DHPs) [47], pyrimido[4,5-*b*]quinoline-tetraones [48], tetrahydrobenzo[*b*]pyrans and indazolo[2,1-*b*]phthalazinetriones [49]. Herein, we report the synthesis of imidazo[1,2-*a*]pyridines via the GBB-3CR using HPW as catalyst in ethanol under microwave (μw) heating. This convenient environmentally benign methodology is broad in scope, provides the heterobicyclic products in high yields (up to 99%), with a low catalyst loading (2 mol %) in only 30 minutes.

## Results and Discussion

**Scheme 1:**
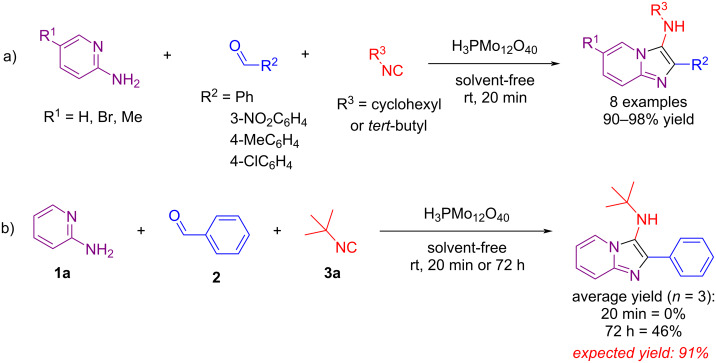
a) Reported phosphomolybdic acid-catalyzed synthesis of imidazo[1,2-*a*]pyridines via GBB-3CR. b) Attempts to reproduce the results reported in (a).

A search in the literature revealed a single report on the use of a heteropolyacid (phosphomolybdic acid) in the GBB reaction but for a limited number of examples (Scheme 1a) [50]. That caught our attention for being the only example so far of a room temperature GBB reaction carried out in less than an 18-hour period. However, at least in our hands, when we tried to reproduce the reaction between 2-aminopyridine (**1a**), benzaldehyde (**2**), and *tert*-butyl isocyanide (**3a**), following in detail the reported protocol using exactly the same reaction conditions, the reaction failed completely in 20 minutes (followed by TLC) and after 72 h only 46% yield (compared to the expected 91% yield) was obtained (Scheme 1b). This reaction was carried out in triplicate with the same outcome. Therefore, the search for a broader and more reliable methodology to obtain imidazo[1,2-*a*]pyridines using heteropolyacids became necessary.

Due to its already mentioned attractive intrinsic properties, HPW was used in our initial studies to find the best reaction conditions. Additionally, knowing that a higher temperature is crucial for faster GBB reactions, μw heating was used, taking into account our experience over the years on the μw-mediated MCRs [51–53]. 2-Aminopyridine (**1a**), 4-nitrobenzaldehyde (**2a**), and *tert*-butyl isocyanide (**3a**) were chosen as model substrates and different conditions were screened (Table 1). Glycerol, a green and sustainable solvent, was tried first, but unfortunately, the expected intense orange solid product **4a** was obtained in low yields (Table 1, entries 1 and 2).

**Table 1:** Optimization of the reaction conditions.^a^

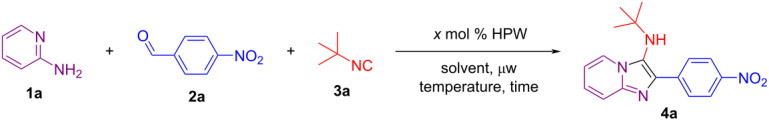					

Entry	HPW (mol %)	Solvent	Temp. (°C)	Time (min)	Yield (%)^b^

1	10	glycerol	100	5	25
2	10	glycerol	100	30	29
3	10	MeOH	100	5	54
4	10	MeOH	120	30	76
5	5	MeOH	120	30	86
6	2	MeOH	100	30	77
7	2	MeOH	120	30	87
8	1	MeOH	120	30	81
9	2	MeOH	100	30	77
10	2	MeOH	150	30	86
11	2	MeOH	120	5	68
12	2	MeOH	120	15	81
**13**	**2**	**EtOH**	**120**	**30**	**87**
14	2	H_2_O	120	30	17
15	2	EtOH/H_2_O 1:1	120	30	25
16	2	solvent-free	120	30	24

^a^Reaction conditions: 2-aminopyridine (0.50 mmol), 4-nitrobenzaldehyde (0.50 mmol), and *tert*-butyl isocyanide (0.50 mmol) in 0.5 mL of the specified solvent. ^b^Isolated yields after column chromatography.

Significantly better yields were obtained using methanol, which is the most common solvent for this reaction, especially at 120 °C (Table 1, entries 3–12). Interestingly, the yield was also better for a lower catalyst loading (Table 1, entry 7). Further changes either in the temperature or in the reaction time were checked, however, with no benefit in the yields (Table 1, entries 9–12). Accordingly, with the established optimal conditions of 2 mol % of HPW, 120 °C, and 30 min, other solvents were tested, and ethanol was found to give similar results (Table 1, entry 13). For being much greener as compared to methanol, it was chosen as the solvent to study the reaction scope. The use of water or solvent-free conditions resulted in much lower reaction yields (Table 1, entries 14–16).

**Scheme 2:**
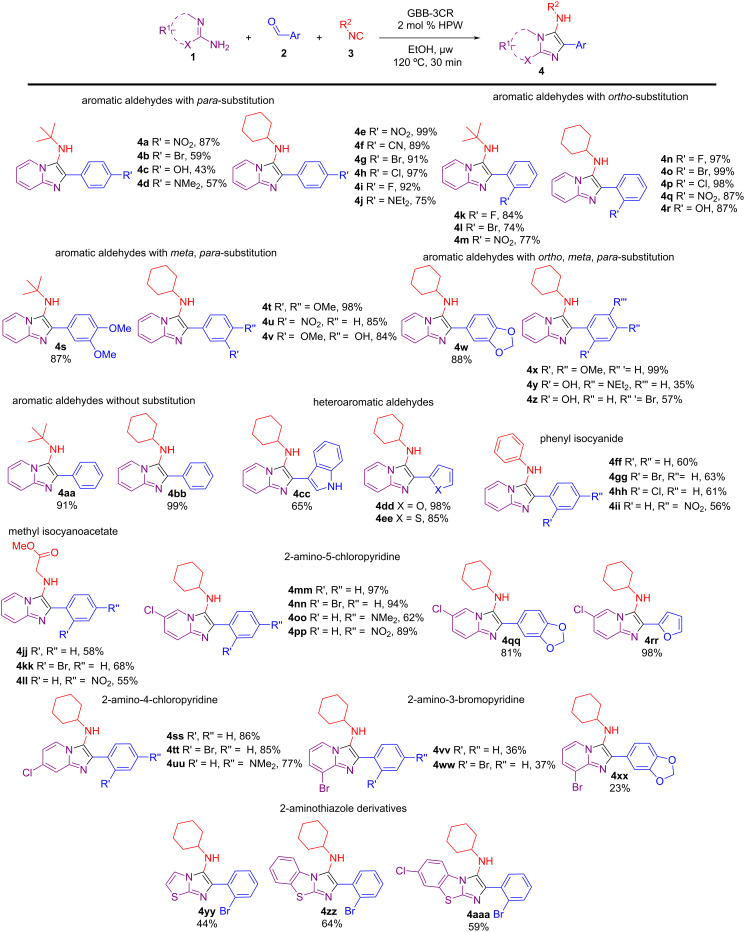
Substrate scope of the HPW-catalyzed GBB reactions using a range of aromatic/heteroaromatic aldehydes. Reaction conditions: 2-aminopyridine/2-aminothiazole (0.50 mmol), aromatic/heteroaromatic aldehyde (0.50 mmol), isocyanide (0.50 mmol), and HPW (0.01 mmol, 2 mol %) in EtOH (0.5 mL), under μw heating. The yields refer to isolated yields after column chromatography and the structures were confirmed by FTIR, NMR, and HRMS.

Next, the scope of the reaction was investigated with a range of 2-aminopyridines/2-aminothiazoles **1**, aromatic/heteroaromatic aldehydes **2**, and isocyanides **3** to obtain the imidazo[1,2-*a*]pyridine derivatives **4** (Scheme 2). In general, the efficiency of the HPW-catalyzed GBB reaction is very dependent upon the type of 2-aminopyridine or isocyanide compound used, and not influenced by different substituents at the aromatic aldehydes (both electron-withdrawing and electron-donating groups can be successfully used). The use of *para*-substituted aromatic aldehydes gave the corresponding products **4a**–**j** in moderate to excellent yields (up to 99%). Aromatic aldehydes with electron-donating groups at the *para*-position gave moderate product yields (**4c** and **4d**). Furthermore, cyclohexyl isocyanide gave higher yields compared to *tert*-butyl isocyanide. *Ortho*-substituted aromatic aldehydes were very efficient, regardless the type of substituents used, and good to excellent yields (74–99%) of products **4k**–**r** were obtained. *Meta*,*para*-substituted aromatic aldehydes showed a high reactivity with yields greater than 84% for products **4s**–**w**. Notably, the use of tri-substituted aromatic aldehydes gave imidazo[1,2-*a*]pyridines **4x**–**z** in moderate to excellent yields (35–99%), though the hydroxy substituent at the *ortho* position made the aldehyde less reactive. The use of non-substituted aromatic aldehydes also provided the expected products (**4aa** and **4bb**) in excellent yields (up to 99%). Heteroaromatic aldehydes gave the respective products **4cc**–**ee** in moderate to excellent yields (65–98%). However, the use of phenyl isocyanide and methyl isocyanoacetate furnished the desired products (**4ff**–**ii** and **4jj**–**ll**, respectively) in moderate yields (55–68%), possibly due to the lower reactivity of these isocyanides compared to *tert*-butyl and cyclohexyl isocyanides [54]. In the latter cases, transesterification of the ester group with ethanol was not observed. 2-Aminopyridines containing a chlorine atom in positions 4 or 5 provided the products **4mm**–**uu** in excellent yields (up to 97%). Nonetheless, with 2-amino-3-bromopyridine, low product yields were observed for **4vv**–**xx**, which may be due to the high hindrance of that substrate to imine formation, essential for the GBB-3CR mechanism. Moderate yields were obtained with the use of 2-aminothiazole derivatives (**4yy**–**aaa**). These lower yields did not change using MeOH as a solvent or increasing the amount of HPW used.

**Scheme 3:**
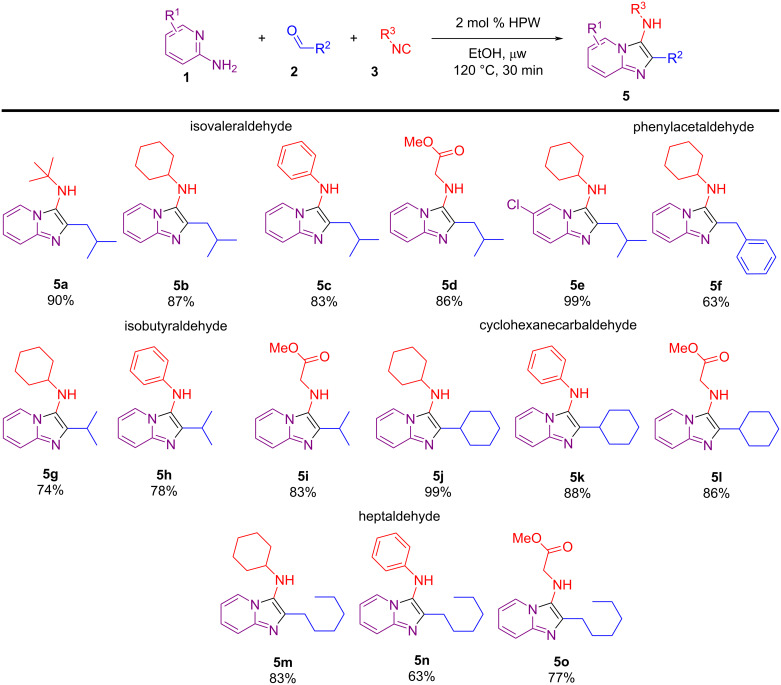
Substrate scope of the HPW-catalyzed GBB reaction using aliphatic aldehydes. Reaction conditions: 2-aminopyridine (0.50 mmol), aliphatic aldehyde (0.50 mmol), isocyanide (0.50 mmol), and HPW (0.01 mmol, 2 mol %) in EtOH (0.5 mL), under μw heating. The yields refer to isolated yields after column chromatography and the structures were confirmed by FTIR, NMR, and HRMS.

The use of aliphatic aldehydes in the GBB multicomponent reaction for the synthesis of imidazo[1,2-*a*]pyridines is not as usual, given that Schiff bases from aliphatic aldehydes are found to be less stable and readily polymerize in comparison to stable Schiff bases of aromatic aldehydes. Nonetheless, our protocol for the HPW-catalyzed GBB multicomponent reaction proved to be very effective for aliphatic aldehydes (Scheme 3). For instance, isovaleraldehyde proved to be quite reactive for a range of isocyanides (**5a**–**e**), furnishing the products in good to excellent yields (83–99%). Phenylacetaldehyde provided the expected product **5f** in a moderate yield. The imidazo[1,2-*a*]pyridines **5g**–**l** were obtained in good to excellent yields (74–99%) when isobutyraldehyde and cyclohexanecarboxaldehyde were used. Notably, even the less reactive isocyanides phenyl isocyanide and methyl isocyanoacetate reacted well and gave high product yields. Besides, the longer chain aldehyde heptaldehyde also furnished good results for the HPW-catalyzed GBB-3CR with yields greater than 63% (**5m**–**o**).

**Scheme 4:**
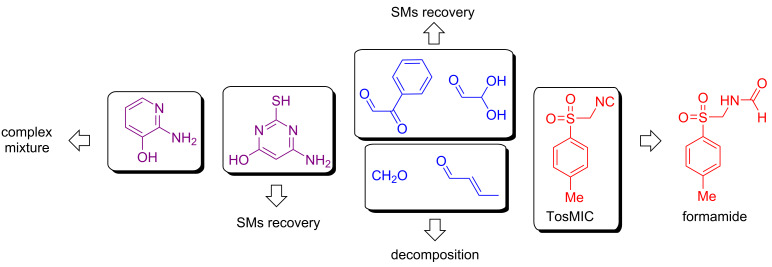
Unsuccessful substrates for the HPW-catalyzed GBB-3CR for the synthesis of imidazo[1,2-*a*]pyridines.

It is always important to point out the limitations of a given method and in our case, we did have some. Purification by column chromatography became necessary since product isolation by precipitation was not effective. The catalyst was impregnated in the isolated crude product, and its removal through a recrystallization step was not successful. The use of a liquid–liquid extraction work-up step proved less effective compared to column chromatography purification. Moreover, attempts to recover the catalyst were not reproducible. Unsuccessful substrates for these reactions were also detected (Scheme 4). The use of 2-amino-3-hydroxypyridine provided a complex mixture of products (^1^H and ^13^C NMR analysis). When 6-amino-2-thiouracil was used, only the starting materials were recovered. Regarding the aldehyde component, the use of glyoxals did not provide the desired products. Instead, the respective starting materials were almost quantitatively recovered from the column chromatography purification step. The use of very reactive aldehydes such as formaldehyde and crotonaldehyde also did not provide the GBB adduct, probably due to decomposition by the high temperature used in the protocol. Furthermore, the use of the less reactive TosMIC as isocyanide did not furnish the expected product; only the formamide product was isolated from the chromatographic column, which may be due to the lower nucleophilicity of this substrate.

**Scheme 5:**
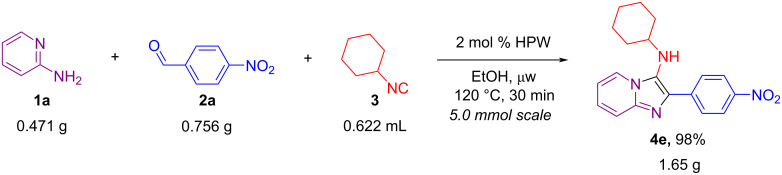
10-Fold scale-up of the HPW-catalyzed GBB reaction (5.0 mmol) between 2-aminopyridine (**1a**), 4-nitrobenzaldehyde (**2a**) and cyclohexyl isocyanide (**3**) in EtOH under μw heating.

The present HPW-catalyzed GBB reaction also worked very well when a 10-fold increase (5.0 mmol) in scale was applied (Scheme 5). In this case, the reaction was carried out using 5.0 mmol of each substrate and the product was obtained in gram scale in excellent yield (98%).

**Scheme 6:**
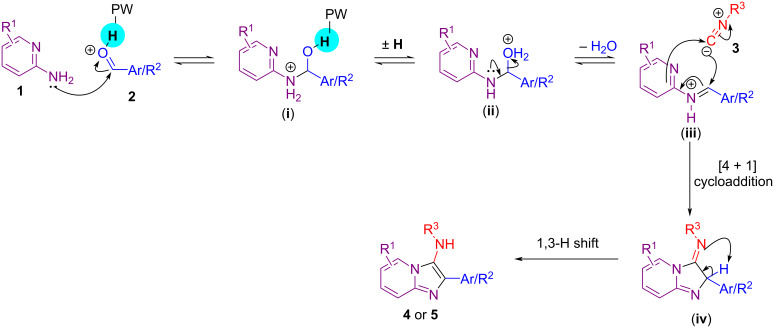
Plausible reaction mechanism for the HPW-catalyzed GBB reaction.

Based on a previous report from the literature [24] a plausible reaction mechanism is shown in Scheme 6. It involves the nucleophilic attack of the aminopyridine **1** to the HPW-activated carbonyl compound **2**, followed by iminium ion formation (iii) and [4 + 1] cycloaddition with the isocyanide. A 1,3-hydrogen shift yields the final products.

Our HPW-catalyzed GBB reaction has some advantages compared to several procedures reported in the literature for GBB reactions under μw and conventional heating conditions (Table 2), such as the use of a relatively inexpensive catalyst and a short reaction time. Furthermore, our protocol was applied to a broader range of aromatic/heteroaromatic or aliphatic aldehydes, 2-aminopyridines/2-aminothiazoles and isocyanides. Regarding some Green Chemistry metrics, our methodology proved superior at least considering E-factor and mass intensity. As can be seen in entry 6 of Table 2, our protocol showed a lower E-factor by far, which makes it a cleaner and environmentally friendly process. Through the mass intensity parameter, our procedure also showed a lower mass loss compared to the total raw material used to produce a given mass of product. The calculations of these parameters can be found in Supporting Information File 1.

**Table 2:** Comparison of reaction conditions and Green Chemistry metrics for the GBB reactions under μw and conventional heating conditions.^a^

Entry	Reaction conditions	Time (min)	Examples	Yields (%)^a^	E-factor^b^	Mass intensity	Ref.

1	CALB, EtOH, rt	18 h	11	0–91	12.79	10.75	[55]
2	20 mol % AgOAc, EG, 90 °C	120	23	72–92	11.01	12.01	[56]
3	5 mol % Gd(OTf)_3_, MeOH, μw, 150 °C	30	23	54–94	8.79	9.79	[28]
4	5 mol % ZnCl_2_, 1,4-dioxane, μw or reflux	60 or 300	17	9–78	7.21	8.21	[37]
5	10 mol % ZrCl_4_, PEG-400, μw, 140 °C	7	28	72–97	4.09	5.09	[30]
6	2 mol % HPW, EtOH, μw, 120 °C	30	68	23–99	2.61	3.61	this work

^a^Isolated yield. ^b^Excluding work-up and purification processes (extraction, column chromatography and/or recrystallization).

## Conclusion

In summary, we have developed a straightforward approach to the GBB reaction using HPW as a cheap catalyst in ethanol under μw heating for the synthesis of imidazo[1,2-*a*]pyridines. Although some minor limitations exist, this convenient environmentally benign methodology is broad in scope (68 products were obtained, many of them not previously reported), provides the heterobicyclic products in moderate to excellent yields (23–99%), with a low catalyst loading (2 mol %). The reactions are fast, and the method can be applied to a wide range of aromatic/heteroaromatic and aliphatic aldehydes. Furthermore, the isolation process is simple and there is no need for liquid–liquid extraction. In addition, our methodology showed superior Green Chemistry metrics as compared to some already reported methodologies.

## Supporting Information

File 1 Typical experimental procedures, FTIR, NMR and mass spectra of all compounds and GC metrics calculations.

## Data Availability

All data that supports the findings of this study is available in the published article and/or the supporting information to this article.
