# Supplementary material for: HPW-Catalyzed environmentally benign approach to imidazo[1,2-a]pyridines
Source: Beilstein J Org Chem. 2024 Mar 19;20:628–37. doi: 10.3762/bjoc.20.55 (PMC10964034; doi:10.3762/bjoc.20.55)
Supplement: File 1 — Typical experimental procedures, FTIR, NMR and mass spectra of all compounds and GC metrics calculations. [file Beilstein_J_Org_Chem-20-628-s001.pdf]

BEILSTEIN JOURNAL OF ORGANIC CHEMISTRY

Supporting Information

for

HPW-Catalyzed environmentally benign approach to imidazo[1,2-*a*]pyridines

Luan A. Martinho and Carlos Kleber Z. Andrade

Beilstein J. Org. Chem. **2024,** *20,* 628–637. doi:10.3762/bjoc.20.55

Typical experimental procedures, FTIR, NMR and mass spectra of all compounds and GC metrics calculations

License and Terms: This is a supporting information file under the terms of the Creative Commons Attribution License (https://creativecommons.org/licenses/by/4.0). Please note that the reuse, redistribution and reproduction in particular requires that the author(s) and source are credited and that individual graphics may be subject to special legal provisions.
The license is subject to the *Beilstein Journal of Organic Chemistry* terms and conditions: (https://www.beilstein-journals.org/bjoc/terms)

Table of contents

1. **Experimental section** S2

  1.1 **Materials and methods** S2

  1.2 **Experimental procedures** S2

    1.2.1 **General synthetic procedure for the HPW-catalyzed GBB reactions under microwave heating** S2

    1.2.2 **Synthesis and characterization of imidazo[1,2-*a*]pyridines** S2

2. **Calculations of green chemistry metrics** S32

3. **References** S36

4. **FTIR, ^{1}H and ^{13}C NMR, HRMS and HRMS/MS spectra for all compounds** S38

1. Experimental section

1.1 Materials and methods

Unless otherwise stated, all reagents and solvents were purchased from Sigma-Aldrich Merck and used without further purification. The aldehydes were distilled prior to their use. Cyclohexyl isocyanide and phenyl isocyanide were prepared according to literature procedures [1,2].

All microwave-mediated reactions were performed on a Biotage® Initiator+ (Uppsala, Sweden) microwave reactor using a sealed vessel with simultaneous cooling, and media stirring, temperature detection via an internal fiber optic probe. The reagents consumption and products formation were confirmed by thin-layer chromatography (TLC) with UV fluorescent silica gel Merck 60 F254 plates and visualized by treatment with a 10% solution of phosphomolybdic acid in ethanol (PMA), followed by heating. Column chromatography was performed on silica gel (Supelco, pore size 60 Å, 230–400 mesh particle size, 40–63 µm particle size) and mixtures of hexane/ethyl acetate were used as eluents as described for each molecule.

FTIR spectra were obtained on a Varian 640 spectrometer with TA DLaTGS as detector in the infrared region (4000–600 cm^{-1}) in attenuated total reflection (ATR) mode. The NMR spectra were recorded at 25 °C on a Bruker Avance 600 spectrometer at 600 MHz for ^{1}H and 151 MHz for ^{13}C with TMS as an internal standard for deuterated chloroform ($CDCl_3$) and dimethyl sulfoxide (DMSO-d_6) as solvents. HRMS experiments were performed on a Triple Tof 5600 Sciex by flow injection analysis using an Eksigent UltraLC 100 Sciex chromatograph set to a flow rate of 0.3 mL/min. A DuoSpray Ion Source (ESI) was used, and the MS spectra were acquired in positive mode, employing external calibration, in the range of 50–1000 Da and 0.1% (v/v) of formic acid in acetonitrile as solvent**.** The melting points were measured with capillary in the LOGEN Scientific equipment (LS III Plus) and were not corrected.

1.2 Experimental procedures

1.2.1 General synthetic procedure for the HPW-catalyzed GBB reactions under microwave heating

A Biotage microwave (µw) reaction vial of 0.5–2.0 mL containing a mixture of 2-aminopyridine (0.50 mmol), aldehyde (0.50 mmol), isocyanide (0.50 mmol), and phosphotungstic acid hydrate HPW (0.01 mmol, 2 mol %) in EtOH (0.50 mL) was introduced into in the cavity of a microwave reactor Biotage® Initiator+ and heated at 120 °C for 30 min under magnetic stirring. The reaction mixture was then cooled to room temperature, and reagents consumption was confirmed by TLC analysis (mixture of ethyl acetate/hexane). The reaction mixture was removed from the µw vial, concentrated under vacuum and the crude product was purified by silica gel column chromatography.

1.2.2 Synthesis and characterization of imidazo[1,2-*a*]pyridines

N-(*tert*-Butyl)-2-(4-nitrophenyl)imidazo[1,2-*a*]pyridin-3-amine (**4a**) [3]

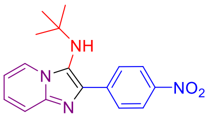

4a

4a was obtained from 2-aminopyridine (0.50 mmol, 0.047 g), 4-nitrobenzaldehyde (0.50 mmol, 0.076 g) and *tert*-butyl isocyanide (0.50 mmol, 0.057 mL), in 87% yield (0.135 g) as

an orange solid (m.p. 174-175 ºC) after silica gel column chromatography (20% ethyl acetate/hexane to 60% ethyl acetate/hexane). Rf = 0.46 (50% ethyl acetate/hexane).

FT-IR (ATR): ν 3305, 2935, 2854, 1724, 1657, 1597, 1510, 1473, 1435, 1362, 1342, 1329, 1282, 1255, 1109, 1012, 912, 856, 812, 773, 754, 739, 710, 690, 667, 646, 636, 625, 600 cm^{-1}.

^{1}H NMR (600 MHz, $CDCl_3$): δ 8.27 (s, 4H), 8.21 (dt, *J* = 6.8, 1.1 Hz, 1H), 7.58 (dt, *J* = 9.0, 1.1 Hz, 1H), 7.21 (ddd, *J* = 9.0, 6.8, 1.1 Hz, 1H), 6.85 (td, *J* = 6.8, 1.1 Hz, 1H), 3.11 (s, 1H), 1.09 (s, 9H) ppm.

^{13}C NMR (151 MHz, $CDCl_3$): δ 146.7, 142.3, 141.6, 136.8, 128.5, 125.3, 124.9, 123.6, 123.5, 117.6, 112.2, 56.9, 30.5 ppm.

HRMS (ESI-QTOF) *m/z* calculated for $C_{17}H_{19}N_4O_2^+$: 311.1503 $[M+H]^+$; found 311.1502.

2-(4-Bromophenyl)-*N*-(*tert*-butyl)imidazo[1,2-*a*]pyridin-3-amine (**4b**) [4]

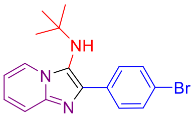

4b

4b was obtained from 2-aminopyridine (0.50 mmol, 0.047 g), 4-bromobenzaldehyde (0.50 mmol, 0.092 g) and *tert*-butyl isocyanide (0.50 mmol, 0.057 mL), in 59% yield (0.102 g) as a white solid (m.p. 140-142 ºC) after silica gel column chromatography (20% ethyl acetate/hexane to 40% ethyl acetate/hexane). Rf = 0.38 (30% ethyl acetate/hexane).

FT-IR (ATR): ν 3282, 2964, 1491, 1444, 1362, 1333, 1207, 1012, 833, 798, 752, 733, 661, 642, 611 cm^{-1}.

^{1}H NMR (600 MHz, $CDCl_3$): δ 8.23 (dt, *J* = 6.8, 1.1 Hz, 1H), 7.91–7.85 (m, 2H), 7.57 (dt, *J* = 9.0, 1.1 Hz, 1H), 7.56–7.53 (m, 2H), 7.19 (ddd, *J* = 9.0, 6.8, 1.1 Hz, 1H), 6.82 (td, *J* = 6.8, 1.1 Hz, 1H), 3.16 (s, 1H), 1.07 (s, 9H) ppm.

^{13}C NMR (151 MHz, $CDCl_3$): δ 141.8, 137.8, 133.6, 131.4, 129.7, 124.9, 123.6, 123.5, 121.6, 117.0, 111.8, 56.5, 30.4.ppm.

HRMS (ESI-QTOF) *m/z* calculated for $C_{17}H_{19}BrN_3^+$: 344.0757 $[M+H]^+$; found 344.0752.

4-(3-(*tert*-Butylamino)imidazo[1,2-*a*]pyridin-2-yl)phenol (**4c**) [5]

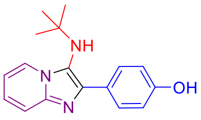

4c

4c was obtained from 2-aminopyridine (0.50 mmol, 0.047 g), 4-hydroxybenzaldehyde (0.50 mmol, 0.061 g) and *tert*-butyl isocyanide (0.50 mmol, 0.057 mL), in 43% yield (0.060 g) as a white solid (m.p. 263-264 ºC) after silica gel column chromatography (40% ethyl acetate/hexane to 70% ethyl acetate/hexane). Rf = 0.33 (50% ethyl acetate/hexane).

FT-IR (ATR): ν 3350, 2970, 1612, 1489, 1444, 1390, 1362, 1346, 1250, 1196, 1173, 1103, 841, 773, 750, 733, 636, 606 cm^{-1}.

^{1}H NMR (600 MHz, DMSO-d_6): δ 9.50 (s, 1H), 8.40 (dt, *J* = 6.8, 1.1 Hz, 1H), 7.99–7.94 (m, 2H), 7.45 (dt, *J* = 9.0, 1.1 Hz, 1H), 7.20 (ddd, *J* = 9.0, 6.8, 1.1 Hz, 1H), 6.89 (td, *J* = 6.8, 1.1 Hz, 1H), 6.83–6.78 (m, 2H), 4.56 (s, 1H), 1.00 (s, 9H) ppm.

^{13}C NMR (151 MHz, DMSO-d_6): δ 157.2, 140.9, 138.2, 129.5, 126.1, 124.7, 124.6, 123.4, 116.4, 115.2, 111.7, 56.1, 30.5 ppm.

HRMS (ESI-QTOF) *m/z* calculated for $C_{17}H_{20}N_3O^+$: 282.1601 $[M+H]^+$; found 282.1597.

N-(*tert*-Butyl)-2-(4-(dimethylamino)phenyl)imidazo[1,2-*a*]pyridin-3-amine (**4d**) [6]

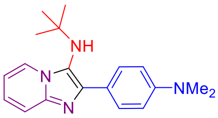

4d

4d was obtained from 2-aminopyridine (0.50 mmol, 0.047 g), 4-(dimethylamino)benzaldehyde (0.50 mmol, 0.075 g) and *tert*-butyl isocyanide (0.50 mmol, 0.057 mL), in 57% yield (0.088 g) as a white solid (m.p. 182-183 ºC) after silica gel column chromatography (40% ethyl acetate/hexane to 70% ethyl acetate/hexane). Rf = 0.34 (50% ethyl acetate/hexane).

FT-IR (ATR): ν 3282, 2970, 1616, 1516, 1441, 1383, 1335, 1205, 1167, 1130, 1057, 947, 825, 754, 739, 650, 633, 607 cm^{-1}.

^1H NMR (600 MHz, $CDCl_3$): δ 8.21 (dt, *J* = 6.8, 1.2 Hz, 1H), 7.83–7.78 (m, 2H), 7.53 (dt, *J* = 9.0, 1.2 Hz, 1H), 7.10 (ddd, *J* = 9.0, 6.8, 1.2 Hz, 1H), 6.80–6.76 (m, 2H), 6.74 (td, *J* = 6.8, 1.2 Hz, 1H), 3.10 (s, 1H), 2.99 (s, 6H), 1.06 (s, 9H) ppm.

^{13}C NMR (151 MHz, $CDCl_3$): δ 149.8, 141.7, 139.6, 128.9, 123.7, 123.3, 123.0, 122.4, 116.7, 112.1, 111.1, 56.3, 40.5, 30.4 ppm.

HRMS (ESI-QTOF) *m/z* calculated for $C_{19}H_{25}N_4^+$: 309.2074 $[M+H]^+$; found 309.2068.

N-Cyclohexyl-2-(4-nitrophenyl)imidazo[1,2-*a*]pyridin-3-amine (**4e**) [6]

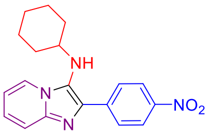

4e

4e was obtained from 2-aminopyridine (0.50 mmol, 0.047 g), 4-nitrobenzaldehyde (0.50 mmol, 0.076 g) and cyclohexyl isocyanide (0.50 mmol, 0.062 mL), in 99% yield (0.166 g) as an orange solid (m.p. 205-207 ºC) after silica gel column chromatography (30% ethyl acetate/hexane to 60% ethyl acetate/hexane). Rf = 0.29 (30% ethyl acetate/hexane).

FT-IR (ATR): ν 3240, 2927, 2852, 1597, 1510, 1335, 1230, 1113, 862, 752, 739, 648 cm^{-1}.

^1H NMR (600 MHz, $CDCl_3$): δ 88.37–8.32 (m, 2H), 8.30–8.24 (m, 2H), 8.11 (dt, *J* = 6.8, 1.3 Hz, 1H), 7.60 (d, *J* = 9.0 Hz, 1H), 7.23 (ddd, *J* = 9.0, 6.8, 1.3 Hz, 1H), 6.88 (td, *J* = 6.8, 1.3 Hz, 1H), 3.31 (d, *J* = 5.1 Hz, 1H), 3.02–2.94 (m, 1H), 1.87–1.81 (m, 2H), 1.76–1.70 (m, 2H), 1.64–1.58 (m, 1H), 1.34–1.25 (m, 2H), 1.25–1.12 (m, 3H) ppm.

^{13}C NMR (151 MHz, $CDCl_3$): δ 146.6, 141.6, 140.3, 133.6, 127.3, 126.7, 125.6, 123.8, 122.9, 117.4, 112.7, 57.1, 34.3, 25.6, 24.8 ppm.

HRMS (ESI-QTOF) *m/z* calculated for $C_{19}H_{21}N_4O_2^+$: 337.1659 $[M+H]^+$; found 337.1657.

4-(3-(Cyclohexylamino)imidazo[1,2-*a*]pyridin-2-yl)benzonitrile (**4f**) [7]

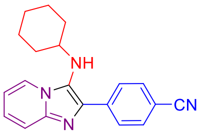

4f

4f was obtained from 2-aminopyridine (0.50 mmol, 0.047 g), 4-formylbenzonitrile (0.50 mmol, 0.066 g) and cyclohexyl isocyanide (0.50 mmol, 0.062 mL), in 89% yield (0.140 g) as a greenish solid (m.p. 166-168 ºC) after silica gel column chromatography (30% ethyl acetate/hexane to 50% ethyl acetate/hexane). Rf = 0.39 (50% ethyl acetate/hexane).

FT-IR (ATR): ν 3303, 2933, 2854, 2224, 1604, 1576, 1448, 1369, 1340, 1230, 1203, 1097, 914, 837, 748, 733, 658, 640, 625 cm^{-1}.

^{1}H NMR (600 MHz, $CDCl_3$): δ 8.28–8.23 (m, 2H), 8.08 (dt, *J* = 6.8, 1.2 Hz, 1H), 7.71–7.67 (m, 2H), 7.56 (dd, *J* = 9.1, 1.2 Hz, 1H), 7.20 (ddd, *J* = 9.1, 6.8, 1.2 Hz, 1H), 6.85 (td, *J* = 6.8, 1.2 Hz, 1H), 3.20 (d, *J* = 5.0 Hz, 1H), 3.00–2.92 (m, 1H), 1.85–1.79 (m, 2H), 1.75–1.69 (m, 2H), 1.32–1.23 (m, 2H), 1.24–1.07 (m, 3H) ppm.

^{13}C NMR (151 MHz, $CDCl_3$): δ 141.7, 138.7, 134.2, 132.2, 127.2, 126.2, 125.2, 122.8, 119.2, 117.5, 112.4, 110.4, 57.0, 34.2, 25.6, 24.8 ppm.

HRMS (ESI-QTOF) *m/z* calculated for $C_{20}H_{21}N_4^+$: 317.1761 $[M+H]^+$; found 317.1761.

2-(4-Bromophenyl)-*N*-cyclohexylimidazo[1,2-*a*]pyridin-3-amine (**4g**) [8]

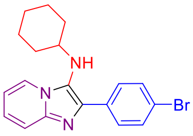

4g

4g was obtained from 2-aminopyridine (0.50 mmol, 0.047 g), 4-bromobenzaldehyde (0.50 mmol, 0.092 g) and cyclohexyl isocyanide (0.50 mmol, 0.062 mL), in 91% yield (0.169 g) as a white solid (m.p. 154-155 ºC) after silica gel column chromatography (20% ethyl acetate/hexane to 40% ethyl acetate/hexane). Rf = 0.37 (30% ethyl acetate/hexane).

FT-IR (ATR): ν 3292, 3278, 3228, 2929, 1657, 1556, 1489, 1446, 1365, 1336, 1227, 1074, 1009, 829, 750, 735, 656, 634, 600 cm^{-1}.

^{1}H NMR (600 MHz, $CDCl_3$): δ 8.13 (dt, *J* = 6.8, 1.2 Hz, 1H), 8.00–7.95 (m, 2H), 7.60 (dt, *J* = 8.8, 1.2 Hz, 1H), 7.56–7.50 (m, 2H), 7.21 (ddd, *J* = 8.8, 6.8, 1.2 Hz, 1H), 6.86 (td, *J* = 6.8, 1.2 Hz, 1H), 3.42 (s, 1H), 2.97–2.89 (m, 1H), 1.84–1.75 (m, 2H), 1.72–1.65 (m, 2H), 1.60–1.56 (m, 1H), 1.29–1.20 (m, 2H), 1.20–1.09 (m, 3H) ppm.

^{13}C NMR (151 MHz, $CDCl_3$): δ 140.7, 131.7, 128.6, 128.4, 125.6, 125.1, 123.0, 121.8, 116.6, 112.6, 56.8, 34.2, 25.7, 24.8 ppm.

HRMS (ESI-QTOF) *m/z* calculated for $C_{19}H_{21}BrN_3^+$: 370.0913 $[M+H]^+$; found 370.0914.

2-(4-Chlorophenyl)-*N*-cyclohexylimidazo[1,2-*a*]pyridin-3-amine (**4h**) [9]

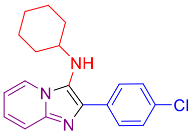

4h

4h was obtained from 2-aminopyridine (0.50 mmol, 0.047 g), 4-chlorobenzaldehyde (0.50 mmol, 0.070 g) and cyclohexyl isocyanide (0.50 mmol, 0.062 mL), in 97% yield (0.158 g) as a white solid (m.p. 179-180 ºC) after silica gel column chromatography (20% ethyl acetate/hexane to 40% ethyl acetate/hexane). Rf = 0.48 (30% ethyl acetate/hexane).

FT-IR (ATR): v 3284, 3226, 2931, 2854, 1558, 1491, 1446, 1387, 1367, 1336, 1228, 1203, 1144, 1093, 1014, 912, 837, 750, 737, 600 cm^{-1}.

^{1}H NMR (600 MHz, $CDCl_3$): δ 8.12 (dt, J = 6.8, 1.2 Hz, 1H), 8.06–8.00 (m, 2H), 7.58 (dt, J = 9.0, 1.2 Hz, 1H), 7.41–7.36 (m, 2H), 7.19 (ddd, J = 9.0, 6.8, 1.2 Hz, 1H), 6.84 (td, J = 6.8, 1.2 Hz, 1H), 3.31 (s, 1H), 2.97–2.89 (m, 1H), 1.83–1.76 (m, 2H), 1.60–1.54 (m, 1H), 1.37–1.19 (m, 2H), 1.20–1.11 (m, 3H) ppm.

^{13}C NMR (151 MHz, $CDCl_3$): δ 141.0, 133.4, 128.7, 128.3, 125.2, 125.0, 122.9, 116.8, 112.3, 56.8, 34.2, 25.7, 24.8 ppm.

HRMS (ESI-QTOF) *m/z* calculated for $C_{19}H_{21}ClN_3^+$: 326.1419 [M+H]$^+$; found 326.1420.

N-Cyclohexyl-2-(4-fluorophenyl)imidazo[1,2-*a*]pyridin-3-amine (**4i**) [10]

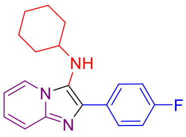

4i

4i was obtained from 2-aminopyridine (0.50 mmol, 0.047 g), 4-fluorobenzaldehyde (0.50 mmol, 0.054 mL) and cyclohexyl isocyanide (0.50 mmol, 0.062 mL), in 92% yield (0.143 g) as a white solid (m.p. 149-150 °C) after silica gel column chromatography (10% ethyl acetate/hexane to 40% ethyl acetate/hexane). Rf = 0.43 (30% ethyl acetate/hexane).

FT-IR (ATR): ν 3228, 2929, 2854, 1570, 1504, 1446, 1385, 1365, 1335, 1219, 1153, 1090, 839, 800, 752, 735, 634, 604 cm^{-1}.

^{1}H NMR (600 MHz, $CDCl_3$): δ 8.10 (dt, J = 6.8, 1.1 Hz, 1H), 8.08–8.01 (m, 2H), 7.56 (dt, J = 9.0, 1.1 Hz, 1H), 7.17–7.10 (m, 3H), 6.81 (td, J = 6.8, 1.1 Hz, 1H), 3.16 (s, 1H), 2.97–2.90 (m, 1H), 1.83–1.76 (m, 2H), 1.72–1.66 (m, 2H), 1.60–1.53 (m, 1H), 1.29–1.19 (m, 2H), 1.19–1.10 (m, 3H) ppm.

^{13}C NMR (151 MHz, $CDCl_3$): δ 162.3 (d, J = 246.7 Hz), 141.3, 135.4, 130.1, 128.8 (d, J = 7.8 Hz), 124.6, 124.5, 122.8, 117.1, 115.4 (d, J = 21.4 Hz), 112.0, 56.8, 34.2, 25.7, 24.8 ppm.

HRMS (ESI-QTOF) *m/z* calculated for $C_{19}H_{21}FN_3^+$: 310.1714 [M+H]$^+$; found 310.1710.

N-Cyclohexyl-2-(4-(diethylamino)phenyl)imidazo[1,2-*a*]pyridin-3-amine (**4j**)

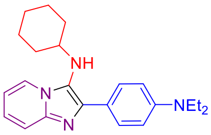

4j

4j was obtained from 2-aminopyridine (0.50 mmol, 0.047 g), 4-(diethylamino)benzaldehyde (0.50 mmol, 0.089 g) and cyclohexyl isocyanide (0.50 mmol, 0.062 mL), in 75% yield (0.136 g) as a light brown solid (m.p. 135-136 °C) after silica gel column chromatography (20% ethyl acetate/hexane to 50% ethyl acetate/hexane). Rf = 0.28 (30% ethyl acetate/hexane).

FT-IR (ATR): ν 3282, 2972, 2927, 2852, 1612, 1552, 1512, 1444, 1404, 1344, 1263, 1200, 1155, 1080, 814, 754, 741, 644, 606 cm^{-1}.

^{1}H NMR (600 MHz, $CDCl_3$): δ 8.12 (dt, J = 6.8, 1.2 Hz, 1H), 7.97–7.91 (m, 2H), 7.55 (dt, J = 9.0, 1.2 Hz, 1H), 7.10 (ddd, J = 9.0, 6.8, 1.2 Hz, 1H), 6.80–6.72 (m, 3H), 3.42 (q, J = 7.1 Hz, 4H), 3.14 (d, J = 4.4 Hz, 1H), 3.07–2.99 (m, 1H), 1.88–1.81 (m, 2H), 1.75–1.68 (m, 2H), 1.63–1.56 (m, 1H), 1.35–1.26 (m, 2H), 1.22 (t, J = 7.1 Hz, 6H), 1.25–1.14 (m, 3H) ppm.

^{13}C NMR (151 MHz, $CDCl_3$): δ 147.2, 141.1, 136.8, 128.1, 123.6, 123.3, 122.6, 121.0, 116.6, 111.7, 111.3, 56.9, 44.3, 34.2, 25.8, 24.9, 12.7 ppm.

HRMS (ESI-QTOF) *m/z* calculated for $C_{23}H_{31}N_4^+$: 363.2543 $[M+H]^+$; found 363.2547.

N-(*tert*-Butyl)-2-(2-fluorophenyl)imidazo[1,2-*a*]pyridin-3-amine (**4k**)

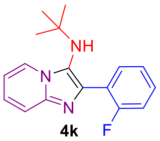

4k

4k was obtained from 2-aminopyridine (0.50 mmol, 0.047 g), 2-fluorobenzaldehyde (0.50 mmol, 0.053 mL) and *tert*-butyl isocyanide (0.50 mmol, 0.057 mL), in 84% yield (0.119 g) as a yellowish white solid (m.p. 78-79 ºC) after silica gel column chromatography (10% ethyl acetate/hexane to 40% ethyl acetate/hexane). Rf = 0.37 (30% ethyl acetate/hexane).

FT-IR (ATR): ν 3315, 2972, 1554, 1497, 1473, 1452, 1389, 1342, 1265, 1219, 1107, 802, 752, 742, 706, 660, 607 cm^{-1}.

^{1}H NMR (600 MHz, $CDCl_3$): δ 8.38–8.30 (m, 1H), 7.93 (td, *J* = 7.6, 1.9 Hz, 1H), 7.57 (dt, *J* = 9.0, 1.2 Hz, 1H), 7.38–7.32 (m, 1H), 7.31–7.24 (m, 1H), 7.20–7.11 (m, 2H), 6.80 (tt, *J* = 6.8, 1.2 Hz, 1H), 3.33 (d, *J* = 8.5 Hz, 1H), 0.99 (s, 9H) ppm.

^{13}C NMR (151 MHz, $CDCl_3$): δ 159.8 (d, *J* = 244.3 Hz), 142.5, 134.4, 131.8 (d, *J* = 3.9 Hz), 129.3 (d, *J* = 8.5 Hz), 125.6, 124.7 (d, *J* = 3.2 Hz), 124.2, 123.8, 123.3 (d, *J* = 14.5 Hz), 117.3, 115.5 (d, *J* = 23.1 Hz), 111.3, 56.0, 29.8.ppm.

HRMS (ESI-QTOF) *m/z* calculated for $C_{17}H_{19}FN_3^+$: 284.1558 $[M+H]^+$; found 284.1556.

2-(2-Bromophenyl)-*N*-(*tert*-butyl)imidazo[1,2-*a*]pyridin-3-amine (**4l**) [11]

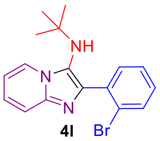

4l

4l was obtained from 2-aminopyridine (0.50 mmol, 0.047 g), 2-bromobenzaldehyde (0.50 mmol, 0.058 mL) and *tert*-butyl isocyanide (0.50 mmol, 0.057 mL), in 74% yield (0.128 g) as a yellowish white solid (m.p. 148-150 ºC) after silica gel column chromatography (20% ethyl acetate/hexane to 50% ethyl acetate/hexane). Rf = 0.56 (50% ethyl acetate/hexane).

FT-IR (ATR): ν 3234, 2964, 1630, 1554, 1502, 1470, 1433, 1385, 1356, 1340, 1225, 1024, 812, 754, 739, 702, 679, 656, 617 cm^{-1}.

^{1}H NMR (600 MHz, $CDCl_3$): δ 8.34 (dt, *J* = 6.8, 1.2 Hz, 1H), 7.69 (dd, *J* = 7.6, 1.7 Hz, 1H), 7.66 (dd, *J* = 8.1, 1.2 Hz, 1H), 7.58 (dt, *J* = 9.0, 1.2 Hz, 1H), 7.43 (td, *J* = 7.6, 1.2 Hz, 1H), 7.29–7.23 (m, 1H), 7.20 (ddd, *J* = 9.0, 6.8, 1.2 Hz, 1H), 6.83 (td, *J* = 6.8, 1.2 Hz, 1H), 3.24 (s, 1H), 0.95 (s, 9H) ppm.

^{13}C NMR (151 MHz, $CDCl_3$): δ 141.9, 139.0, 136.8, 133.1, 132.6, 129.4, 127.4, 124.6, 124.3, 123.7, 122.7, 122.7, 117.4, 111.5, 55.7, 30.0 ppm.

HRMS (ESI-QTOF) *m/z* calculated for $C_{17}H_{19}BrN_3^+$: 344.0757 $[M+H]^+$; found 344.0764.

N-(*tert*-Butyl)-2-(2-nitrophenyl)imidazo[1,2-*a*]pyridin-3-amine (**4m**) [11]

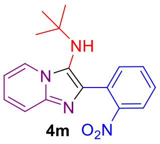

4m

4m was obtained from 2-aminopyridine (0.50 mmol, 0.047 g), 2-nitrobenzaldehyde (0.50 mmol, 0.076 g) and *tert*-butyl isocyanide (0.50 mmol, 0.057 mL), in 77% yield (0.120 g) as a light-yellow solid (m.p. 159-161 ºC. 171°C) after silica gel column chromatography (20% ethyl acetate/hexane to 50% ethyl acetate/hexane). Rf = 0.52 (50% ethyl acetate/hexane).

FT-IR (ATR): ν 3246, 1524, 1441, 1389, 1356, 1338, 1275, 1223, 1201, 860, 810, 783, 750, 721, 698, 652, 611 cm^{-1}.

^{1}H NMR (600 MHz, $CDCl_3$): δ 8.22–8.18 (m, 1H), 7.92 (dd, *J* = 8.2, 1.3 Hz, 1H), 7.82 (dd, *J* = 7.5, 1.5 Hz, 1H), 7.66 (tdd, *J* = 7.5, 1.3, 0.5 Hz, 1H), 7.56 (dt, *J* = 9.0, 1.3 Hz, 1H), 7.50 (tdd, *J* = 7.5, 1.5, 0.5 Hz, 1H), 7.21–7.16 (m, 1H), 6.83 (td, *J* = 6.9, 1.3 Hz, 1H), 2.74 (s, 1H), 0.95 (s, 9H) ppm.

^{13}C NMR (151 MHz, $CDCl_3$): δ 149.4, 142.2, 132.9, 132.5, 130.0, 128.5, 124.7, 124.3, 123.4, 117.6, 112.0, 55.6, 30.0 ppm.

HRMS (ESI-QTOF) *m/z* calculated for $C_{17}H_{19}N_4O_2^+$: 311.1503 $[M+H]^+$; found 311.1505.

N-Cyclohexyl-2-(2-fluorophenyl)imidazo[1,2-*a*]pyridin-3-amine (**4n**) [3]

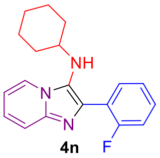

4n

4n was obtained from 2-aminopyridine (0.50 mmol, 0.047 g), 2-fluorobenzaldehyde (0.50 mmol, 0.053 mL) and cyclohexyl isocyanide (0.50 mmol, 0.062 mL), in 97% yield (0.155 g) as a yellow oil after silica gel column chromatography (10% ethyl acetate/hexane to 40% ethyl acetate/hexane). Rf = 0.43 (30% ethyl acetate/hexane).

FT-IR (ATR): ν 3359, 2920, 2854, 1570, 1502, 1448, 1398, 1350, 1265, 1203, 1149, 1107, 1070, 933, 895, 839, 796, 750, 704, 604 cm^{-1}.

^{1}H NMR (600 MHz, $CDCl_3$): δ 8.23 (dd, *J* = 6.8, 1.4 Hz, 1H), 7.99 (td, *J* = 7.6, 1.8 Hz, 1H), 7.74 (d, *J* = 9.0 Hz, 1H), 7.43–7.36 (m, 1H), 7.33 (td, *J* = 7.5, 1.4 Hz, 1H), 7.31–7.24 (m, 1H), 7.23–7.18 (m, 1H), 6.92 (t, *J* = 6.8 Hz, 1H), 3.50 (t, *J* = 8.2 Hz, 1H), 2.75–2.71 (m, 1H), 1.78–1.72 (m, 2H), 1.67–1.61 (m, 2H), 1.56–1.49 (m, 1H), 1.20–1.03 (m, 5H) ppm.

^{13}C NMR (151 MHz, $CDCl_3$): δ 159.5 (d, *J* = 245.2 Hz), 141.3, 131.6 (d, *J* = 3.8 Hz), 129.8 (d, *J* = 8.3 Hz), 127.0, 125.3, 125.0 (d, *J* = 3.1 Hz), 123.1, 116.8, 115.7 (d, *J* = 23.1 Hz), 112.5, 56.7, 34.0, 25.6, 24.8 ppm.

HRMS (ESI-QTOF) *m/z* calculated for $C_{19}H_{21}FN_3^+$: 310.1714 $[M+H]^+$; found 310.1718.

2-(2-Bromophenyl)-*N*-cyclohexylimidazo[1,2-*a*]pyridin-3-amine (**4o**) [7]

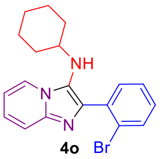

4o

4o was obtained from 2-aminopyridine (0.50 mmol, 0.047 g), 2-bromobenzaldehyde (0.50 mmol, 0.058 mL) and cyclohexyl isocyanide (0.50 mmol, 0.062 mL), in 99% yield (0.184 g) as a brown oil after silica gel column chromatography (20% ethyl acetate/hexane to 60% ethyl acetate/hexane). Rf = 0.48 (50% ethyl acetate/hexane).

FT-IR (ATR): ν 2927, 2852, 1736, 1558, 1504, 1448, 1348, 1234, 1024, 752, 700, 609 cm^{-1}.

^{1}H NMR (600 MHz, $CDCl_3$): δ 8.21 (dt, *J* = 6.8, 1.2 Hz, 1H), 7.68 (dd, *J* = 8.1, 1.2 Hz, 1H), 7.67–7.63 (m, 2H), 7.43 (td, *J* = 7.5, 1.2 Hz, 1H), 7.29 (td, *J* = 7.5, 1.7 Hz, 1H), 7.23 (ddd, *J* = 9.0, 6.8, 1.2 Hz, 1H), 6.89 (td, *J* = 6.8, 1.2 Hz, 1H), 3.32 (d, *J* = 7.0 Hz, 1H), 2.74–2.65 (m, 1H), 1.72–1.65 (m, 2H), 1.62–1.56 (m, 2H), 1.52–1.46 (m, 1H), 1.18–0.97 (m, 5H) ppm.

^{13}C NMR (151 MHz, $CDCl_3$): δ 141.0, 135.7, 135.2, 132.8, 132.7, 129.7, 127.5, 126.0, 124.6, 123.0, 122.9, 117.2, 112.2, 56.4, 33.8, 25.6, 24.5 ppm.

HRMS (ESI-QTOF) *m/z* calculated for $C_{19}H_{21}BrN_3^+$: 370.0913 [M+H]$^+$; found 370.0907.

2-(2-Chlorophenyl)-*N*-cyclohexylimidazo[1,2-*a*]pyridin-3-amine (**4p**) [7]

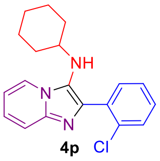

4p

4p was obtained from 2-aminopyridine (0.50 mmol, 0.047 g), 2-chlorobenzaldehyde (0.50 mmol, 0.056 mL) and cyclohexyl isocyanide (0.50 mmol, 0.062 mL), in 98% yield (0.160 g) as a brown oil after silica gel column chromatography (20% ethyl acetate/hexane to 40% ethyl acetate/hexane). Rf = 0.37 (30% ethyl acetate/hexane).

FT-IR (ATR): ν 2927, 2980, 2852, 1560, 1504, 1450, 1348, 1263, 1230, 1194, 1041, 752, 737 cm^{-1}.

^{1}H NMR (600 MHz, $CDCl_3$): δ 8.18 (dt, *J* = 6.8, 1.2 Hz, 1H), 7.69 (dd, *J* = 7.6, 1.8 Hz, 1H), 7.64–7.59 (m, 1H), 7.51–7.45 (m, 1H), 7.41–7.30 (m, 2H), 7.22–7.16 (m, 1H), 6.85 (tt, *J* = 6.8, 1.2 Hz, 1H), 3.30 (d, *J* = 6.8 Hz, 1H), 2.70–2.64 (m, 1H), 1.70–1.64 (m, 2H), 1.61–1.54 (m, 2H), 1.51–1.44 (m, 1H), 1.13–0.97 (m, 5H) ppm.

^{13}C NMR (151 MHz, $CDCl_3$): δ 141.3, 134.3, 133.4, 132.6, 129.6, 129.4, 127.0, 126.4, 124.4, 123.0, 117.3, 112.0, 56.4, 33.9, 25.6, 24.6 ppm.

HRMS (ESI-QTOF) *m/z* calculated for $C_{19}H_{21}ClN_3^+$: 326.1419 [M+H]$^+$; found 326.1420.

N-Cyclohexyl-2-(2-nitrophenyl)imidazo[1,2-*a*]pyridin-3-amine (**4q**) [12]

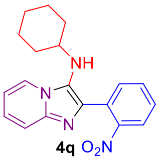

4q

4q was obtained from 2-aminopyridine (0.50 mmol, 0.047 g), 2-nitrobenzaldehyde (0.50 mmol, 0.076 g) and cyclohexyl isocyanide (0.50 mmol, 0.062 mL), in 87% yield (0.146 g) as a dark-brown oil after silica gel column chromatography (30% ethyl acetate/hexane to 60% ethyl acetate/hexane). Rf = 0.40 (50% ethyl acetate/hexane).

FT-IR (ATR): ν 3302, 2927, 1529, 1444, 1358, 1277, 1203, 1144, 912, 858, 781, 741, 700, 631 cm^{-1}.

^{1}H NMR (600 MHz, $CDCl_3$): δ 8.12 (d, *J* = 6.8 Hz, 1H), 7.95 (dd, *J* = 8.2, 1.1 Hz, 1H), 7.83 (dd, *J* = 7.6, 1.3 Hz, 1H), 7.69 (td, *J* = 7.6, 1.3 Hz, 1H), 7.61 (d, *J* = 9.0 Hz, 1H), 7.56–7.50 (m, 1H), 7.22 (ddd, *J* = 9.0, 6.8, 1.1 Hz, 1H), 6.88 (td, *J* = 6.8, 1.1 Hz, 1H), 2.92 (d, *J* = 5.0 Hz, 1H), 2.80–2.73 (m, 1H), 1.70–1.64 (m, 2H), 1.63–1.56 (m, 2H), 1.51–1.45 (m, 1H), 1.16–0.98 (m, 5H) ppm.

^{13}C NMR (151 MHz, $CDCl_3$): δ 149.4, 141.5, 133.0, 132.7, 132.5, 128.7, 126.1, 124.9, 124.4, 122.9, 117.5, 112.4, 56.3, 33.8, 25.6, 24.5 ppm.

HRMS (ESI-QTOF) *m/z* calculated for $C_{19}H_{21}N_4O_2^+$: 337.1659 $[M+H]^+$; found 337.1673.

2-(3-(Cyclohexylamino)imidazo[1,2-*a*]pyridin-2-yl)phenol (**4r**) [8]

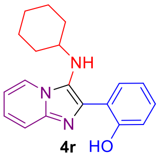

4r

4r was obtained from 2-aminopyridine (0.50 mmol, 0.047 g), salicylaldehyde (0.50 mmol, 0.052 mL) and cyclohexyl isocyanide (0.50 mmol, 0.062 mL), in 87% yield (0.133 g) as a white solid (m.p. 131-133 ºC) after silica gel column chromatography (5% ethyl acetate/hexane to 30% ethyl acetate/hexane). Rf = 0.43 (10% ethyl acetate/hexane).

FT-IR (ATR): ν 3325, 2927, 2848, 1572, 1504, 1448, 1367, 1294, 1228, 1190, 1147, 1109, 1080, 800, 752, 708, 669, 623 cm^{-1}.

^1H NMR (600 MHz, $CDCl_3$): δ 8.23 (d, *J* = 6.8 Hz, 1H), 8.01 (dd, *J* = 8.0, 1.6 Hz, 1H), 7.53 (dt, *J* = 9.0, 1.2 Hz, 1H), 7.28–7.16 (m, 2H), 7.04 (dd, *J* = 8.0, 1.2 Hz, 1H), 6.97–6.85 (m, 2H), 3.38 (s, 1H), 3.06–2.99 (m, 1H), 1.87–1.81 (m, 2H), 1.76–1.67 (m, 2H), 1.64–1.59 (m, 1H), 1.39–1.27 (m, 2H), 1.24–1.15 (m, 3H) ppm.

^{13}C NMR (151 MHz, $CDCl_3$): δ 157.2, 139.2, 129.3, 126.6, 123.6, 122.7, 118.8, 117.7, 116.1, 112.7, 57.0, 34.0, 25.7, 24.8 ppm.

HRMS (ESI-QTOF) *m/z* calculated for $C_{19}H_{22}N_3O^+$: 308.1757 $[M+H]^+$; found 308.1756.

N-(*tert*-Butyl)-2-(3,4-dimethoxyphenyl)imidazo[1,2-*a*]pyridin-3-amine (**4s**) [13]

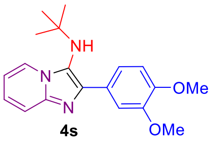

4s

4s was obtained from 2-aminopyridine (0.50 mmol, 0.047 g), 3,4-dimethoxybenzaldehyde (0.50 mmol, 0.083 g) and *tert*-butyl isocyanide (0.50 mmol, 0.057 mL), in 87% yield (0.163 g) as an orange oil after silica gel column chromatography (30% ethyl acetate/hexane to 90% ethyl acetate/hexane). Rf = 0.24 (50% ethyl acetate/hexane).

FT-IR (ATR): ν 2968, 1587, 1508, 1464, 1389, 1365, 1255, 1230, 1174, 1138, 1026, 893, 814, 756, 735, 634 cm^{-1}.

^1H NMR (600 MHz, $CDCl_3$): δ 8.25 (dt, *J* = 6.8, 1.2 Hz, 1H), 7.61 (d, *J* = 2.0 Hz, 1H), 7.59 (d, *J* = 9.0, 1.2 Hz, 1H), 7.48 (dd, *J* = 8.3, 2.0 Hz, 1H), 7.17 (ddd, *J* = 9.0, 6.8, 1.2 Hz, 1H), 6.92 (d, *J* = 8.3 Hz, 1H), 6.82 (td, *J* = 6.8, 1.2 Hz, 1H), 4.01 (s, 3H), 3.94 (s, 3H), 3.24 (s, 1H), 1.08 (s, 9H) ppm.

^{13}C NMR (151 MHz, $CDCl_3$): δ 148.9, 148.6, 129.5, 124.6, 123.5, 123.1, 120.6, 116.7, 111.7, 111.5, 110.8, 56.4, 56.1, 55.8, 30.4 ppm.

HRMS (ESI-QTOF) *m/z* calculated for $C_{19}H_{24}N_3O_2^+$: 326.1863 $[M+H]^+$; found 326.1855.

N-Cyclohexyl-2-(3,4-dimethoxyphenyl)imidazo[1,2-*a*]pyridin-3-amine (**4t**) [13]

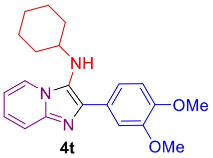

4t

4t was obtained from 2-aminopyridine (0.50 mmol, 0.047 g), 3,4-dimethoxybenzaldehyde (0.50 mmol, 0.083 g) and cyclohexyl isocyanide (0.50 mmol, 0.062 mL), in 98% yield (0.172 g) as an orange oil after silica gel column chromatography (30% ethyl acetate/hexane to 70% ethyl acetate/hexane). Rf = 0.23 (50% ethyl acetate/hexane).

FT-IR (ATR): ν 3259, 2929, 2850, 1585, 1508, 1450, 1423, 1363, 1257, 1227, 1200, 1173, 1136, 1066, 1026, 895, 808, 750, 735, 629, 607 cm^{-1}.

^{1}H NMR (600 MHz, $CDCl_3$): δ 8.12 (dt, *J* = 6.8, 1.3 Hz, 1H), 7.72 (d, *J* = 1.9 Hz, 1H), 7.59 (dd, *J* = 8.4, 1.9 Hz, 1H), 7.57 (d, *J* = 9.0 Hz, 1H), 7.18–7.12 (m, 1H), 6.92 (d, *J* = 8.4 Hz, 1H), 6.83–6.78 (m, 1H), 4.01 (s, 3H), 3.93 (s, 3H), 3.33 (s, 1H), 3.03–2.96 (m, 1H), 1.84–1.79 (m, 2H), 1.73–1.66 (m, 2H), 1.61–1.55 (m, 1H), 1.31–1.22 (m, 2H), 1.22–1.10 (m, 3H) ppm.

^{13}C NMR (151 MHz, $CDCl_3$): δ 149.0, 148.6, 140.8, 135.6, 128.7, 128.6, 126.4, 124.6, 124.3, 122.8, 119.4, 116.6, 112.0, 111.0, 110.5, 56.8, 56.1, 55.9, 34.2, 25.7, 24.8 ppm.

HRMS (ESI-QTOF) *m/z* calculated for $C_{21}H_{26}N_3O_2^+$: 352.2020 [M+H]$^+$; found 352.2013.

N-Cyclohexyl-2-(3-nitrophenyl)imidazo[1,2-*a*]pyridin-3-amine (**4u**) [14]

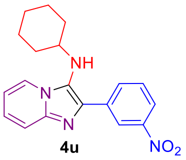

4u

4u was obtained from 2-aminopyridine (0.50 mmol, 0.047 g), 3-nitrobenzaldehyde (0.50 mmol, 0.076 g) and cyclohexyl isocyanide (0.50 mmol, 0.062 mL), in 85% yield (0.144 g) as a yellow solid (m.p. 181-183 ºC) after silica gel column chromatography (20% ethyl acetate/hexane to 60% ethyl acetate/hexane). Rf = 0.37 (50% ethyl acetate/hexane).

FT-IR (ATR): ν 3228, 2924, 2852, 1524, 1444, 1348, 1309, 1271, 1230, 1103, 887, 806, 760, 746, 721, 710, 671, 658, 636 cm^{-1}.

^{1}H NMR (600 MHz, DMSO-d_6): δ 9.13 (t, *J* = 2.0 Hz, 1H), 8.67 (dt, *J* = 7.8, 1.2 Hz, 1H), 8.37 (dt, *J* = 6.8, 1.2 Hz, 1H), 8.15–8.10 (m, 1H), 7.74 (t, *J* = 8.0 Hz, 1H), 7.53 (dd, *J* = 9.0, 1.2 Hz, 1H), 7.24 (ddd, *J* = 9.0, 6.8, 1.2 Hz, 1H), 6.94 (td, *J* = 6.8, 1.2 Hz, 1H), 5.01 (d, *J* = 6.3 Hz, 1H), 2.91–2.82 (m, 1H), 1.80–1.75 (m, 2H), 1.69–1.62 (m, 2H), 1.53–1.49 (m, 1H), 1.37–1.28 (m, 2H), 1.21–1.07 (m, 3H) ppm.

^{13}C NMR (151 MHz, DMSO-d_6): δ 148.6, 141.3, 136.9, 132.7, 132.5, 130.4, 127.6, 125.2, 124.1, 121.7, 121.0, 117.5, 112.3, 57.3, 34.1, 25.8, 25.0 ppm.

HRMS (ESI-QTOF) *m/z* calculated for $C_{19}H_{21}N_4O_2^+$: 337.1659 [M+H]$^+$; found 337.1666.

4-(3-(Cyclohexylamino)imidazo[1,2-*a*]pyridin-2-yl)-2-methoxyphenol (**4v**) [15]

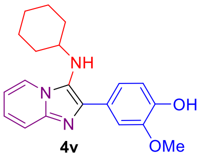

4v

4v was obtained from 2-aminopyridine (0.50 mmol, 0.047 g), vanillin (0.50 mmol, 0.076 g) and cyclohexyl isocyanide (0.50 mmol, 0.062 mL), in 84% yield (0.142 g) as a white solid m.p. 201-202 ºC) after silica gel column chromatography (50% ethyl acetate/hexane to 90% ethyl acetate/hexane). Rf = 0.29 (70% ethyl acetate/hexane).

FT-IR (ATR): ν 3361, 3305, 2929, 2850, 1631, 1604, 1568, 1502, 1444, 1410, 1342, 1281, 1255, 1225, 1178, 1124, 1030, 874, 814, 777, 754, 742, 661, 631, 604 cm^{-1}.

^1H NMR (600 MHz, DMSO-d_6): δ 9.04 (s, 1H), 8.29 (dt, *J* = 6.8, 1.2 Hz, 1H), 7.82 (d, *J* = 1.9 Hz, 1H), 7.67 (dd, *J* = 8.2, 1.9 Hz, 1H), 7.43 (dt, *J* = 9.0, 1.2 Hz, 1H), 7.14 (ddd, *J* = 9.0, 6.8, 1.2 Hz, 1H), 6.87–6.82 (m, 2H), 4.70 (d, *J* = 5.4 Hz, 1H), 2.91–2.84 (m, 1H), 1.74–1.68 (m, 2H), 1.67–1.61 (m, 2H), 1.53–1.49 (m, 1H), 1.32–1.23 (m, 2H), 1.14–1.07 (m, 3H) ppm.

^{13}C NMR (151 MHz, DMSO-d_6): δ 147.8, 146.2, 140.7, 135.8, 126.6, 124.9, 123.8, 123.6, 119.9, 116.8, 115.8, 111.5, 111.0, 56.8, 56.0, 34.1, 26.0, 24.9 ppm.

HRMS (ESI-QTOF) *m/z* calculated for $C_{20}H_{24}N_3O_2^+$: 338.1863 [M+H]$^+$; found 338.1870.

2-(Benzo[*d*][1,3]dioxol-5-yl)-*N*-cyclohexylimidazo[1,2-*a*]pyridin-3-amine (**4w**)

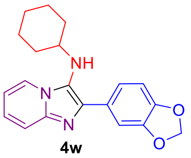

4w

4w was obtained from 2-aminopyridine (0.50 mmol, 0.047 g), piperonal (0.50 mmol, 0.076 g) and cyclohexyl isocyanide (0.50 mmol, 0.062 mL), in 88% yield (0.147 g) as a white solid (m.p. 163-165 ºC) after silica gel column chromatography (30% ethyl acetate/hexane to 70% ethyl acetate/hexane). Rf = 0.50 (50% ethyl acetate/hexane).

FT-IR (ATR): ν 3249, 2927, 2360, 1502, 1485, 1450, 1363, 1238, 1221, 1109, 1043, 943, 897, 816, 752, 737, 677, 634, 604 cm^{-1}.

^1H NMR (600 MHz, $CDCl_3$): δ 8.14 (dt, *J* = 6.8, 1.2 Hz, 1H), 7.62–7.58 (m, 2H), 7.56 (dd, *J* = 8.1, 1.7 Hz, 1H), 7.17 (ddd, *J* = 9.1, 6.8, 1.2 Hz, 1H), 6.88 (d, *J* = 8.1 Hz, 1H), 6.83 (td, *J* = 6.8, 1.2 Hz, 1H), 6.00 (s, 2H), 3.31 (s, 1H), 2.98–2.91 (m, 1H), 1.83–1.77 (m, 2H), 1.73–1.66 (m, 2H), 1.60–1.54 (m, 1H), 1.30–1.20 (m, 2H), 1.19–1.10 (m, 3H) ppm.

^{13}C NMR (151 MHz, $CDCl_3$): δ 147.9, 147.2, 140.5, 125.0, 124.3, 122.9, 121.1, 116.5, 112.3, 108.5, 107.7, 101.1, 56.7, 34.1, 25.7, 24.8 ppm.

HRMS (ESI-QTOF) *m/z* calculated for $C_{20}H_{22}N_3O_2^+$: 336.1707 [M+H]$^+$; found 336.1715.

N-Cyclohexyl-2-(2,4-dimethoxyphenyl)imidazo[1,2-*a*]pyridin-3-amine (**4x**)

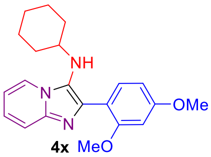

4x

4x was obtained from 2-aminopyridine (0.50 mmol, 0.047 g), 2,4-dimethoxybenzaldehyde (0.50 mmol, 0.083 g) and cyclohexyl isocyanide (0.50 mmol, 0.062 mL), in 99% yield (0.174 g) as an orange oil after silica gel column chromatography (30% ethyl acetate/hexane to 70% ethyl acetate/hexane). Rf = 0.27 (50% ethyl acetate/hexane).

FT-IR (ATR): ν 3334, 2926, 2846, 1616, 1562, 1506, 1452, 1313, 1281, 1205, 1113, 1032, 939, 822, 750, 729, 636 cm^{-1}.

^1H NMR (600 MHz, $CDCl_3$): δ 8.13 (dt, *J* = 6.8, 1.2 Hz, 1H), 7.79 (d, *J* = 8.5 Hz, 1H), 7.61 (dt, *J* = 9.0, 1.2 Hz, 1H), 7.14 (ddd, *J* = 9.1, 6.8, 1.2 Hz, 1H), 6.81 (td, *J* = 6.8, 1.2 Hz, 1H), 6.69 (dd, *J* = 8.5, 2.4 Hz, 1H), 6.60 (d, *J* = 2.4 Hz, 1H), 3.91 (s, 1H), 3.89 (s, 3H), 3.88 (s, 3H), 2.71–2.62 (m, 1H), 1.75–1.69 (m, 2H), 1.63–1.58 (m, 2H), 1.55–1.47 (m, 1H), 1.17–1.09 (m, 3H), 1.09–1.00 (m, 2H) ppm.

^{13}C NMR (151 MHz, $CDCl_3$): δ 160.7, 156.9, 141.3, 132.8, 132.3, 126.7, 123.5, 122.6, 116.9, 116.4, 111.5, 105.8, 99.3, 56.4, 56.1, 55.4, 34.1, 25.7, 24.8 ppm.

HRMS (ESI-QTOF) *m/z* calculated for $C_{21}H_{26}N_3O_2^+$: 352.2020 [M+H]$^+$; found 352.2022.

2-(3-(Cyclohexylamino)imidazo[1,2-*a*]pyridin-2-yl)-5-(diethylamino)phenol (**4y**)

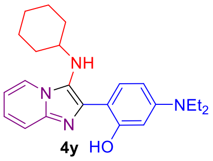

4y was obtained from 2-aminopyridine (0.50 mmol, 0.047 g), 4-(diethylamino)salicylaldehyde (0.50 mmol, 0.076 g) and cyclohexyl isocyanide (0.50 mmol, 0.062 mL), in 35% yield (0.066 g) as a yellow solid (m.p. 128-130 ºC) after silica gel column chromatography (5% ethyl acetate/hexane to 30% ethyl acetate/hexane). Rf = 0.28 (20% ethyl acetate/hexane).

FT-IR (ATR): ν 2970, 2927, 2854, 1624, 1562, 1535, 1448, 1404, 1358, 1300, 1273, 1221, 1147, 1078, 823, 789, 733, 660, 646, 631 cm^{-1}.

^1H NMR (600 MHz, $CDCl_3$): δ 8.19 (d, *J* = 6.8 Hz, 1H), 7.83 (d, *J* = 8.8 Hz, 1H), 7.47 (d, *J* = 8.8 Hz, 1H), 7.16 (ddd, *J* = 8.8, 6.8, 1.2 Hz, 1H), 6.83 (td, *J* = 6.8, 1.2 Hz, 1H), 6.34 (s, 1H), 6.31 (d, *J* = 8.8 Hz, 1H), 3.40 (q, *J* = 7.1 Hz, 4H), 3.07–3.04 (m, 1H), 1.87–1.81 (m, 2H), 1.77–1.70 (m, 2H), 1.64–1.60 (m, 1H), 1.38–1.29 (m, 2H), 1.23 (t, *J* = 7.1 Hz, 6H), 1.22–1.15 (m, 3H) ppm.

^{13}C NMR (151 MHz, $CDCl_3$): δ 158.9, 148.9, 139.1, 127.4, 122.4, 121.4, 115.5, 112.0, 103.3, 99.5, 56.9, 44.3, 33.9, 25.8, 24.9, 12.9 ppm.

HRMS (ESI-QTOF) *m/z* calculated for $C_{23}H_{31}N_4O^+$: 379.2492 [M+H]$^+$; found 379.2507.

4-Bromo-2-(3-(cyclohexylamino)imidazo[1,2-*a*]pyridin-2-yl)phenol (**4z**) [6]

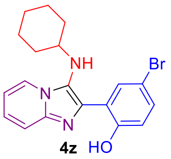

4z was obtained from 2-aminopyridine (0.50 mmol, 0.047 g), 5-bromosalicylaldehyde (0.50 mmol, 0.100 g) and cyclohexyl isocyanide (0.50 mmol, 0.062 mL), in 57% yield (0.109 g) as a yellowish white solid (m.p. 134-135 ºC) after silica gel column chromatography (5% ethyl acetate/hexane to 10% ethyl acetate/hexane). Rf = 0.50 (10% ethyl acetate/hexane).

FT-IR (ATR): ν 3292, 3354, 2980, 2929, 2852, 1566, 1473, 1437, 1363, 1346, 1281, 1248, 1080, 872, 812, 752, 739, 688, 650, 623 cm^{-1}.

^1H NMR (600 MHz, $CDCl_3$): δ 8.22 (s, 1H), 8.19 (d, *J* = 6.8 Hz, 1H), 7.54 (d, *J* = 9.0 Hz, 1H), 7.30–7.22 (m, 2H), 6.94 (td, *J* = 6.8, 1.1 Hz, 1H), 6.91 (d, *J* = 8.7 Hz, 1H), 3.29 (s, 1H), 3.03–2.96 (m, 1H), 1.89–1.82 (m, 2H), 1.79–1.70 (m, 2H), 1.66–1.59 (m, 1H), 1.39–1.30 (m, 2H), 1.30–1.15 (m, 3H) ppm.

^{13}C NMR (151 MHz, $CDCl_3$): δ 156.3, 139.3, 131.7, 129.2, 125.7, 123.9, 122.6, 119.3, 118.9, 116.3, 112.9, 110.6, 57.1, 34.1, 25.6, 24.8 ppm.

HRMS (ESI-QTOF) *m/z* calculated for $C_{19}H_{21}BrN_3O^+$: 386.0863 [M+H]$^+$; found 386.0851.

N-(*tert*-Butyl)-2-phenylimidazo[1,2-*a*]pyridin-3-amine (**4aa**) [6]

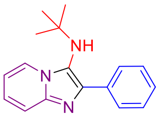
4aa

4aa was obtained from 2-aminopyridine (0.50 mmol, 0.047 g), benzaldehyde (0.50 mmol, 0.051 mL) and *tert*-butyl isocyanide (0.50 mmol, 0.057 mL), in 91% yield (0.120 g) as a white solid (m.p. 162-164 ºC) after silica gel column chromatography (20% ethyl acetate/hexane to 50% ethyl acetate/hexane). Rf = 0.50 (50% ethyl acetate/hexane).

FT-IR (ATR): ν 3315, 2968, 1649, 1604, 1543, 1491, 1444, 1385, 1363, 1336, 1213, 1149, 1066, 1030, 922, 750, 698, 642, 607 cm^{-1}.

^{1}H NMR (600 MHz, $CDCl_3$): δ 8.30–8.20 (m, 1H), 8.00–7.89 (m, 2H), 7.57 (dt, *J* = 8.9, 1.2 Hz, 1H), 7.48–7.40 (m, 2H), 7.39–7.30 (m, 1H), 7.20–7.10 (m, 1H), 6.84–6.76 (m, 1H), 3.14 (s, 1H), 1.07 (s, 9H) ppm.

^{13}C NMR (151 MHz, $CDCl_3$): δ 142.0, 139.6, 135.3, 128.2, 128.2, 127.4, 124.0, 123.5, 123.5, 117.4, 111.3, 56.4, 30.3 ppm.

HRMS (ESI-QTOF) *m/z* calculated for $C_{17}H_{20}N_3^+$: 266.1652 [M+H]$^+$; found 266.1649.

N-Cyclohexyl-2-phenylimidazo[1,2-*a*]pyridin-3-amine (**4bb**) [8]

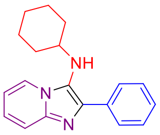
4bb

4bb was obtained from 2-aminopyridine (0.50 mmol, 0.047 g), benzaldehyde (0.50 mmol, 0.051 mL) and cyclohexyl isocyanide (0.50 mmol, 0.062 mL), in 99% yield (0.145 g) as a white solid (m.p. 174-175 ºC) after silica gel column chromatography (20% ethyl acetate/hexane to 50% ethyl acetate/hexane). Rf = 0.50 (50% ethyl acetate/hexane).

FT-IR (ATR): ν 3238, 2922, 2852, 1491, 1444, 1387, 1365, 1336, 1228, 1028, 769, 752, 737, 694, 648, 615 cm^{-1}.

^{1}H NMR (600 MHz, $CDCl_3$): δ 8.15 (dd, *J* = 6.8, 1.2 Hz, 1H), 8.08–8.04 (m, 2H), 7.61 (d, *J* = 9.0 Hz, 1H), 7.46 (t, *J* = 7.7 Hz, 2H), 7.33 (tt, *J* = 7.4, 1.2 Hz, 1H), 7.16 (ddd, *J* = 9.0, 6.8, 1.2 Hz, 1H), 6.82 (td, *J* = 6.8, 1.2 Hz, 1H), 3.26 (s, 1H), 3.02–2.95 (m, 1H), 1.86–1.80 (m, 2H), 1.73–1.67 (m, 2H), 1.61–1.55 (m, 1H), 1.31–1.22 (m, 2H), 1.22–1.12 (m, 3H) ppm.

^{13}C NMR (151 MHz, $CDCl_3$): δ 141.2, 135.8, 133.8, 128.6, 127.5, 127.1, 125.0, 124.5, 122.9, 117.1, 111.9, 56.9, 34.2, 25.7, 24.8 ppm.

HRMS (ESI-QTOF) *m/z* calculated for $C_{19}H_{22}N_3^+$: 292.1808 [M+H]$^+$; found 292.1815.

N-Cyclohexyl-2-(1*H*-indol-3-yl)imidazo[1,2-*a*]pyridin-3-amine (**4cc**) [16]

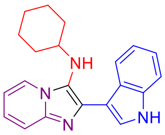
4cc

4cc was obtained from 2-aminopyridine (0.50 mmol, 0.047 g), indole-3-carboxaldehyde (0.50 mmol, 0.073 g) and cyclohexyl isocyanide (0.50 mmol, 0.062 mL), in 65% yield (0.108 g) as a white solid (m.p. 242-244 ºC) after silica gel column chromatography (80% ethyl acetate/hexane to 100% ethyl acetate/hexane). Rf = 0.43 (90% ethyl acetate/hexane).

FT-IR (ATR): ν 3147, 2924, 2850, 1668, 1620, 1595, 1493, 1448, 1329, 1269, 1236, 1203, 1126, 1009, 910, 735, 631, 600 cm^{-1}.

^{1}H NMR (600 MHz, DMSO-d_6): δ 11.24 (d, J = 2.6 Hz, 1H), 8.60 (dd, J = 8.0, 1.2 Hz, 1H), 8.28 (dt, J = 6.8, 1.2 Hz, 1H), 8.02 (d, J = 2.6 Hz, 1H), 7.49 (dt, J = 9.0, 1.2 Hz, 1H), 7.42 (dt, J = 8.0, 1.2 Hz, 1H), 7.17–7.06 (m, 3H), 6.86 (td, J = 6.8, 1.2 Hz, 1H), 4.66 (d, J = 5.7 Hz, 1H), 2.95–2.86 (m, 1H), 1.77–1.72 (m, 2H), 1.67–1.61 (m, 2H), 1.53–1.49 (m, 1H), 1.34–1.25 (m, 2H), 1.16–1.05 (m, 3H) ppm.

^{13}C NMR (151 MHz, DMSO-d_6): δ 141.0, 136.4, 134.8, 126.8, 124.1, 124.0, 123.1, 122.8, 122.6, 121.8, 119.4, 116.6, 111.7, 111.4, 109.8, 56.2, 34.2, 26.0, 25.0 ppm.

HRMS (ESI-QTOF) *m/z* calculated for $C_{21}H_{23}N_4^+$: 331.1917 [M+H]$^+$; found 331.1917.

N-Cyclohexyl-2-(furan-2-yl)imidazo[1,2-*a*]pyridin-3-amine (**4dd**) [6]

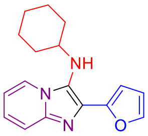
4dd

4dd was obtained from 2-aminopyridine (0.50 mmol, 0.047 g), furfural (0.50 mmol, 0.041 mL) and cyclohexyl isocyanide (0.50 mmol, 0.062 mL), in 98% yield (0.138 g) as a white solid (m.p. 96-97 ºC) after silica gel column chromatography (20% ethyl acetate/hexane to 50% ethyl acetate/hexane). Rf = 0.28 (30% ethyl acetate/hexane).

FT-IR (ATR): ν 3228, 2927, 2854, 1547, 1493, 1450, 1354, 1248, 1219, 1103, 1011, 931, 885, 800, 754, 741, 710, 650, 638, 627, 600 cm^{-1}.

^{1}H NMR (600 MHz, $CDCl_3$): δ 8.09 (dt, J = 6.8, 1.2 Hz, 1H), 7.58 (dd, J = 9.2, 1.2 Hz, 1H), 7.52–7.49 (m, 1H), 7.18 (ddd, J = 9.2, 6.8, 1.2 Hz, 1H), 6.97 (d, J = 3.3 Hz, 1H), 6.83 (td, J = 6.8, 1.2 Hz, 1H), 6.56–6.52 (m, 1H), 3.65 (s, 1H), 3.01–2.94 (m, 1H), 1.93–1.87 (m, 2H), 1.77–1.71 (m, 2H), 1.64–1.58 (m, 1H), 1.36–1.26 (m, 2H), 1.26–1.15 (m, 3H) ppm.

^{13}C NMR (151 MHz, $CDCl_3$): δ 149.4, 141.8, 141.2, 127.0, 125.5, 124.9, 122.9, 116.8, 112.2, 111.6, 107.3, 57.1, 34.1, 25.7, 25.0 ppm.

HRMS (ESI-QTOF) *m/z* calculated for $C_{17}H_{20}N_3O^+$: 282.1601 [M+H]$^+$; found 282.1595.

N-Cyclohexyl-2-(thiophen-2-yl)imidazo[1,2-*a*]pyridin-3-amine (**4ee**) [17]

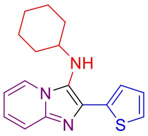
4ee

4ee was obtained from 2-aminopyridine (0.50 mmol, 0.047 g), thiophene-2-carbaldehyde (0.50 mmol, 0.047 mL) and cyclohexyl isocyanide (0.50 mmol, 0.062 mL), in 85% yield (0.126 g) as a white solid (m.p. 160-162 °C) after silica gel column chromatography (20% ethyl acetate/hexane to 40% ethyl acetate/hexane). Rf = 0.30 (30% ethyl acetate/hexane).

FT-IR (ATR): ν 3284, 3076, 2926, 2850, 1630, 1581, 1502, 1446, 1338, 1246, 1203, 1084, 852, 806, 754, 737, 688, 638, 625 cm^{-1}.

^{1}H NMR (600 MHz, $CDCl_3$): δ 8.11 (dt, J = 6.8, 1.2 Hz, 1H), 7.66 (dd, J = 3.6, 1.2 Hz, 1H), 7.56 (dt, J = 9.1, 1.2 Hz, 1H), 7.32 (dd, J = 5.0, 1.2 Hz, 1H), 7.18–7.10 (m, 2H), 6.80 (td, J = 6.8, 1.2 Hz, 1H), 3.13 (d, J = 4.7 Hz, 1H), 3.12–3.06 (m, 1H), 1.91–1.85 (m, 2H), 1.77–1.70 (m, 2H), 1.65–1.59 (m, 1H), 1.39–1.29 (m, 2H), 1.27–1.12 (m, 3H) ppm.

^{13}C NMR (151 MHz, $CDCl_3$): δ 141.4, 132.2, 127.7, 124.9, 124.8, 124.4, 124.0, 122.9, 116.8, 112.0, 57.1, 34.3, 25.7, 24.9 ppm.

HRMS (ESI-QTOF) *m/z* calculated for $C_{17}H_{20}N_3S^+$: 298.1372 $[M+H]^+$; found 298.1375.

N,2-Diphenylimidazo[1,2-*a*]pyridin-3-amine (**4ff**) [18]

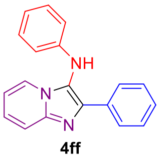

4ff was obtained from 2-aminopyridine (0.50 mmol, 0.047 g), benzaldehyde (0.50 mmol, 0.051 mL) and phenyl isocyanide (0.50 mmol, 0.053 mL), in 60% yield (0.086 g) as a white solid (m.p. 215-217 °C) after silica gel column chromatography (30% ethyl acetate/hexane to 50% ethyl acetate/hexane). Rf = 0.52 (50% ethyl acetate/hexane).

FT-IR (ATR): ν 3199, 3170, 3099, 3061, 2983, 2924, 1684, 1601, 1562, 1495, 1444, 1385, 1354, 1340, 1315, 1300, 1254, 1200, 1176, 1146, 1072, 1028, 841, 775, 754, 739, 706, 694, 611 cm^{-1}.

^{1}H NMR (600 MHz, DMSO-d_6): δ 8.28 (d, *J* = 1.3 Hz, 1H), 8.07 (dt, *J* = 7.9, 1.3 Hz, 2H), 7.97 (dt, *J* = 6.8, 1.2 Hz, 1H), 7.66 (dt, *J* = 9.0, 1.2 Hz, 1H), 7.43–7.37 (m, 2H), 7.34 (ddd, *J* = 9.0, 6.8, 1.2 Hz, 1H), 7.29 (tt, *J* = 7.3, 1.2 Hz, 1H), 7.14 (dd, *J* = 8.5, 7.3 Hz, 2H), 6.97–6.92 (m, 1H), 6.73 (tt, *J* = 7.3, 1.2 Hz, 1H), 6.53 (d, *J* = 7.9 Hz, 2H) ppm.

^{13}C NMR (151 MHz, DMSO-d_6): δ 146.0, 142.1, 137.5, 133.8, 130.0, 129.0, 128.1, 127.0, 126.0, 123.6, 119.5, 119.1, 117.4, 113.4, 113.0 ppm.

HRMS (ESI-QTOF) *m/z* calculated for $C_{19}H_{16}N_3^+$: 286.1339 $[M+H]^+$; found 286.1336.

2-(2-Bromophenyl)-*N*-phenylimidazo[1,2-*a*]pyridin-3-amine (**4gg**)

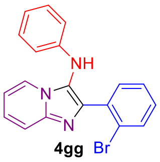

4gg was obtained from 2-aminopyridine (0.50 mmol, 0.047 g), 2-bromobenzaldehyde (0.50 mmol, 0.058 mL) and phenyl isocyanide (0.50 mmol, 0.053 mL), in 63% yield (0.116 g) as a white solid (m.p. 183-184 °C) after silica gel column chromatography (20% ethyl acetate/hexane to 50% ethyl acetate/hexane). Rf = 0.28 (30% ethyl acetate/hexane).

FT-IR (ATR): ν 3157, 3091, 3018, 2987, 2929, 1691, 1597, 1574, 1495, 1433, 1392, 1342, 1302, 1282, 1236, 1024, 922, 748, 694, 650, 609 cm^{-1}.

^{1}H NMR (600 MHz, $CDCl_3$): δ 7.86 (d, *J* = 6.7 Hz, 1H), 7.69 (d, *J* = 9.0 Hz, 1H), 7.60 (dd, *J* = 8.1, 1.1 Hz, 1H), 7.56 (dd, *J* = 7.7, 1.6 Hz, 1H), 7.33–7.26 (m, 2H), 7.19 (td, *J* = 7.7, 1.6 Hz, 1H), 7.16–7.09 (m, 2H), 6.83 (t, *J* = 6.7 Hz, 1H), 6.79 (tt, *J* = 7.4, 1.1 Hz, 1H), 6.49–6.44 (m, 2H), 5.96 (s, 1H) ppm.

^{13}C NMR (151 MHz, $CDCl_3$): δ 144.3, 142.0, 138.6, 134.4, 132.8, 132.5, 129.9, 129.5, 127.4, 125.3, 123.3, 123.2, 119.9, 119.8, 117.8, 113.7, 112.6 ppm.

HRMS (ESI-QTOF) *m/z* calculated for $C_{19}H_{15}BrN_3^+$: 364.0444 $[M+H]^+$; found 364.0430.

2-(2-Chlorophenyl)-*N*-phenylimidazo[1,2-*a*]pyridin-3-amine (**4hh**)

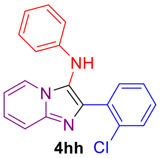

4hh

4hh was obtained from 2-aminopyridine (0.50 mmol, 0.047 g), 2-chlorobenzaldehyde (0.50 mmol, 0.056 mL) and phenyl isocyanide (0.50 mmol, 0.053 mL), in 61% yield (0.098 g) as a white solid (m.p. 174-175 ºC) after silica gel column chromatography (20% ethyl acetate/hexane to 50% ethyl acetate/hexane). Rf = 0.23 (30% ethyl acetate/hexane).

FT-IR (ATR): ν 3201, 3170, 3097, 3062, 2991, 2927, 1601, 1577, 1497, 1437, 1389, 1346, 1300, 1277, 1261, 1045, 920, 752, 739, 696, 600 cm^{-1}.

^{1}H NMR (600 MHz, DMSO-d_6): δ 8.04 (s, 1H), 7.92 (dt, *J* = 6.8, 1.2 Hz, 1H), 7.66 (dt, *J* = 9.1, 1.2 Hz, 1H), 7.55–7.48 (m, 2H), 7.41–7.33 (m, 3H), 7.11–7.05 (m, 2H), 6.98 (td, *J* = 6.8, 1.2 Hz, 1H), 6.67 (tt, *J* = 7.3, 1.2 Hz, 1H), 6.48–6.43 (m, 2H) ppm.

^{13}C NMR (151 MHz, DMSO-d_6): δ 145.9, 141.8, 137.1, 133.3, 133.2, 132.6, 130.2, 130.1, 129.7, 127.3, 125.7, 123.9, 121.0, 118.8, 117.6, 113.5, 113.0 ppm.

HRMS (ESI-QTOF) *m/z* calculated for $C_{19}H_{15}ClN_3^+$: 320.0949 $[M+H]^+$; found 320.0953.

2-(4-Nitrophenyl)-*N*-phenylimidazo[1,2-*a*]pyridin-3-amine (**4ii**)

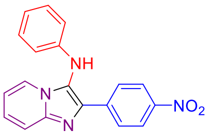

4ii

4ii was obtained from 2-aminopyridine (0.50 mmol, 0.047 g), 4-nitrobenzaldehyde (0.50 mmol, 0.076 g) and phenyl isocyanide (0.50 mmol, 0.053 mL), in 56% yield (0.093 g) as a yellow solid (m.p. 213-214 ºC) after silica gel column chromatography (30% ethyl acetate/hexane to 50% ethyl acetate/hexane). Rf = 0.23 (30% ethyl acetate/hexane).

FT-IR (ATR): ν 3371, 3207, 1599, 1570, 1516, 1497, 1385, 1340, 1302, 1246, 1198, 1147, 1111, 918, 860, 746, 735, 710, 692, 648, 638, 617, 604 cm^{-1}.

^{1}H NMR (600 MHz, DMSO-d_6): δ 8.46 (s, 1H), 8.33–8.26 (m, 4H), 8.02 (dd, *J* = 6.8, 1.2 Hz, 1H), 7.71 (dd, *J* = 9.1, 1.2 Hz, 1H), 7.42 (ddd, *J* = 9.1, 6.8, 1.2 Hz, 1H), 7.16 (dd, *J* = 8.5, 7.3 Hz, 2H), 7.01 (td, *J* = 6.8, 1.2 Hz, 1H), 6.76 (dd, *J* = 7.3, 1.2 Hz, 1H), 6.59–6.53 (m, 2H) ppm.

^{13}C NMR (151 MHz, DMSO-d_6): δ 146.8, 145.3, 142.4, 140.2, 135.0, 130.1, 127.6, 127.2, 124.4, 124.0, 121.7, 119.5, 117.6, 113.7 ppm.

HRMS (ESI-QTOF) *m/z* calculated for $C_{19}H_{15}N_4O_2^+$: 331.1190 $[M+H]^+$; found 331.1189.

Methyl (2-phenylimidazo[1,2-*a*]pyridin-3-yl)glycinate (**4jj**) [19]

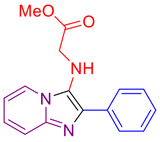

4jj

4jj was obtained from 2-aminopyridine (0.50 mmol, 0.047 g), benzaldehyde (0.50 mmol, 0.051 mL) and methyl isocyanoacetate (0.50 mmol, 0.046 mL), in 58% yield (0.082 g) as a yellow oil after silica gel column chromatography (30% ethyl acetate/hexane to 60% ethyl acetate/hexane). Rf = 0.32 (50% ethyl acetate/hexane).

FT-IR (ATR): ν 3228, 3357, 2953, 1741, 1631, 1564, 1502, 1444, 1390, 1356, 1200, 1074, 1026, 914, 752, 737, 698, 642 cm^{-1}.

^{1}H NMR (600 MHz, $CDCl_3$): δ 8.34–8.30 (m, 1H), 8.07–8.02 (m, 2H), 7.64–7.59 (m, 1H), 7.49–7.43 (m, 2H), 7.33 (tt, *J* = 7.4, 1.3 Hz, 1H), 7.24–7.17 (m, 1H), 6.90–6.84 (m, 1H), 3.98 (s, 1H), 3.84 (d, *J* = 5.6 Hz, 2H), 3.73 (s, 3H) ppm.

^{13}C NMR (151 MHz, $CDCl_3$): δ 172.2, 141.1, 128.7, 128.6, 127.8, 127.0, 125.1, 124.7, 123.1, 116.9, 112.3, 52.2, 49.1 ppm.

HRMS (ESI-QTOF) *m/z* calculated for $C_{16}H_{16}N_3O_2^+$: 282.1237 $[M+H]^+$; found 282.1237.

Methyl (2-(2-bromophenyl)imidazo[1,2-*a*]pyridin-3-yl)glycinate (**4kk**)

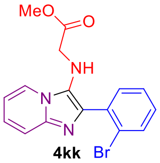

4kk

4kk was obtained from 2-aminopyridine (0.50 mmol, 0.047 g), 2-bromobenzaldehyde (0.50 mmol, 0.058 mL) and methyl isocyanoacetate (0.50 mmol, 0.046 mL), in 68% yield (0.122 g) as an orange oil after silica gel column chromatography (50% ethyl acetate/hexane to 80% ethyl acetate/hexane). Rf = 0.45 (70% ethyl acetate/hexane).

FT-IR (ATR): ν 3232, 2951, 2980, 1743, 1633, 1572, 1502, 1435, 1354, 1198, 1024, 752, 700, 650, 634 cm^{-1}.

^{1}H NMR (600 MHz, $CDCl_3$): δ 8.32 (d, *J* = 6.8 Hz, 1H), 7.70 (dd, *J* = 8.1, 1.2 Hz, 1H), 7.67 (d, *J* = 9.0 Hz, 1H), 7.61 (dd, *J* = 7.5, 1.7 Hz, 1H), 7.43 (td, *J* = 7.5, 1.2 Hz, 1H), 7.34–7.25 (m, 2H), 6.94 (td, *J* = 6.8, 1.2 Hz, 1H), 4.01 (t, *J* = 6.0 Hz, 1H), 3.65 (d, *J* = 6.0 Hz, 2H), 3.58 (s, 3H) ppm.

^{13}C NMR (151 MHz, $CDCl_3$): δ 171.4, 140.8, 132.8, 132.7, 130.1, 127.5, 125.9, 125.3, 123.4, 123.1, 117.1, 112.6, 52.0, 49.2 ppm.

HRMS (ESI-QTOF) *m/z* calculated for $C_{16}H_{15}BrN_3O_2^+$: 360.0342 $[M+H]^+$; found 360.0335.

Methyl (2-(4-nitrophenyl)imidazo[1,2-*a*]pyridin-3-yl)glycinate (**4ll**)

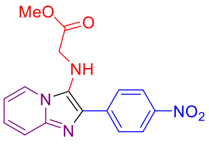

4ll

4ll was obtained from 2-aminopyridine (0.50 mmol, 0.047 g), 4-nitrobenzaldehyde (0.50 mmol, 0.076 g) and methyl isocyanoacetate (0.50 mmol, 0.046 mL), in 55% yield (0.090 g) as an orange solid (m.p. 128-130 ºC) after silica gel column chromatography (30% ethyl acetate/hexane to 70% ethyl acetate/hexane). Rf = 0.31 (50% ethyl acetate/hexane).

FT-IR (ATR): ν 3373, 1738, 1595, 1498, 1333, 1221, 1198, 1111, 1011, 972, 854, 750, 737, 715 cm^{-1}.

^{1}H NMR (600 MHz, $CDCl_3$): δ 8.38–8.27 (m, 5H) 7.60 (d, *J* = 9.1 Hz, 1H), 7.28–7.22 (m, 1H), 6.93–6.88 (m, 1H), 3.92–3.83 (m, 3H), 3.78 (s, 3H) ppm.

^{13}C NMR (151 MHz, $CDCl_3$): δ 172.2, 146.7, 127.2, 126.5, 125.6, 124.0, 122.9, 117.7, 112.7, 52.5, 49.1 ppm.

HRMS (ESI-QTOF) *m/z* calculated for $C_{16}H_{15}N_4O_4^+$: 327.1088 $[M+H]^+$; found 327.1089.

6-Chloro-*N*-cyclohexyl-2-phenylimidazo[1,2-*a*]pyridin-3-amine (**4mm**) [20]

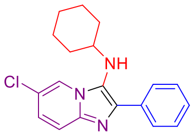
4mm

4mm was obtained from 2-amino-5-chloropyridine (0.50 mmol, 0.064 g), benzaldehyde (0.50 mmol, 0.051 mL) and cyclohexyl isocyanide (0.50 mmol, 0.062 mL), in 97% yield (0.157 g) as a white solid (m.p. 183-184 ºC) after silica gel column chromatography (5% ethyl acetate/hexane to 30% ethyl acetate/hexane). Rf = 0.59 (30% ethyl acetate/hexane).

FT-IR (ATR): ν 3255, 2927, 1603, 1498, 1448, 1412, 1321, 1267, 1217, 1186, 1099, 1070, 835, 804, 771, 696, 615 cm^{-1}.

^{1}H NMR (600 MHz, $CDCl_3$): δ 8.19 (d, *J* = 1.5 Hz, 1H), 8.02 (dd, *J* = 8.3, 1.3 Hz, 2H), 7.57 (d, *J* = 9.4 Hz, 1H), 7.46 (t, *J* = 7.5 Hz, 2H), 7.35 (t, *J* = 7.5 Hz, 1H), 7.13 (dd, *J* = 9.4, 2.0 Hz, 1H), 3.40 (s, 1H), 3.01–2.93 (m, 1H), 1.84–1.79 (m, 2H), 1.74–1.69 (m, 2H), 1.61–1.58 (m, 1H), 1.26 (d, *J* = 11.1 Hz, 2H), 1.22–1.15 (m, 3H) ppm.

^{13}C NMR (151 MHz, $CDCl_3$): δ 139.1, 136.6, 132.8, 128.7, 128.7, 128.0, 127.1, 126.3, 125.4, 120.9, 120.8, 117.2, 56.8, 34.1, 25.6, 24.8 ppm.

HRMS (ESI-QTOF) *m/z* calculated for $C_{19}H_{21}ClN_3^+$: 326.1419 $[M+H]^+$; found 326.1417.

2-(2-Bromophenyl)-6-chloro-*N*-cyclohexylimidazo[1,2-*a*]pyridin-3-amine (**4nn**)

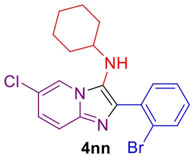
4nn

4nn was obtained from 2-amino-5-chloropyridine (0.50 mmol, 0.064 g), 2-bromobenzaldehyde (0.50 mmol, 0.058 mL) and cyclohexyl isocyanide (0.50 mmol, 0.062 mL), in 94% yield (0.157 g) as a grayish white solid (m.p. 110-111 ºC) after silica gel column chromatography (5% ethyl acetate/hexane to 30% ethyl acetate/hexane). Rf = 0.41 (20% ethyl acetate/hexane).

FT-IR (ATR): ν 3348, 2927, 2852, 1558, 1514, 1495, 1477, 1450, 1392, 1323, 1259, 1225, 1184, 1111, 1049, 1018, 937, 889, 831, 789, 735, 700, 658, 642, 600 cm^{-1}.

^{1}H NMR (600 MHz, $CDCl_3$): δ 8.23 (d, *J* = 2.0 Hz, 1H), 7.69 (dd, *J* = 8.1, 1.2 Hz, 1H), 7.65–7.58 (m, 2H), 7.44 (td, *J* = 7.5, 1.2 Hz, 1H), 7.33–7.27 (m, 1H), 7.19 (dd, *J* = 9.4, 2.0 Hz, 1H), 3.31 (d, *J* = 6.8 Hz, 1H), 2.72–2.65 (m, 1H), 1.71–1.64 (m, 2H), 1.63–1.55 (m, 2H), 1.53–1.46 (m, 1H), 1.15–1.07 (m, 3H), 1.07–0.98 (m, 2H) ppm.

^{13}C NMR (151 MHz, $CDCl_3$): δ 139.3, 136.9, 134.7, 132.8, 132.7, 130.0, 127.6, 126.4, 126.0, 122.9, 120.9, 120.8, 117.7, 56.4, 33.8, 25.6, 24.5 ppm.

HRMS (ESI-QTOF) *m/z* calculated for $C_{19}H_{20}BrClN_3^+$: 404.0524 $[M+H]^+$; found 404.0530.

N-Cyclohexyl-2-(4-(dimethylamino)phenyl)imidazo[1,2-*a*]pyridin-3-amine (**4oo**)

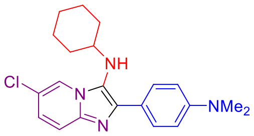
4oo

4oo was obtained from 2-amino-5-chloropyridine (0.50 mmol, 0.064 g), 4-(dimethylamino)benzaldehyde (0.50 mmol, 0.075 g) and cyclohexyl isocyanide (0.50 mmol, 0.062 mL), in 62% yield (0.117 g) as a white solid (m.p. 107-108 ºC) after silica gel column chromatography (10% ethyl acetate/hexane to 30% ethyl acetate/hexane). Rf = 0.36 (30% ethyl acetate/hexane).

FT-IR (ATR): ν 3456, 3292, 3238, 2980, 2927, 2852, 1616, 1554, 1522, 1483, 1444, 1392, 1323, 1228, 1169, 1111, 951, 804, 741, 690, 669, 644, 617 cm^{-1}.

^{1}H NMR (600 MHz, $CDCl_3$): δ 8.16 (dd, *J* = 2.1, 0.9 Hz, 1H), 7.95–7.89 (m, 2H), 7.52 (d, *J* = 9.4 Hz, 1H), 7.08 (dd, *J* = 9.4, 2.0 Hz, 1H), 6.81–6.75 (m, 2H), 3.36 (s, 1H), 3.03 (s, 6H), 3.02–2.95 (m, 1H), 1.85–1.79 (m, 2H), 1.75–1.68 (m, 2H), 1.63–1.57 (m, 1H), 1.34–1.25 (m, 2H), 1.25–1.12 (m, 3H) ppm.

^{13}C NMR (151 MHz, $CDCl_3$): δ 150.0, 146.5, 139.0, 137.5, 127.8, 125.4, 123.9, 120.6, 120.2, 116.7, 112.2, 56.7, 40.4, 34.1, 25.7, 24.8 ppm.

HRMS (ESI-QTOF) *m/z* calculated for $C_{21}H_{26}ClN_4^+$: 369.1841 $[M+H]^+$; found 369.1849.

6-Chloro-*N*-cyclohexyl-2-(4-nitrophenyl)imidazo[1,2-*a*]pyridin-3-amine (**4pp**)

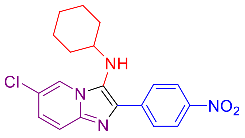

4pp

4pp was obtained from 2-amino-5-chloropyridine (0.50 mmol, 0.064 g), 4-nitrobenzaldehyde (0.50 mmol, 0.076 g) and cyclohexyl isocyanide (0.50 mmol, 0.062 mL), in 89% yield (0.165 g) as an orange solid (m.p. 107-108 ºC) after silica gel column chromatography (5% ethyl acetate/hexane to 60% ethyl acetate/hexane). Rf = 0.57 (30% ethyl acetate/hexane).

FT-IR (ATR): ν 3302, 3238, 2929, 2852, 1601, 1508, 1410, 1369, 1333, 1319, 1221, 1186, 1095, 860, 804, 719, 656, 634, 600 cm^{-1}.

^{1}H NMR (600 MHz, DMSO-*d*$_6$): δ 8.58 (dd, *J* = 2.0, 0.9 Hz, 1H), 8.48–8.45 (m, 2H), 8.35–8.30 (m, 2H), 7.58 (dd, *J* = 9.5, 0.9 Hz, 1H), 7.28 (dd, *J* = 9.5, 2.0 Hz, 1H), 5.10 (d, *J* = 7.2 Hz, 1H), 2.87–2.81 (m, 1H), 1.78–1.73 (m, 2H), 1.71–1.62 (m, 2H), 1.56–1.47 (m, 1H), 1.34–1.27 (m, 2H), 1.17–1.05 (m, 3H) ppm.

^{13}C NMR (151 MHz, DMSO-*d*$_6$): δ 141.5, 139.8, 133.1, 129.6, 127.3, 126.1, 124.3, 121.9, 119.8, 118.7, 57.6, 34.0, 25.8, 25.1 ppm.

HRMS (ESI-QTOF) *m/z* calculated for $C_{19}H_{20}ClN_4O_2^+$: 371.1269 $[M+H]^+$; found 371.1279.

2-(Benzo[*d*][1,3]dioxol-5-yl)-6-chloro-*N*-cyclohexylimidazo[1,2-*a*]pyridin-3-amine (**4qq**)

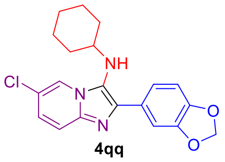

4qq

4qq was obtained from 2-amino-5-chloropyridine (0.50 mmol, 0.064 g), piperonal (0.50 mmol, 0.076 g) and cyclohexyl isocyanide (0.50 mmol, 0.062 mL), in 81% yield (0.150 g) as a yellowish white solid (m.p. 133-135 ºC) after silica gel column chromatography (5% ethyl acetate/hexane to 30% ethyl acetate/hexane). Rf = 0.50 (30% ethyl acetate/hexane).

FT-IR (ATR): ν 2926, 2885, 2848, 1624, 1554, 1502, 1483, 1448, 1363, 1240, 1217, 1188, 1120, 1065, 1043, 935, 916, 879, 812, 777, 737, 656, 648, 633 cm^{-1}.

^{1}H NMR (600 MHz, $CDCl_3$): δ 8.09 (d, *J* = 7.2 Hz, 1H), 7.60–7.58 (m, 1H), 7.56 (d, *J* = 1.7 Hz, 1H), 7.52 (dd, *J* = 8.1, 1.7 Hz, 1H), 6.89 (d, *J* = 8.1 Hz, 1H), 6.82 (dd, *J* = 7.2, 2.0 Hz, 1H), 6.03 (s, 2H), 3.39 (s, 1H), 2.98–2.90 (m, 1H), 1.83–1.77 (m, 2H), 1.74–1.67 (m, 2H), 1.62–1.56 (m, 1H), 1.30–1.11 (m, 5H) ppm.

^{13}C NMR (151 MHz, $CDCl_3$): δ 148.0, 147.4, 124.5, 123.4, 121.0, 115.2, 113.9, 108.5, 107.6, 101.2, 56.7, 34.1, 25.6, 24.8 ppm.

HRMS (ESI-QTOF) *m/z* calculated for $C_{20}H_{21}ClN_3O_2^+$: 370.1317 $[M+H]^+$; found 370.1319.

6-Chloro-*N*-cyclohexyl-2-(furan-2-yl)imidazo[1,2-*a*]pyridin-3-amine (**4rr**)

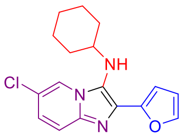

4rr

4rr was obtained from 2-amino-5-chloropyridine (0.50 mmol, 0.064 g), furfural (0.50 mmol, 0.041 mL) and cyclohexyl isocyanide (0.50 mmol, 0.062 mL), in 98% yield (0.154 g) as a white solid (m.p. 160-162 ºC) after silica gel column chromatography (10% ethyl acetate/hexane to 30% ethyl acetate/hexane). Rf = 0.32 (20% ethyl acetate/hexane).

FT-IR (ATR): ν 3286, 3236, 2927, 2854, 1493, 1450, 1414, 1358, 1319, 1250, 1215, 1163, 1113, 1007, 943, 885, 802, 731, 694, 642, 609 cm^{-1}.

^{1}H NMR (600 MHz, $CDCl_3$): δ 8.10 (d, *J* = 2.0 Hz, 1H), 7.53 (d, *J* = 1.8 Hz, 1H), 7.50 (d, *J* = 9.5 Hz, 1H), 7.12 (dd, *J* = 9.5, 2.0 Hz, 1H), 6.94 (d, *J* = 3.4 Hz, 1H), 6.55 (dd, *J* = 3.4, 1.8 Hz, 1H), 3.64 (d, *J* = 7.0 Hz, 1H), 3.02–2.94 (m, 1H), 1.93–1.87 (m, 2H), 1.79–1.73 (m, 2H), 1.67–1.60 (m, 1H), 1.35–1.15 (m, 5H) ppm.

^{13}C NMR (151 MHz, $CDCl_3$): δ 149.4, 141.9, 139.8, 128.7, 125.8, 125.8, 120.7, 120.5, 117.4, 111.7, 107.4, 57.1, 34.1, 25.7, 25.0 ppm.

HRMS (ESI-QTOF) *m/z* calculated for $C_{17}H_{19}ClN_3O^+$: 316.1211 $[M+H]^+$; found 316.1207.

7-Chloro-*N*-cyclohexyl-2-phenylimidazo[1,2-*a*]pyridin-3-amine (**4ss**)

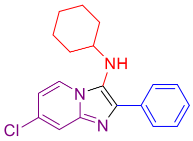

4ss

4ss was obtained from 2-amino-4-chloropyridine (0.50 mmol, 0.064 g), benzaldehyde (0.50 mmol, 0.051 mL) and cyclohexyl isocyanide (0.50 mmol, 0.062 mL), in 86% yield (0.140 g) as a white solid (m.p. 166-168 ºC) after silica gel column chromatography (5% ethyl acetate/hexane to 10% ethyl acetate/hexane). Rf = 0.59 (10% ethyl acetate/hexane).

FT-IR (ATR): ν 3280, 3055, 2927, 2852, 1626, 1560, 1487, 1444, 1387, 1367, 1342, 1225, 1194, 1066, 1030, 933, 883, 775, 715, 690, 669, 642, 629, 606 cm^{-1}.

^{1}H NMR (600 MHz, $CDCl_3$): δ 8.17 (t, *J* = 2.1 Hz, 1H), 8.01 (dd, *J* = 8.3, 1.4 Hz, 2H), 7.53 (dd, *J* = 9.5, 2.4 Hz, 1H), 7.46 (td, *J* = 8.0, 2.4 Hz, 2H), 7.35 (td, *J* = 7.2, 1.4 Hz, 1H), 7.11 (dt, *J* = 9.5, 2.4 Hz, 1H), 3.31 (s, 1H), 3.03–2.91 (m, 1H), 1.92–1.78 (m, 2H), 1.75–1.64 (m, 2H), 1.65–1.55 (m, 1H), 1.39–1.08 (m, 5H) ppm.

^{13}C NMR (151 MHz, $CDCl_3$): δ 139.4, 137.0, 133.3, 128.6, 128.6, 127.8, 127.1, 125.8, 125.4, 120.8, 120.5, 117.4, 117.4, 56.8, 34.1, 25.6, 25.6, 24.8 ppm.

HRMS (ESI-QTOF) *m/z* calculated for $C_{19}H_{21}ClN_3^+$: 326.1419 $[M+H]^+$; found 326.1433.

2-(2-Bromophenyl)-7-chloro-*N*-cyclohexylimidazo[1,2-*a*]pyridin-3-amine (**4tt**)

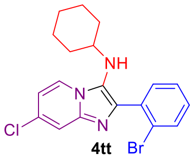
4tt

4tt was obtained from 2-amino-4-chloropyridine (0.50 mmol, 0.064 g), 2-bromobenzaldehyde (0.50 mmol, 0.058 mL) and cyclohexyl isocyanide (0.50 mmol, 0.062 mL), in 85% yield (0.171 g) as a dark-brown oil after silica gel column chromatography (5% ethyl acetate/hexane to 20% ethyl acetate/hexane). Rf = 0.24 (20% ethyl acetate/hexane).

FT-IR (ATR): ν 2929, 2852, 1626, 1562, 1498, 1475, 1448, 1394, 1346, 1230, 1188, 1093, 1059, 1024, 933, 908, 854, 779, 758, 733, 706, 642, 627, 606 cm^{-1}.

^{1}H NMR (600 MHz, $CDCl_3$): δ 8.12 (d, *J* = 7.3 Hz, 1H), 7.67 (dd, *J* = 8.1, 1.2 Hz, 1H), 7.61 (dd, *J* = 7.5, 1.7 Hz, 1H), 7.59 (d, *J* = 2.0 Hz, 1H), 7.42 (td, *J* = 7.5, 1.2 Hz, 1H), 7.28 (td, *J* = 7.5, 1.7 Hz, 1H), 6.84 (dd, *J* = 7.3, 2.0 Hz, 1H), 3.31 (d, *J* = 6.7 Hz, 1H), 2.70–2.63 (m, 1H), 1.69–1.63 (m, 2H), 1.63–1.55 (m, 2H), 1.53–1.43 (m, 1H), 1.14–0.97 (m, 5H) ppm.

^{13}C NMR (151 MHz, $CDCl_3$): δ 140.8, 136.9, 135.2, 132.7, 132.7, 130.8, 129.8, 127.6, 126.3, 123.4, 122.9, 116.1, 113.6, 56.5, 33.8, 25.6, 24.5 ppm.

HRMS (ESI-QTOF) *m/z* calculated for $C_{19}H_{20}BrClN_3^+$: 404.0524 $[M+H]^+$; found 404.0522.

7-Chloro-*N*-cyclohexyl-2-(4-(dimethylamino)phenyl)imidazo[1,2-*a*]pyridin-3-amine (**4uu**)

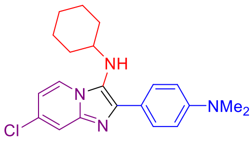
4uu

4uu was obtained from 2-amino-4-chloropyridine (0.50 mmol, 0.064 g), 4-(dimethylamino)benzaldehyde (0.50 mmol, 0.075 g) and cyclohexyl isocyanide (0.50 mmol, 0.062 mL), in 77% yield (0.142 g) as a white solid (m.p. 173-175 ºC) after silica gel column chromatography (10% ethyl acetate/hexane to 50% ethyl acetate/hexane). Rf = 0.40 (30% ethyl acetate/hexane).

FT-IR (ATR): ν 3290, 2980, 2929, 2854, 1618, 1522, 1504, 1446, 1400, 1346, 1227, 1198, 1059, 937, 852, 820, 779, 737, 656, 609 cm^{-1}.

^{1}H NMR (600 MHz, $CDCl_3$): δ 8.06 (dd, *J* = 7.2, 0.8 Hz, 1H), 7.94–7.89 (m, 2H), 7.56 (d, *J* = 2.0 Hz, 1H), 6.82–6.77 (m, 2H), 6.76 (dd, *J* = 7.2, 2.0 Hz, 1H), 3.33 (s, 1H), 3.03 (s, 6H), 3.02–2.93 (m, 1H), 1.85–1.79 (m, 2H), 1.74–1.67 (m, 2H), 1.63–1.57 (m, 1H), 1.32–1.23 (m, 2H), 1.23–1.12 (m, 3H) ppm.

^{13}C NMR (151 MHz, $CDCl_3$): δ 149.9, 127.8, 123.7, 123.1, 115.1, 113.2, 112.2, 56.8, 40.4, 34.1, 25.7, 24.8 ppm.

HRMS (ESI-QTOF) *m/z* calculated for $C_{21}H_{26}ClN_4^+$: 369.1841 $[M+H]^+$; found 369.1839.

8-Bromo-*N*-cyclohexyl-2-phenylimidazo[1,2-*a*]pyridin-3-amine (**4vv**)

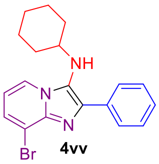
4vv

4vv was obtained from 2-amino-3-bromopyridine (0.50 mmol, 0.086 g), benzaldehyde (0.50 mmol, 0.051 mL) and cyclohexyl isocyanide (0.50 mmol, 0.062 mL), in 36% yield (0.067 g) as a white solid (m.p. 151-152 ºC) after silica gel column chromatography (5% ethyl acetate/hexane to 30% ethyl acetate/hexane). Rf = 0.50 (10% ethyl acetate/hexane).

FT-IR (ATR): ν 3303, 2922, 2854, 1552, 1495, 1446, 1392, 1348, 1234, 1188, 1074, 928, 769, 758, 735, 717, 700, 667, 640, 615 cm^{-1}.

^1H NMR (600 MHz, $CDCl_3$): δ 8.15 (dd, *J* = 6.8, 1.1 Hz, 1H), 8.05–8.00 (m, 2H), 7.44–7.39 (m, 3H), 7.29 (t, *J* = 7.4 Hz, 1H), 6.70 (t, *J* = 7.0 Hz, 1H), 3.44 (s, 1H), 2.96–2.88 (m, 1H), 1.81–1.75 (m, 2H), 1.70–1.62 (m, 2H), 1.58–1.52 (m, 1H), 1.26–1.09 (m, 5H) ppm.

^{13}C NMR (151 MHz, $CDCl_3$): δ 138.8, 137.1, 133.2, 128.4, 127.7, 127.6, 126.9, 126.6, 122.4, 111.9, 111.2, 56.9, 34.1, 25.7, 24.8 ppm.

HRMS (ESI-QTOF) *m/z* calculated for $C_{19}H_{21}BrN_3^+$: 370.0913 [M+H]$^+$; found 370.0906.

8-Bromo-2-(2-bromophenyl)-*N*-cyclohexylimidazo[1,2-*a*]pyridin-3-amine (**4ww**)

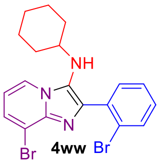

4ww

4ww was obtained from 2-amino-3-bromopyridine (0.50 mmol, 0.086 g), 2-bromobenzaldehyde (0.50 mmol, 0.058 mL) and cyclohexyl isocyanide (0.50 mmol, 0.062 mL), in 37% yield (0.083 g) as a dark-brown oil after silica gel column chromatography (5% ethyl acetate/hexane to 10% ethyl acetate/hexane). Rf = 0.32 (10% ethyl acetate/hexane).

FT-IR (ATR): ν 2980, 2929, 2852, 1684, 1560, 1510, 1448, 1346, 1263, 1207, 1024, 931, 762, 735, 648, 604 cm^{-1}.

^1H NMR (600 MHz, $CDCl_3$): δ 8.25 (d, *J* = 7.0 Hz, 1H), 7.68 (dd, *J* = 7.5, 1.7 Hz, 1H), 7.66 (dd, *J* = 8.1, 1.2 Hz, 1H), 7.50 (d, *J* = 7.0 Hz, 1H), 7.42 (td, *J* = 7.5, 1.2 Hz, 1H), 7.31–7.25 (m, 1H), 6.79 (t, *J* = 7.0 Hz, 1H), 3.46 (s, 1H), 2.72–2.65 (m, 1H), 1.70–1.64 (m, 2H), 1.62–1.53 (m, 2H), 1.52–1.44 (m, 1H), 1.12–0.99 (m, 5H) ppm.

^{13}C NMR (151 MHz, $CDCl_3$): δ 138.5, 134.5, 133.2, 132.5, 130.0, 127.6, 127.5, 127.2, 123.1, 122.6, 112.3, 111.0, 56.4, 33.8, 25.6, 24.5 ppm.

HRMS (ESI-QTOF) *m/z* calculated for $C_{19}H_{20}Br_2N_3^+$: 448.0018 [M+H]$^+$; found 448.0022.

2-(Benzo[*d*][1,3]dioxol-5-yl)-8-bromo-*N*-cyclohexylimidazo[1,2-*a*]pyridin-3-amine (**4xx**)

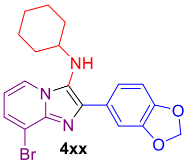

4xx

4xx was obtained from 2-amino-3-bromopyridine (0.50 mmol, 0.086 g), piperonal (0.50 mmol, 0.076 g) and cyclohexyl isocyanide (0.50 mmol, 0.062 mL), in 23% yield (0.048 g) as a white solid (m.p. 163-164 °C) after silica gel column chromatography (5% ethyl acetate/hexane to 20% ethyl acetate/hexane). Rf = 0.29 (10% ethyl acetate/hexane).

FT-IR (ATR): ν 3307, 2927, 2854, 1556, 1502, 1489, 1464, 1433, 1394, 1342, 1252, 1221, 1072, 1038, 931, 895, 866, 820, 762, 735, 660, 636, 625 cm^{-1}.

^1H NMR (600 MHz, $CDCl_3$): δ 8.09 (dd, *J* = 7.0, 1.1 Hz, 1H), 7.57 (d, *J* = 1.7 Hz, 1H), 7.53 (dd, *J* = 8.1, 1.7 Hz, 1H), 7.39 (dd, *J* = 7.0, 1.1 Hz, 1H), 6.86 (d, *J* = 8.1 Hz, 1H), 6.66 (t, *J* = 7.0 Hz, 1H), 6.00 (s, 2H), 3.23 (s, 1H), 2.95–2.88 (m, 1H), 1.80–1.74 (m, 2H), 1.70–1.65 (m, 2H), 1.59–1.53 (m, 1H), 1.26–1.09 (m, 5H) ppm.

^{13}C NMR (151 MHz, CDCl$_3$): δ 147.8, 147.2, 138.7, 137.2, 127.8, 126.4, 125.7, 122.2, 121.3, 111.7, 111.2, 108.3, 108.2, 101.1, 56.8, 34.1, 25.7, 24.8 ppm.

HRMS (ESI-QTOF) *m/z* calculated for $C_{20}H_{21}BrN_3O_2^+$: 414.0812 [M+H]$^+$; found 414.0821.

6-(2-bromophenyl)-*N*-cyclohexylimidazo[2,1-*b*]thiazol-5-amine (**4yy**)

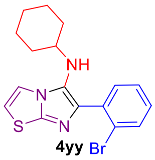

4yy was obtained from 2-aminothiazole (0.50 mmol, 0.050 g), 2-bromobenzaldehyde (0.50 mmol, 0.058 mL) and cyclohexyl isocyanide (0.50 mmol, 0.062 mL), in 44% yield (0.083 g) as an orange oil after silica gel column chromatography (20% ethyl acetate/hexane to 50% ethyl acetate/hexane). Rf = 0.52 (30% ethyl acetate/hexane).

FT-IR (ATR): ν 3109, 2927, 2852, 1672, 1543, 1477, 1375, 1240, 1180, 1024, 858, 760, 735, 660, 623, 600 cm^{-1}.

^{1}H NMR (600 MHz, CDCl$_3$): δ 7.66 (dd, *J* = 8.0, 1.2 Hz, 1H), 7.58 (dd, *J* = 7.6, 1.7 Hz, 1H), 7.45 (d, *J* = 4.4 Hz, 1H), 7.40 (td, *J* = 7.6, 1.2 Hz, 1H), 7.25 (td, *J* = 7.6, 1.7 Hz, 1H), 6.85 (d, *J* = 4.4 Hz, 1H), 3.31 (s, 1H), 2.75–2.67 (m, 1H), 1.74–1.67 (m, 2H), 1.64–1.57 (m, 2H), 1.53–1.47 (m, 1H), 1.17–1.07 (m, 3H), 1.07–0.99 (m, 2H) ppm.

^{13}C NMR (151 MHz, CDCl$_3$): δ 144.2, 132.8, 132.6, 129.5, 128.3, 127.5, 122.9, 117.3, 112.5, 57.1, 33.7, 25.6, 24.5 ppm.

HRMS (ESI-QTOF) *m/z* calculated for $C_{17}H_{19}BrN_3S^+$: 376.0478 [M+H]$^+$; found 376.0484.

2-(2-bromophenyl)-*N*-cyclohexylbenzo[*d*]imidazo[2,1-*b*]thiazol-3-amine (**4zz**)

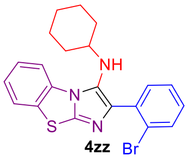

4zz was obtained from 2-aminobenzothiazole (0.50 mmol, 0.075 g), 2-bromobenzaldehyde (0.50 mmol, 0.058 mL) and cyclohexyl isocyanide (0.50 mmol, 0.062 mL), in 64% yield (0.137 g) as a dark-brown oil after silica gel column chromatography (5% ethyl acetate/hexane to 20% ethyl acetate/hexane). Rf = 0.54 (20% ethyl acetate/hexane).

FT-IR (ATR): ν 2927, 2852, 1693, 1572, 1489, 1373, 1259, 1205, 1018, 744, 650, 615 cm^{-1}.

^{1}H NMR (600 MHz, CDCl$_3$): δ 8.20 (dd, *J* = 8.1, 1.2 Hz, 1H), 7.73 (dd, *J* = 8.1, 1.2 Hz, 1H), 7.68 (dd, *J* = 8.1, 1.2 Hz, 1H), 7.63 (dd, *J* = 7.6, 1.2 Hz, 1H), 7.52–7.45 (m, 1H), 7.43 (td, *J* = 7.6, 1.2 Hz, 1H), 7.40–7.35 (m, 1H), 7.30–7.24 (m, 1H), 3.34 (s, 1H), 2.84–2.76 (m, 1H), 1.84–1.77 (m, 2H), 1.63–1.56 (m, 2H), 1.52–1.46 (m, 1H), 1.16–1.08 (m, 3H), 1.07–1.00 (m, 2H) ppm.

^{13}C NMR (151 MHz, CDCl$_3$): δ 143.0, 133.1, 132.8, 132.6, 130.7, 130.3, 129.5, 127.9, 127.6, 126.1, 124.7, 124.0, 122.8, 114.3, 57.1, 33.4, 25.6, 24.5 ppm.

HRMS (ESI-QTOF) *m/z* calculated for $C_{21}H_{21}BrN_3S^+$: 426.0634 [M+H]$^+$; found 426.0640.

2-(2-bromophenyl)-7-chloro-*N*-cyclohexylbenzo[*d*]imidazo[2,1-*b*]thiazol-3-amine (**4aaa**)

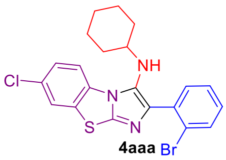

4aaa

4aaa was obtained from 2-amino-6-chlorobenzothiazole (0.50 mmol, 0.092 g), 2-bromobenzaldehyde (0.50 mmol, 0.058 mL) and cyclohexyl isocyanide (0.50 mmol, 0.062 mL), in 59% yield (0.136 g) as a yellow oil after silica gel column chromatography (5% ethyl acetate/hexane to 20% ethyl acetate/hexane). Rf = 0.32 (10% ethyl acetate/hexane).

FT-IR (ATR): ν 2929, 2852, 1693, 1593, 1564, 1489, 1371, 1309, 1265, 1205, 1097, 1028, 816, 764, 737, 644 cm^{-1}.

^{1}H NMR (600 MHz, $CDCl_3$): δ 8.14 (d, *J* = 8.6 Hz, 1H), 7.72 (d, *J* = 2.1 Hz, 1H), 7.69 (dd, *J* = 8.1, 1.2 Hz, 1H), 7.62 (dd, *J* = 7.6, 1.7 Hz, 1H), 7.46 (dd, *J* = 8.6, 2.1 Hz, 1H), 7.43 (td, *J* = 7.6, 1.2 Hz, 1H), 7.28 (td, *J* = 7.5, 1.7 Hz, 1H), 3.35 (s, 1H), 2.80–2.70 (m, 1H), 1.82–1.75 (m, 2H), 1.63–1.55 (m, 2H), 1.52–1.47 (m, 1H), 1.17–1.06 (m, 3H), 1.06–0.98 (m, 2H) ppm.

^{13}C NMR (151 MHz, $CDCl_3$): δ 142.7, 132.9, 132.6, 131.8, 131.6, 130.7, 130.4, 129.8, 127.7, 126.6, 123.7, 122.8, 115.1, 57.1, 33.4, 25.6, 24.5 ppm.

HRMS (ESI-QTOF) *m/z* calculated for $C_{21}H_{20}BrClN_3S^+$: 460.0244 $[M+H]^+$; found 460.0254.

N-(*tert*-Butyl)-2-isobutylimidazo[1,2-*a*]pyridin-3-amine (**5a**)

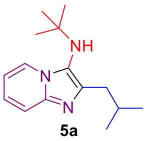

5a

5a was obtained from 2-aminopyridine (0.50 mmol, 0.047 g), isovaleraldehyde (0.50 mmol, 0.055 mL) and *tert*-butyl isocyanide (0.50 mmol, 0.057 mL), in 90% yield (0.110 g) as a white solid (m.p. 109-111 °C) after silica gel column chromatography (20% ethyl acetate/hexane to 50% ethyl acetate/hexane). Rf = 0.24 (50% ethyl acetate/hexane).

FT-IR (ATR): ν 3234, 2954, 2868, 1560, 1500, 1466, 1385, 1358, 1340, 1281, 1215, 796, 758, 741, 679, 609 cm^{-1}.

^{1}H NMR (600 MHz, $CDCl_3$): δ 8.20 (dt, *J* = 6.8, 1.2 Hz, 1H), 7.54 (d, *J* = 9.0 Hz, 1H), 7.14 (ddd, *J* = 9.0, 6.8, 1.3 Hz, 1H), 6.78 (td, *J* = 6.8, 1.2 Hz, 1H), 2.75 (s, 1H), 2.67 (d, *J* = 7.3 Hz, 2H), 2.29 (hept, *J* = 6.7 Hz, 1H), 1.22 (s, 9H), 0.96 (d, *J* = 6.7 Hz, 6H) ppm.

^{13}C NMR (151 MHz, $CDCl_3$): δ 141.4, 139.7, 124.1, 124.0, 123.4, 116.4, 111.2, 55.7, 36.8, 30.5, 28.7, 22.7 ppm.

HRMS (ESI-QTOF) *m/z* calculated for $C_{15}H_{24}N_3^+$: 246.1965 $[M+H]^+$; found 246.1970.

N-Cyclohexyl-2-isobutylimidazo[1,2-*a*]pyridin-3-amine (**5b**) [21]

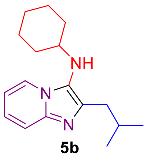

5b

5b was obtained from 2-aminopyridine (0.50 mmol, 0.047 g), isovaleraldehyde (0.50 mmol, 0.055 mL) and cyclohexyl isocyanide (0.50 mmol, 0.062 mL), in 87% yield (0.118 g) as a brown oil after silica gel column chromatography (20% ethyl acetate/hexane to 30% ethyl acetate/hexane). Rf = 0.23 (30% ethyl acetate/hexane).

FT-IR (ATR): ν 3226, 2926, 2850, 1630, 1568, 1498, 1464, 1350, 1271, 1209, 1178, 1109, 816, 750, 739, 665, 609 cm^{-1}.

^{1}H NMR (600 MHz, $CDCl_3$): δ 8.06 (dt, *J* = 6.7, 1.2 Hz, 1H), 7.51 (dt, *J* = 9.0, 1.2 Hz, 1H), 7.10 (ddd, *J* = 9.0, 6.7, 1.2 Hz, 1H), 6.76 (td, *J* = 6.7, 1.2 Hz, 1H), 2.93–2.86 (m, 1H), 2.84 (d, *J* = 5.8 Hz, 1H), 2.61 (d, *J* = 7.3 Hz, 2H), 2.24 (hept, *J* = 6.7 Hz, 1H), 1.93–1.82 (m, 2H), 1.81–1.67 (m, 2H), 1.70–1.56 (m, 1H), 1.34–1.12 (m, 6H), 0.97 (d, *J* = 6.7 Hz, 6H) ppm.

^{13}C NMR (151 MHz, $CDCl_3$): δ 141.0, 138.1, 125.3, 123.4, 122.5, 116.6, 116.6, 111.3, 57.2, 36.4, 34.3, 28.8, 25.8, 24.9, 22.7 ppm.

HRMS (ESI-QTOF) *m/z* calculated for $C_{17}H_{26}N_3^+$: 272.2121 [M+H]$^+$; found 272.2124.

2-Isobutyl-*N*-phenylimidazo[1,2-*a*]pyridin-3-amine (**5c**)

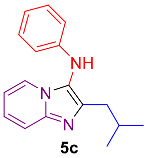

5c

5c was obtained from 2-aminopyridine (0.50 mmol, 0.047 g), isovaleraldehyde (0.50 mmol, 0.055 mL) and phenyl isocyanide (0.50 mmol, 0.053 mL), in 83% yield (0.110 g) as a white solid (m.p. 148-150 ºC) after silica gel column chromatography (20% ethyl acetate/hexane to 50% ethyl acetate/hexane). Rf = 0.24 (30% ethyl acetate/hexane).

FT-IR (ATR): ν 3201, 3170, 2956, 1684, 1601, 1579, 1497, 1462, 1392, 1342, 1300, 1211, 1169, 841, 754, 741, 696, 646 cm^{-1}.

^{1}H NMR (600 MHz, DMSO-*d*$_6$): δ 7.83 (s, 1H), 7.80 (dt, *J* = 6.7, 1.2 Hz, 1H), 7.50 (dt, *J* = 9.0, 1.2 Hz, 1H), 7.20 (ddd, *J* = 9.0, 6.7, 1.2 Hz, 1H), 7.14–7.08 (m, 2H), 6.84 (td, *J* = 6.7, 1.2 Hz, 1H), 6.69 (tt, *J* = 7.2, 1.2 Hz, 1H), 6.45–6.40 (m, 2H), 2.47 (d, *J* = 7.2 Hz, 2H), 2.08 (hept, *J* = 6.7 Hz, 1H), 0.86 (d, *J* = 6.7 Hz, 6H) ppm.

^{13}C NMR (151 MHz, DMSO-*d*$_6$): δ 146.8, 141.8, 141.0, 129.7, 124.1, 123.2, 120.1, 118.6, 117.1, 113.2, 111.9, 36.3, 28.3, 23.1 ppm.

HRMS (ESI-QTOF) *m/z* calculated for $C_{17}H_{20}N_3^+$: 266.1652 [M+H]$^+$; found 266.1655.

Methyl (2-isobutylimidazo[1,2-*a*]pyridin-3-yl)glycinate (**5d**)

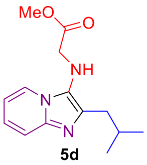

5d

5d was obtained from 2-aminopyridine (0.50 mmol, 0.047 g), isovaleraldehyde (0.50 mmol, 0.055 mL) and methyl isocyanoacetate (0.50 mmol, 0.046 mL), in 86% yield (0.112 g) as a yellow oil after silica gel column chromatography (50% ethyl acetate/hexane to 80% ethyl acetate/hexane). Rf = 0.27 (70% ethyl acetate/hexane).

FT-IR (ATR): ν 3234, 2954, 2868, 1745, 1633, 1577, 1504, 1437, 1350, 1277, 1209, 1007, 920, 754, 737, 633, 600 cm^{-1}.

^{1}H NMR (600 MHz, $CDCl_3$): δ 8.15 (dt, *J* = 6.7, 1.2 Hz, 1H), 7.51 (dt, *J* = 9.1, 1.2 Hz, 1H), 7.13 (ddd, *J* = 9.1, 6.7, 1.2 Hz, 1H), 6.80 (td, *J* = 6.7, 1.2 Hz, 1H), 3.79 (d, *J* = 5.8 Hz, 2H), 3.78 (s, 3H), 3.57 (t, *J* = 5.8 Hz, 1H), 2.64 (d, *J* = 7.3 Hz, 2H), 2.20 (hept, *J* = 6.7 Hz, 1H), 0.97 (d, *J* = 6.7 Hz, 6H) ppm.

^{13}C NMR (151 MHz, $CDCl_3$): δ 172.3, 141.1, 138.2, 125.4, 123.9, 122.5, 116.7, 111.6, 52.2, 50.0, 36.1, 28.8, 22.7 ppm.

HRMS (ESI-QTOF) *m/z* calculated for $C_{14}H_{20}N_3O_2^+$: 262.1550 [M+H]$^+$; found 262.1545.

6-Chloro-*N*-cyclohexyl-2-isobutylimidazo[1,2-*a*]pyridin-3-amine (**5e**)

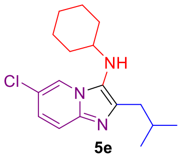

5e

5e was obtained from 2-amino-5-chloropyridine (0.50 mmol, 0.064 g), isovaleraldehyde (0.50 mmol, 0.055 mL) and cyclohexyl isocyanide (0.50 mmol, 0.062 mL), in 99% yield (0.153 g) as a white solid (m.p. 92-93 ºC) after silica gel column chromatography (5% ethyl acetate/hexane to 30% ethyl acetate/hexane). Rf = 0.43 (30% ethyl acetate/hexane).

FT-IR (ATR): ν 3244, 2929, 2852, 1574, 1497, 1448, 1408, 1367, 1315, 1273, 1201, 1051, 814, 800, 727, 683, 609 cm^{-1}.

^1H NMR (600 MHz, $CDCl_3$): δ 8.06 (d, *J* = 1.9 Hz, 1H), 7.39 (dd, *J* = 9.4, 0.8 Hz, 1H), 7.02 (dd, *J* = 9.4, 1.9 Hz, 1H), 2.90–2.85 (m, 1H), 2.83 (d, *J* = 5.7 Hz, 1H), 2.58 (d, *J* = 7.2 Hz, 2H), 2.19 (hept, *J* = 6.7 Hz, 1H), 1.88–1.82 (m, 2H), 1.78–1.71 (m, 2H), 1.65–1.59 (m, 1H), 1.29–1.13 (m, 5H), 0.95 (d, *J* = 6.7 Hz, 6H) ppm.

^{13}C NMR (151 MHz, $CDCl_3$): δ 139.9, 139.5, 125.8, 124.3, 120.4, 119.6, 117.1, 57.2, 36.5, 34.3, 28.8, 25.7, 24.9, 22.7 ppm.

HRMS (ESI-QTOF) *m/z* calculated for $C_{17}H_{25}ClN_3^+$: 306.1732 [M+H]$^+$; found 306.1736.

2-Benzyl-*N*-cyclohexylimidazo[1,2-*a*]pyridin-3-amine (**5f**) [22]

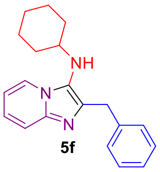

5f

5f was obtained from 2-aminopyridine (0.50 mmol, 0.047 g), phenylacetaldehyde (0.50 mmol, 0.060 g) and cyclohexyl isocyanide (0.50 mmol, 0.062 mL), in 63% yield (0.097 g) as a white solid (m.p. 124-125 ºC) after silica gel column chromatography (30% ethyl acetate/hexane to 50% ethyl acetate/hexane). Rf = 0.50 (50% ethyl acetate/hexane).

FT-IR (ATR): ν 3244, 2926, 2854, 1585, 1495, 1454, 1348, 1254, 1142, 1115, 841, 750, 735, 704, 692, 667, 617 cm^{-1}.

^1H NMR (600 MHz, $CDCl_3$): δ 8.06 (d, *J* = 6.8 Hz, 1H), 7.55–7.49 (m, 1H), 7.34–7.26 (m, 4H), 7.23–7.18 (m, 1H), 7.15–7.09 (m, 1H), 6.81–6.75 (m, 1H), 4.18 (d, *J* = 1.7 Hz, 2H), 2.79 (s, 1H), 2.66 (s, 1H), 1.82–1.66 (m, 4H), 1.62–1.58 (m, 1H), 1.22–1.12 (m, 5H) ppm.

^{13}C NMR (151 MHz, $CDCl_3$): δ 141.0, 139.6, 137.3, 128.7, 128.7, 128.5, 128.5, 126.2, 125.5, 123.6, 122.6, 116.8, 116.8, 111.5, 57.3, 34.2, 34.0, 34.0, 25.7, 24.9 ppm.

HRMS (ESI-QTOF) *m/z* calculated for $C_{20}H_{24}N_3^+$: 306.1965 [M+H]$^+$; found 306.1966.

N-Cyclohexyl-2-isopropylimidazo[1,2-*a*]pyridin-3-amine (**5g**) [7]

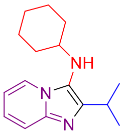

5g

5g was obtained from 2-aminopyridine (0.50 mmol, 0.047 g), isobutyraldehyde (0.50 mmol, 0.061 mL) and cyclohexyl isocyanide (0.50 mmol, 0.062 mL), in 74% yield (0.096 g) as a yellow oil after silica gel column chromatography (30% ethyl acetate/hexane to 50% ethyl acetate/hexane). Rf = 0.27 (50% ethyl acetate/hexane).

FT-IR (ATR): ν 3244, 2962, 2933, 2856, 1630, 1572, 1504, 1448, 1396, 1342, 1269, 1230, 1184, 1113, 1065, 953, 889, 806, 756, 739, 696, 621 cm^{-1}.

^{1}H NMR (600 MHz, $CDCl_3$): δ 8.05 (dt, *J* = 6.7, 1.2 Hz, 1H), 7.54 (dt, *J* = 9.0, 1.2 Hz, 1H), 7.09 (ddd, *J* = 9.0, 6.7, 1.2 Hz, 1H), 6.75 (td, *J* = 6.7, 1.2 Hz, 1H), 3.16 (hept, *J* = 6.9 Hz, 1H), 2.92–2.84 (m, 1H), 2.83 (s, 1H), 1.91–1.85 (m, 2H), 1.80–1.73 (m, 2H), 1.66–1.60 (m, 1H), 1.38 (d, *J* = 6.9 Hz, 6H), 1.30–1.15 (m, 5H) ppm.

^{13}C NMR (151 MHz, $CDCl_3$): δ 144.3, 141.2, 123.2, 123.1, 122.6, 116.8, 111.3, 57.2, 34.3, 26.1, 25.8, 24.9, 22.8 ppm.

HRMS (ESI-QTOF) *m/z* calculated for $C_{16}H_{24}N_3^+$: 258.1965 $[M+H]^+$; found 258.1967.

2-Isopropyl-*N*-phenylimidazo[1,2-*a*]pyridin-3-amine (**5h**)

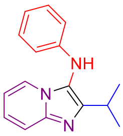

5h

5h was obtained from 2-aminopyridine (0.50 mmol, 0.047 g), isobutyraldehyde (0.50 mmol, 0.061 mL) and phenyl isocyanide (0.50 mmol, 0.053 mL), in 78% yield (0.098 g) as a white solid (m.p. 204-206 ºC) after silica gel column chromatography (20% ethyl acetate/hexane to 70% ethyl acetate/hexane). Rf = 0.43 (30% ethyl acetate/hexane).

FT-IR (ATR): ν 3197, 3159, 3082, 2980, 2926, 1599, 1576, 1498, 1396, 1346, 1327, 1257, 1159, 1066, 947, 754, 739, 696, 633, 604 cm^{-1}.

^{1}H NMR (600 MHz, DMSO-d_6): δ 7.83 (dt, *J* = 6.7, 1.2 Hz, 1H), 7.80 (s, 1H), 7.51 (dt, *J* = 9.0, 1.2 Hz, 1H), 7.20 (ddd, *J* = 9.1, 6.7, 1.3 Hz, 1H), 7.15–7.09 (m, 2H), 6.83 (td, *J* = 6.7, 1.2 Hz, 1H), 6.69 (tt, *J* = 7.3, 1.2 Hz, 1H), 6.45–6.39 (m, 2H), 3.03 (hept, *J* = 6.9 Hz, 1H), 1.23 (d, *J* = 6.9 Hz, 6H) ppm.

^{13}C NMR (151 MHz, DMSO-d_6): δ 147.1, 146.9, 141.8, 129.8, 124.1, 123.1, 118.5, 118.1, 117.2, 113.1, 112.0, 26.6, 22.8 ppm.

HRMS (ESI-QTOF) *m/z* calculated for $C_{16}H_{18}N_3^+$: 252.1495 $[M+H]^+$; found 252.1492.

Methyl (2-isopropylimidazo[1,2-*a*]pyridin-3-yl)glycinate (**5i**)

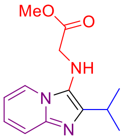

5i

5i was obtained from 2-aminopyridine (0.50 mmol, 0.047 g), isobutyraldehyde (0.50 mmol, 0.061 mL) and methyl isocyanoacetate (0.50 mmol, 0.046 mL), in 83% yield (0.102 g) as a yellow oil after silica gel column chromatography (50% ethyl acetate/hexane to 70% ethyl acetate/hexane). Rf = 0.43 (70% ethyl acetate/hexane).

FT-IR (ATR): ν 3359, 3232, 2964, 2873, 1743, 1633, 1574, 1504, 1439, 1396, 1350, 1275, 1207, 1068, 756, 739, 634, 604 cm^{-1}.

^{1}H NMR (600 MHz, $CDCl_3$): δ 8.18 (dt, J = 6.8, 1.2 Hz, 1H), 7.60 (dt, J = 9.0, 1.2 Hz, 1H), 7.17 (ddd, J = 9.0, 6.8, 1.2 Hz, 1H), 6.83 (td, J = 6.8, 1.2 Hz, 1H), 3.80 (d, J = 5.8 Hz, 2H), 3.79 (s, 3H), 3.56 (t, J = 5.8 Hz, 1H), 3.25 (hept, J = 6.9 Hz, 1H), 1.39 (d, J = 6.9 Hz, 6H) ppm.

^{13}C NMR (151 MHz, $CDCl_3$): δ 172.3, 143.9, 141.0, 124.2, 123.4, 122.6, 116.7, 111.9, 52.2, 50.1, 26.1 ppm.

HRMS (ESI-QTOF) *m/z* calculated for $C_{13}H_{18}N_3O_2^+$: 248.1394 [M+H]$^+$; found 248.1399.

N,2-Dicyclohexylimidazo[1,2-*a*]pyridin-3-amine (**5j**) [23]

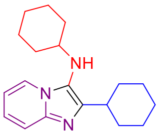

5j

5j was obtained from 2-aminopyridine (0.50 mmol, 0.047 g), cyclohexanecarbaldehyde (0.50 mmol, 0.061 mL) and cyclohexyl isocyanide (0.50 mmol, 0.062 mL), in 99% yield (0.147 g) as a white solid (m.p. 149-150 ºC) after silica gel column chromatography (30% ethyl acetate/hexane to 60% ethyl acetate/hexane). Rf = 0.50 (50% ethyl acetate/hexane).

FT-IR (ATR): ν 3244, 2933, 2848, 1574, 1498, 1448, 1402, 1344, 1306, 1269, 1217, 1178, 1117, 889, 804, 754, 741, 661, 617 cm^{-1}.

^{1}H NMR (600 MHz, $CDCl_3$): δ 8.05 (dt, J = 6.7, 1.2 Hz, 1H), 7.55 (d, J = 9.0 Hz, 1H), 7.10 (ddd, J = 9.0, 6.8, 1.2 Hz, 1H), 6.76 (td, J = 6.8, 1.2 Hz, 1H), 2.88–2.84 (m, 2H), 2.79–2.72 (m, 1H), 1.92–1.72 (m, 11H), 1.66–1.60 (m, 1H), 1.44–1.36 (m, 3H), 1.29–1.19 (m, 5H) ppm.

^{13}C NMR (151 MHz, $CDCl_3$): δ 143.5, 141.1, 123.4, 123.3, 122.6, 116.8, 111.3, 57.2, 36.3, 34.2, 32.9, 26.9, 25.9, 25.8, 24.9 ppm.

HRMS (ESI-QTOF) *m/z* calculated for $C_{19}H_{28}N_3^+$: 298.2278 [M+H]$^+$; found 298.2277.

2-Cyclohexyl-*N*-phenylimidazo[1,2-*a*]pyridin-3-amine (**5k**) [23]

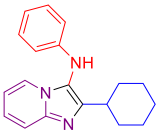

5k

5k was obtained from 2-aminopyridine (0.50 mmol, 0.047 g), cyclohexanecarbaldehyde (0.50 mmol, 0.061 mL) and phenyl isocyanide (0.50 mmol, 0.053 mL), in 88% yield (0.128 g) as a white solid (m.p. 171-173 ºC) after silica gel column chromatography (30% ethyl acetate/hexane to 60% ethyl acetate/hexane). Rf = 0.46 (50% ethyl acetate/hexane).

FT-IR (ATR): ν 3201, 3172, 3093, 2989, 2927, 2852, 1684, 1601, 1570, 1495, 1444, 1398, 1342, 1308, 1259, 752, 741, 696 cm^{-1}.

^{1}H NMR (600 MHz, DMSO-d_6): δ 7.81 (s, 1H), 7.79 (dt, J = 6.7, 1.2 Hz, 1H), 7.50 (dt, J = 9.0, 1.2 Hz, 1H), 7.19 (ddd, J = 9.0, 6.7, 1.2 Hz, 1H), 7.15–7.09 (m, 2H), 6.82 (td, J = 6.7, 1.2 Hz, 1H), 6.69 (tt, J = 7.4, 1.2 Hz, 1H), 6.44–6.39 (m, 2H), 2.70 (tt, J = 11.8, 3.4 Hz, 1H), 1.80–1.72 (m, 4H), 1.69–1.59 (m, 3H), 1.33–1.16 (m, 3H) ppm.

^{13}C NMR (151 MHz, DMSO-d_6): δ 147.0, 146.3, 141.8, 129.8, 124.1, 123.1, 118.5, 118.2, 117.2, 113.1, 111.9, 36.4, 32.6, 26.6, 26.2 ppm.

HRMS (ESI-QTOF) *m/z* calculated for $C_{19}H_{22}N_3^+$: 292.1808 [M+H]$^+$; found 292.1812.

Methyl (2-cyclohexylimidazo[1,2-*a*]pyridin-3-yl)glycinate (**5l**)

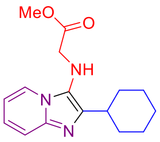

5l

5l was obtained from 2-aminopyridine (0.50 mmol, 0.047 g), cyclohexanecarbaldehyde (0.50 mmol, 0.061 mL) and methyl isocyanoacetate (0.50 mmol, 0.046 mL), in 86% yield (0.123 g) as a yellow oil after silica gel column chromatography (30% ethyl acetate/hexane to 70% ethyl acetate/hexane). Rf = 0.27 (50% ethyl acetate/hexane).

FT-IR (ATR): ν 2926, 2850, 1745, 1633, 1574, 1504, 1446, 1346, 1273, 1205, 1174, 1128, 1005, 754, 737, 638, 600 cm^{-1}.

^{1}H NMR (600 MHz, $CDCl_3$): δ 8.19 (d, *J* = 6.8 Hz, 1H), 7.62 (d, *J* = 9.0 Hz, 1H), 7.21–7.15 (m, 1H), 6.84 (t, *J* = 6.8 Hz, 1H), 3.82–3.79 (m, 5H), 3.56 (t, *J* = 5.9 Hz, 1H), 2.92–2.82 (m, 1H), 1.92–1.86 (m, 2H), 1.86–1.80 (m, 4H), 1.79–1.73 (m, 1H), 1.48–1.35 (m, 3H) ppm.

^{13}C NMR (151 MHz, $CDCl_3$): δ 172.2, 140.9, 124.4, 123.6, 122.6, 117.1, 116.7, 112.0, 52.3, 50.5, 50.3, 36.2, 32.8, 26.7, 26.5, 25.9 ppm.

HRMS (ESI-QTOF) *m/z* calculated for $C_{16}H_{22}N_3O_2^+$: 288.1707 [M+H]$^+$; found 288.1705.

N-Cyclohexyl-2-hexylimidazo[1,2-*a*]pyridin-3-amine (**5m**) [21]

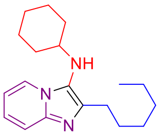

5m

5m was obtained from 2-aminopyridine (0.50 mmol, 0.047 g), heptaldehyde (0.50 mmol, 0.071 mL) and cyclohexyl isocyanide (0.50 mmol, 0.062 mL), in 83% yield (0.124 g) as a brown oil after silica gel column chromatography (30% ethyl acetate/hexane to 60% ethyl acetate/hexane). Rf = 0.33 (50% ethyl acetate/hexane).

FT-IR (ATR): ν 3226, 2924, 2850, 1631, 1574, 1502, 1448, 1398, 1344, 1273, 1093, 889, 808, 754, 739, 665, 633 cm^{-1}.

^{1}H NMR (600 MHz, $CDCl_3$): δ 8.10 (dd, *J* = 6.8, 1.2 Hz, 1H), 7.60 (d, *J* = 8.8 Hz, 1H), 7.18 (ddd, *J* = 8.8, 6.8, 1.2 Hz, 1H), 6.83 (td, *J* = 6.8, 1.2 Hz, 1H), 2.94–2.83 (m, 2H), 2.76 (t, *J* = 7.8 Hz, 2H), 1.93–1.85 (m, 2H), 1.85–1.74 (m, 4H), 1.67–1.61 (m, 1H), 1.45–1.37 (m, 2H), 1.39–1.29 (m, 4H), 1.31–1.17 (m, 5H), 0.95–0.85 (m, 3H) ppm.

^{13}C NMR (151 MHz, $CDCl_3$): δ 140.6, 124.6, 124.3, 122.6, 116.3, 111.9, 57.3, 34.3, 31.7, 29.5, 29.5, 26.9, 25.8, 24.9, 22.6, 14.1 ppm.

HRMS (ESI-QTOF) *m/z* calculated for $C_{19}H_{30}N_3^+$: 300.2434 [M+H]$^+$; found 300.2436.

2-Hexyl-*N*-phenylimidazo[1,2-*a*]pyridin-3-amine (**5n**)

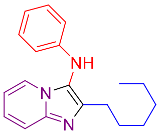

5n

5n was obtained from 2-aminopyridine (0.50 mmol, 0.047 g), heptaldehyde (0.50 mmol, 0.071 mL) and phenyl isocyanide (0.50 mmol, 0.053 mL), in 63% yield (0.093 g) as a white solid (m.p. 148-149 ºC) after silica gel column chromatography (30% ethyl acetate/hexane to 60% ethyl acetate/hexane). Rf = 0.42 (50% ethyl acetate/hexane).

FT-IR (ATR): ν 3159, 3084, 2985, 2852, 2926, 1684, 1601, 1579, 1495, 1402, 1348, 1308, 1254, 750, 694, 650, 600 cm^{-1}.

^{1}H NMR (600 MHz, DMSO-*d*$_6$): δ 7.85–7.80 (m, 2H), 7.49 (dt, *J* = 9.0, 1.2 Hz, 1H), 7.20 (ddd, *J* = 9.0, 6.7, 1.2 Hz, 1H), 7.14–7.08 (m, 2H), 6.84 (td, *J* = 6.7, 1.2 Hz, 1H), 6.69 (tt, *J* = 7.3, 1.2 Hz, 1H), 6.42 (dd, *J* = 7.8, 1.2 Hz, 2H), 2.58 (t, *J* = 7.5 Hz, 2H), 1.64 (quint, *J* = 7.5 Hz, 2H), 1.28–1.22 (m, 2H), 1.22–1.17 (m, 4H), 0.83–0.77 (m, 3H) ppm.

^{13}C NMR (151 MHz, DMSO-*d*$_6$): δ 146.9, 141.8, 141.7, 129.7, 124.1, 123.1, 119.6, 118.6, 117.1, 113.2, 111.9, 31.5, 29.0, 29.0, 27.0, 22.5, 14.4 ppm.

HRMS (ESI-QTOF) *m/z* calculated for $C_{19}H_{24}N_3^+$: 294.1965 [M+H]$^+$; found 294.1964.

Methyl (2-hexylimidazo[1,2-*a*]pyridin-3-yl)glycinate (**5o**)

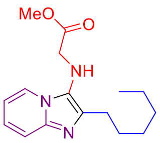

5o

5o was obtained from 2-aminopyridine (0.50 mmol, 0.047 g), heptaldehyde (0.50 mmol, 0.071 mL) and methyl isocyanoacetate (0.50 mmol, 0.046 mL), in 77% yield (0.112 g) as a yellow oil after silica gel column chromatography (70% ethyl acetate/hexane to 90% ethyl acetate/hexane). Rf = 0.30 (70% ethyl acetate/hexane).

FT-IR (ATR): ν 3215, 2927, 1745, 1633, 1577, 1502, 1439, 1348, 1205, 1005, 754, 739, 600 cm^{-1}.

^{1}H NMR (600 MHz, $CDCl_3$): δ 8.14 (dt, *J* = 6.8, 1.2 Hz, 1H), 7.52–7.47 (m, 1H), 7.15–7.09 (m, 1H), 6.79 (tt, *J* = 6.8, 1.2 Hz, 1H), 3.79 (dd, *J* = 5.8, 1.2 Hz, 2H), 3.77 (d, *J* = 1.8 Hz, 3H), 3.56 (t, *J* = 5.7 Hz, 1H), 2.75 (td, *J* = 7.7, 1.5 Hz, 2H), 1.79–1.71 (m, 2H), 1.43–1.36 (m, 2H), 1.36–1.26 (m, 4H) 0.91–0.85 (m, 3H) ppm.

^{13}C NMR (151 MHz, $CDCl_3$): δ 172.3, 141.2, 139.2, 124.7, 123.7, 122.5, 116.7, 111.6, 52.2, 50.2, 50.0, 31.7, 29.7, 29.4, 27.1, 22.6, 14.1 ppm.

HRMS (ESI-QTOF) *m/z* calculated for $C_{16}H_{24}N_3O_2^+$: 290.1863 [M+H]$^+$; found 290.1871.

2. Calculations of green chemistry metrics

Comparison of the present protocol for the synthesis imidazo[1,2-*a*]pyridines with already reported methods:

1. Calculation for the synthesis of *N*-(*tert*-butyl)-2-phenylimidazo[1,2-*a*]pyridin-3-amine in EtOH with CALB [24]

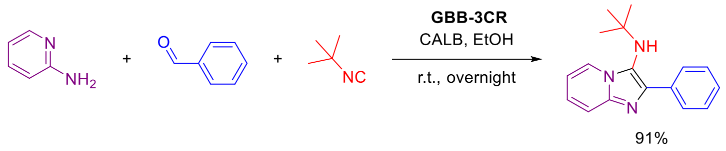

Raw material	Mass, mg
2-aminopyridine	50.00
Benzaldehyde	67.61
tert-butyl isocyanide	72.50
CALB	40.00
EtOH (2 mL)	1,578.00
**Total**	1,808.11

Product and waste	Mass, mg
Product	128.22
Recovered CALB	40.00
Waste	1,639.89
**Total**	1,808.11

$$\mathbf{E-factor\ (E)} = \frac{\text{1,639.89 mg of waste produced}}{\text{128.22 mg of product}} = 12.79$$

$$\mathbf{Mass\ intensity} = \frac{\text{1,808.11 mg of raw material used}}{\text{168.22 mg of CALB + product}} = 10.75$$

Observations:

- CALB catalyst was recovered and reused, it was not considered as waste in calculations.
- Calculation did not consider the ethyl acetate (2 × 5 mL) used for washing during filtration through Celite.
- Calculation did not consider the solvent system ethyl acetate/*n*-hexane used for column chromatography.

2. Calculation for the synthesis of *N*-cyclohexyl-2-(4-nitrophenyl)imidazo[1,2-*a*]pyridin-3-amine in ethylene glycol with AgOAc [3]

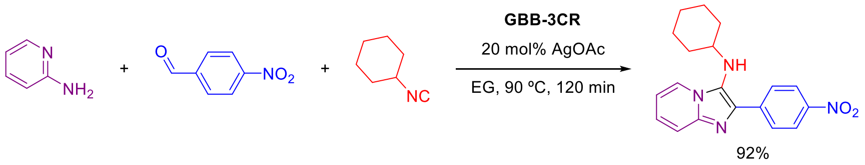

Raw material	Mass, mg
2-aminopyridine	94.12
4-nitrobenzaldehyde	151.12
Cyclohexyl isocyanide	109.17
AgOAc	33.38
EG (3 mL)	3,330.00
**Total**	3,717.79

Product and waste	Mass, mg
Product	309.48
Waste	3,408.31
**Total**	3,717.79

$$\mathbf{E-factor\ (E)} = \frac{3{,}408.31\ \text{mg of waste produced}}{309.48\ \text{mg of product}} = 11.01$$

$$\mathbf{Mass\ intensity} = \frac{3{,}717.79\ \text{mg of raw material used}}{309.48\ \text{mg of product}} = 12.01$$

Observation:

- Calculation did not consider the solvent system ethyl acetate/*n*-hexane (4:6) used for column chromatography.

3. **3.** Calculation for the synthesis of *N*-(*tert*-butyl)-2-(4-methoxyphenyl)imidazo[1,2-*a*]pyridin-3-amine in MeOH with $Gd(OTf)_3$ [19]

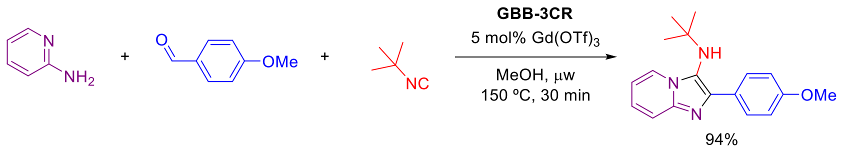

Raw material	Mass, mg	Product and waste	Mass, mg
2-aminopyridine	47.06	Product	138.83
4-methoxybenzaldehyde	68.07	Waste	1220.98
tert-butyl isocyanide	41.57	**Total**	1,359.81
$Gd(OTf)_3$	15.11		
MeOH (1.5 mL)	1,188.00		
**Total**	1,359.81		

$$\mathbf{E-factor\ (E)} = \frac{1{,}220.98\ \text{mg of waste produced}}{138.83\ \text{mg of product}} = 8.79$$

$$\mathbf{Mass\ intensity} = \frac{1{,}359.81\ \text{mg of raw material used}}{138.83\ \text{mg of product}} = 9.79$$

Observation:

- Calculation did not consider the solvent system ethyl acetate/hexane (gradient 0 to 50%) used for column chromatography.

4. **4.** Calculation for the synthesis of *N*-cyclohexyl-2-phenylimidazo[1,2-*a*]pyridin-3-amine in 1,4-dioxane with $ZnCl_2$ [13]

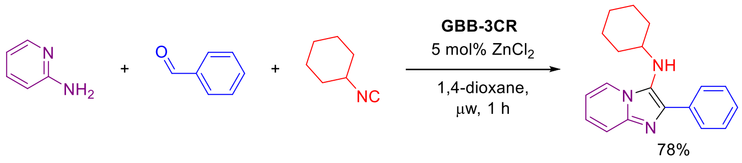

Raw material	Mass, mg	Product and waste	Mass, mg
2-aminopyridine	125.00	Product	302.30
Benzaldehyde	141.14	Waste	2,178.58
Cyclohexyl isocyanide	145.20	**Total**	2,480.88
$ZnCl_2$	9.54		
1,4-dioxane (2 mL)	2,060.00		
**Total**	2,480.88		

$$\mathbf{E-factor\ (E)} = \frac{2{,}178.58\ \text{mg of waste produced}}{302.30\ \text{mg of product}} = 7.21$$

$$\mathbf{Mass\ intensity} = \frac{2{,}480.88\ \text{mg of raw material used}}{302.30\ \text{mg of product}} = 8.21$$

Observation:

- Calculation did not consider the solvent system ethyl acetate/hexane used to afford the product as a precipitate.
- Calculation did not consider the solvent system 30% ethyl acetate/hexane, or 5% methanol/chloroform used for column chromatography.

5. Calculation for the synthesis of *N*-cyclohexyl-2-(thiophen-2-yl)imidazo[1,2-*a*]pyridin-3-amine in PEG-400 with $ZrCl_4$ [25]

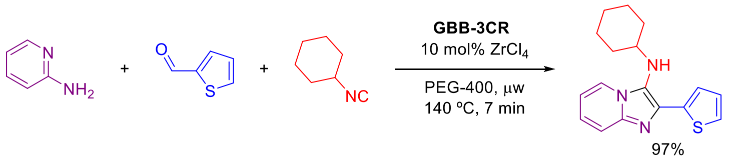

Raw material	Mass, mg	Product and waste	Mass, mg
2-aminopyridine	94.12	Product	288.50
2-thiophenecarboxaldehyde	112.15	Waste	1,180.24
Cyclohexyl isocyanide	109.17	**Total**	1,468.74
$ZrCl_4$	23.30		
PEG-400 (1 mL)	1,130.00		
**Total**	1,468.74		

$$\mathbf{E-factor\ (E)} = \frac{1{,}180.24\ \text{mg of waste produced}}{288.50\ \text{mg of product}} = 4.09$$

$$\mathbf{Mass\ intensity\ (PMI)} = \frac{1{,}468.74\ \text{mg of raw material used}}{288.50\ \text{mg of product}} = 5.09$$

Observation:

- Calculation did not consider the solvent ethyl acetate (60 mL) used in the extraction step.
- Calculation did not consider the solvent system ethyl acetate/hexane (1:1.5) used for column chromatography.

6. Calculation for the synthesis of *N*-cyclohexyl-2-(4-nitrophenyl)imidazo[1,2-*a*]pyridin-3-amine in EtOH with HPW (**this work**)

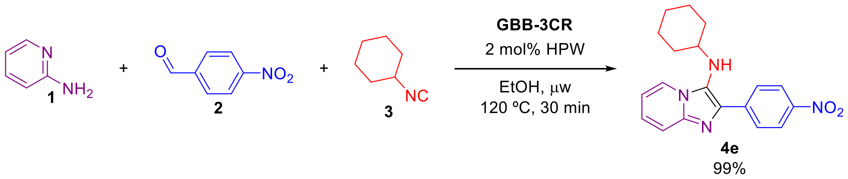

Raw material	Mass, mg	Product and waste	Mass, mg
2-aminopyridine	47.06	Product	166.52
4-nitrobenzaldehyde	75.56	Waste	433.99
Cyclohexyl isocyanide	54.59	**Total**	600.51
HPW	28.80		
EtOH (0.5 mL)	394.50		
**Total**	600.51		

$$\mathbf{E-factor\ (E)} = \frac{433.99 \text{ mg of waste produced}}{166.52 \text{ mg of product}} = 2.61$$

$$\mathbf{Mass\ intensity} = \frac{600.51 \text{ mg of raw material used}}{166.52 \text{ mg of product}} = 3.61$$

Observation:

- Calculation did not consider the solvent system ethyl acetate/hexane used for column chromatography.

3. References

[1] Ugi, I.; Meyr, R.; Lipinski, M.; Bodesheim, F.; Rosendahl, F. *Org. Synth.* **1961**, *41*, 13. doi:10.15227/orgsyn.041.0013

[2] Zhang, B.; Kurpiewska, K.; Dömling, A. *J. Org. Chem.* **2022**, *87*, 7085–7096. doi:10.1021/acs.joc.2c00244

[3] Hussain, M.; Liu, J.; Fu, L.; Hasan, M. *J. Heterocycl. Chem.* **2020**, *57*, 955–964. doi:10.1002/jhet.3746

[4] Singh, H. K.; Kamal, A.; Kumari, S.; Kumar, D.; Maury, S. K.; Srivastava, V.; Singh, S. *ACS Omega* **2020**, *5*, 29854–29863. doi:10.1021/acsomega.0c03941

[5] Swami, S.; Agarwala, A.; Shrivastava, R. *Mol. Divers.* **2017**, *21*, 81–88. doi:10.1007/s11030-016-9699-2

[6] Shivhare, K. N.; Jaiswal, M. K.; Srivastava, A.; Tiwari, S. K.; Siddiqui, I. R. *New J. Chem.* **2018**, *42*, 16591–16601. doi:10.1039/c8nj03339k

[7] Bode, M. L.; Gravestock, D.; Moleele, S. S.; van der Westhuyzen, C. W.; Pelly, S. C.; Steenkamp, P. A.; Hoppe, H. C.; Khan, T.; Nkabinde, L. A. *Bioorg. Med. Chem.* **2011**, *19*, 4227–4237. doi:10.1016/j.bmc.2011.05.062

[8] Azizi, N.; Dezfooli, S. *Environ. Chem. Lett.* **2016**, *14*, 201–206. doi:10.1007/s10311-015-0541-3

[9] Rostamnia, S.; Hassankhani, A. *RSC Adv.* **2013**, *3*, 18626-18629. doi:10.1039/c3ra42752h

[10] Habibi, A.; Tarameshloo, Z.; Rostamizadeh, S.; M. Amani, A. *Lett. Org. Chem.* **2012**, *9*, 155–159. doi:10.2174/157017812800167439

[11] Ansari, A. J.; Sharma, S.; Pathare, R. S.; Gopal, K.; Sawant, D. M.; Pardasani, R. T. *ChemistrySelect* **2016**, *1*, 1016–1021. doi:10.1002/slct.201600241

[12] Shahrisa, A.; Esmati, S. *Synlett* **2013**, *24*, 595–602. doi:10.1055/s-0032-1318221

[13] Rousseau, A. L.; Matlaba, P.; Parkinson, C. J. *Tetrahedron Lett.* **2007**, *48*, 4079–4082. doi:10.1016/j.tetlet.2007.04.008

[14] Heydari, M.; Azizi, N.; Mirjafari, Z.; Hashemi, M. M. *J. Iran. Chem. Soc.* **2019**, *16*, 2357–2363. doi:10.1007/s13738-019-01705-3

[15] Xu, F.; Wang, Y.; Xun, X.; Huang, Y.; Jin, Z.; Song, B.; Wu, J. *J. Org. Chem.* **2019**, *84*, 8411–8422. doi:10.1021/acs.joc.9b00208

[16] Il'in, M. V.; Sysoeva, A. A.; Novikov, A. S.; Bolotin, D. S. *J. Org. Chem.* **2022**, *87*, 4569–4579. doi:10.1021/acs.joc.1c02885

[17] Vidyacharan, S.; Shinde, A. H.; Satpathi, B.; Sharada, D. S. *Green Chem.* **2014**, *16*, 1168-1175. doi:10.1039/c3gc42130a

[18] Han, X.; Ma, C.; Wu, Z.; Huang, G. *Synthesis* **2015**, *48*, 351–356. doi:10.1055/s-0035-1560375

[19] Santos, G. F. D.; Anjos, N. S.; Gibeli, M. M.; Silva, G. A.; Fernandes, P. C. S.; Fiorentino, E. S. C.; Longo, L. S. *J. Braz. Chem. Soc.* **2020**, *31*, 1434–1444. doi:10.21577/0103-5053.20200028

[20] Mathavan, S.; B. R. D. Yamajala, R. *ChemistrySelect* **2020**, *5*, 10637–10642. doi:10.1002/slct.202002894

[21] Ghorbani-Vaghei, R.; Amiri, M. *J. Heterocycl. Chem.* **2014**, *51*, E372-E379. doi:10.1002/jhet.1875

[22] Shukla, N. M.; Salunke, D. B.; Yoo, E.; Mutz, C. A.; Balakrishna, R.; David, S. A. *Bioorg. Med. Chem.* **2012**, *20*, 5850–5863. doi:10.1016/j.bmc.2012.07.052

[23] Shinde, A. H.; Srilaxmi, M.; Satpathi, B.; Sharada, D. S. *Tetrahedron Lett.* **2014**, *55*, 5915–5920. doi:10.1016/j.tetlet.2014.08.126

[24] Budhiraja, M.; Kondabala, R.; Ali, A.; Tyagi, V. *Tetrahedron* **2020**, *76*. doi:10.1016/j.tet.2020.131643

[25] Guchhait, S. K.; Madaan, C.; Thakkar, B. S. *Synthesis* **2009**, 3293–3300. doi:10.1055/s-0029-1216916

4. FTIR, ^{1}H and ^{13}C NMR, HRMS and HRMS/MS spectra for all compounds

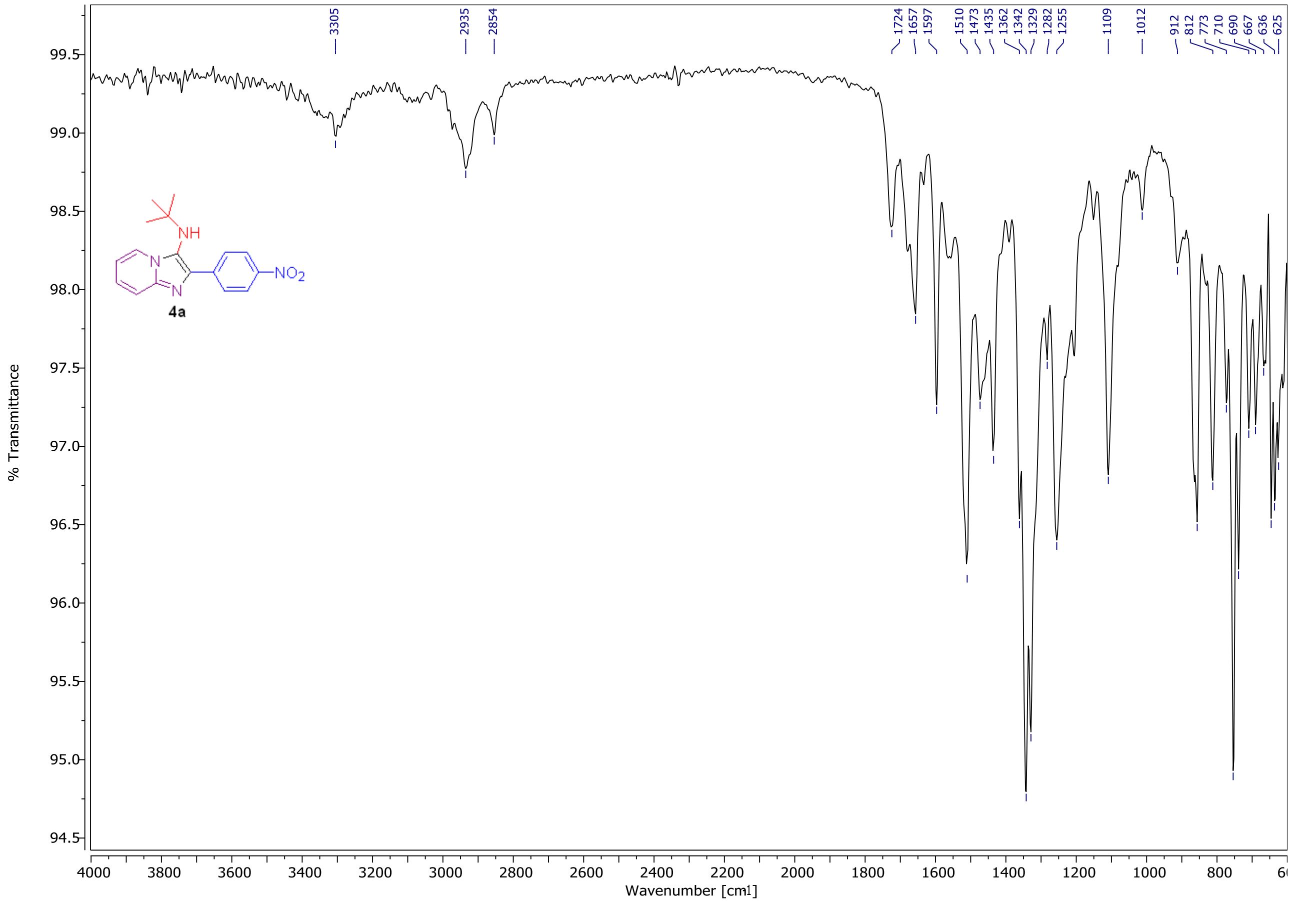

S 1. FT-IR (ATR) of compound **4a**.

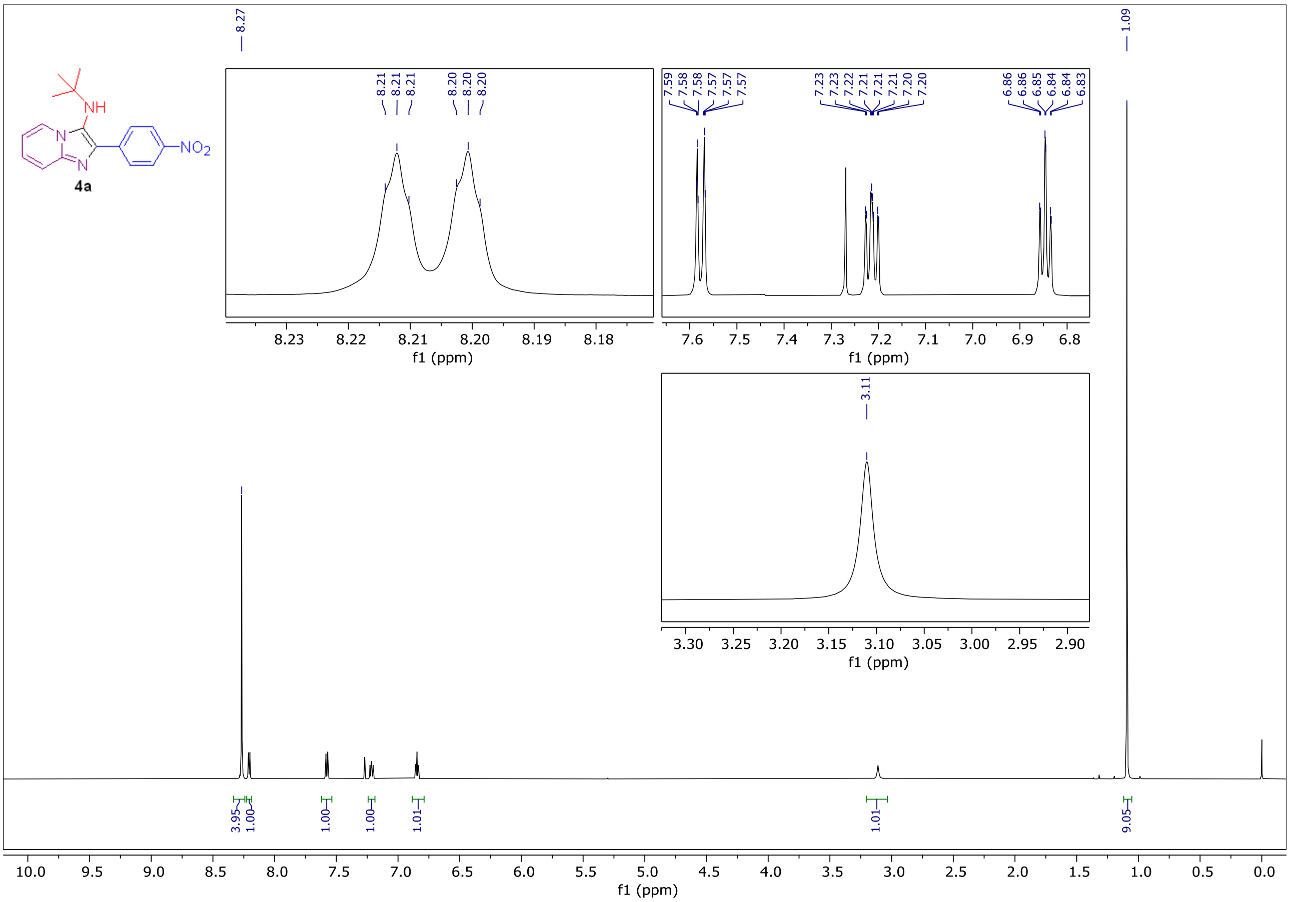

S 1. ^{1}H NMR spectrum (600 MHz, $CDCl_3$) of compound **4a**.

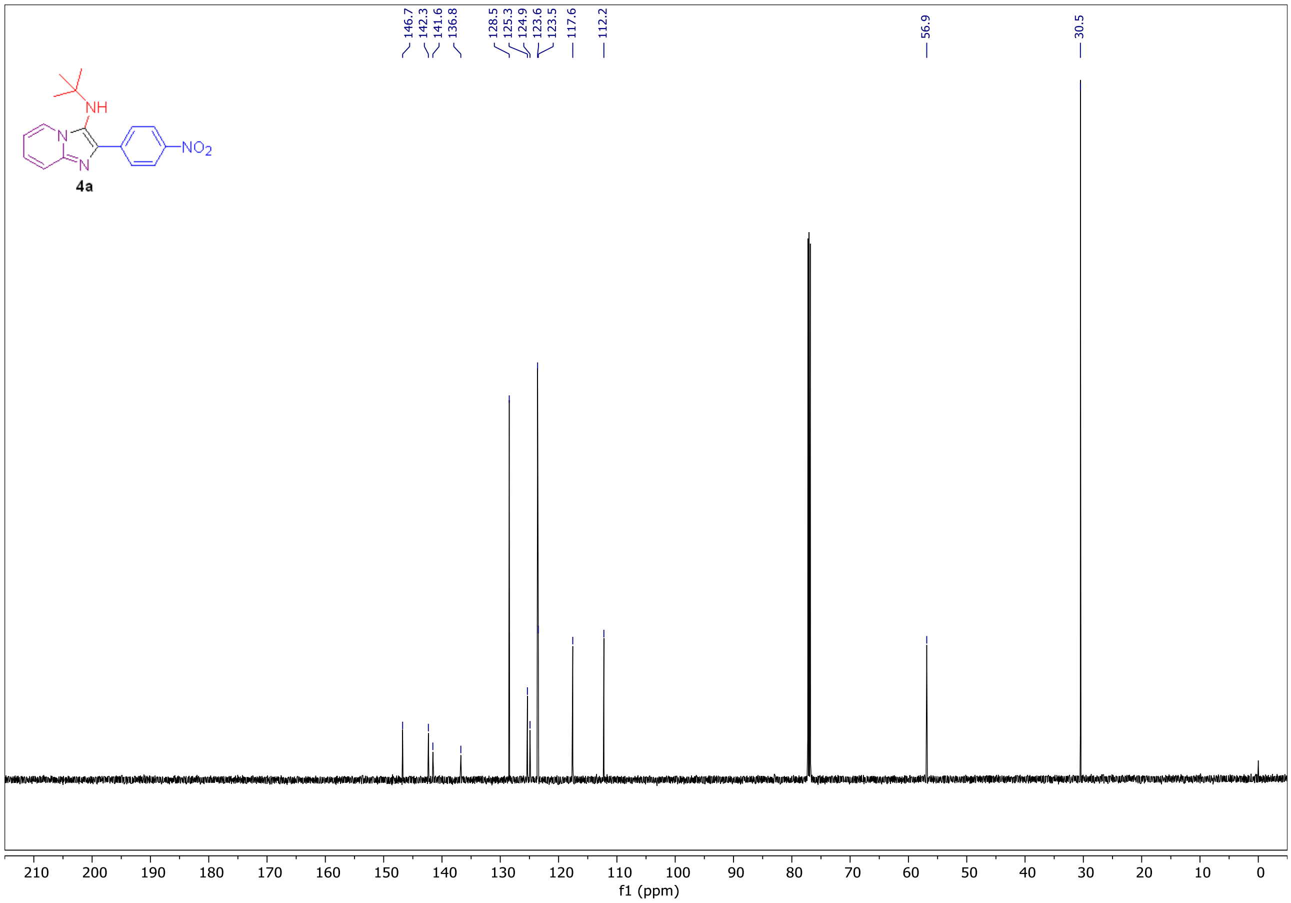

S 3. ^{13}C NMR spectrum (151 MHz, $CDCl_3$) of compound **4a**.

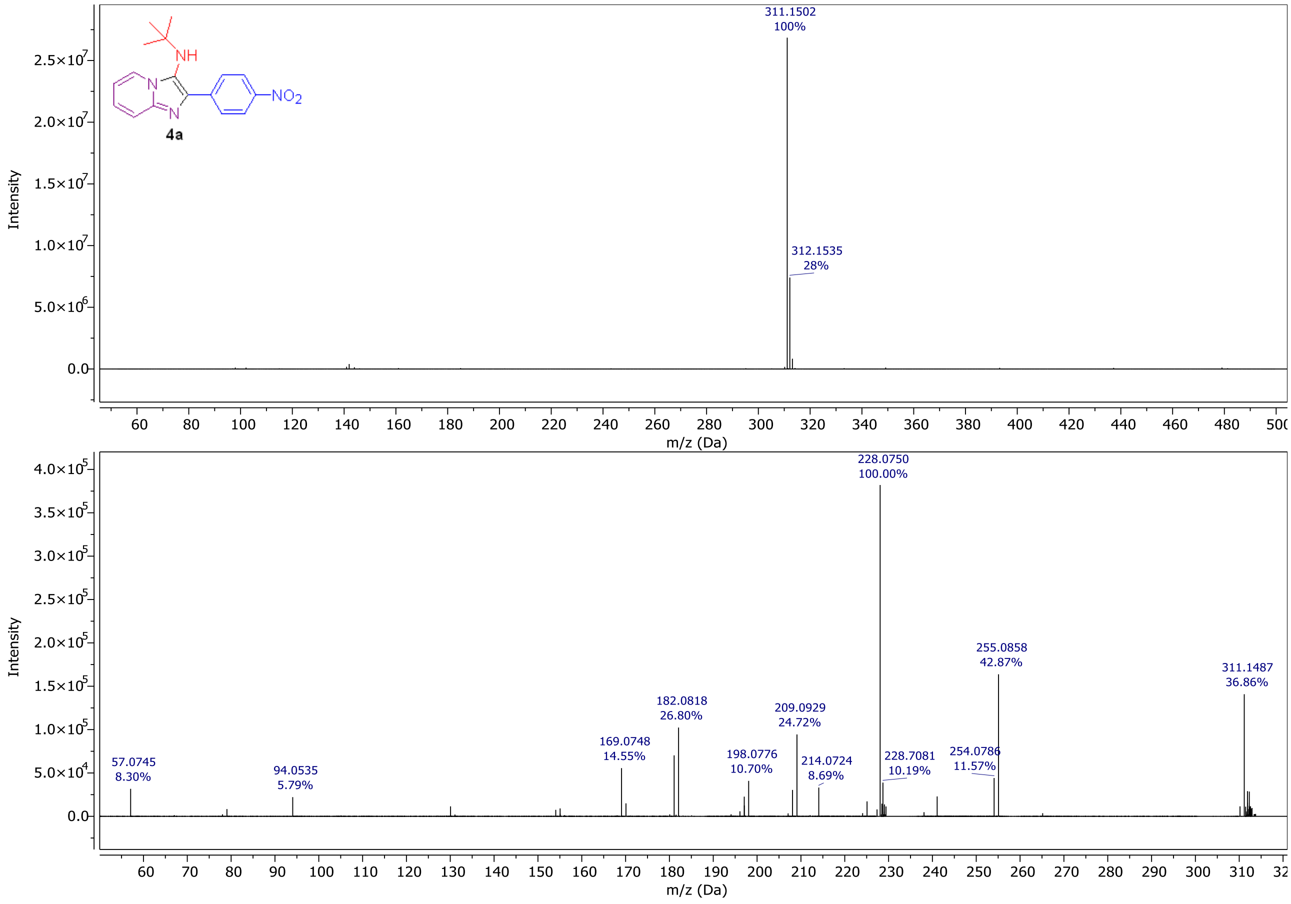

S 4. HRMS (ESI-QTOF) of compound **4a** and HRMS/MS for $[M+H]^+$.

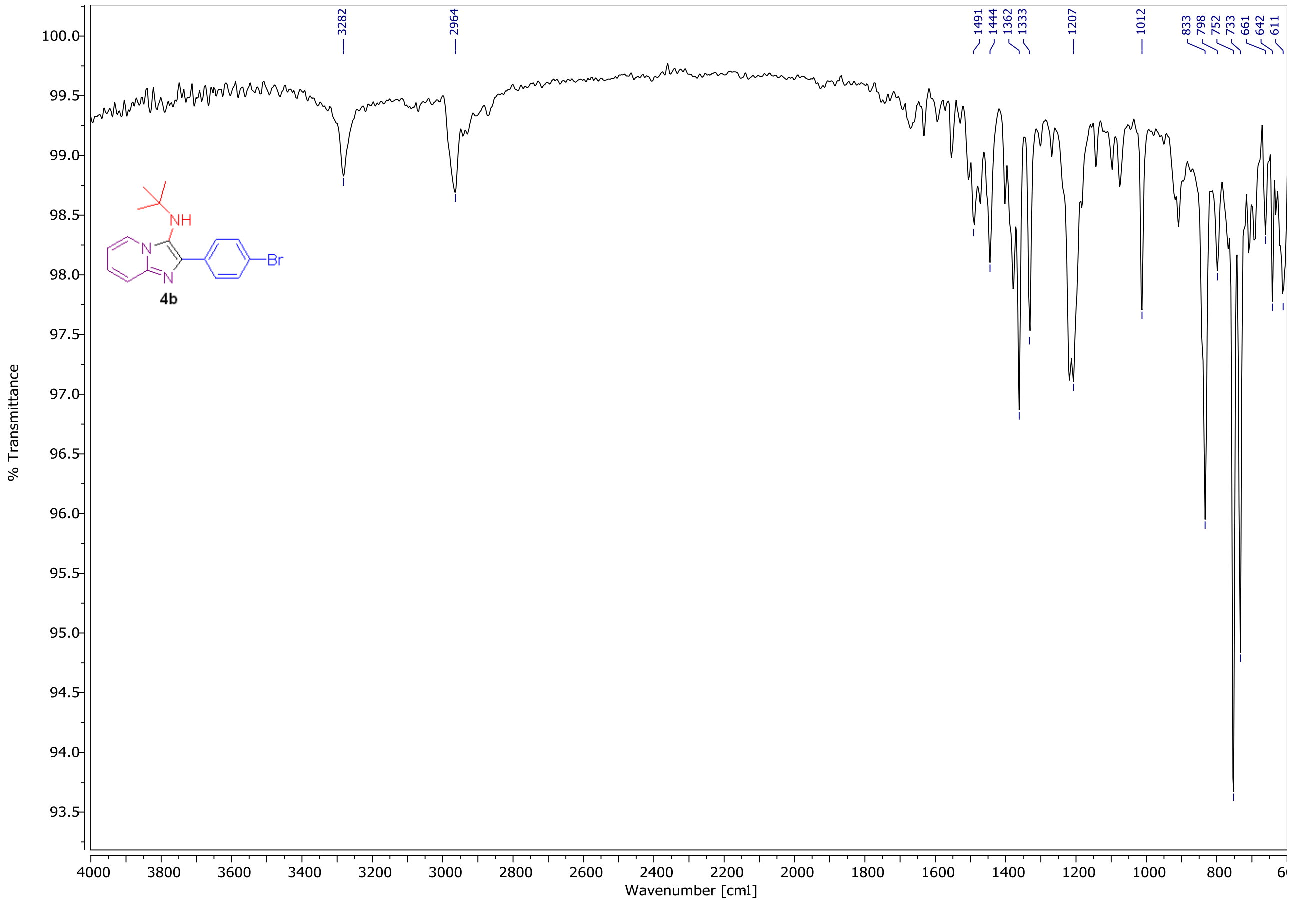

S 5. FT-IR (ATR) of compound **4b**.

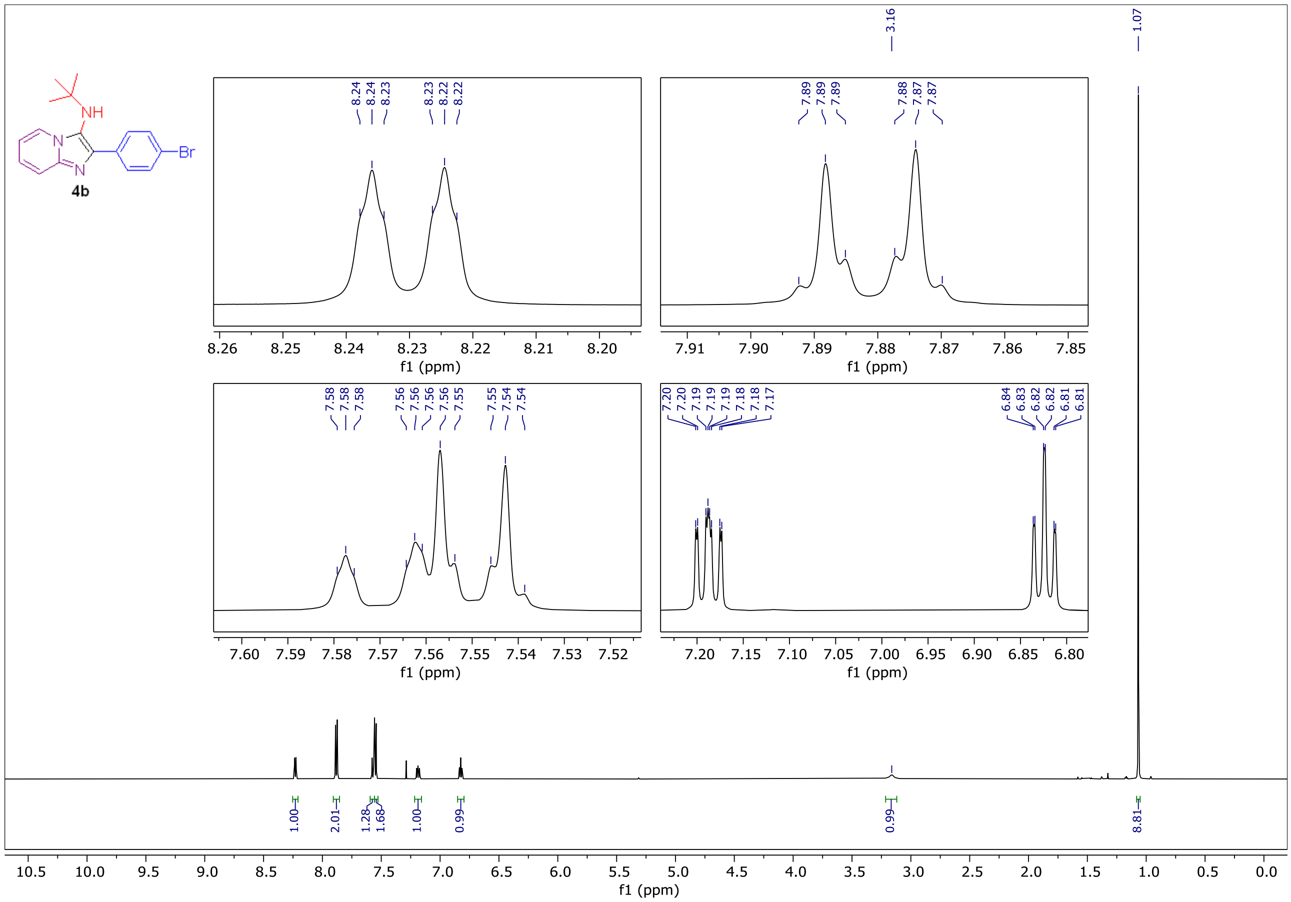

S 6. ^{1}H NMR spectrum (600 MHz, $CDCl_3$) of compound **4b**.

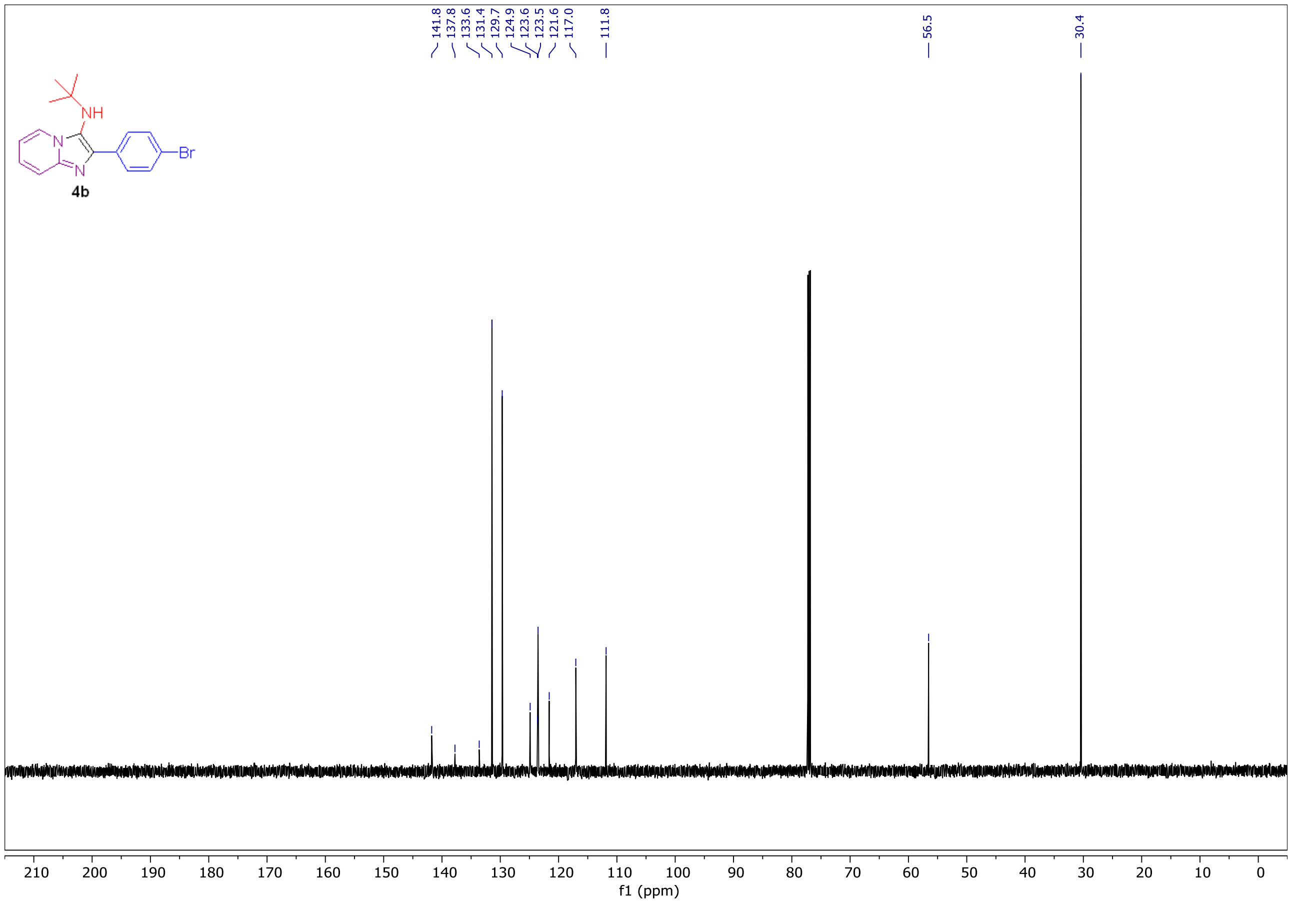

S 7. ^{13}C NMR spectrum (151 MHz, $CDCl_3$) of compound **4b**.

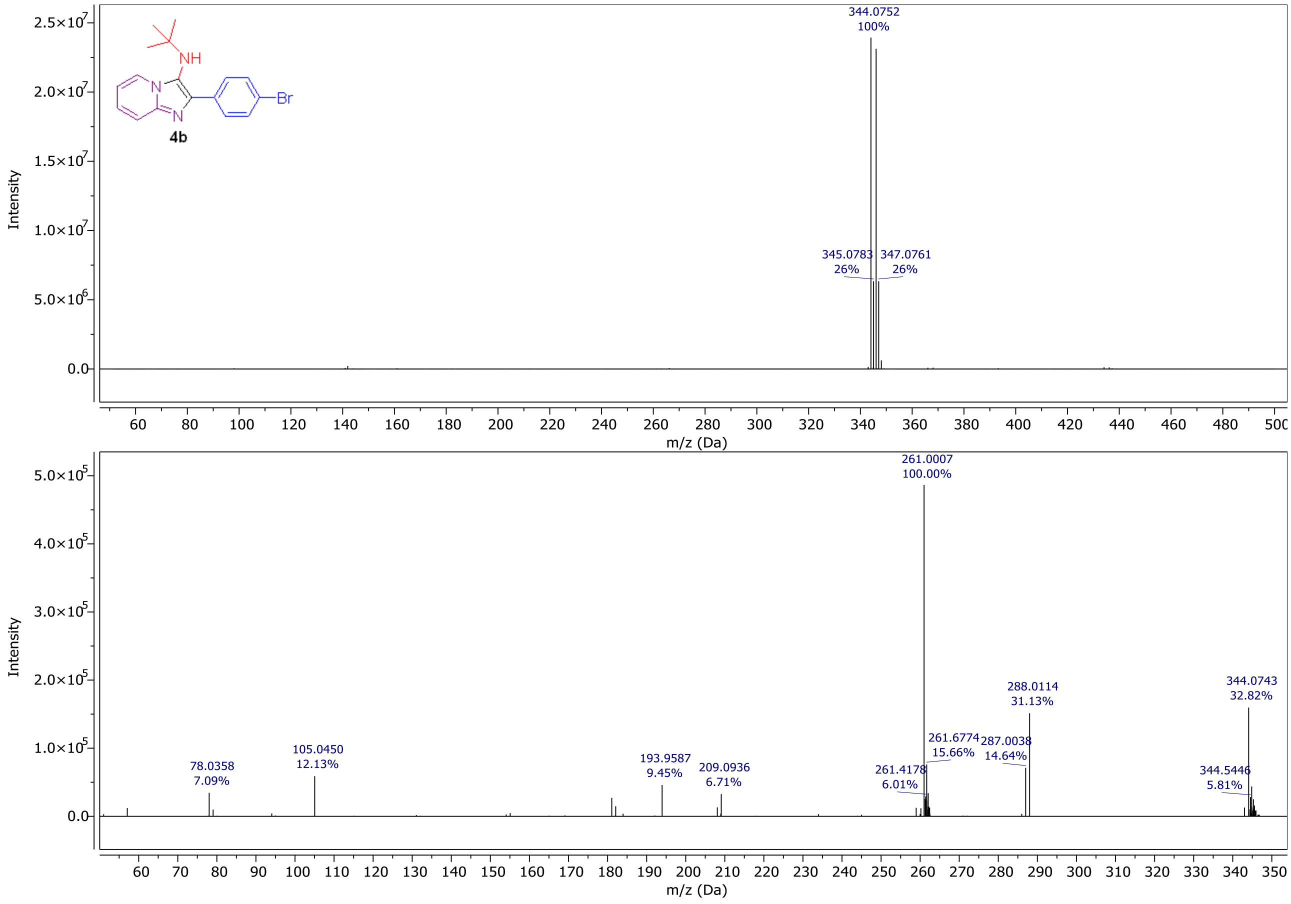

S 8. HRMS (ESI-QTOF) of compound **4b** and HRMS/MS for $[M+H]^+$.

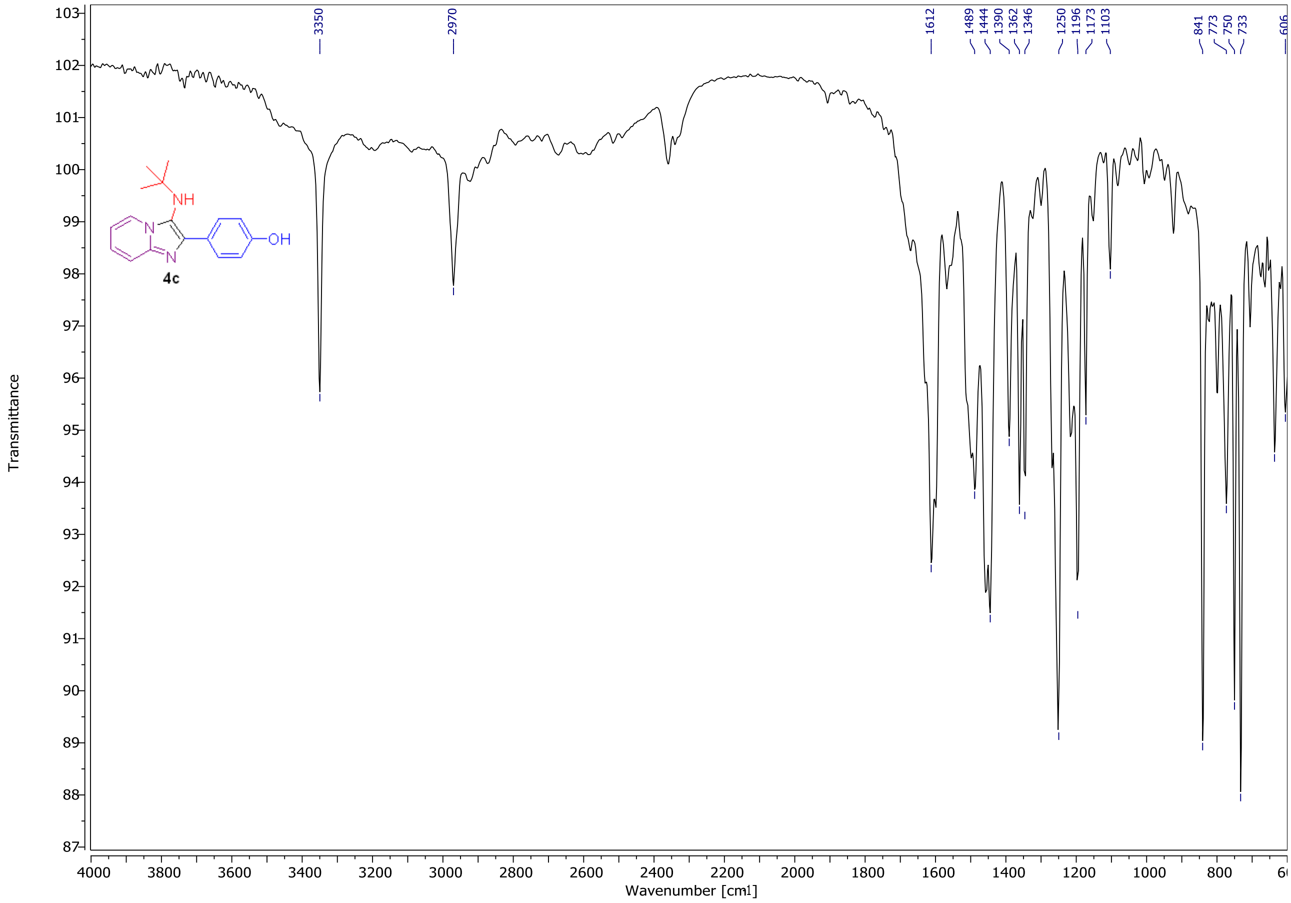

S 9. FT-IR (ATR) of compound **4c**.

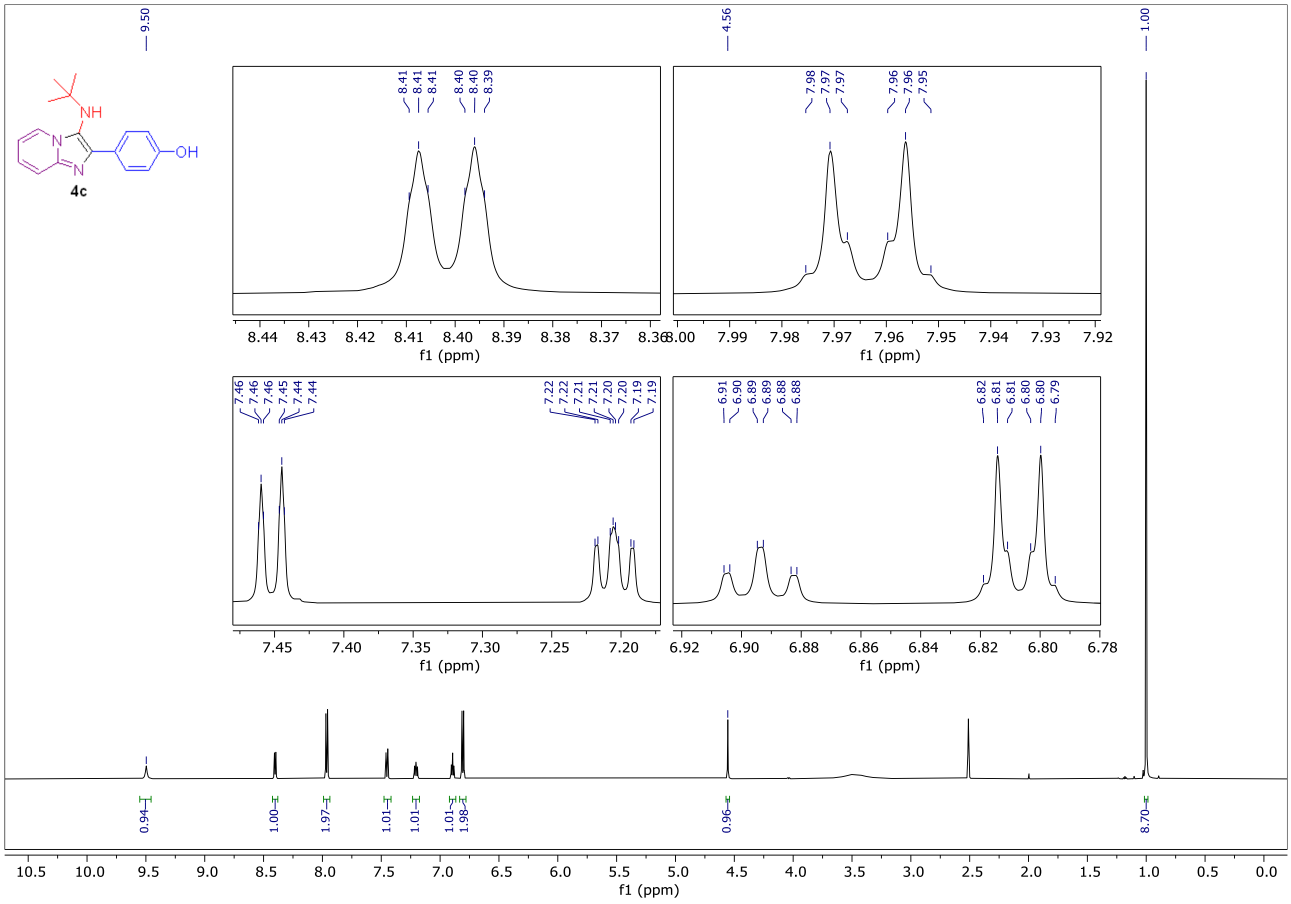

S 10. ^{1}H NMR spectrum (600 MHz, $CDCl_3$) of compound **4c**.

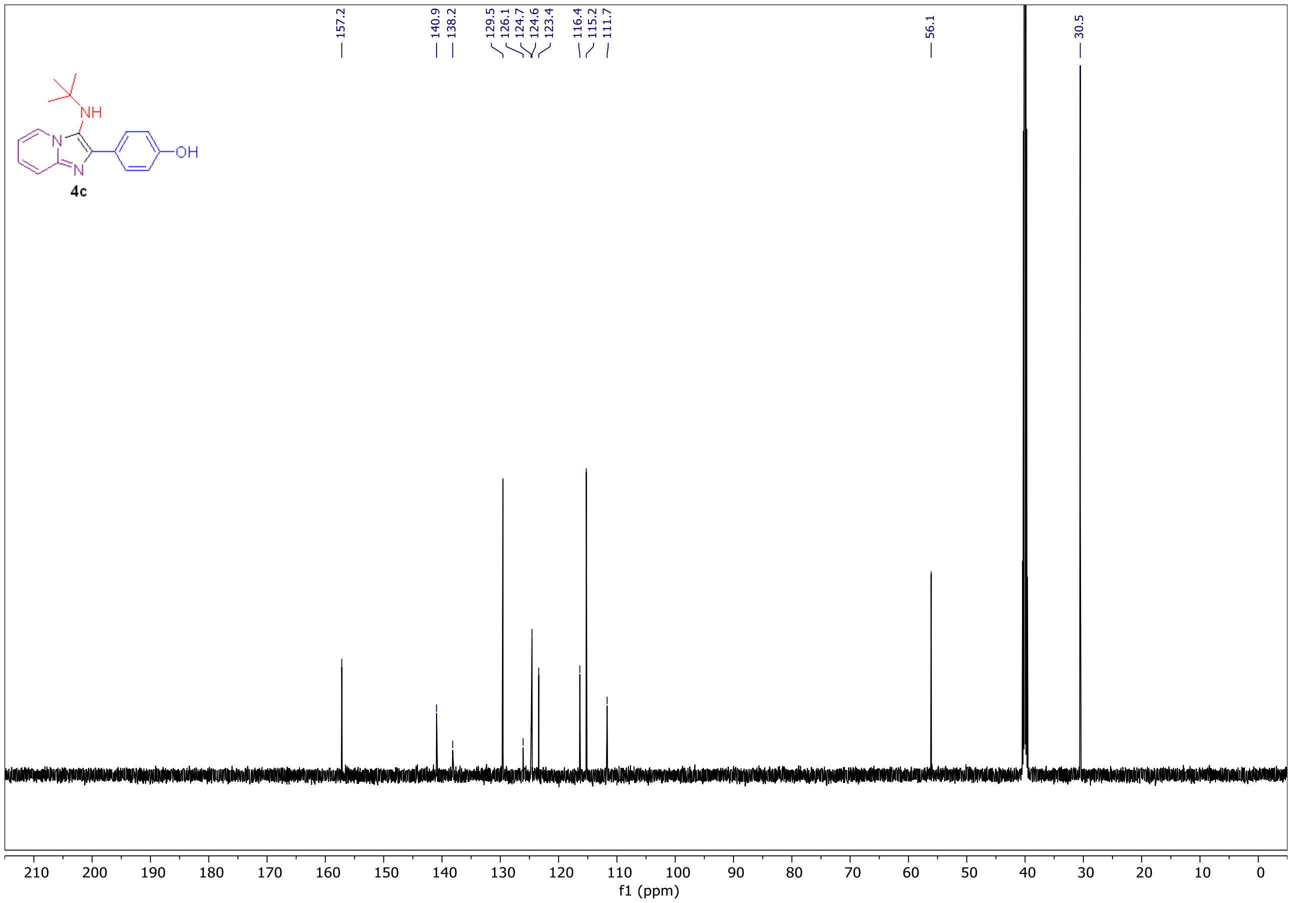

S 11. ^{13}C NMR spectrum (151 MHz, $CDCl_3$) of compound **4c**.

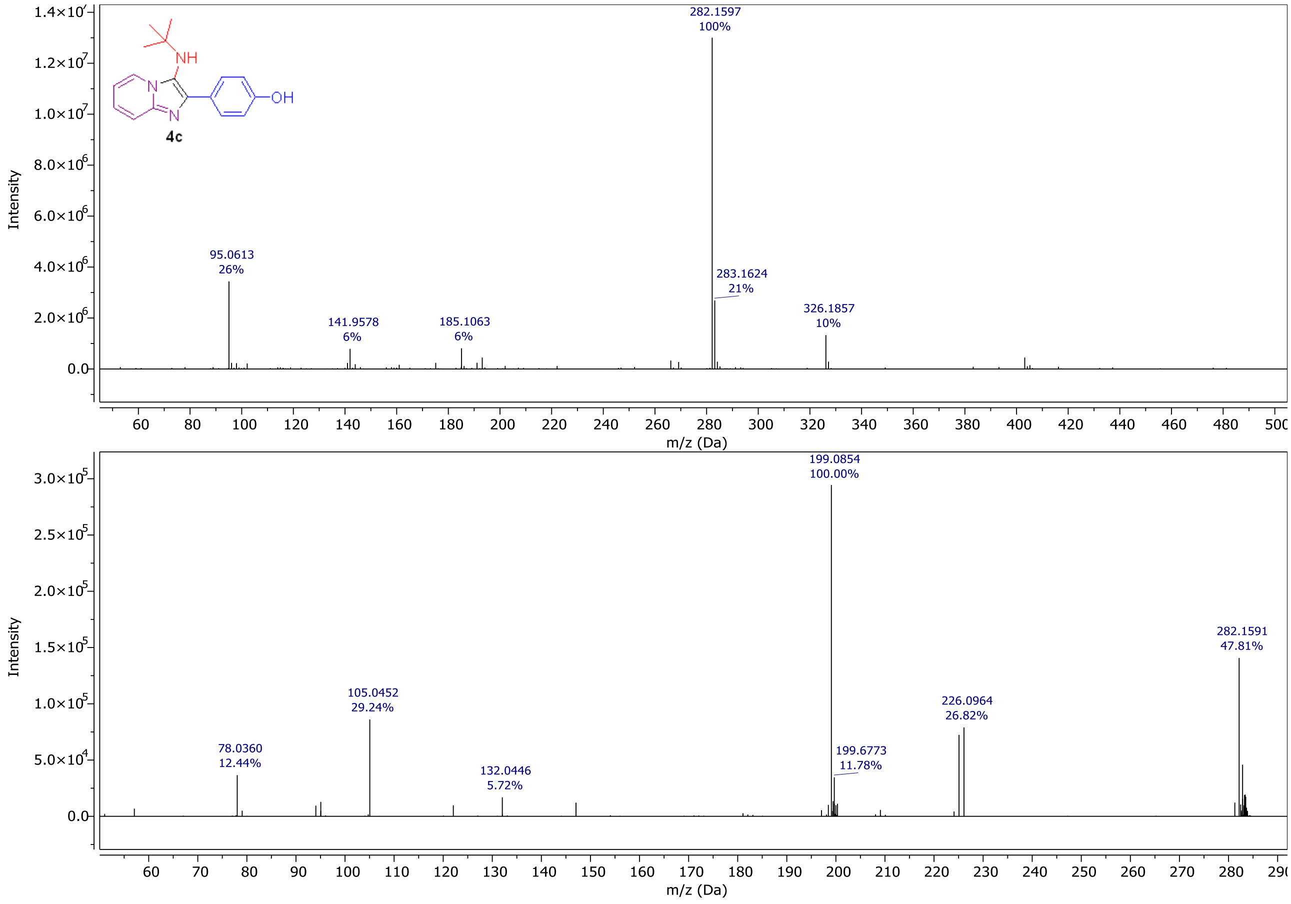

S 12. HRMS (ESI-QTOF) of compound **4c** and HRMS/MS for $[M+H]^+$.

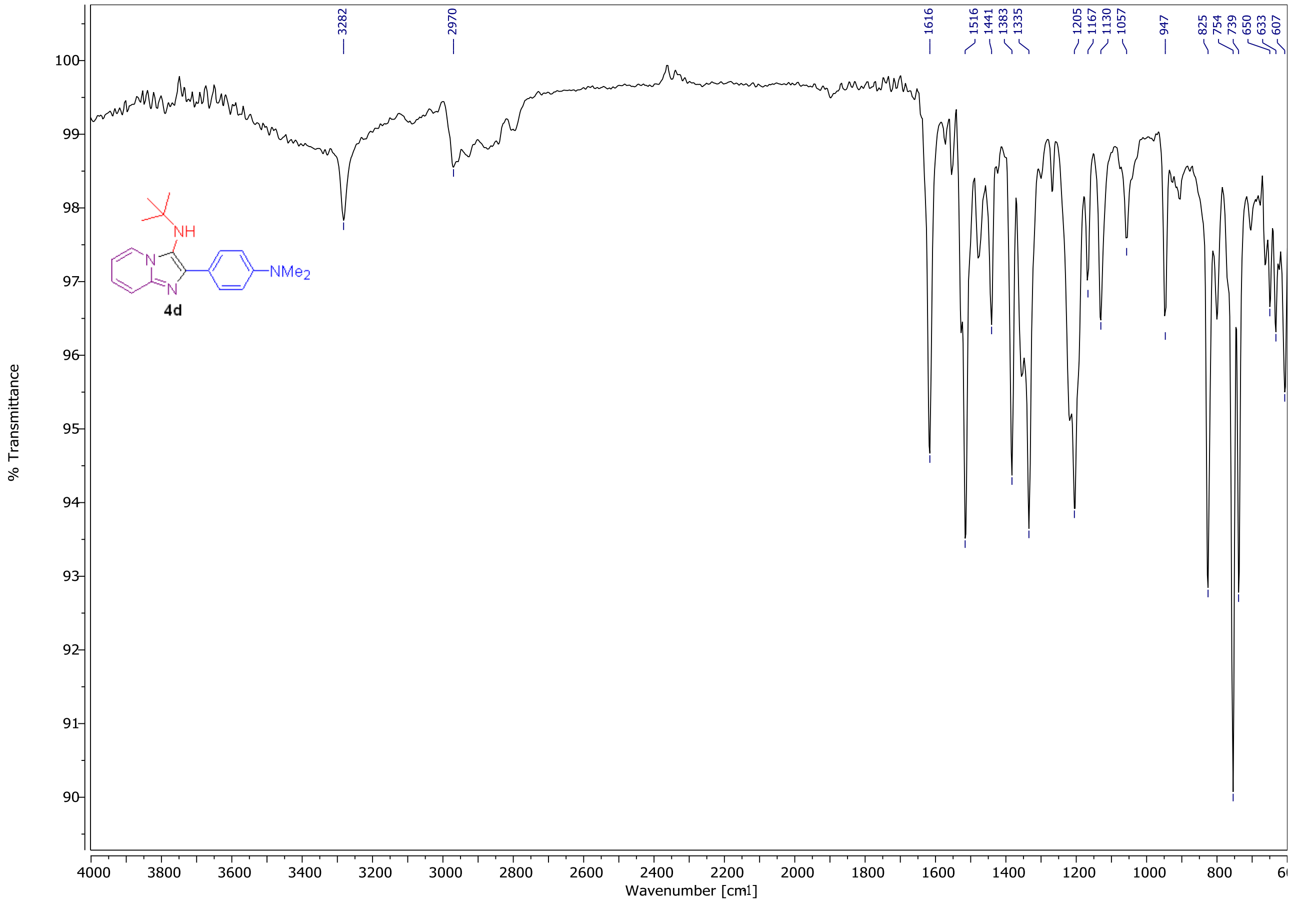

S 13. FT-IR (ATR) of compound **4d**.

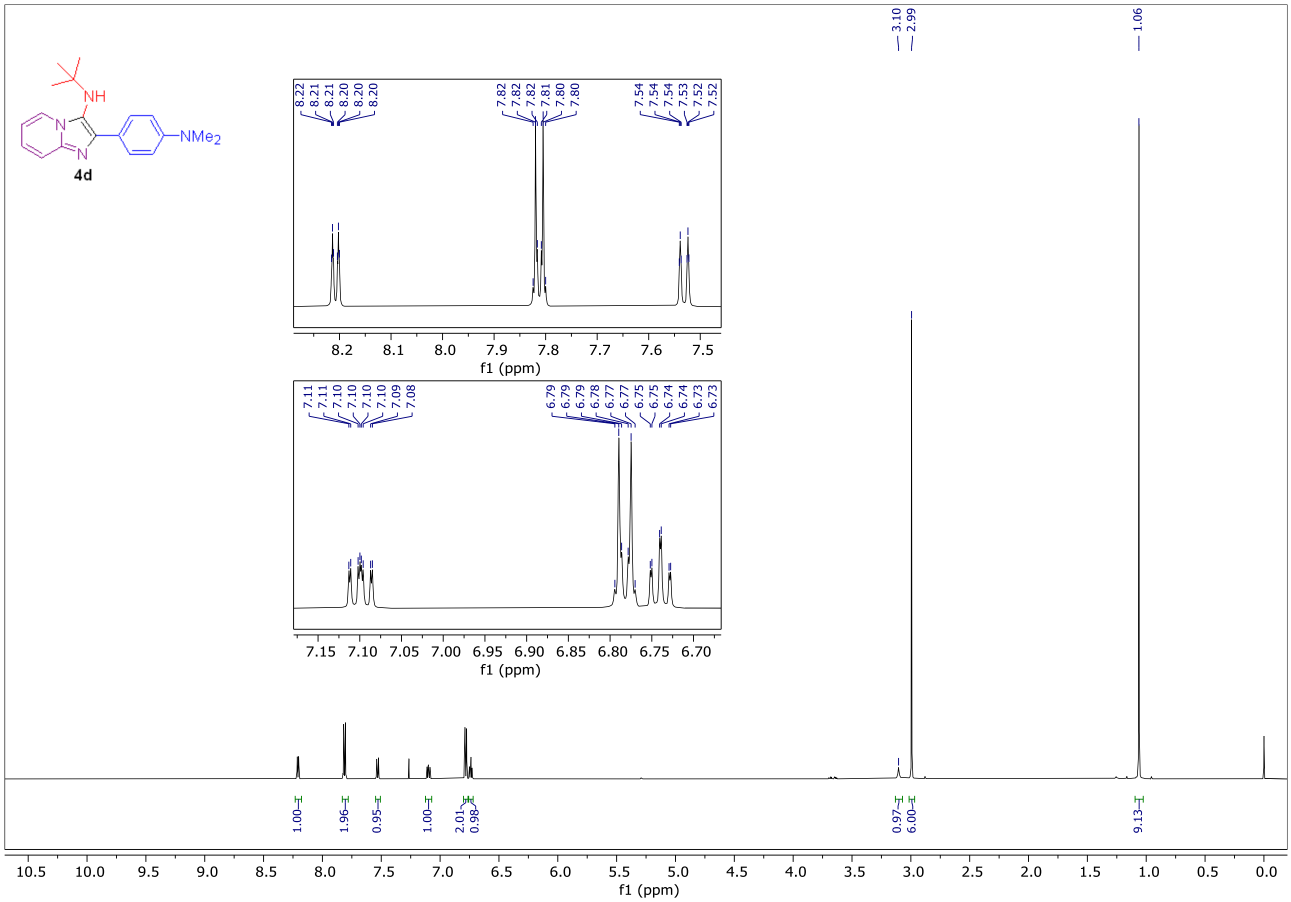

S 14. ^{1}H NMR spectrum (600 MHz, $CDCl_3$) of compound **4d**.

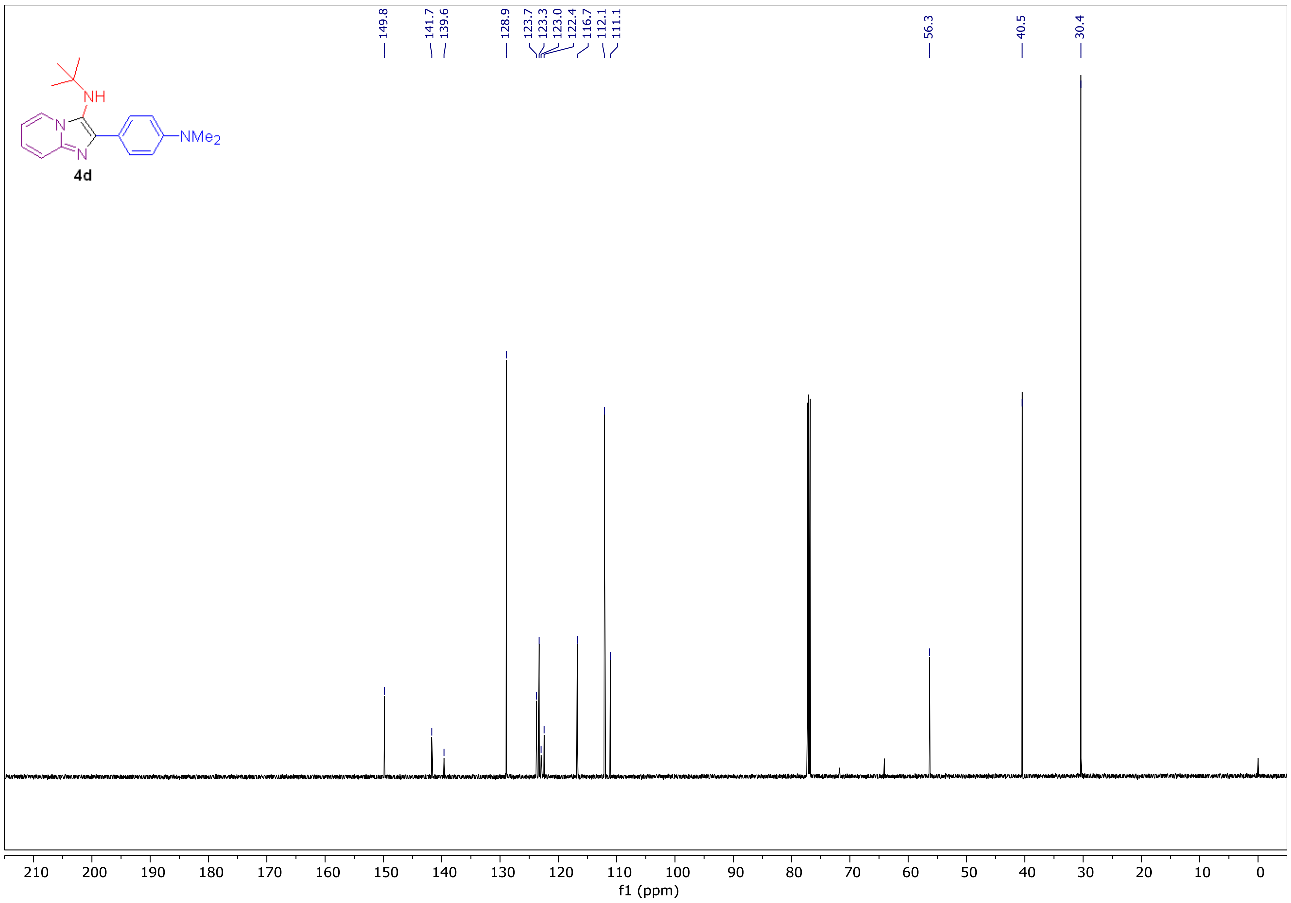

S 15. ^{13}C NMR spectrum (151 MHz, $CDCl_3$) of compound **4d**.

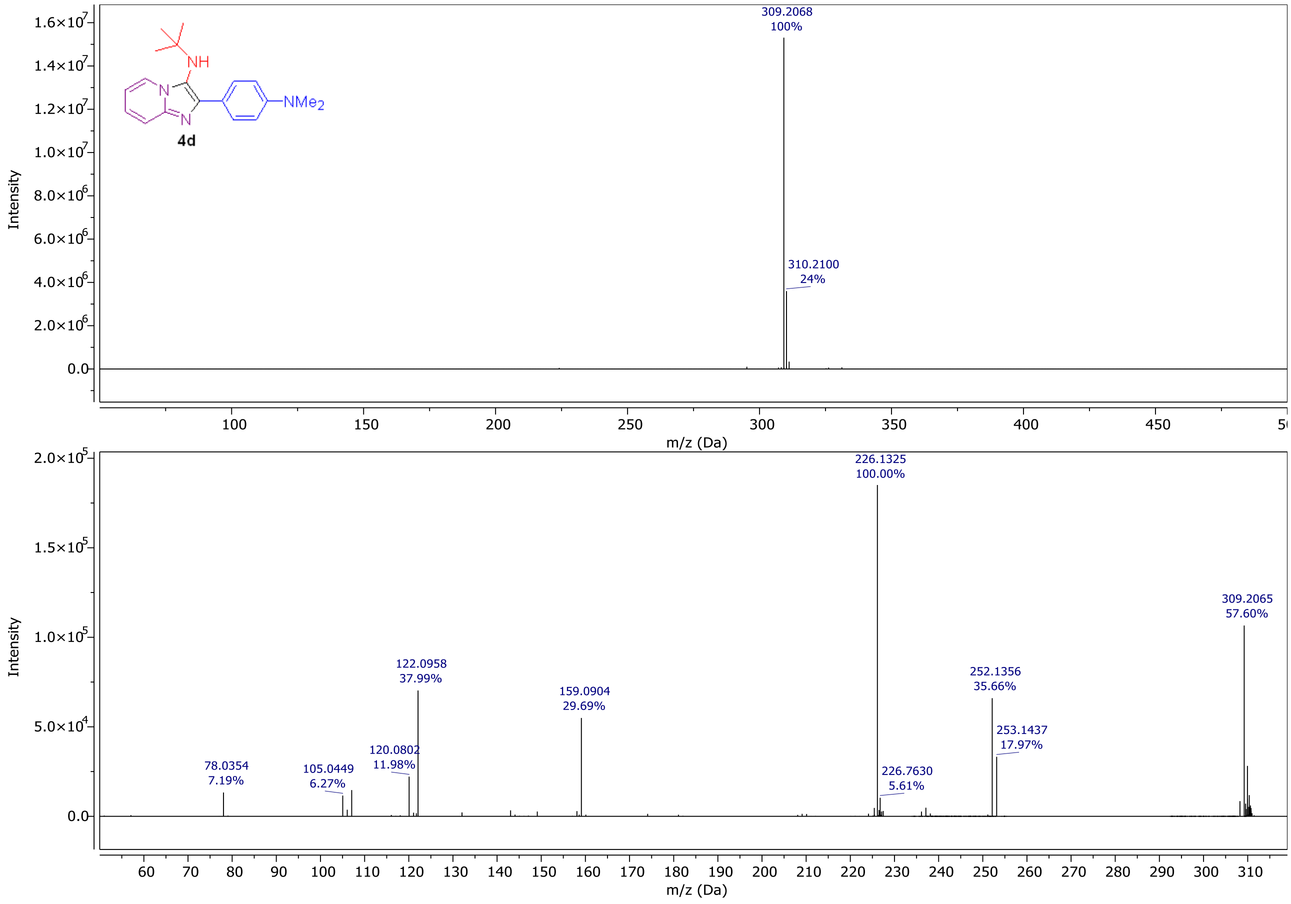

S 16. HRMS (ESI-QTOF) of compound **4d** and HRMS/MS for $[M+H]^+$.

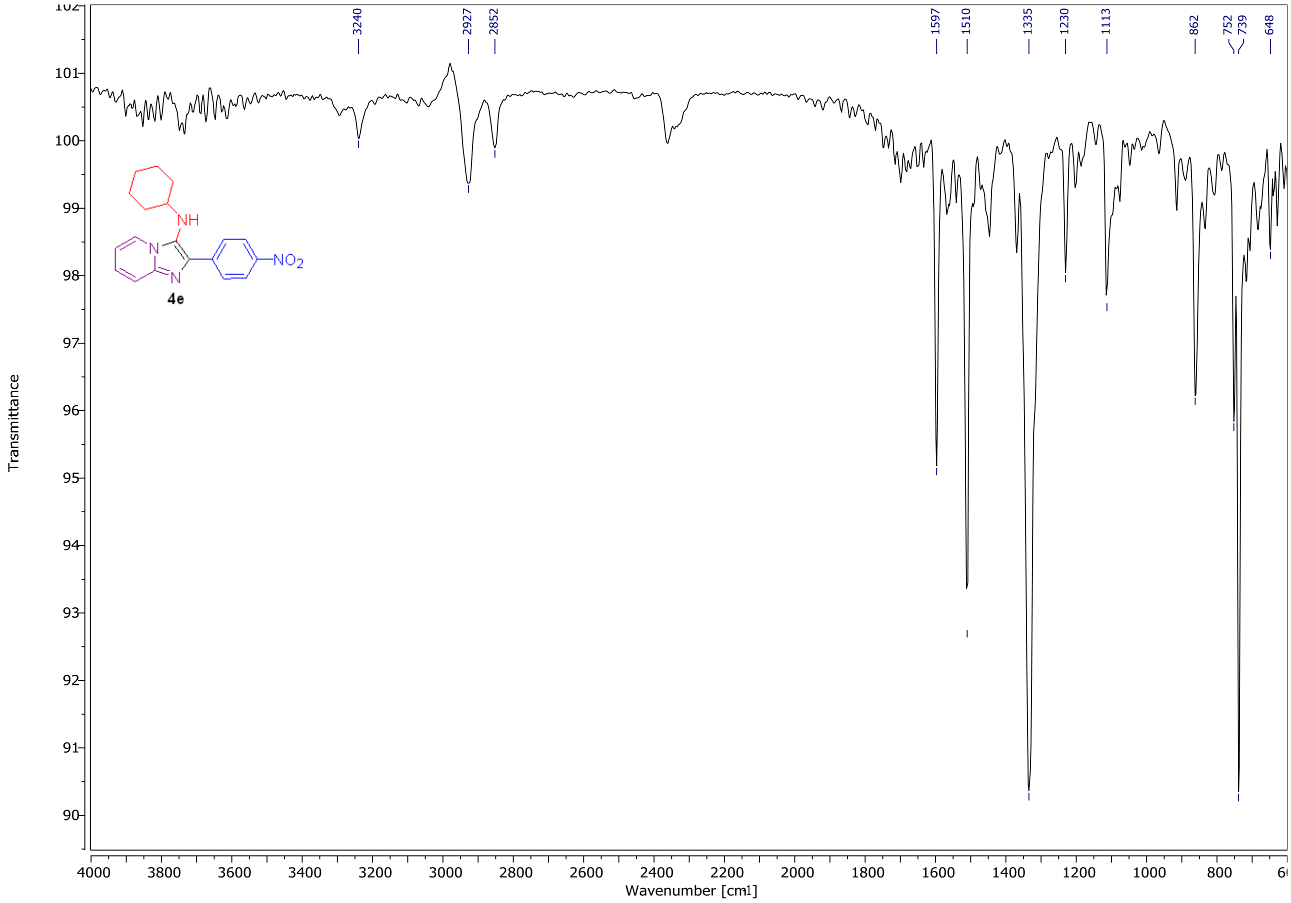

S 17. FT-IR (ATR) of compound **4e**.

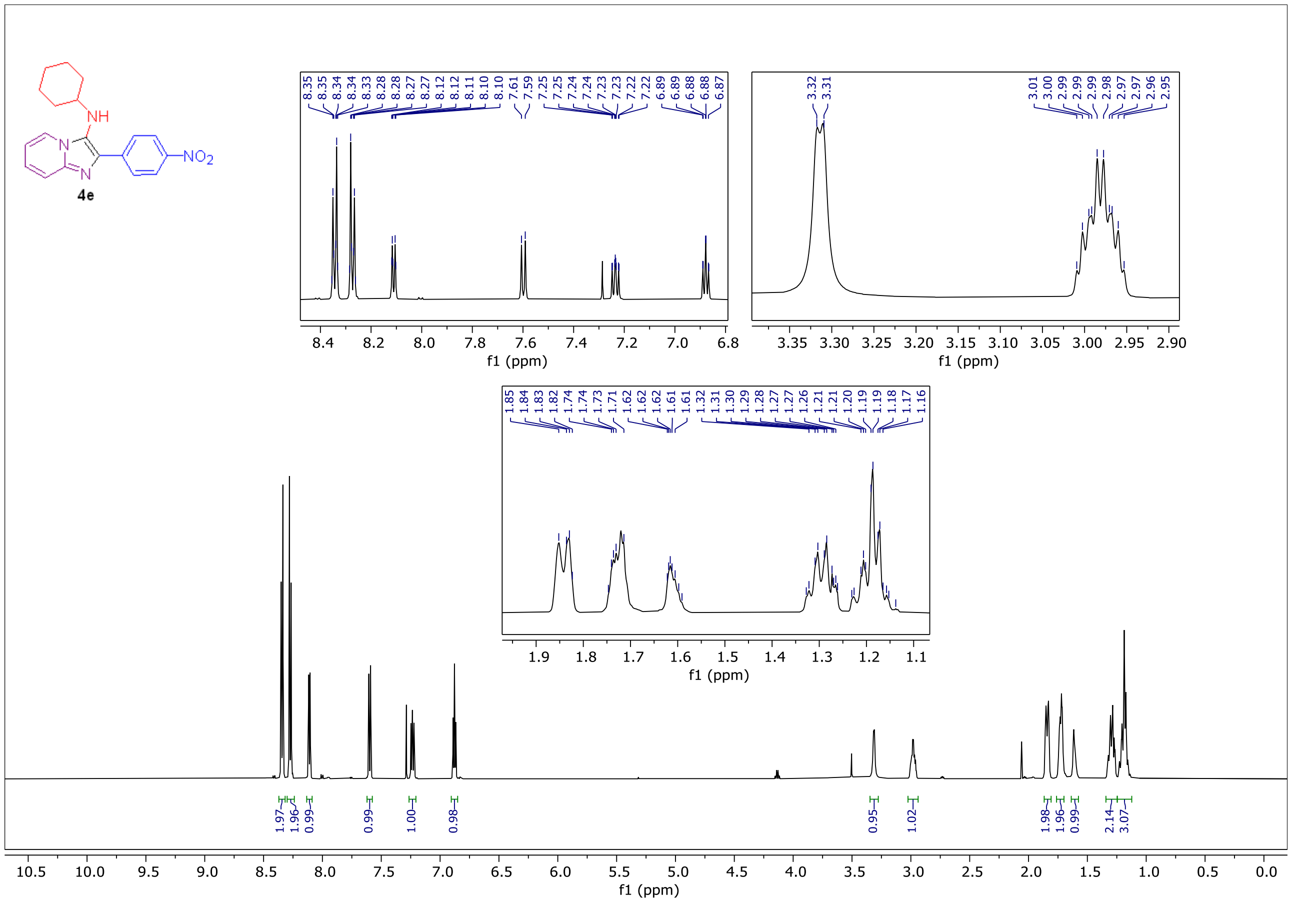

S 18. ^{1}H NMR spectrum (600 MHz, $CDCl_3$) of compound **4e**.

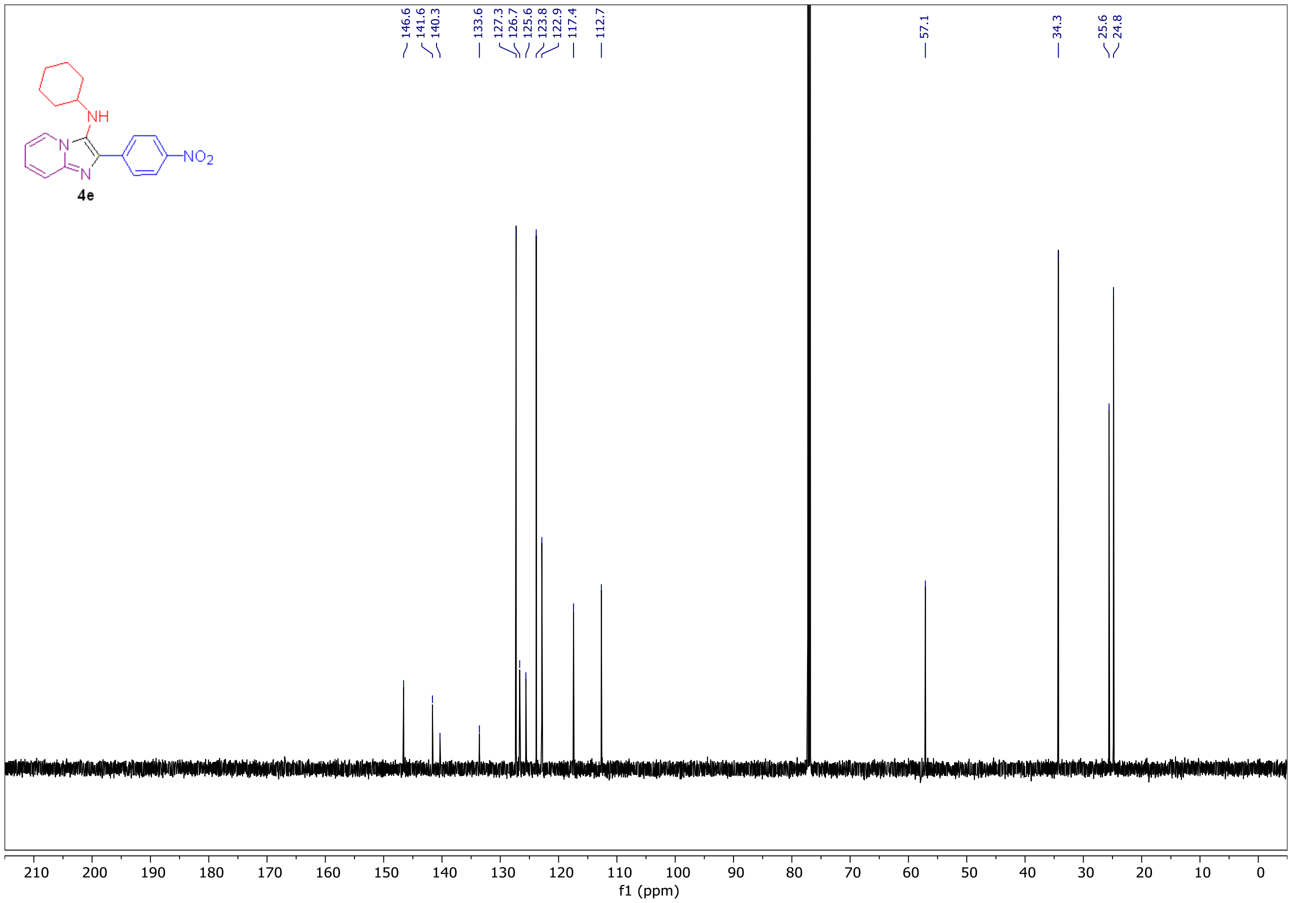

S 19. ^{13}C NMR spectrum (151 MHz, $CDCl_3$) of compound **4e**.

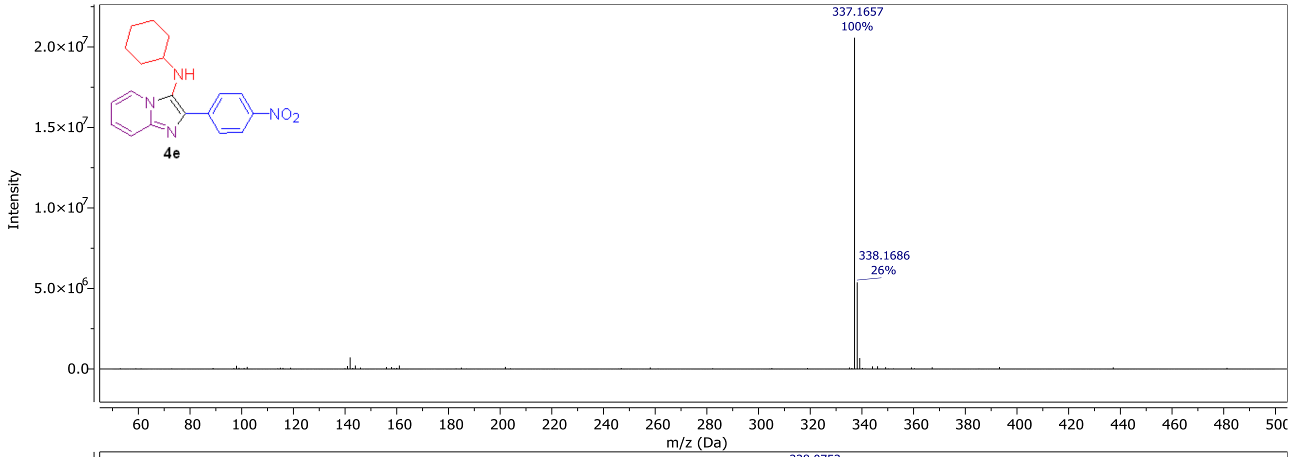

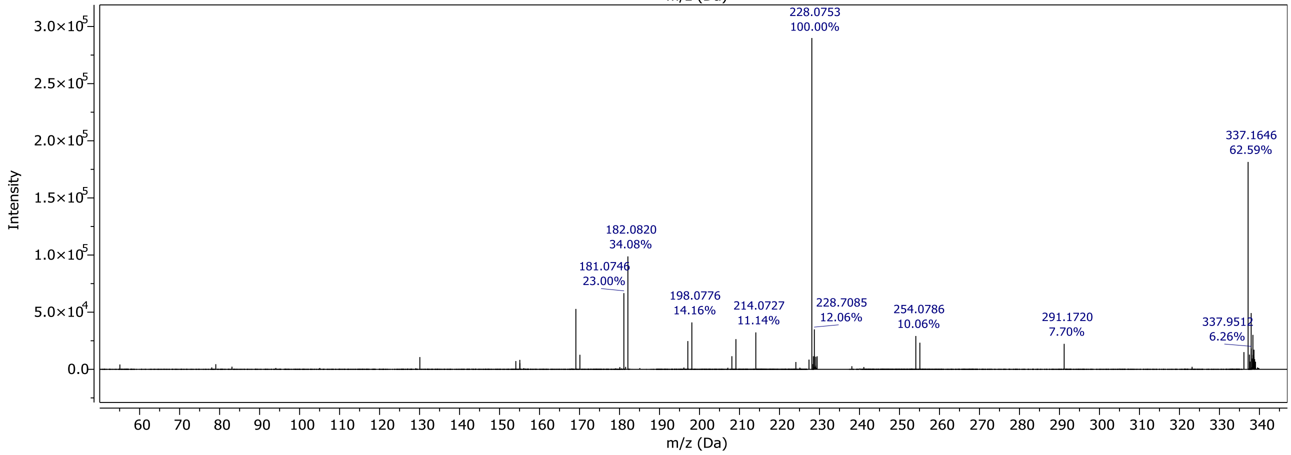

S 20. HRMS (ESI-QTOF) of compound **4e** and HRMS/MS for [M+H]$^+$.

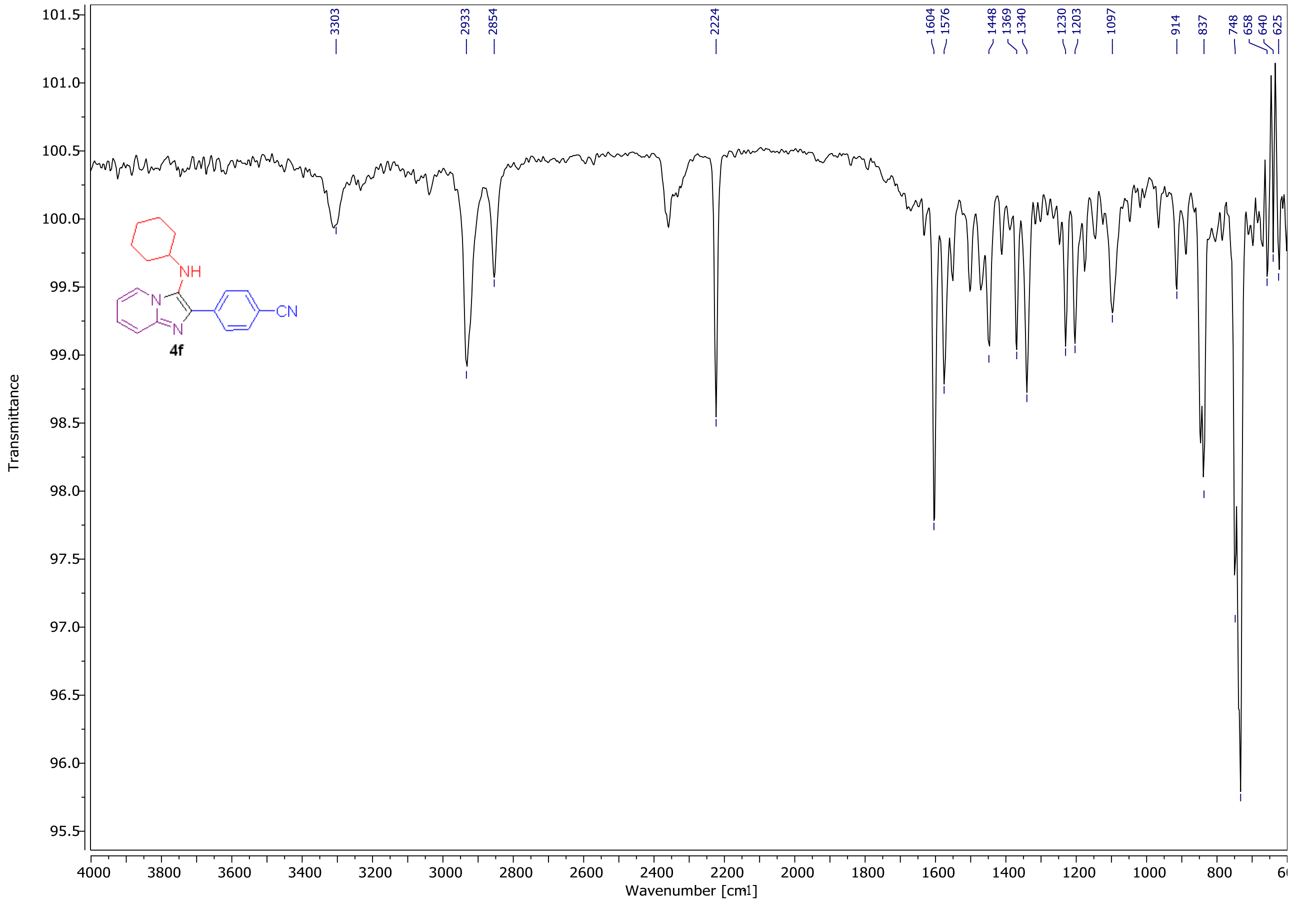

S 21. FT-IR (ATR) of compound **4f**.

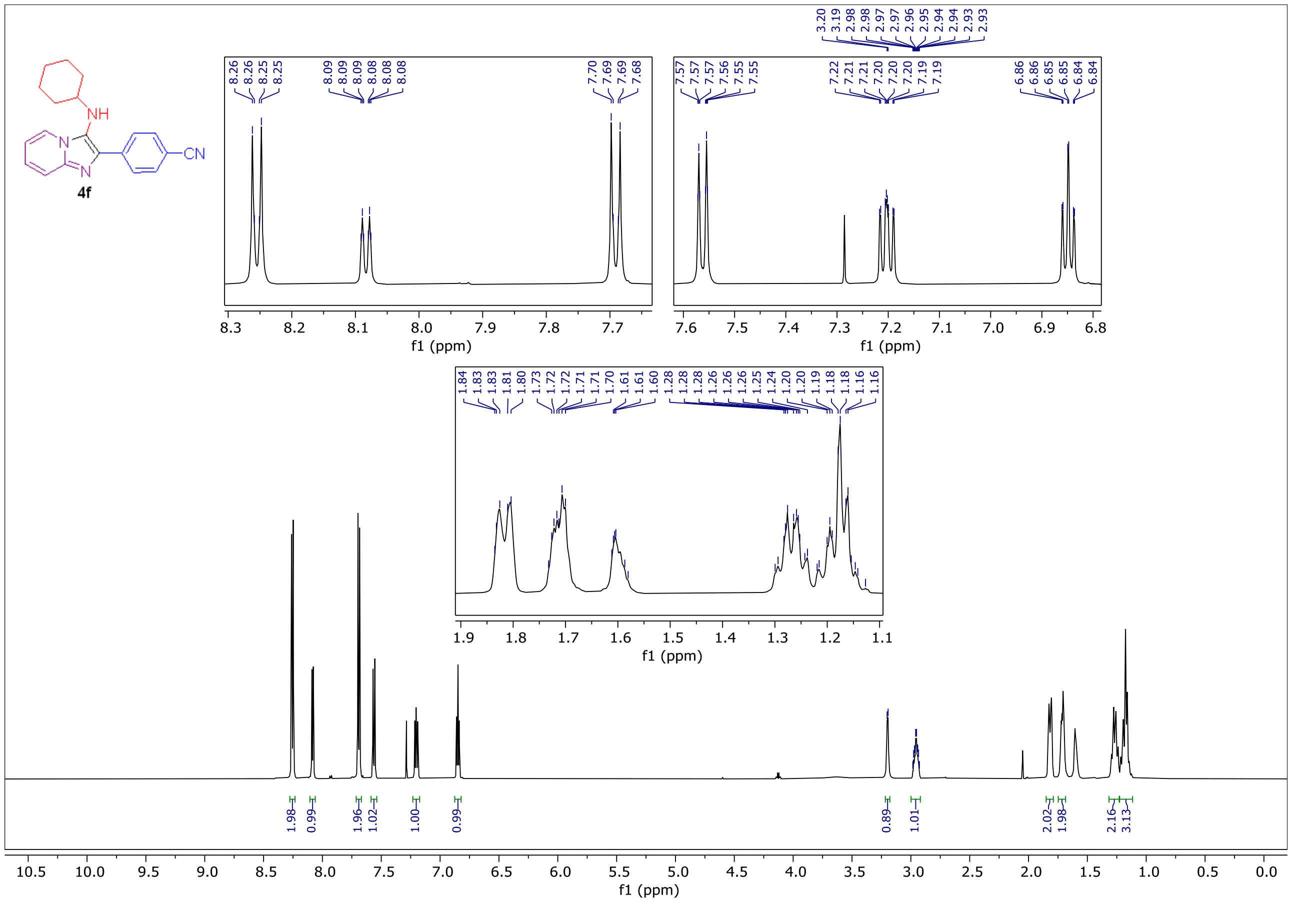

S 22. ¹H NMR spectrum (600 MHz, $CDCl_3$) of compound **4f**.

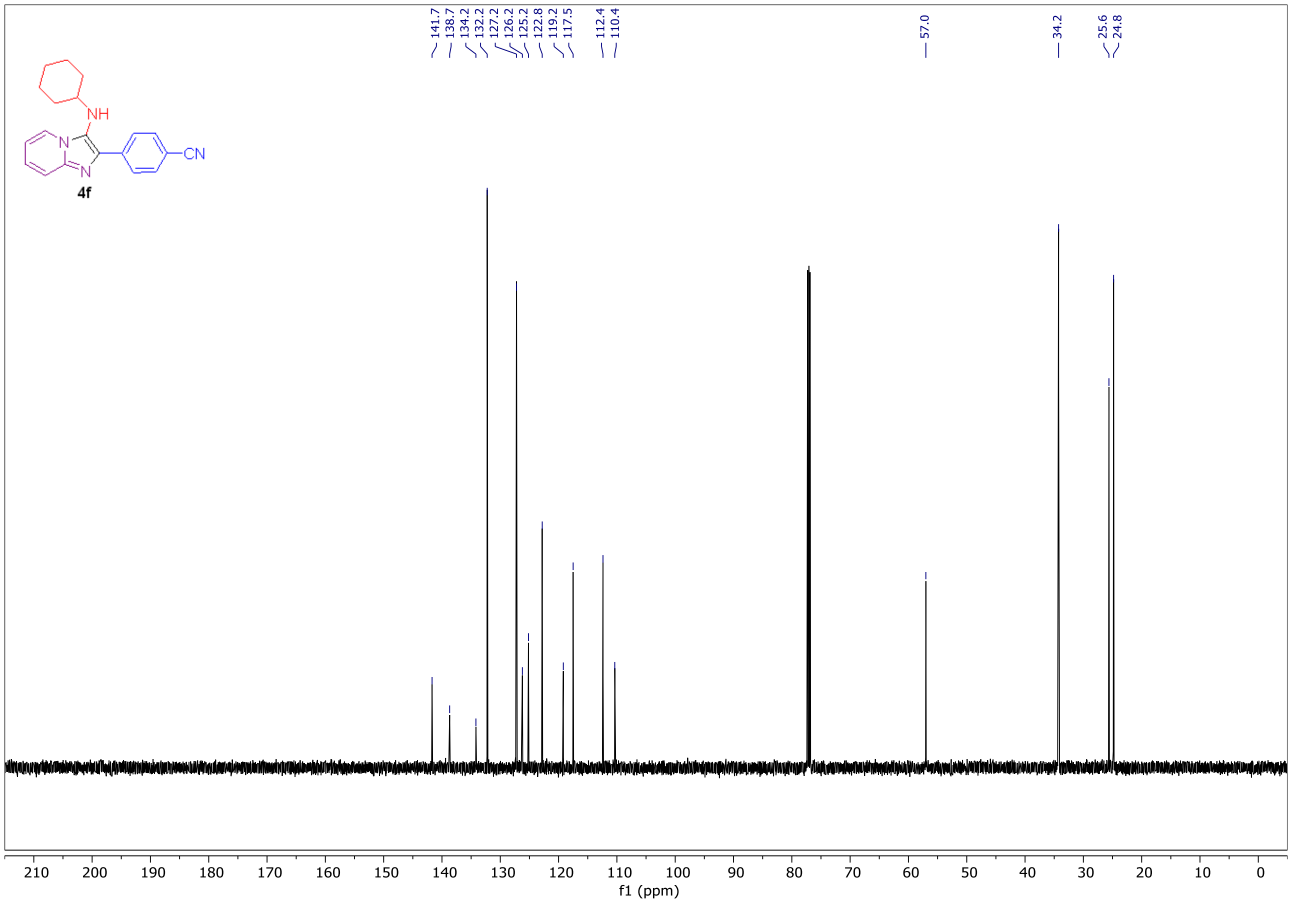

S 23. ^{13}C NMR spectrum (151 MHz, $CDCl_3$) of compound **4f**.

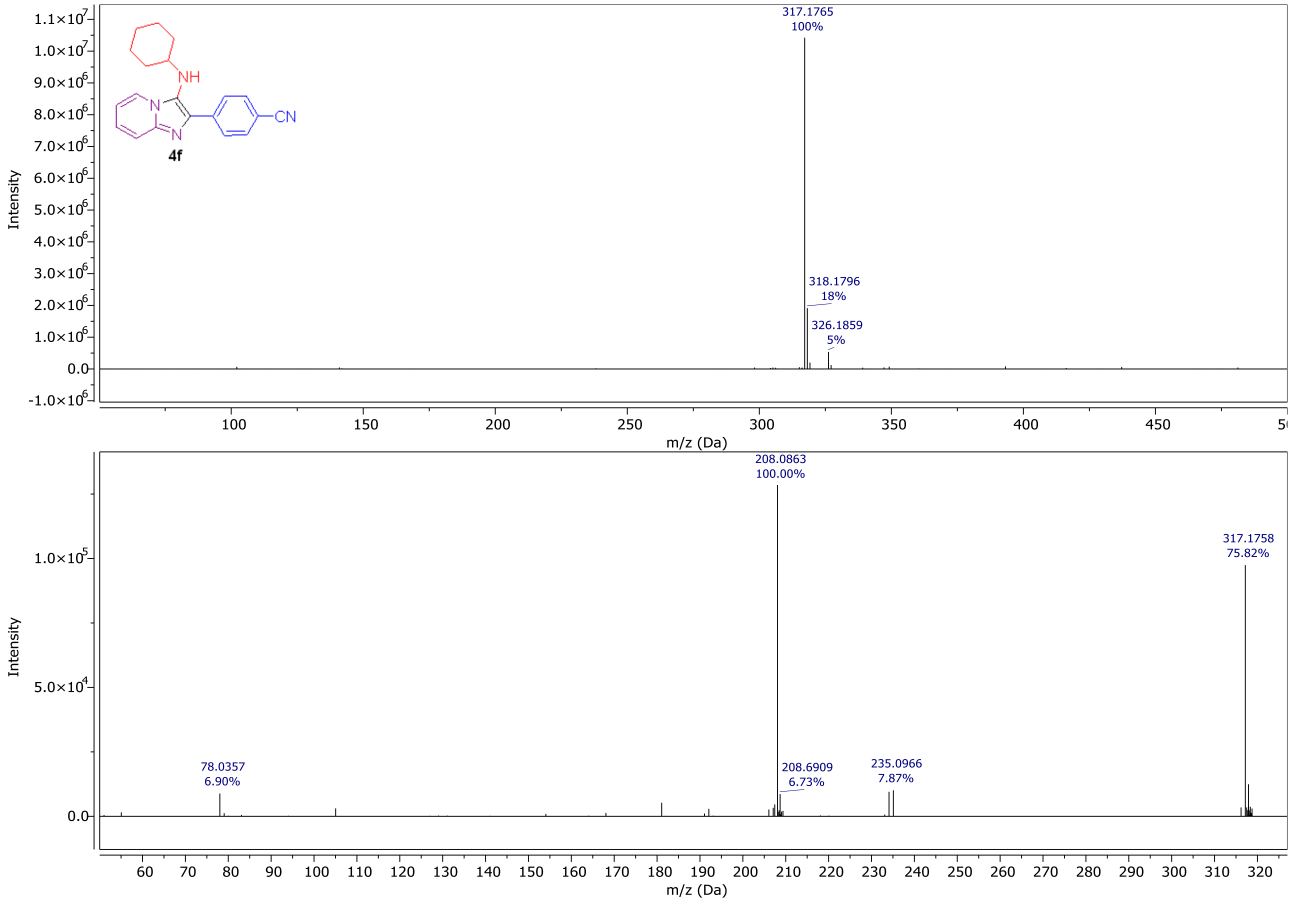

S 24. HRMS (ESI-QTOF) of compound **4f** and HRMS/MS for $[M+H]^+$.

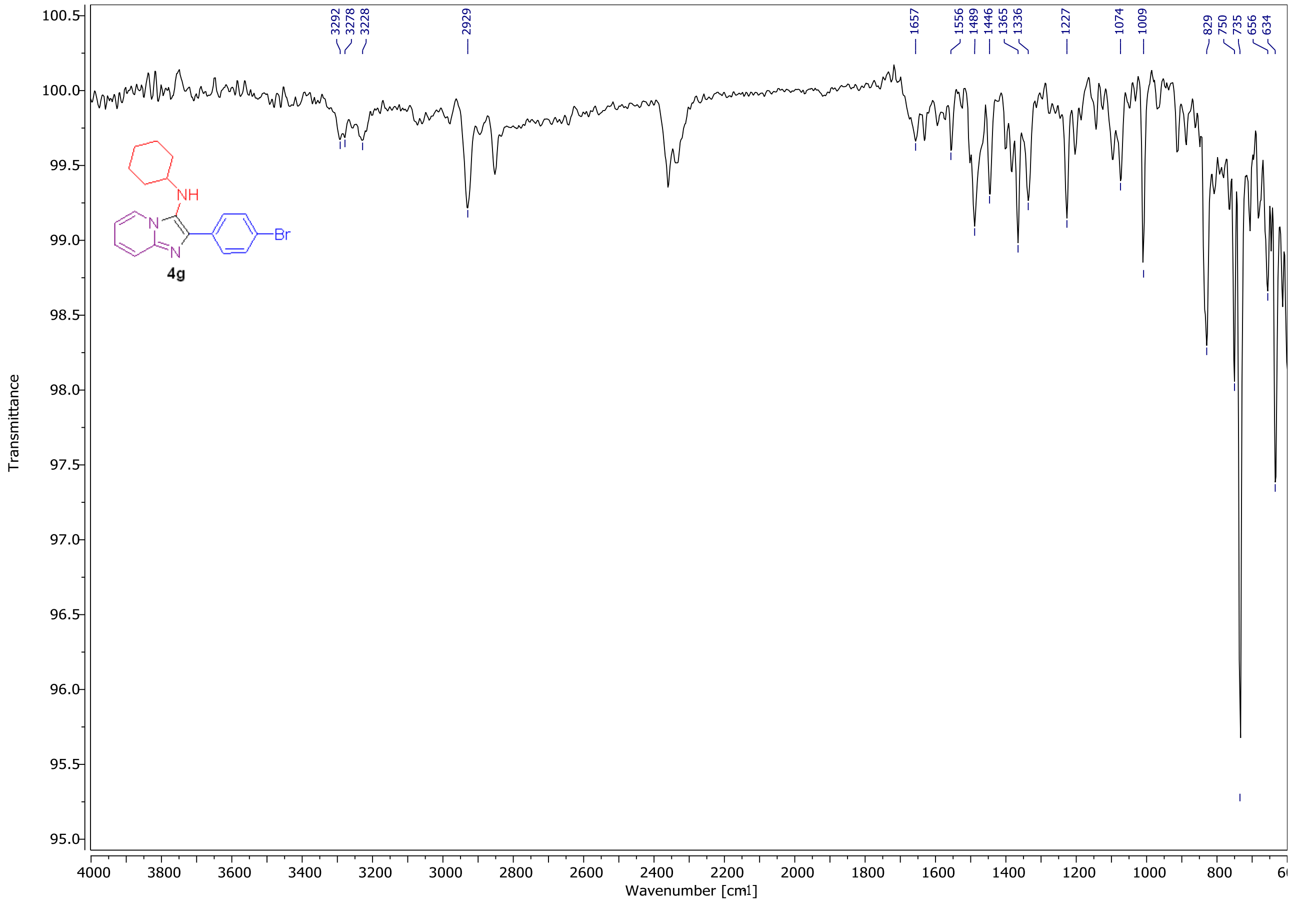

S 25. FT-IR (ATR) of compound **4g**.

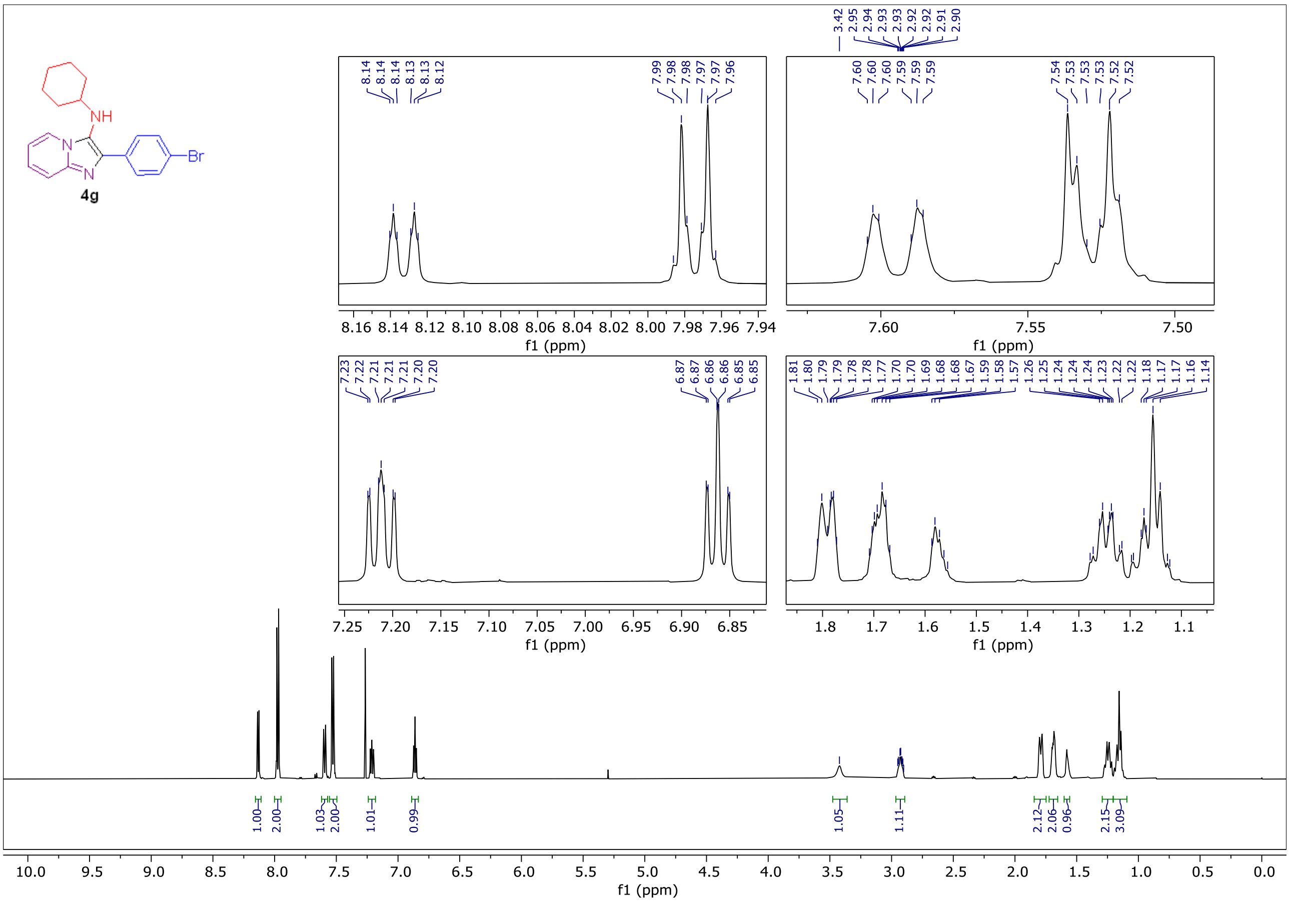

S 26. ^{1}H NMR spectrum (600 MHz, $CDCl_3$) of compound **4g**.

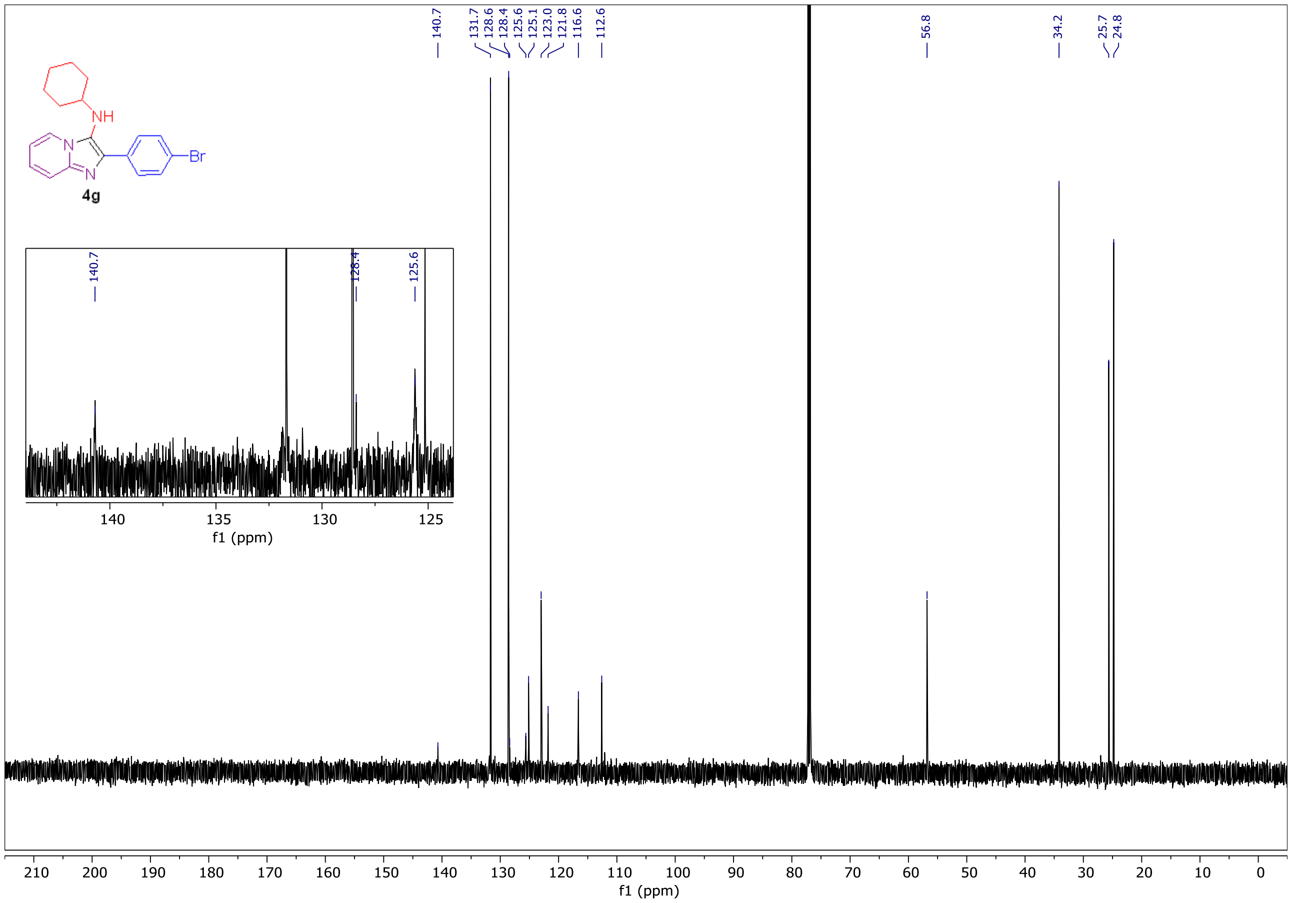

S 27. ^{13}C NMR spectrum (151 MHz, $CDCl_3$) of compound **4g**.

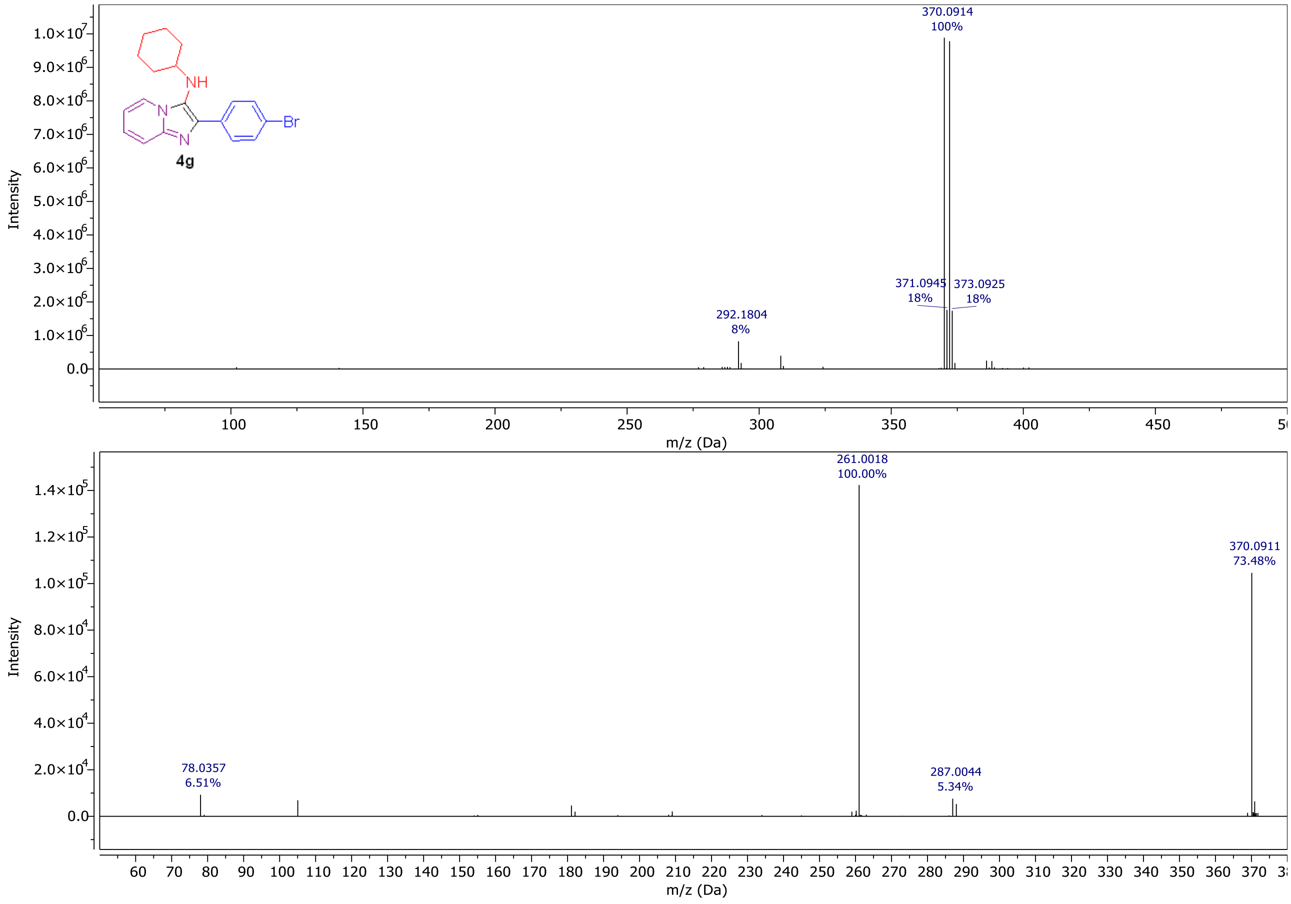

S 28. HRMS (ESI-QTOF) of compound **4g** and HRMS/MS for [M+H]$^+$.

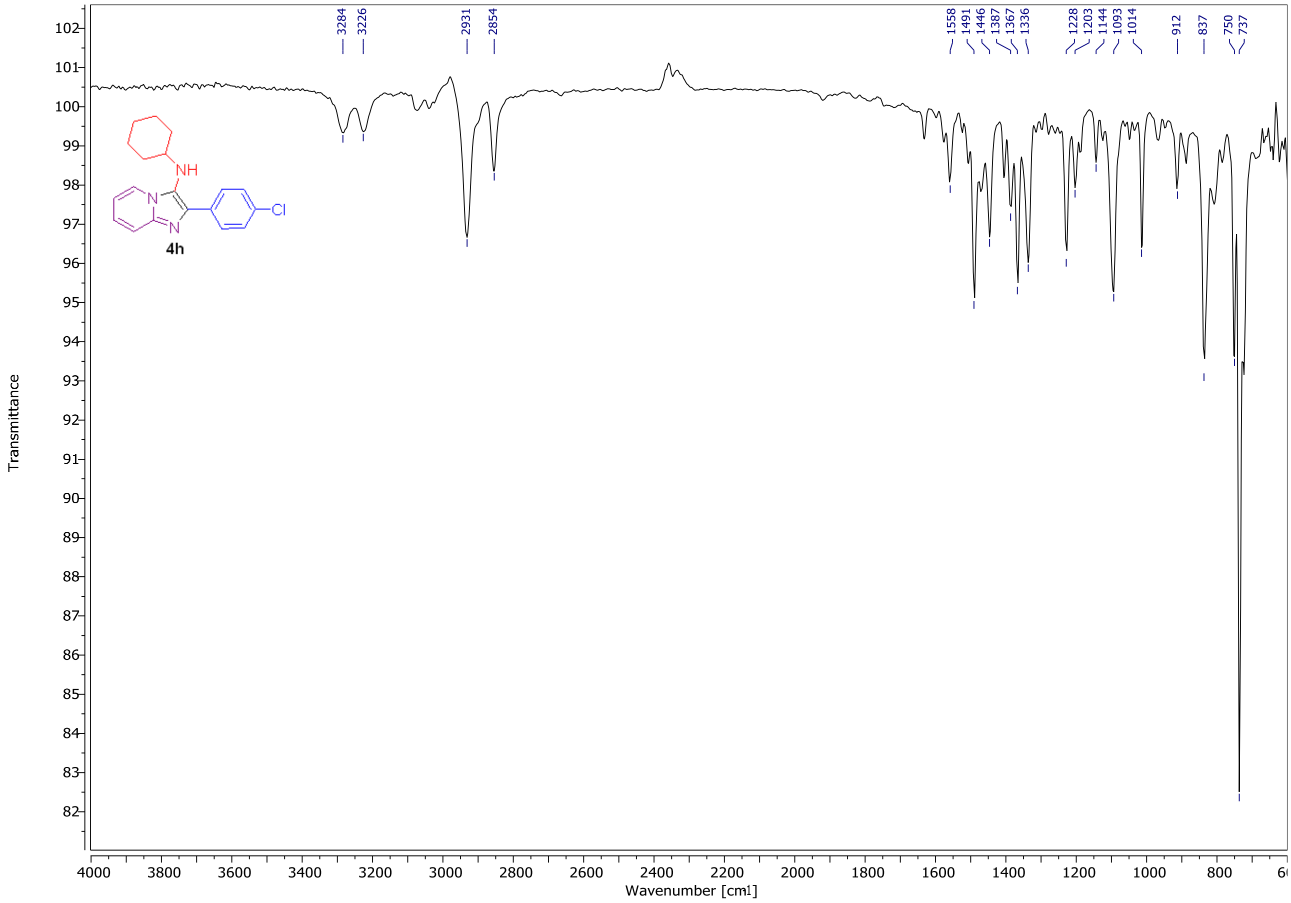

S 29. FT-IR (ATR) of compound **4h**.

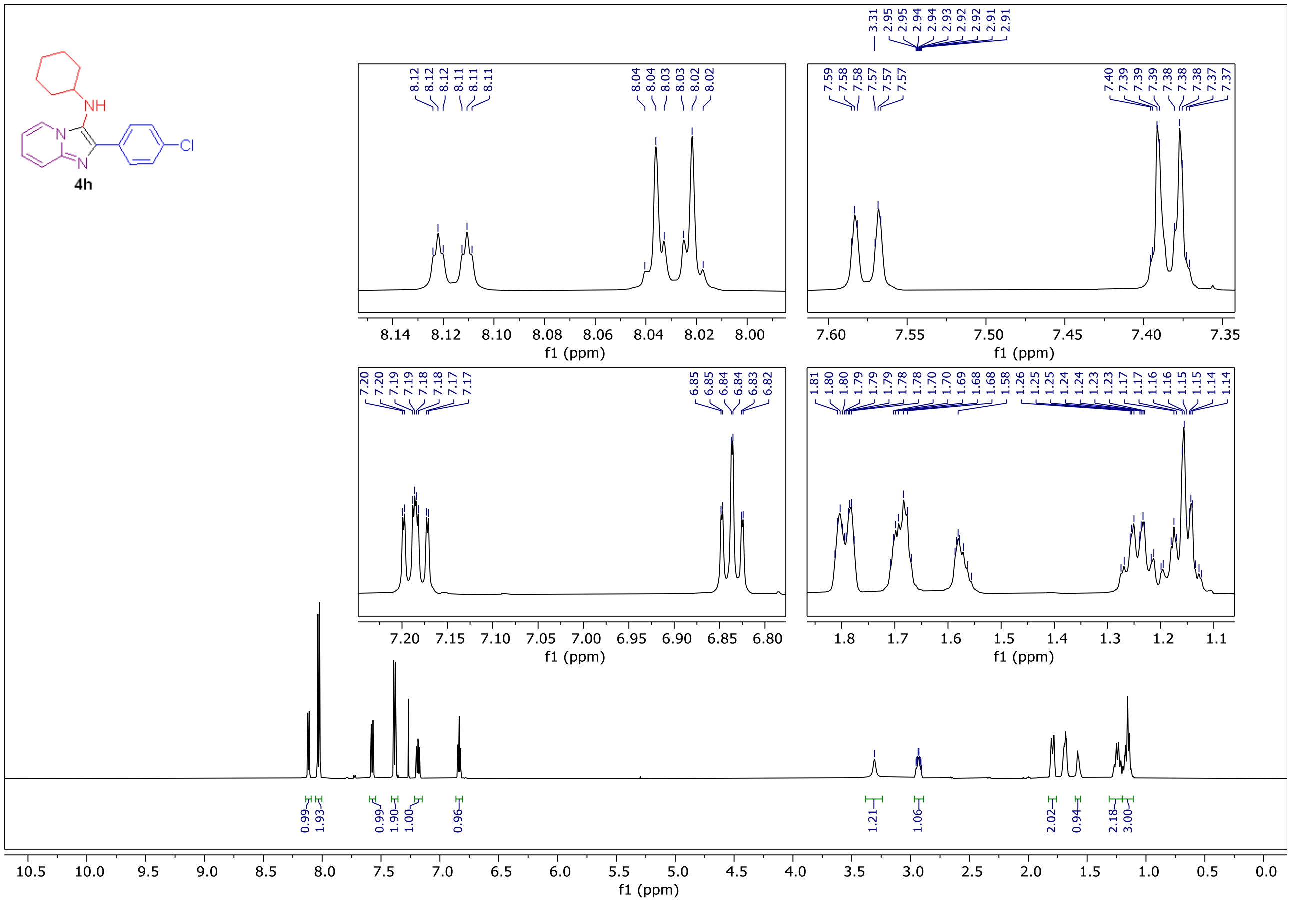

S 30. ^1H NMR spectrum (600 MHz, $CDCl_3$) of compound **4h**.

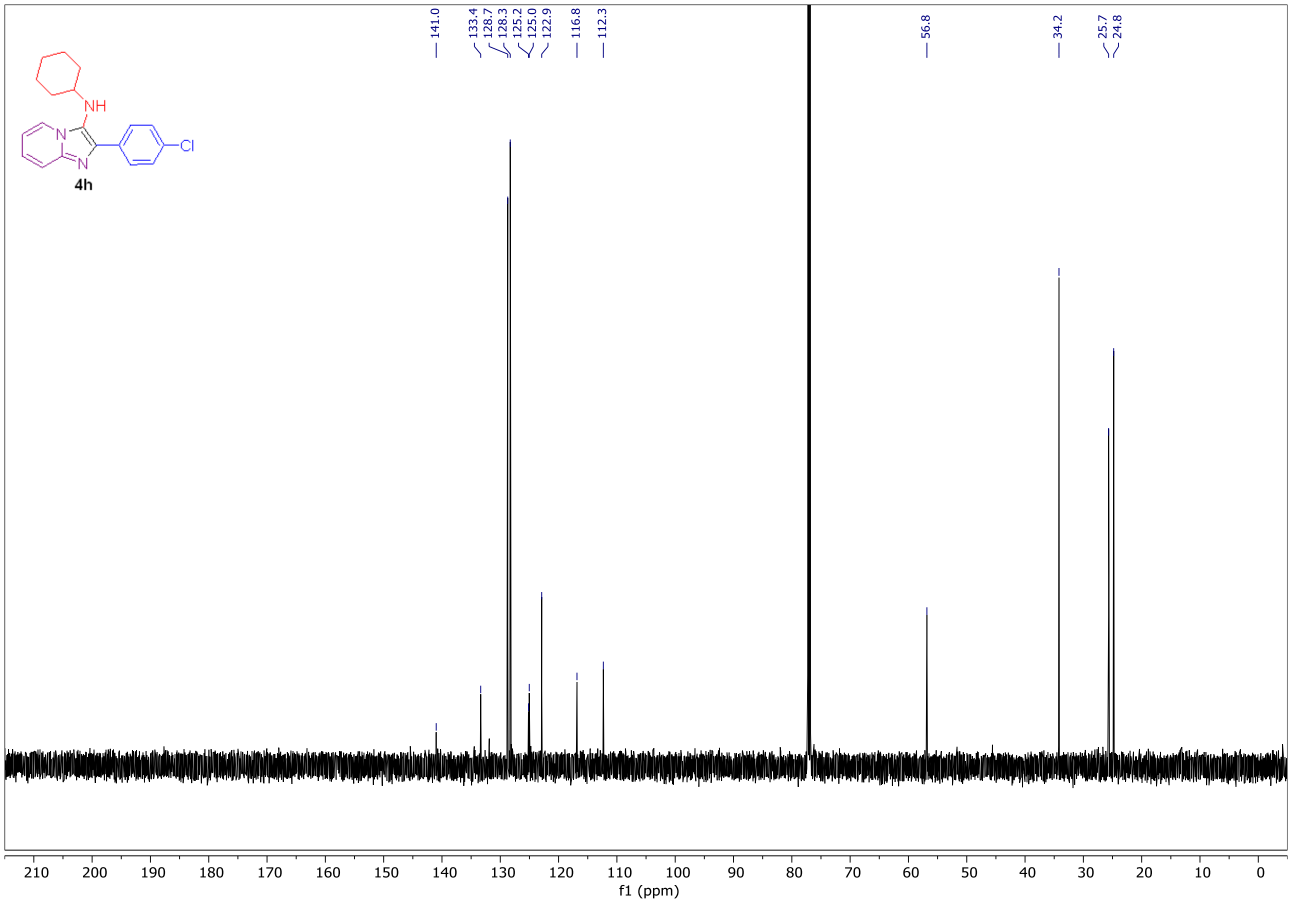

S 31. ^{13}C NMR spectrum (151 MHz, $CDCl_3$) of compound **4h**.

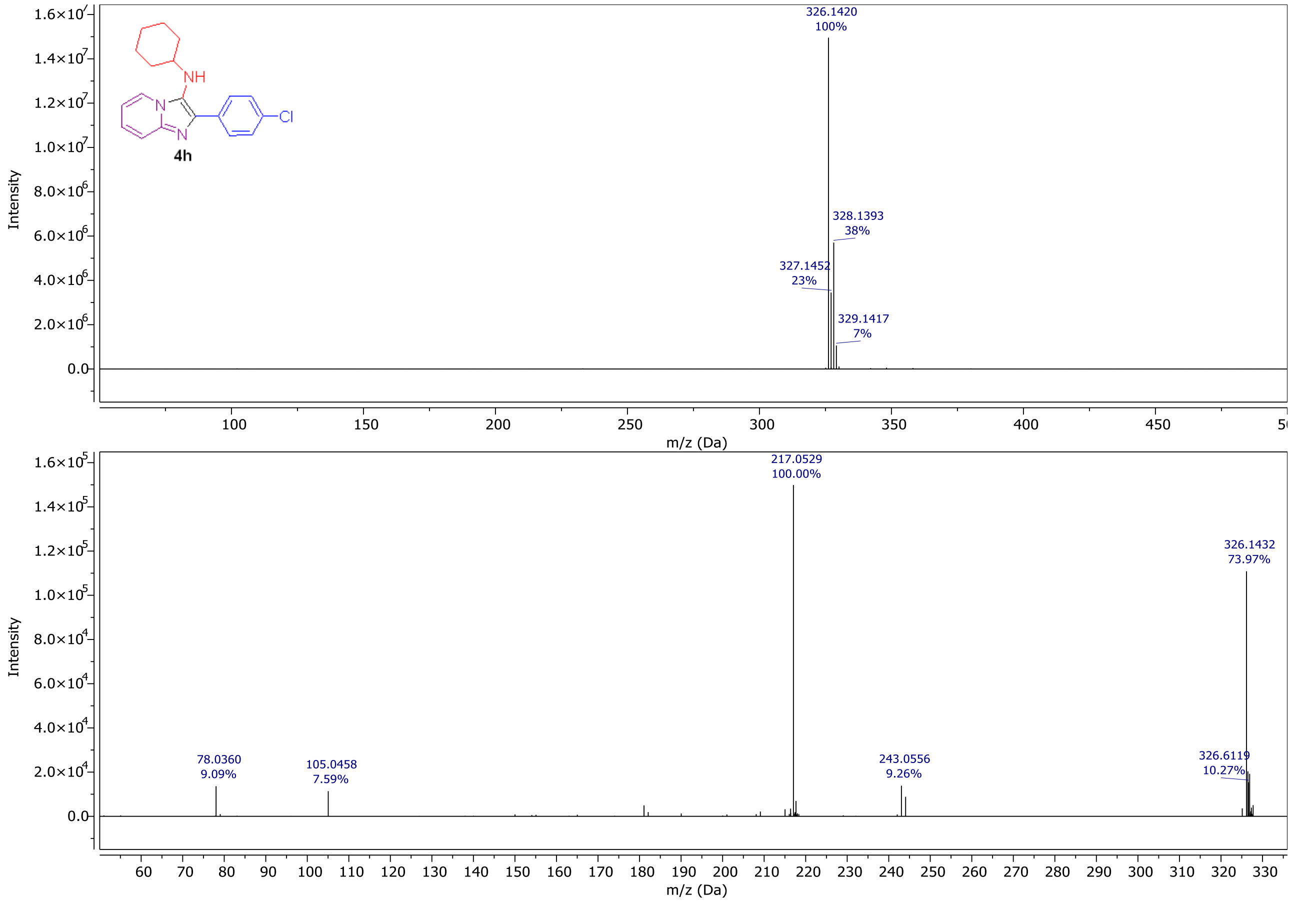

S 32. HRMS (ESI-QTOF) of compound **4h** and HRMS/MS for $[M+H]^+$.

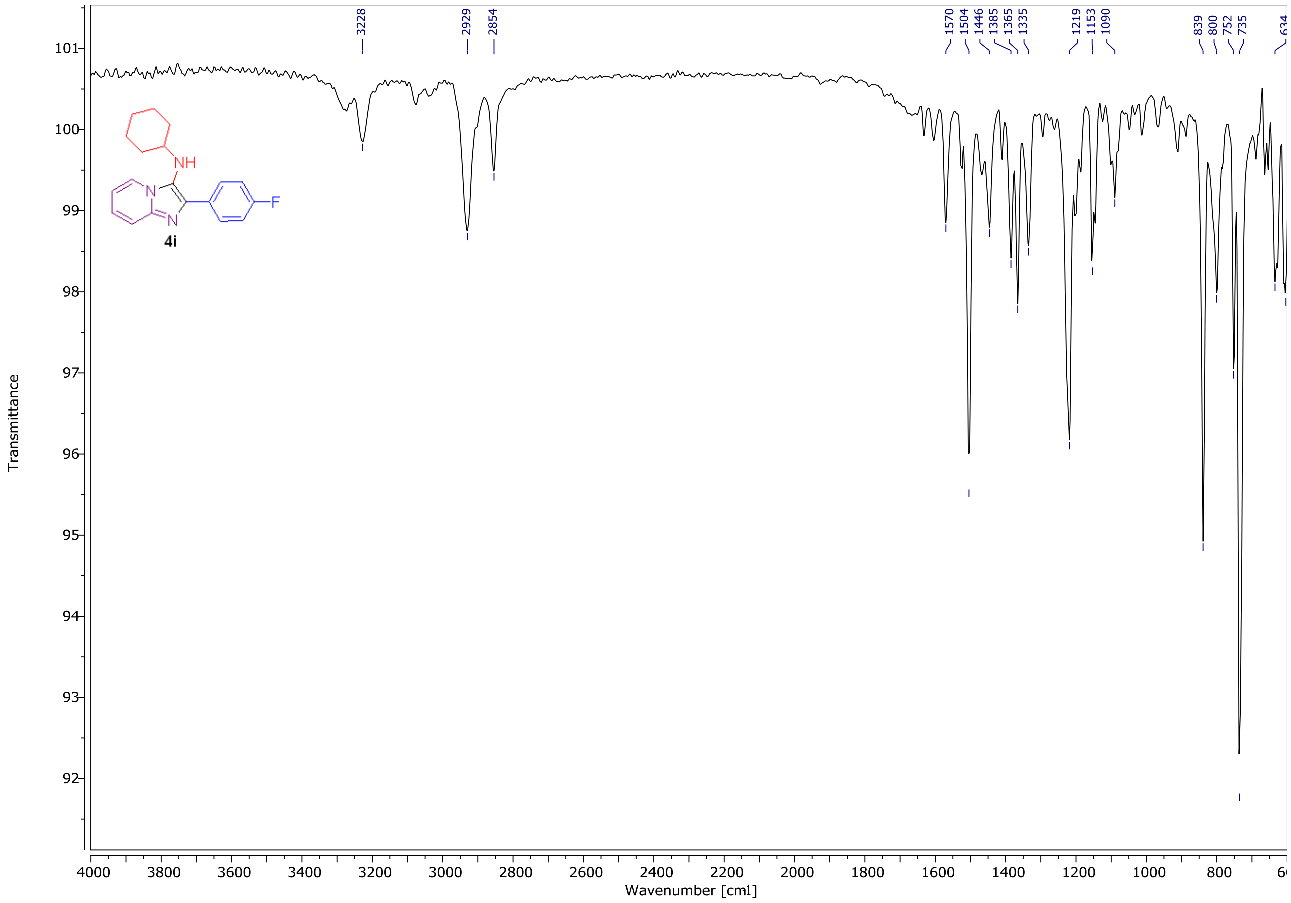

S 33. FT-IR (ATR) of compound **4i**.

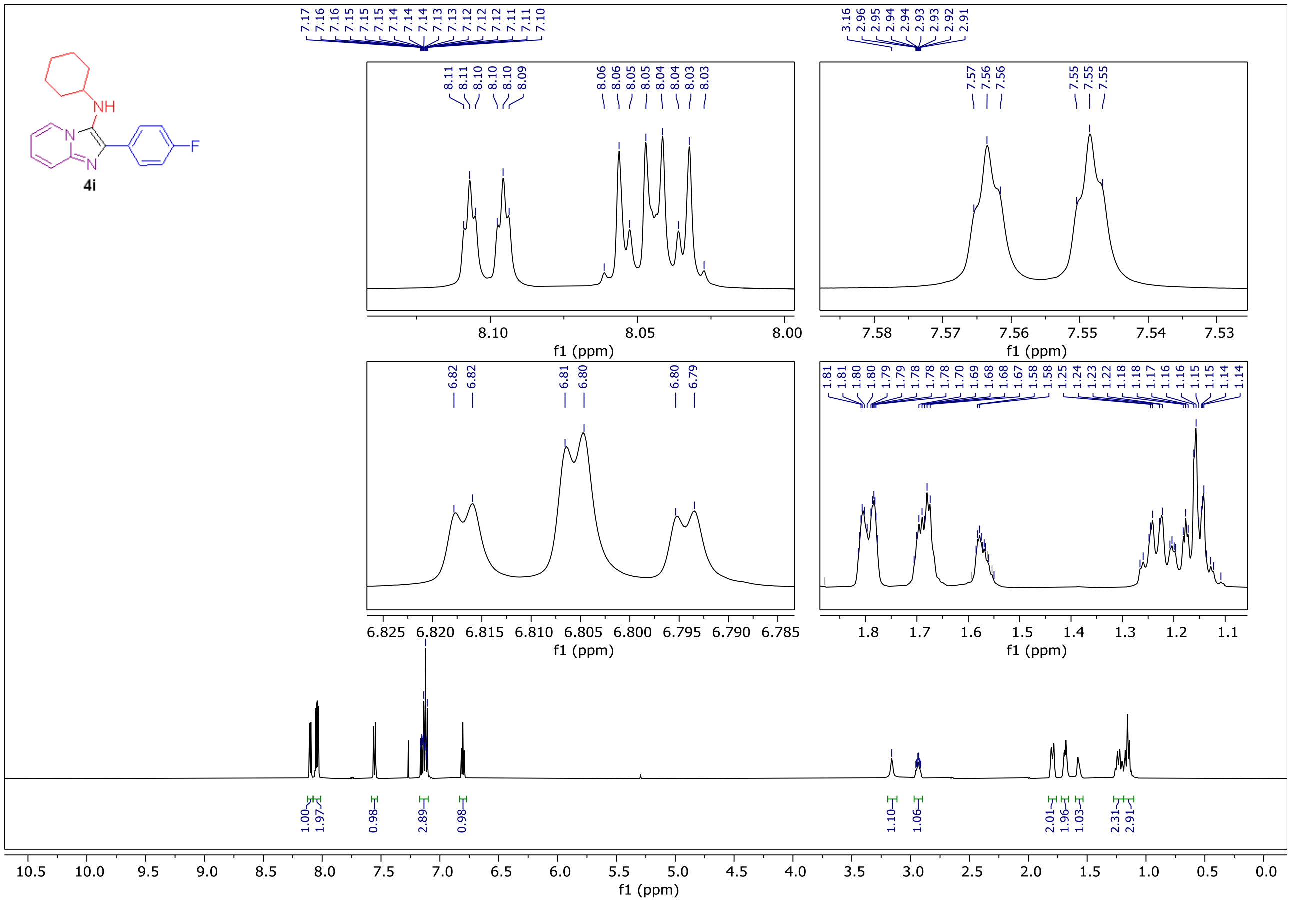

S 34. ^{1}H NMR spectrum (600 MHz, $CDCl_3$) of compound **4i**.

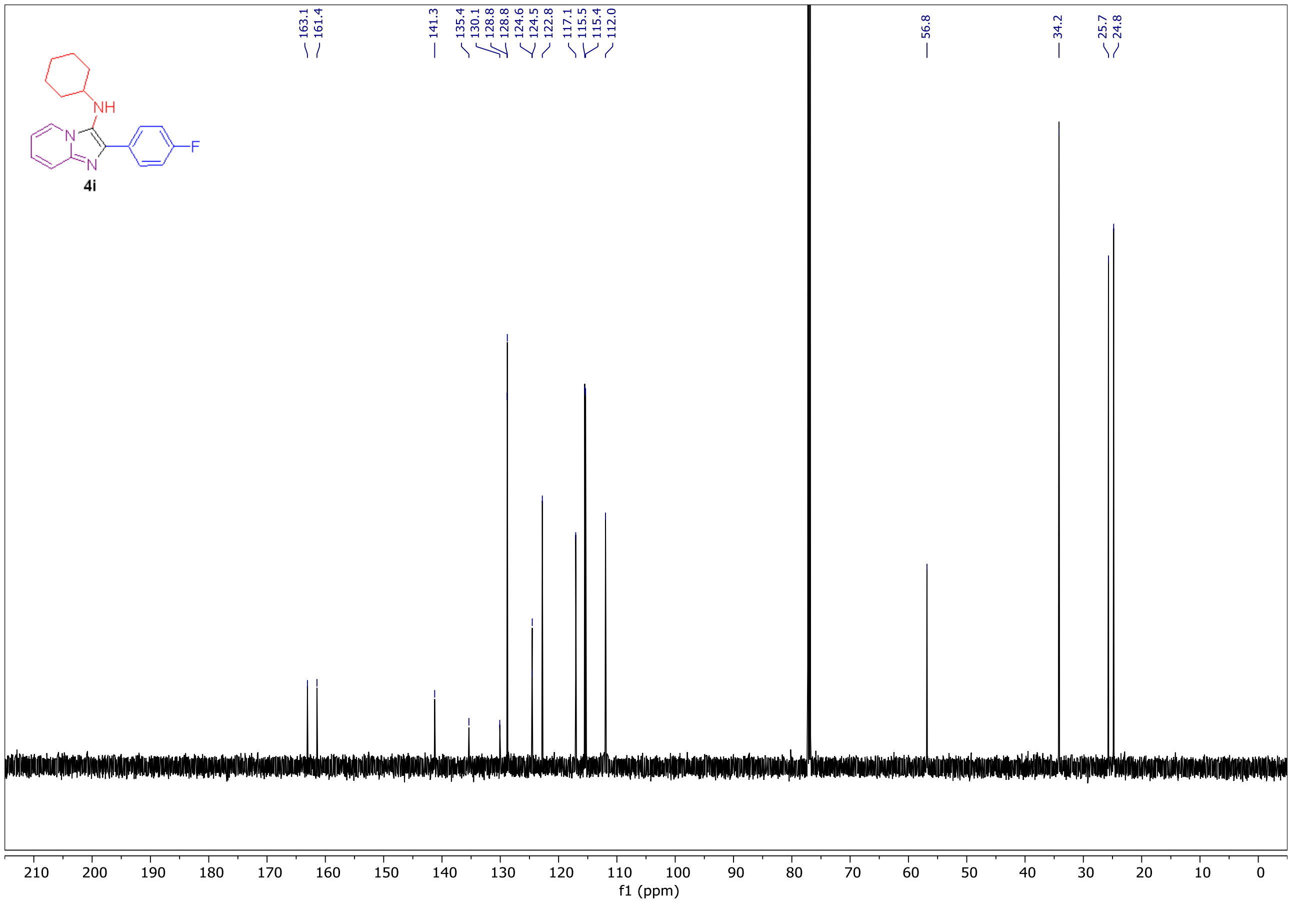

S 35. ^{13}C NMR spectrum (151 MHz, $CDCl_3$) of compound **4i**.

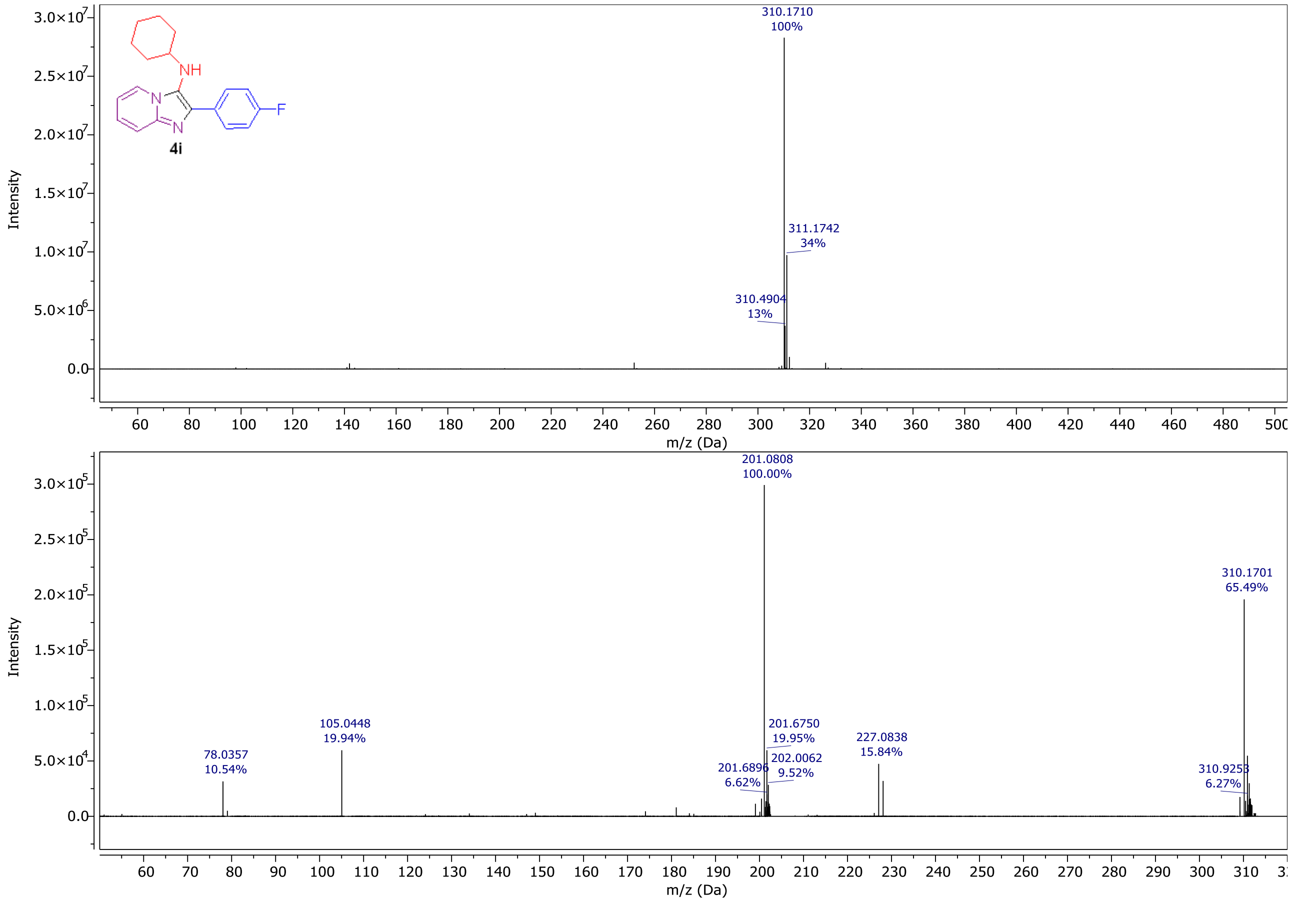

S 36. HRMS (ESI-QTOF) of compound **4i** and HRMS/MS for $[M+H]^+$.

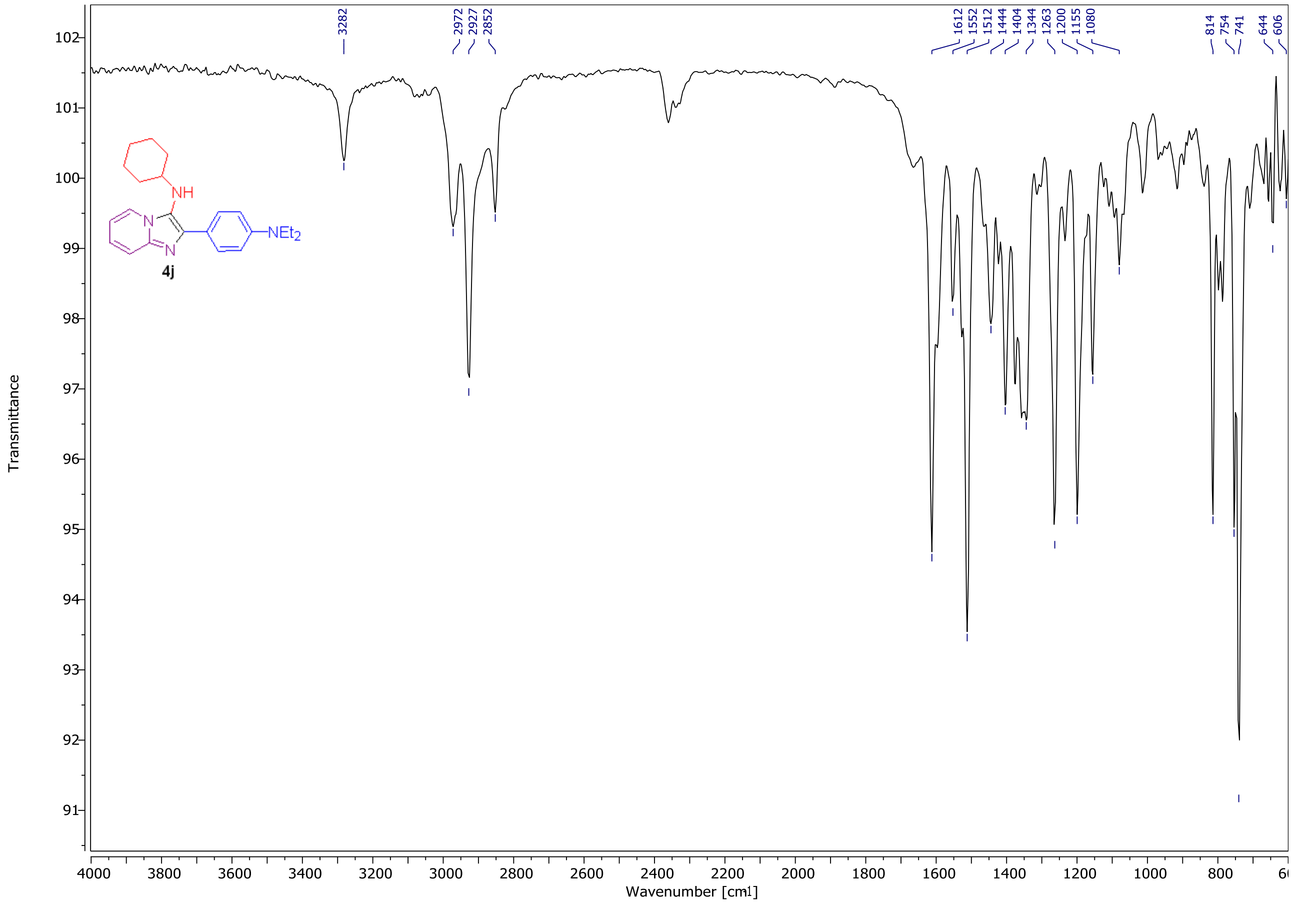

S 37. FT-IR (ATR) of compound **4j**.

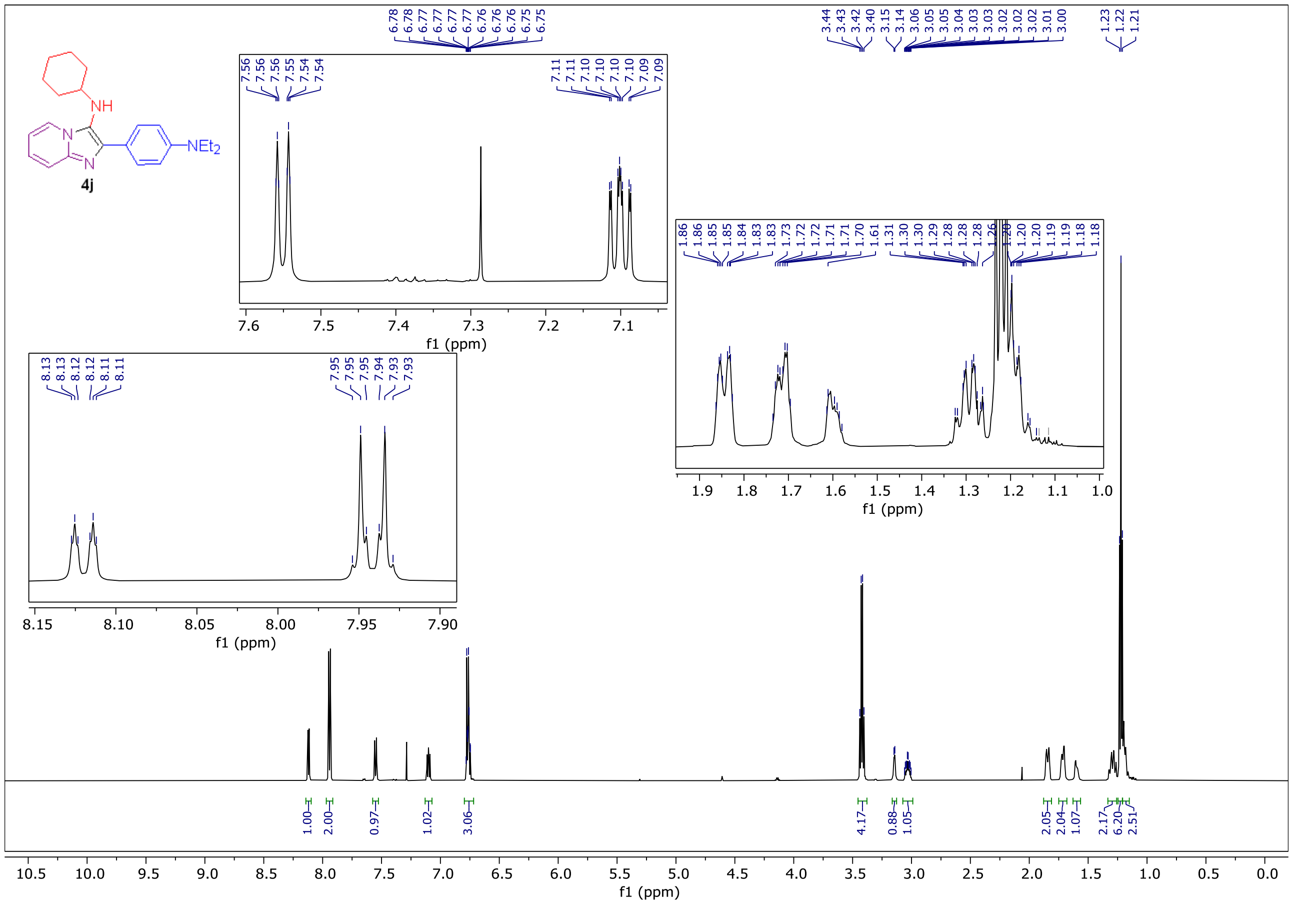

S 38. ^{1}H NMR spectrum (600 MHz, $CDCl_3$) of compound **4j**.

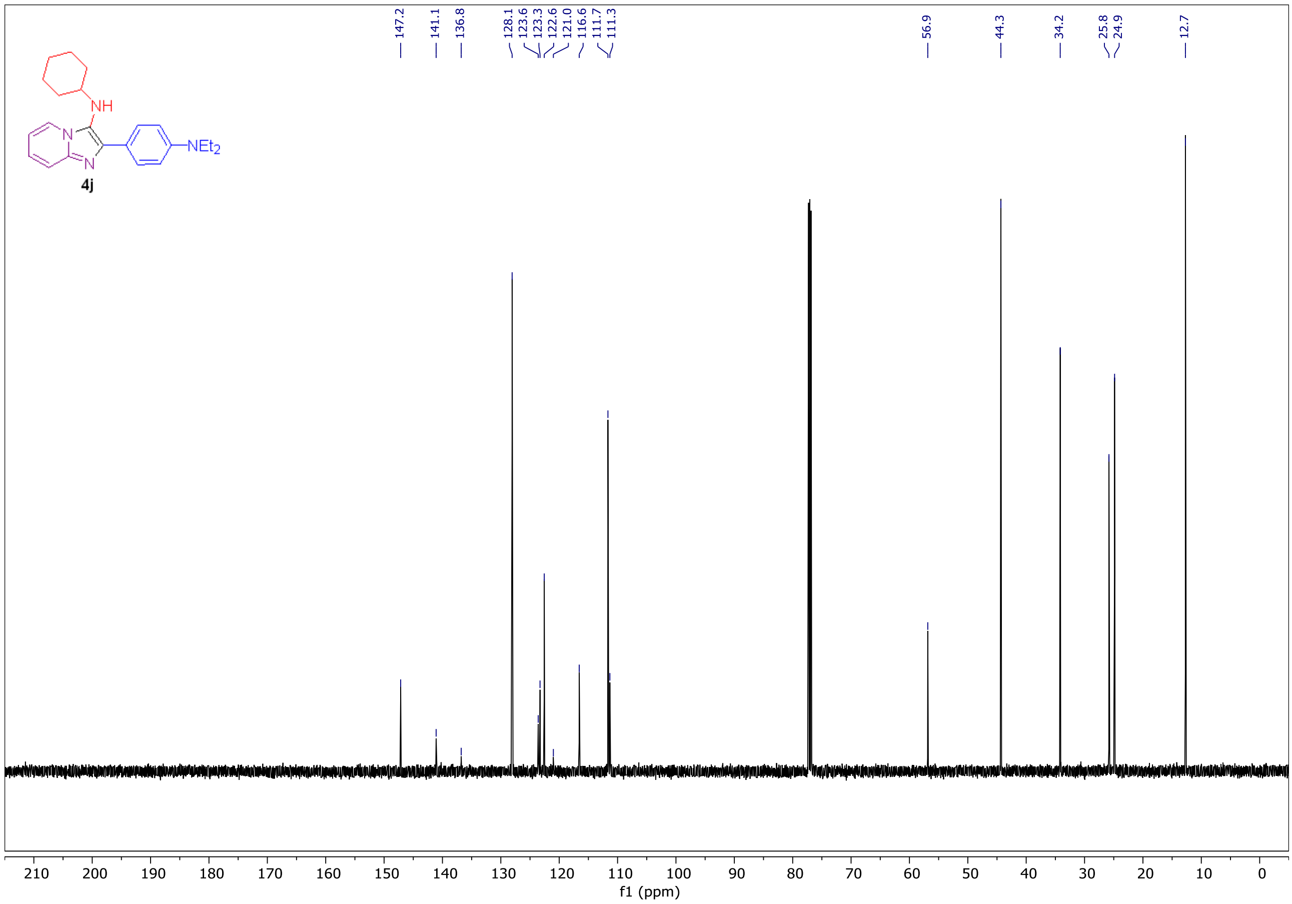

S 39. ^{13}C NMR spectrum (151 MHz, $CDCl_3$) of compound **4j**.

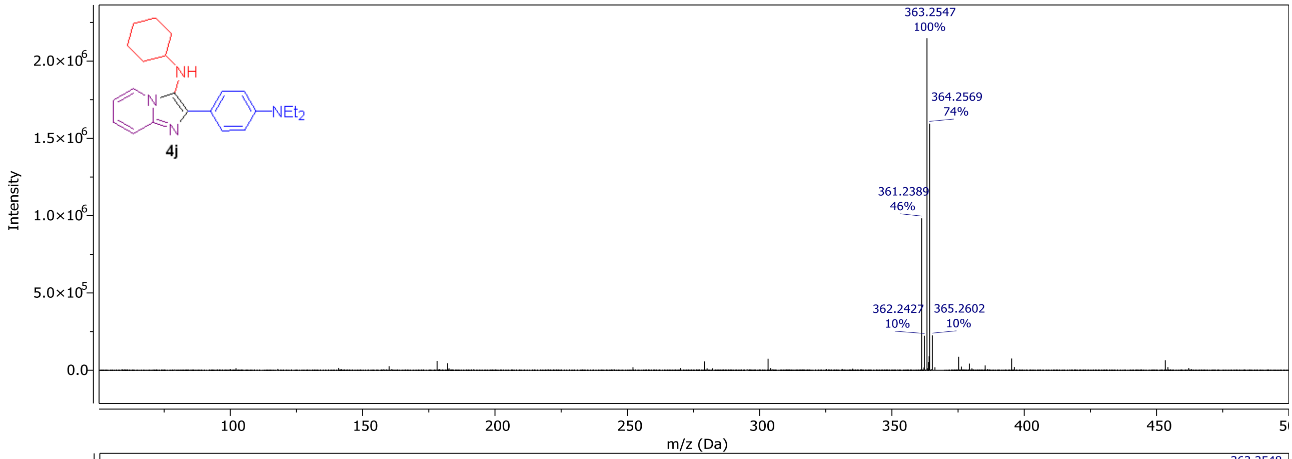

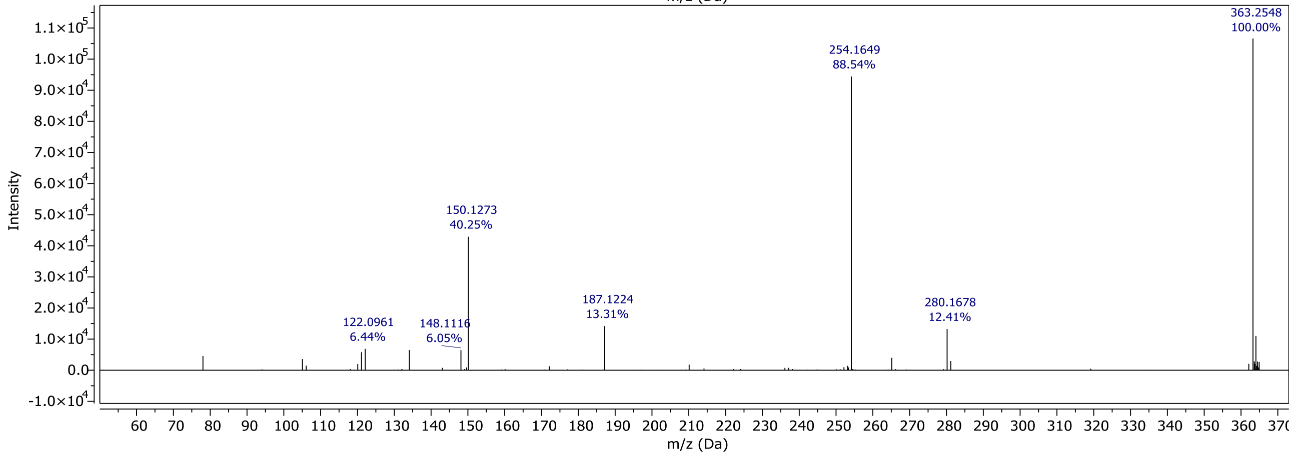

S 40. HRMS (ESI-QTOF) of compound **4j** and HRMS/MS for $[M+H]^+$.

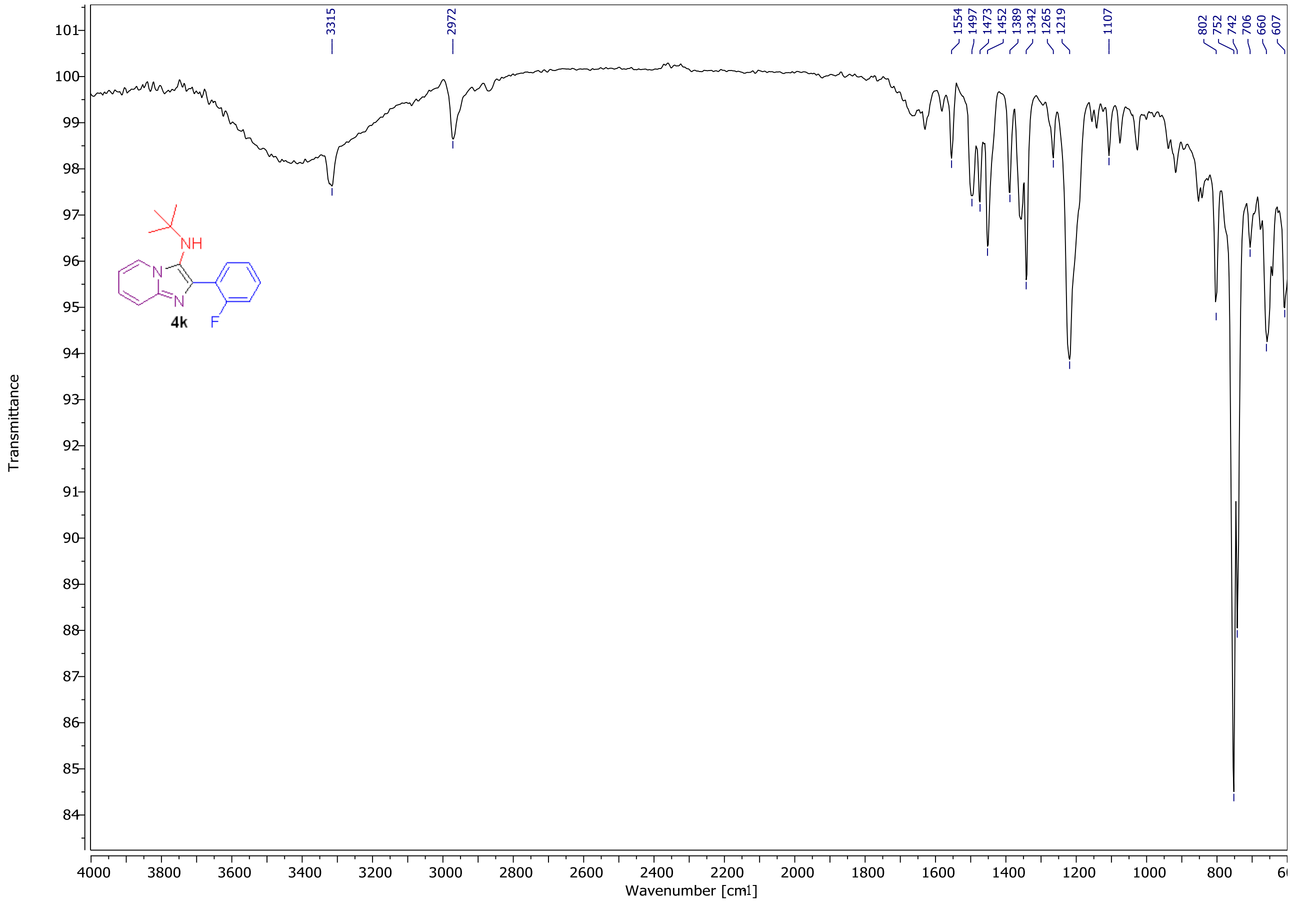

S 41. FT-IR (ATR) of compound **4k**.

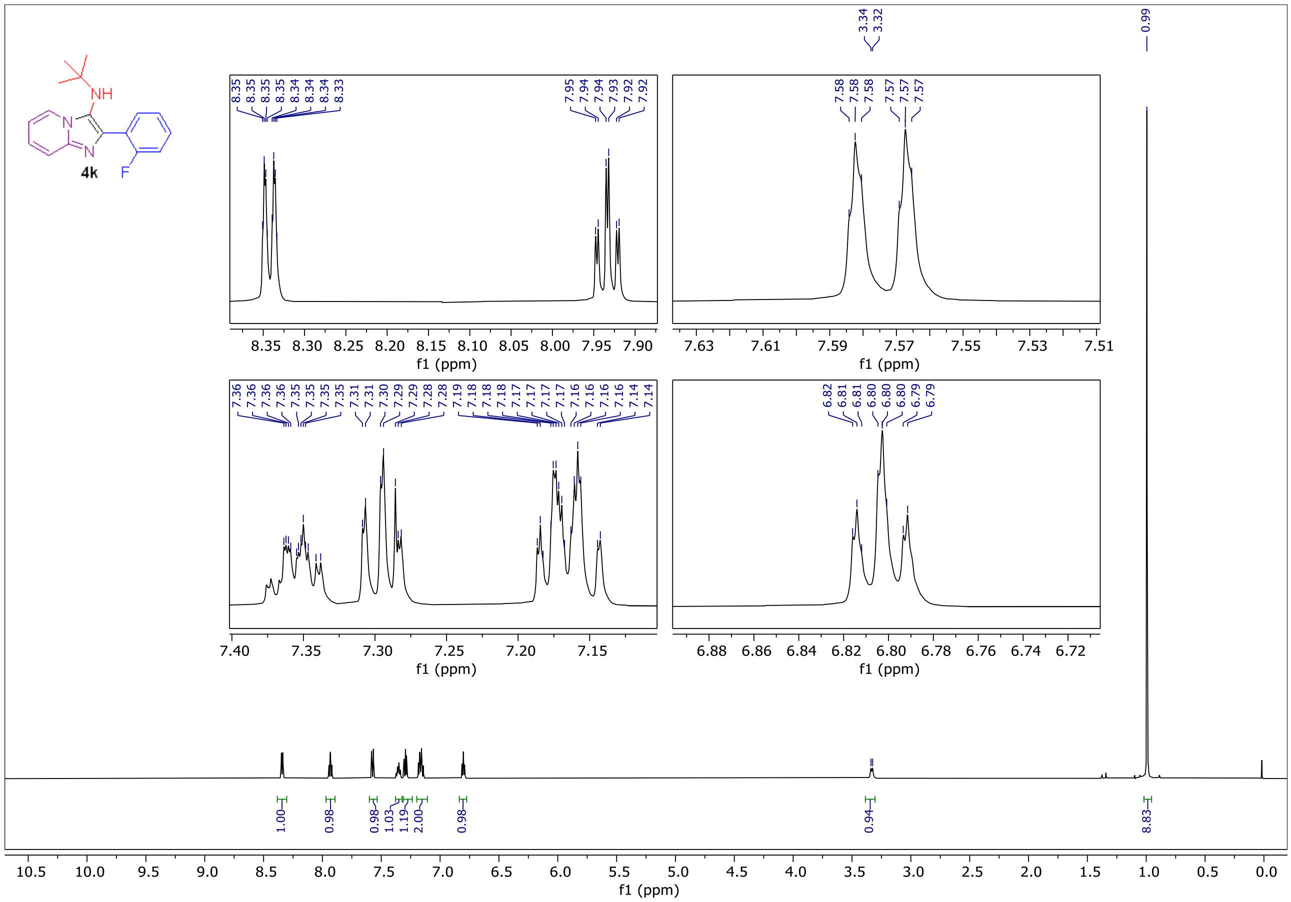

S 42. ^{1}H NMR spectrum (600 MHz, $CDCl_3$) of compound **4k**.

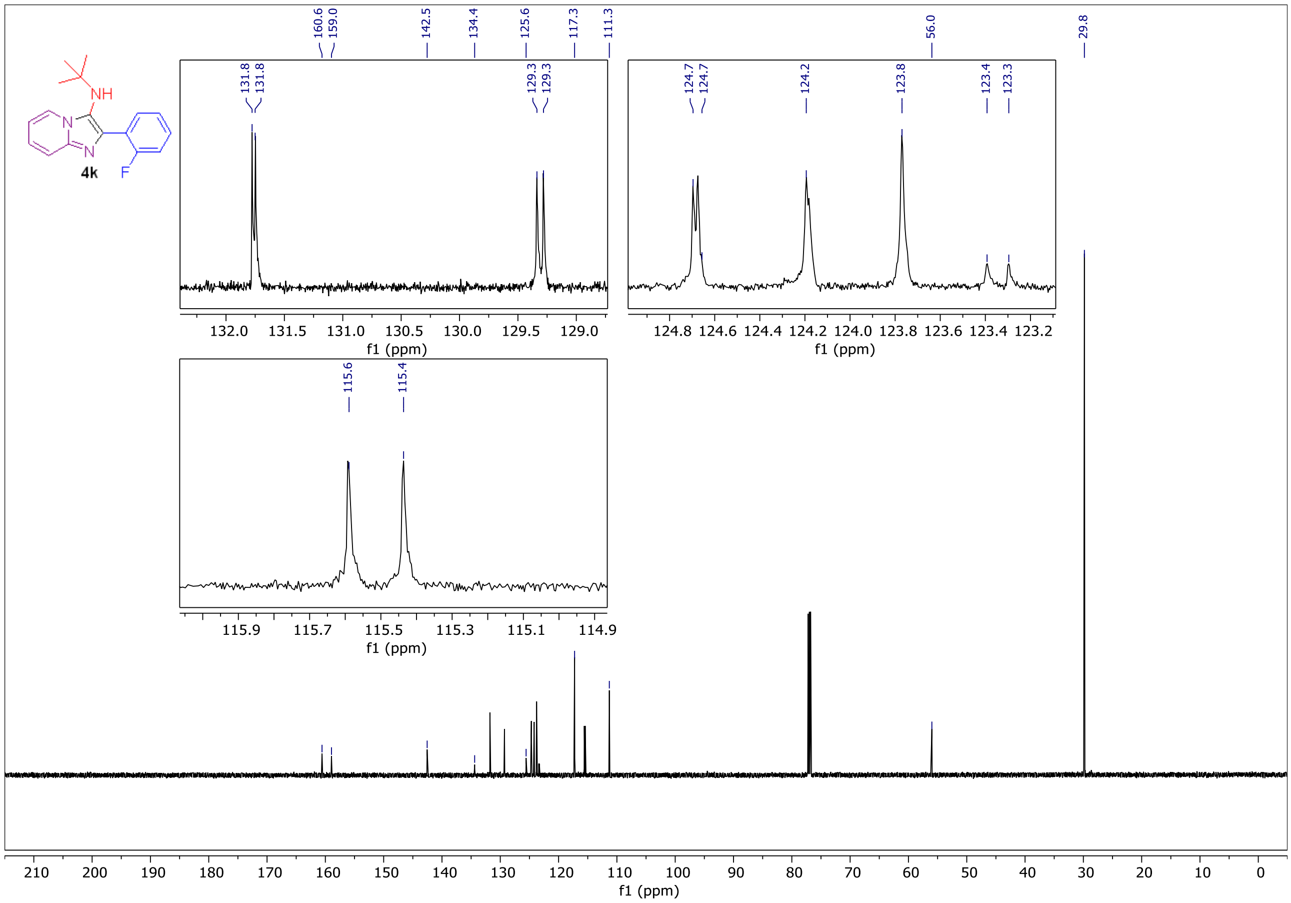

S 43. ^{13}C NMR spectrum (151 MHz, $CDCl_3$) of compound **4k**.

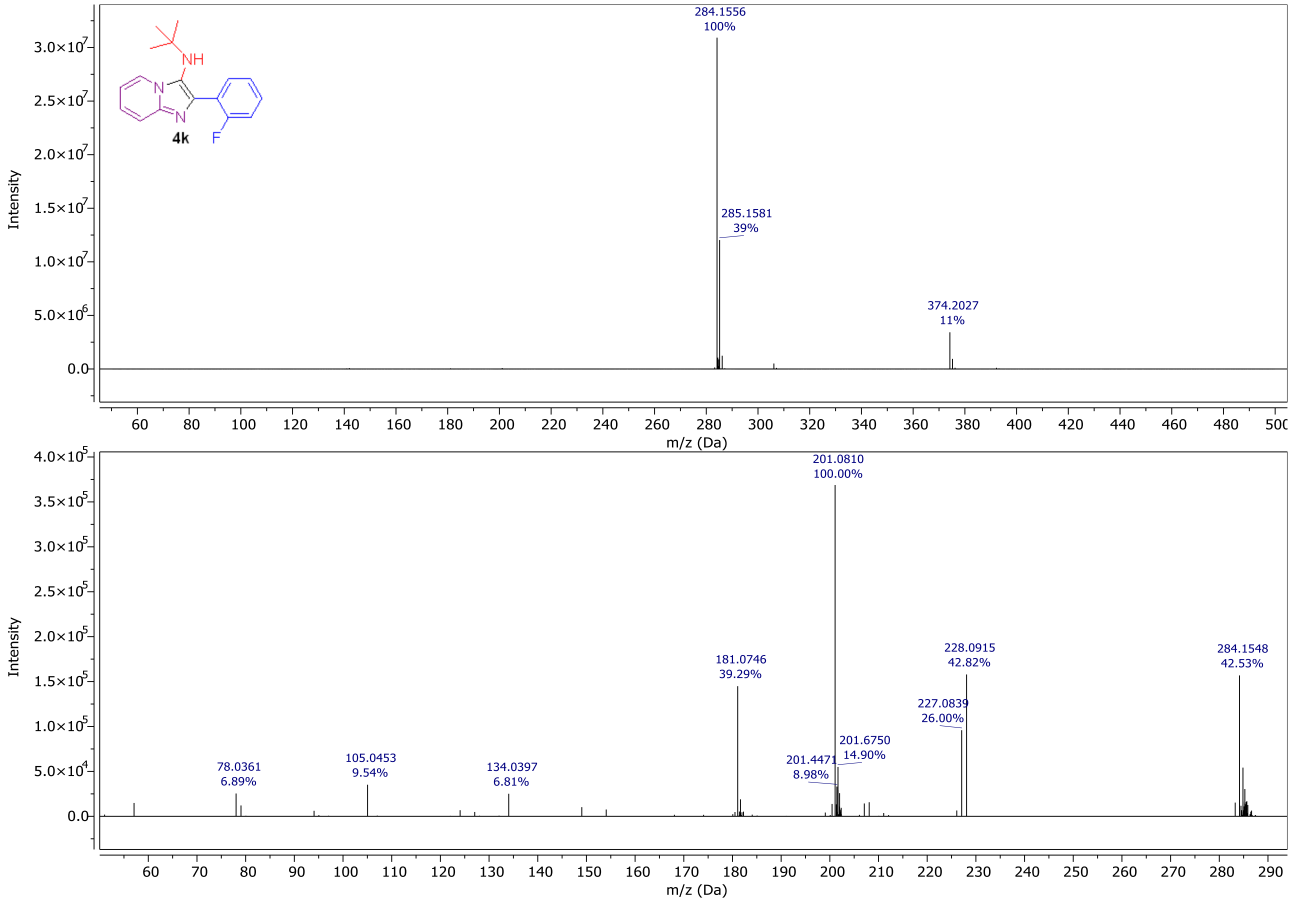

S 44. HRMS (ESI-QTOF) of compound **4k** and HRMS/MS for $[M+H]^+$.

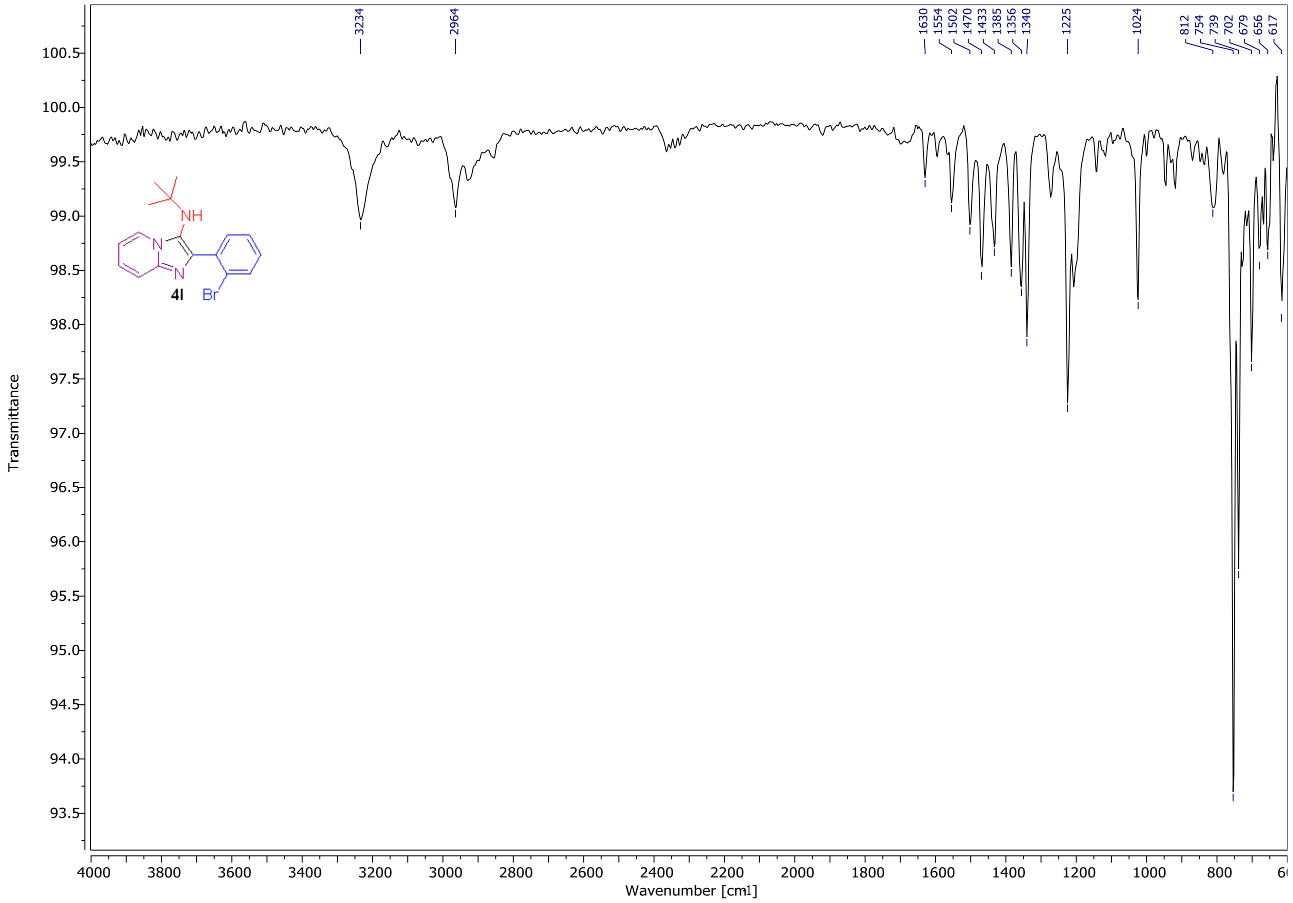

S 45. FT-IR (ATR) of compound **4l**.

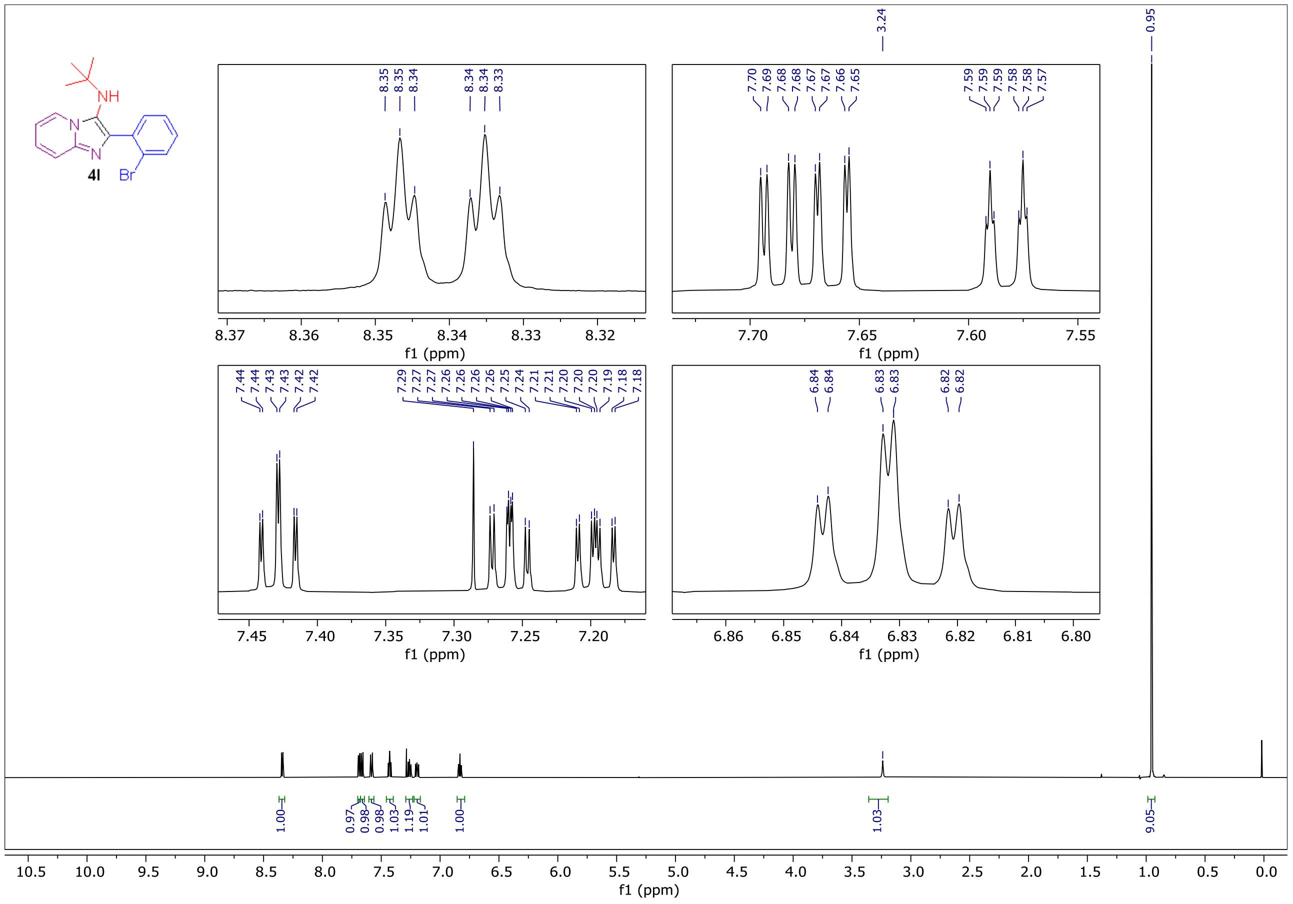

S 46. ^{1}H NMR spectrum (600 MHz, $CDCl_3$) of compound **4l**.

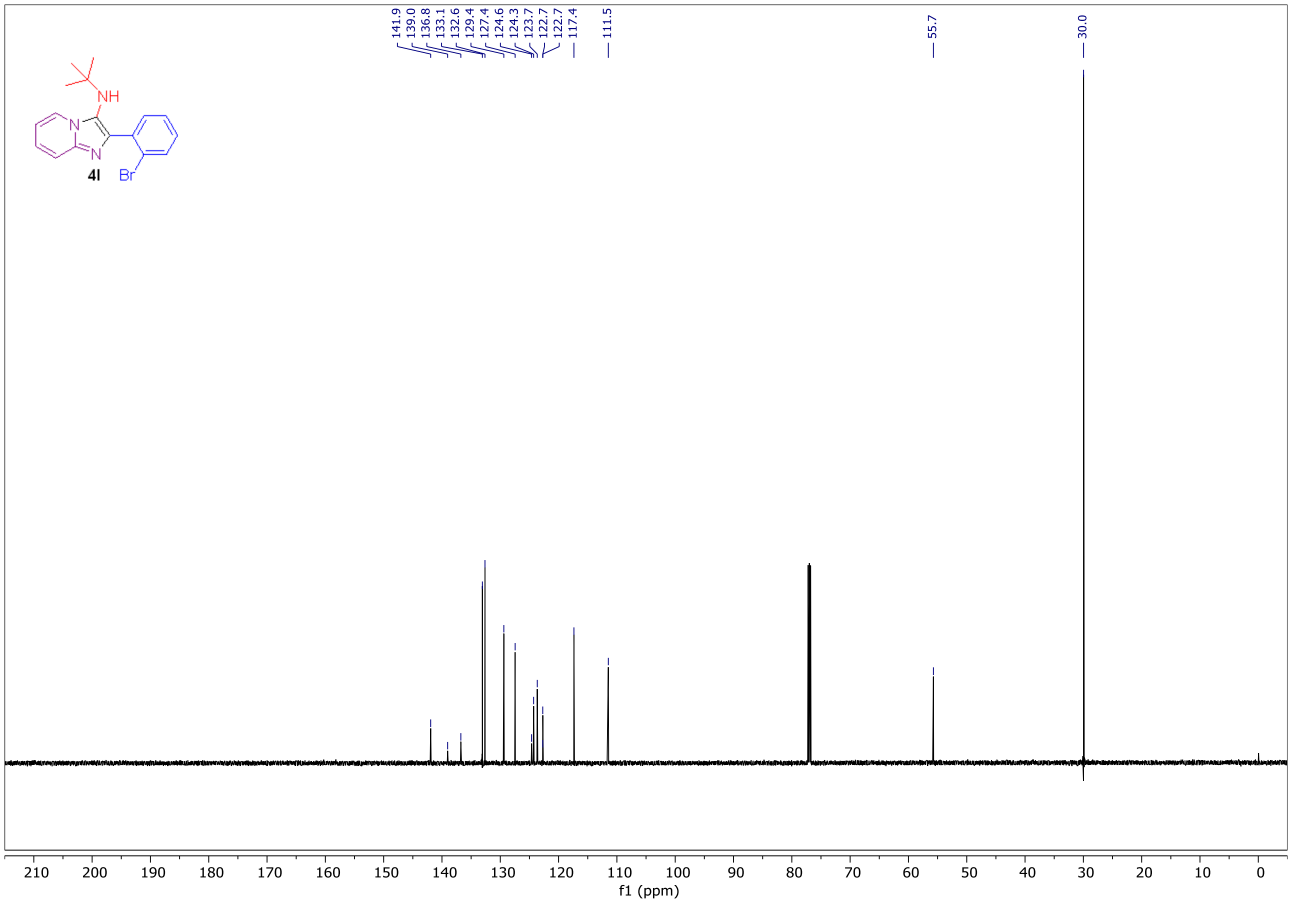

S 47. ^{13}C NMR spectrum (151 MHz, $CDCl_3$) of compound **4l**.

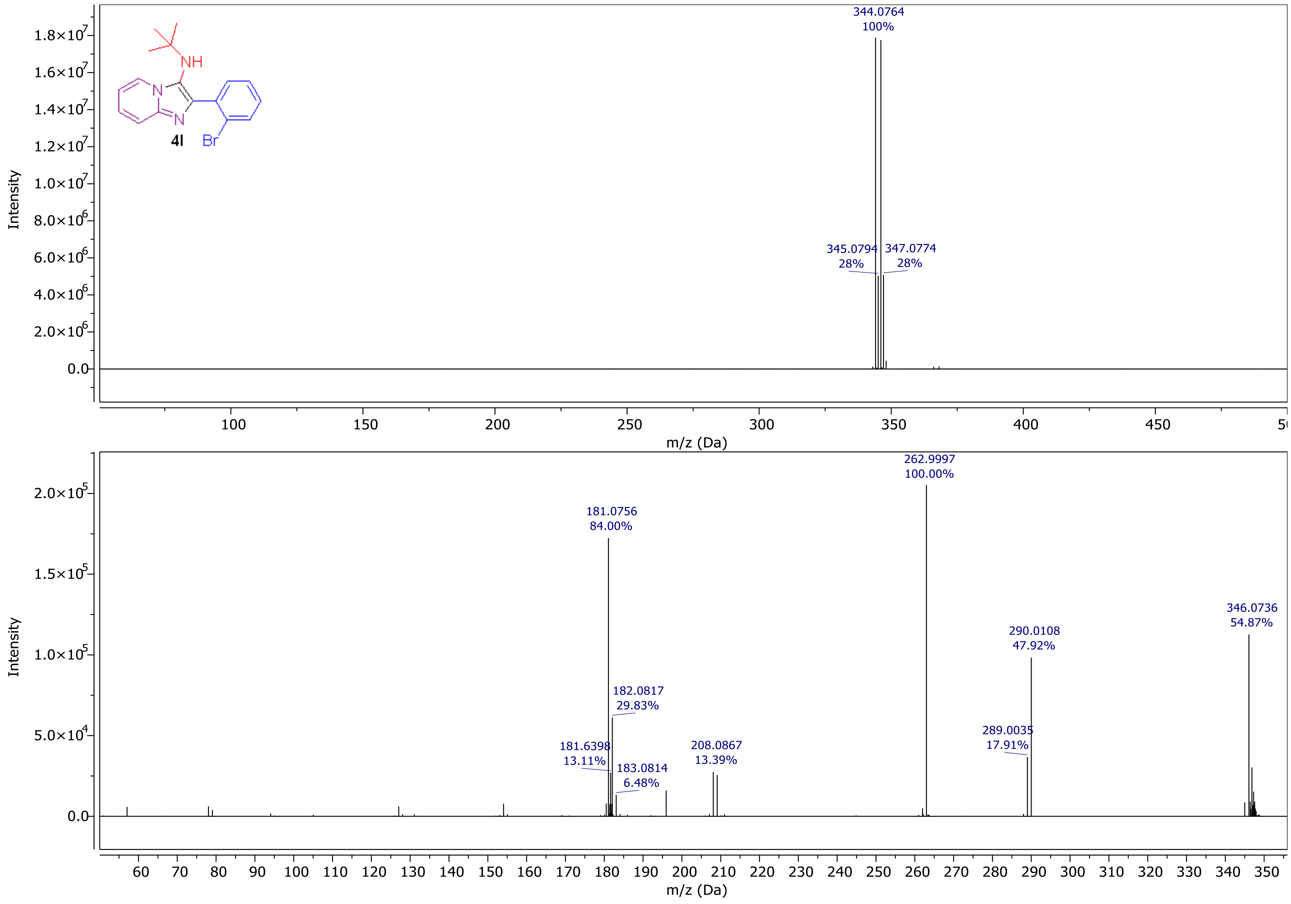

S 48. HRMS (ESI-QTOF) of compound **4l** and HRMS/MS for $[M+H]^+$.

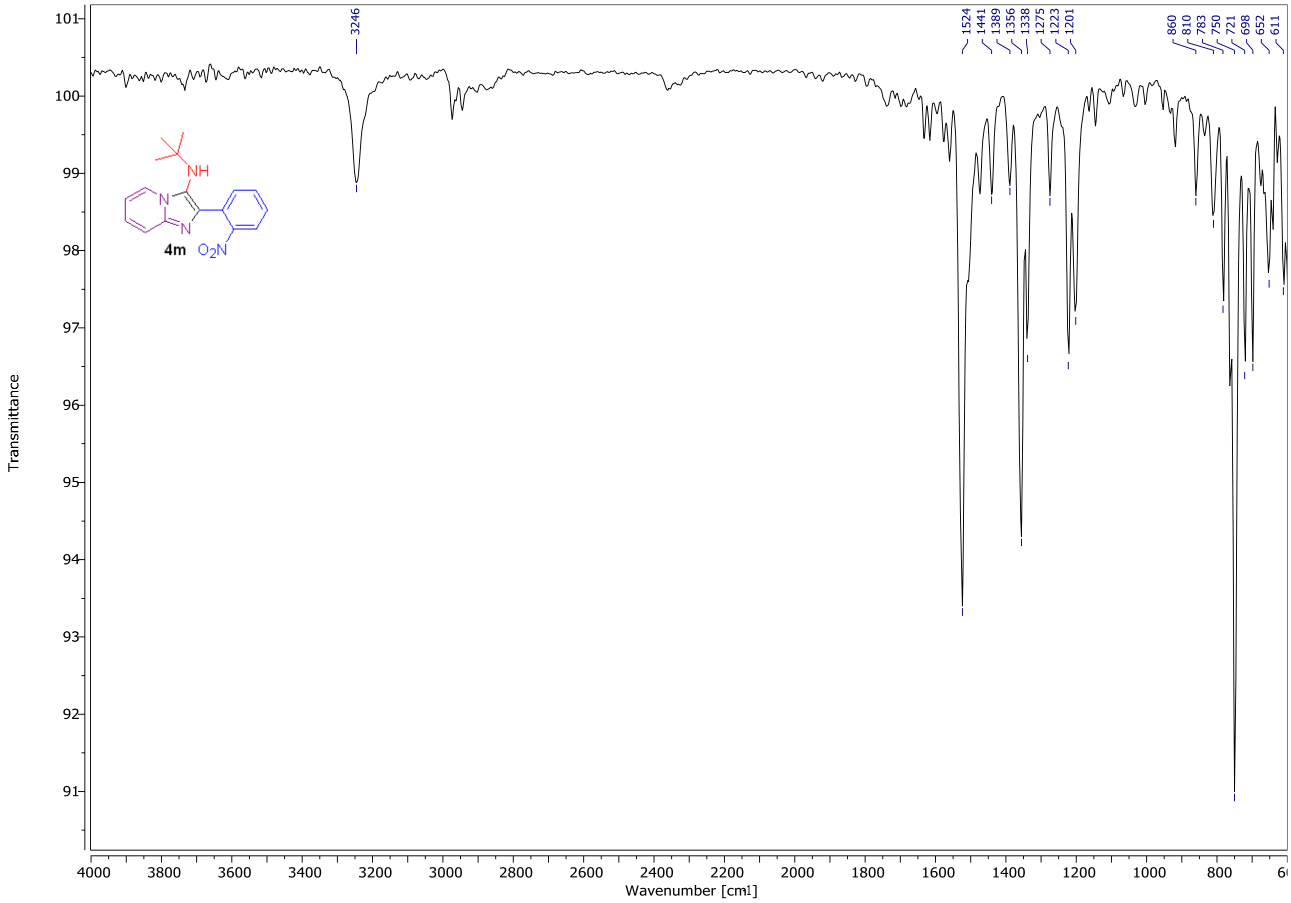

S 49. FT-IR (ATR) of compound **4m**.

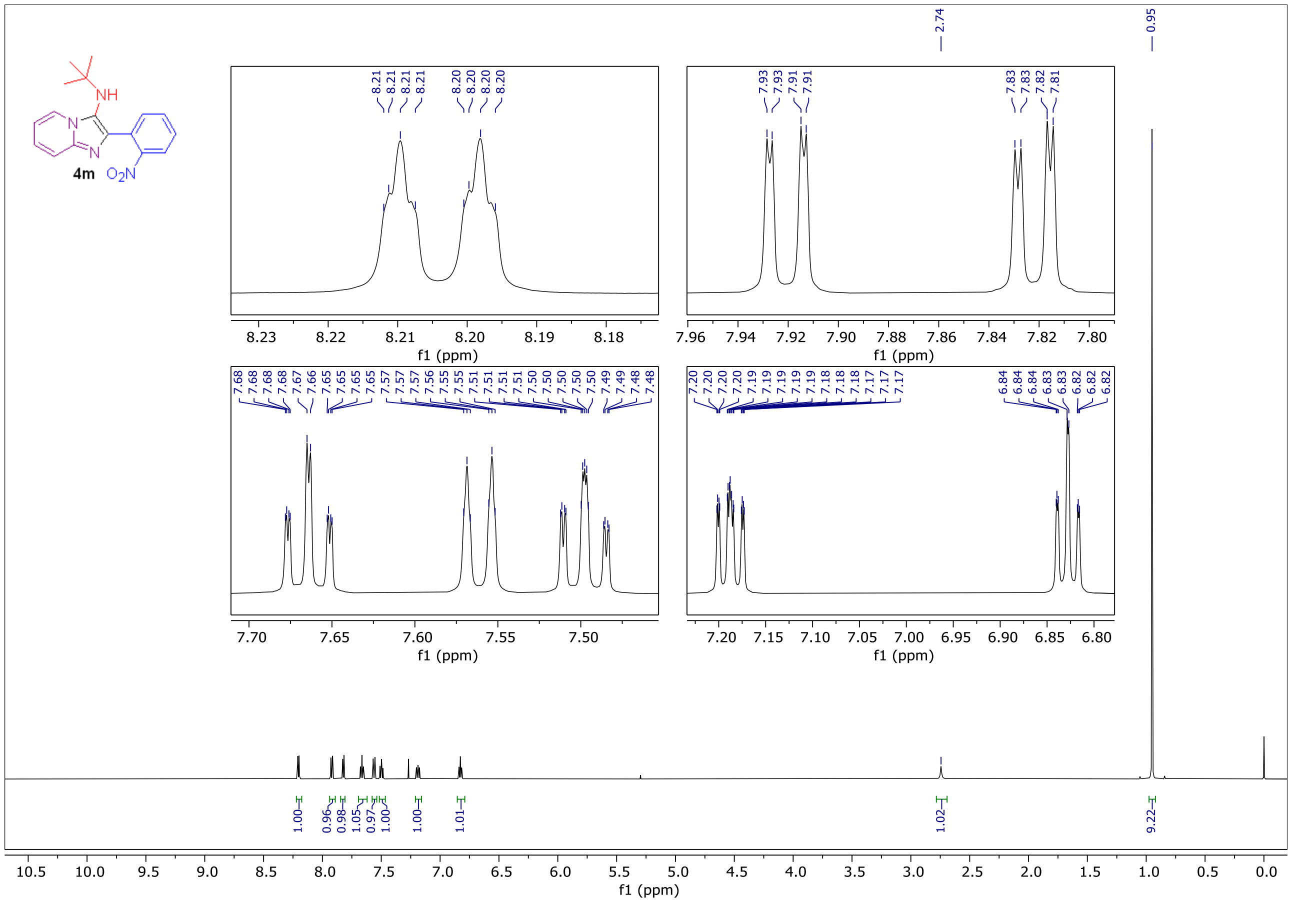

S 50. ^{1}H NMR spectrum (600 MHz, $CDCl_3$) of compound **4m**.

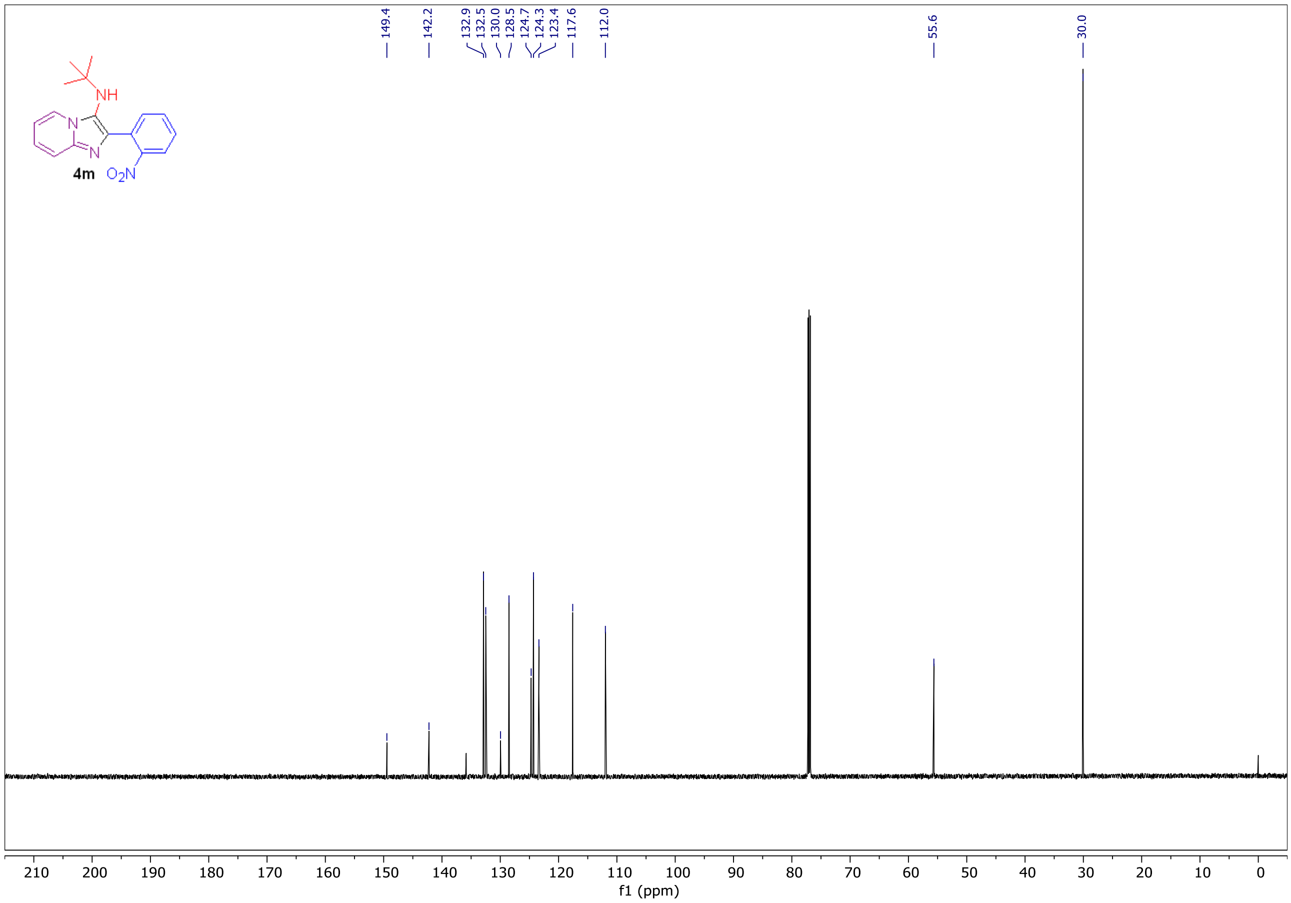

S 51. ^{13}C NMR spectrum (151 MHz, $CDCl_3$) of compound **4m**.

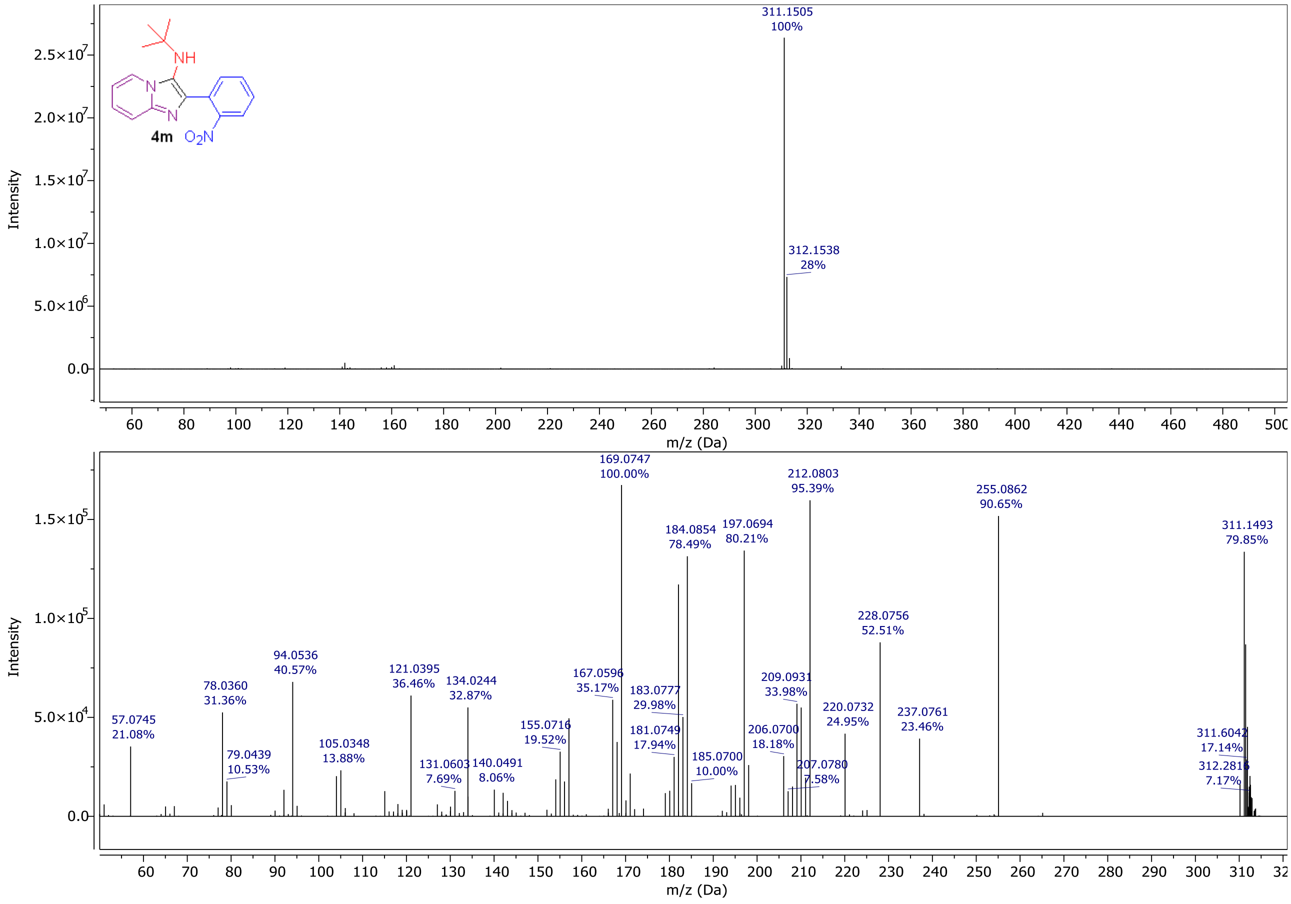

S 52. HRMS (ESI-QTOF) of compound **4m** and HRMS/MS for $[M+H]^+$.

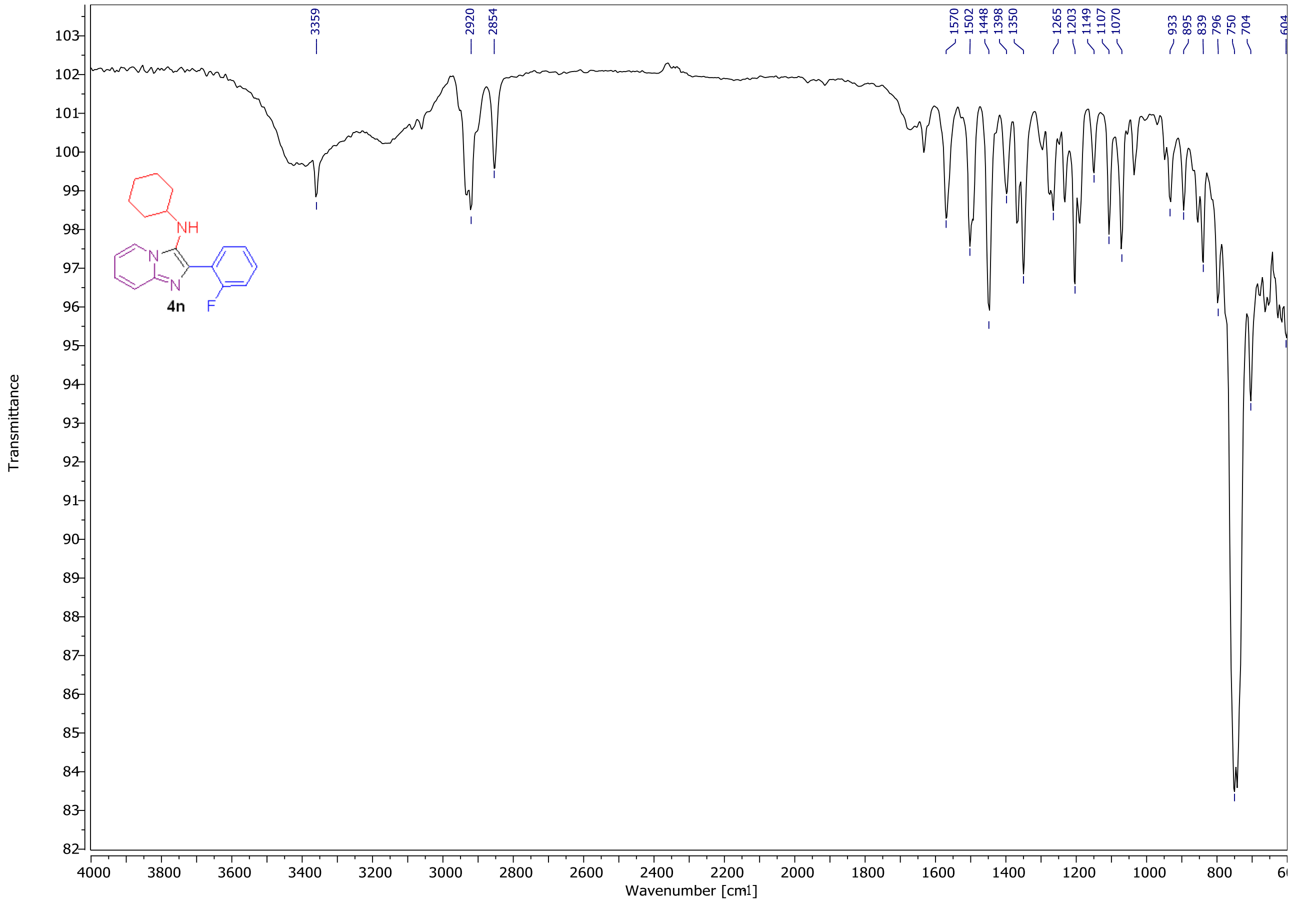

S 53. FT-IR (ATR) of compound **4n**.

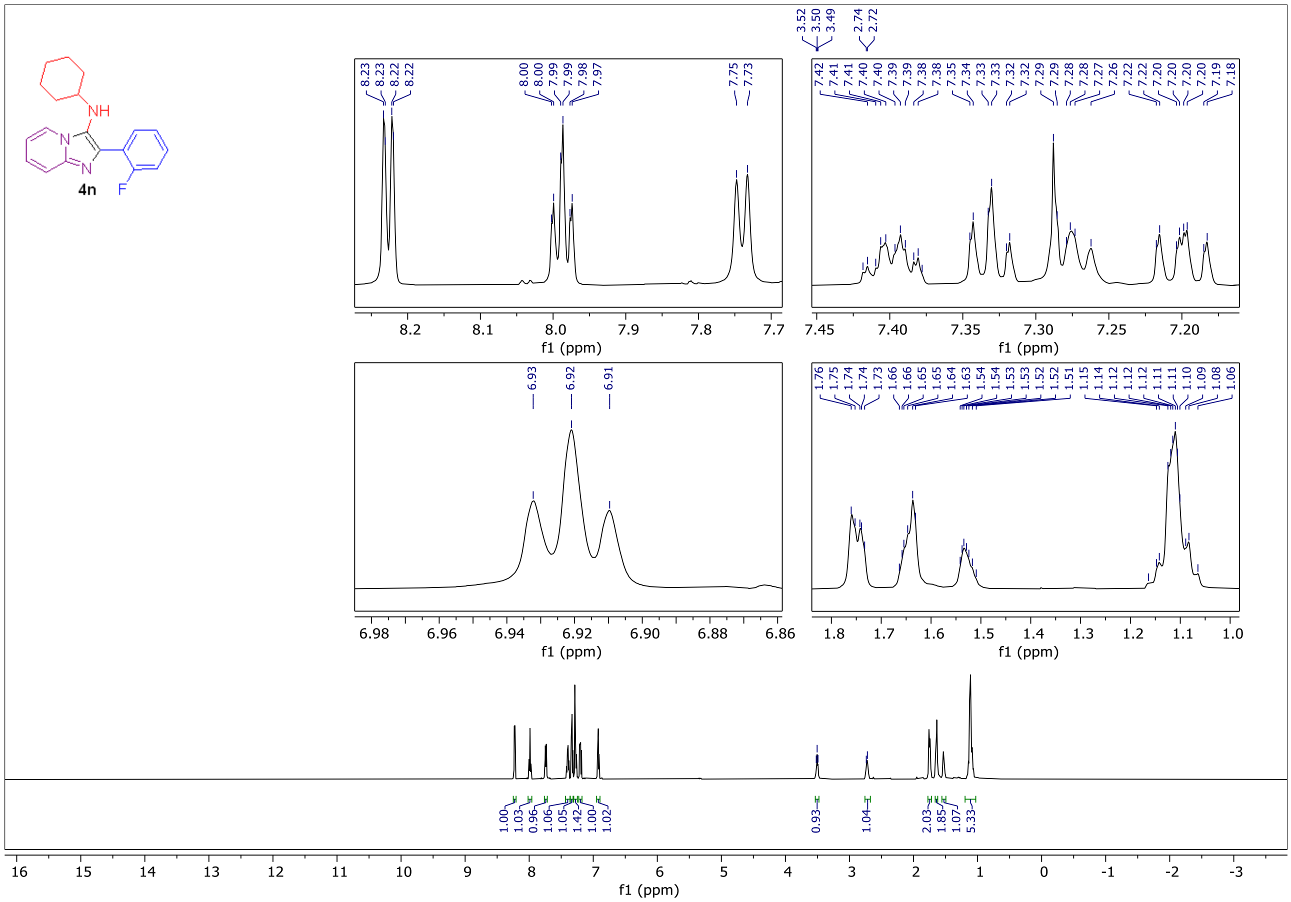

S 54. ^{1}H NMR spectrum (600 MHz, $CDCl_3$) of compound **4n**.

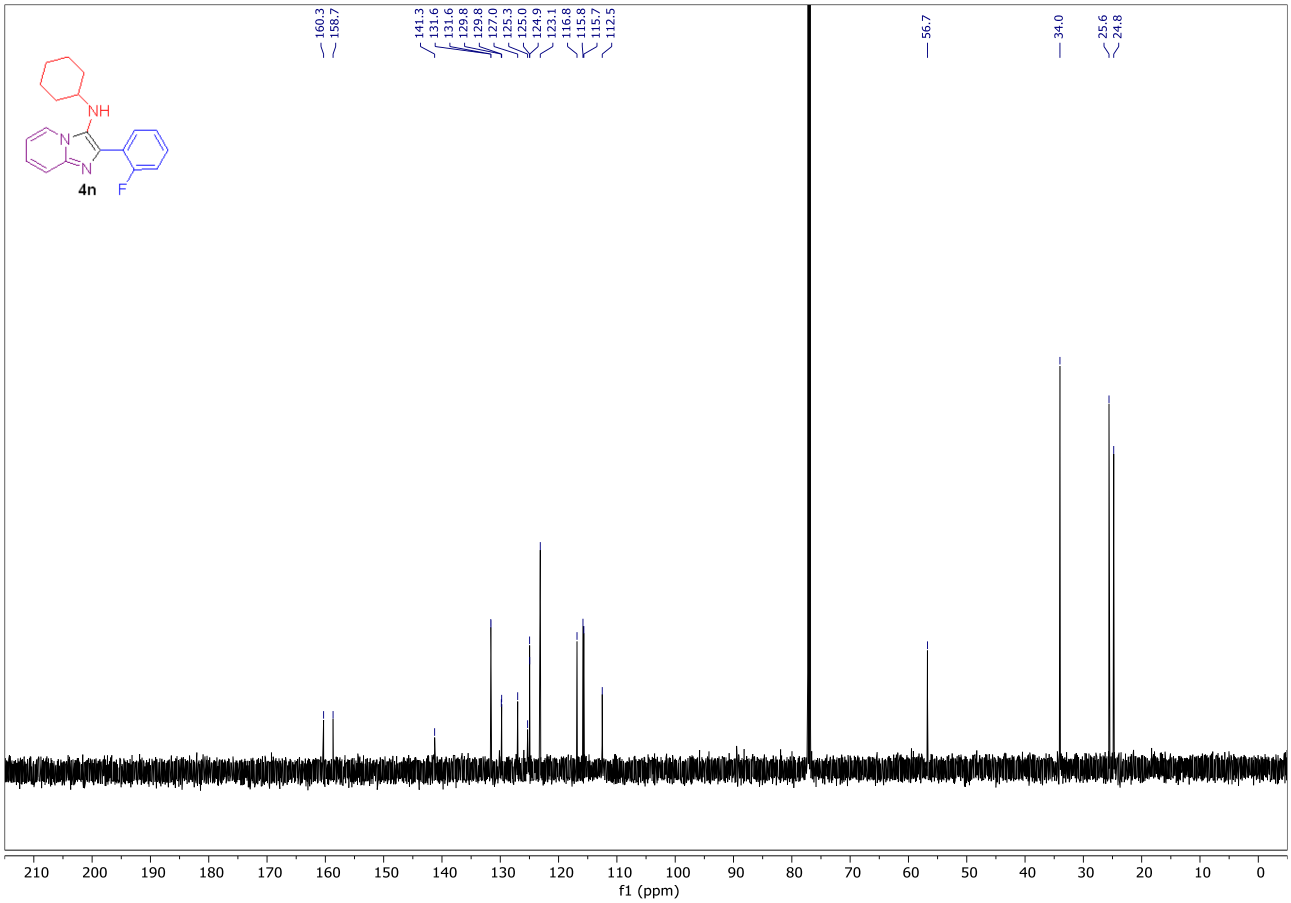

S 55. ^{13}C NMR spectrum (151 MHz, $CDCl_3$) of compound **4n**.

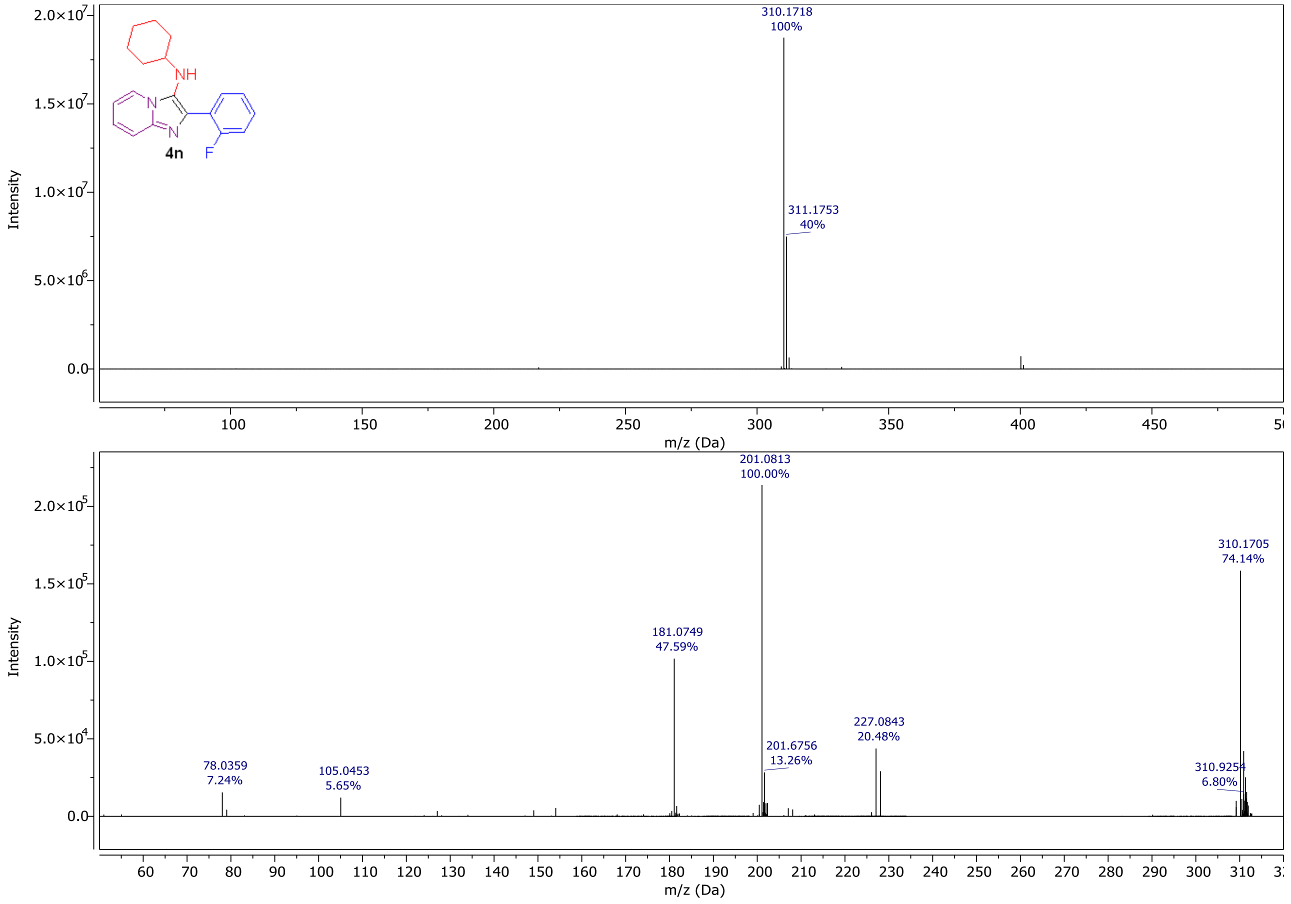

S 56. HRMS (ESI-QTOF) of compound **4n** and HRMS/MS for $[M+H]^+$.

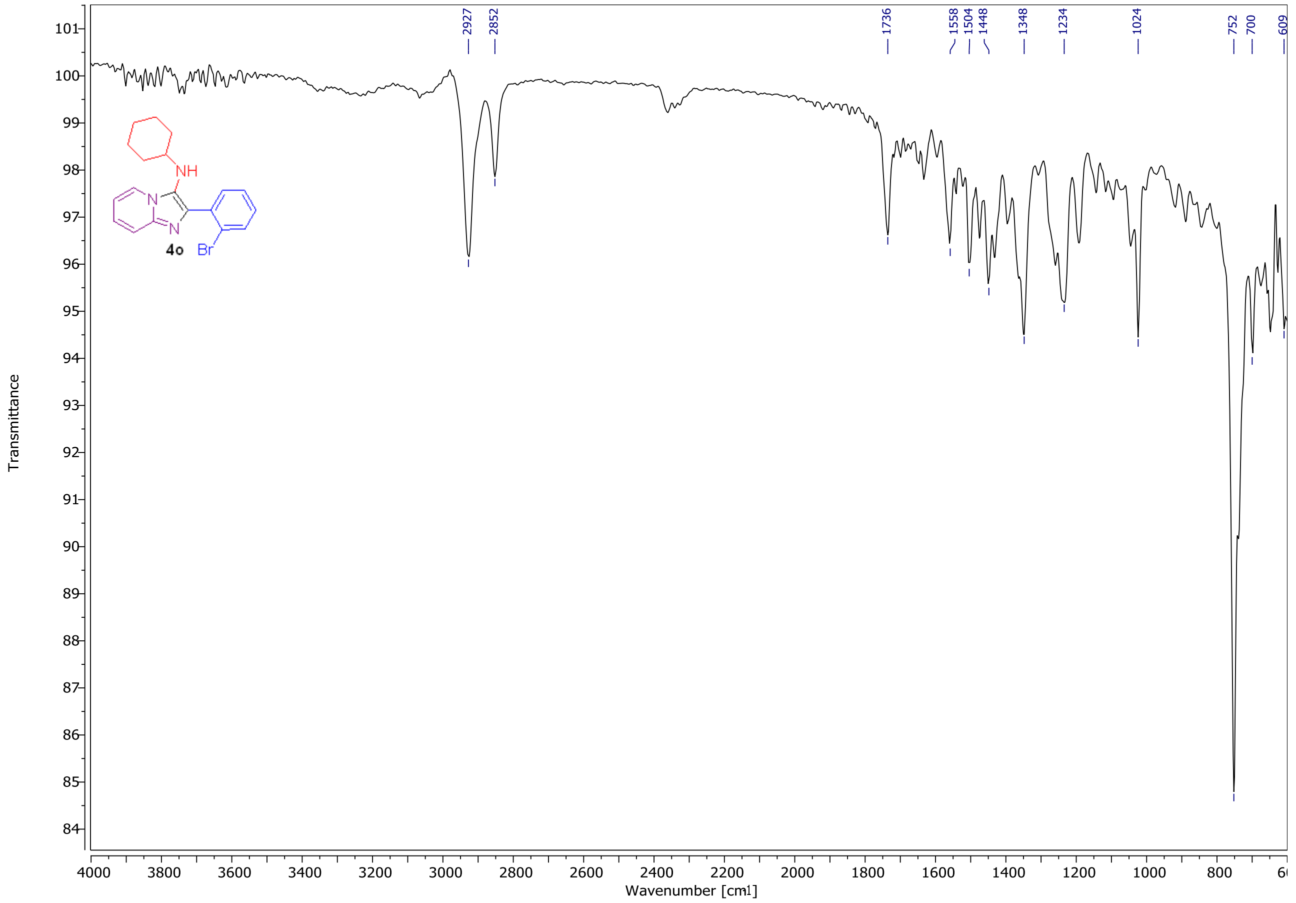

S 57. FT-IR (ATR) of compound **4o**.

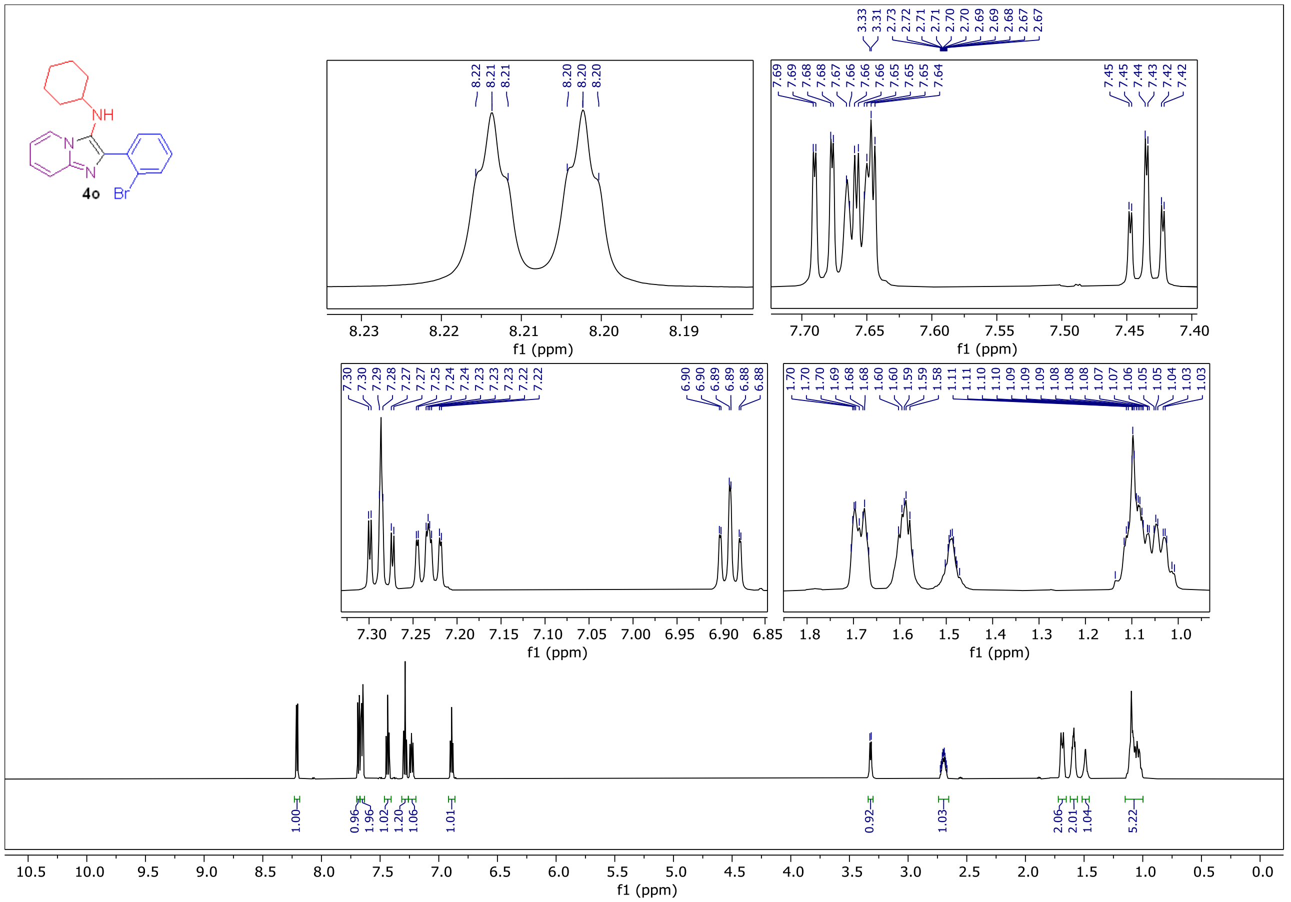

S 58. ^{1}H NMR spectrum (600 MHz, $CDCl_3$) of compound **4o**.

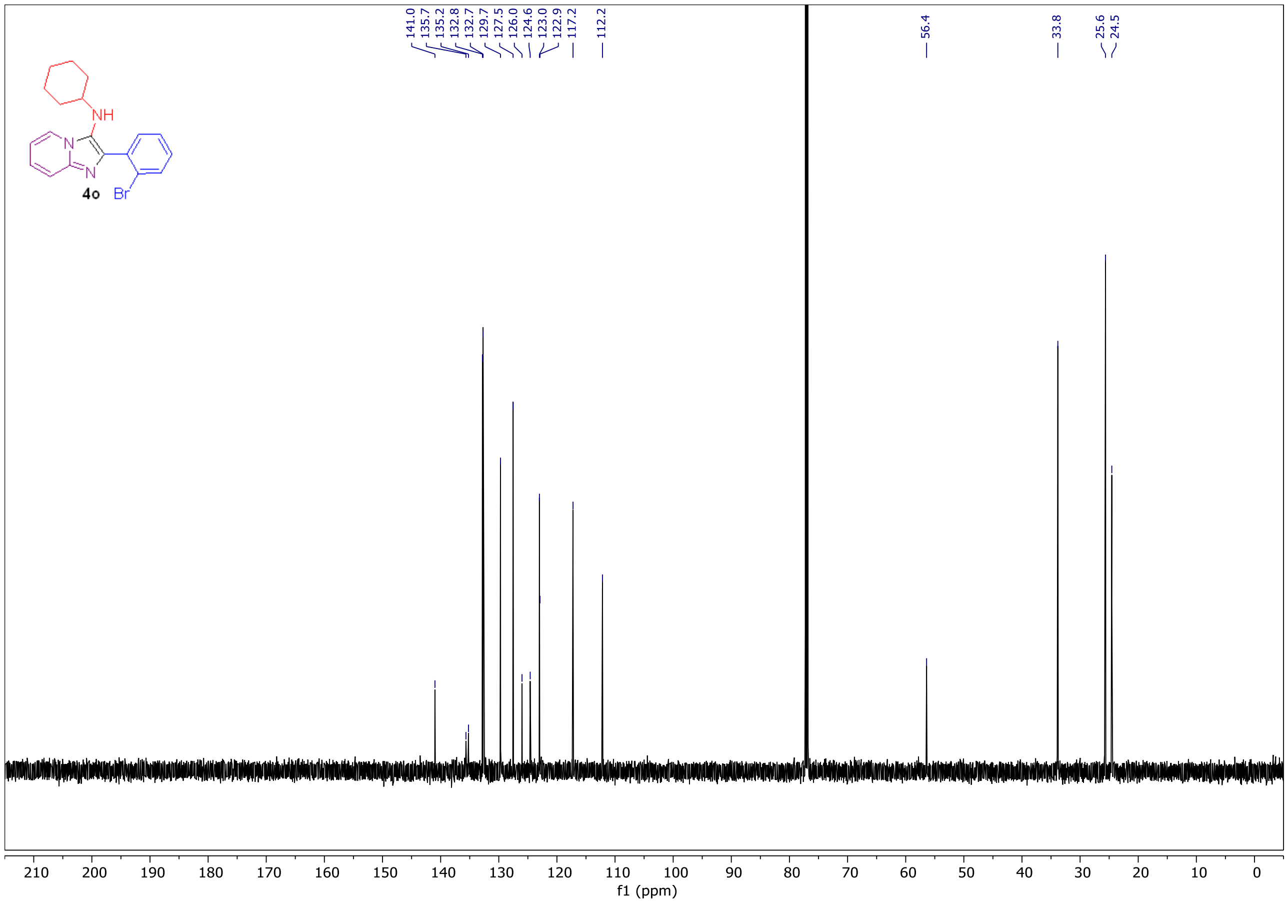

S 59. ^{13}C NMR spectrum (151 MHz, $CDCl_3$) of compound **4o**.

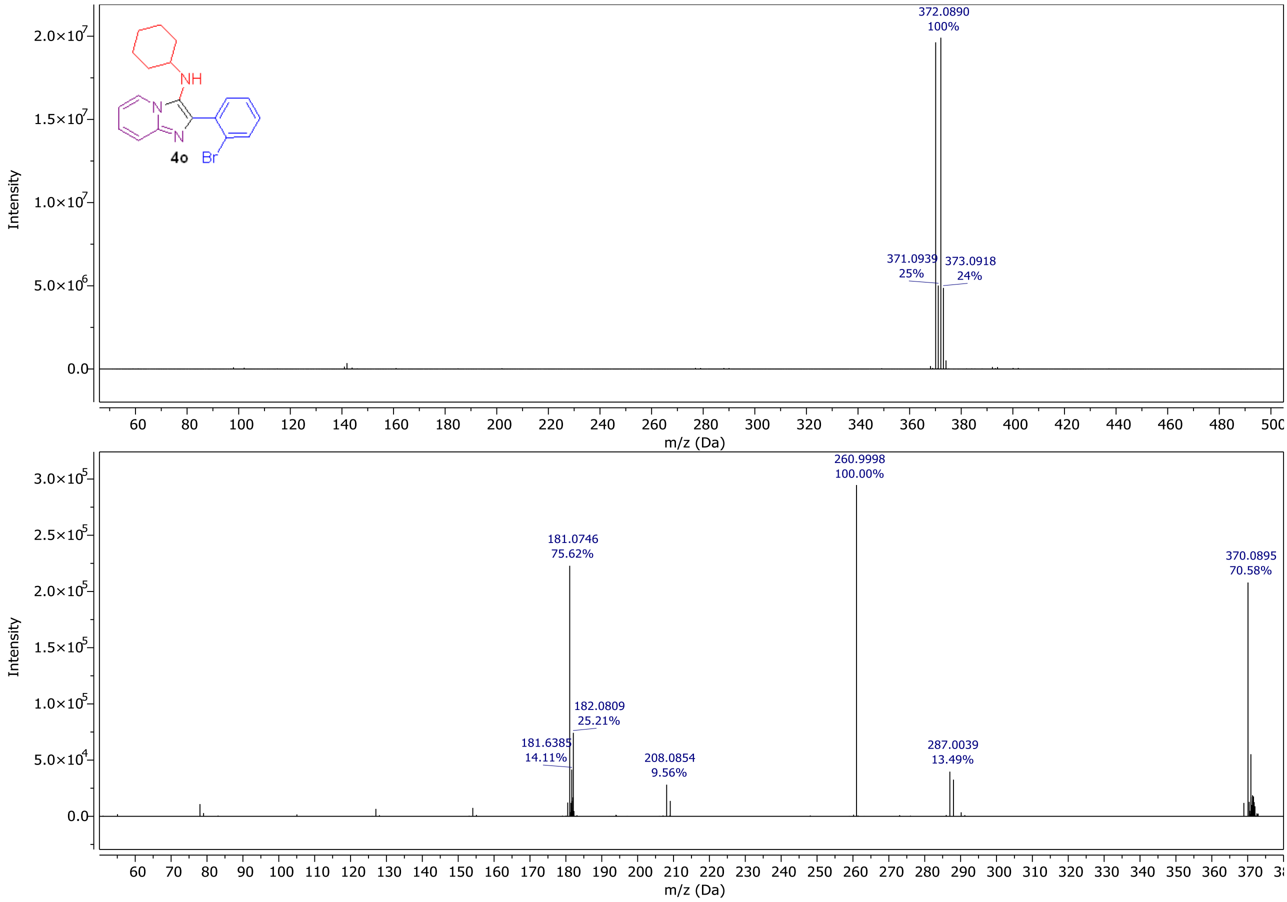

S 60. HRMS (ESI-QTOF) of compound **4o** and HRMS/MS for $[M+H]^+$.

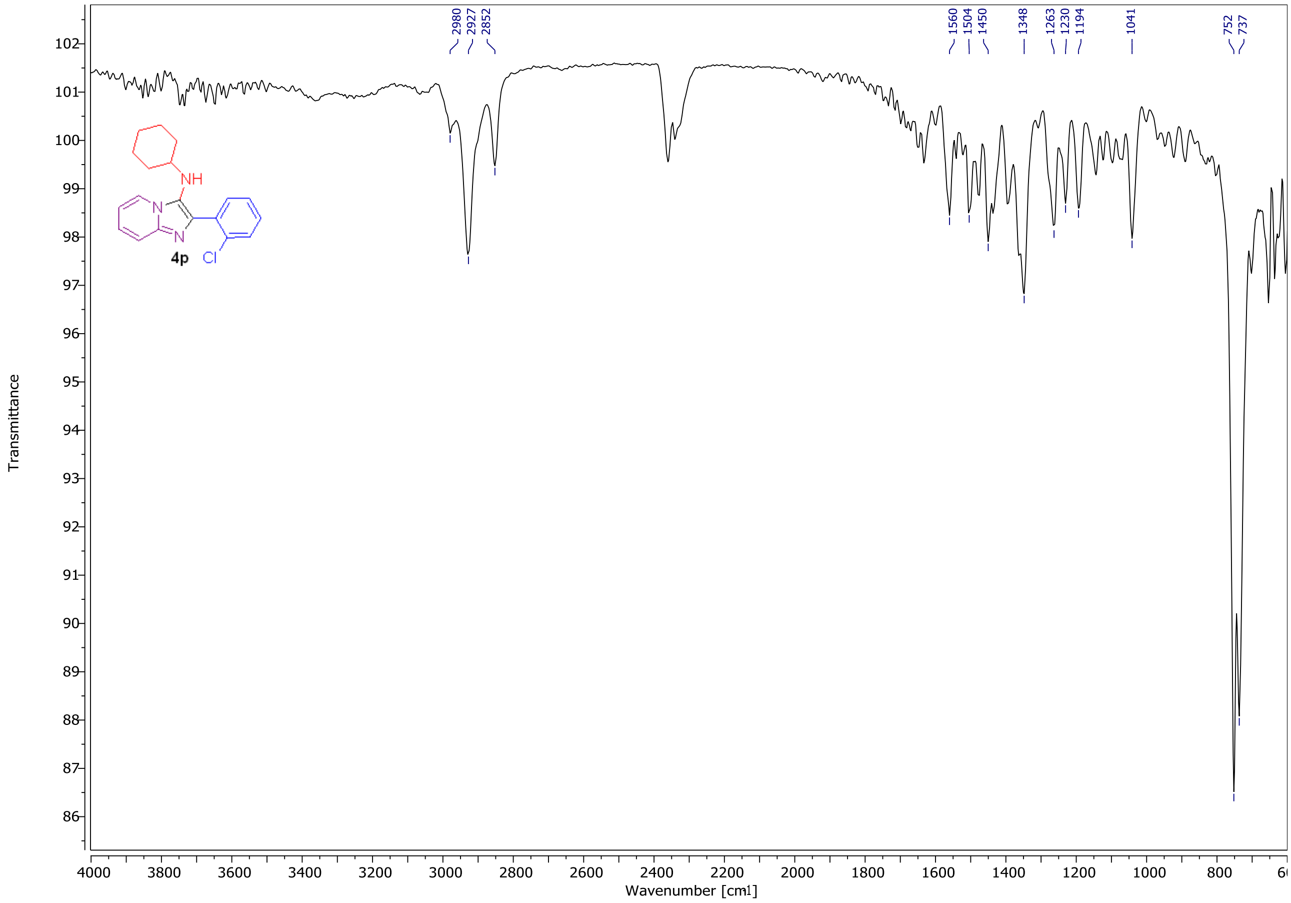

S 61. FT-IR (ATR) of compound **4p**.

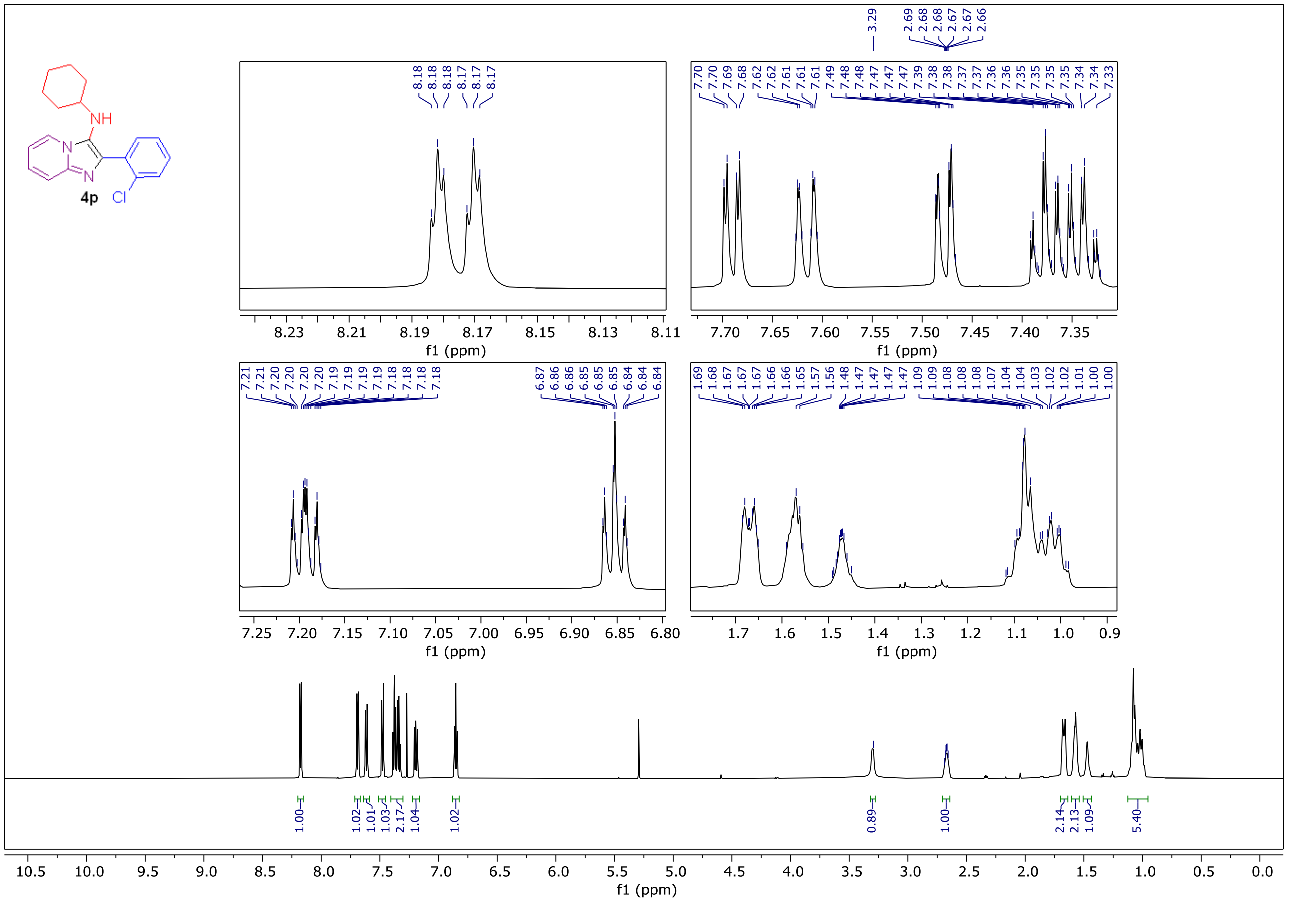

S 62. ^{1}H NMR spectrum (600 MHz, $CDCl_3$) of compound **4p**.

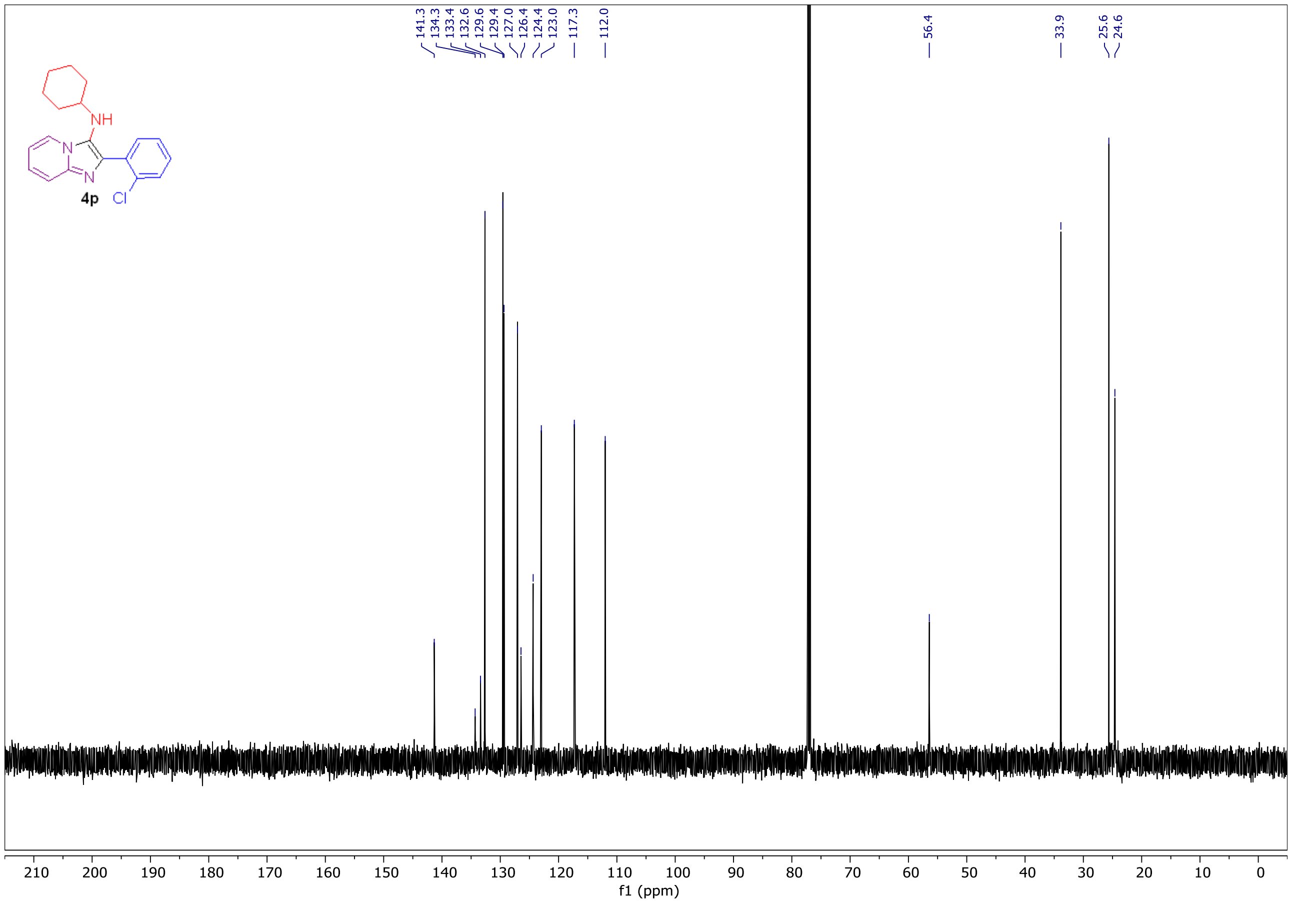

S 63. ^{13}C NMR spectrum (151 MHz, $CDCl_3$) of compound **4p**.

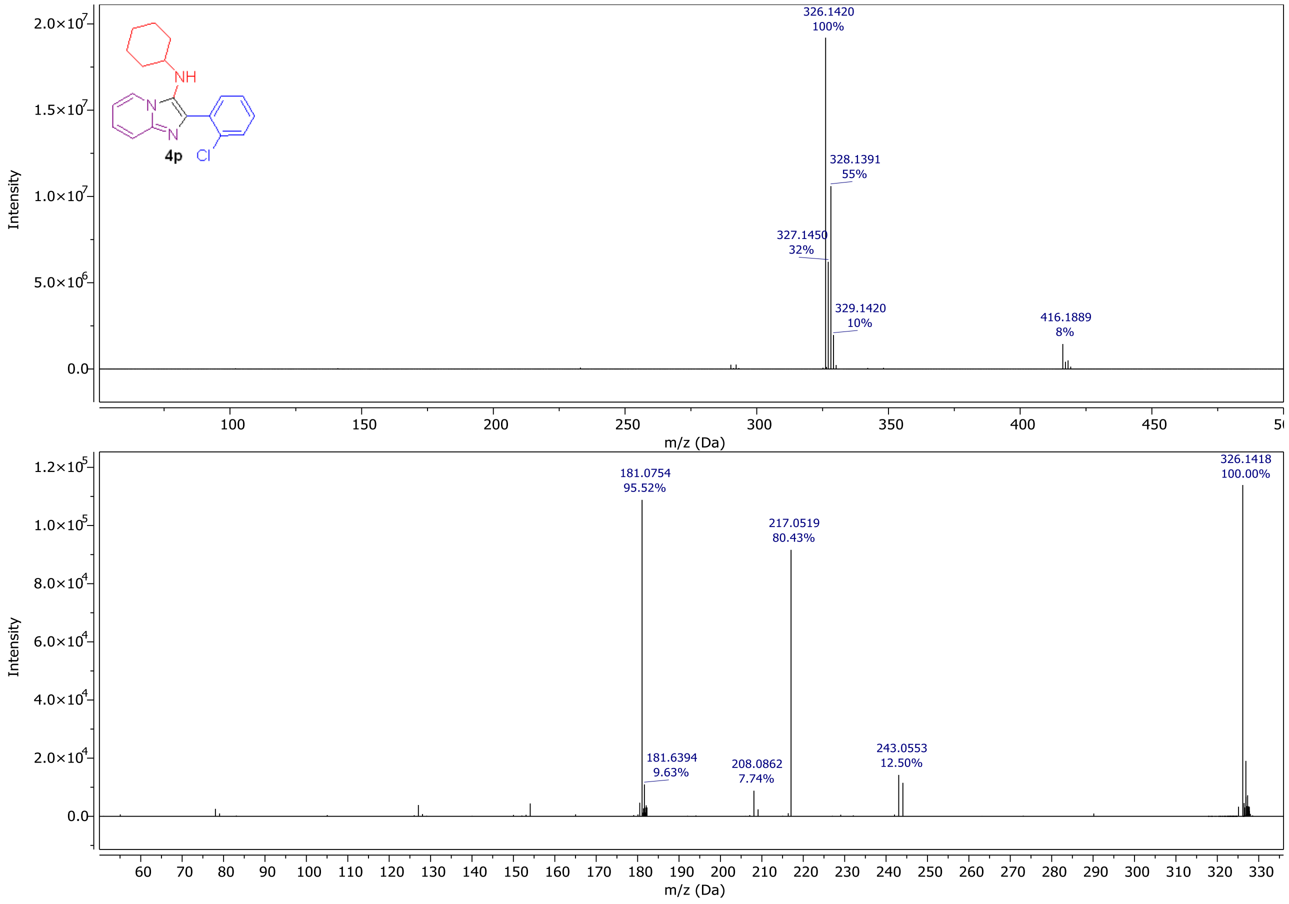

S 64. HRMS (ESI-QTOF) of compound **4p** and HRMS/MS for $[M+H]^+$.

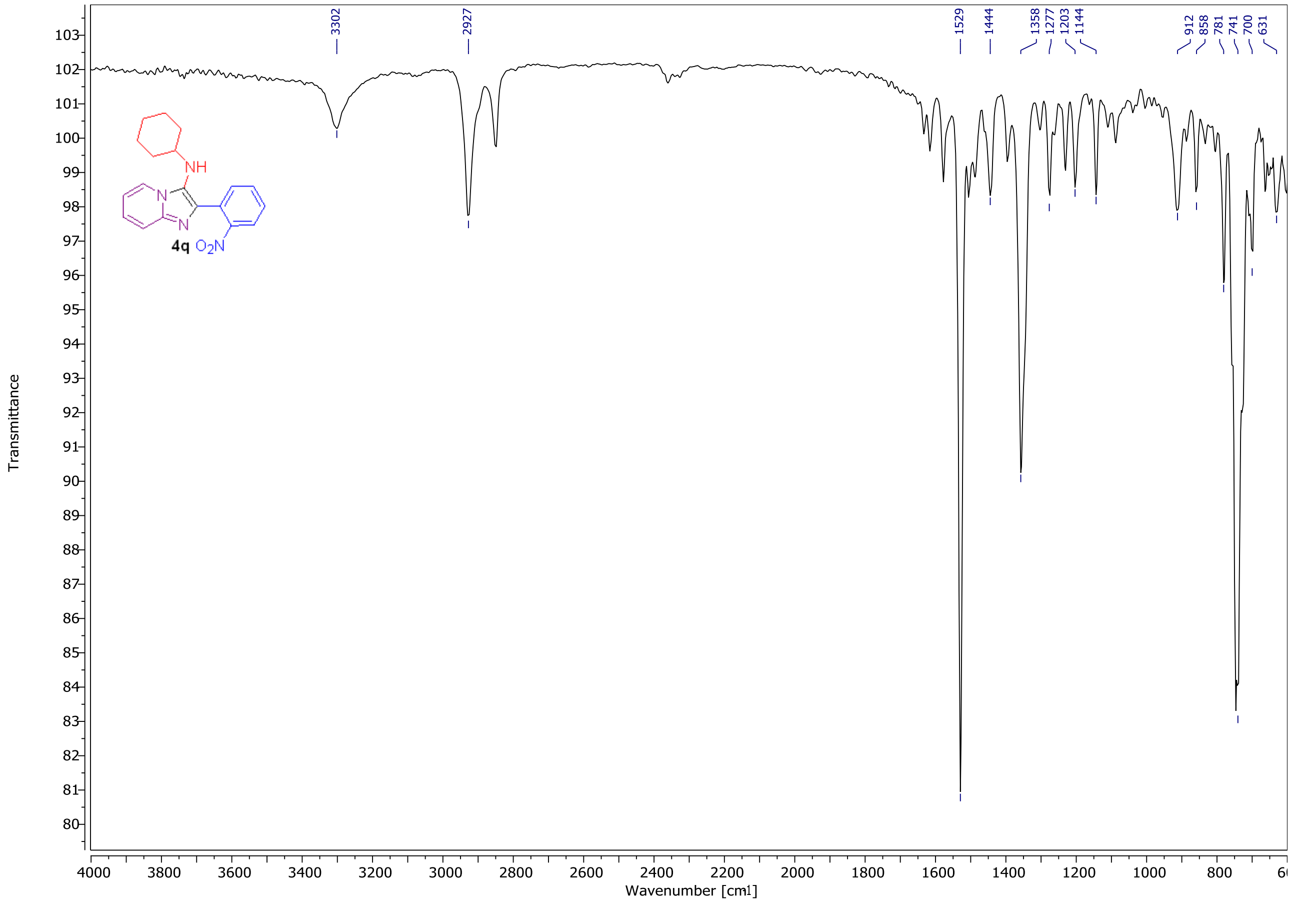

S 65. FT-IR (ATR) of compound **4q**.

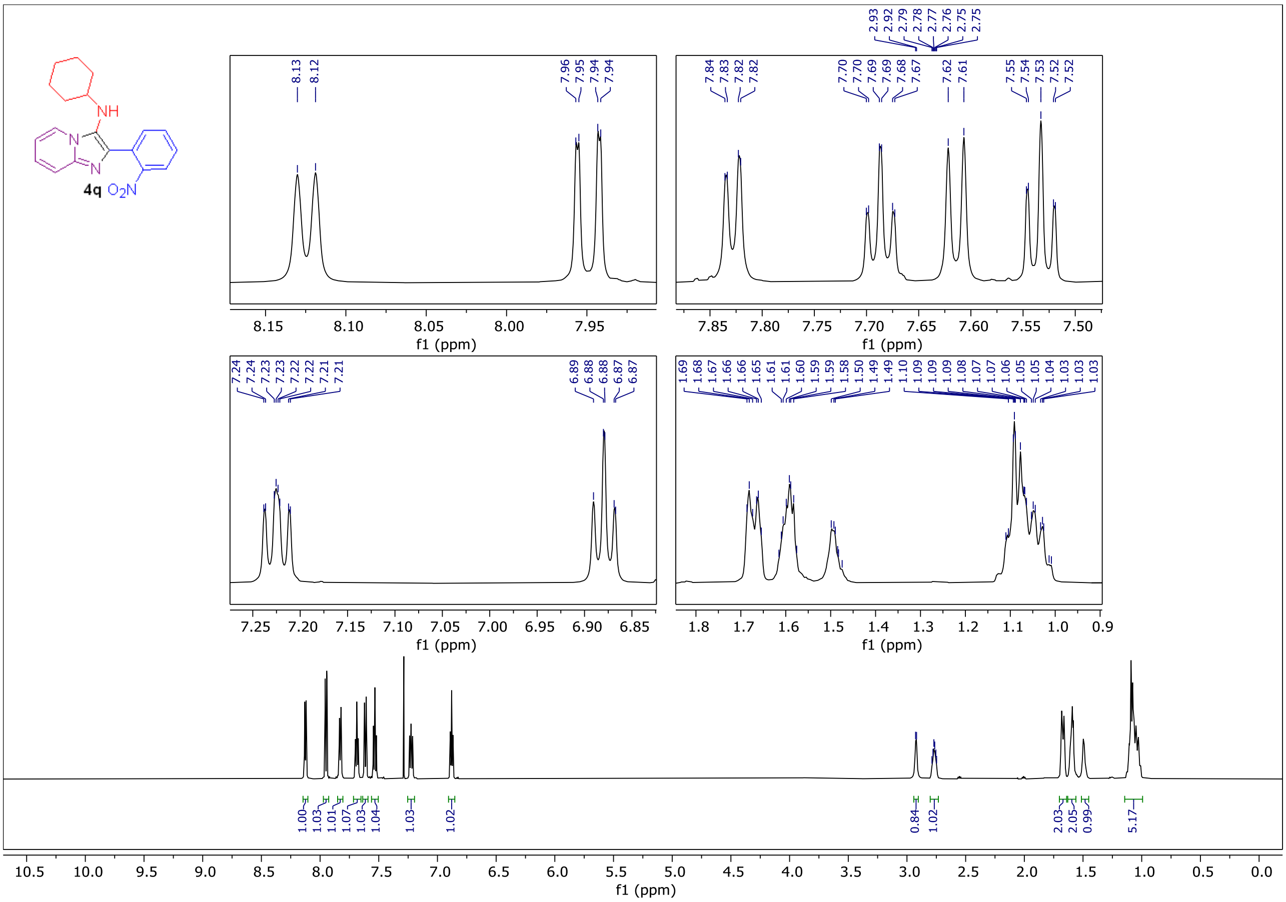

S 66. ^{1}H NMR spectrum (600 MHz, $CDCl_3$) of compound **4q**.

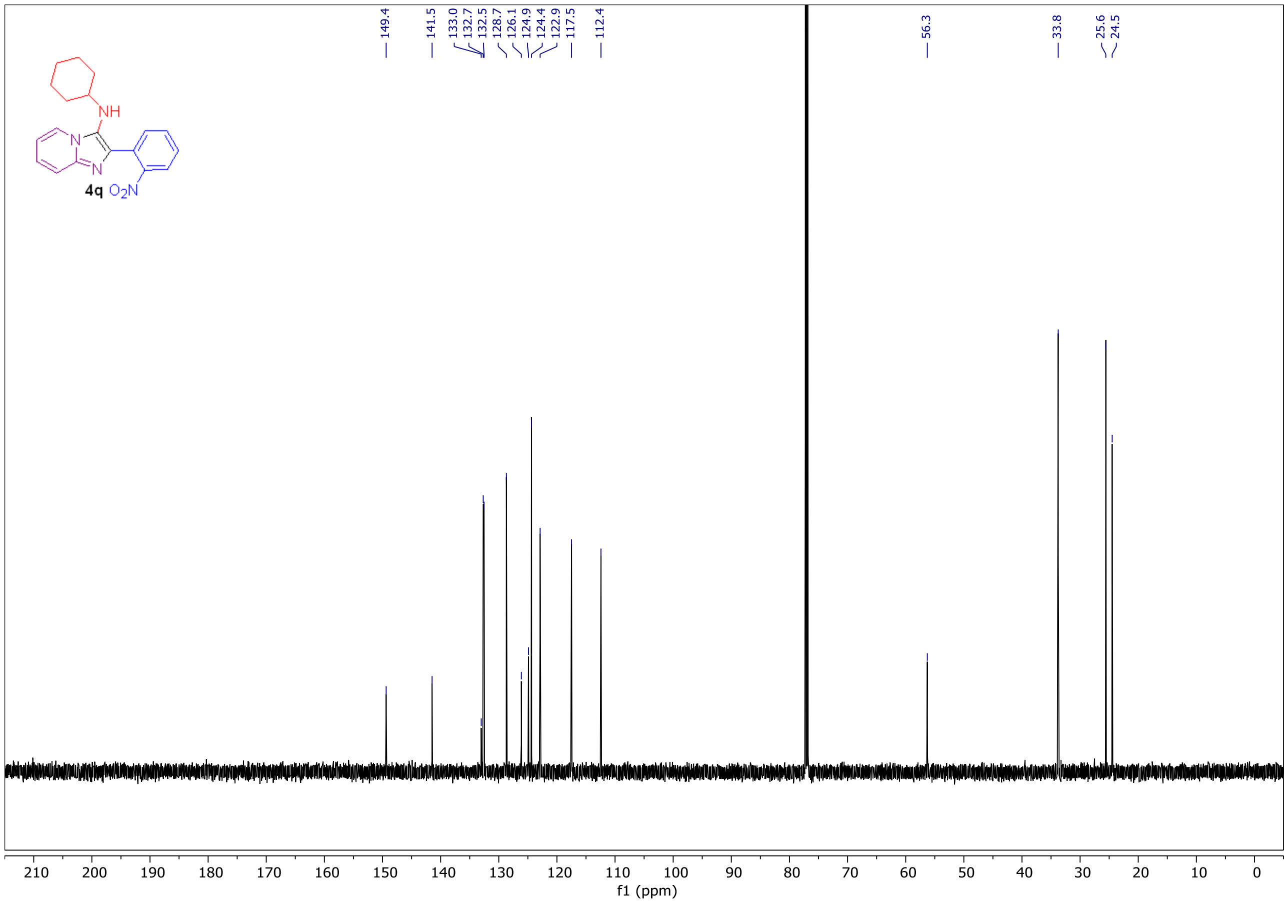

S 67. ^{13}C NMR spectrum (151 MHz, $CDCl_3$) of compound **4q**.

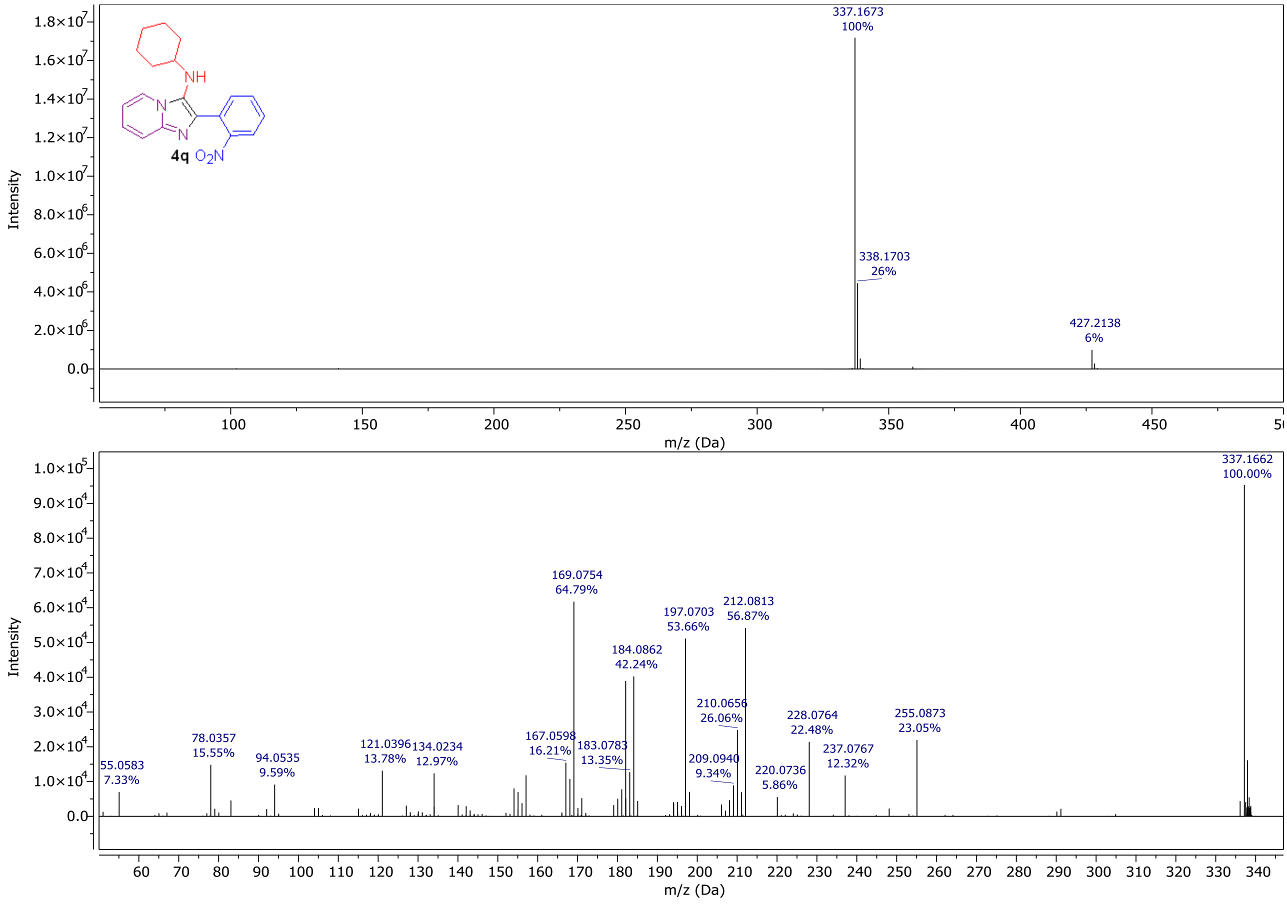

S 68. HRMS (ESI-QTOF) of compound **4q** and HRMS/MS for [M+H]$^+$.

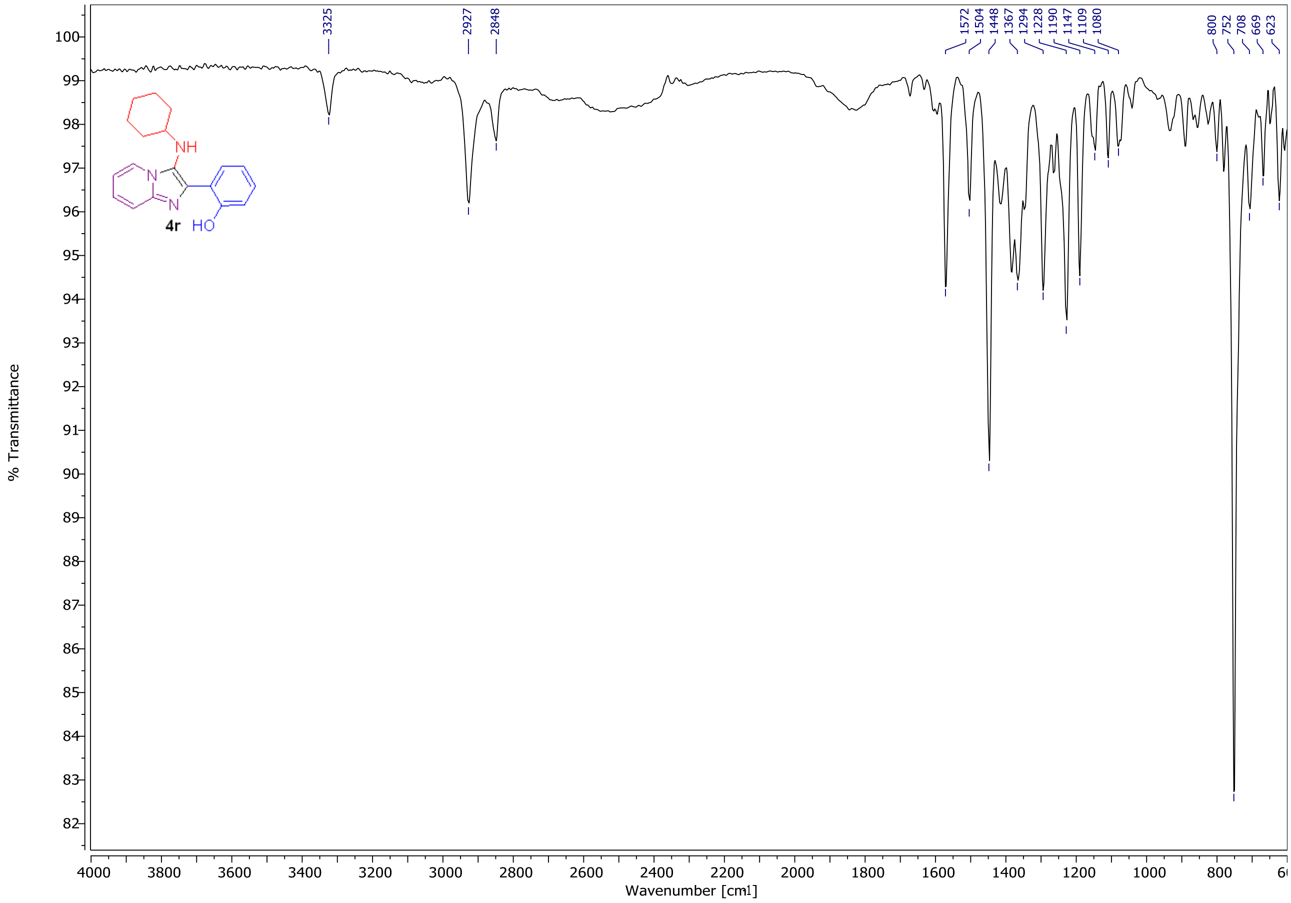

S 69. FT-IR (ATR) of compound **4r**.

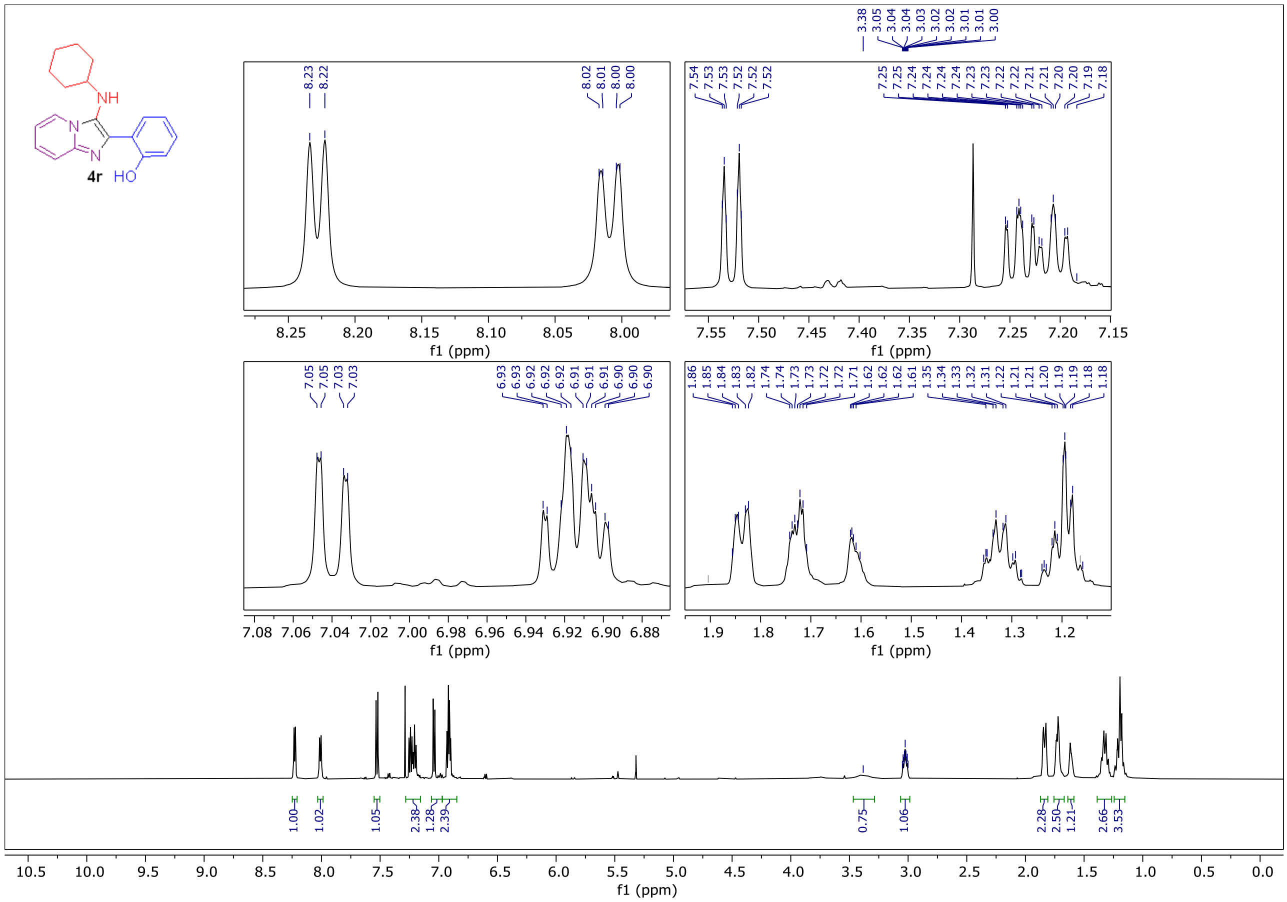

S 70. ^{1}H NMR spectrum (600 MHz, $CDCl_3$) of compound **4r**.

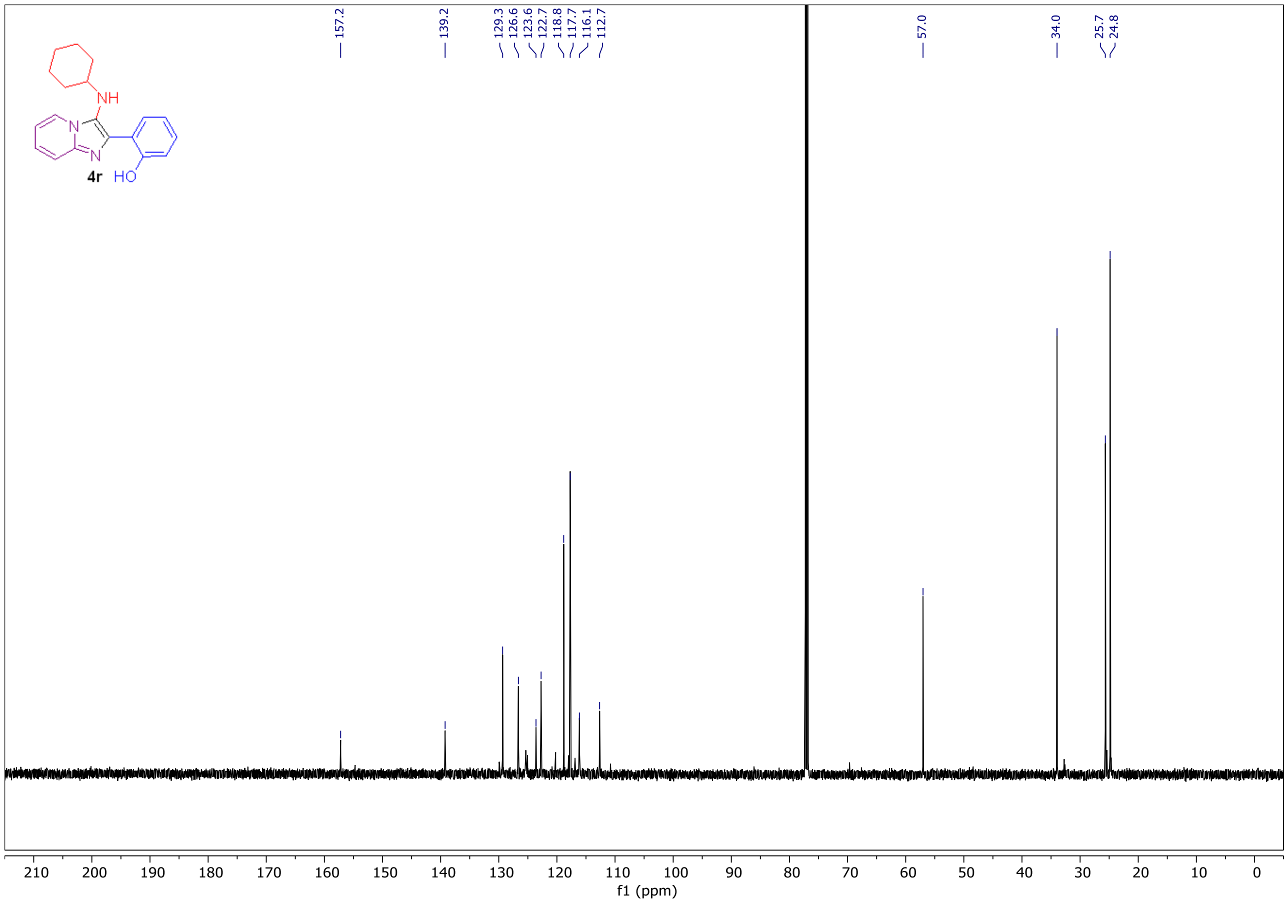

S 71. ^{13}C NMR spectrum (151 MHz, $CDCl_3$) of compound **4r**.

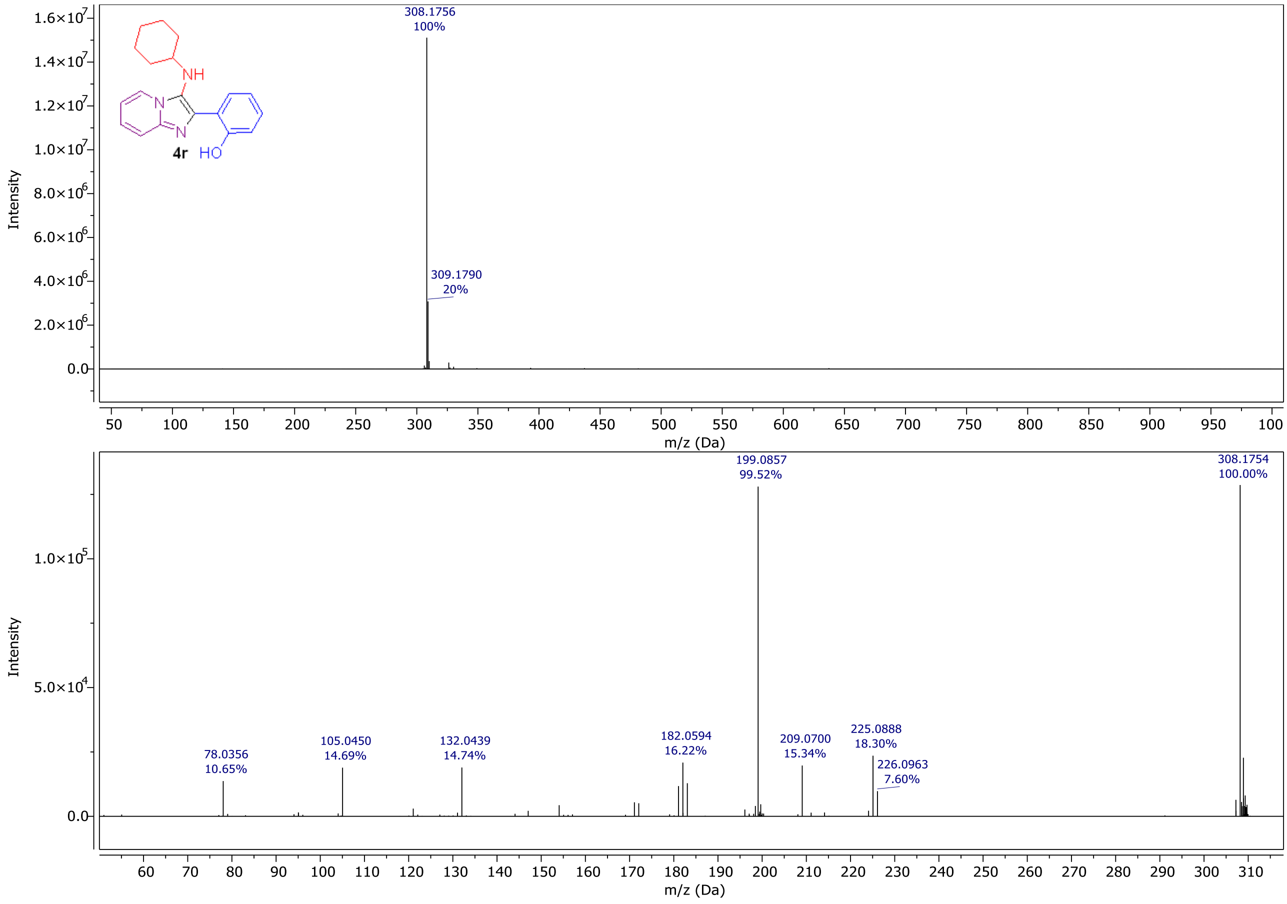

S 72. HRMS (ESI-QTOF) of compound **4r** and HRMS/MS for $[M+H]^+$.

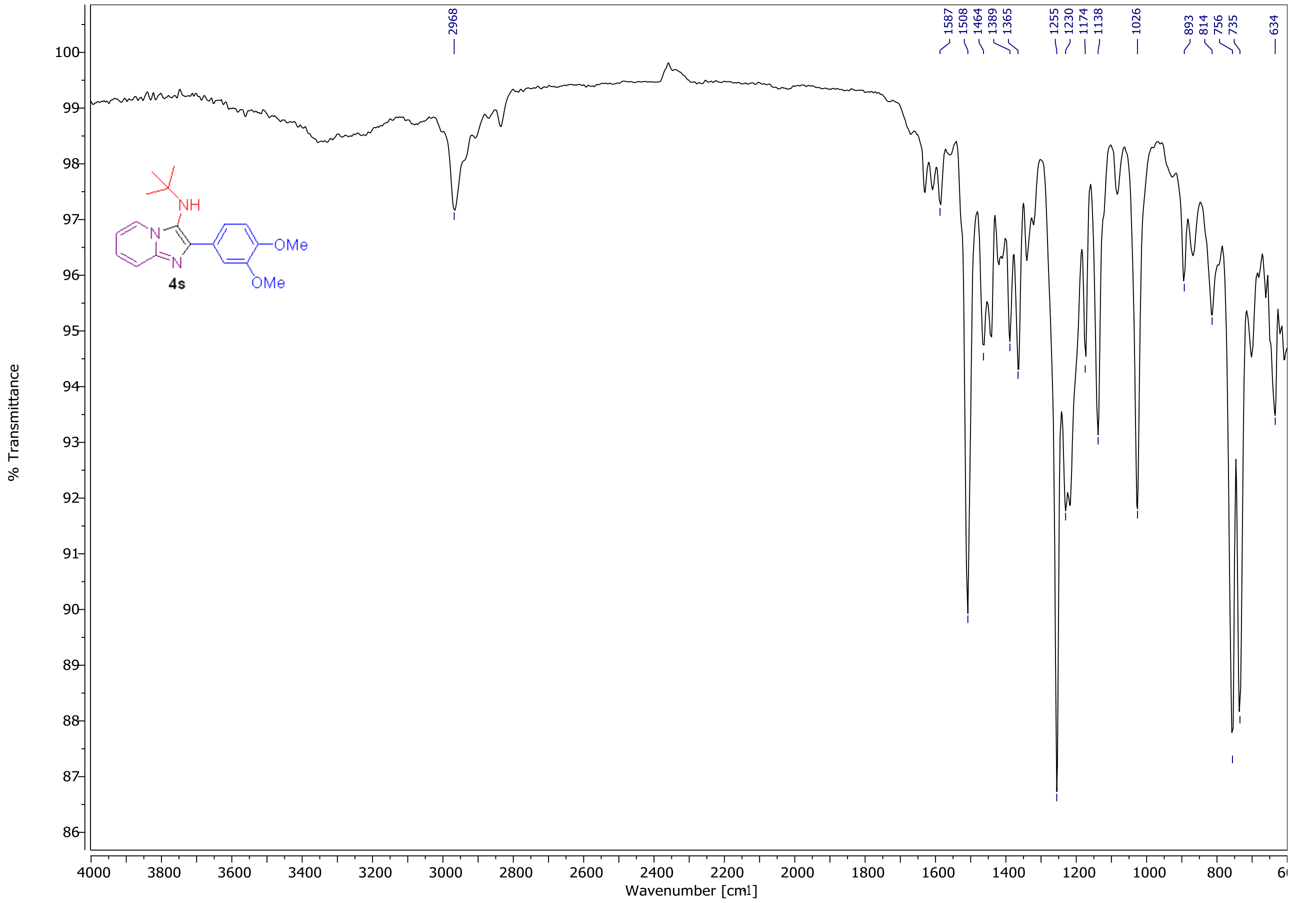

S 73. FT-IR (ATR) of compound **4s**.

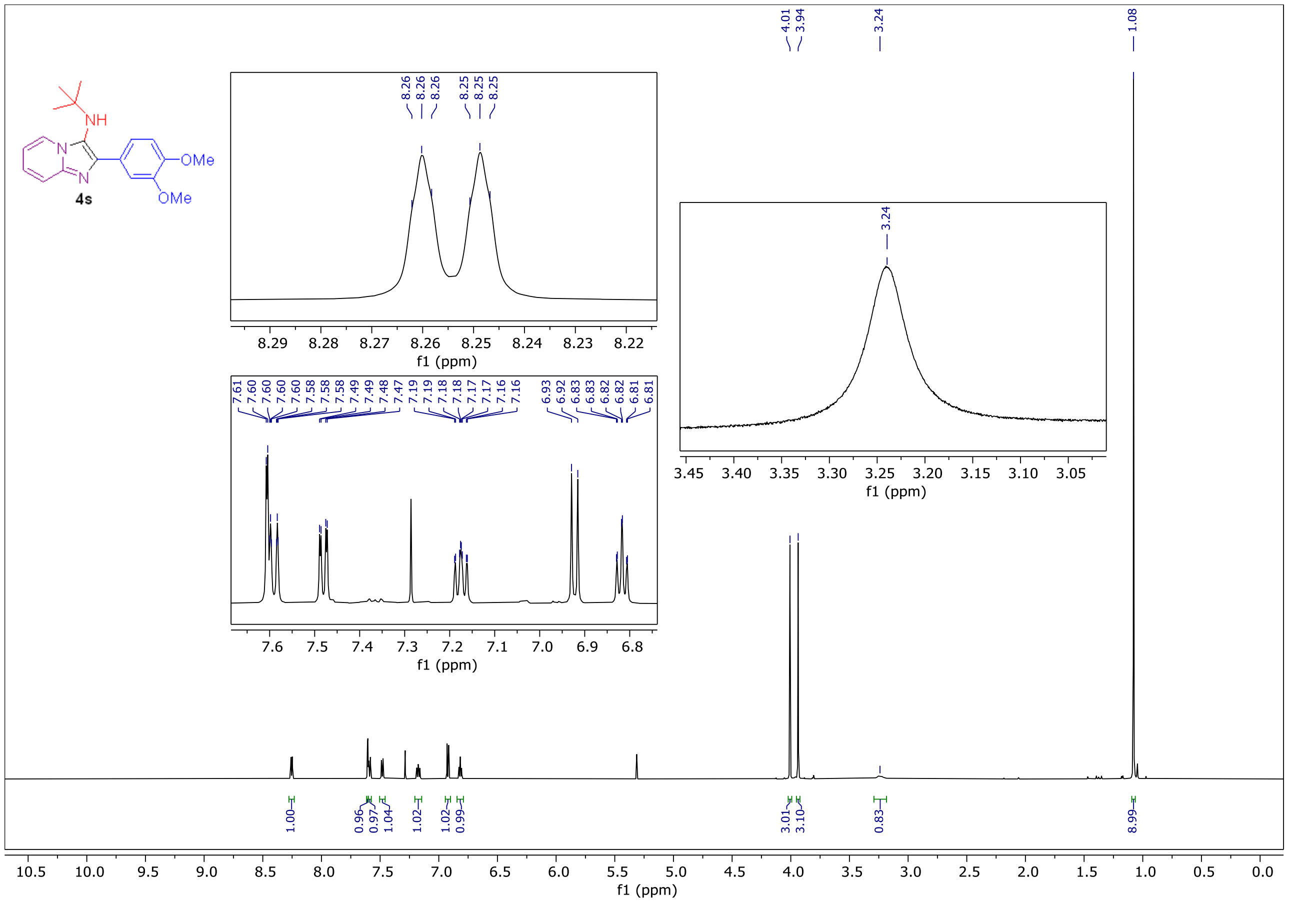

S 74. ^{1}H NMR spectrum (600 MHz, $CDCl_3$) of compound **4s**.

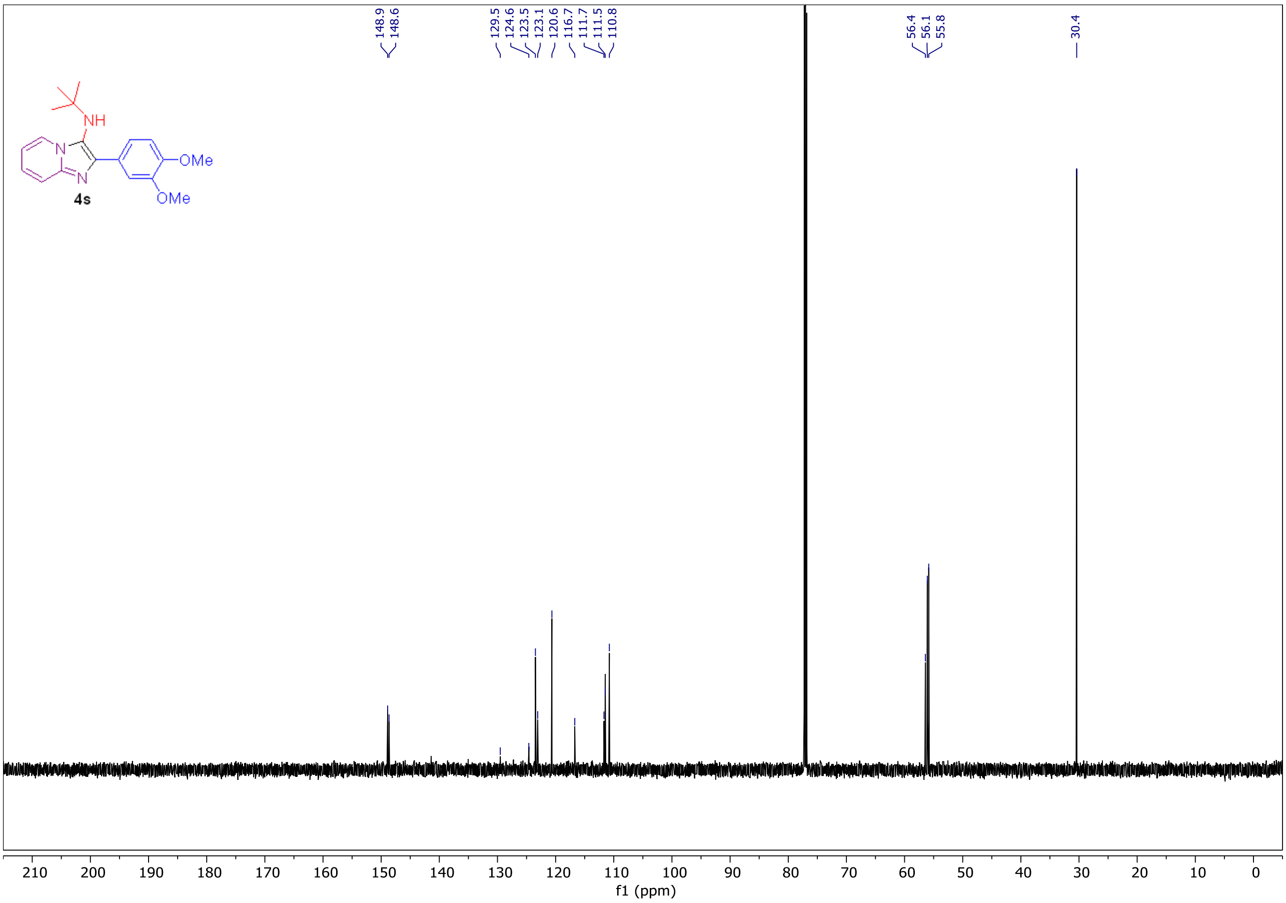

S 75. ^{13}C NMR spectrum (151 MHz, $CDCl_3$) of compound **4s**.

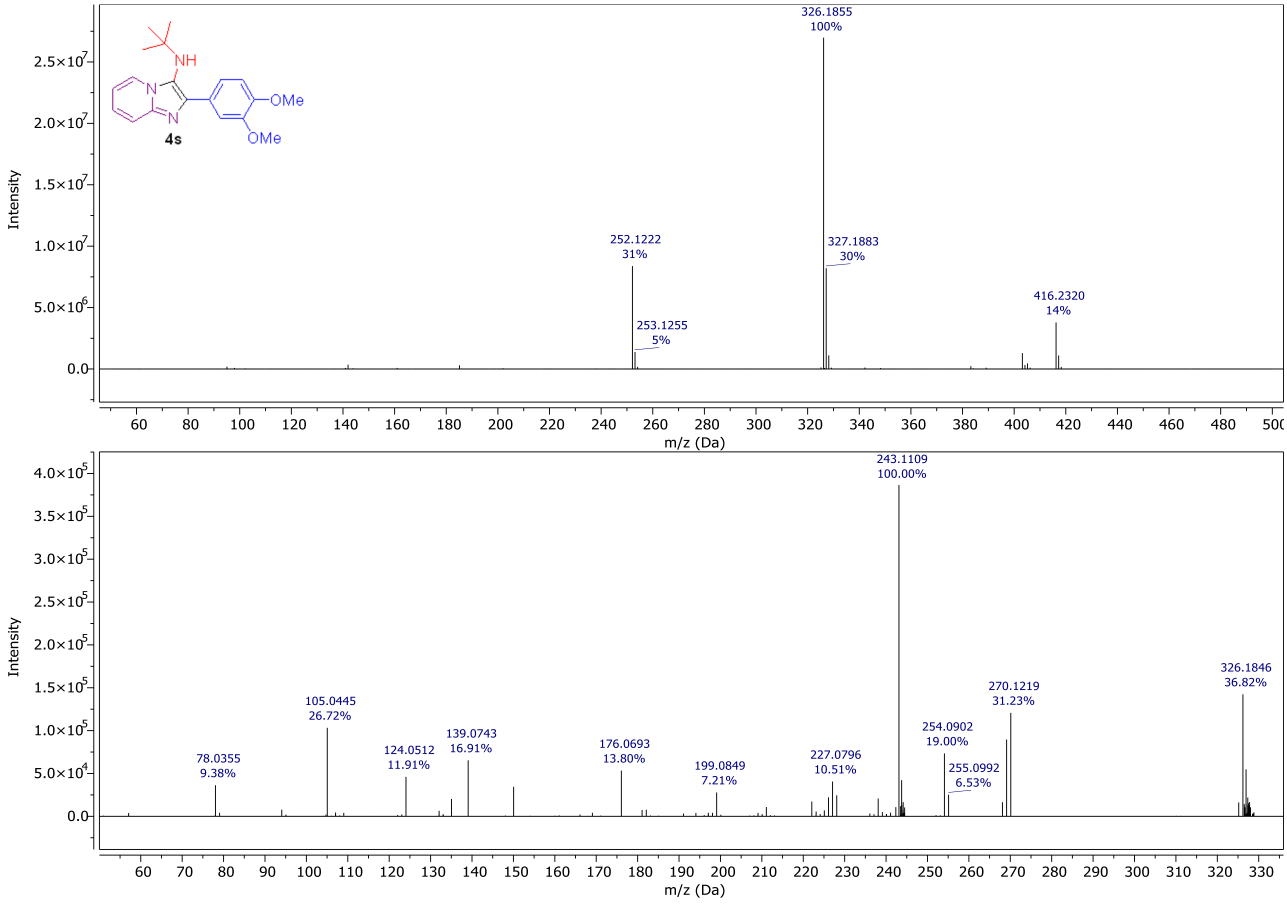

S 76. HRMS (ESI-QTOF) of compound **4s** and HRMS/MS for $[M+H]^+$.

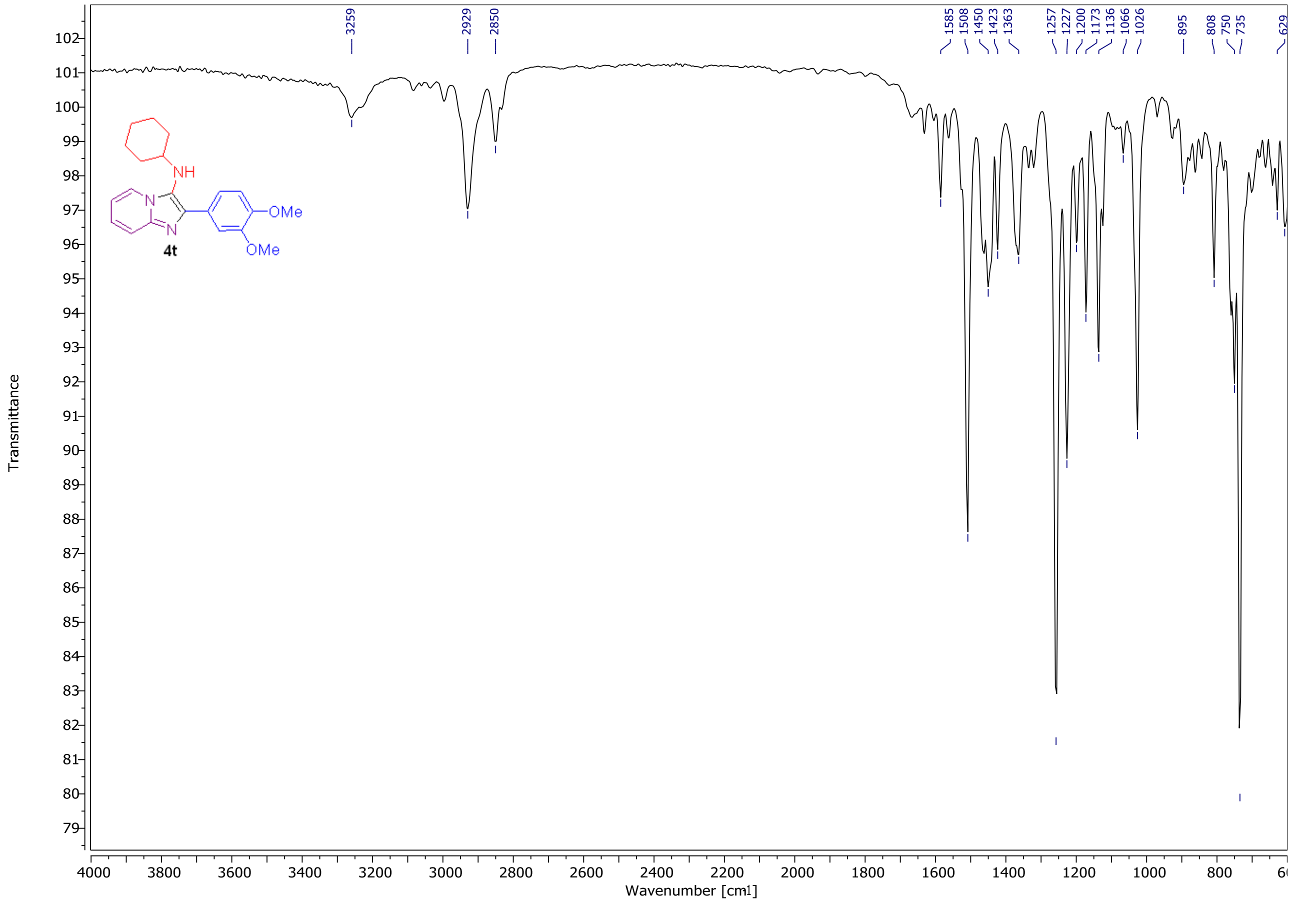

S 77. FT-IR (ATR) of compound **4t**.

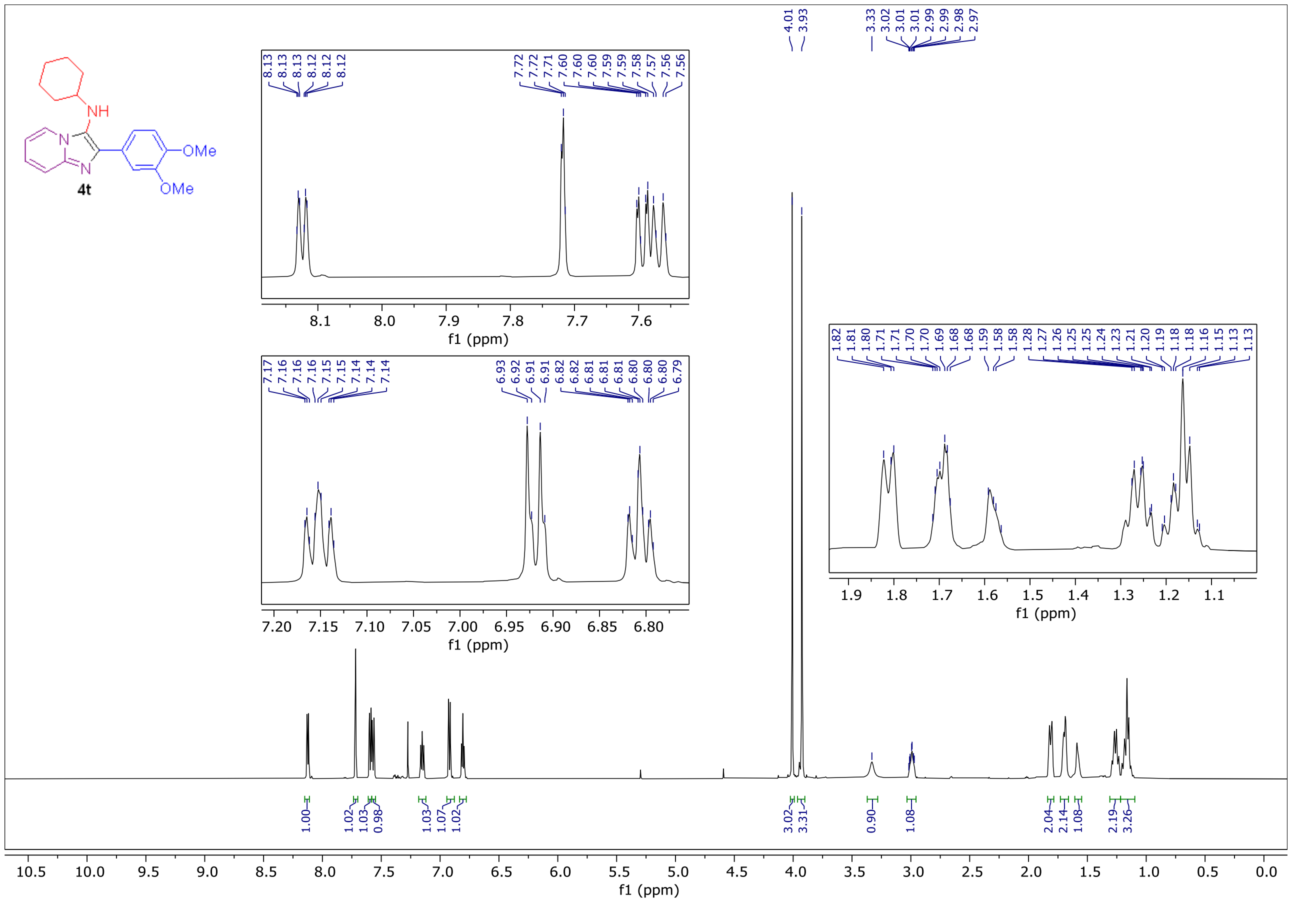

S 78. ^{1}H NMR spectrum (600 MHz, $CDCl_3$) of compound **4t**.

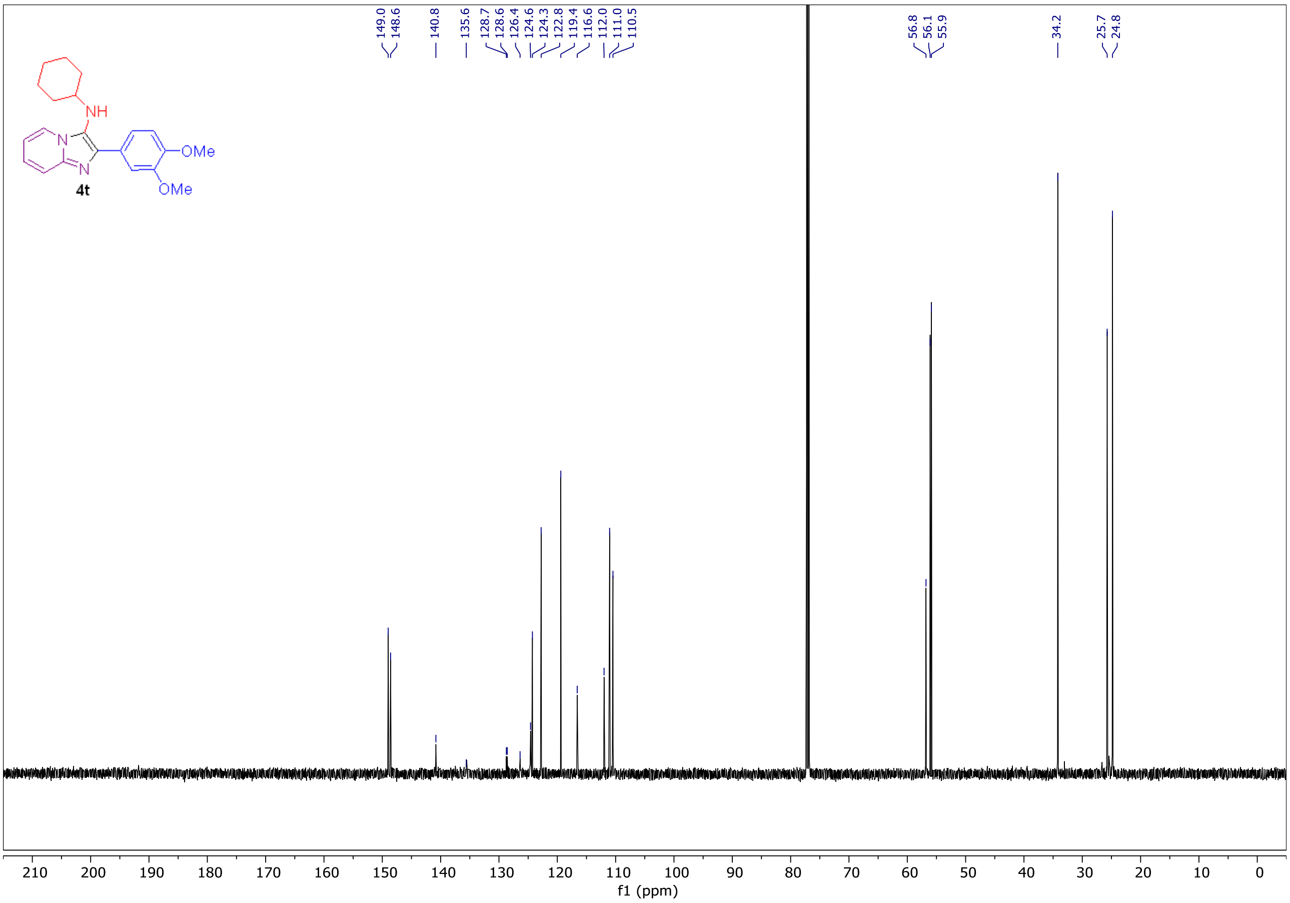

S 79. ^{13}C NMR spectrum (151 MHz, $CDCl_3$) of compound **4t**.

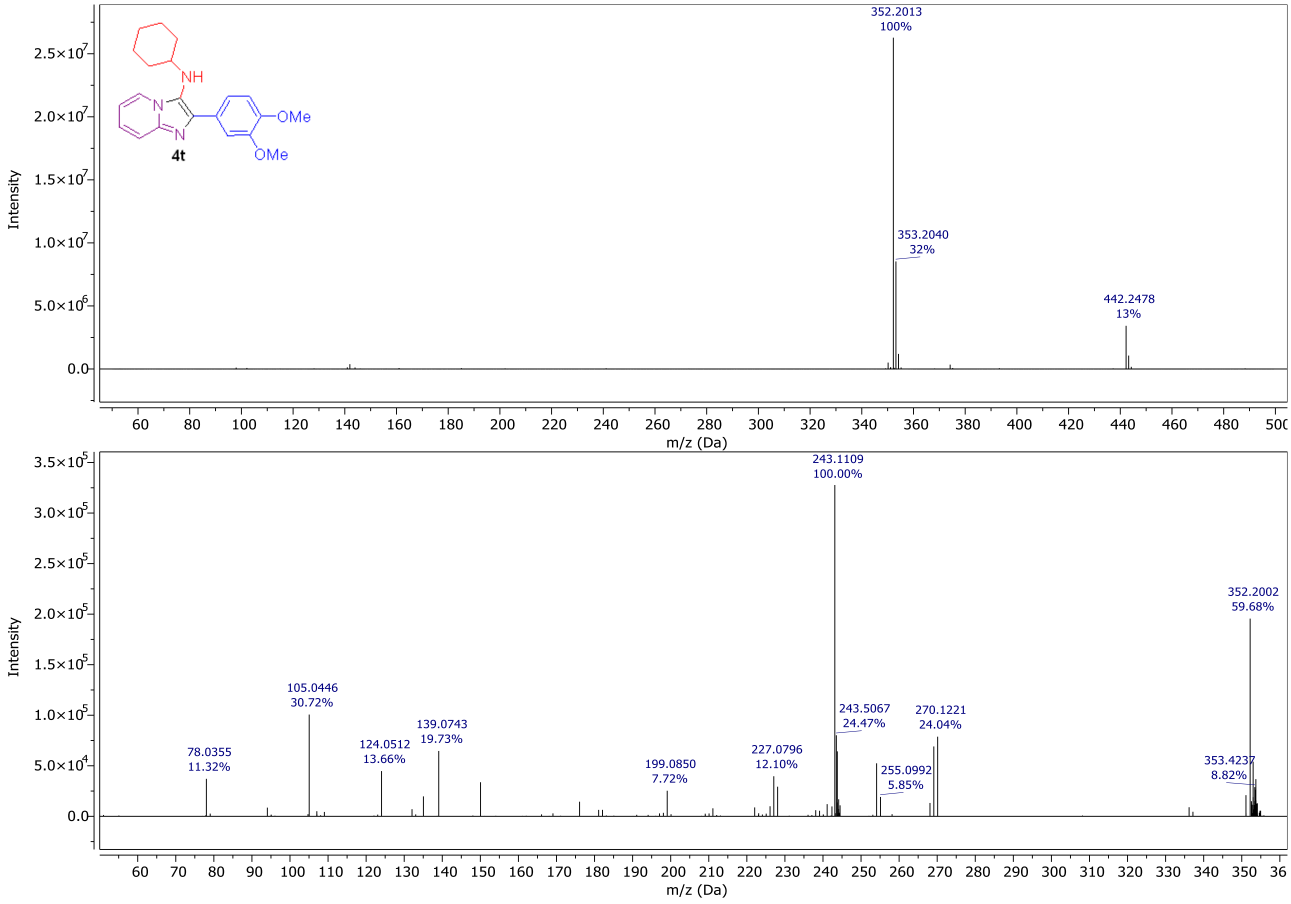

S 80. HRMS (ESI-QTOF) of compound **4t** and HRMS/MS for $[M+H]^+$.

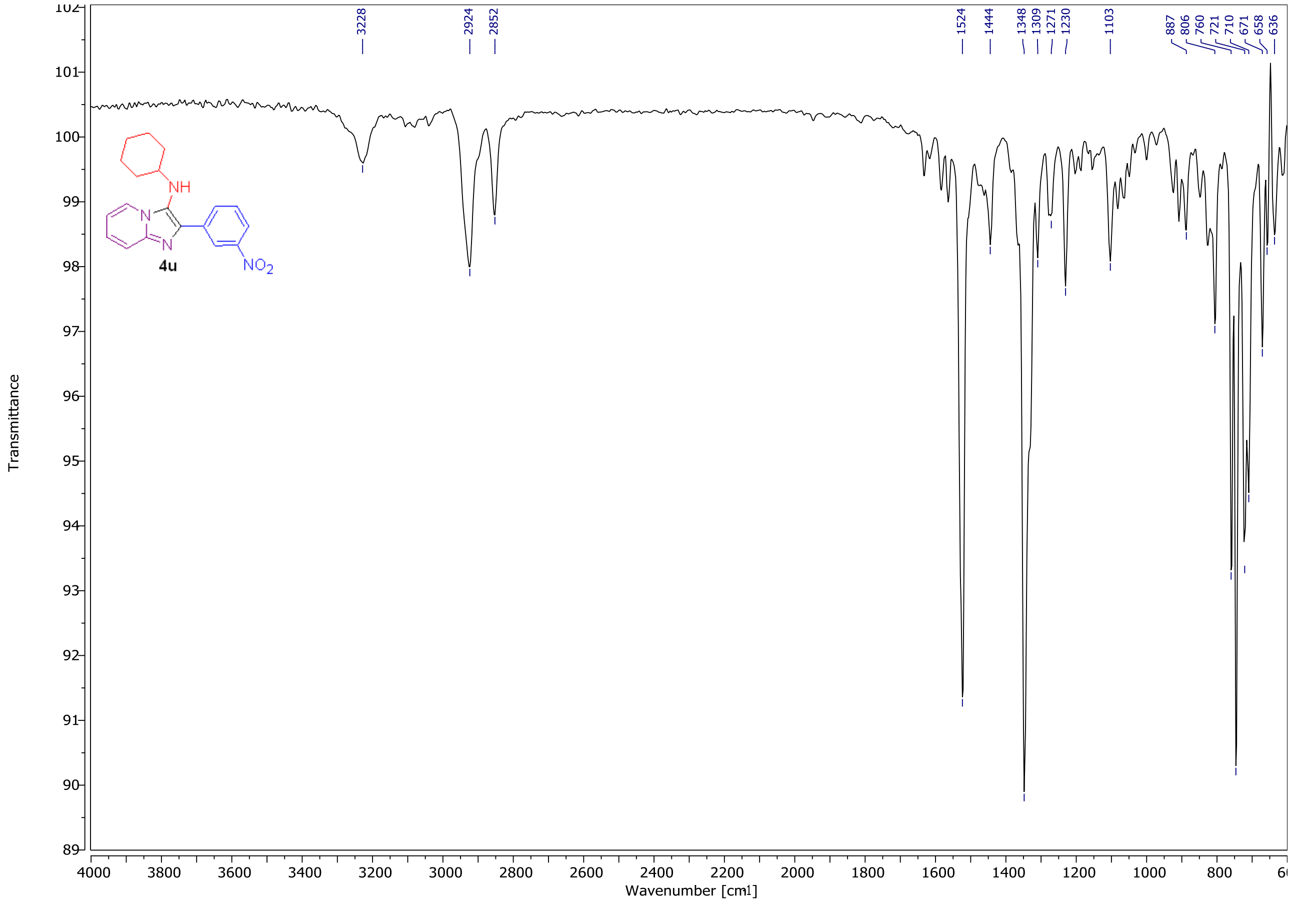

S 81. FT-IR (ATR) of compound **4u**.

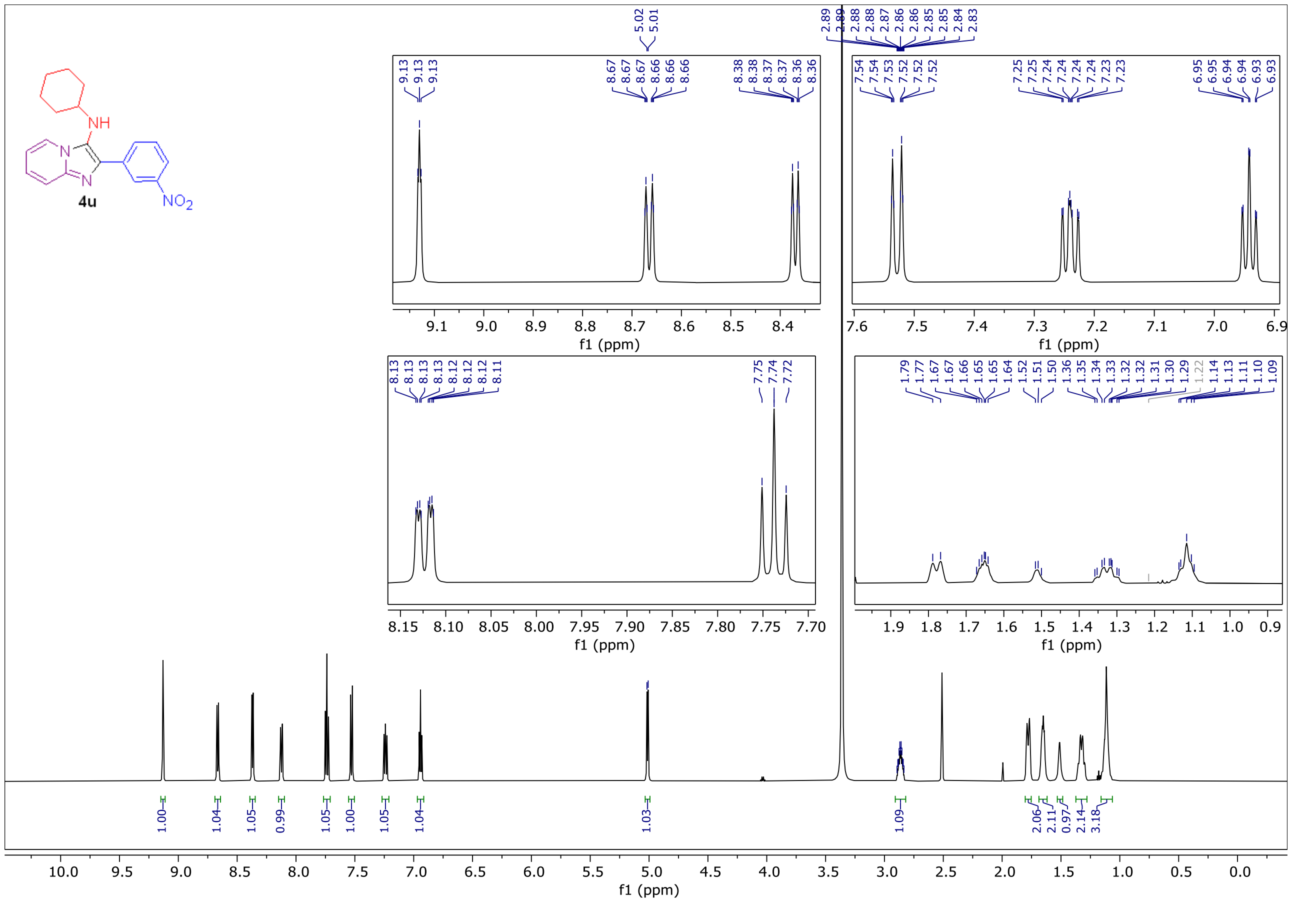

S 82. ^{1}H NMR spectrum (600 MHz, DMSO-d_6) of compound **4u**.

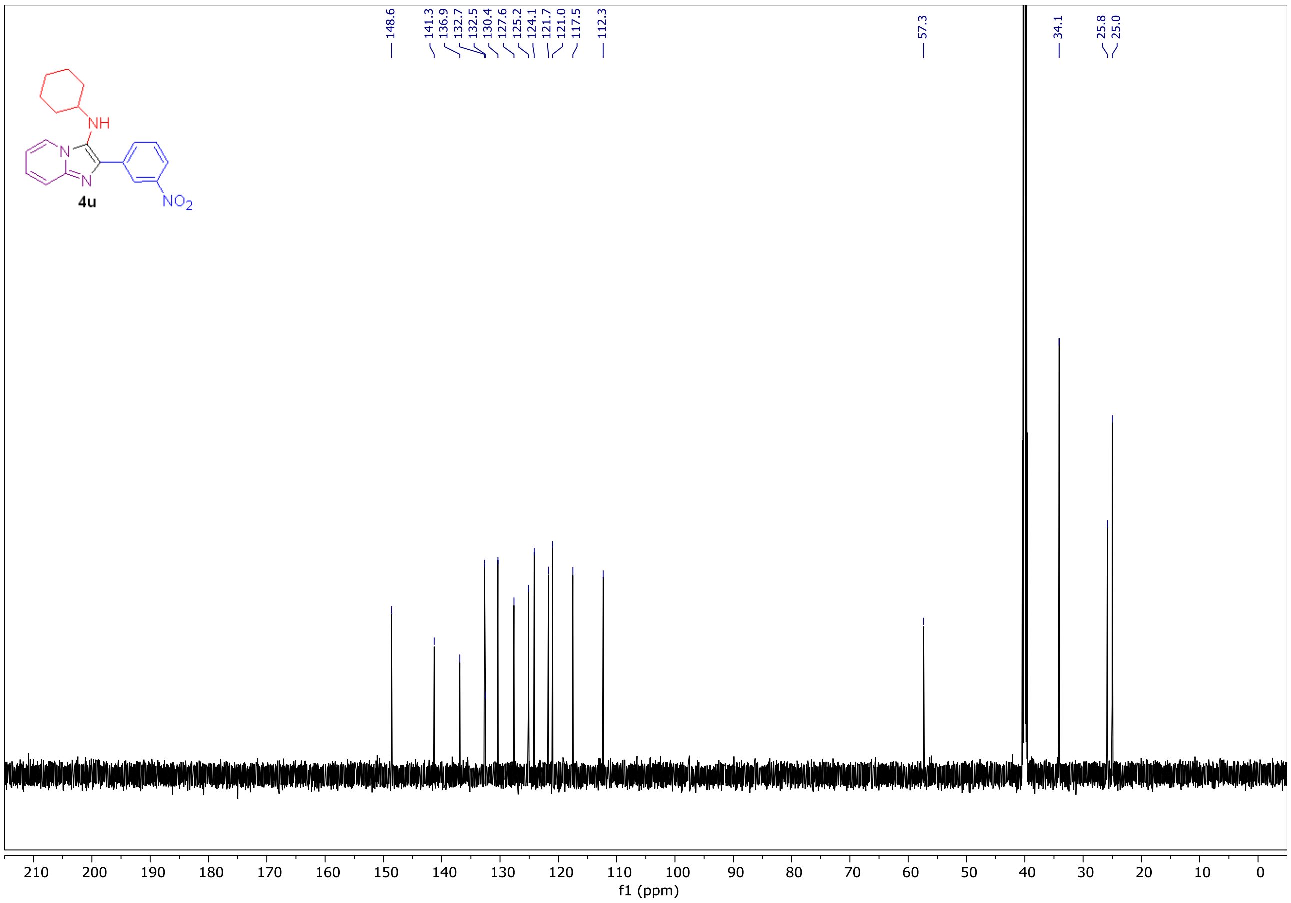

S 83. ^{13}C NMR spectrum (151 MHz, DMSO-d_6) of compound **4u**.

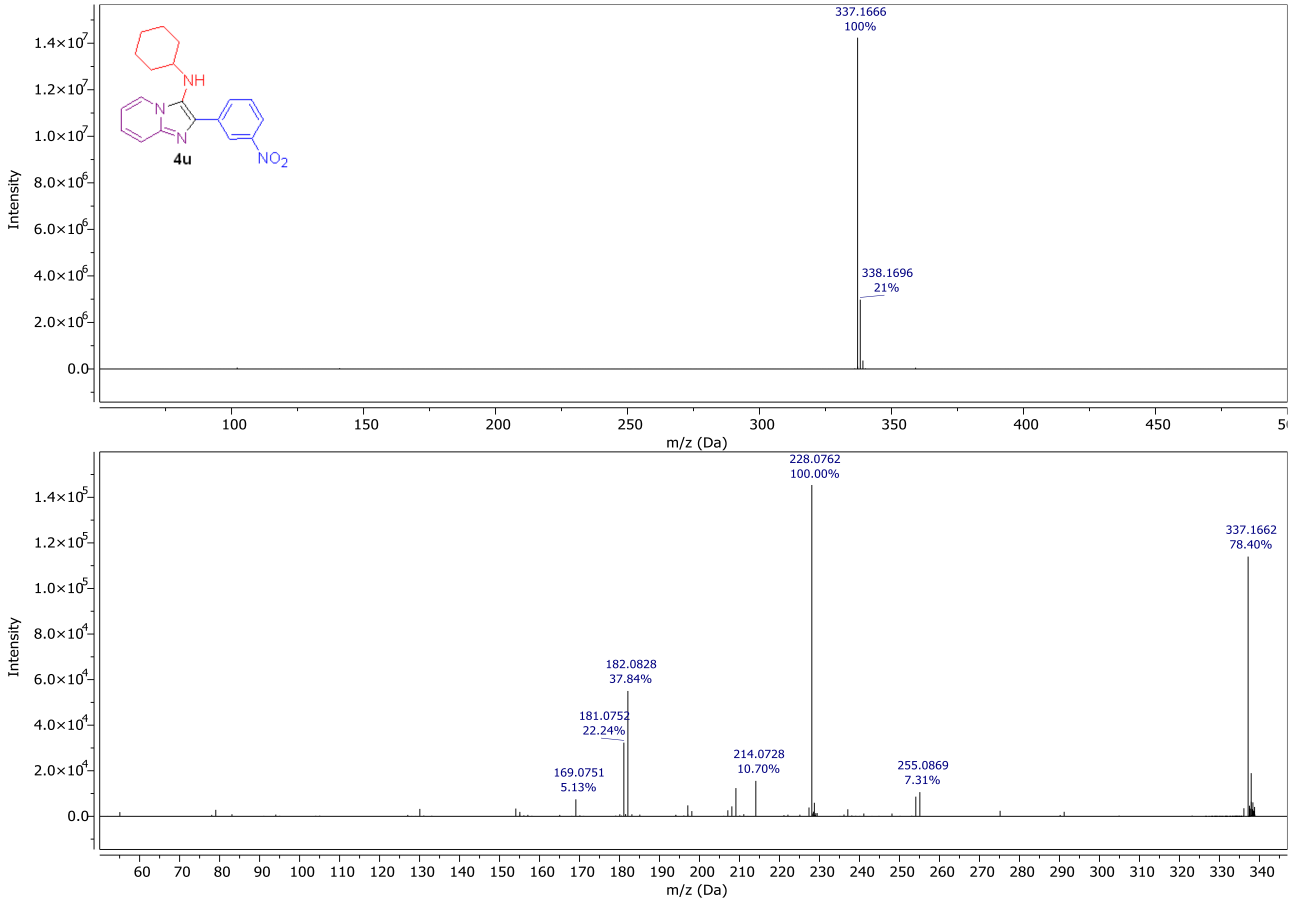

S 84. HRMS (ESI-QTOF) of compound **4t** and HRMS/MS for [M+H]$^+$.

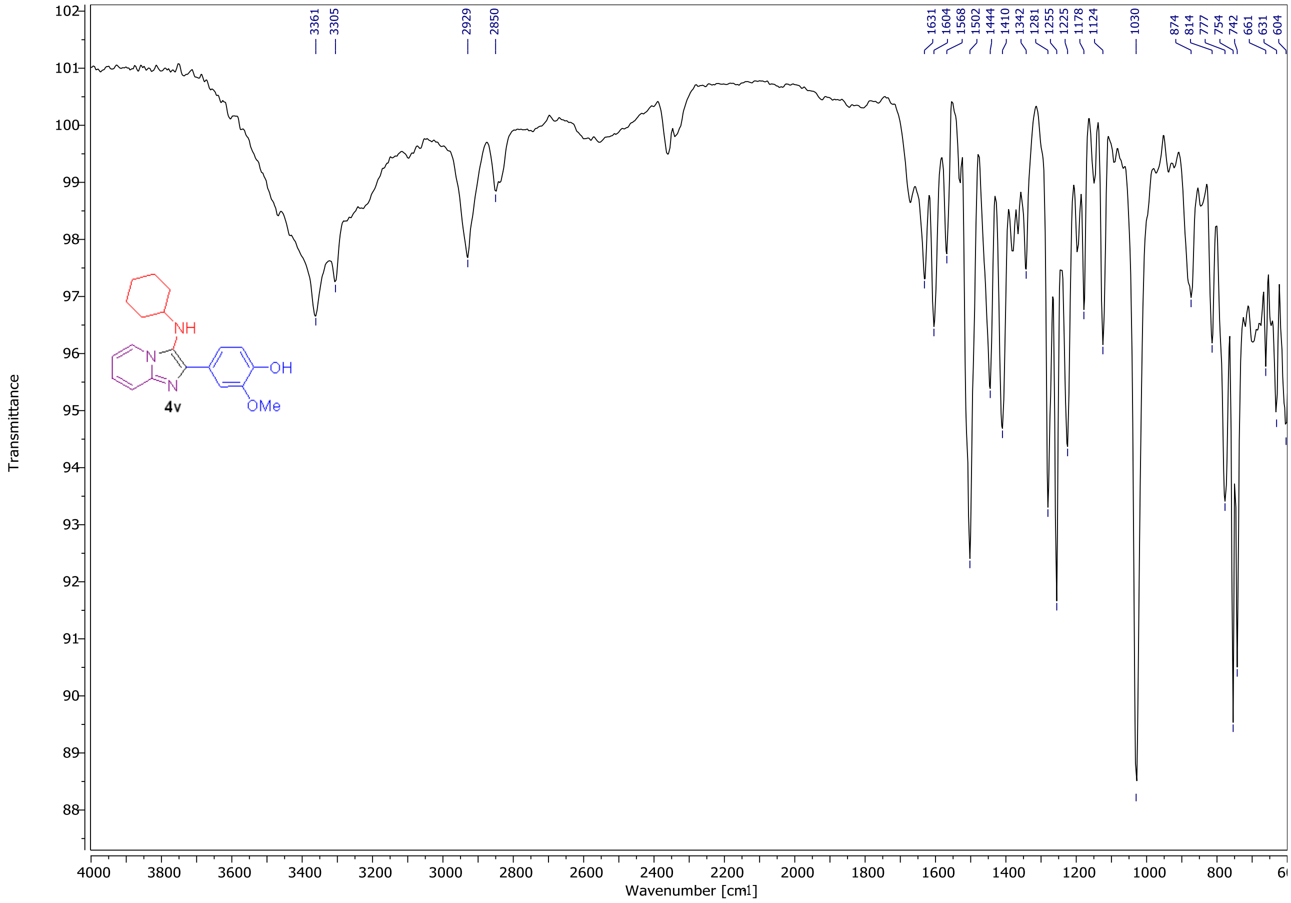

S 85. FT-IR (ATR) of compound **4v**.

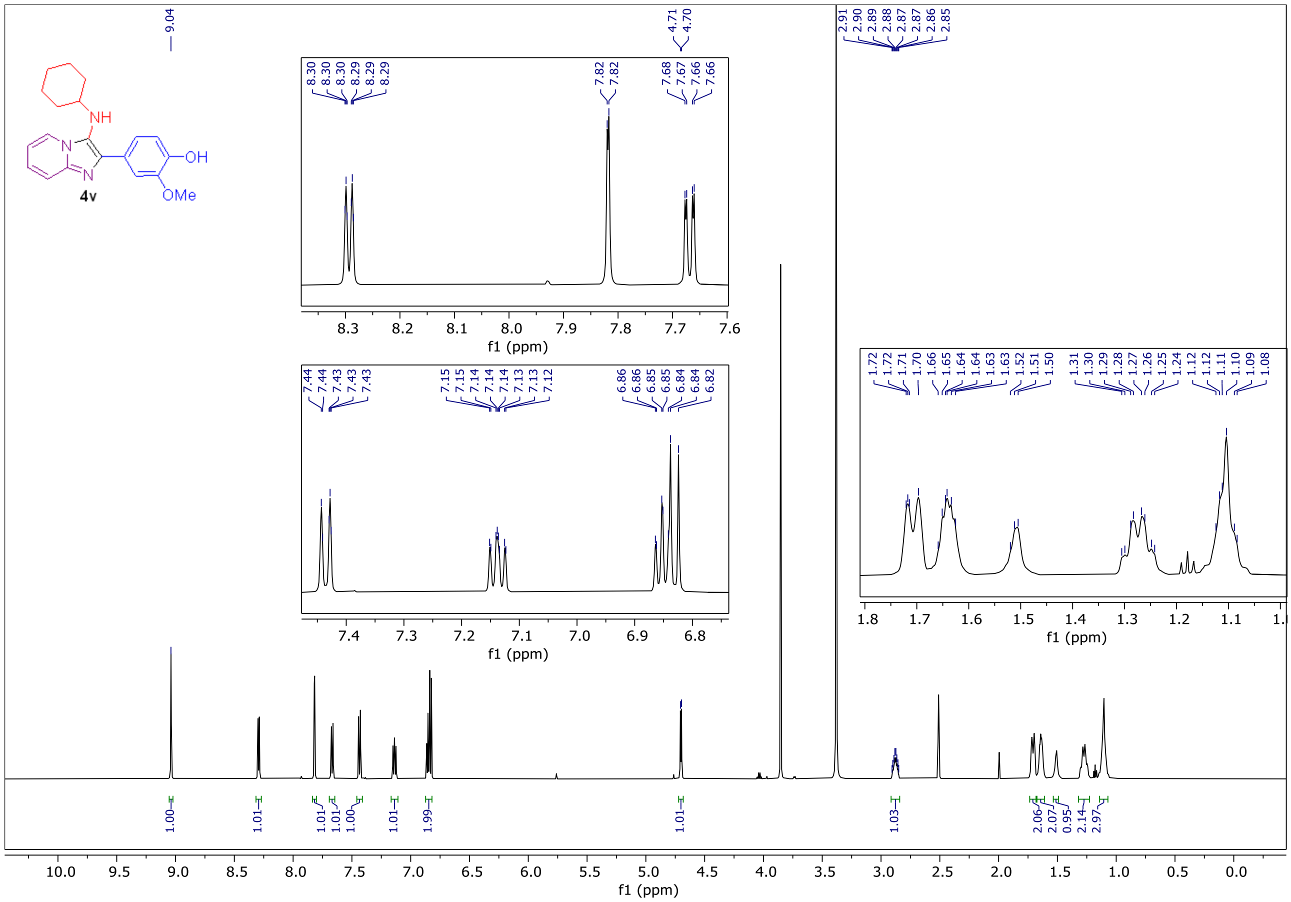

S 86. ^{1}H NMR spectrum (600 MHz, DMSO-d_6) of compound **4v**.

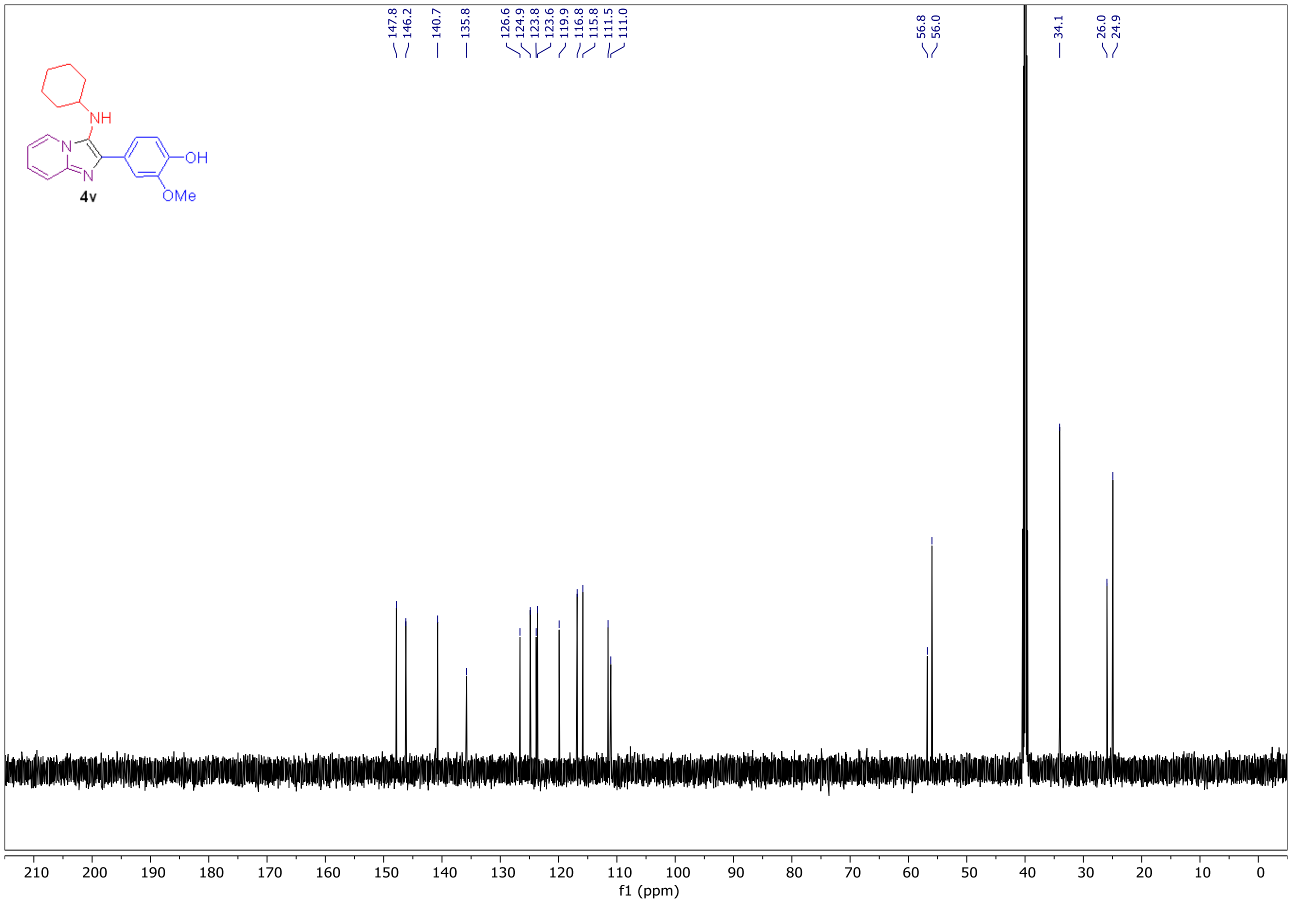

S 87. ^{13}C NMR spectrum (151 MHz, DMSO-d_6) of compound **4v**.

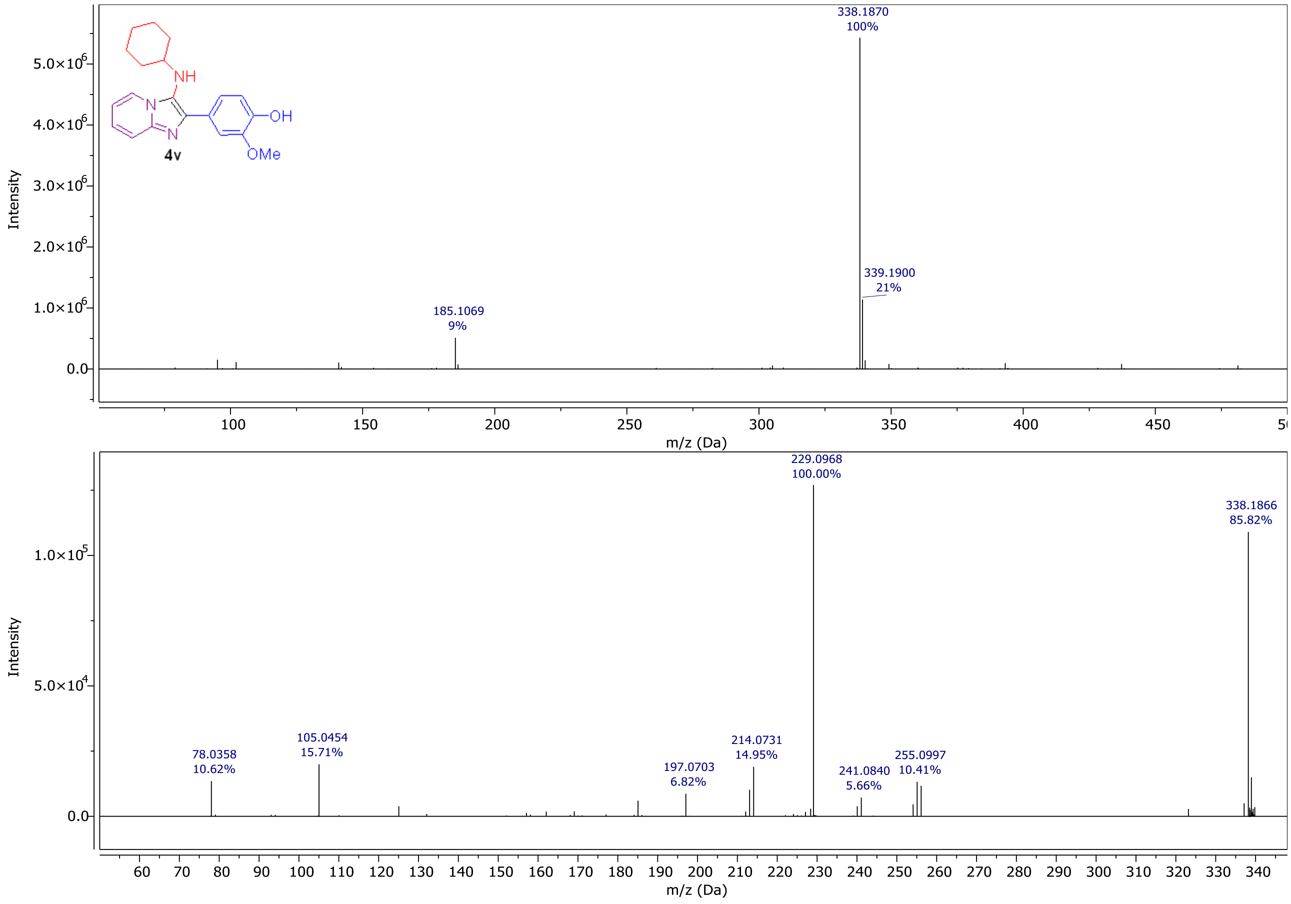

S 88. HRMS (ESI-QTOF) of compound **4v** and HRMS/MS for [M+H]$^+$.

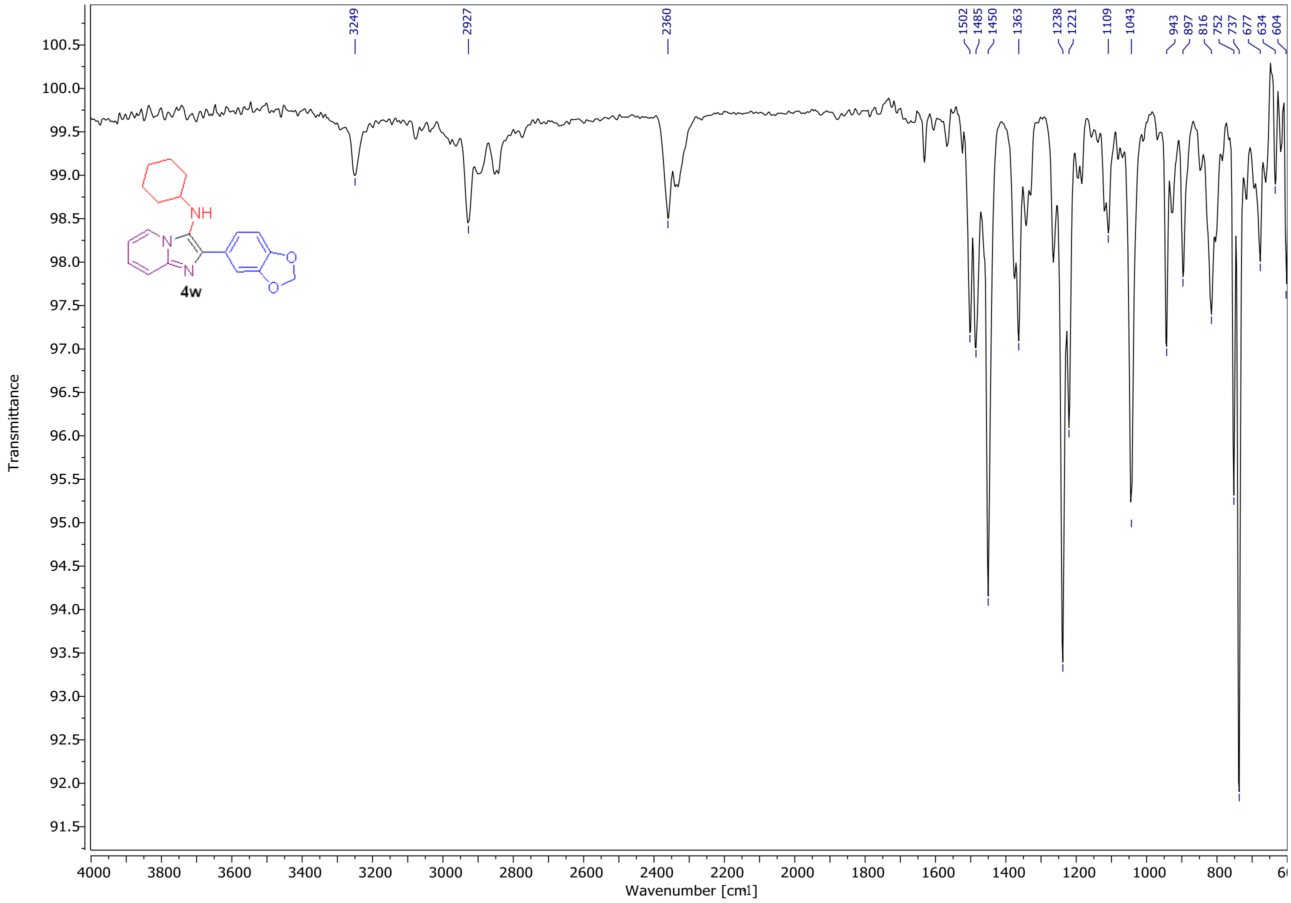

S 89. FT-IR (ATR) of compound **4w**.

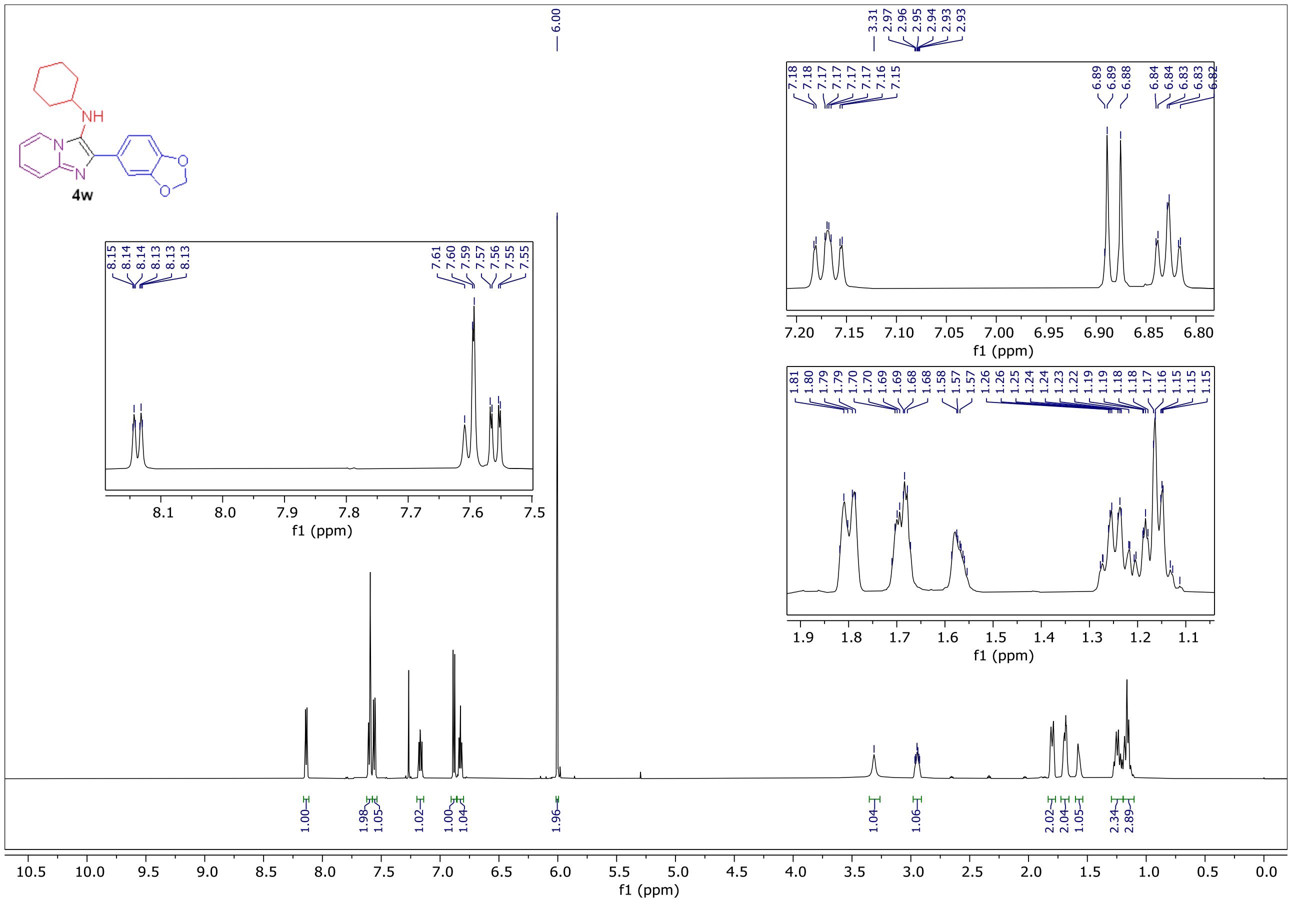

S 90. ^{1}H NMR spectrum (600 MHz, $CDCl_3$) of compound **4w**.

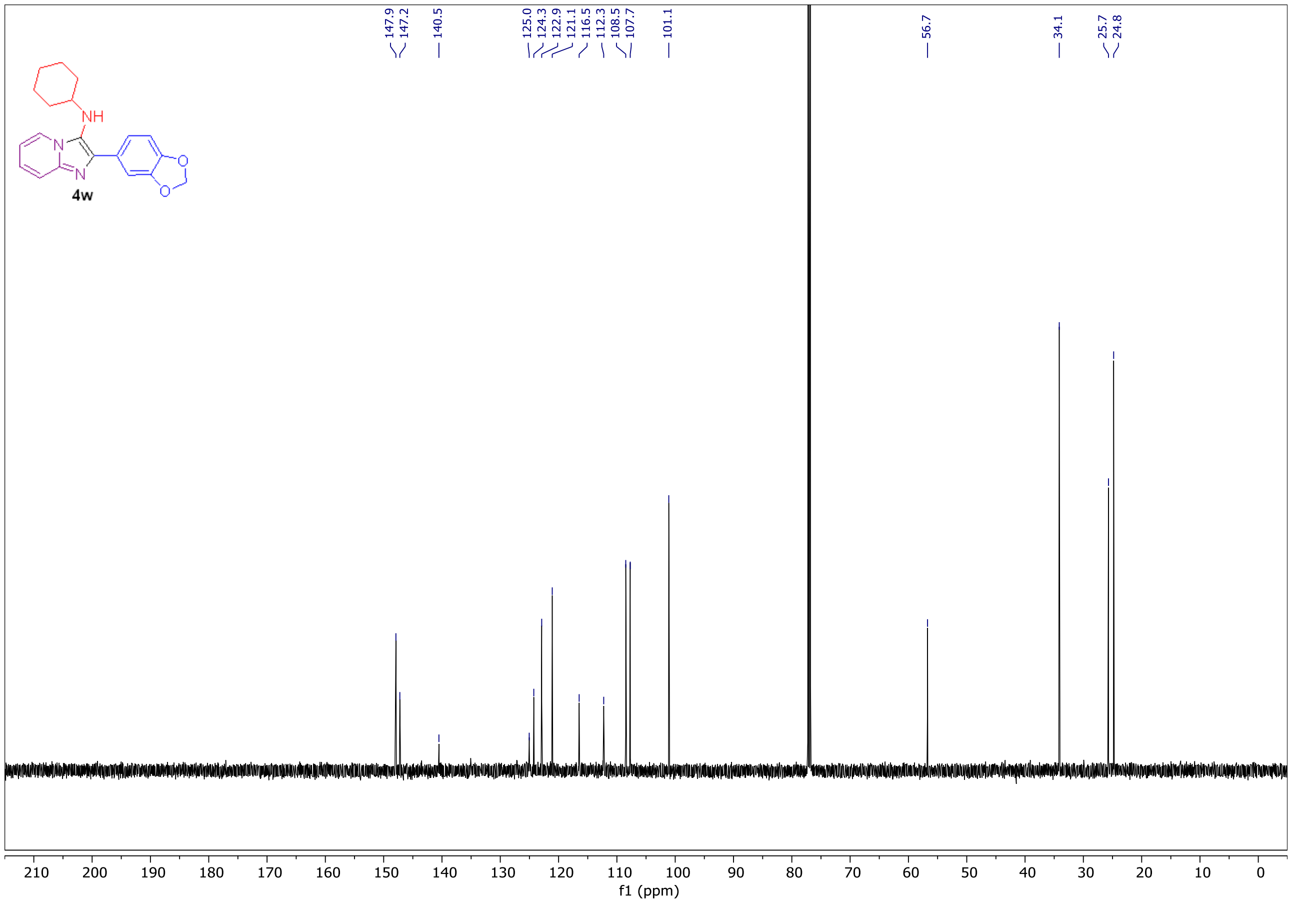

S 91. ^{13}C NMR spectrum (151 MHz, $CDCl_3$) of compound **4w**.

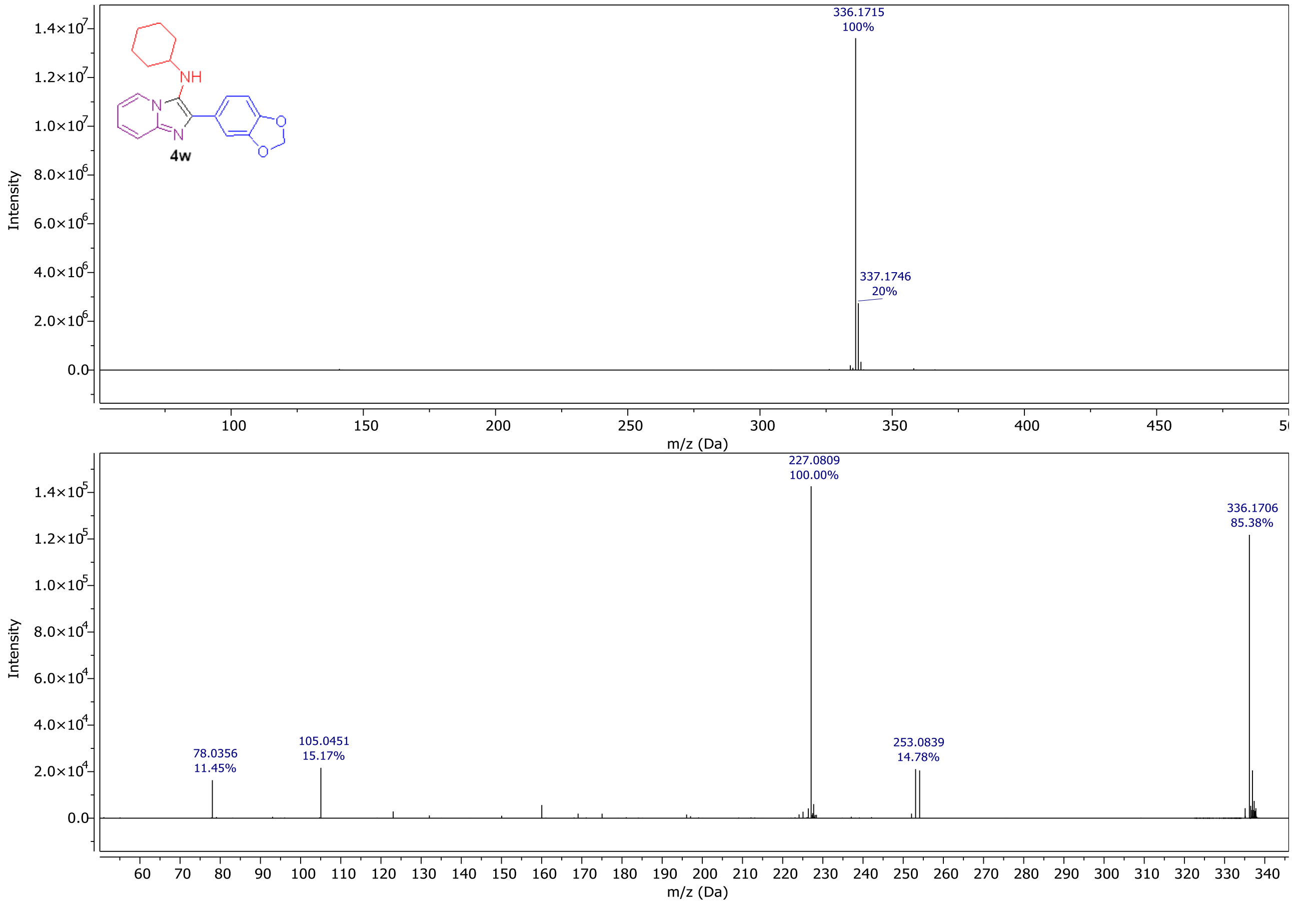

S 92. HRMS (ESI-QTOF) of compound **4w** and HRMS/MS for $[M+H]^+$.

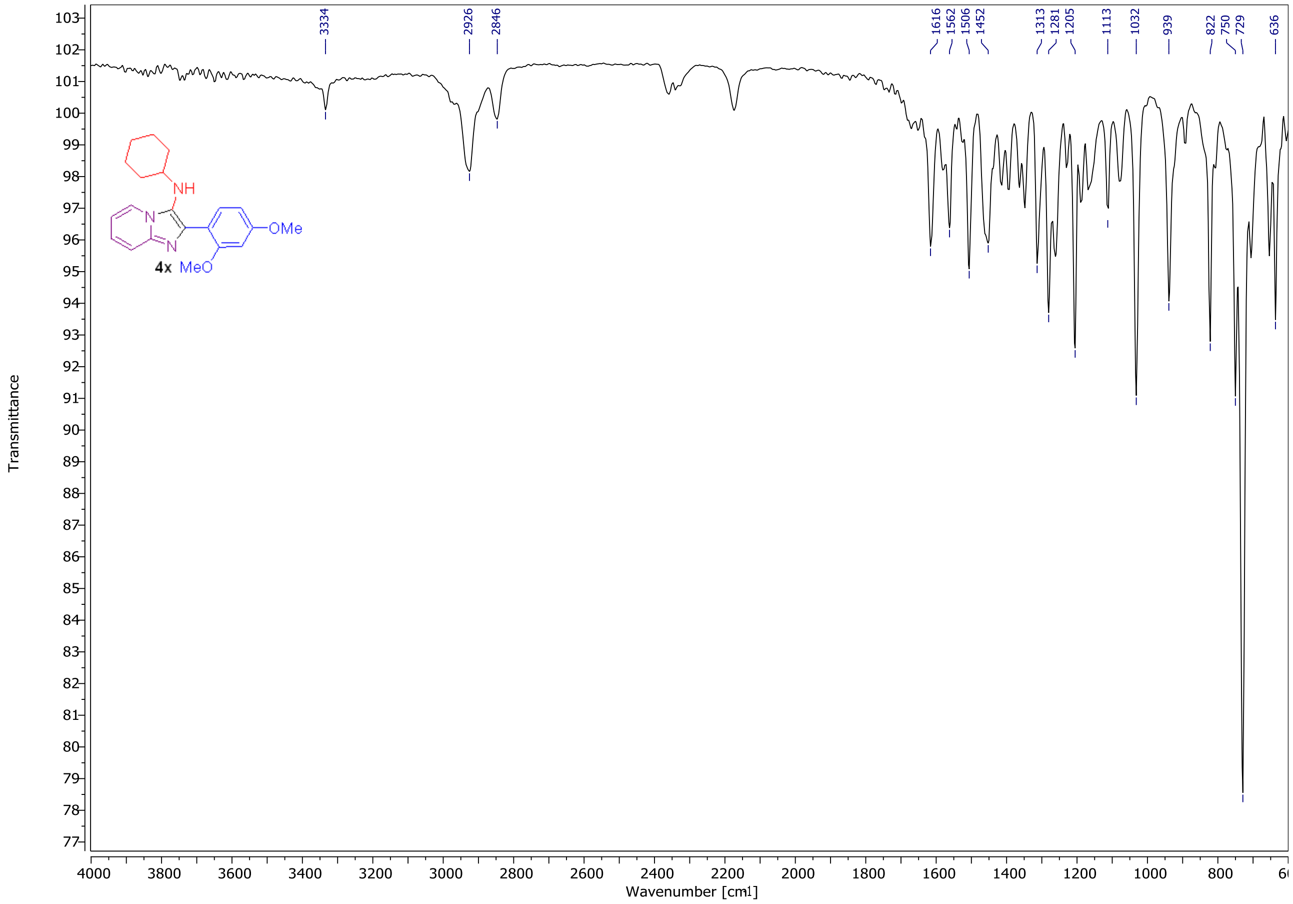

S 93. FT-IR (ATR) of compound **4x**.

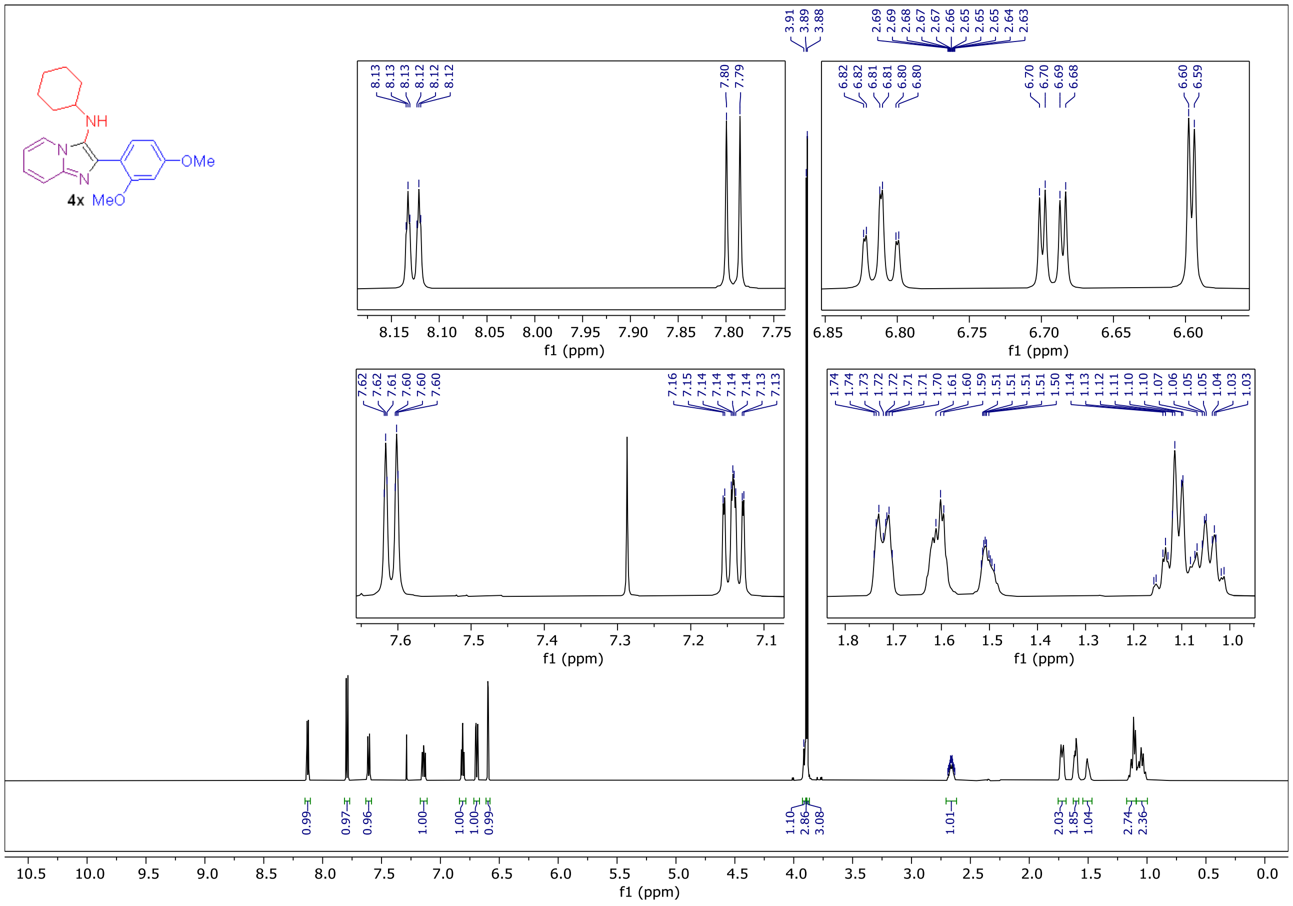

S 94. ^{1}H NMR spectrum (600 MHz, $CDCl_3$) of compound **4x**.

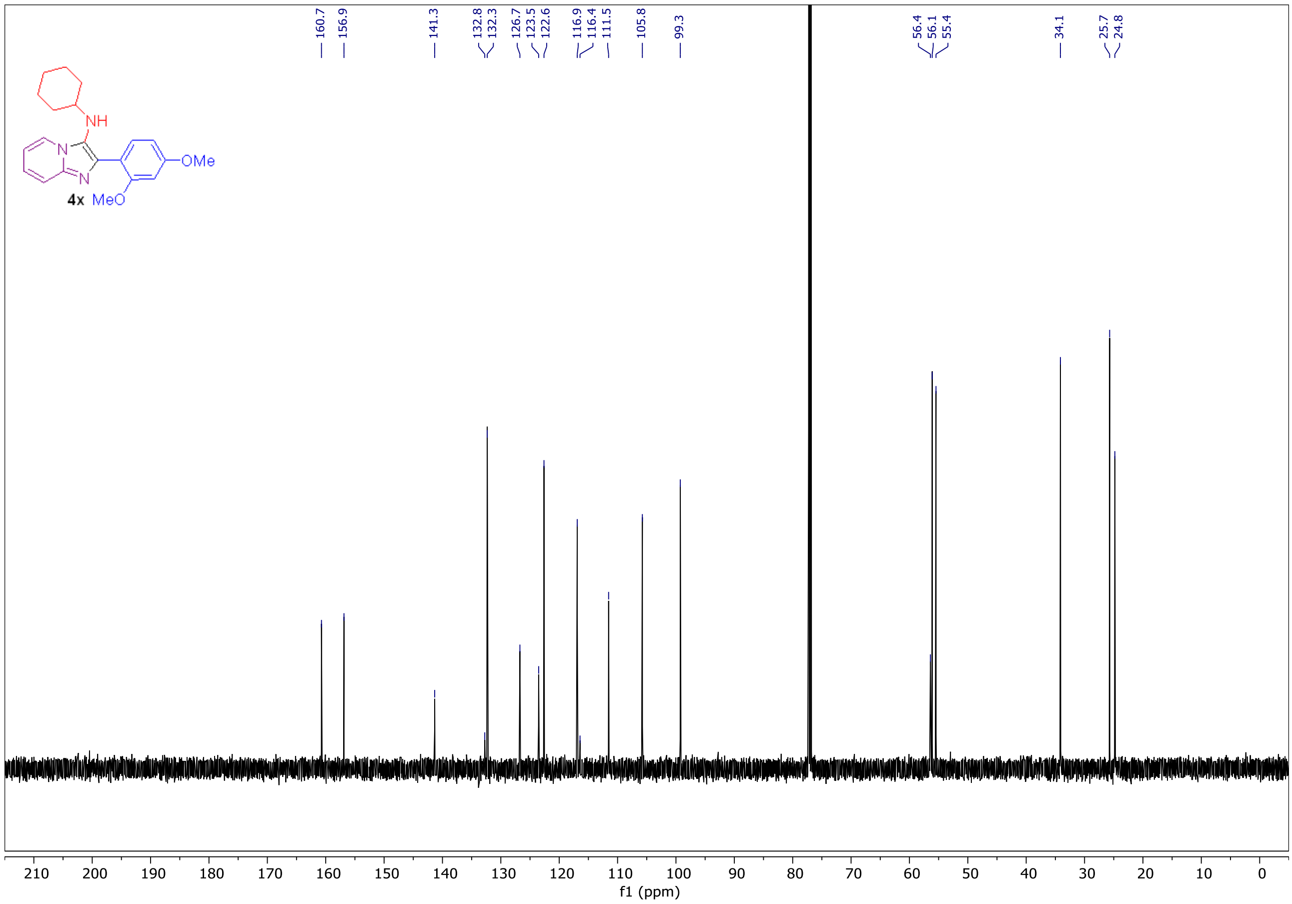

S 95. ^{13}C NMR spectrum (151 MHz, $CDCl_3$) of compound **4x**.

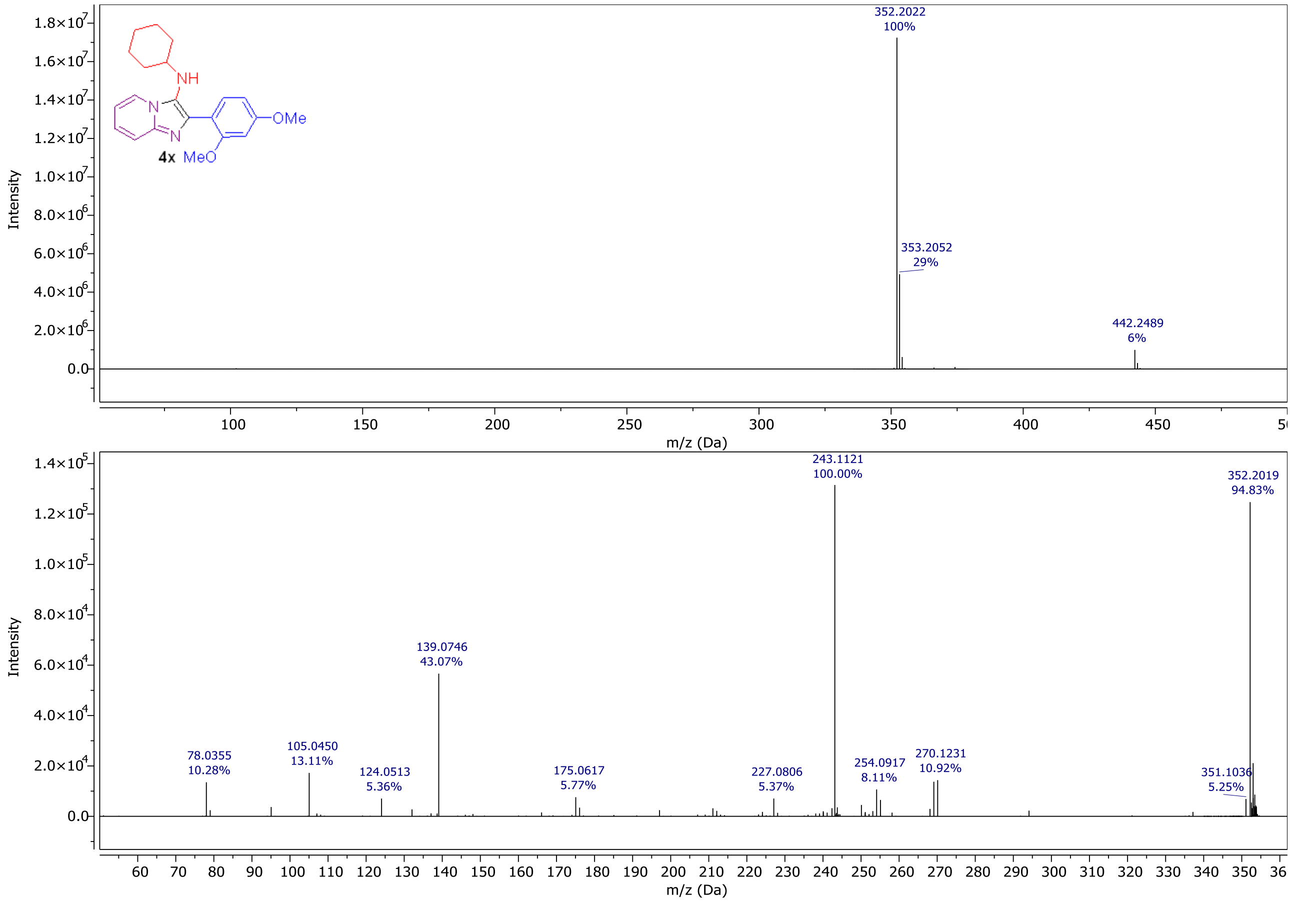

S 96. HRMS (ESI-QTOF) of compound **4x** and HRMS/MS for [M+H]$^+$.

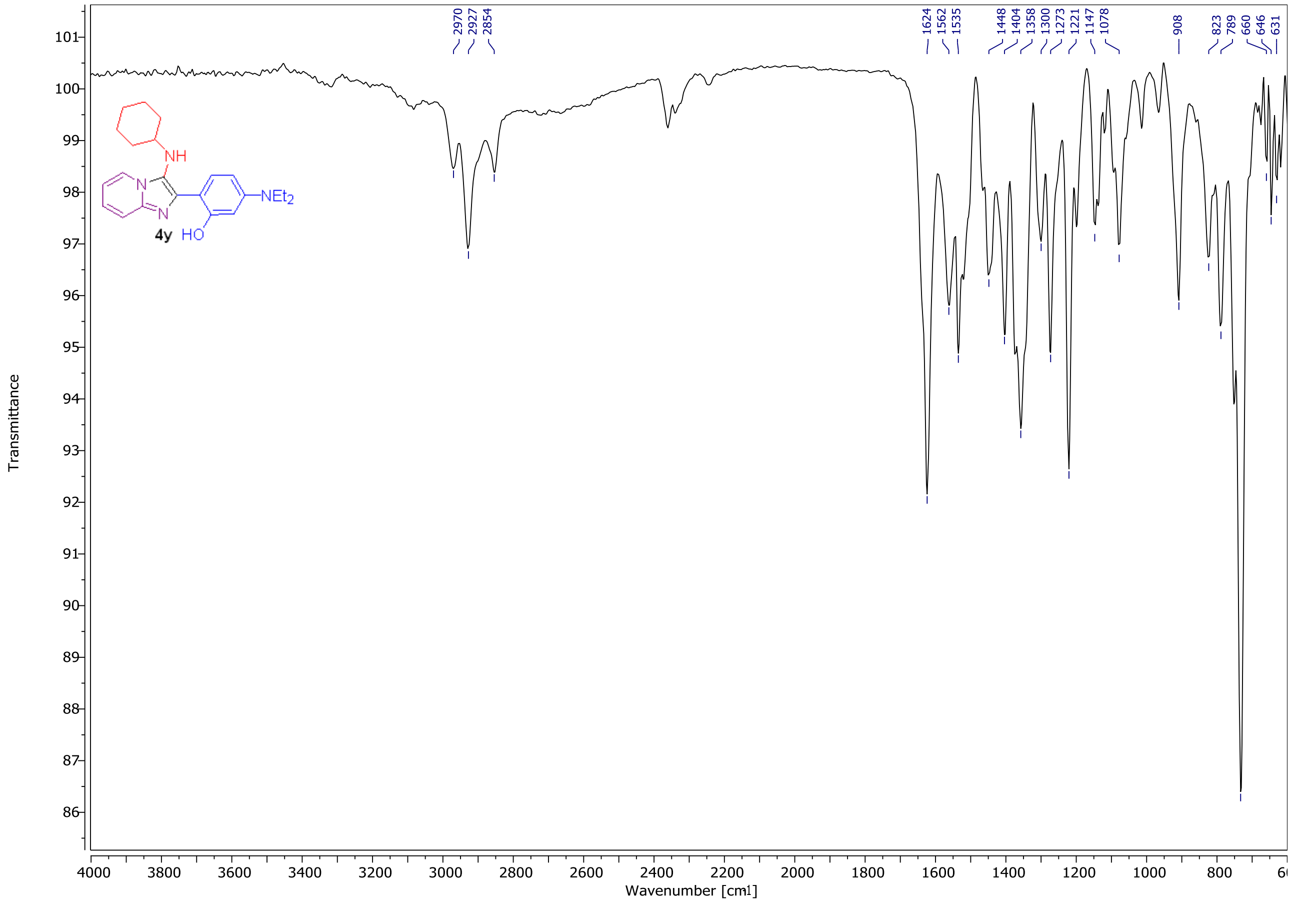

S 97. FT-IR (ATR) of compound **4y**.

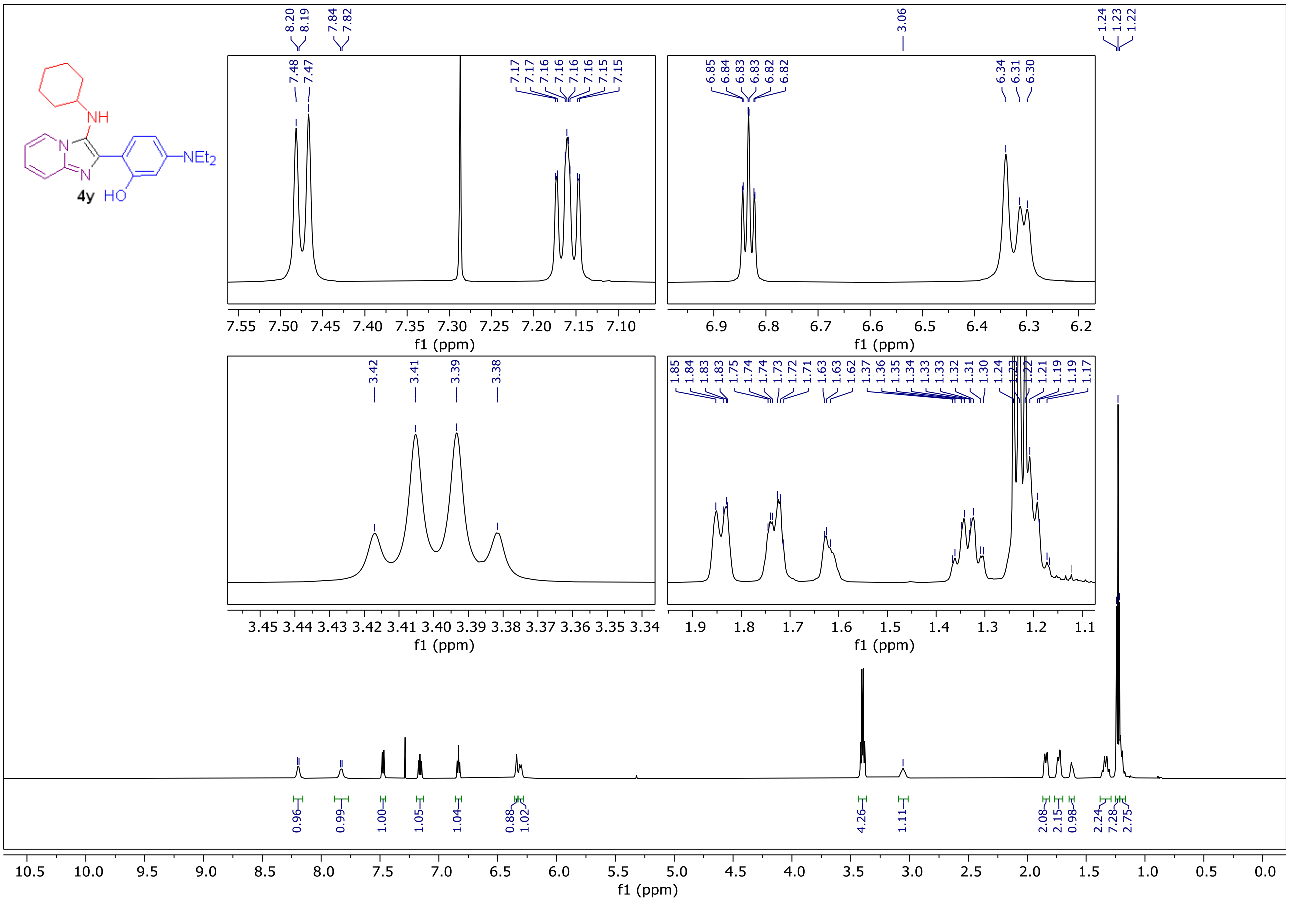

S 98. ^{1}H NMR spectrum (600 MHz, $CDCl_3$) of compound **4y**.

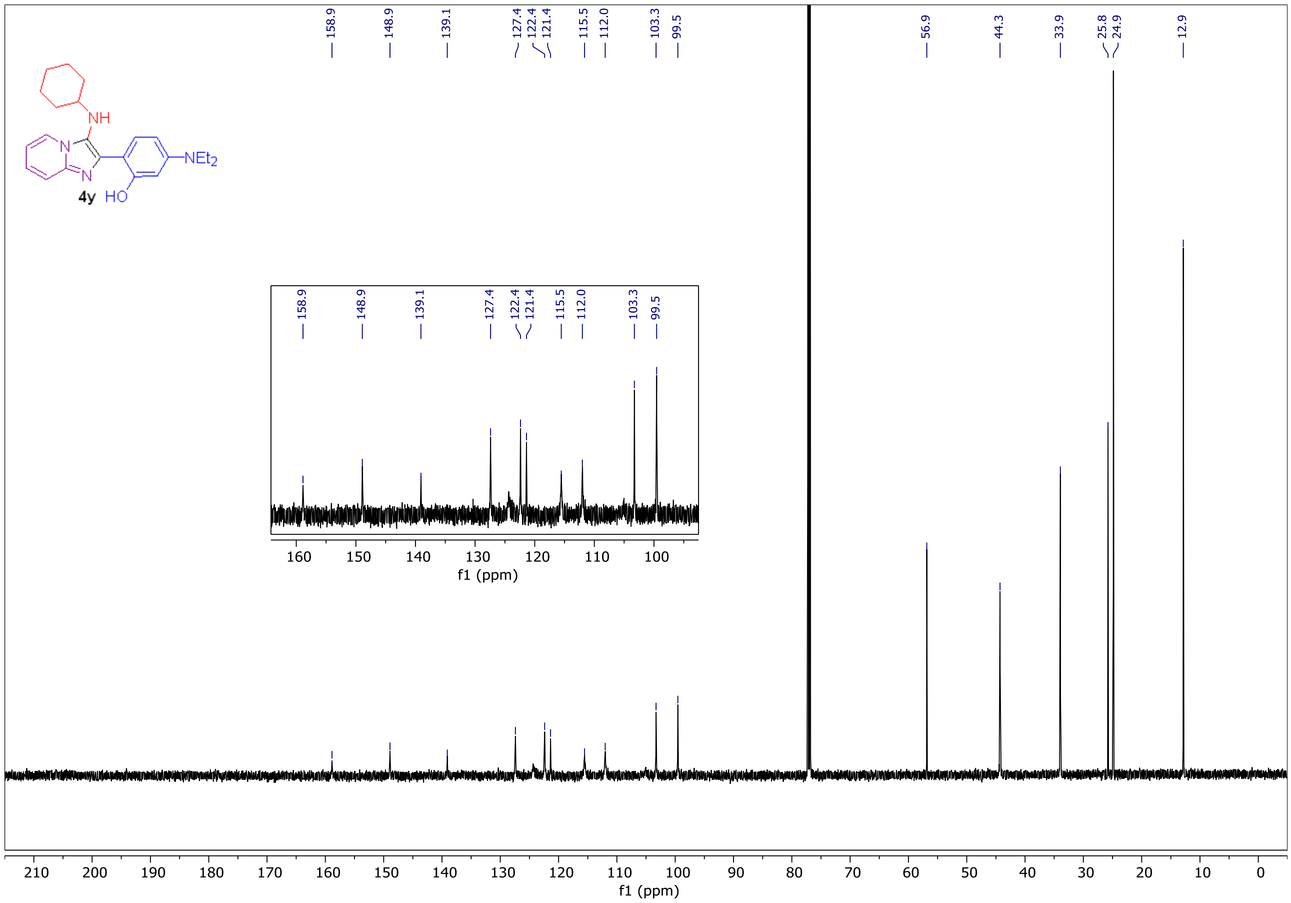

S 99. ^{13}C NMR spectrum (151 MHz, $CDCl_3$) of compound **4y**.

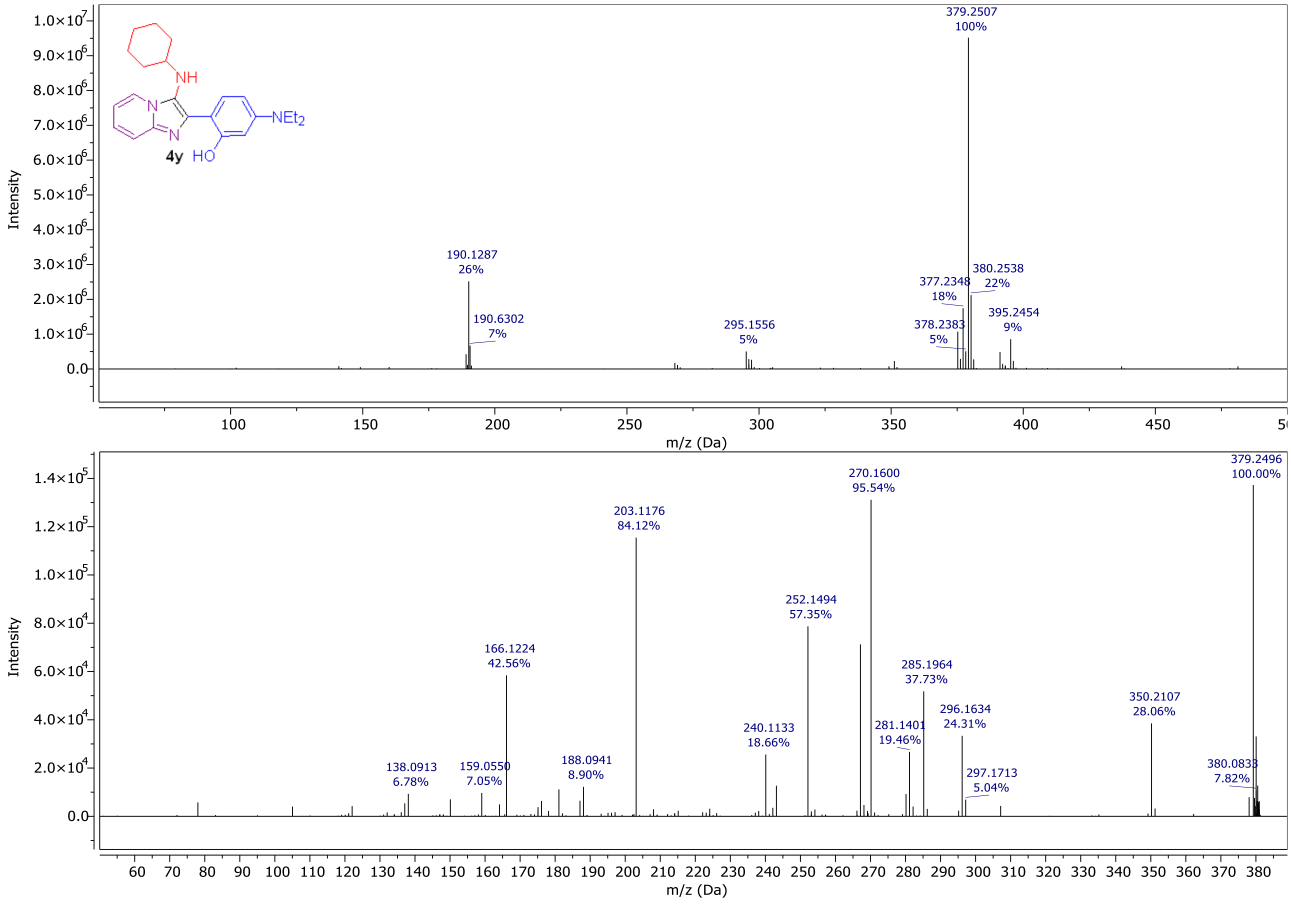

S 100. HRMS (ESI-QTOF) of compound **4y** and HRMS/MS for $[M+H]^+$.

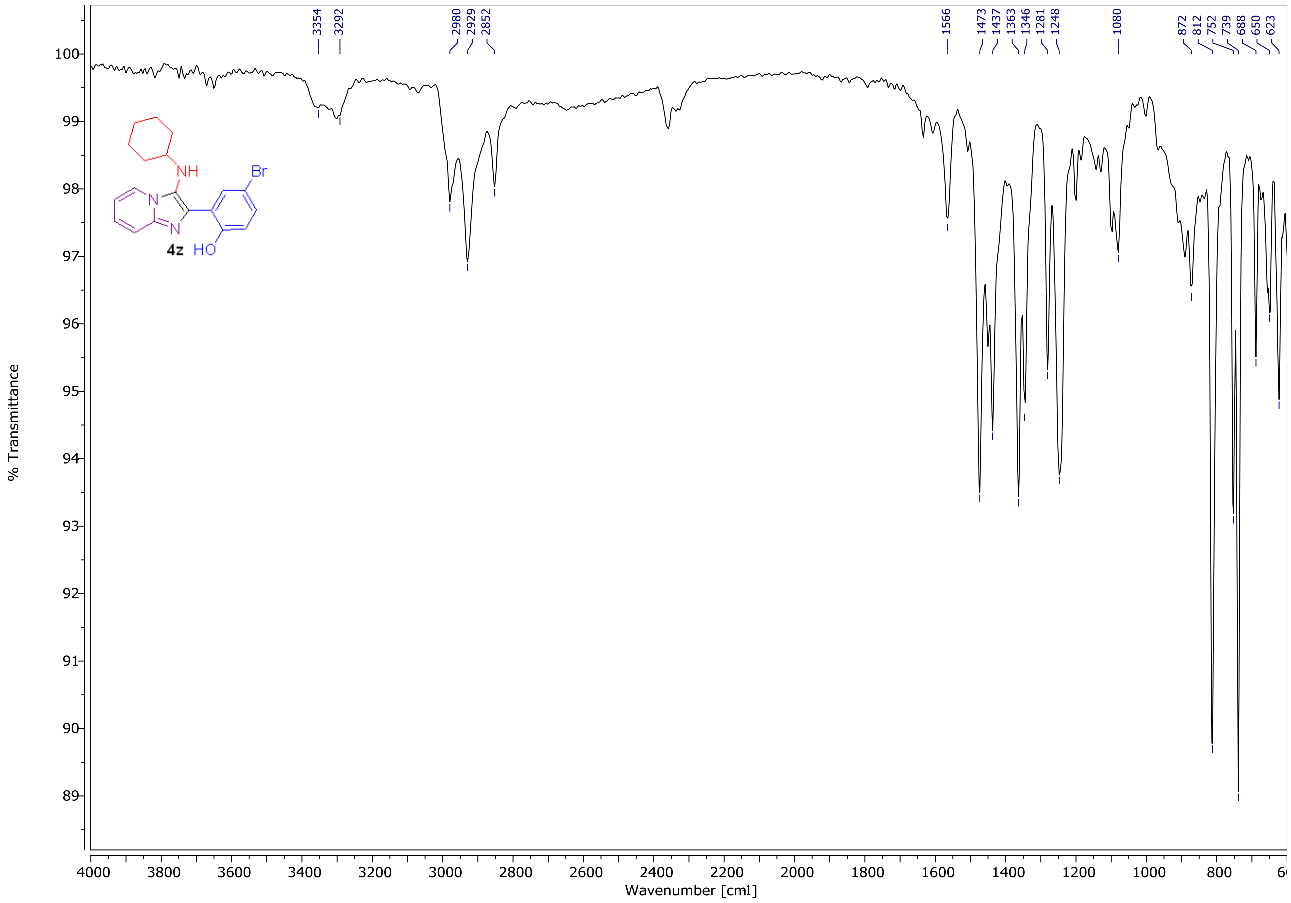

S 101. FT-IR (ATR) of compound **4z**.

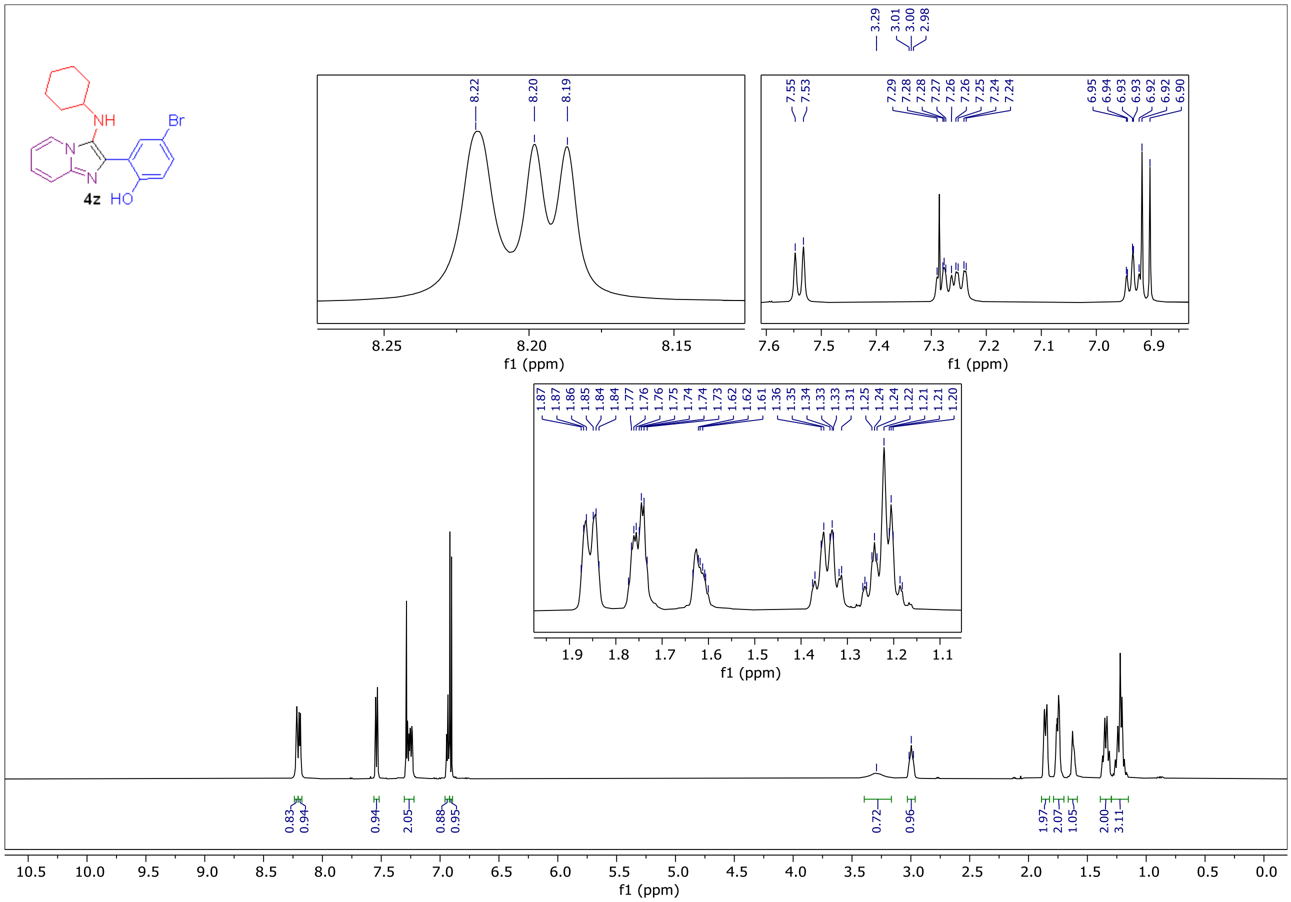

S 102. ^{1}H NMR spectrum (600 MHz, $CDCl_3$) of compound **4z**.

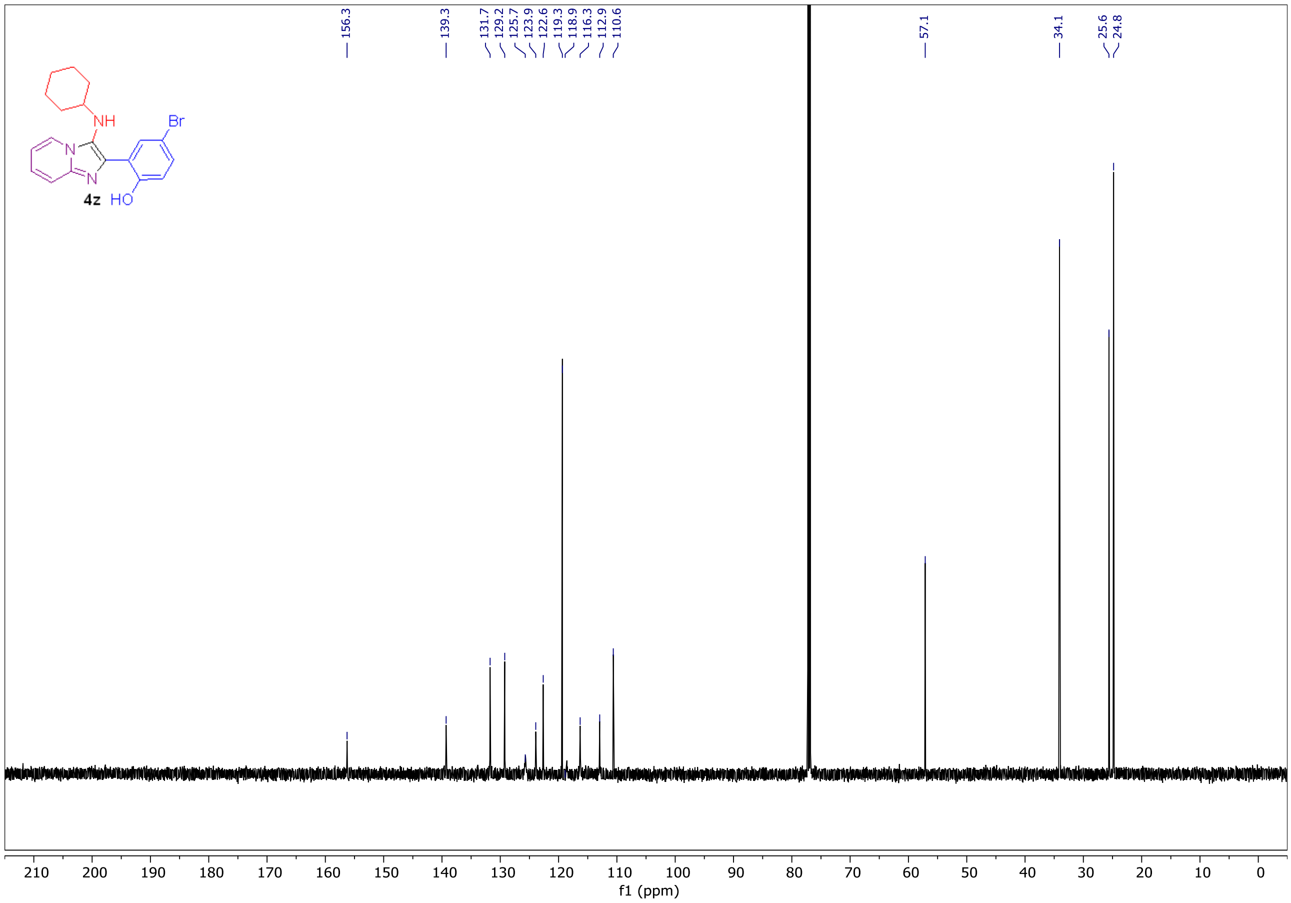

S 103. ^{13}C NMR spectrum (151 MHz, $CDCl_3$) of compound **4z**.

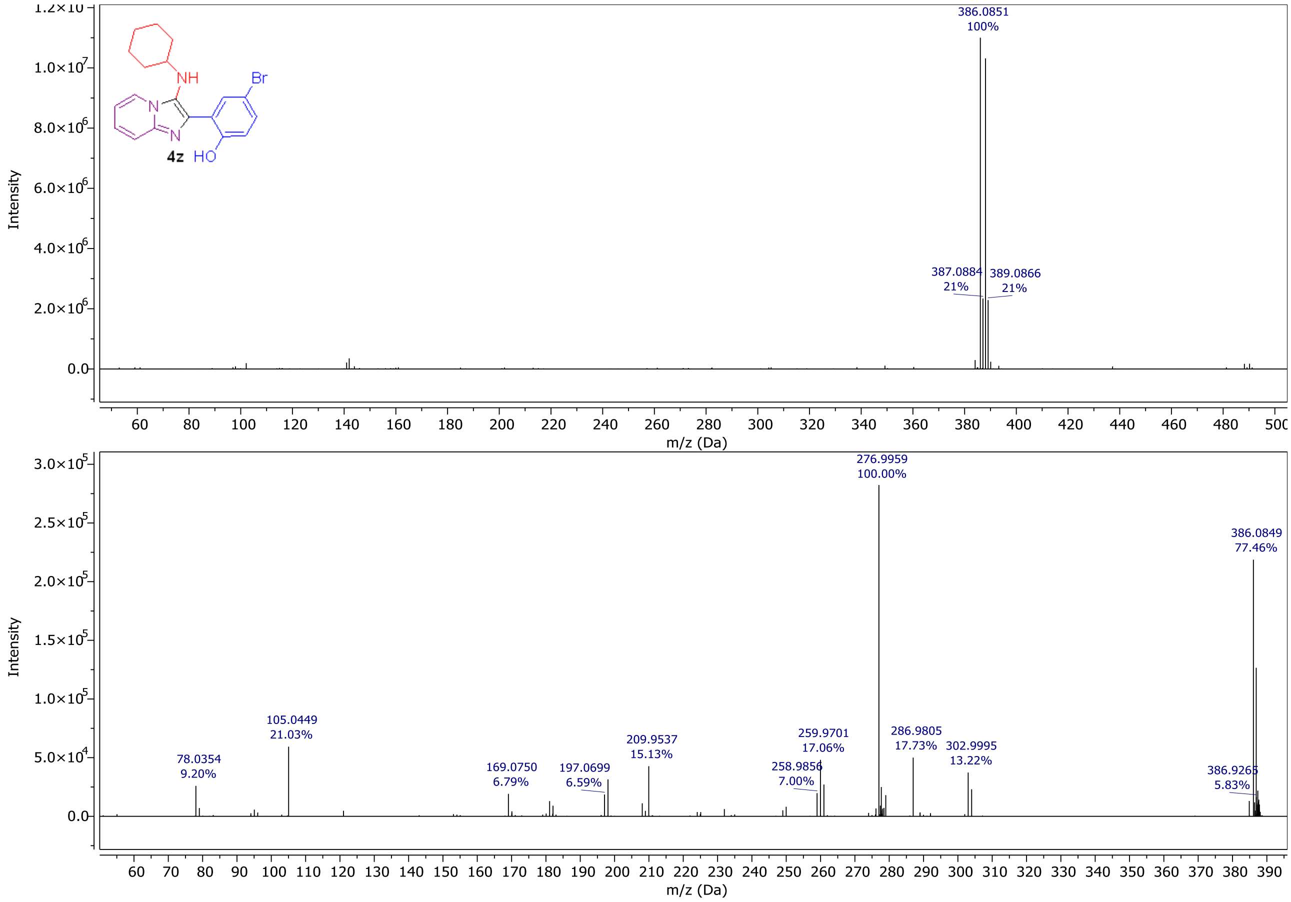

S 104. HRMS (ESI-QTOF) of compound **4z** and HRMS/MS for $[M+H]^+$.

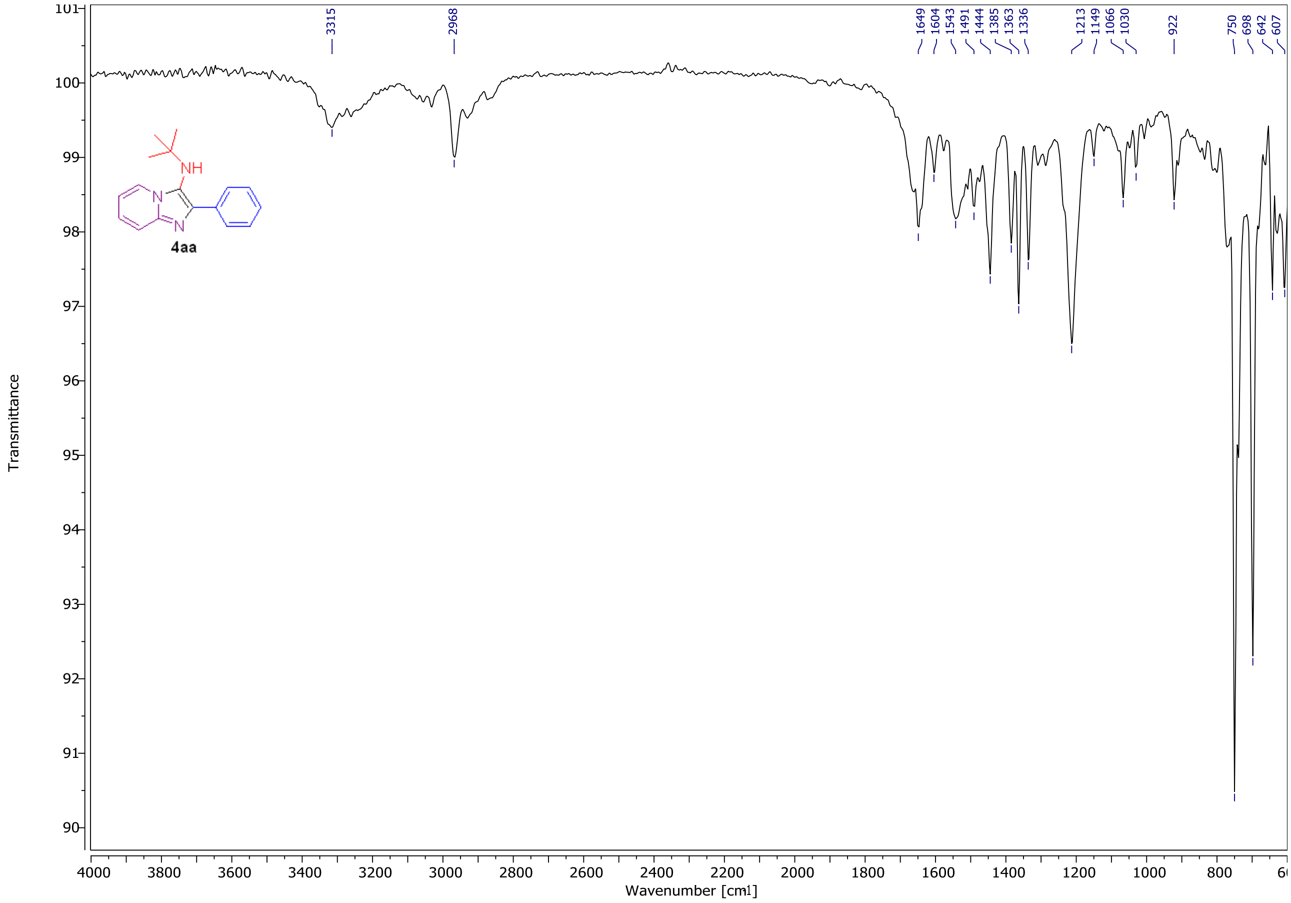

S 105. FT-IR (ATR) of compound **4aa**.

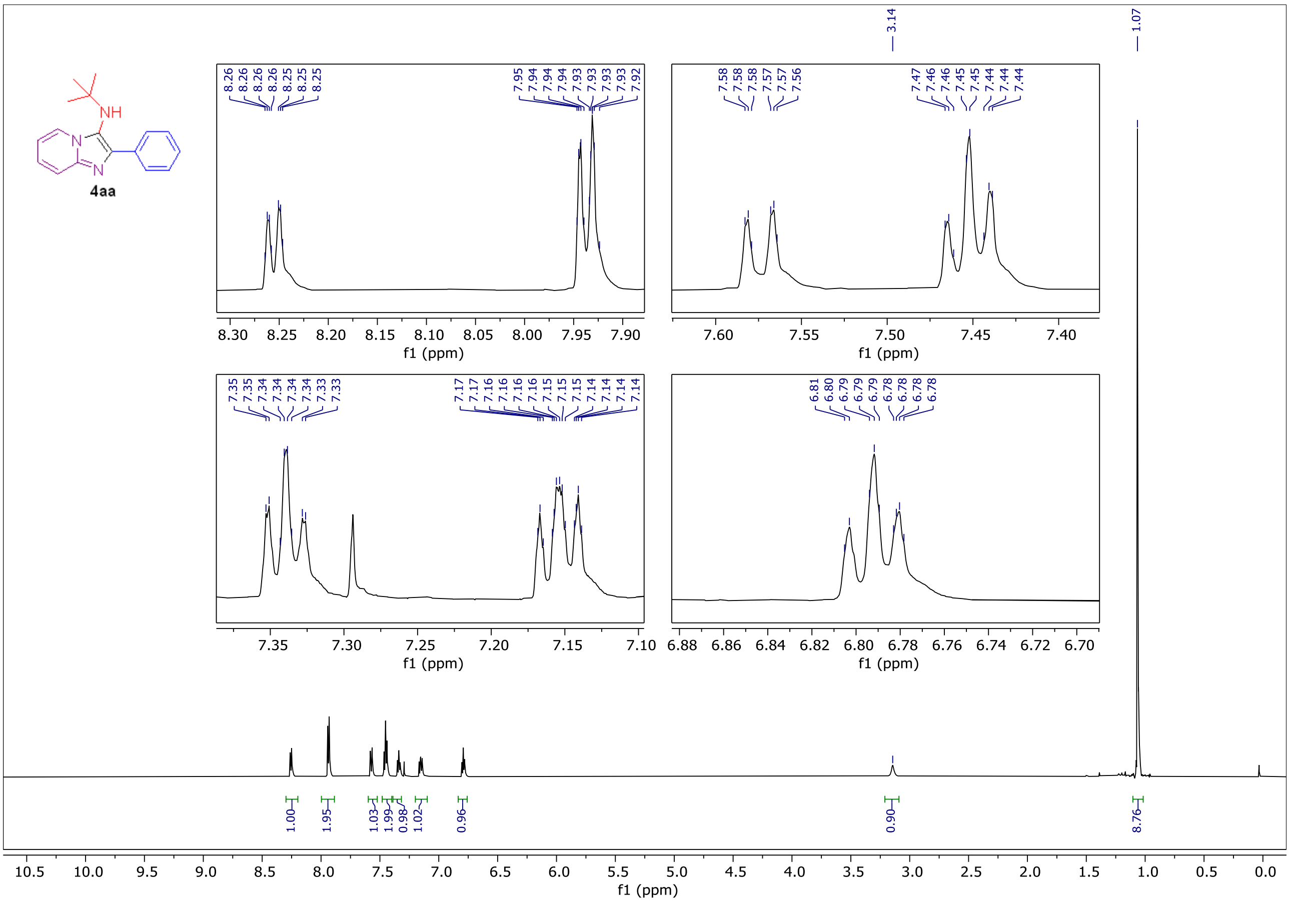

S 106. ^{1}H NMR spectrum (600 MHz, $CDCl_3$) of compound **4aa**.

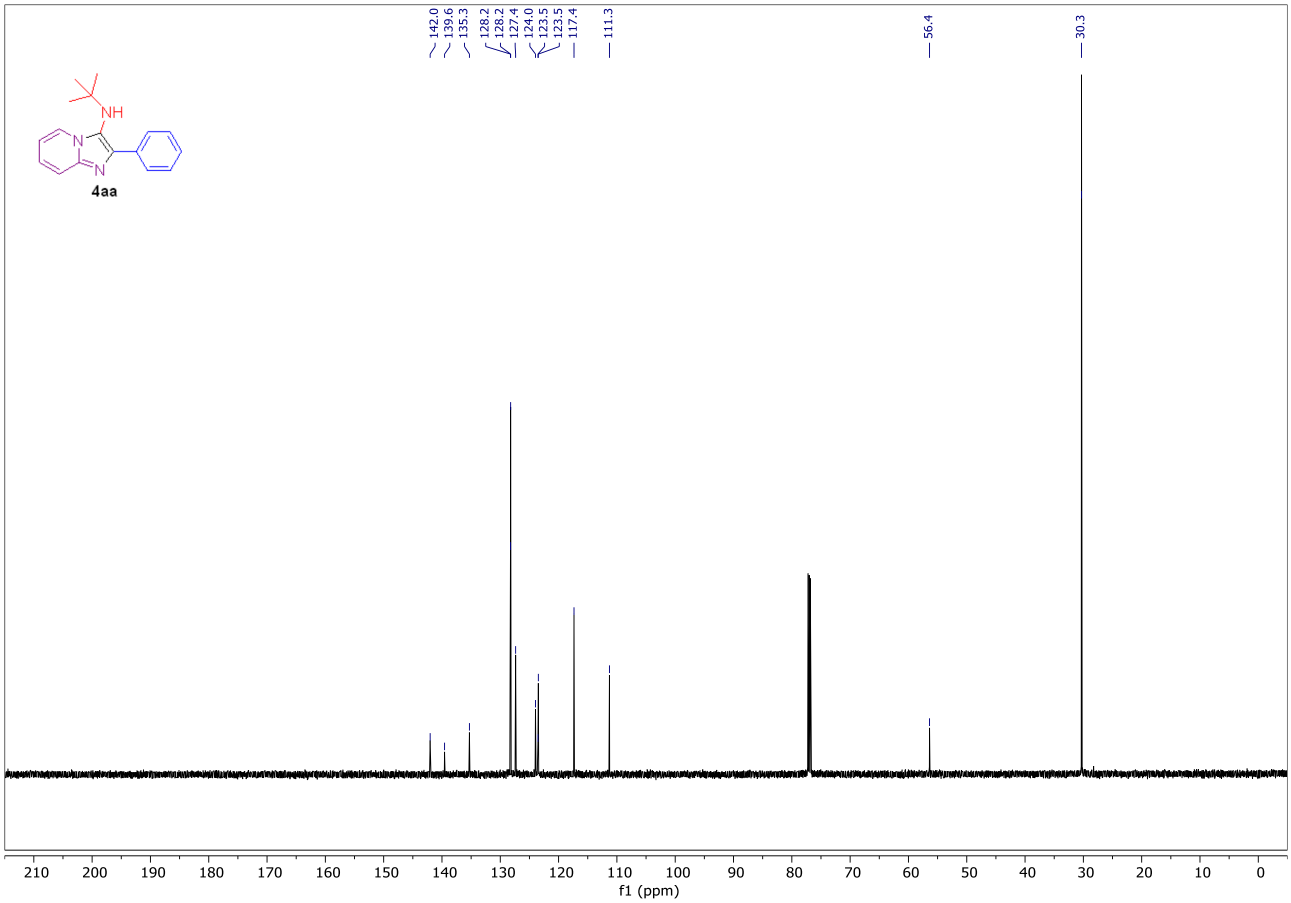

S 107. ^{13}C NMR spectrum (151 MHz, $CDCl_3$) of compound **4aa**.

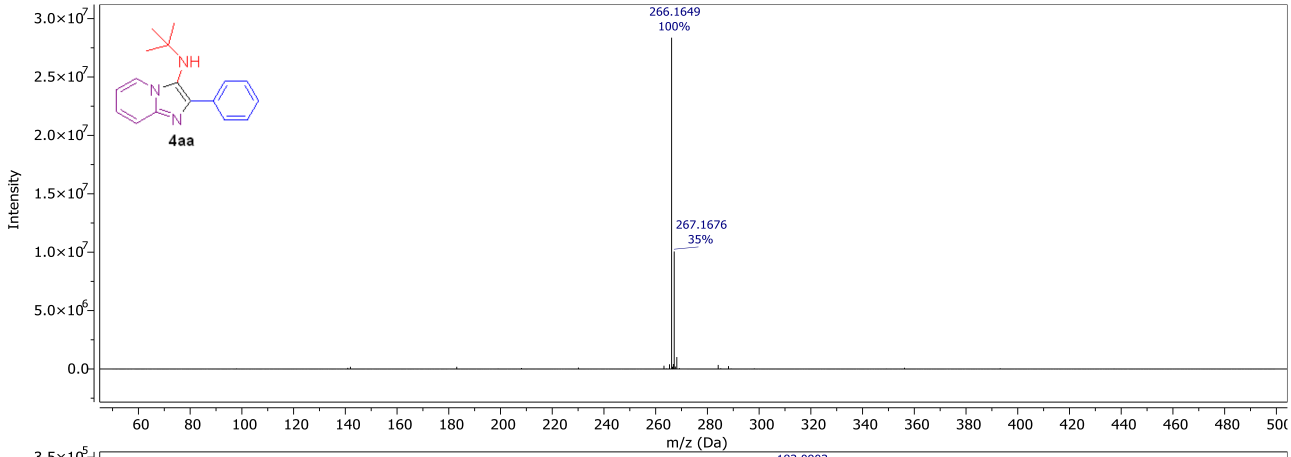

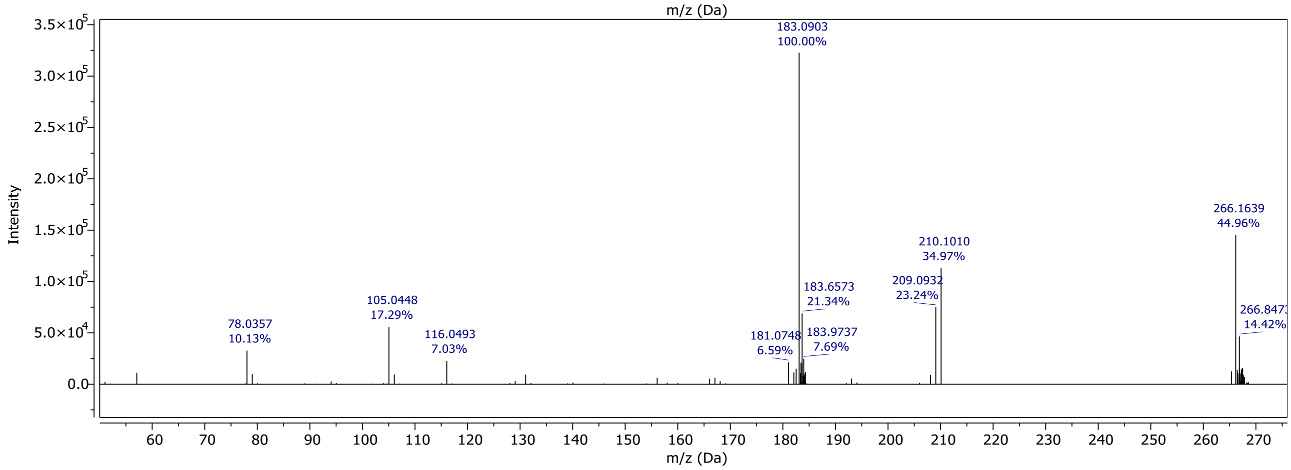

S 108. HRMS (ESI-QTOF) of compound **4aa** and HRMS/MS for [M+H]$^+$.

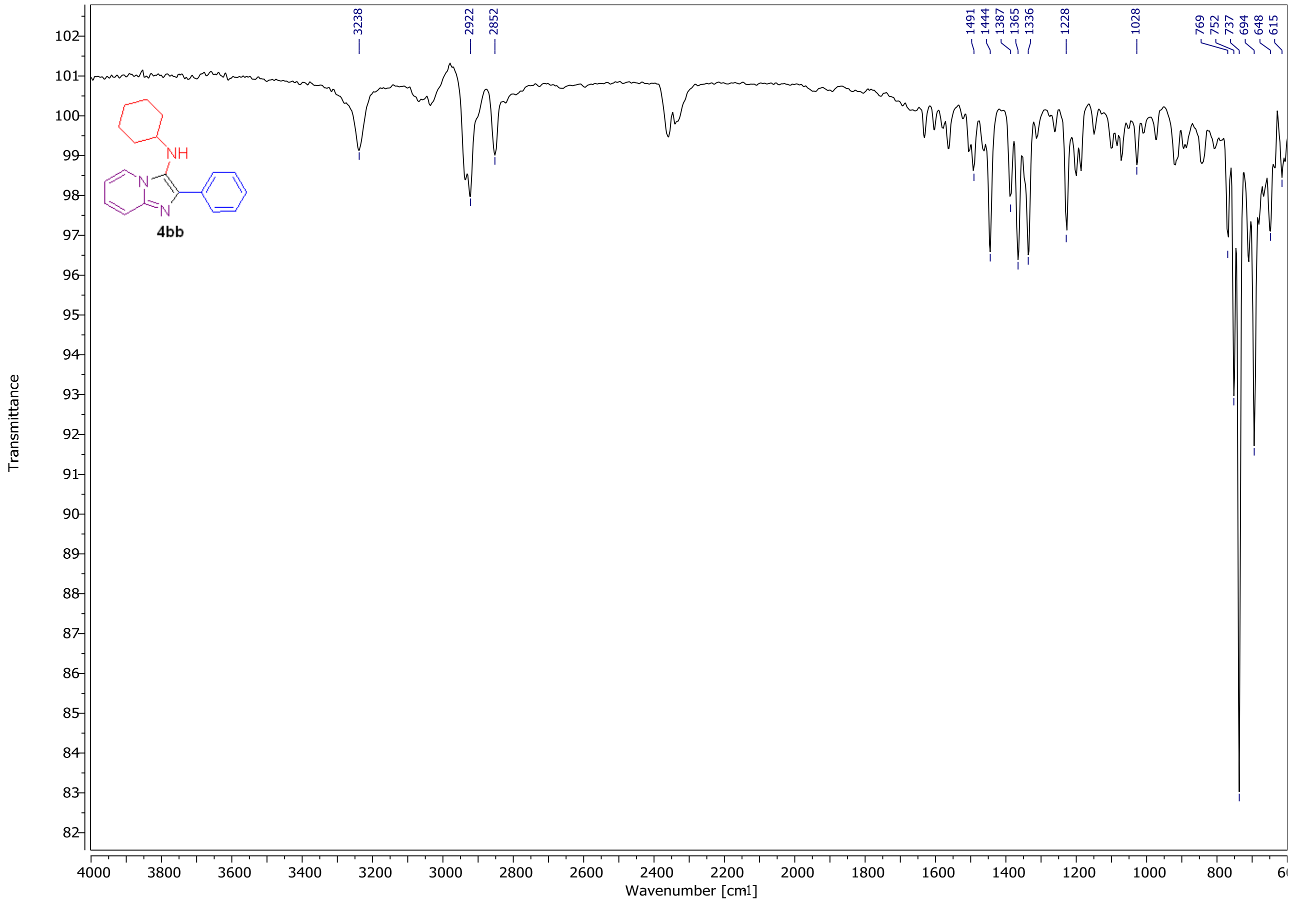

S 109. FT-IR (ATR) of compound **4bb**.

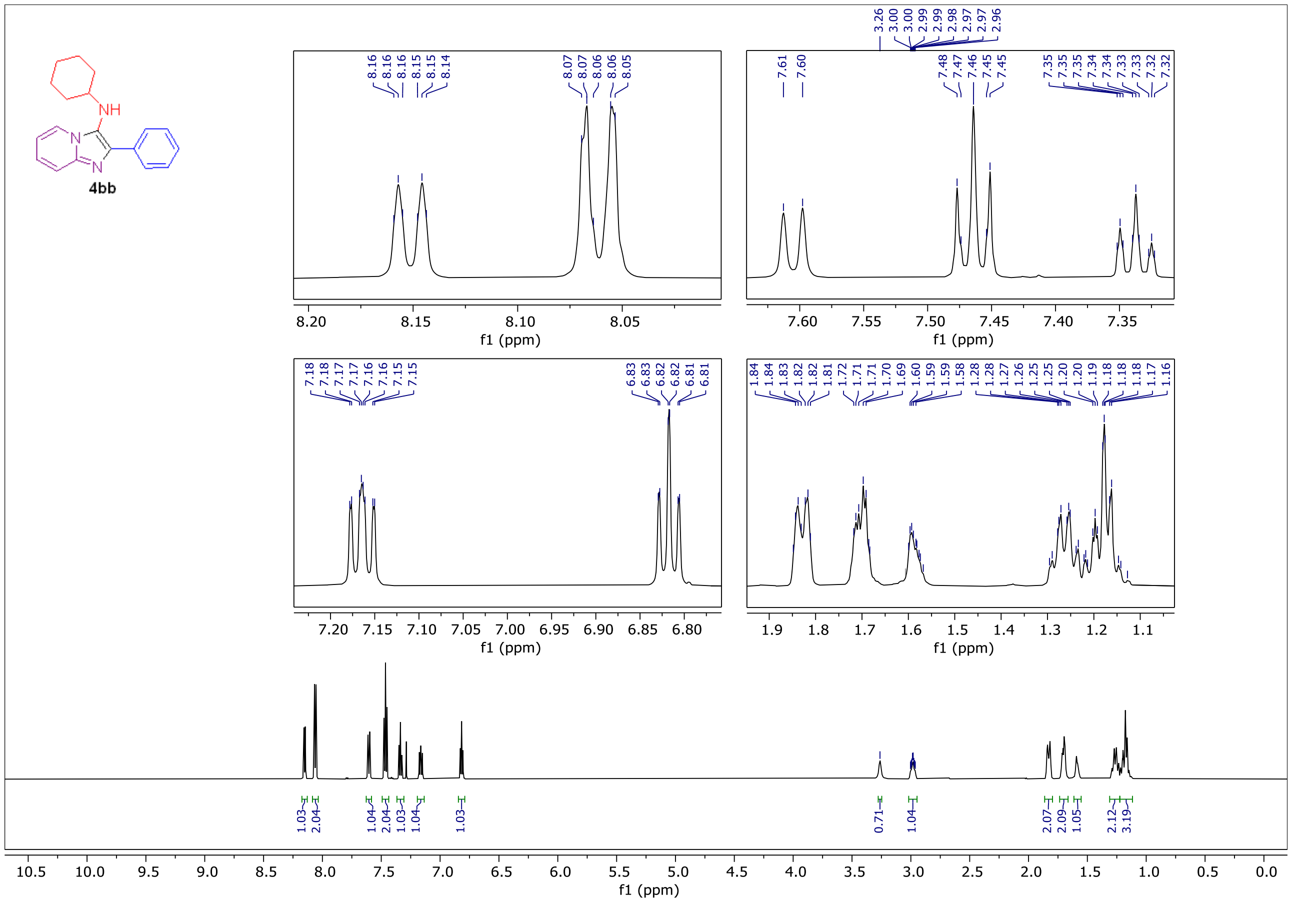

S 110. ^{1}H NMR spectrum (600 MHz, $CDCl_3$) of compound **4bb**.

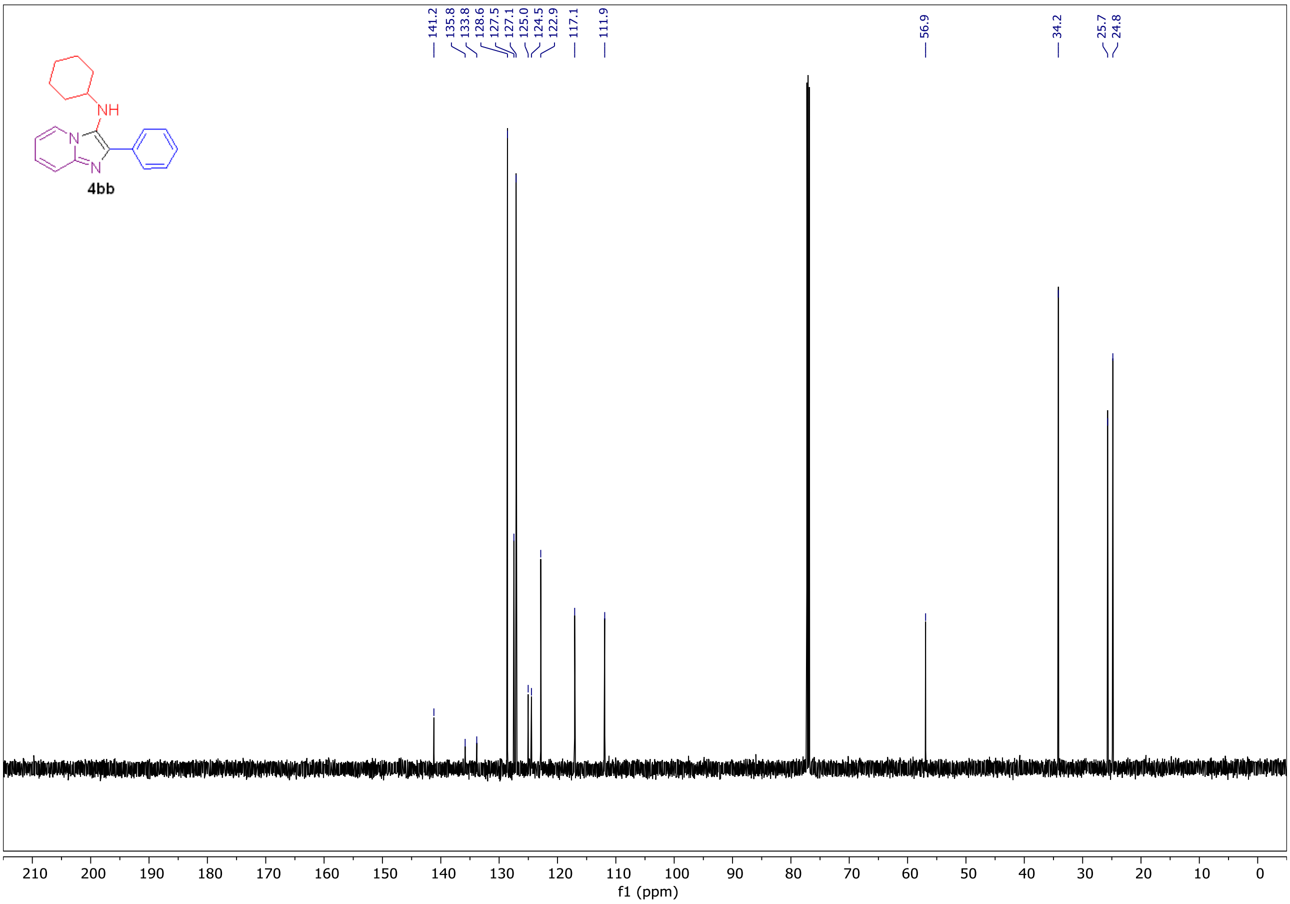

S 111. ^{13}C NMR spectrum (151 MHz, $CDCl_3$) of compound **4bb**.

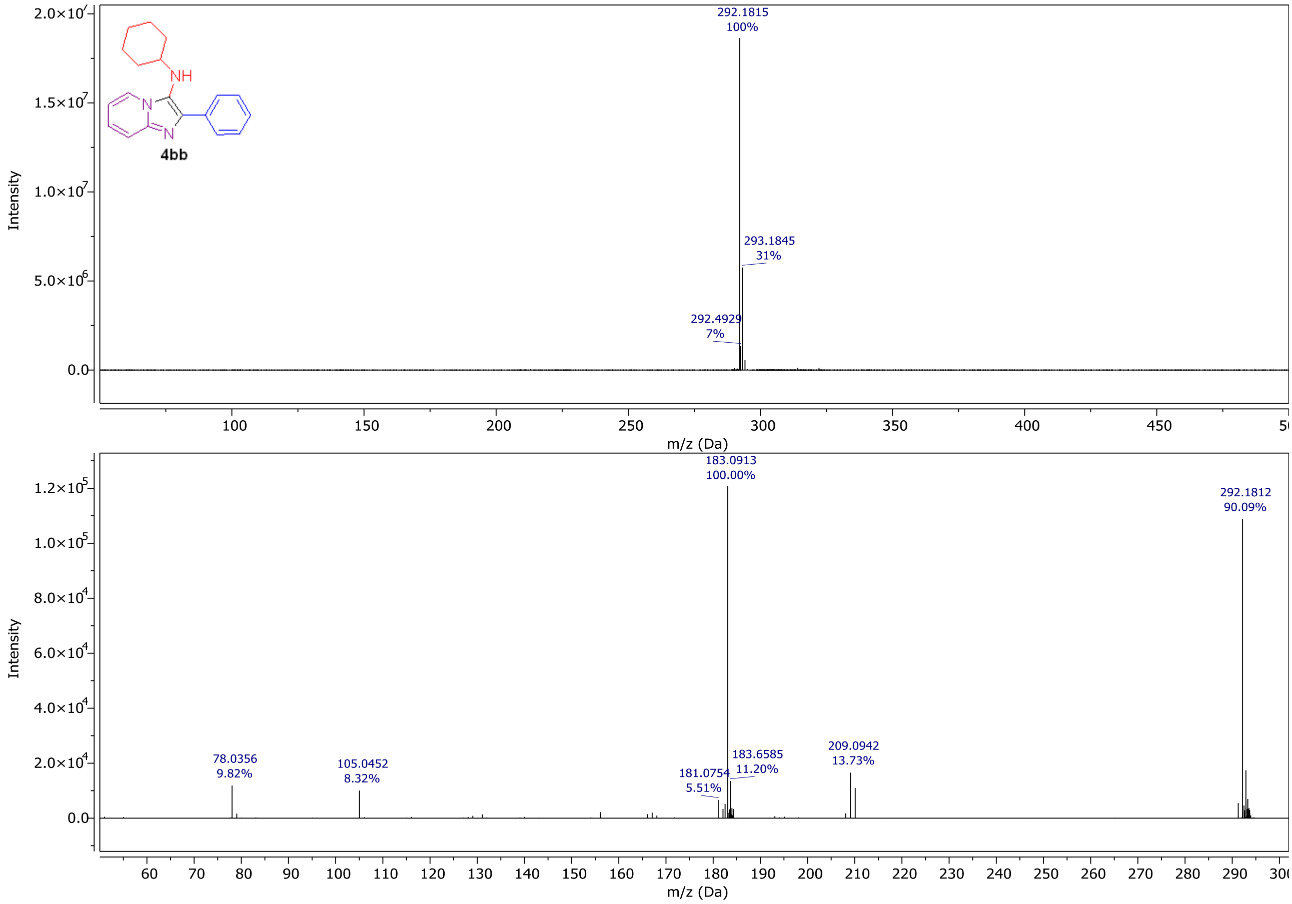

S 112. HRMS (ESI-QTOF) of compound **4bb** and HRMS/MS for $[M+H]^+$.

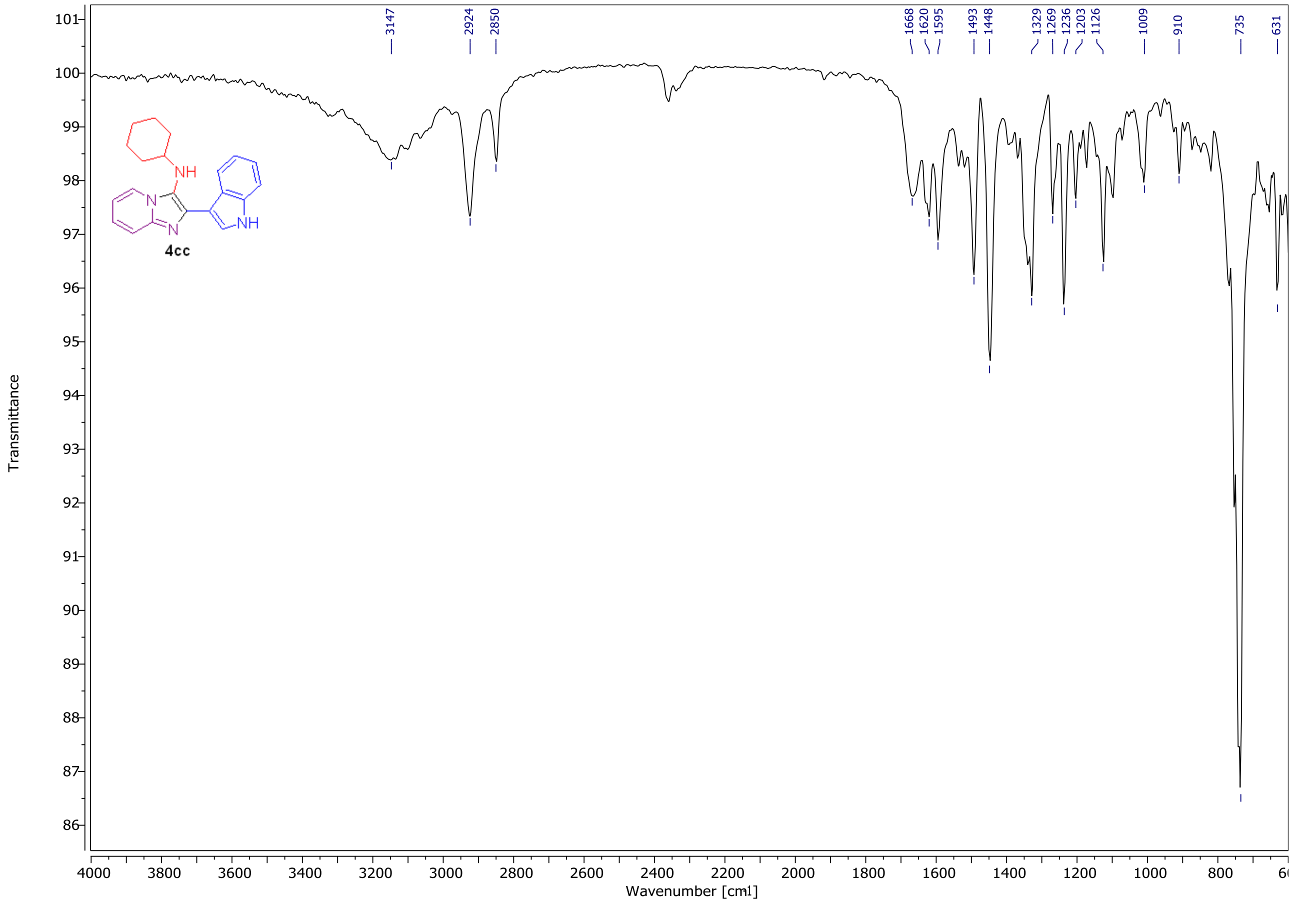

S 113. FT-IR (ATR) of compound **4cc**.

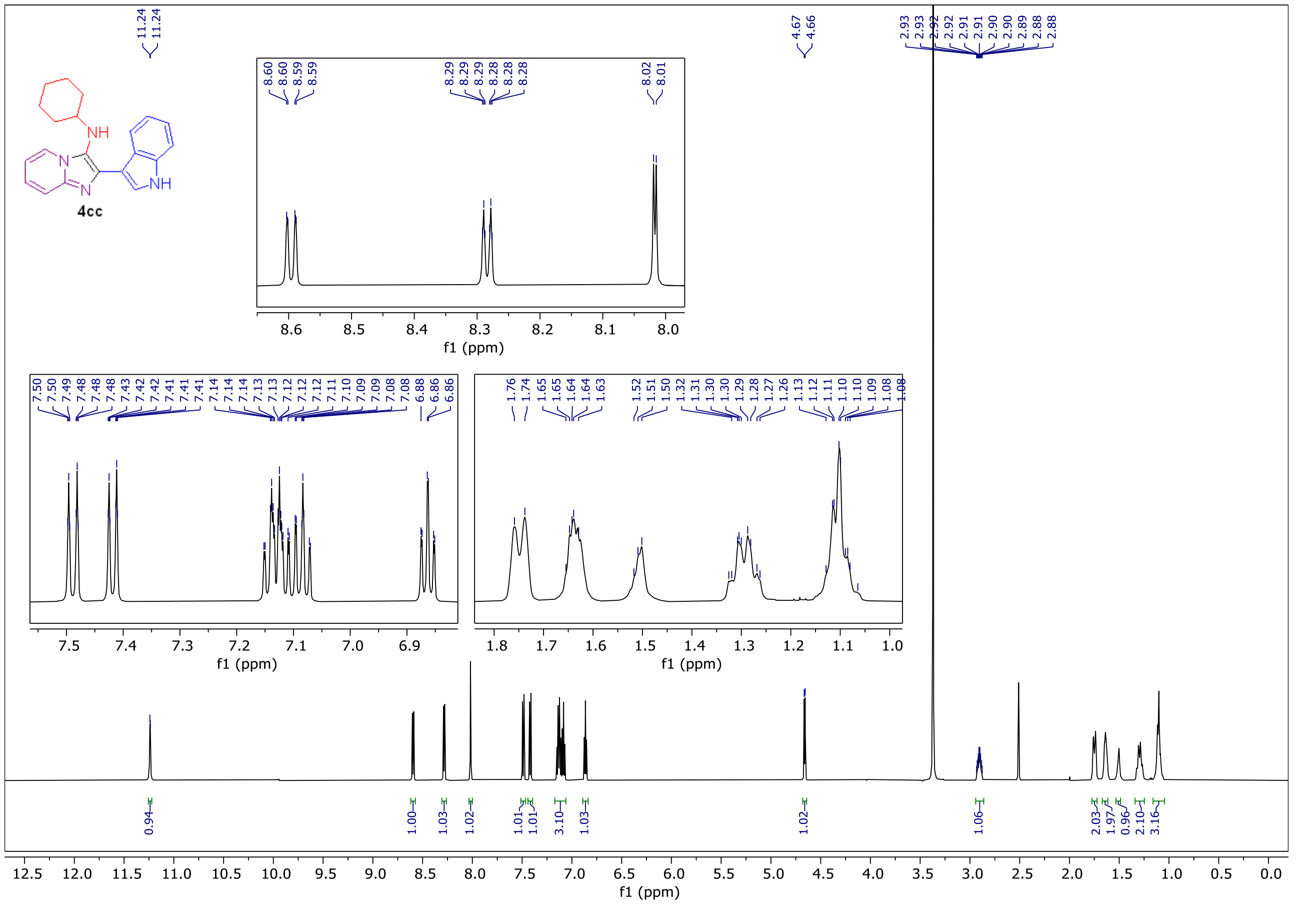

S 114. ^{1}H NMR spectrum (600 MHz, DMSO-*d*6) of compound **4cc**.

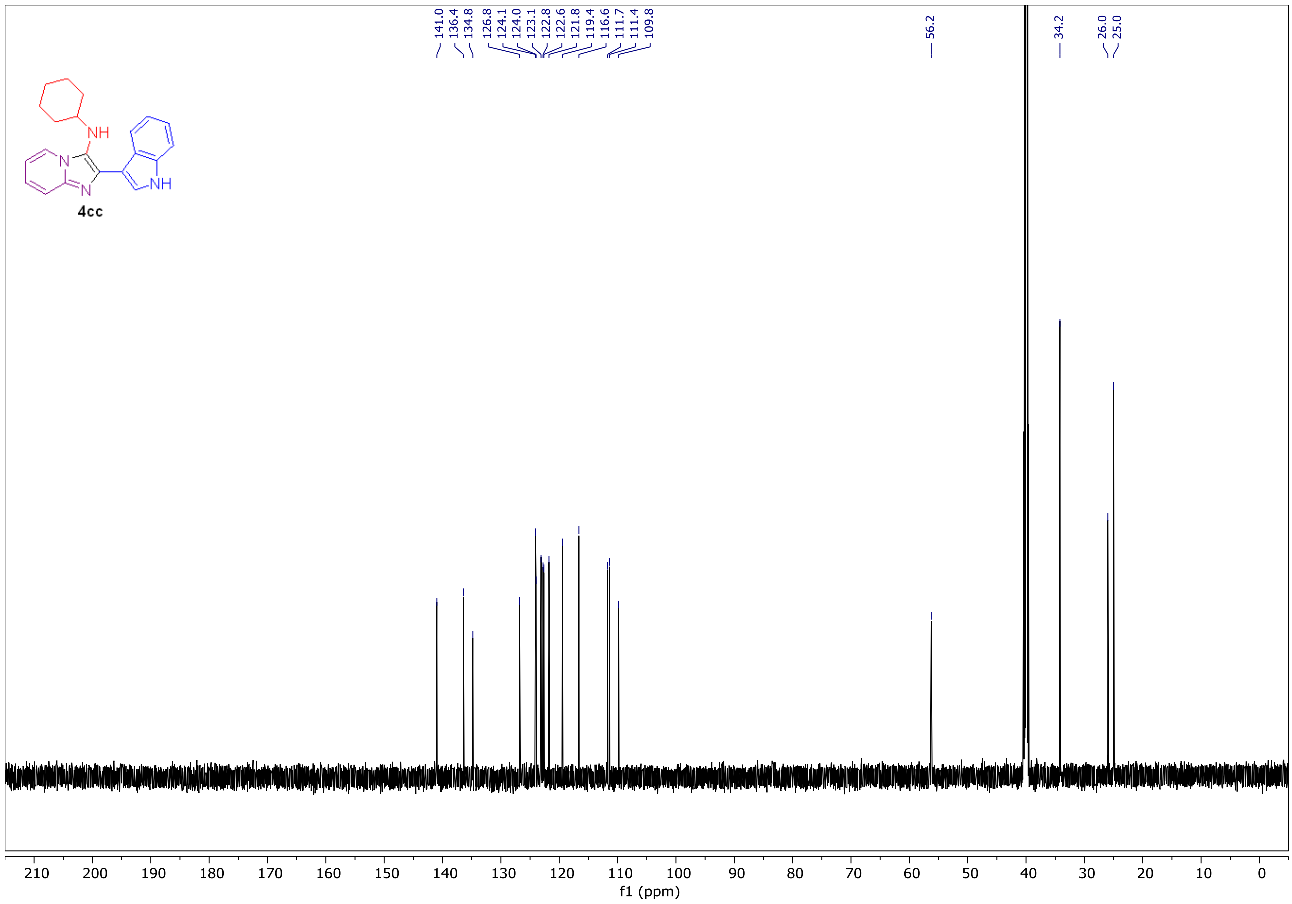

S 115. ^{13}C NMR spectrum (151 MHz, DMSO-d_6) of compound **4cc**.

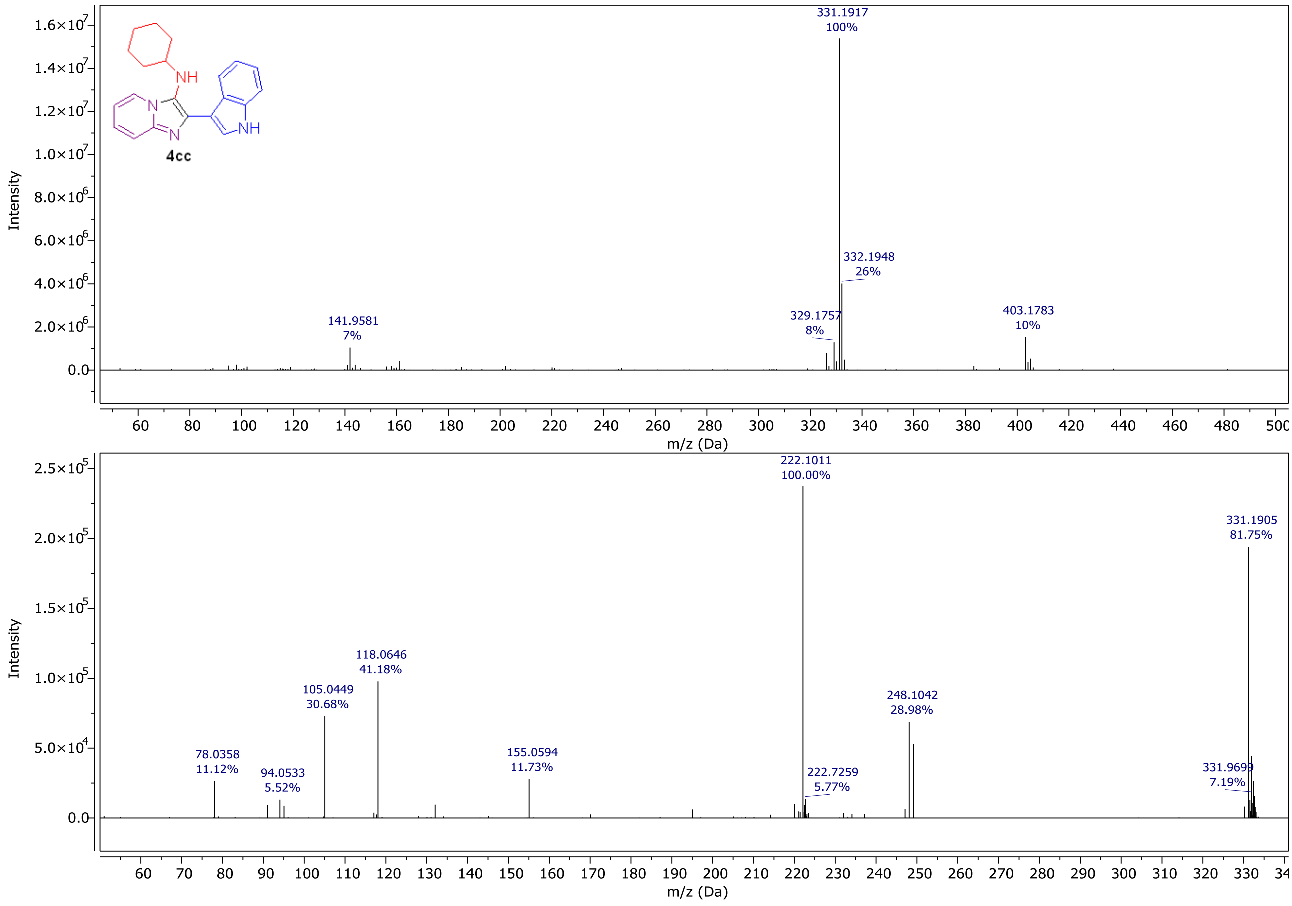

S 116. HRMS (ESI-QTOF) of compound **4cc** and HRMS/MS for [M+H]$^+$.

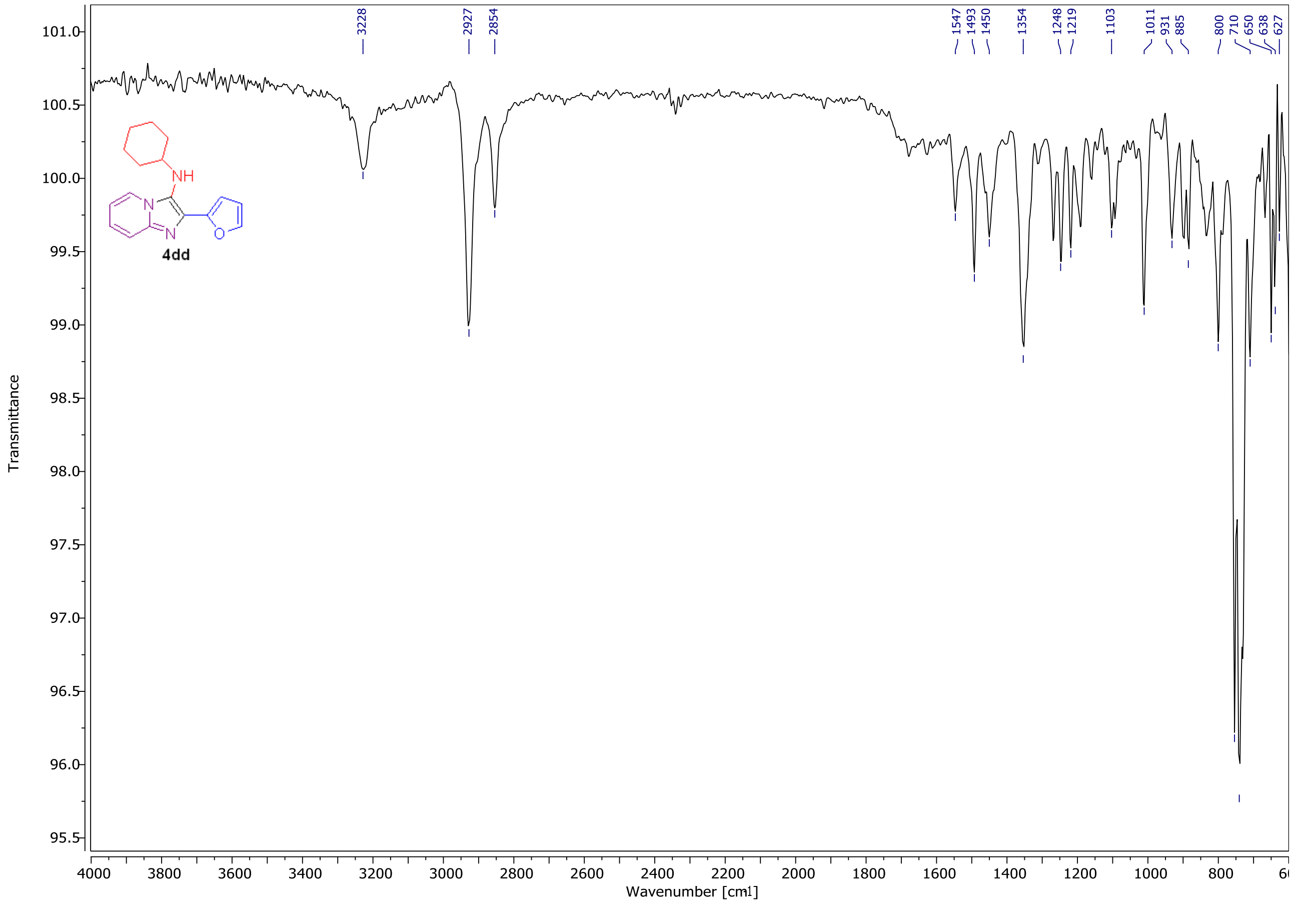

S 117. FT-IR (ATR) of compound **4dd**.

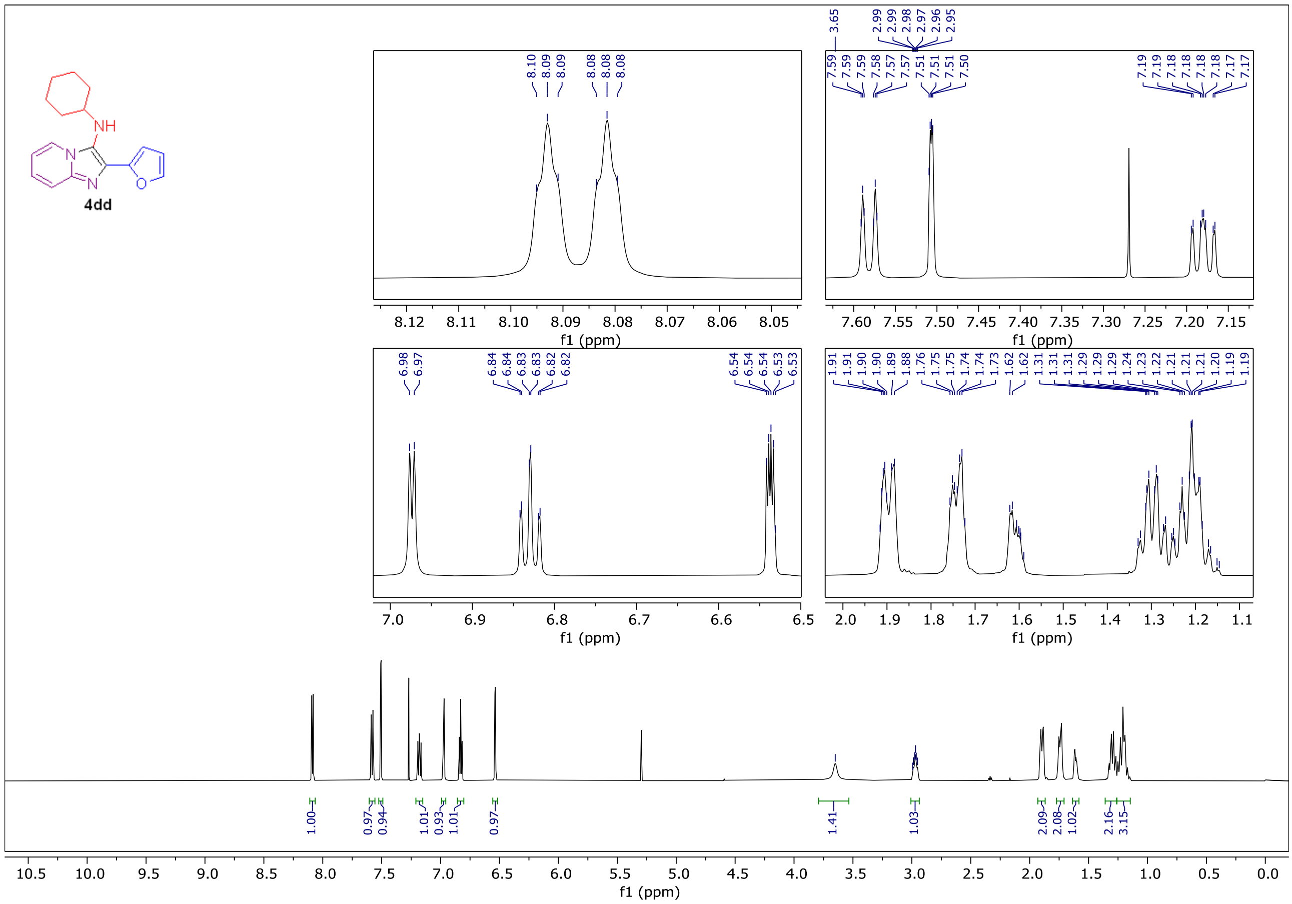

S 118. ^{1}H NMR spectrum (600 MHz, $CDCl_3$) of compound **4dd**.

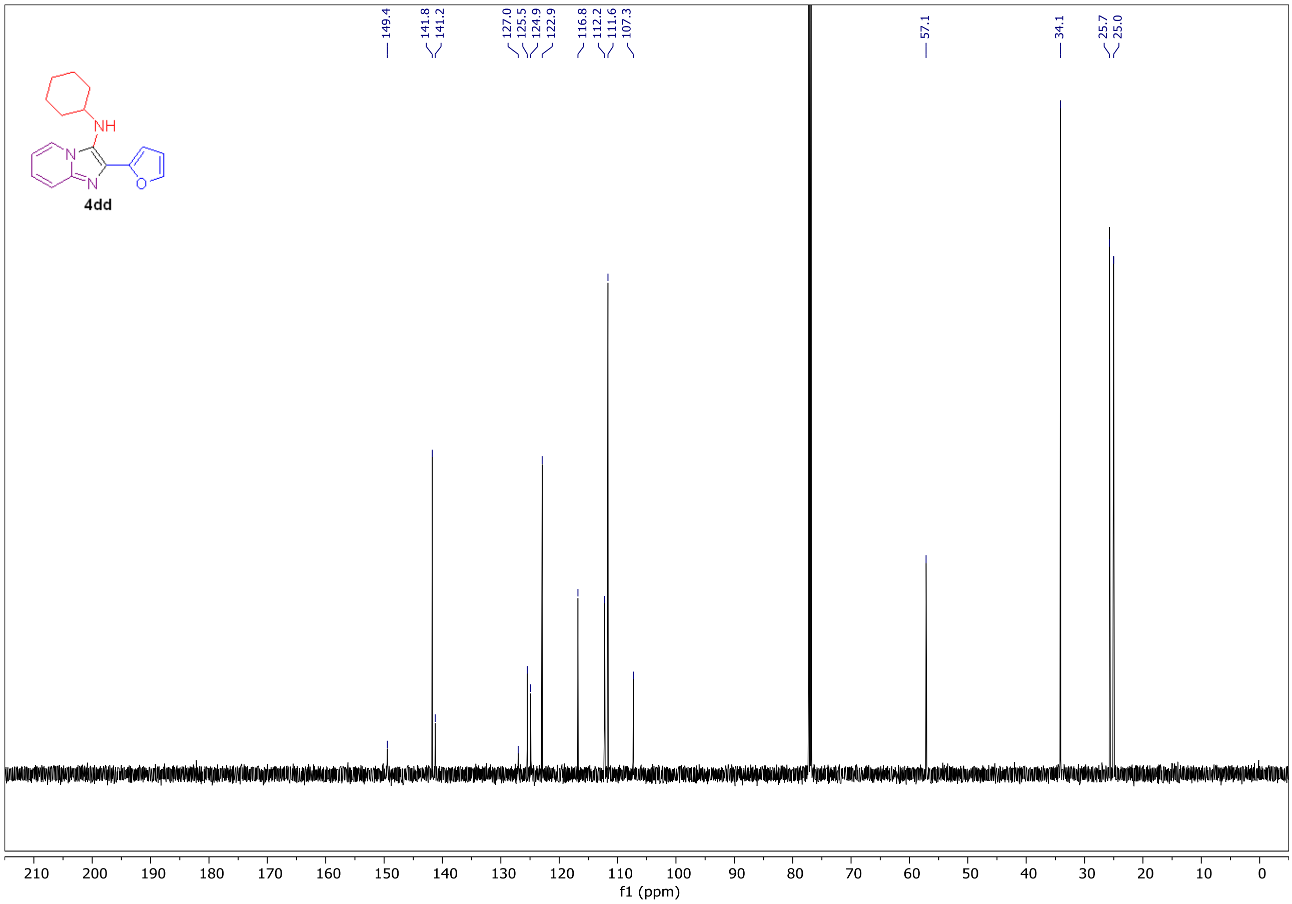

S 119. ^{13}C NMR spectrum (151 MHz, $CDCl_3$) of compound **4dd**.

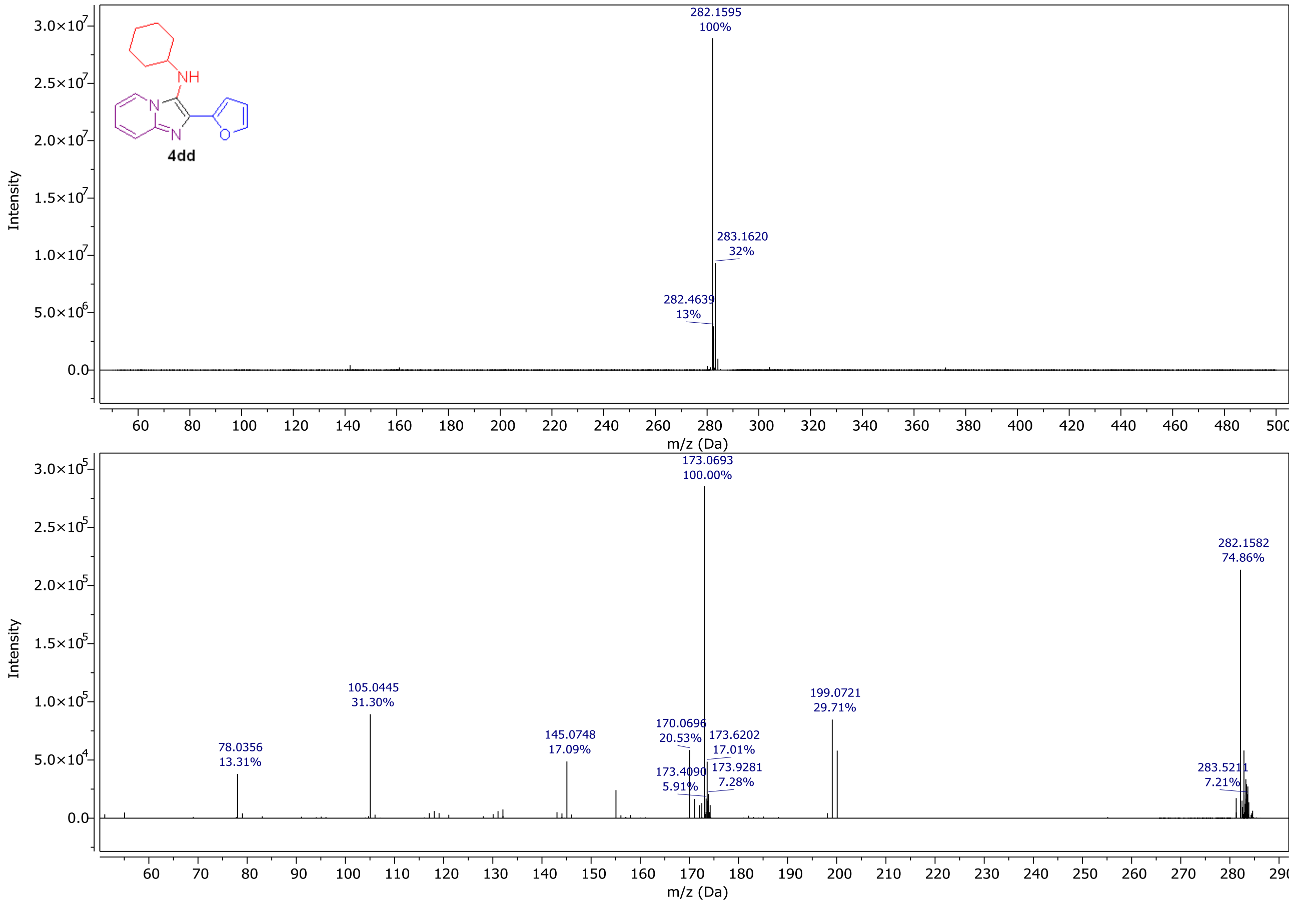

S 120. HRMS (ESI-QTOF) of compound **4dd** and HRMS/MS for $[M+H]^+$.

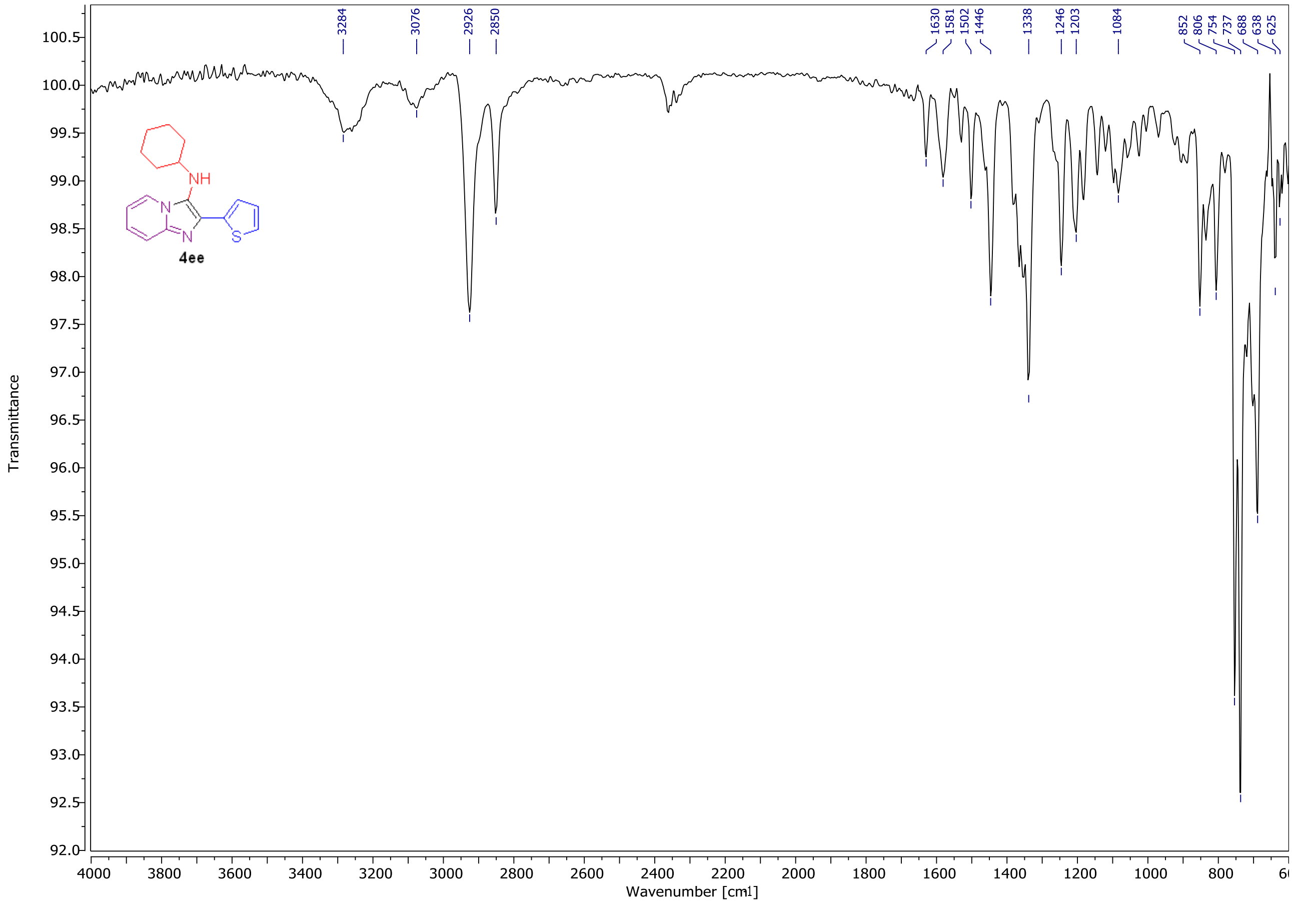

S 121. FT-IR (ATR) of compound **4ee**.

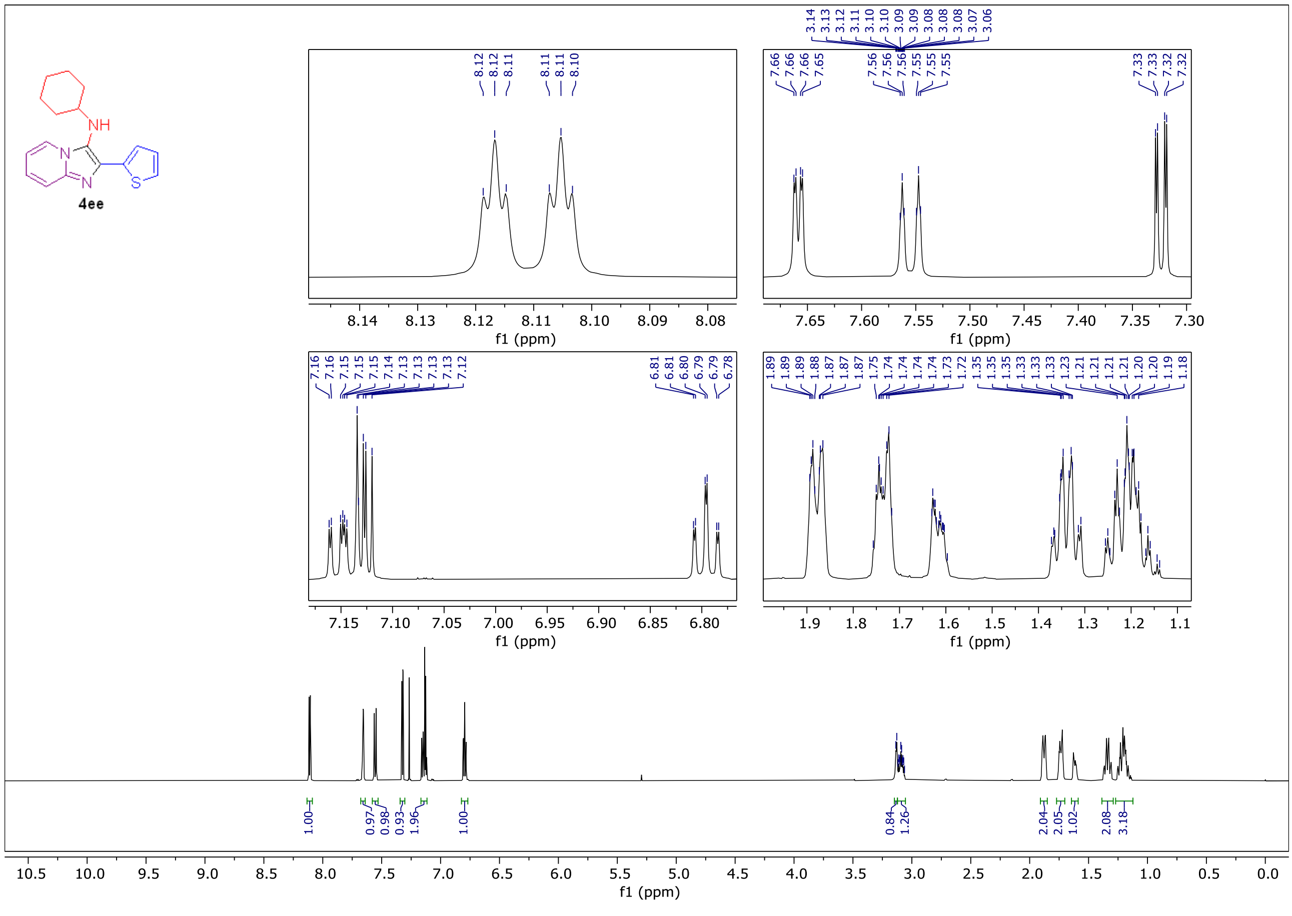

S 122. ^{1}H NMR spectrum (600 MHz, $CDCl_3$) of compound **4ee**.

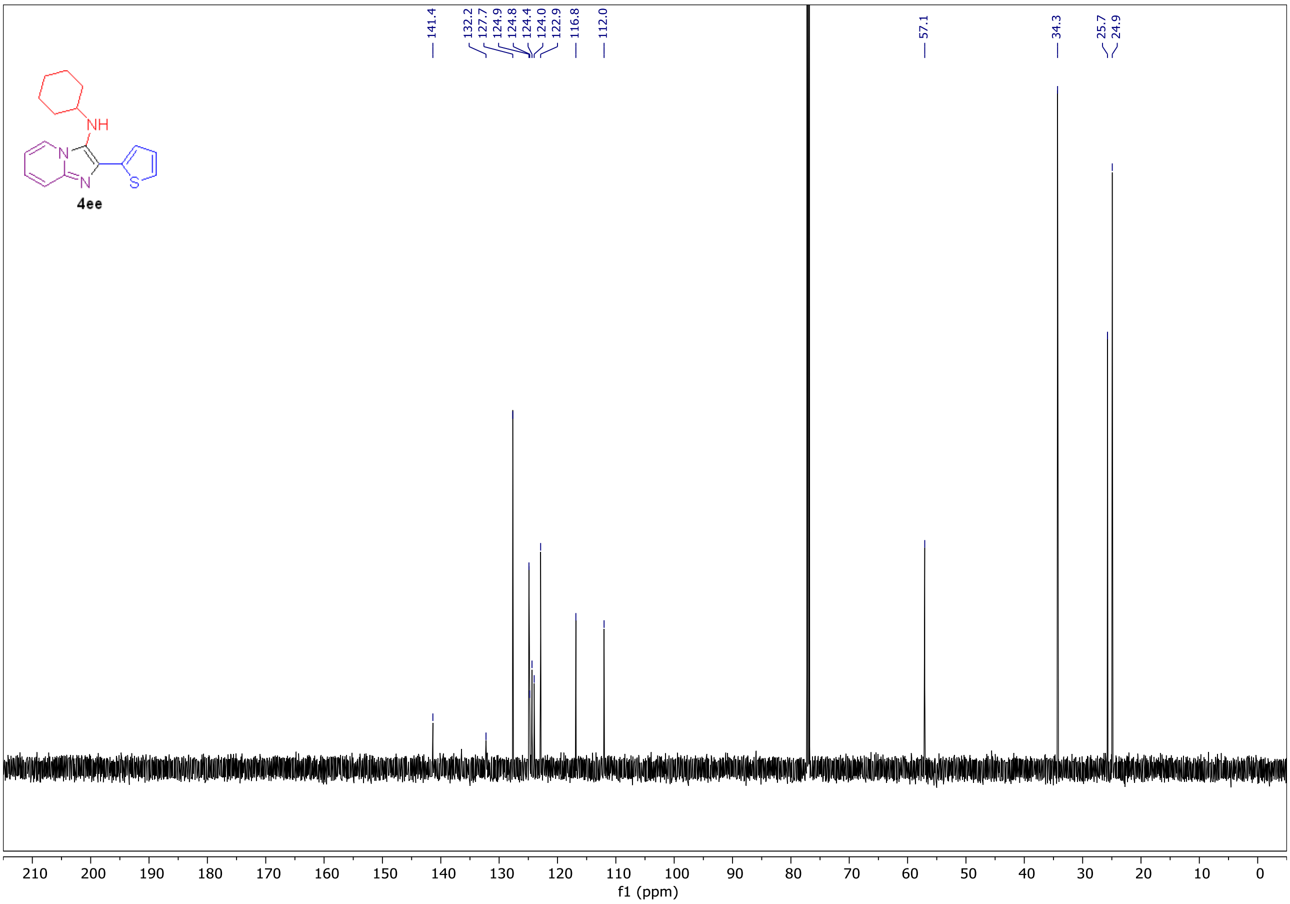

S 123. ^{13}C NMR spectrum (151 MHz, $CDCl_3$) of compound **4ee**.

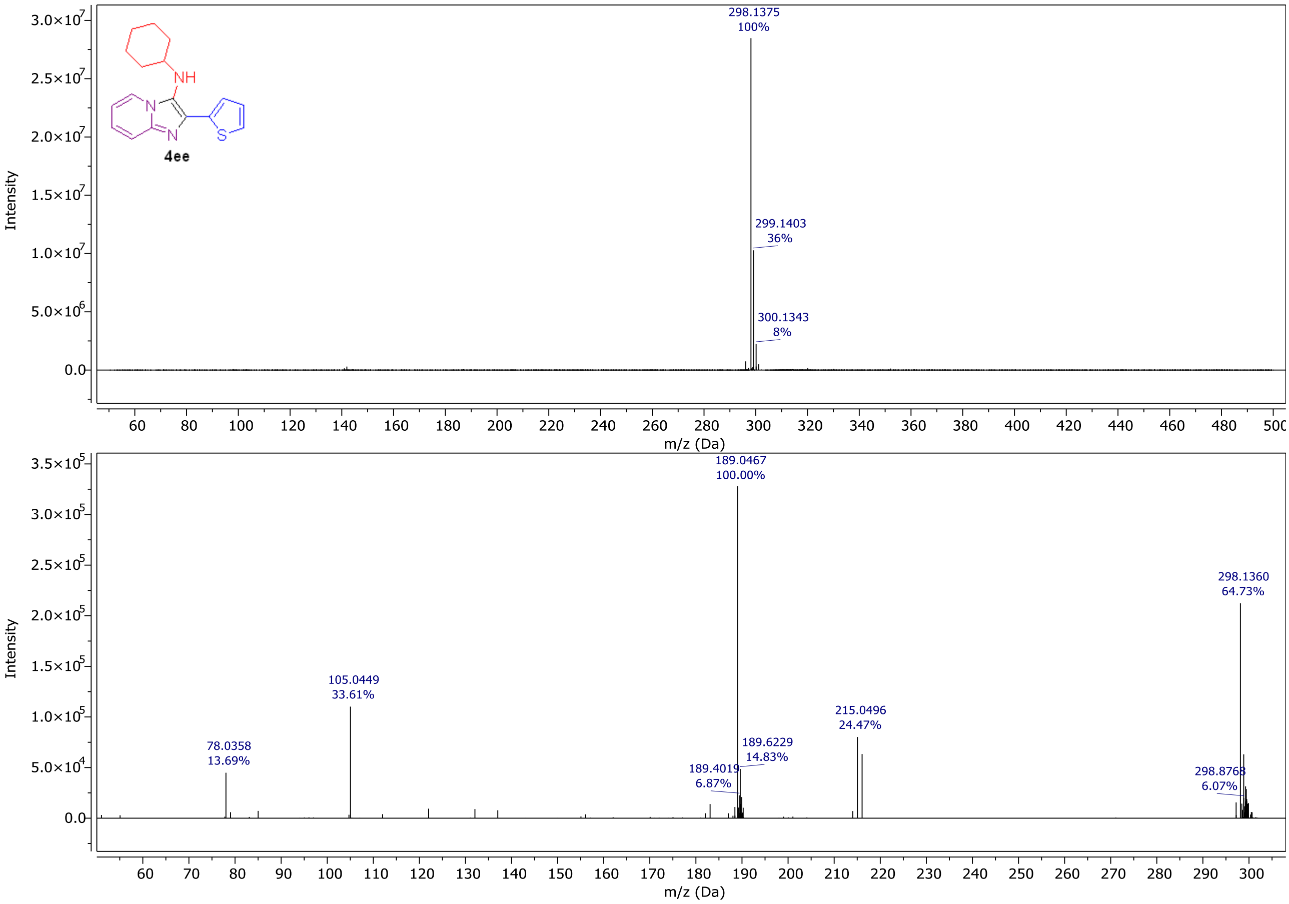

S 124. HRMS (ESI-QTOF) of compound **4ee** and HRMS/MS for $[M+H]^+$.

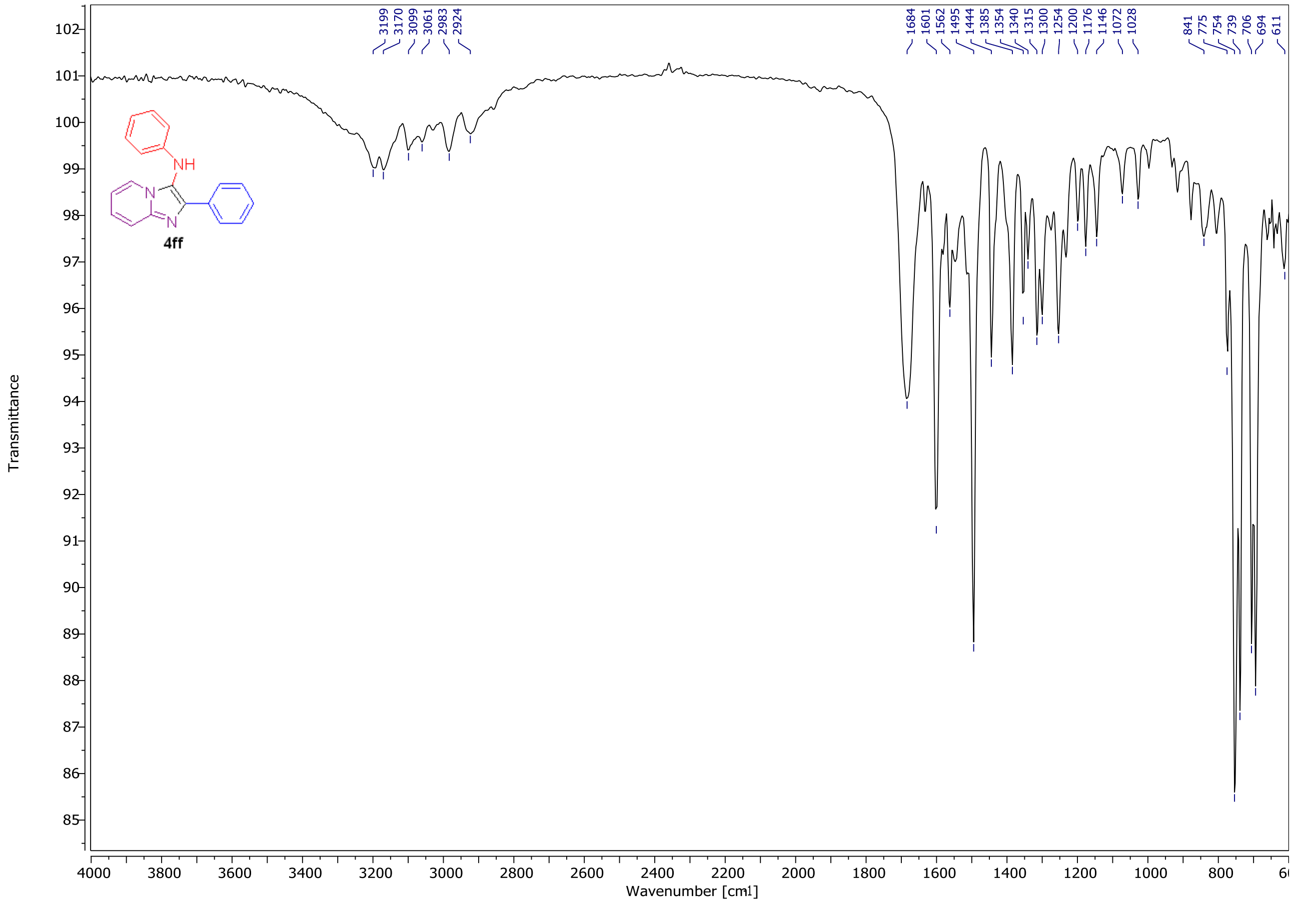

S 125. FT-IR (ATR) of compound **4ff**.

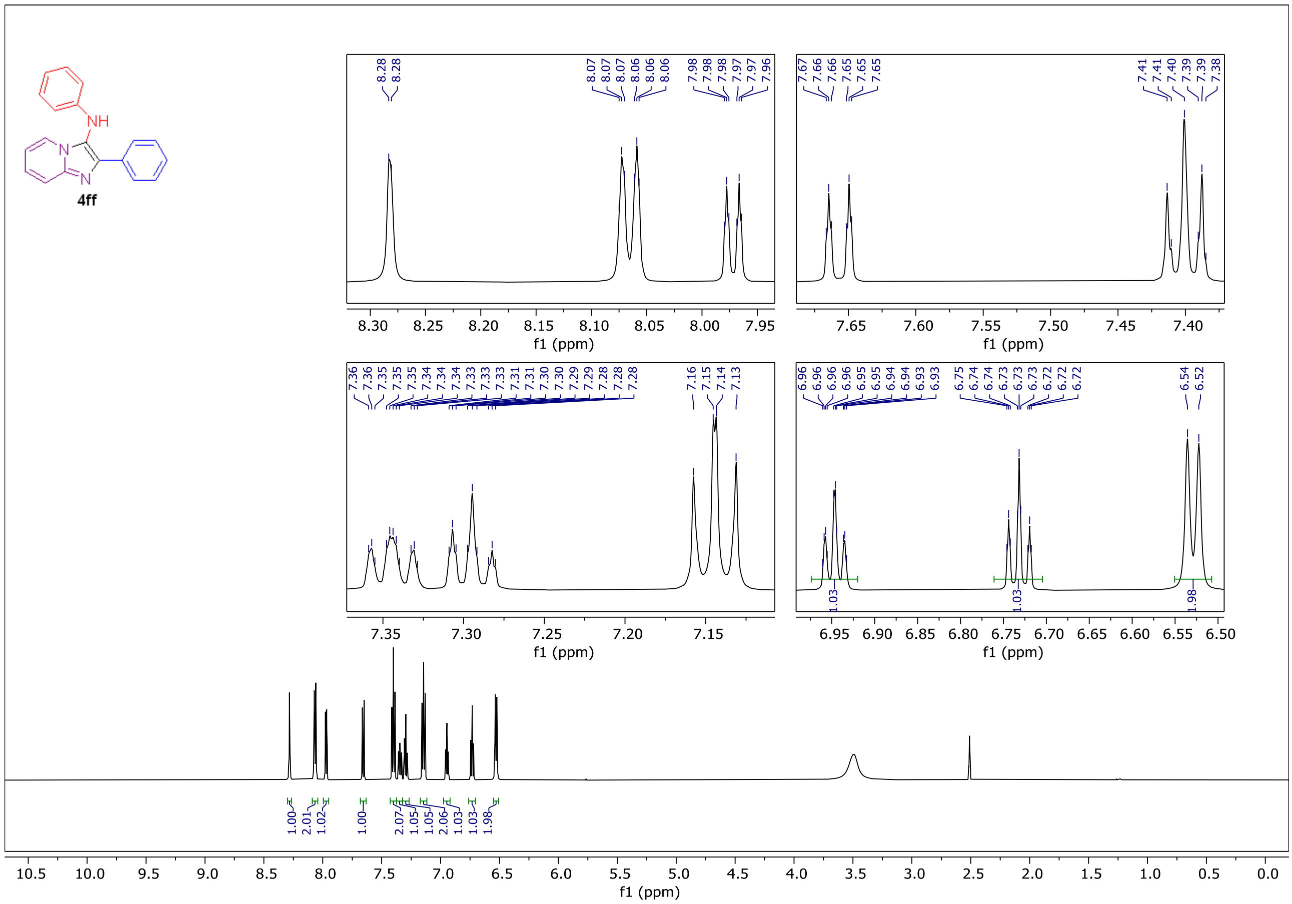

S 126. ^{1}H NMR spectrum (600 MHz, DMSO-d_6) of compound **4ff**.

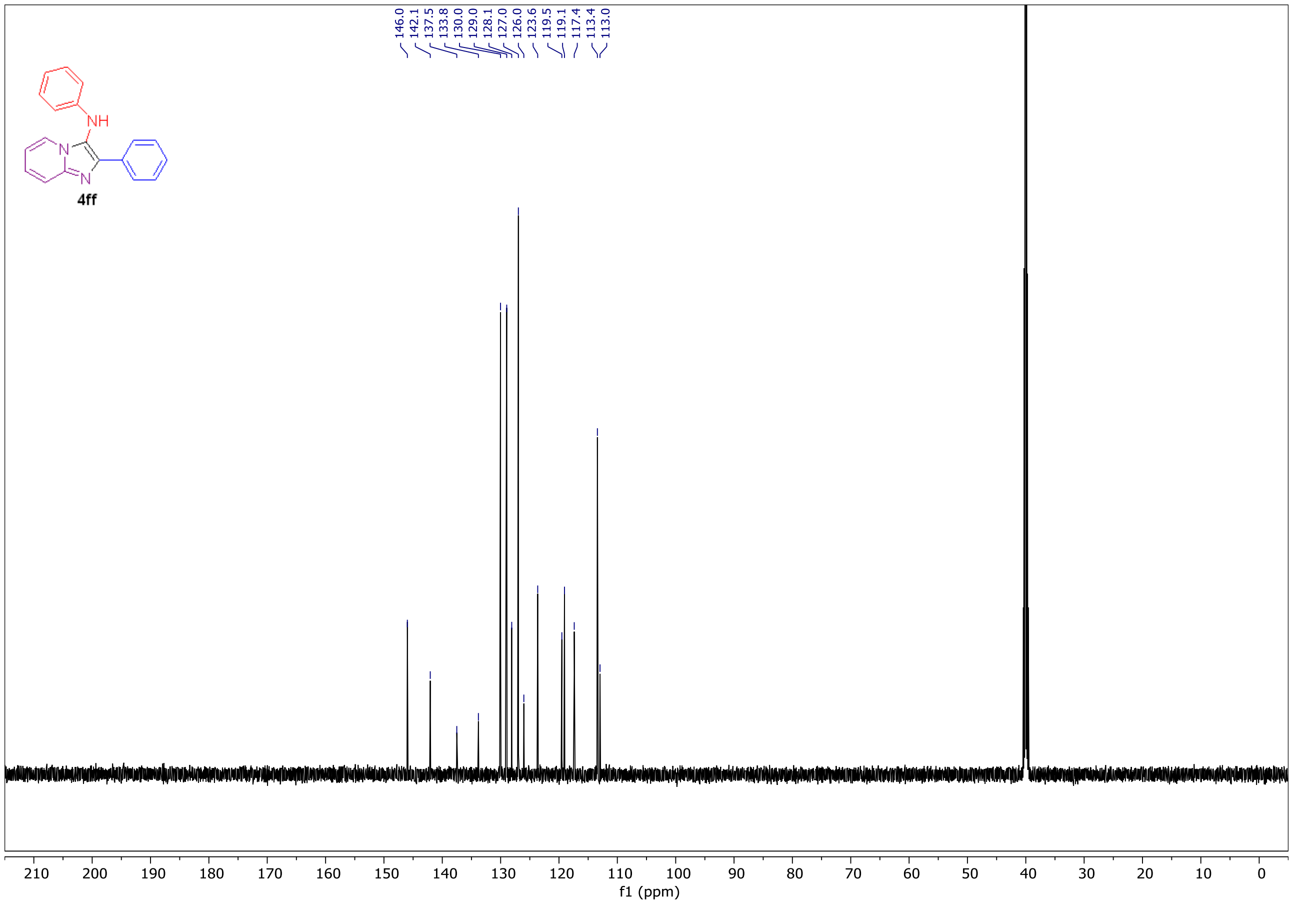

S 127. ^{13}C NMR spectrum (151 MHz, DMSO-d_6) of compound **4ff**.

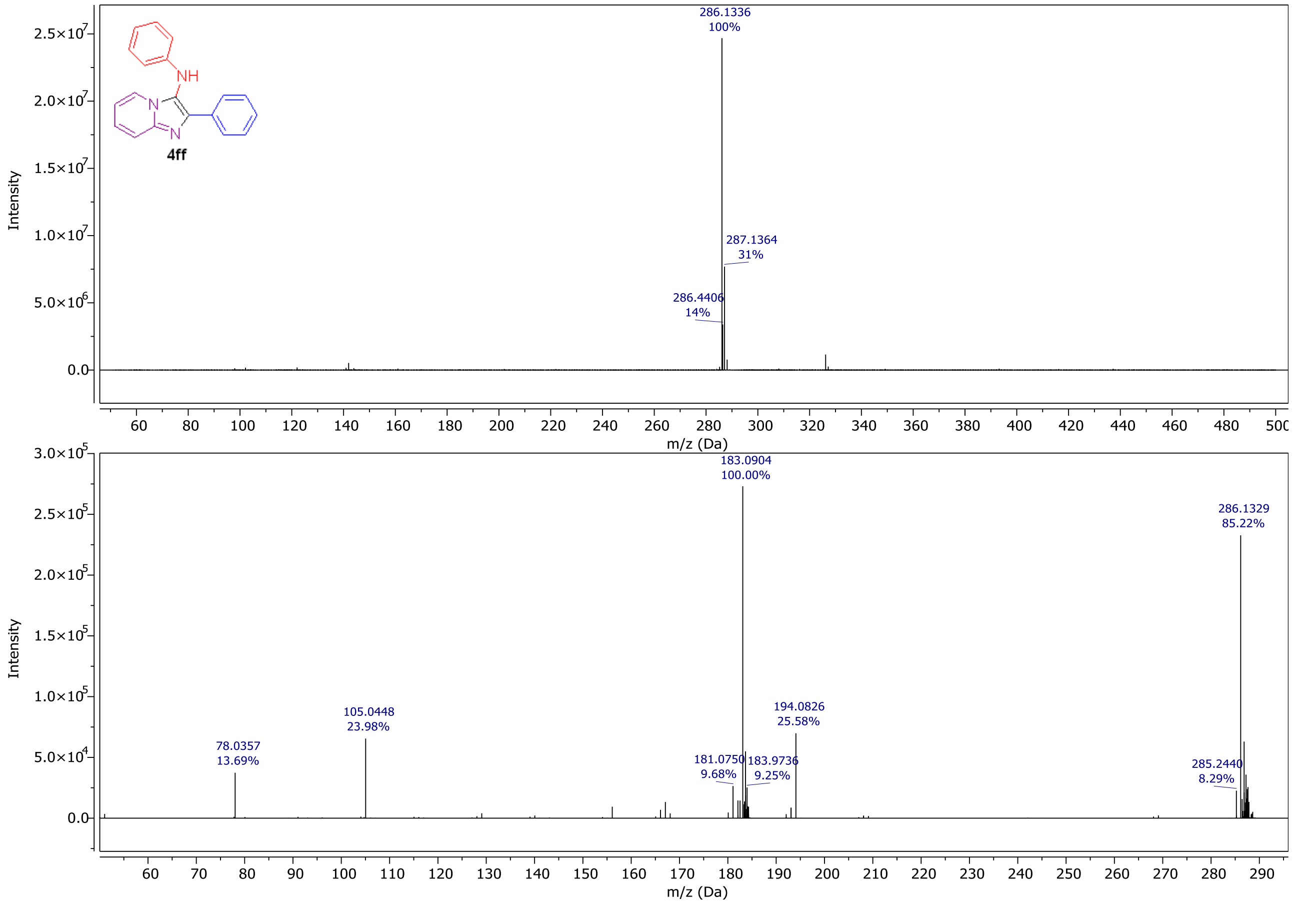

S 128. HRMS (ESI-QTOF) of compound **4ff** and HRMS/MS for [M+H]$^+$.

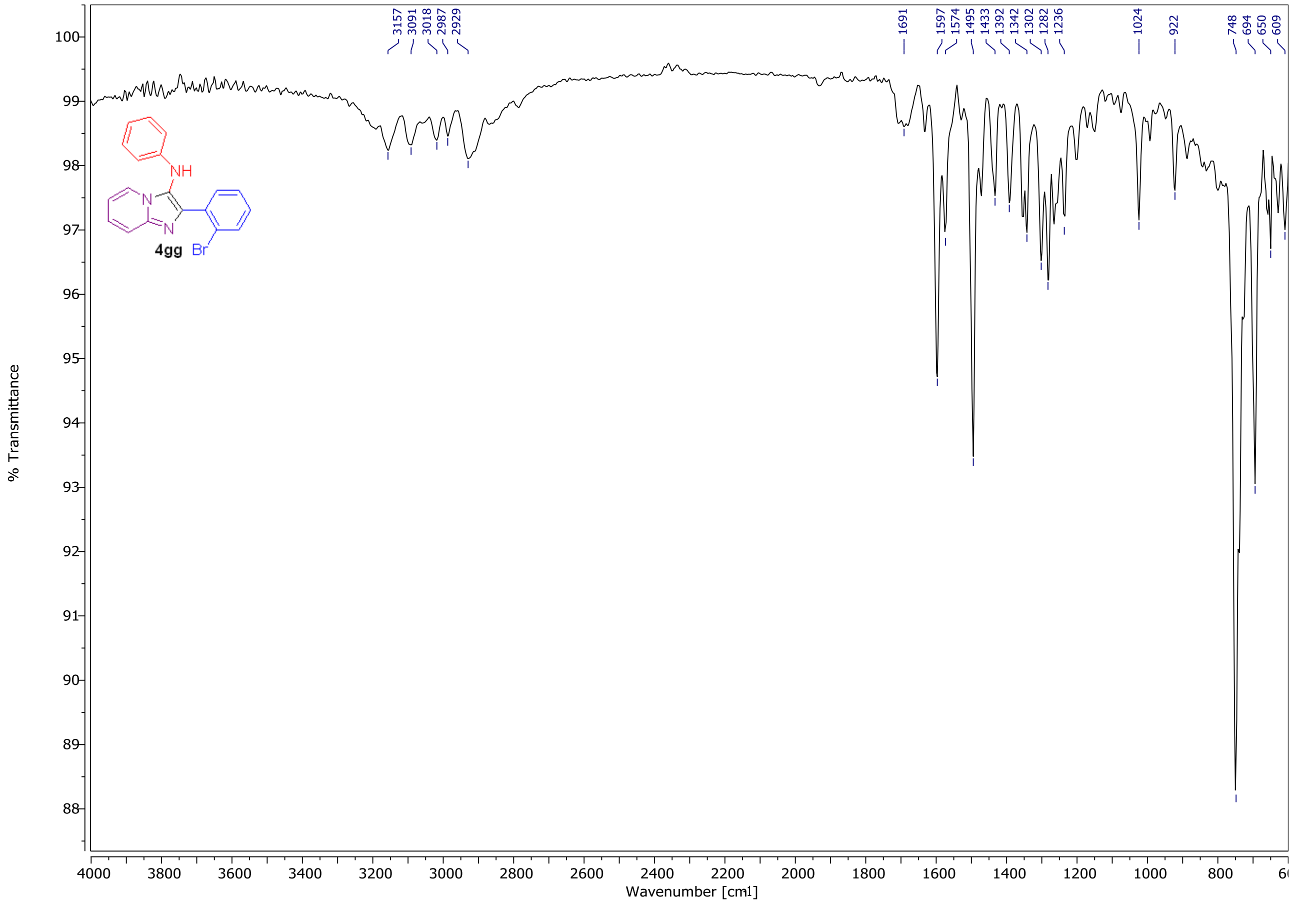

S 129. FT-IR (ATR) of compound **4gg**.

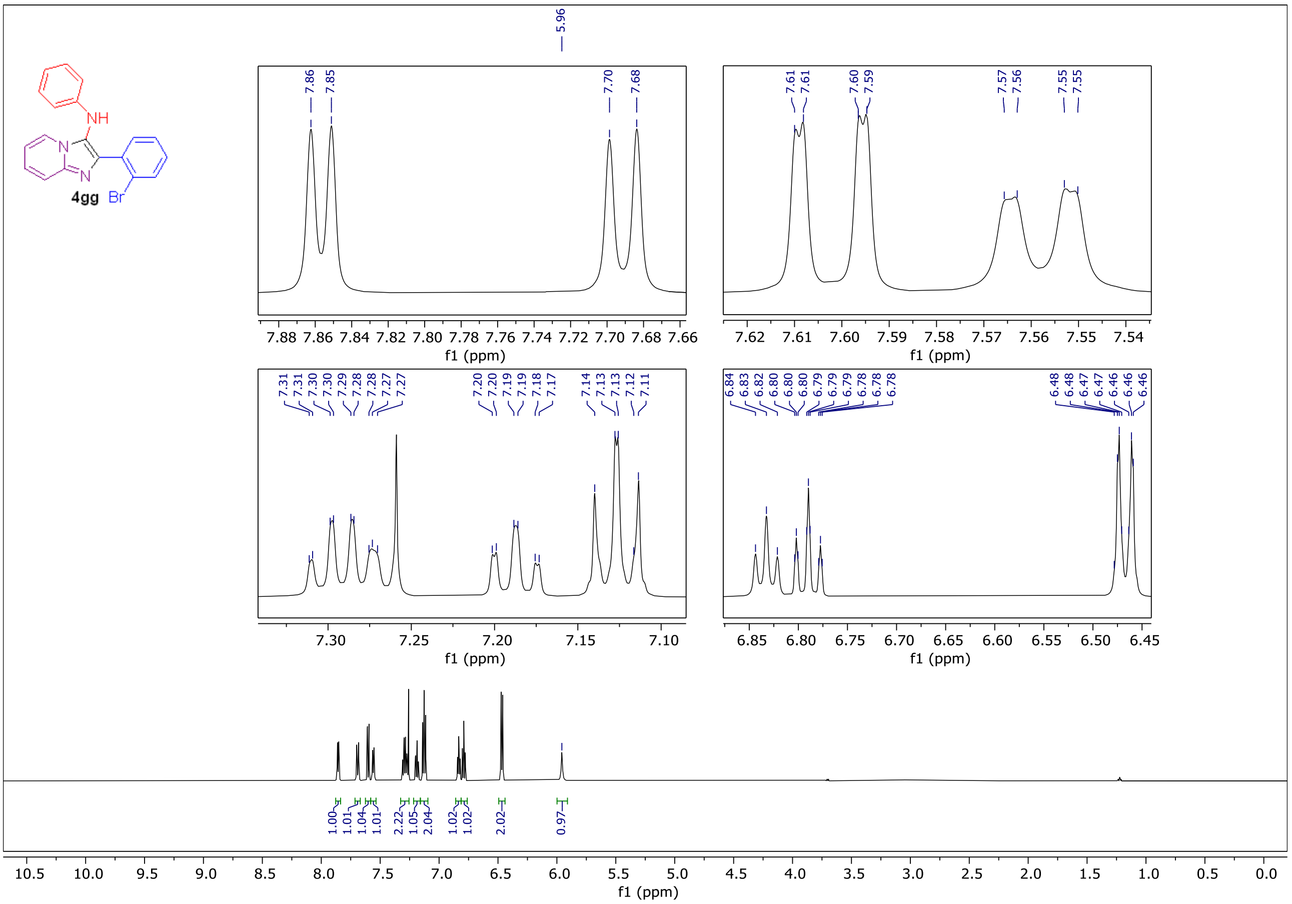

S 130. ^{1}H NMR spectrum (600 MHz, $CDCl_3$) of compound **4gg**.

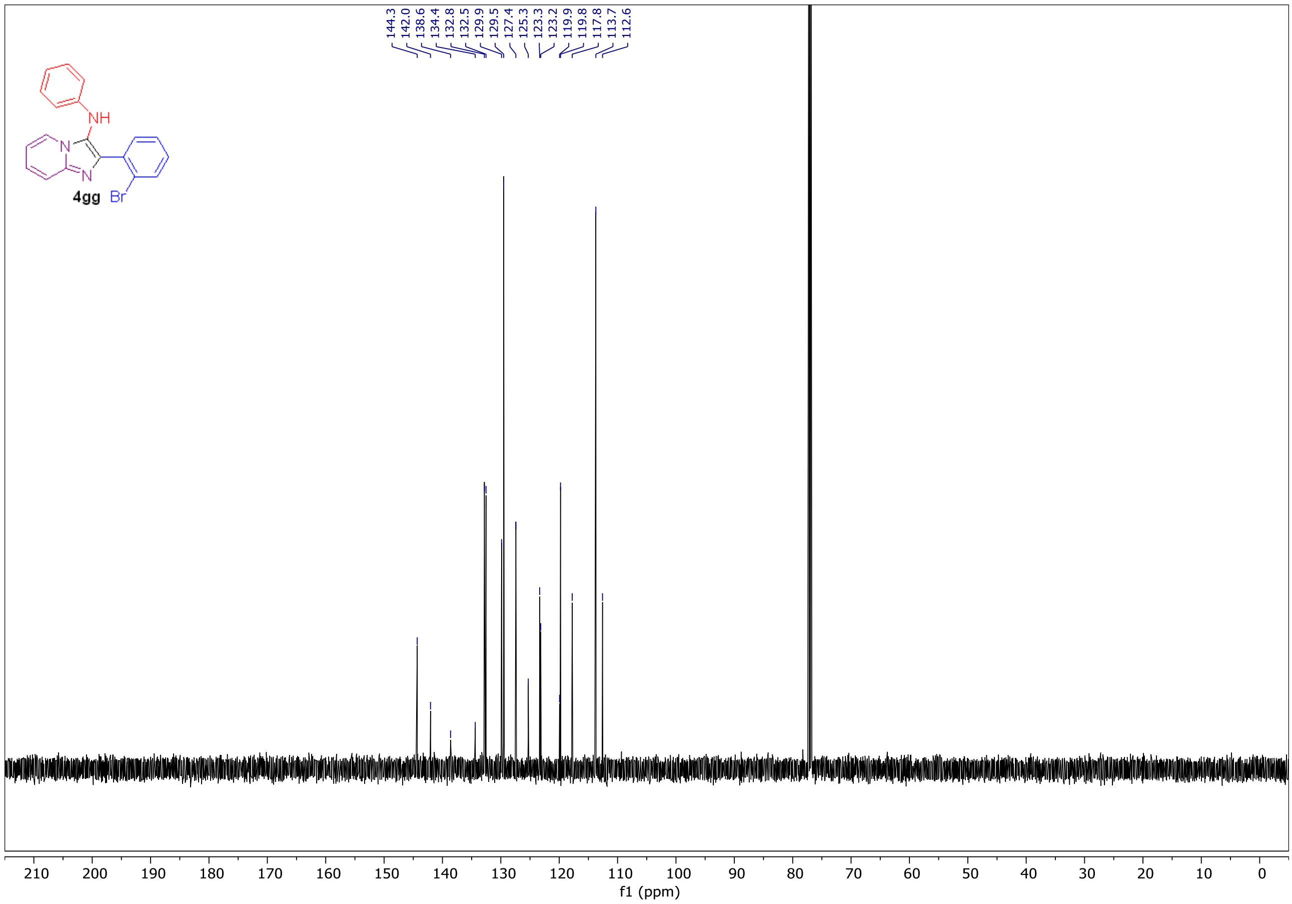

S 131. ^{13}C NMR spectrum (151 MHz, $CDCl_3$) of compound **4gg**.

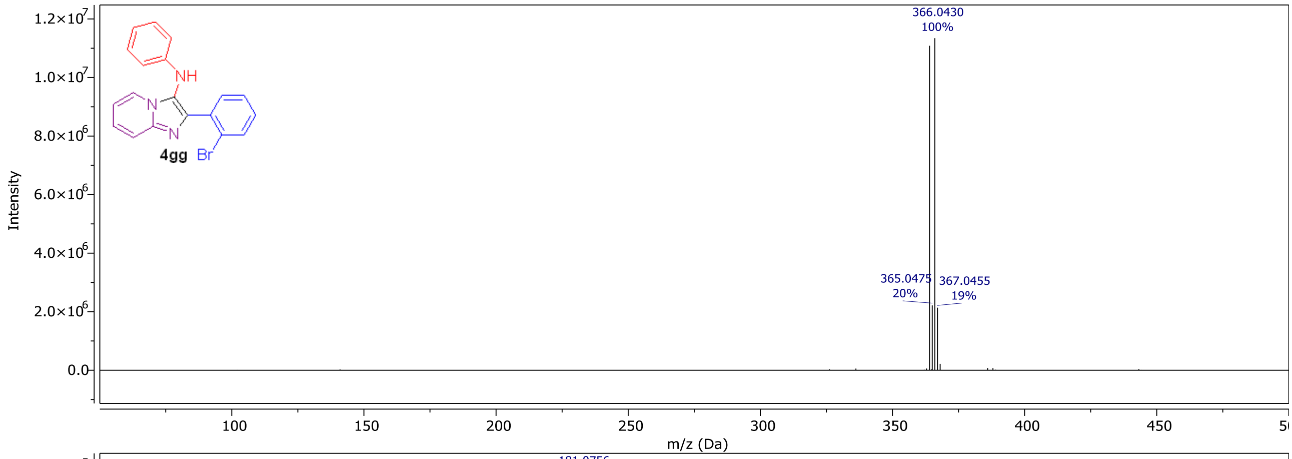

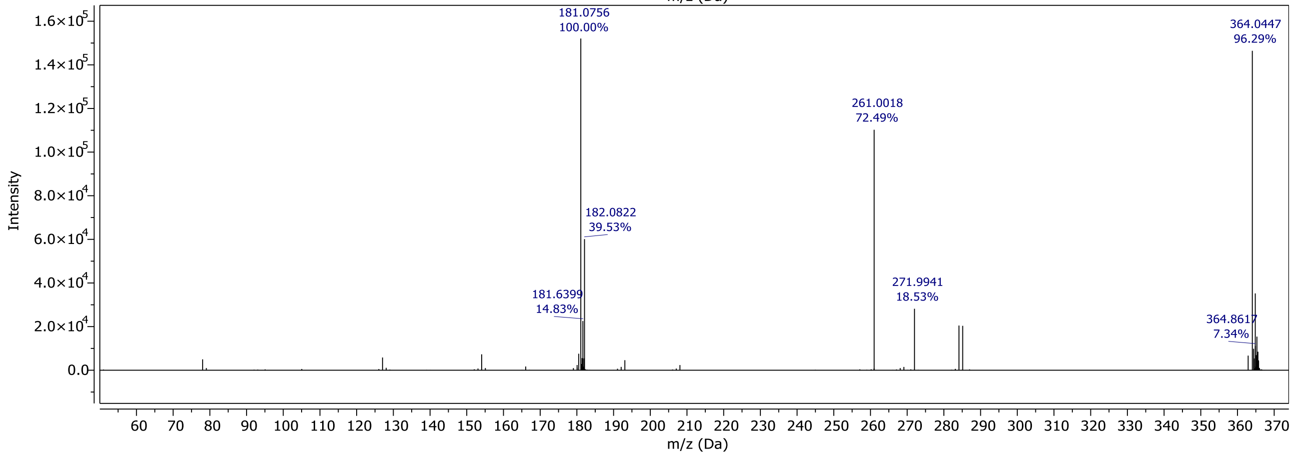

S 132. HRMS (ESI-QTOF) of compound **4gg** and HRMS/MS for [M+H]$^+$.

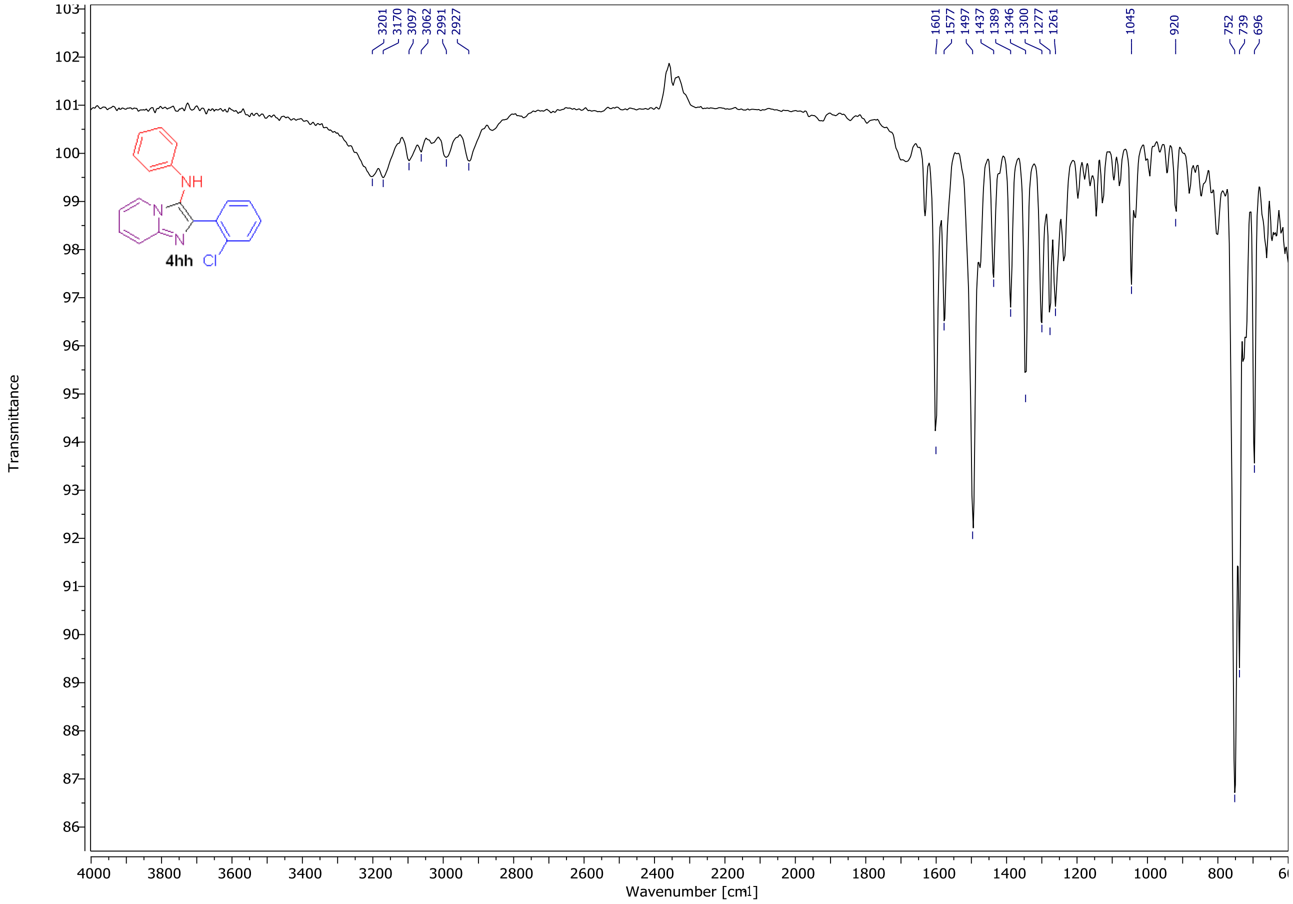

S 133. FT-IR (ATR) of compound **4hh**.

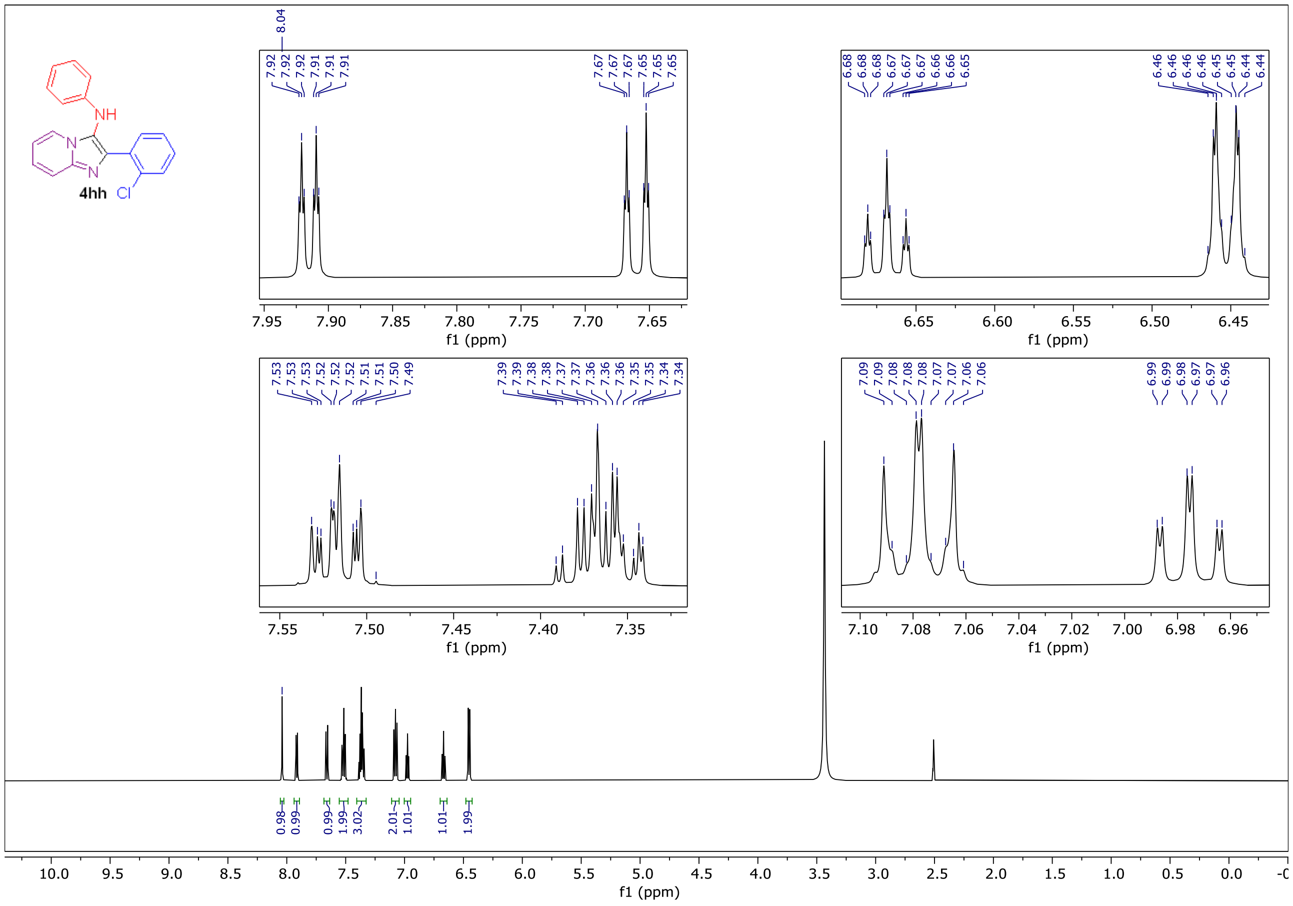

S 134. ^{1}H NMR spectrum (600 MHz, DMSO-d_6) of compound **4hh**.

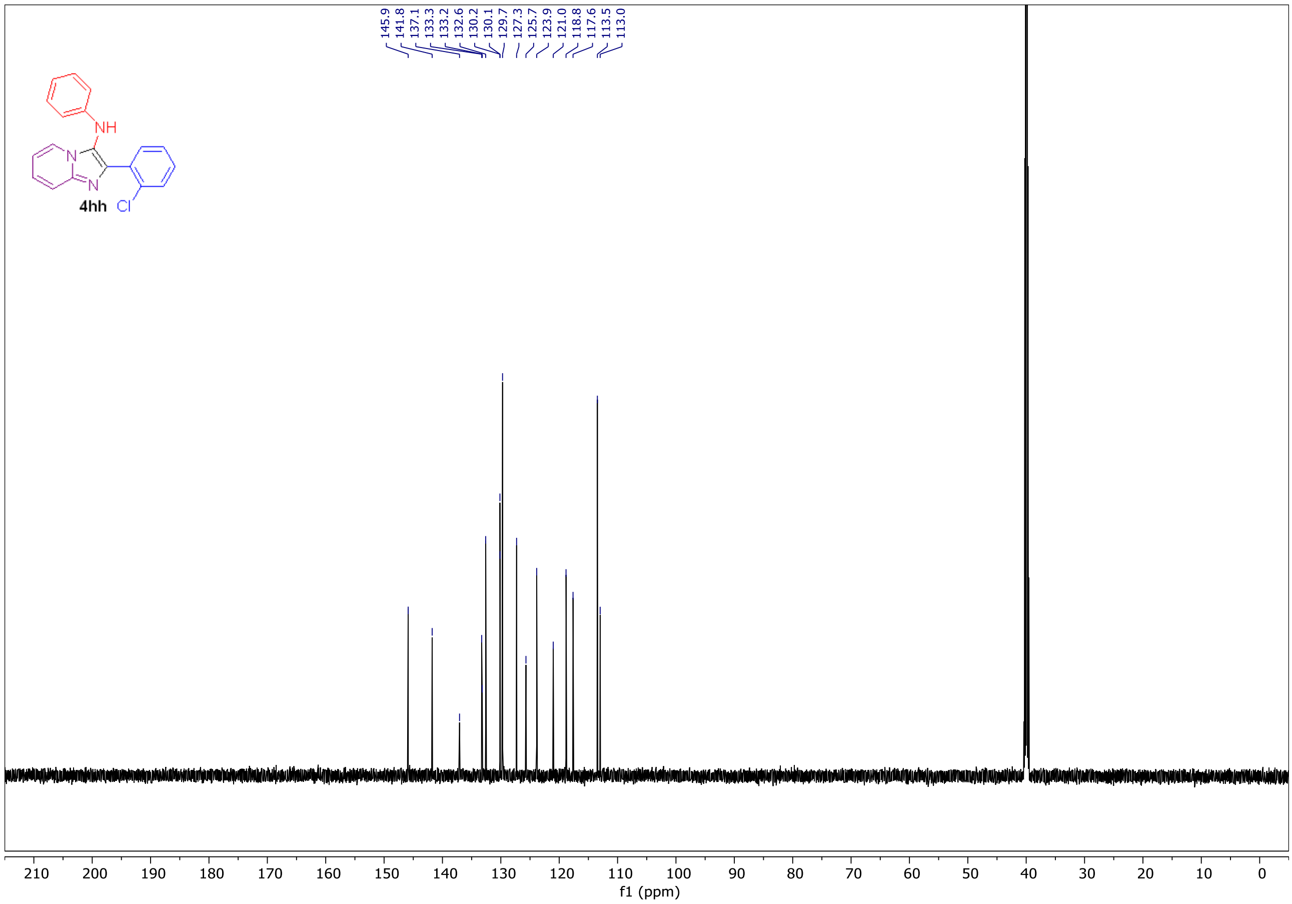

S 135. ^{13}C NMR spectrum (151 MHz, DMSO-d_6) of compound **4hh**.

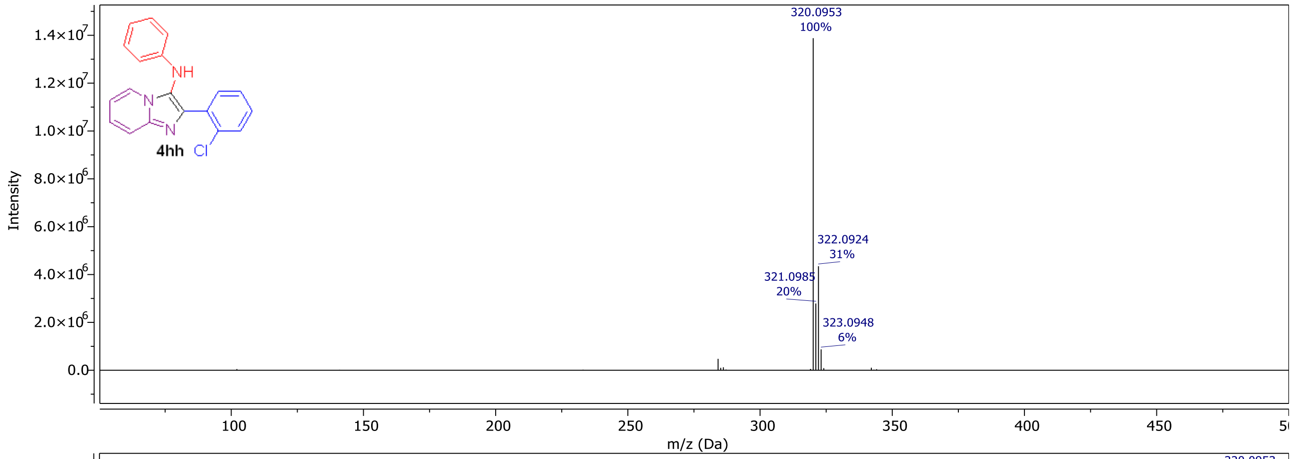

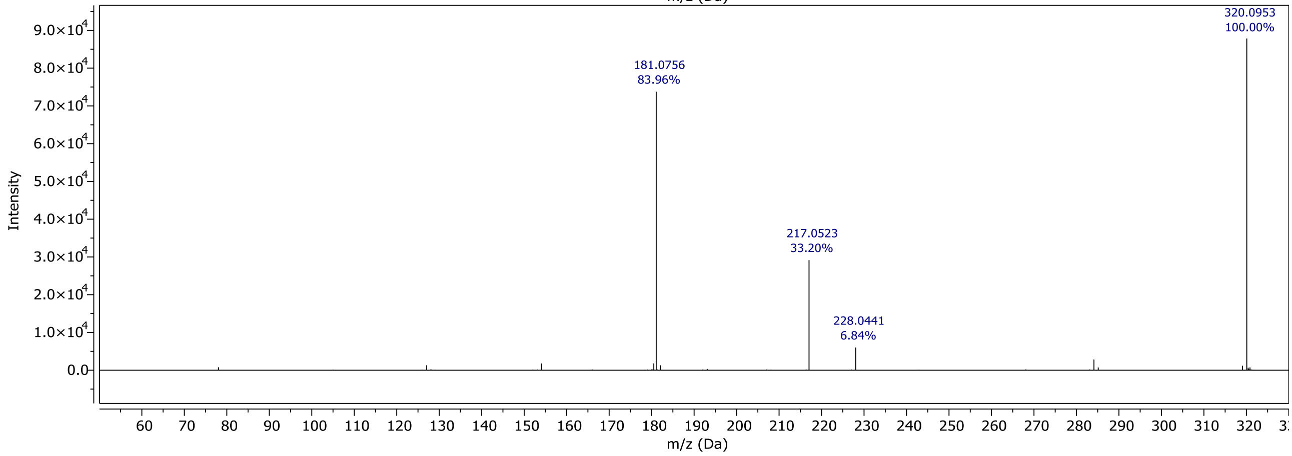

S 136. HRMS (ESI-QTOF) of compound **4hh** and HRMS/MS for [M+H]$^+$.

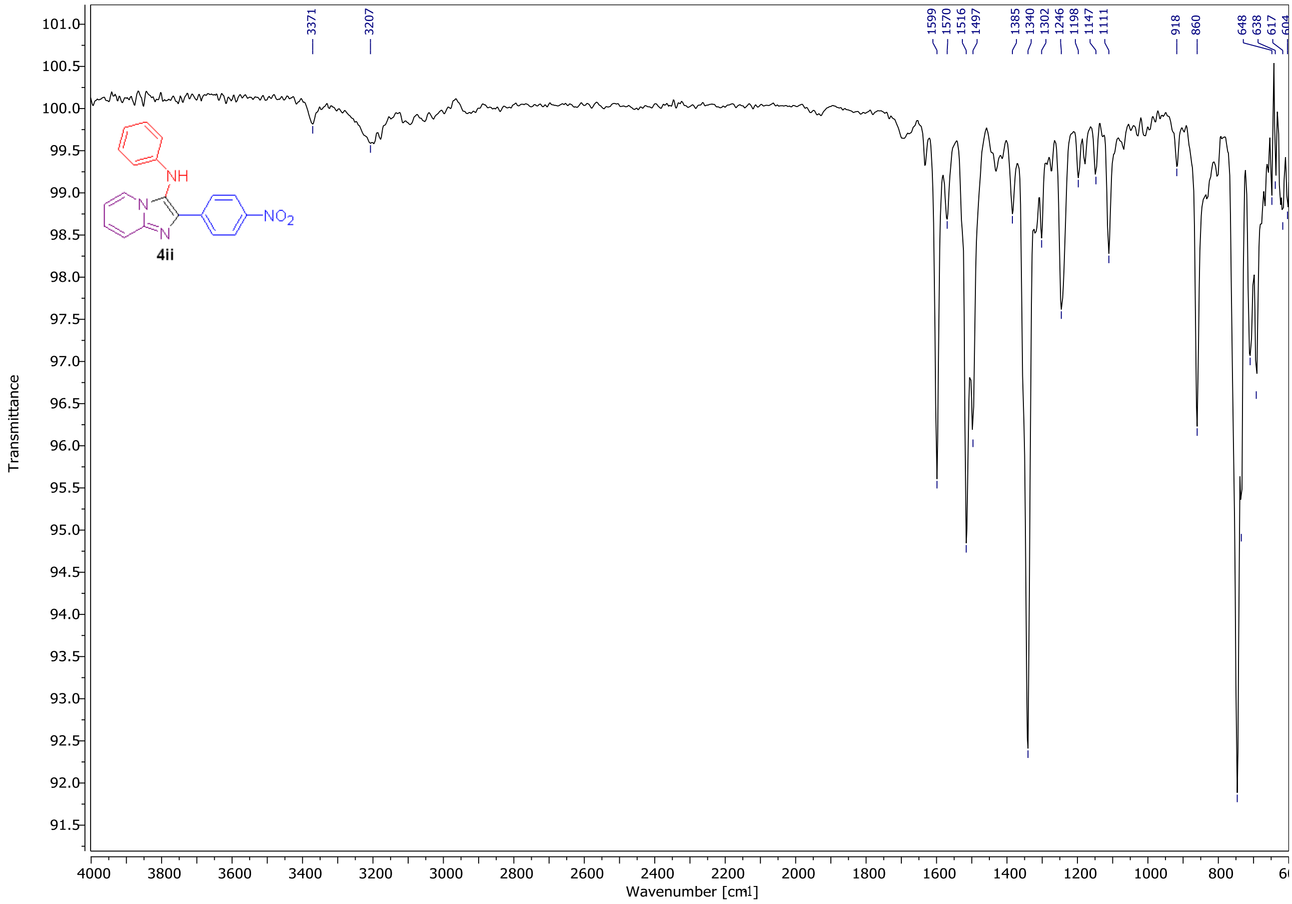

S 137. FT-IR (ATR) of compound **4ii**.

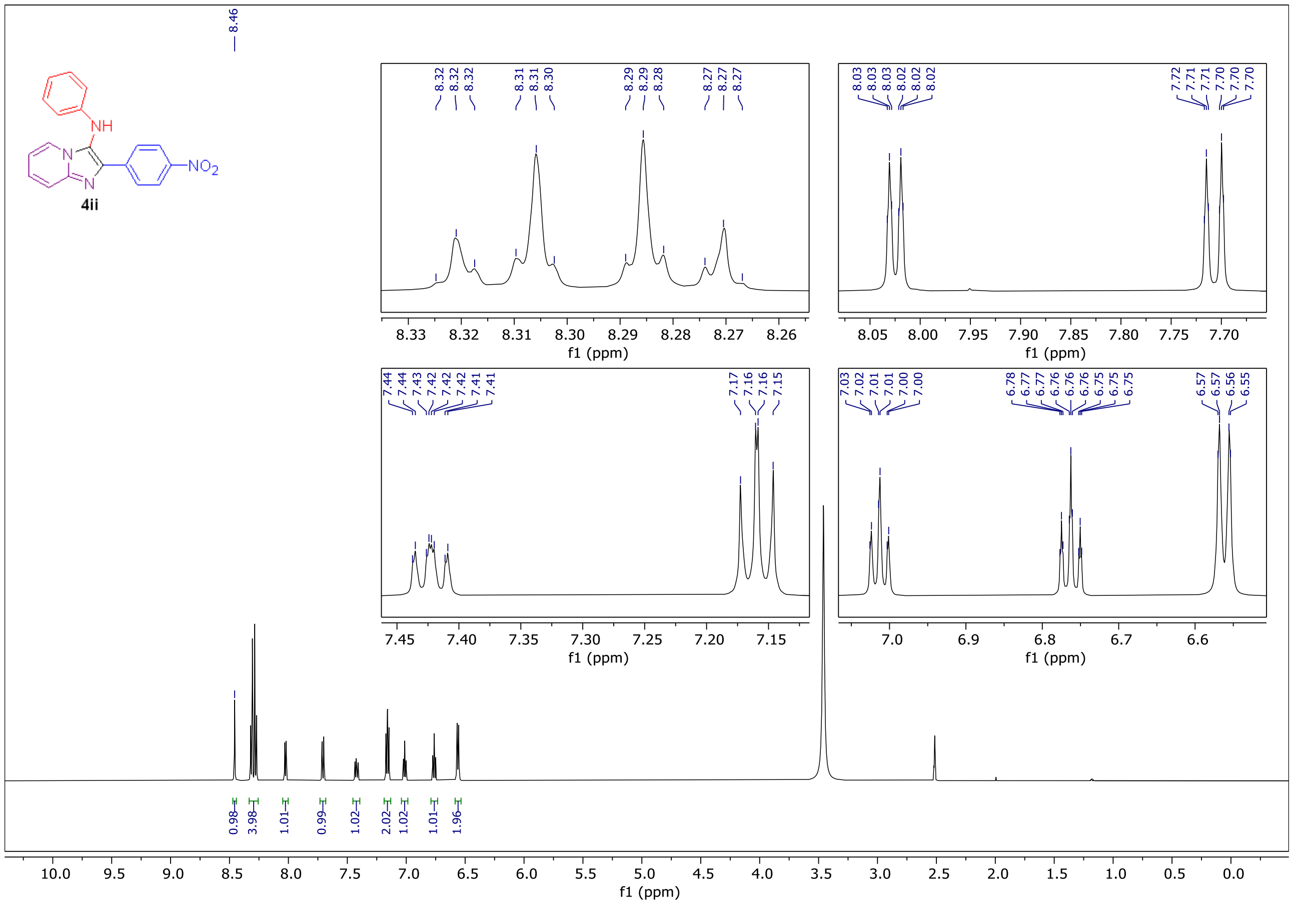

S 138. ^{1}H NMR spectrum (600 MHz, DMSO-d_6) of compound **4ii**.

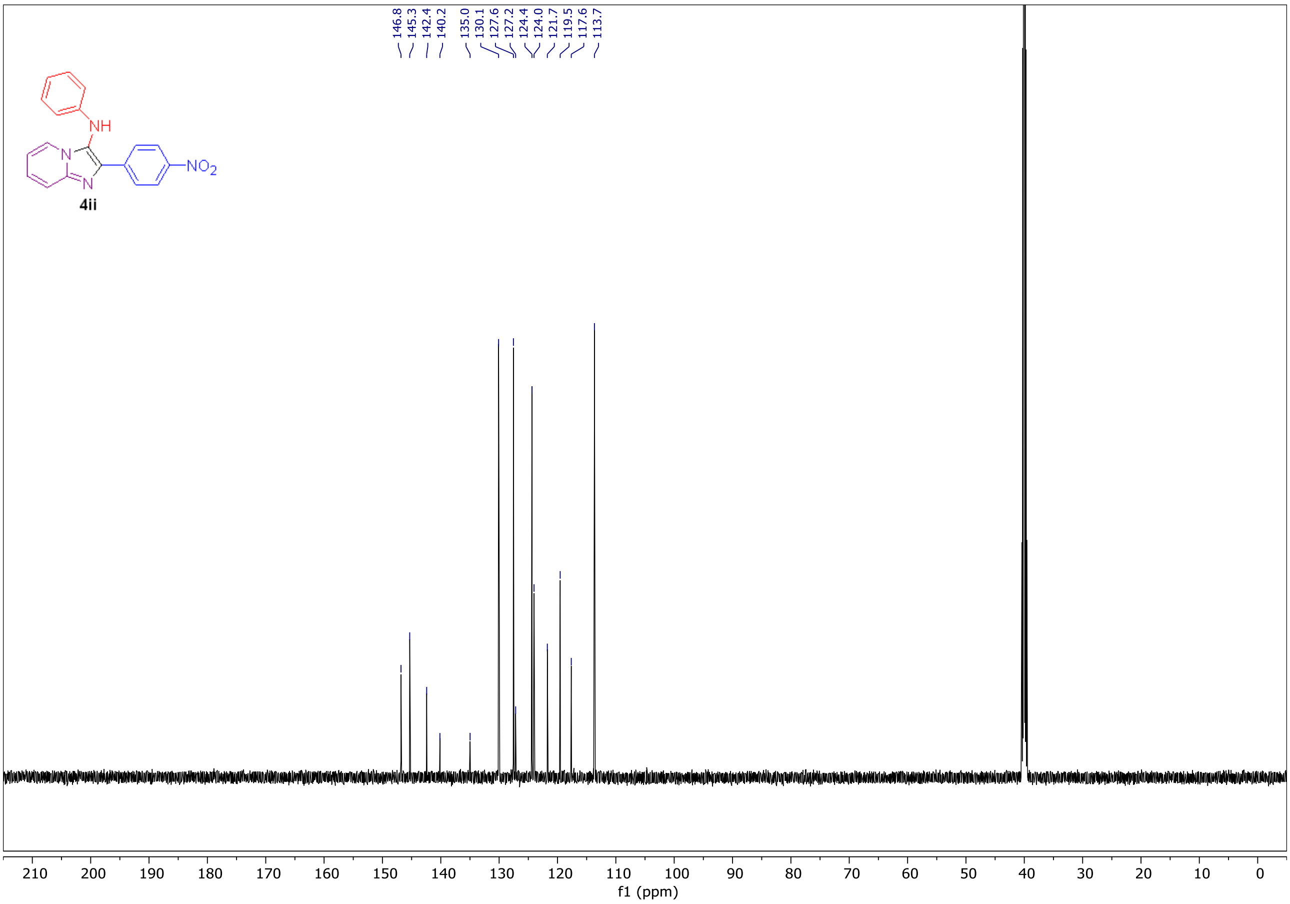

S 139. ^{13}C NMR spectrum (151 MHz, DMSO-d_6) of compound **4ii**.

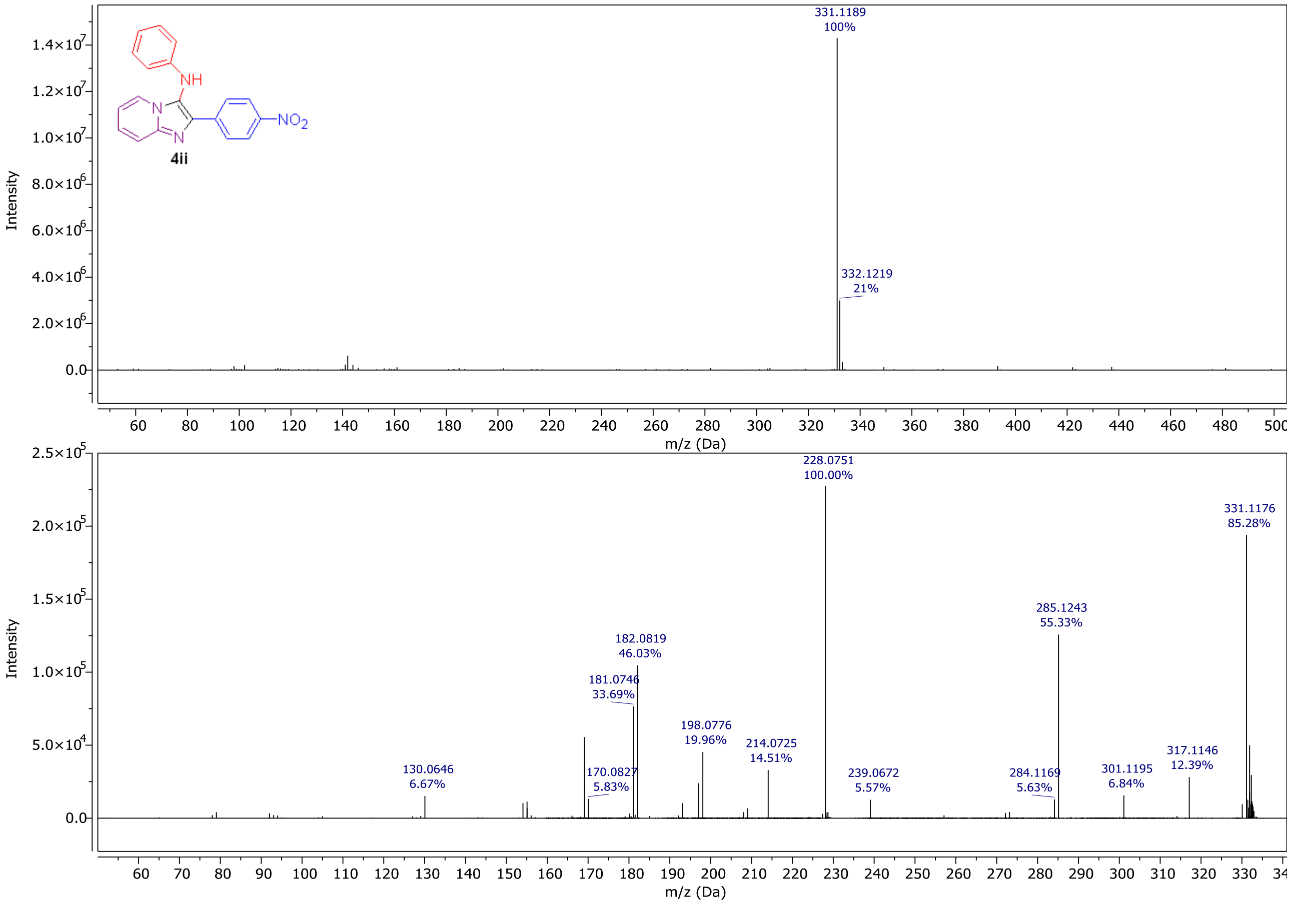

S 140. HRMS (ESI-QTOF) of compound **4ii** and HRMS/MS for [M+H]$^+$.

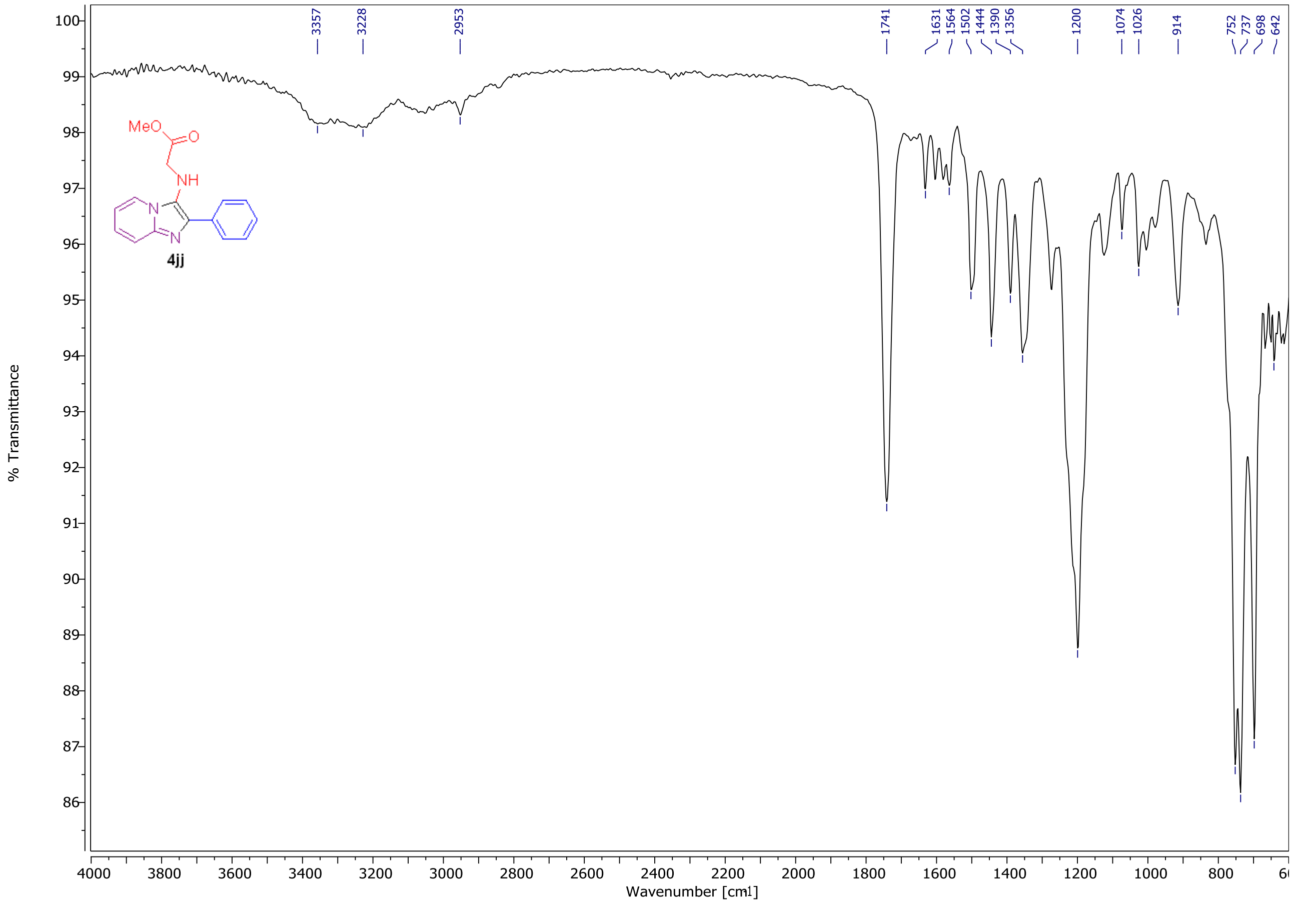

S 141. FT-IR (ATR) of compound **4jj**.

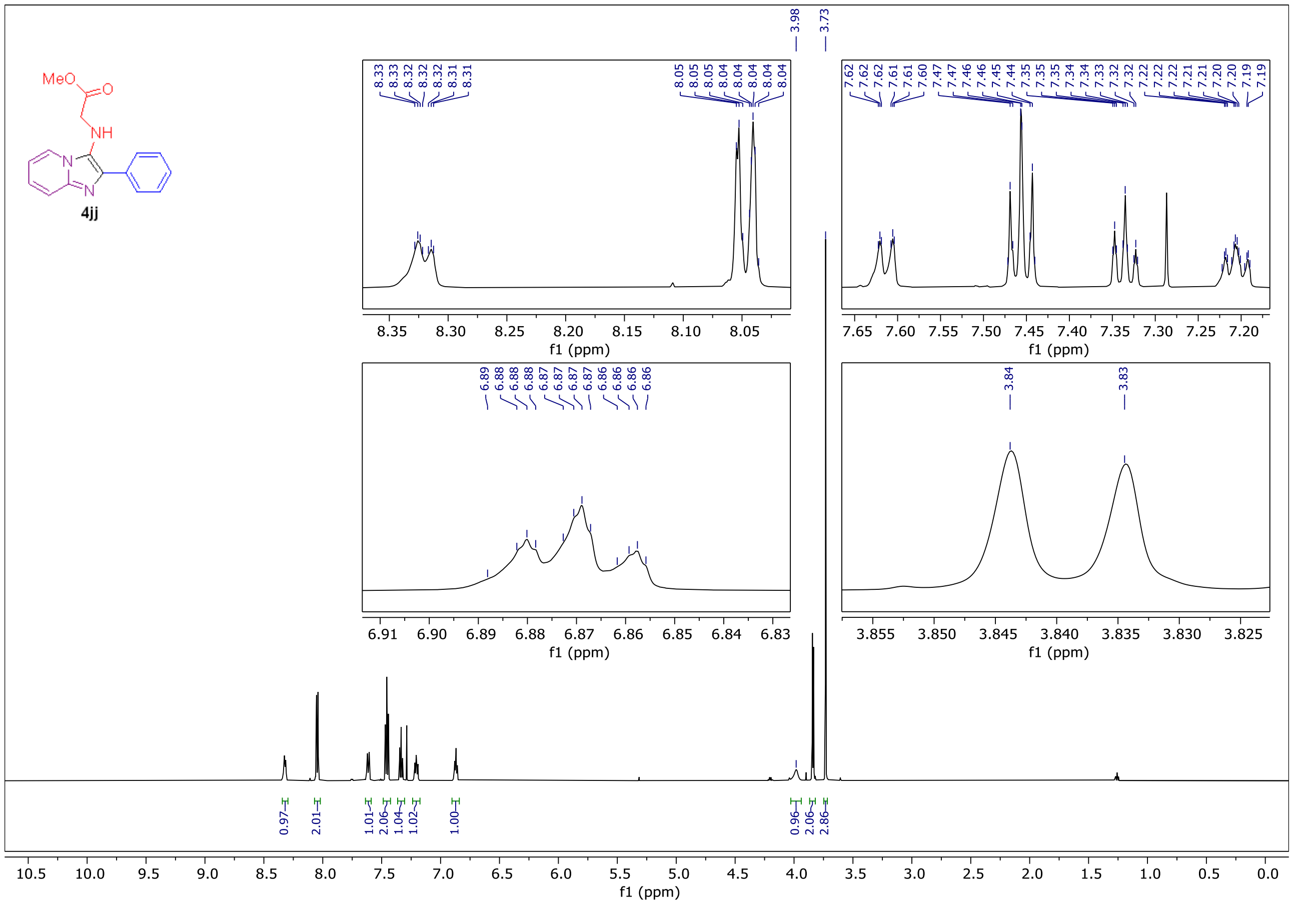

S 142. ^{1}H NMR spectrum (600 MHz, $CDCl_3$) of compound **4jj**.

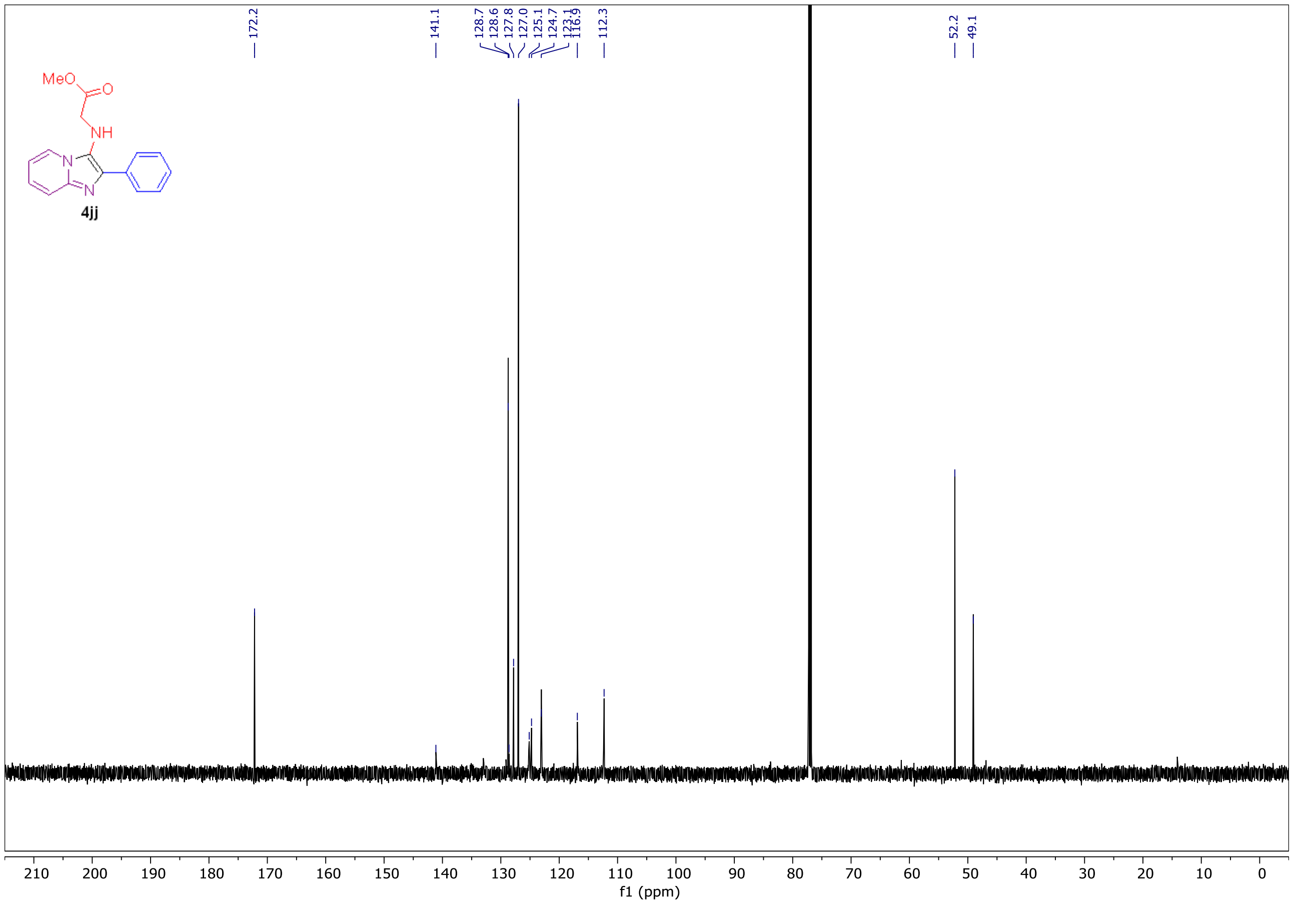

S 143. ^{13}C NMR spectrum (151 MHz, $CDCl_3$) of compound **4jj**.

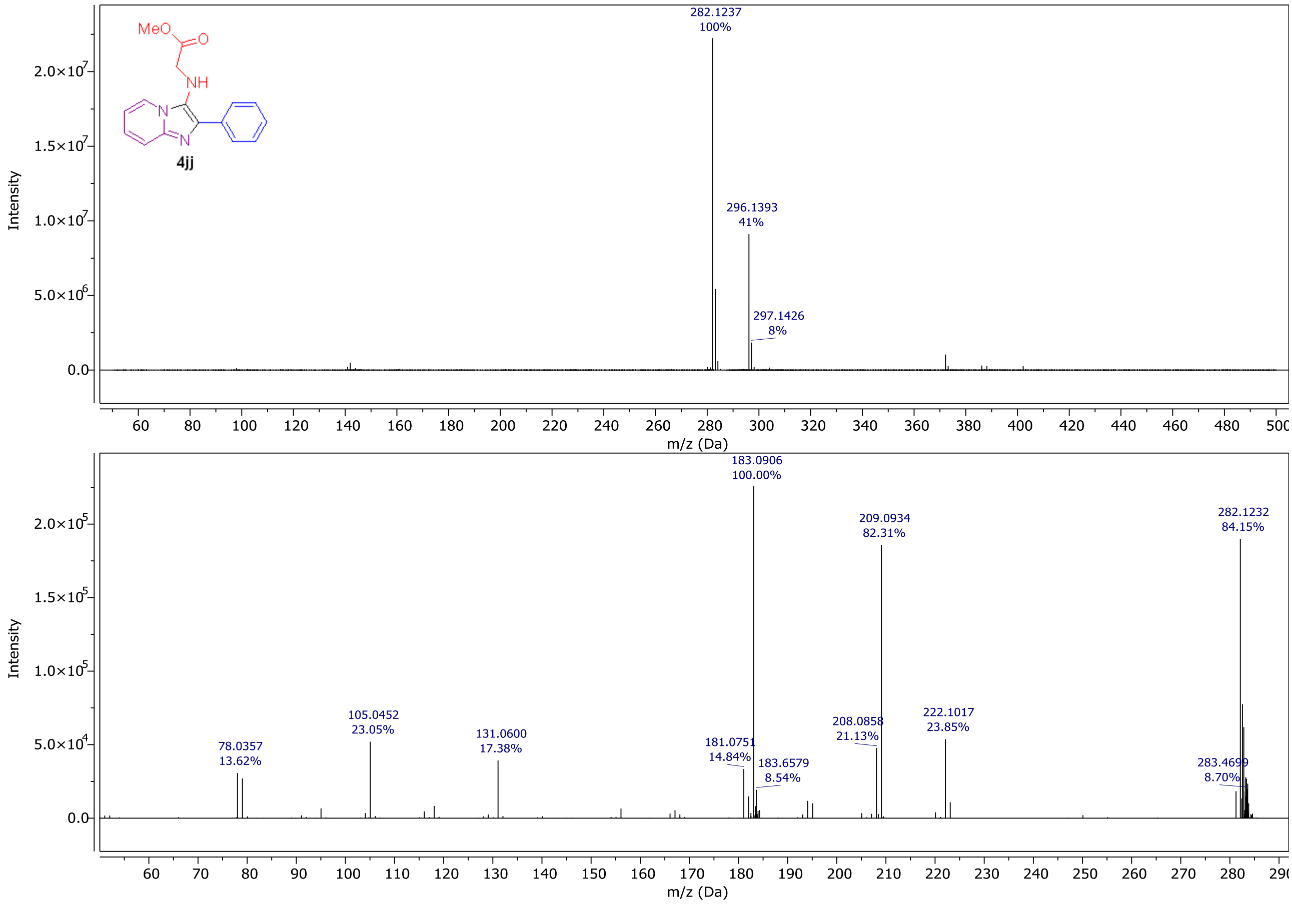

S 144. HRMS (ESI-QTOF) of compound **4jj** and HRMS/MS for [M+H]$^+$.

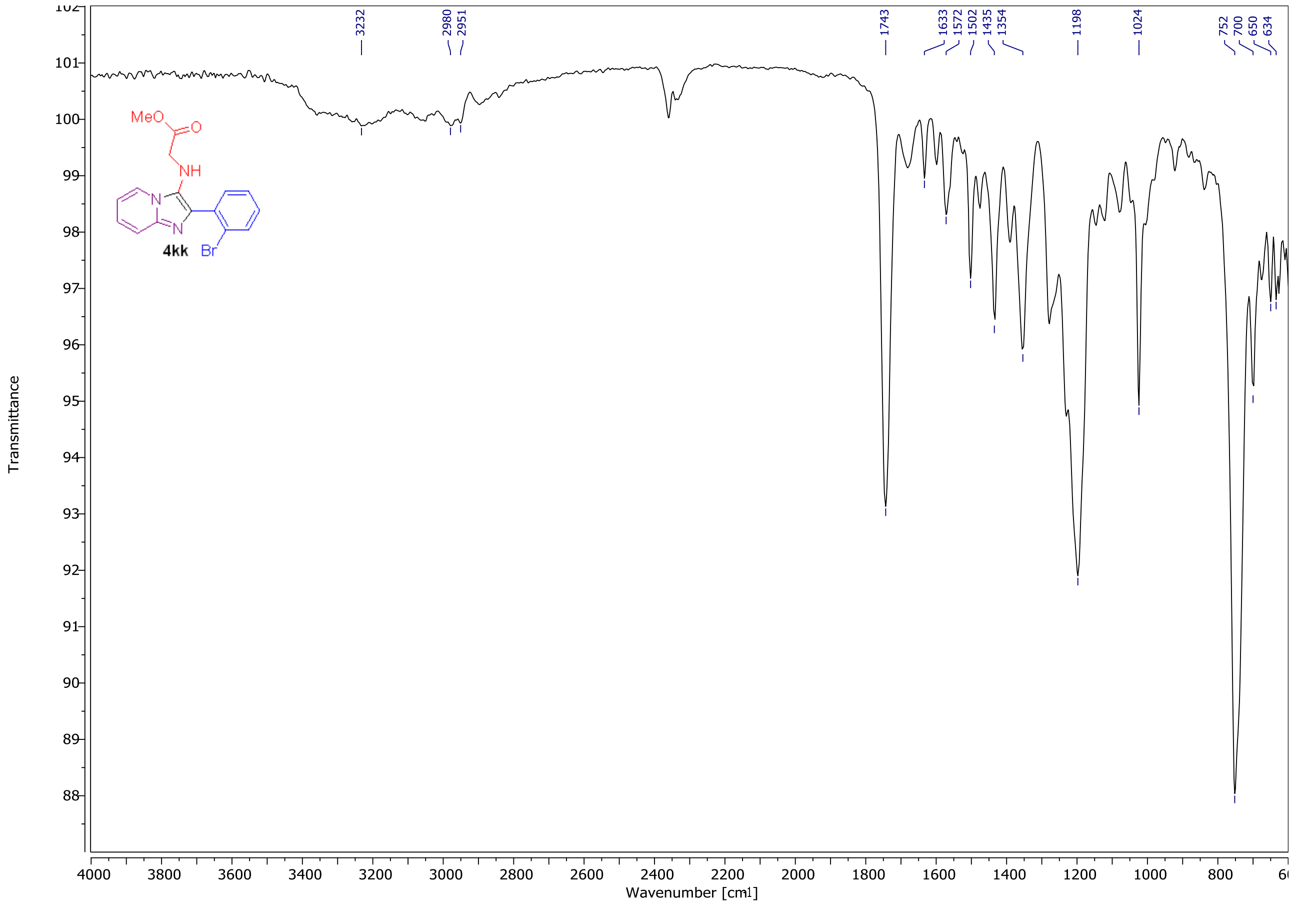

S 145. FT-IR (ATR) of compound **4kk**.

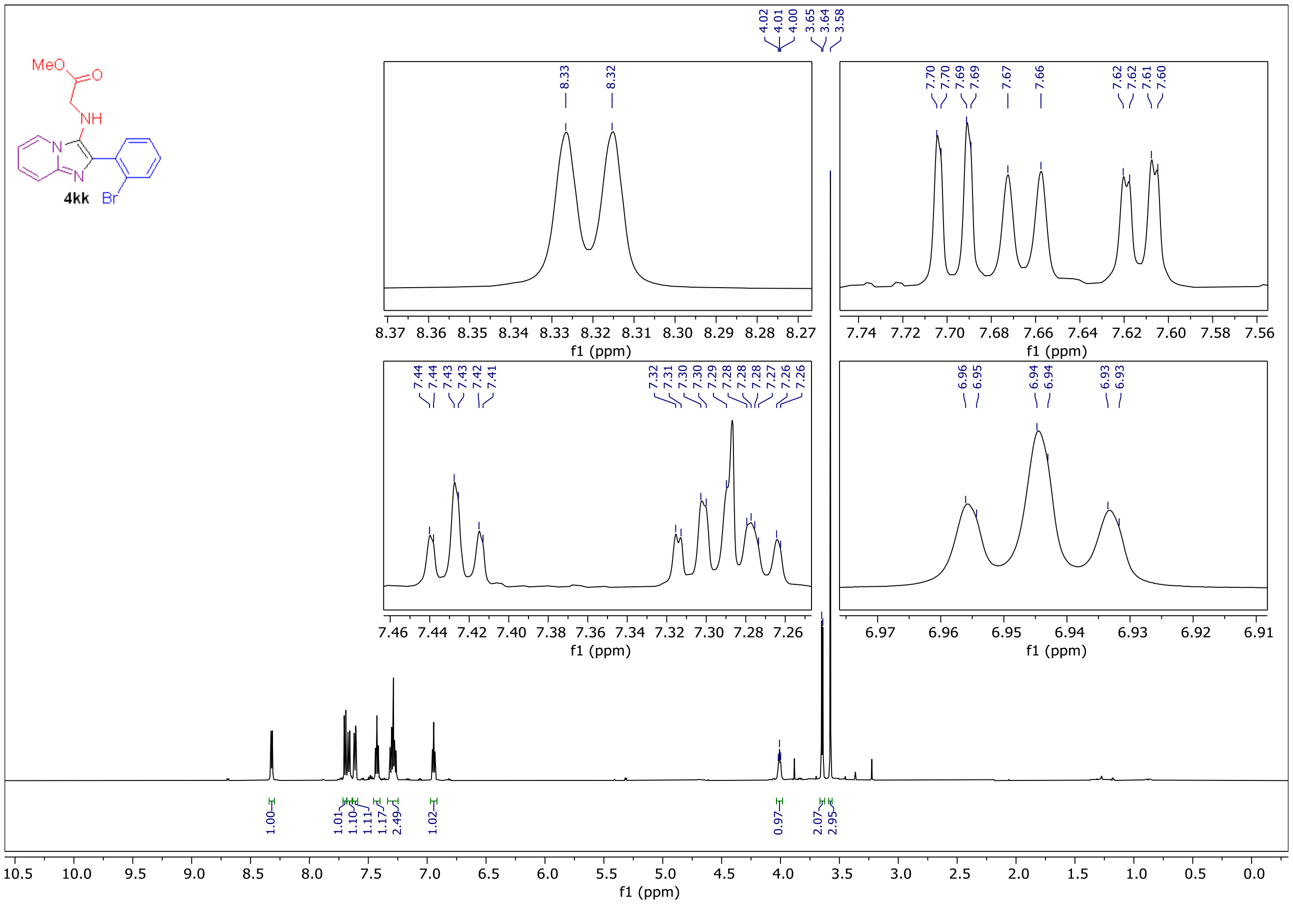

S 146. ^{1}H NMR spectrum (600 MHz, $CDCl_3$) of compound **4kk**.

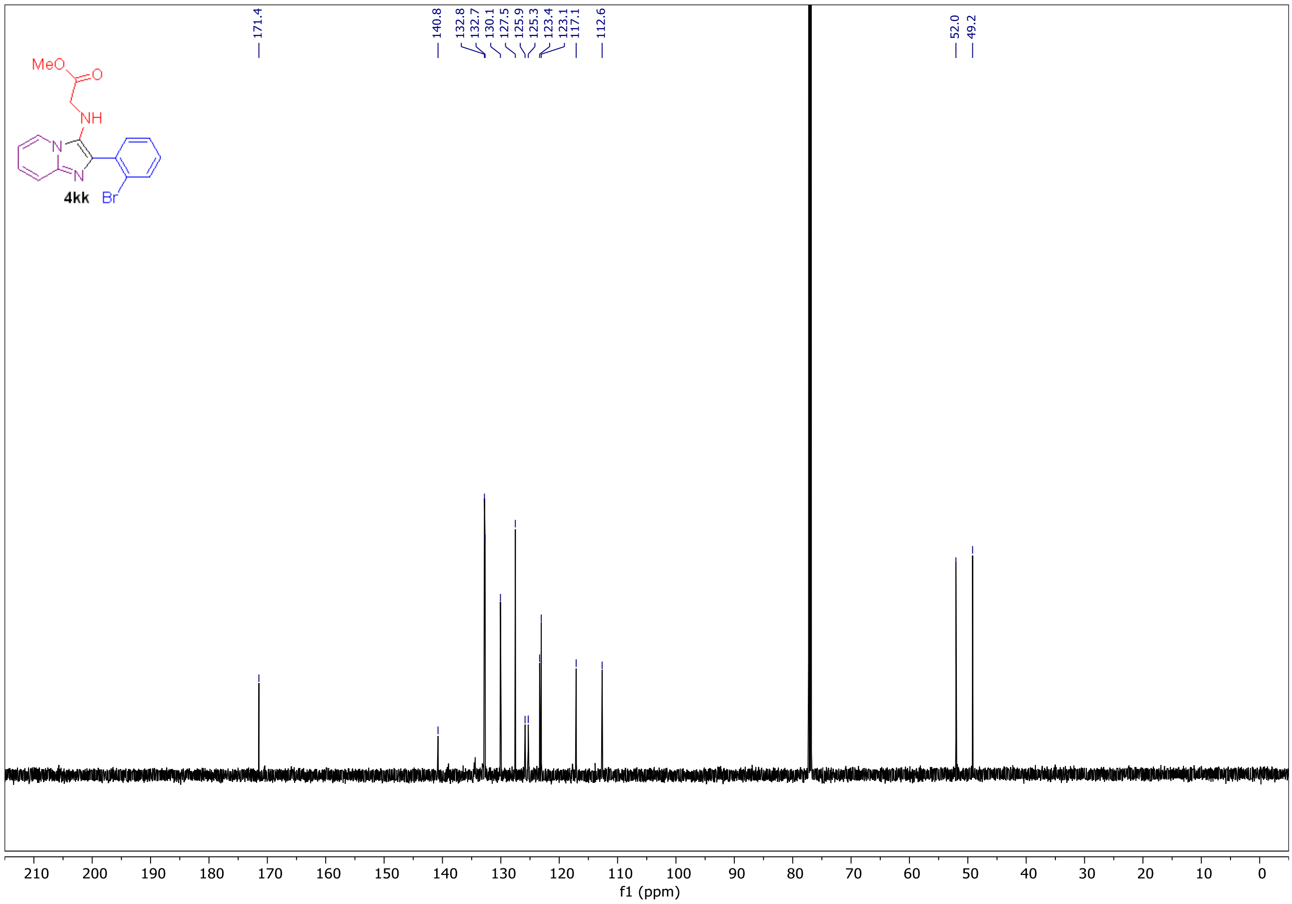

S 147. ^{13}C NMR spectrum (151 MHz, $CDCl_3$) of compound **4kk**.

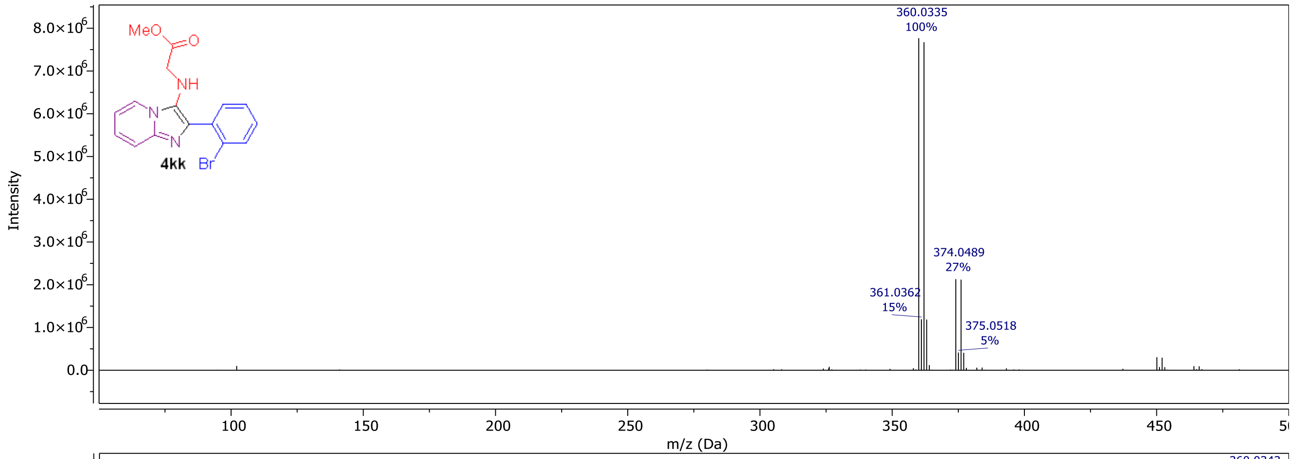

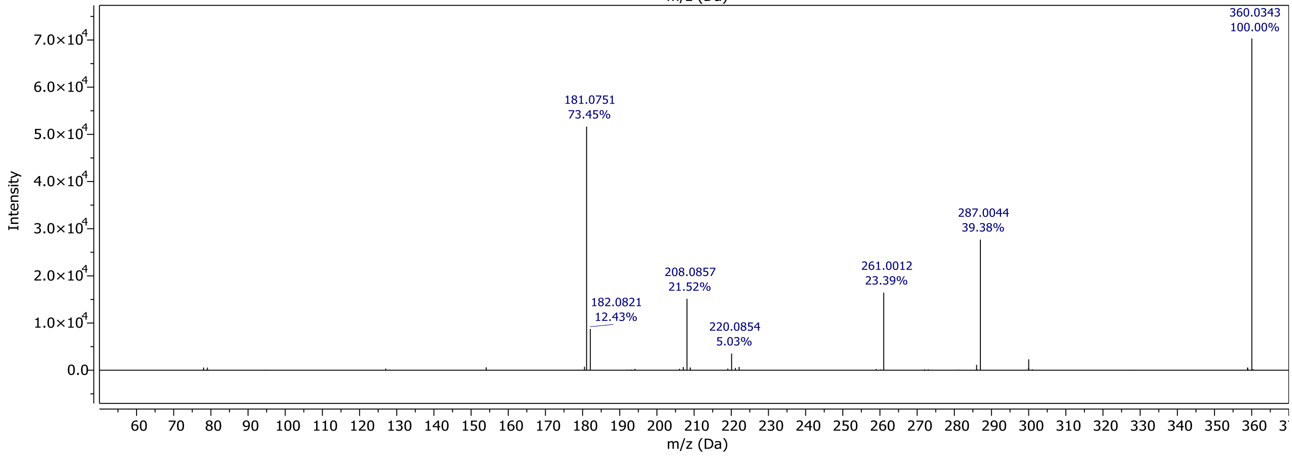

S 148. HRMS (ESI-QTOF) of compound **4kk** and HRMS/MS for $[M+H]^+$.

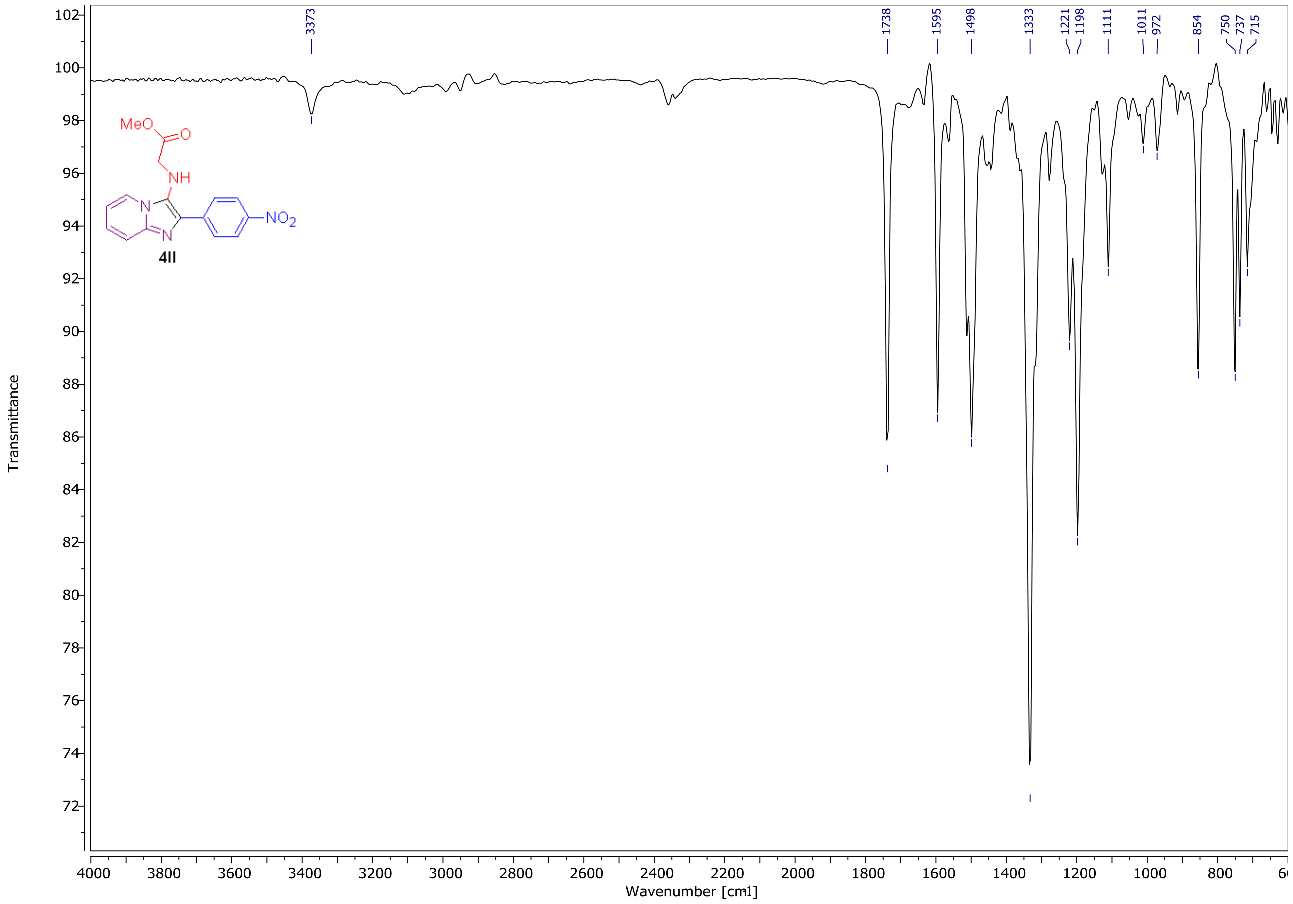

S 149. FT-IR (ATR) of compound **4ll**.

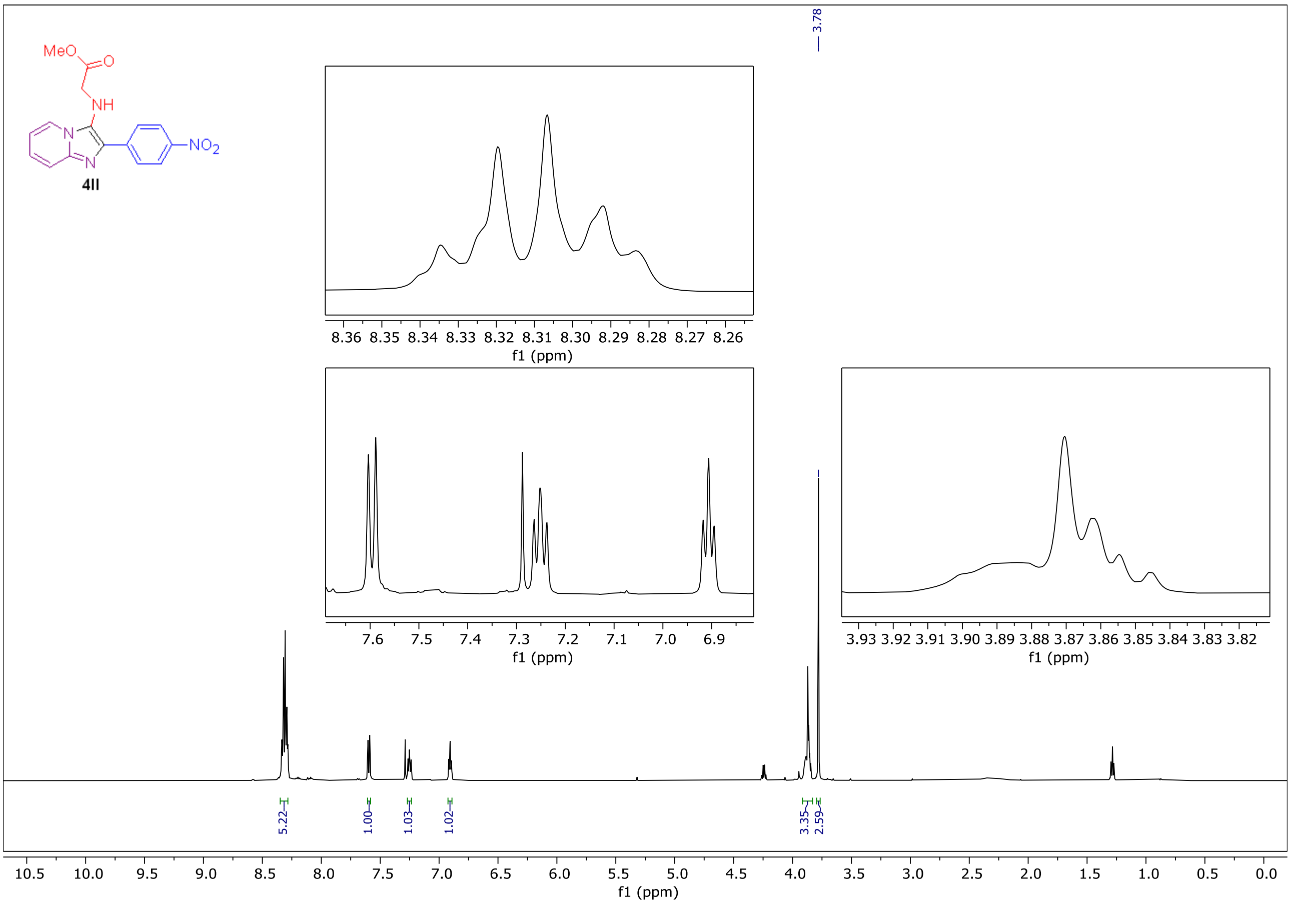

S 150. ^{1}H NMR spectrum (600 MHz, $CDCl_3$) of compound **4ll**.

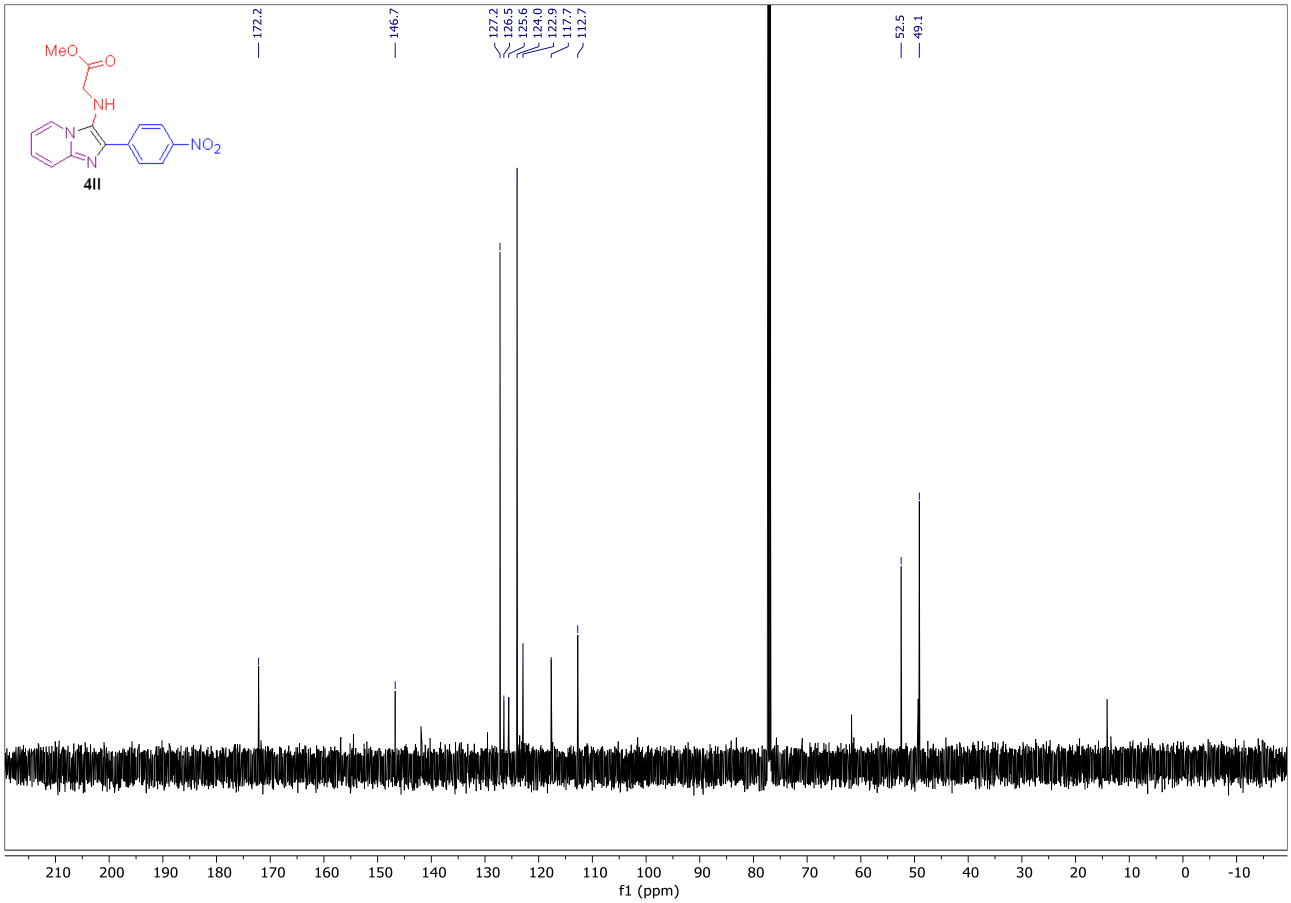

S 151. ^{13}C NMR spectrum (151 MHz, $CDCl_3$) of compound **4ll**.

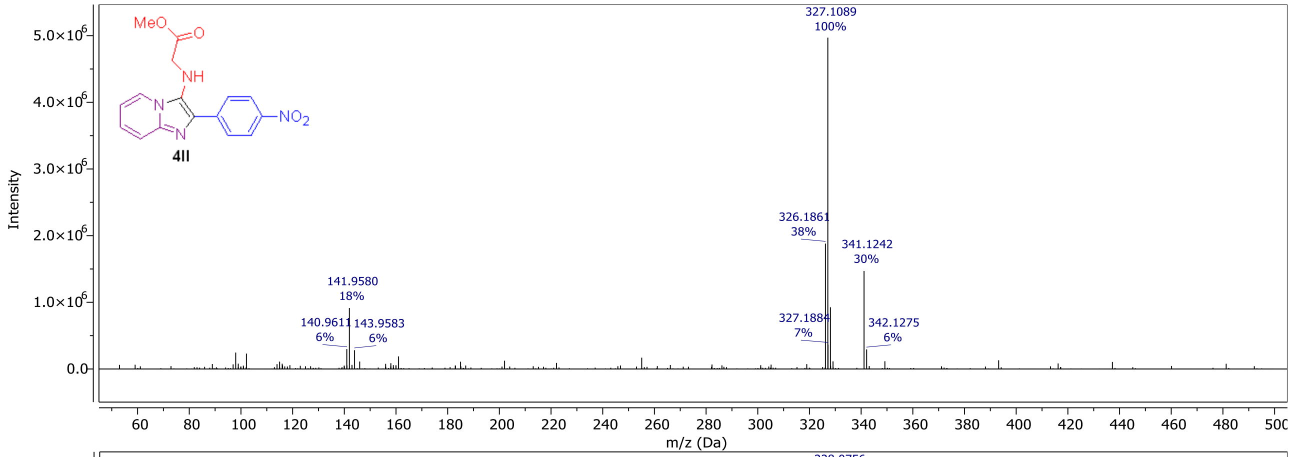

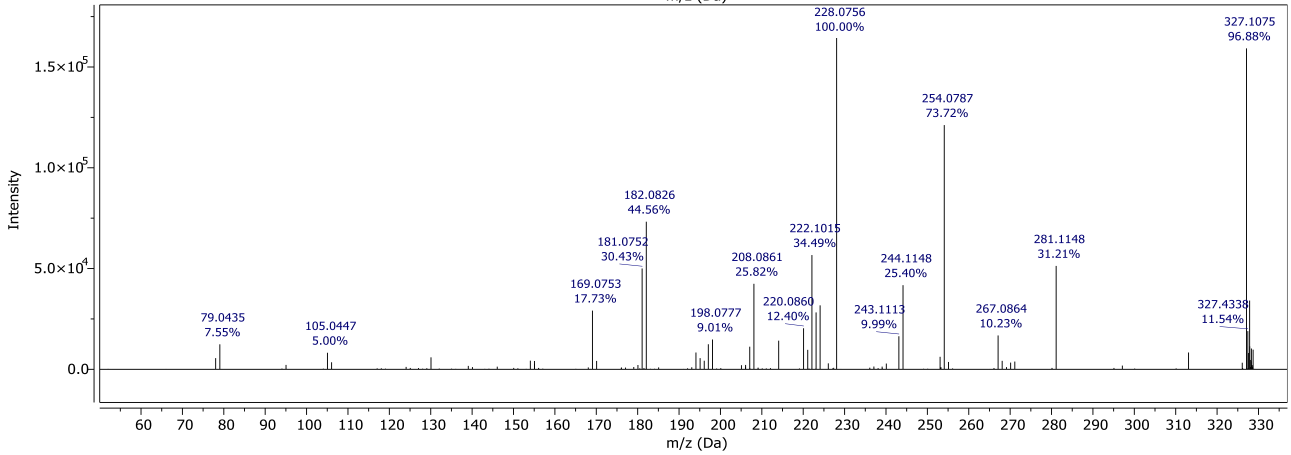

S 152. HRMS (ESI-QTOF) of compound **4ll** and HRMS/MS for $[M+H]^+$.

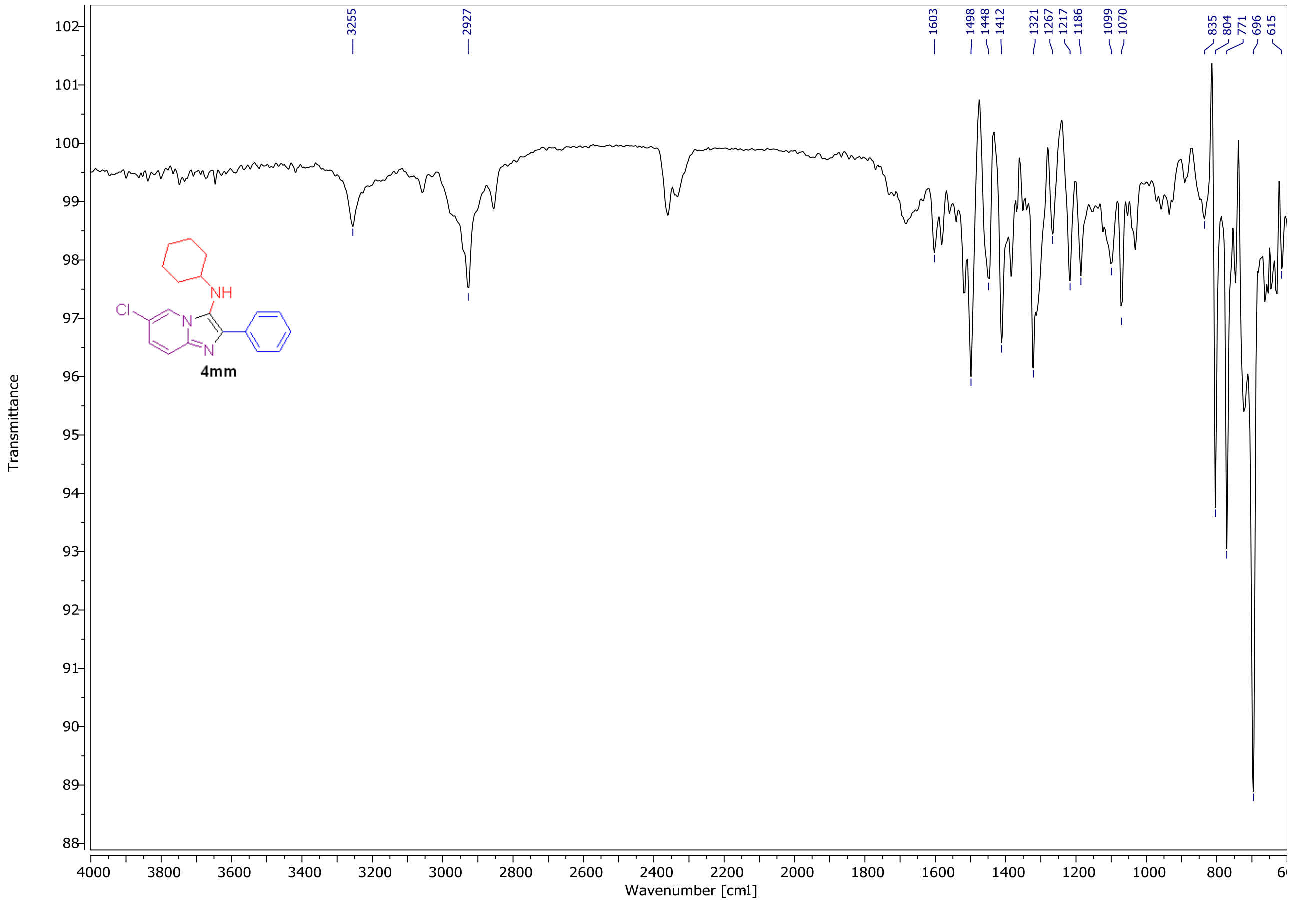

S 153. FT-IR (ATR) of compound **4mm**.

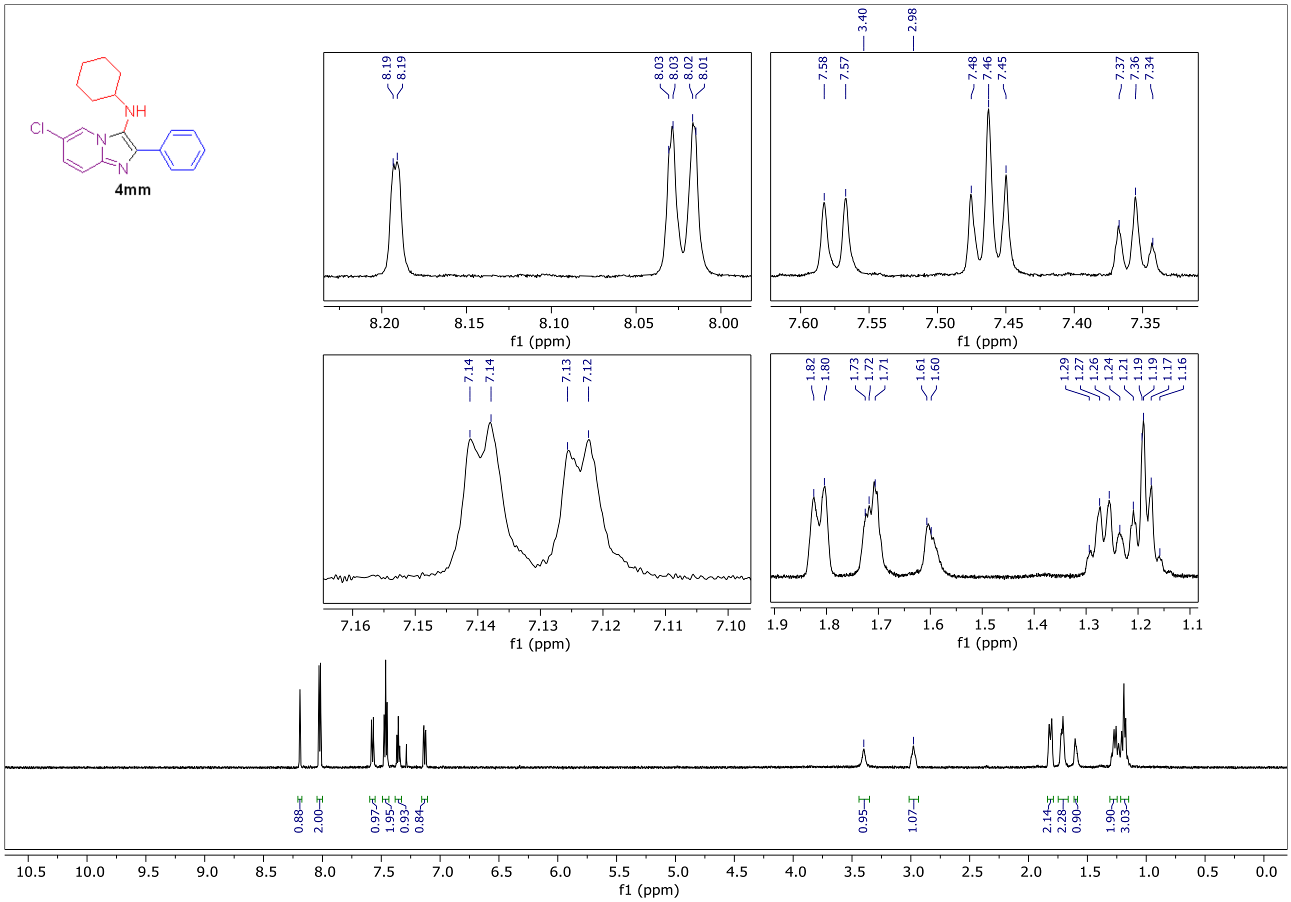

S 154. ^{1}H NMR spectrum (600 MHz, $CDCl_3$) of compound **4mm**.

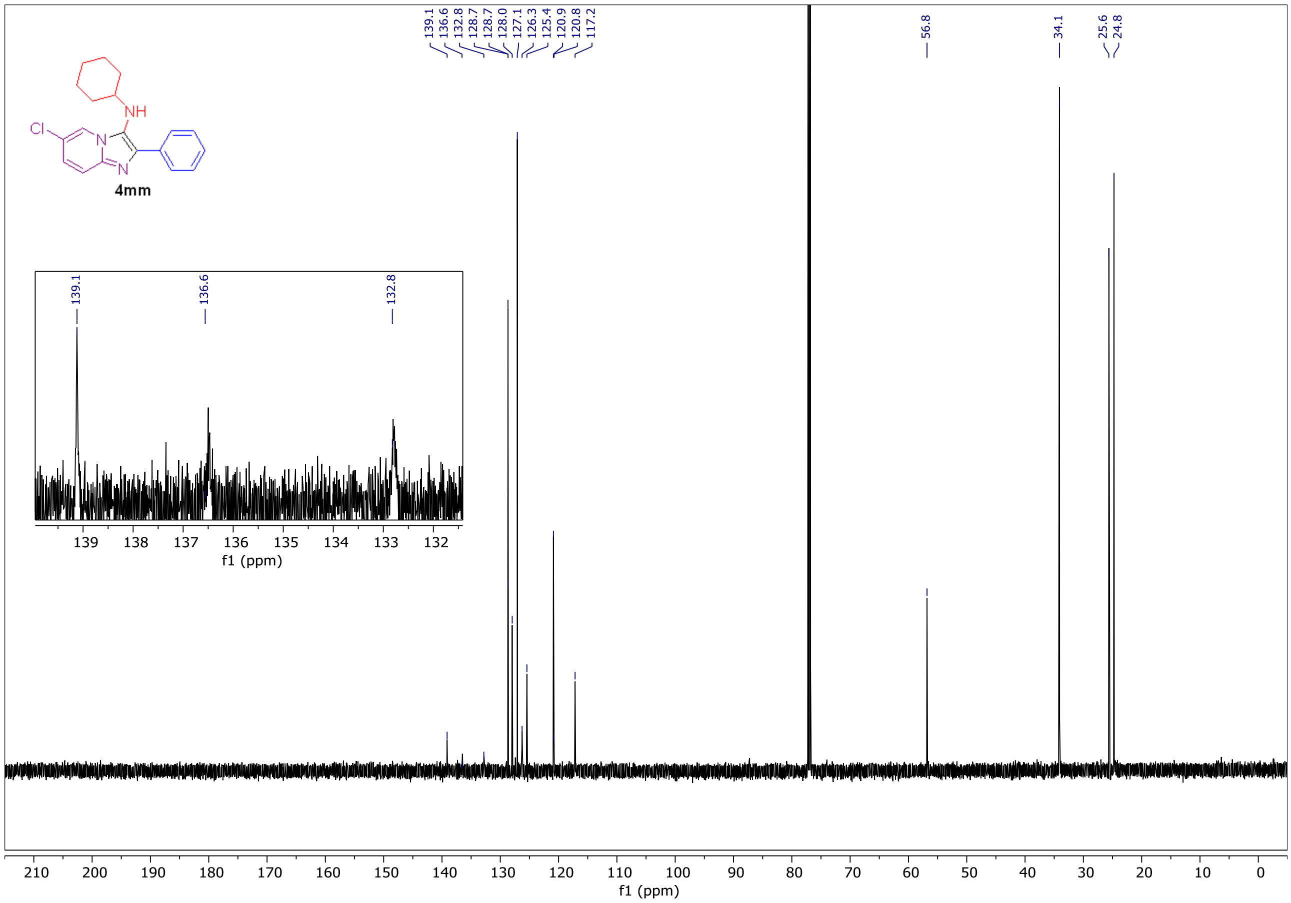

S 155. ^{13}C NMR spectrum (151 MHz, $CDCl_3$) of compound **4mm**.

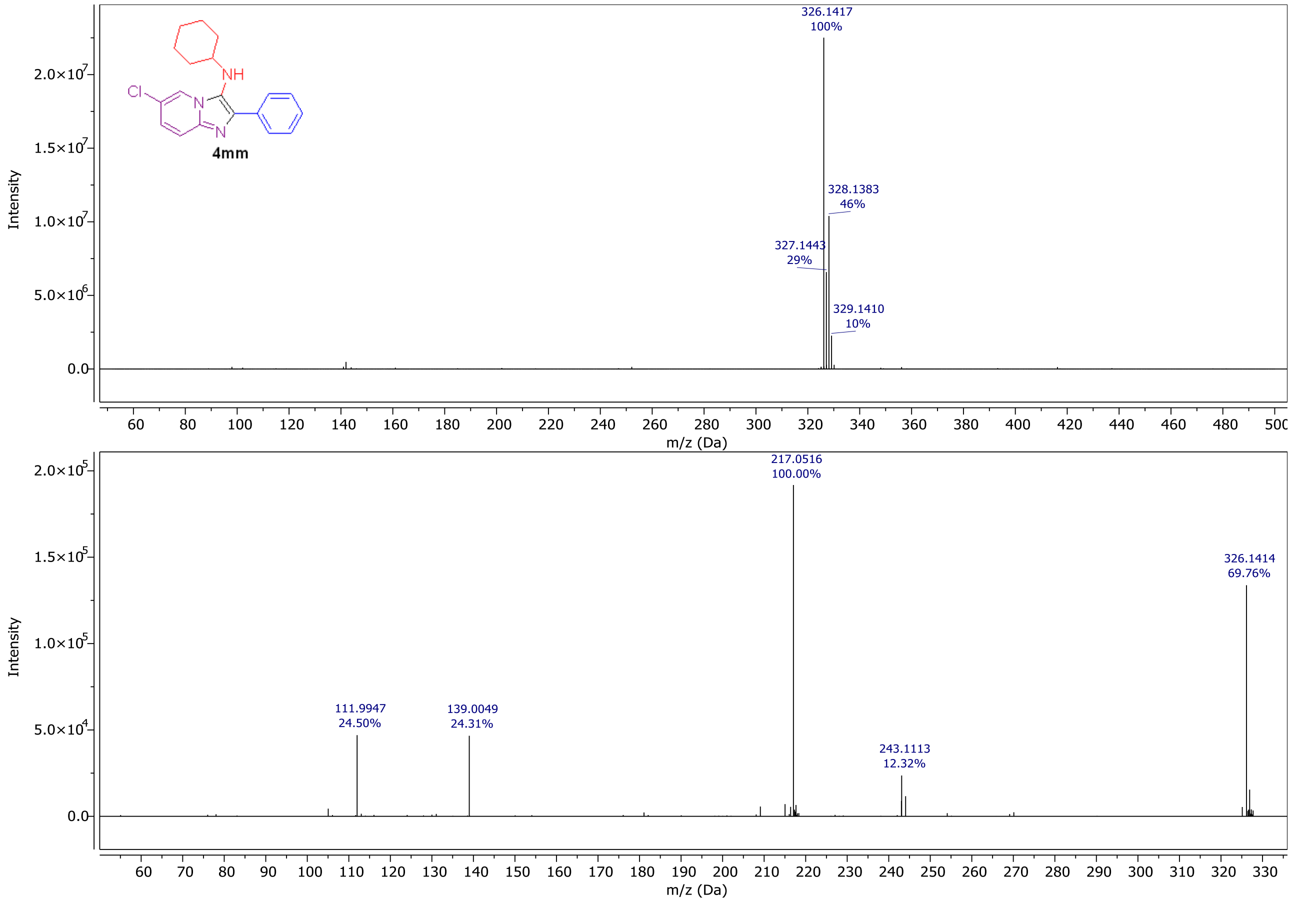

S 156. HRMS (ESI-QTOF) of compound **4mm** and HRMS/MS for $[M+H]^+$.

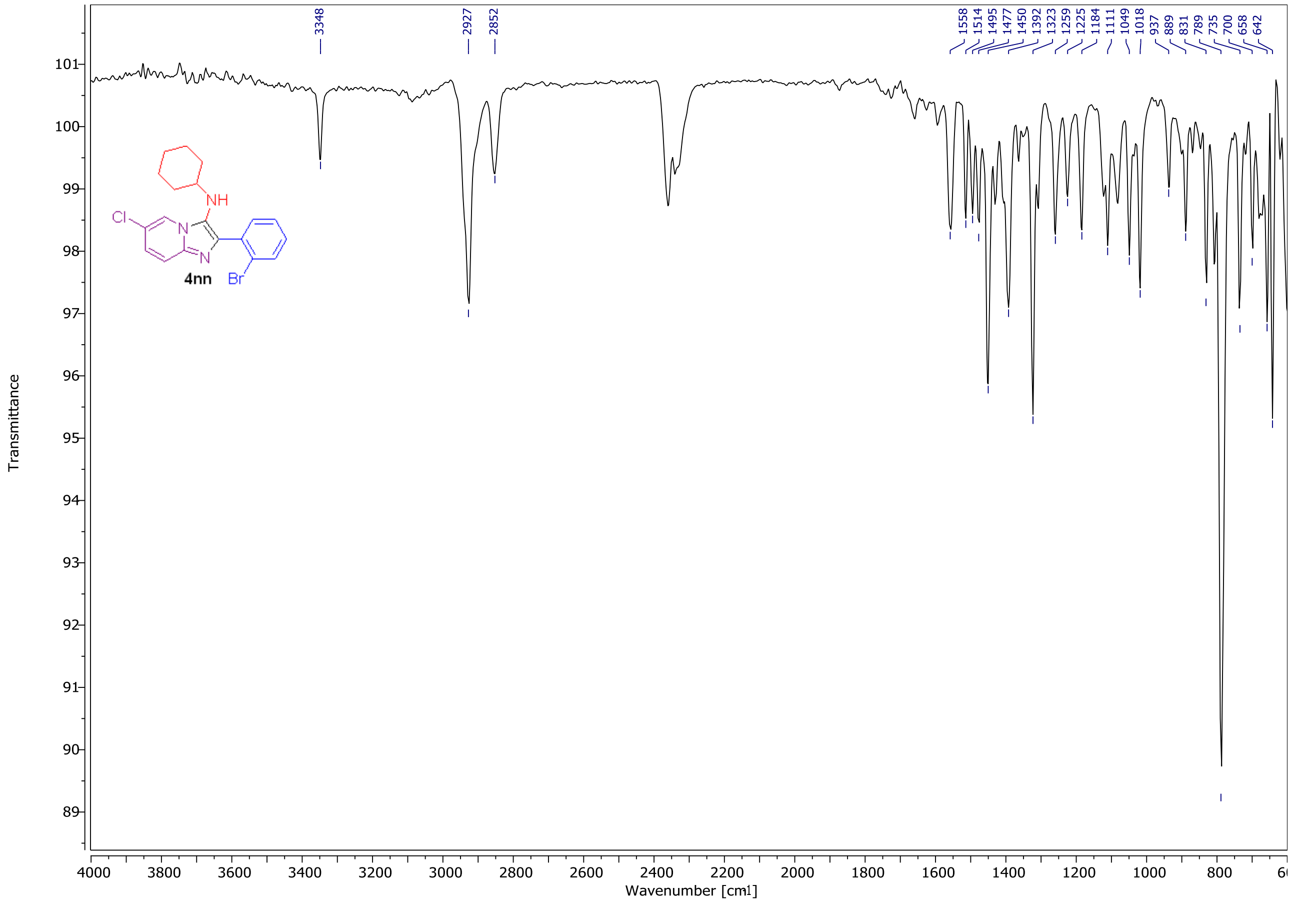

S 157. FT-IR (ATR) of compound **4nn**.

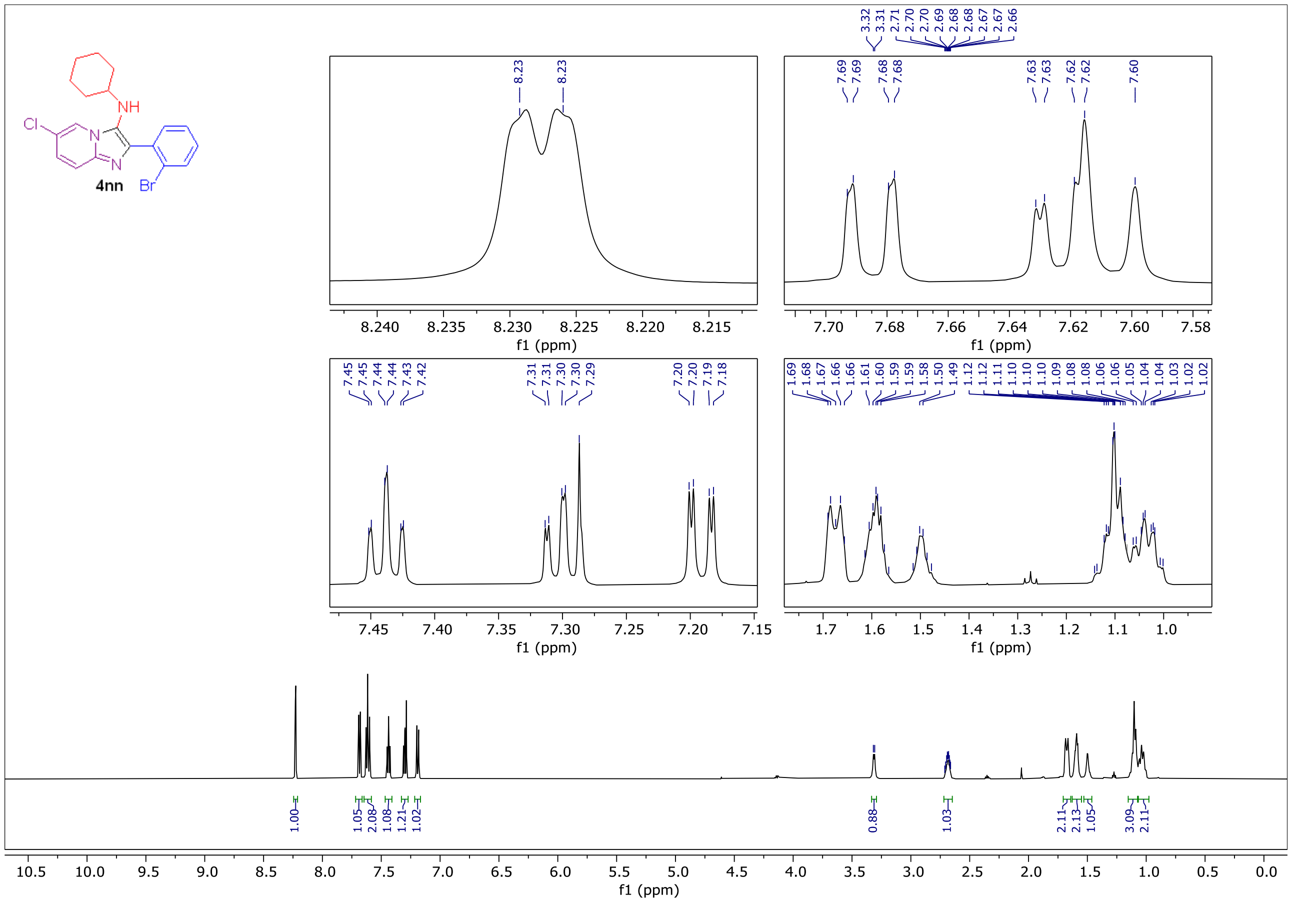

S 158. ^{1}H NMR spectrum (600 MHz, $CDCl_3$) of compound **4nn**.

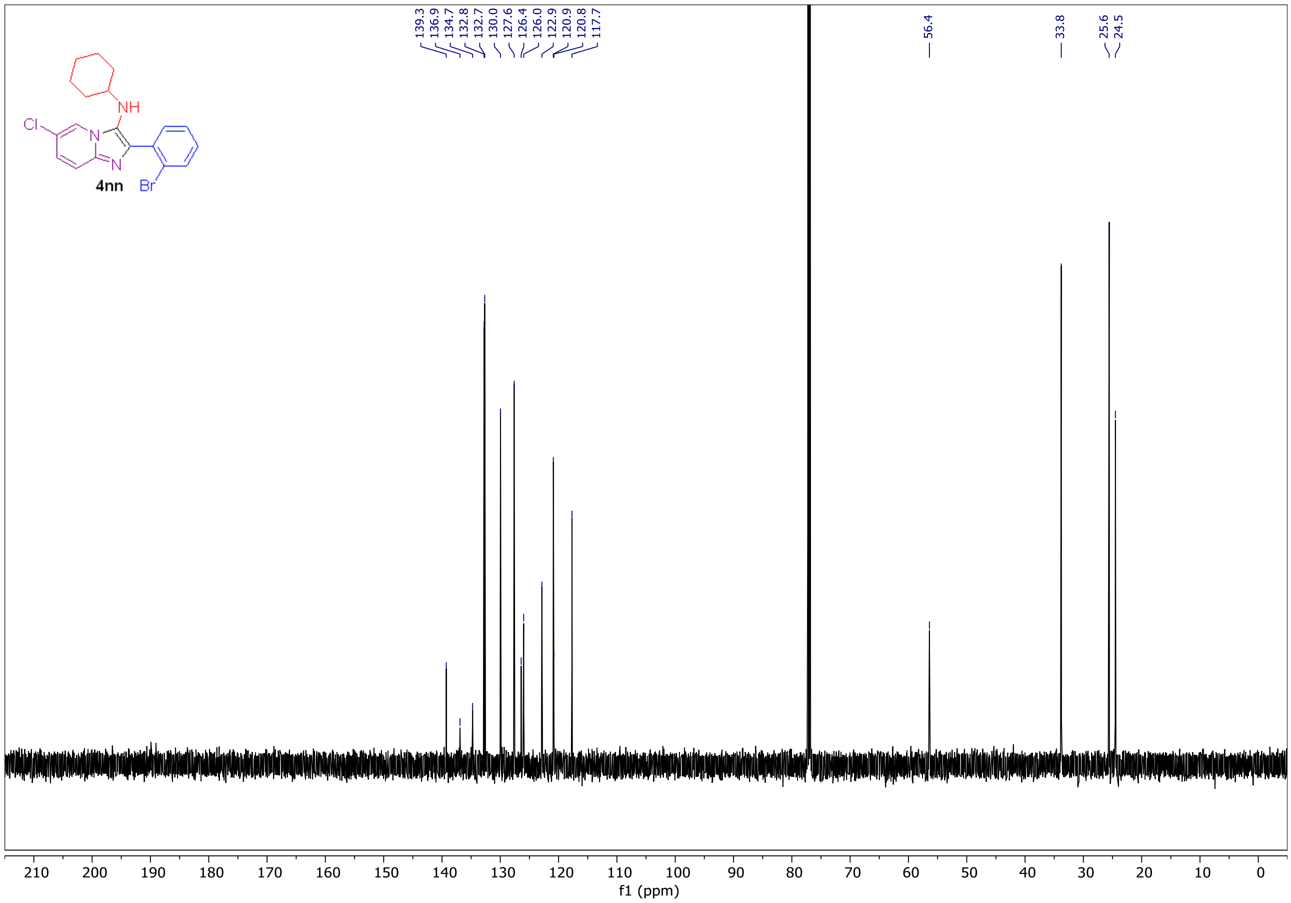

S 159. ^{13}C NMR spectrum (151 MHz, $CDCl_3$) of compound **4nn**.

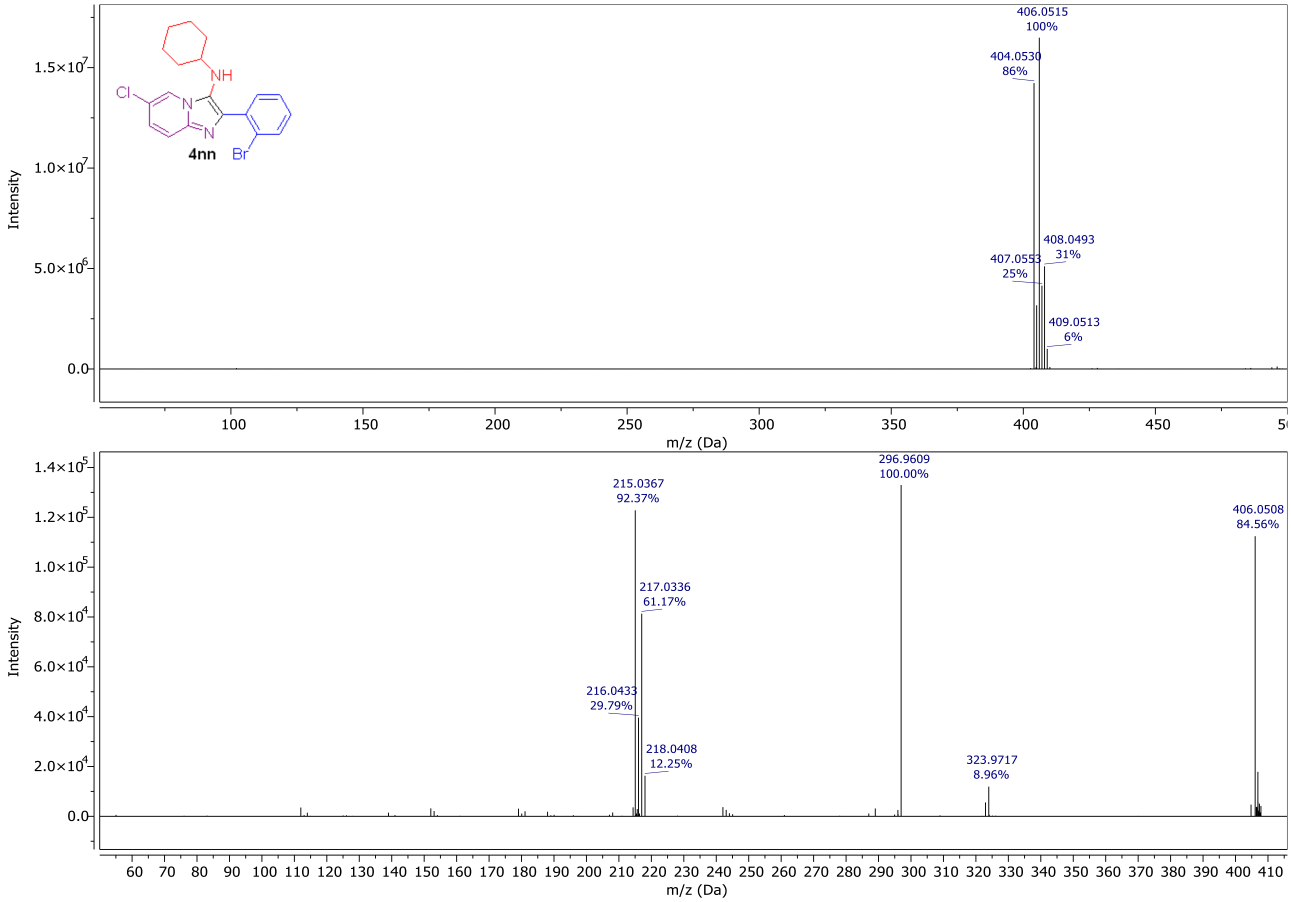

S 160. HRMS (ESI-QTOF) of compound **4nn** and HRMS/MS for $[M+H]^+$.

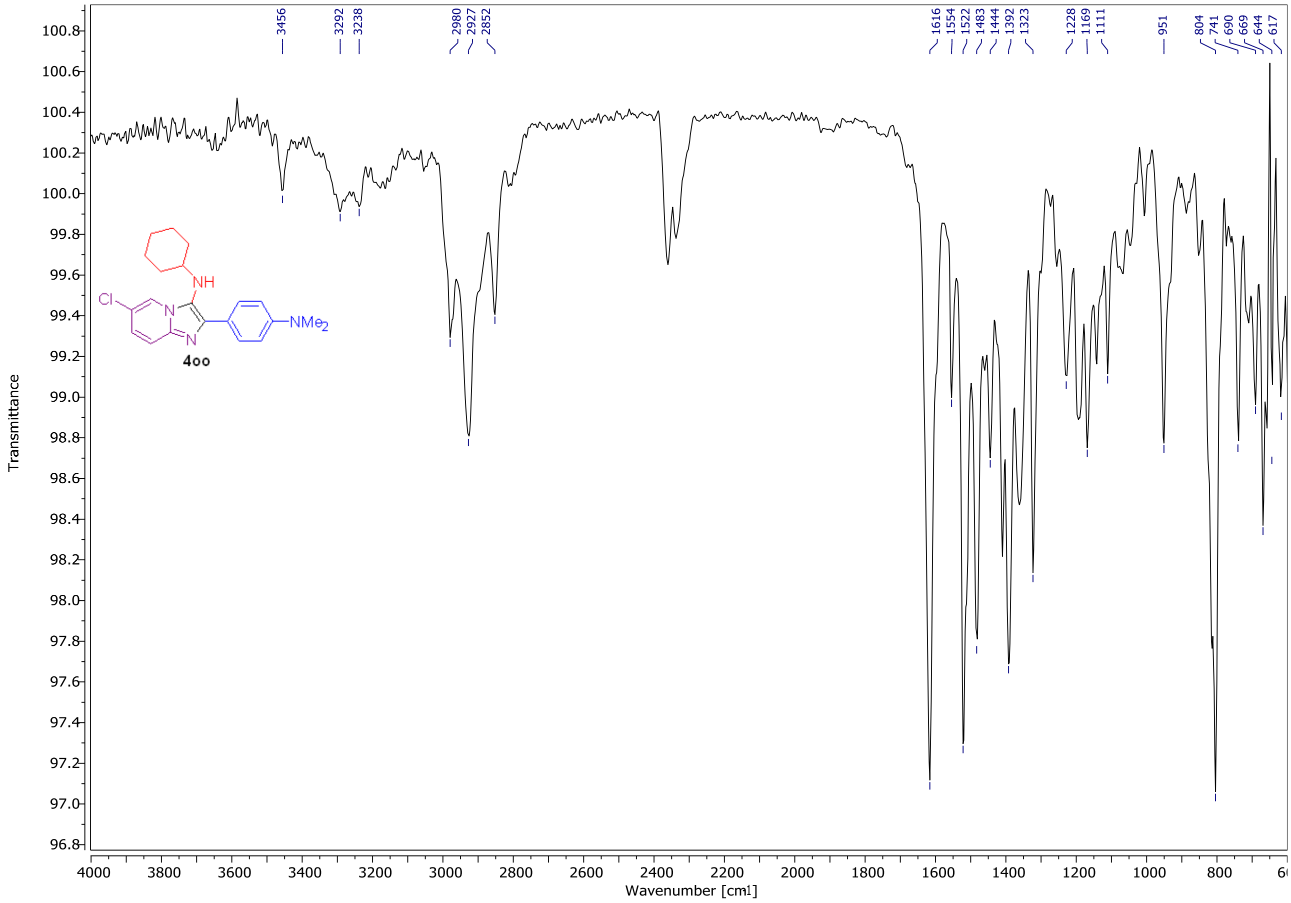

S 161. FT-IR (ATR) of compound **4oo**.

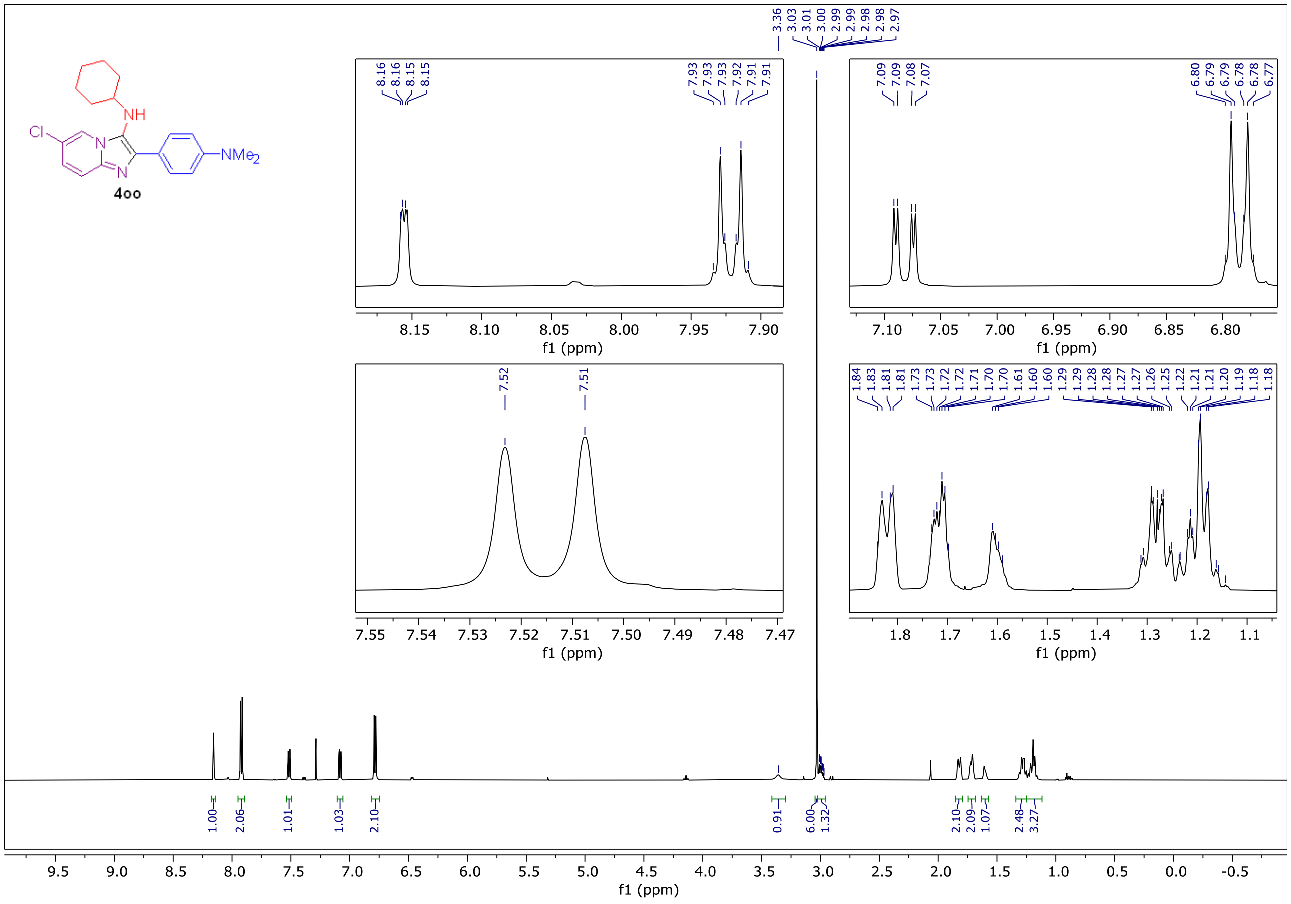

S 162. ^{1}H NMR spectrum (600 MHz, $CDCl_3$) of compound **4oo**.

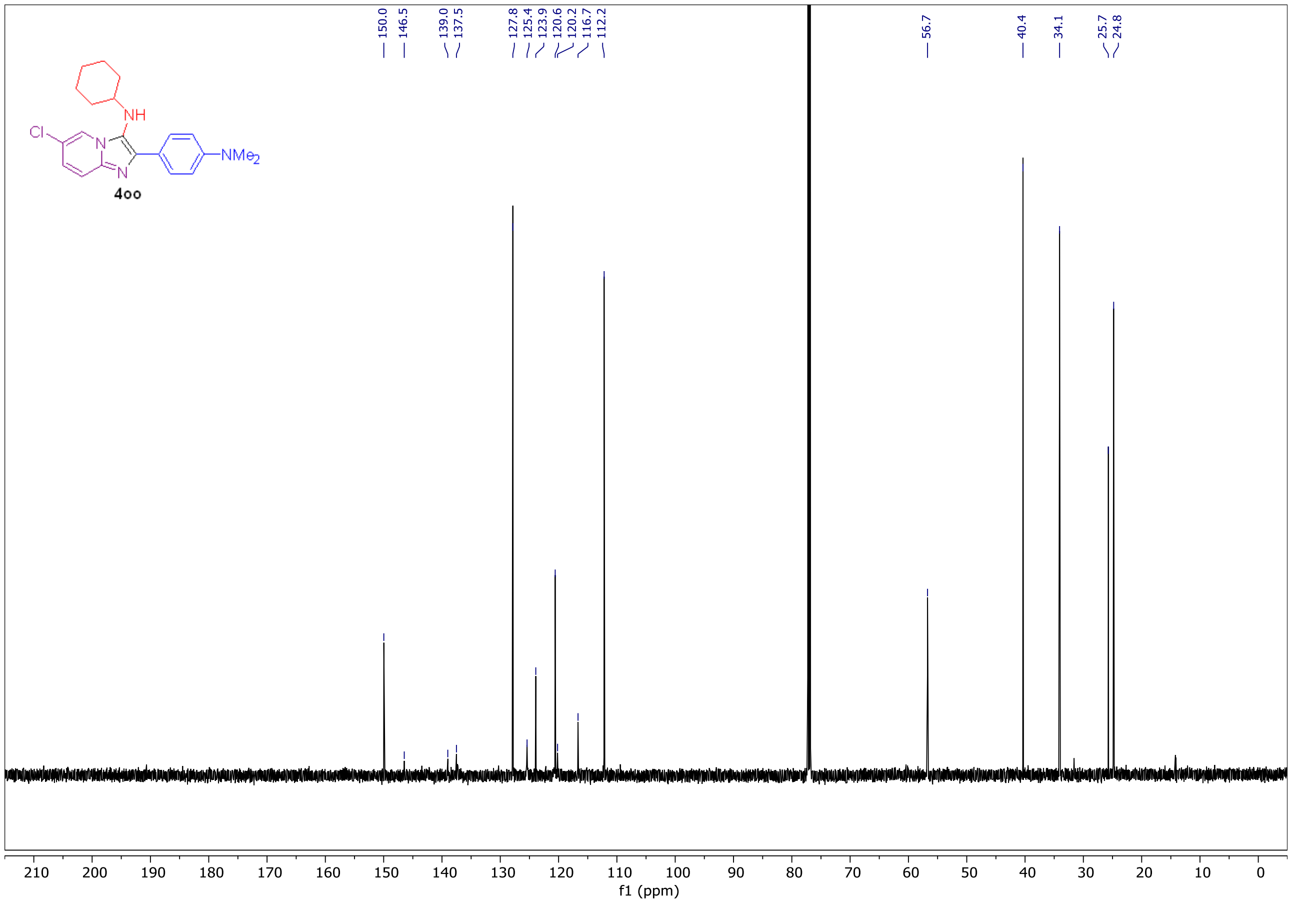

S 163. ^{13}C NMR spectrum (151 MHz, $CDCl_3$) of compound **4oo**.

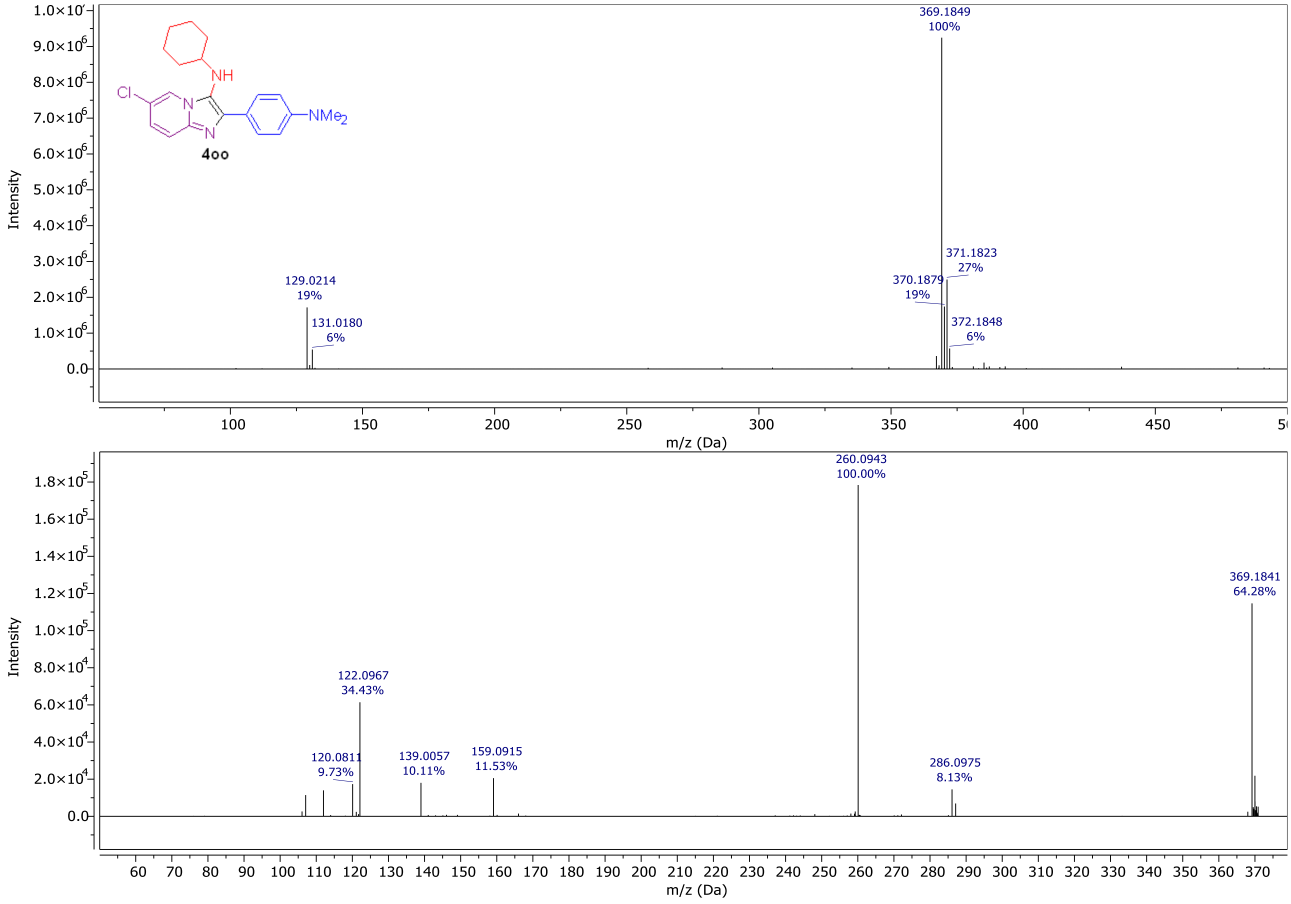

S 164. HRMS (ESI-QTOF) of compound **4oo** and HRMS/MS for $[M+H]^+$.

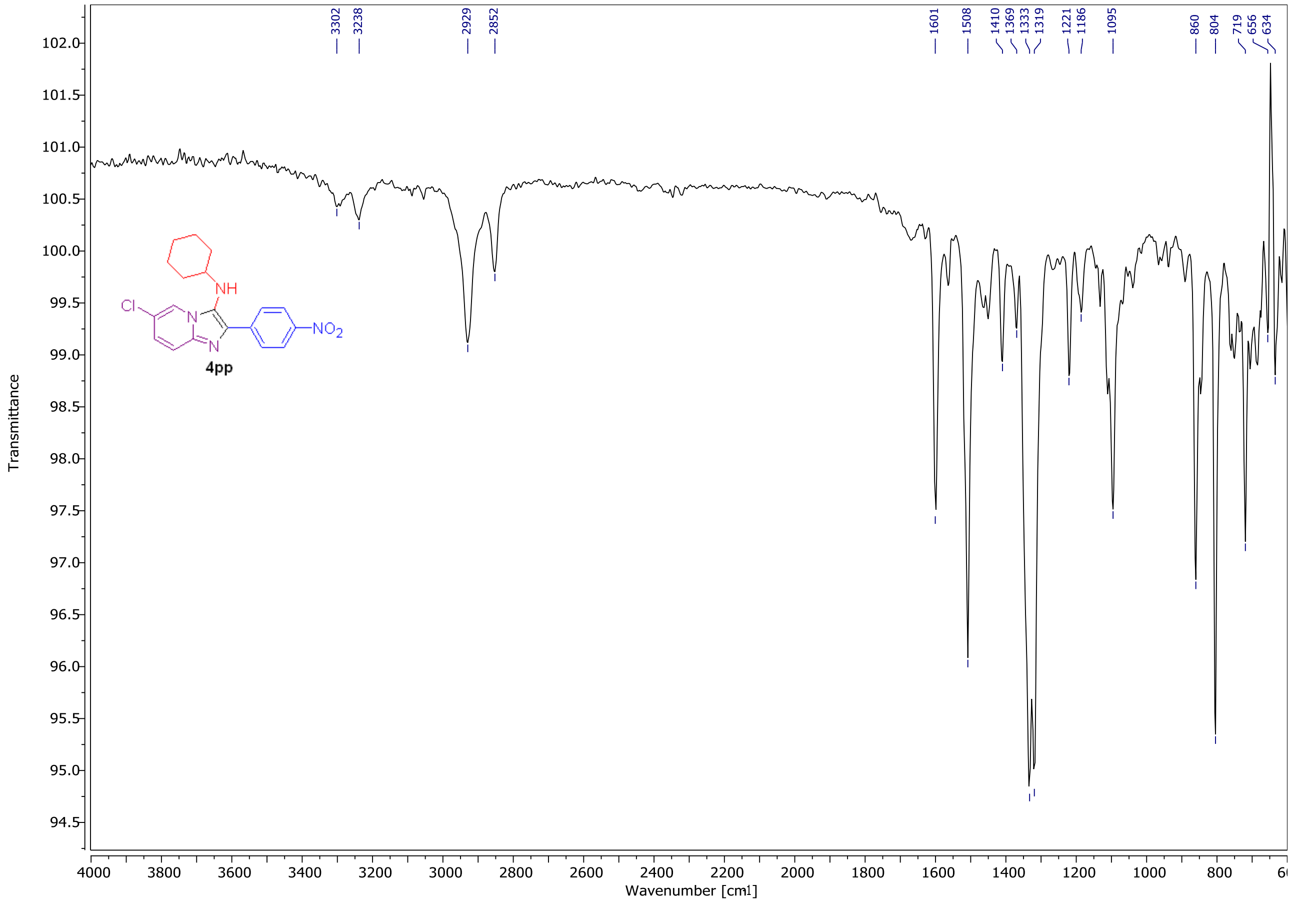

S 165. FT-IR (ATR) of compound **4pp**.

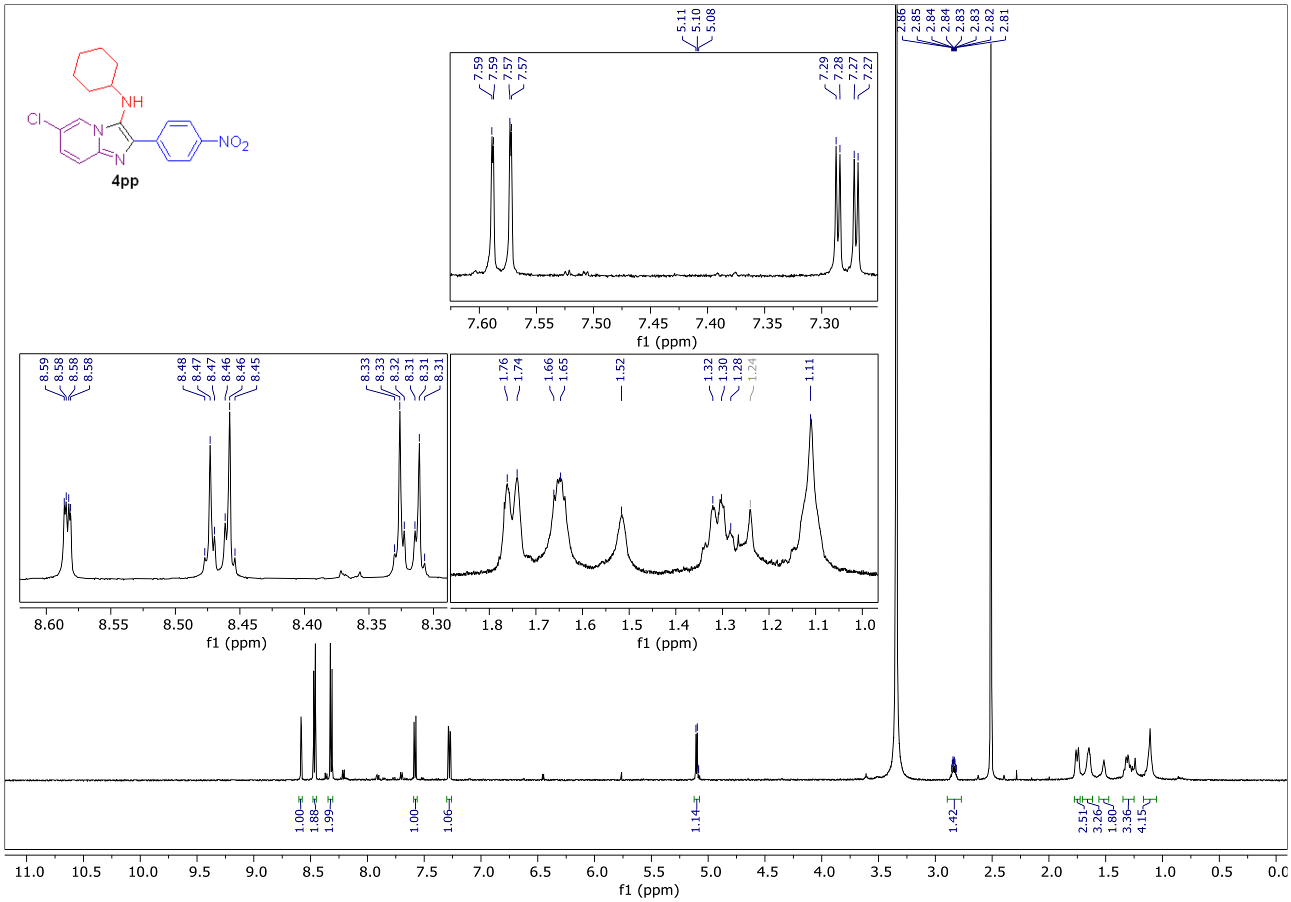

S 166. ^{1}H NMR spectrum (600 MHz, DMSO-d_6) of compound **4pp**.

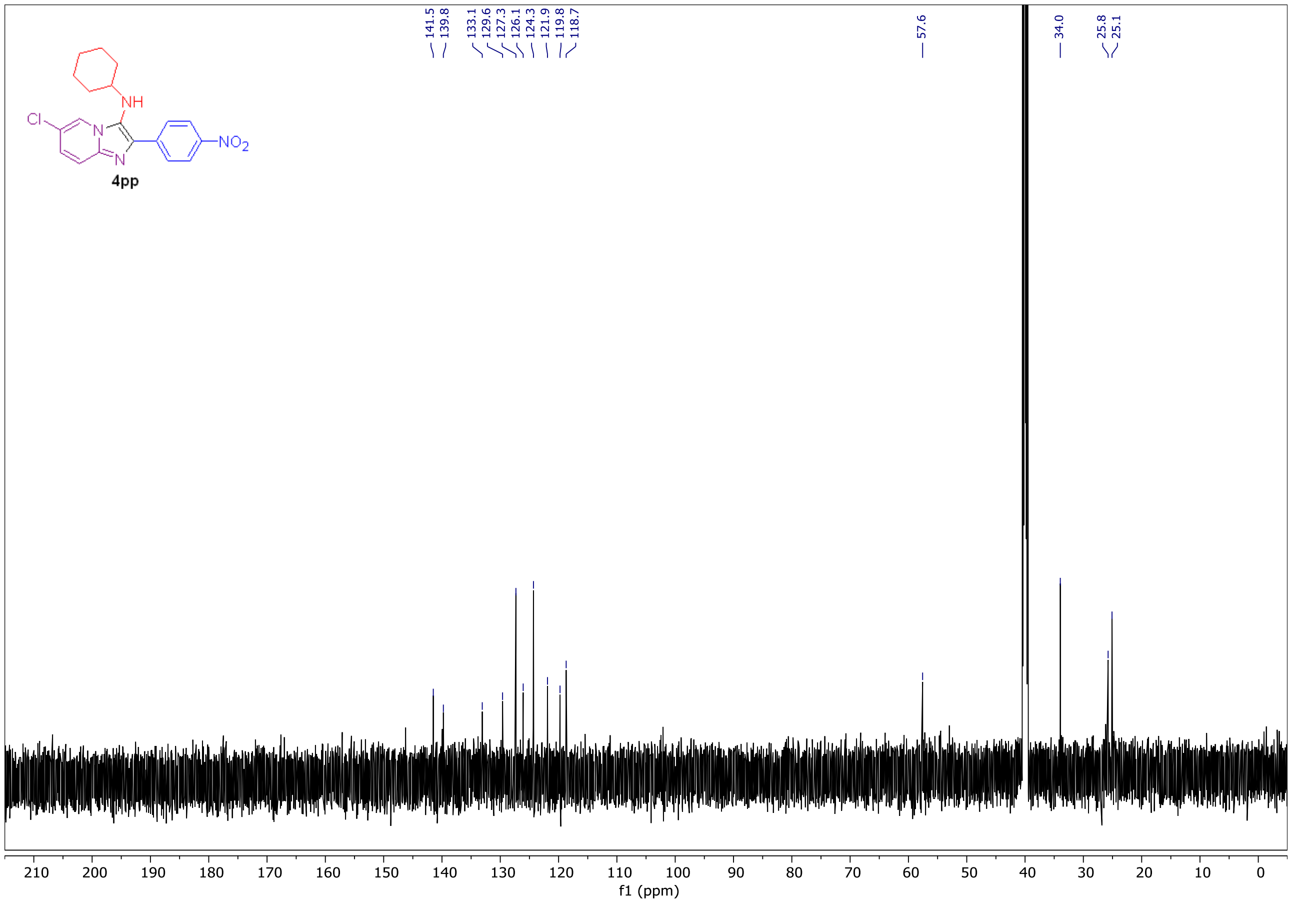

S 167. ^{13}C NMR spectrum (151 MHz, $CDCl_3$) of compound **4pp**.

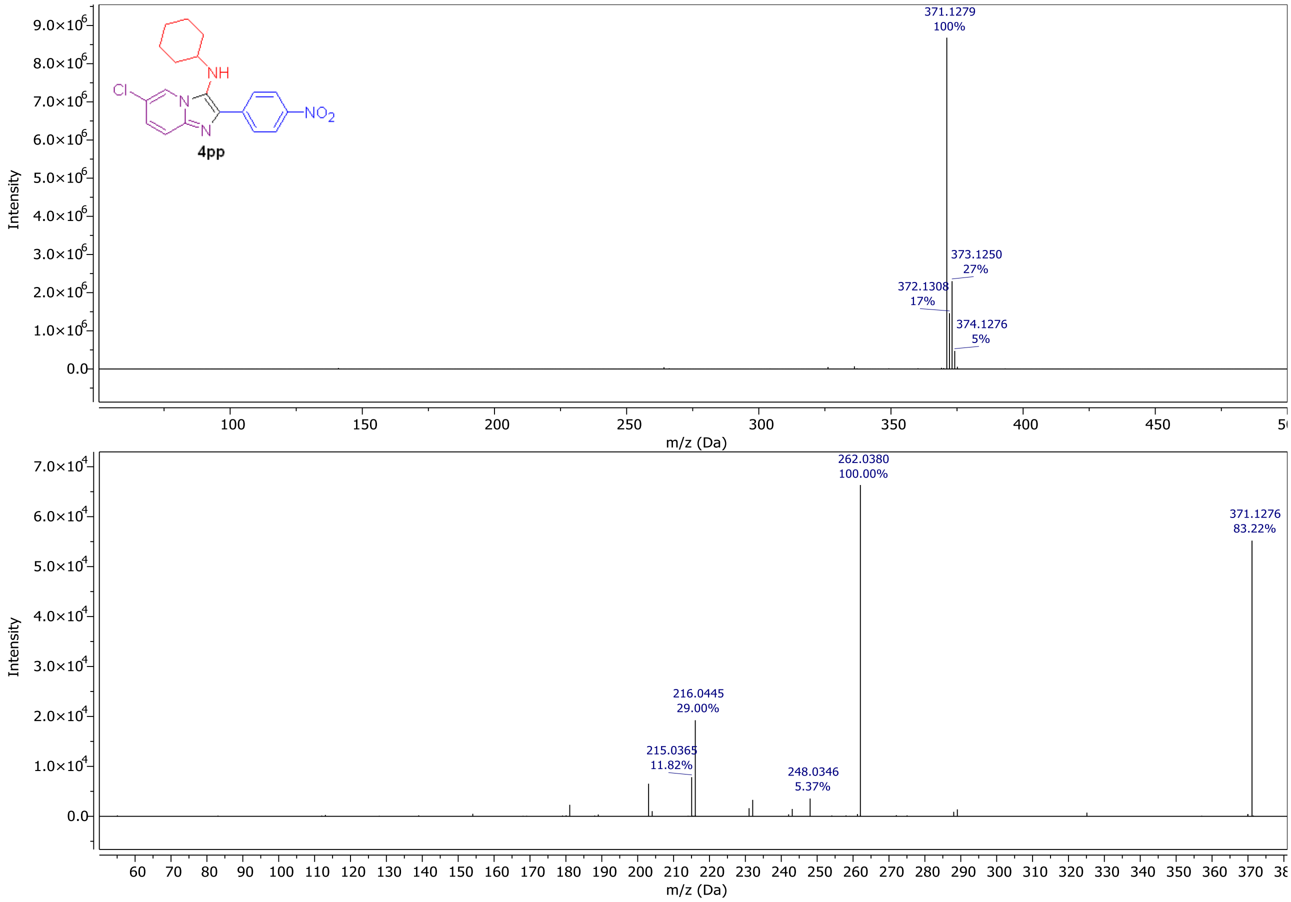

S 168. HRMS (ESI-QTOF) of compound **4pp** and HRMS/MS for $[M+H]^+$.

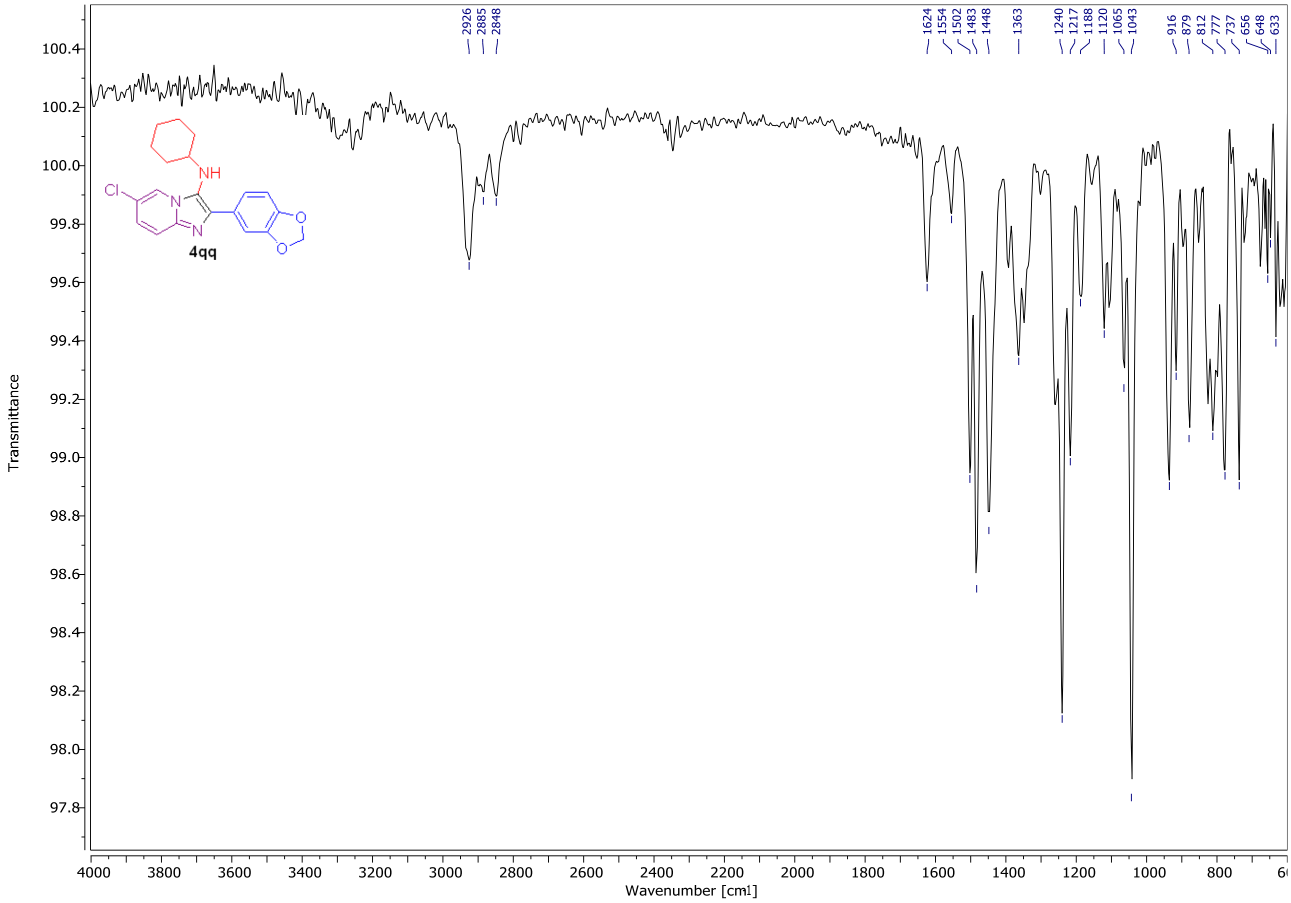

S 169. FT-IR (ATR) of compound **4qq**.

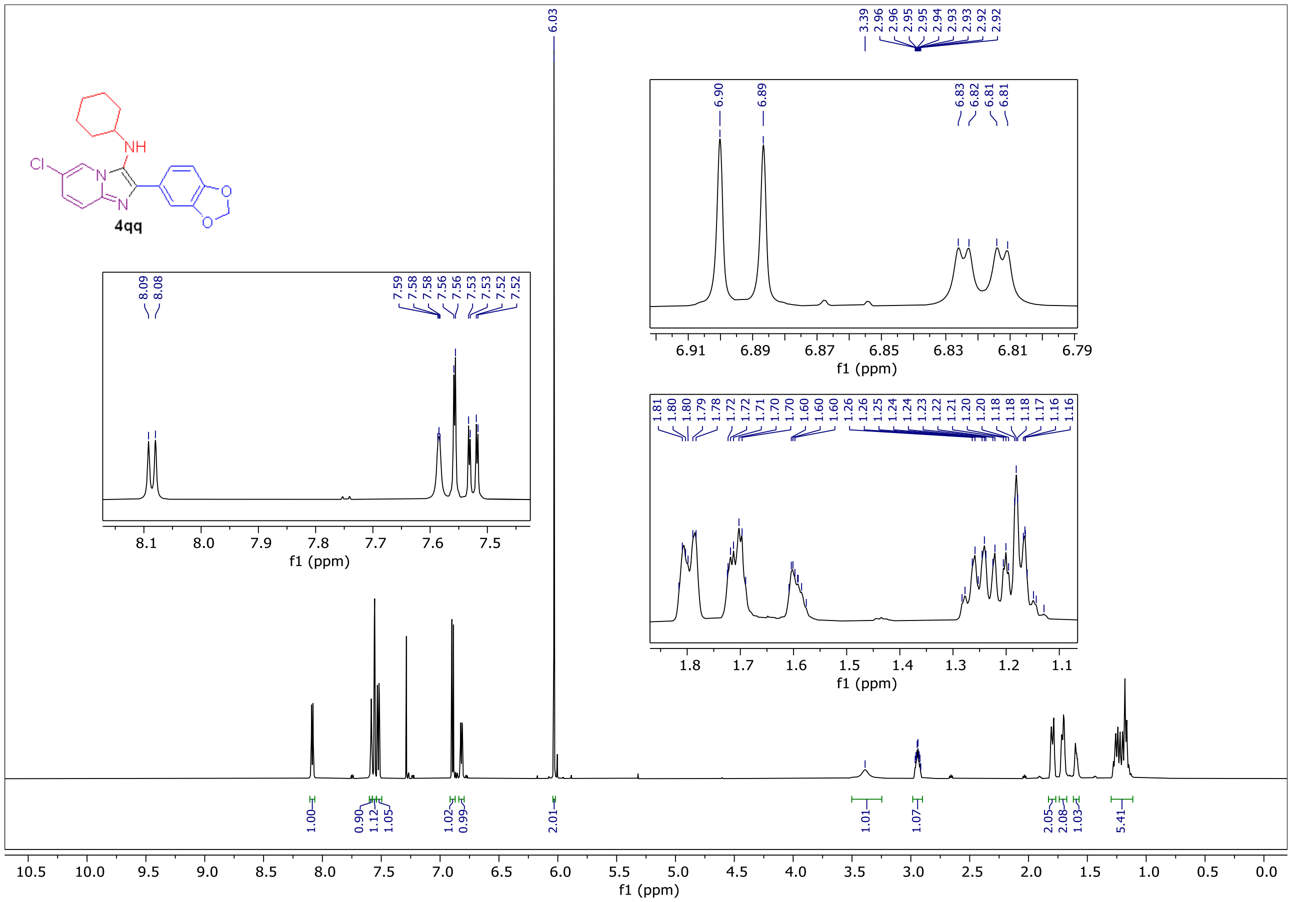

S 170. ^{1}H NMR spectrum (600 MHz, $CDCl_3$) of compound **4qq**.

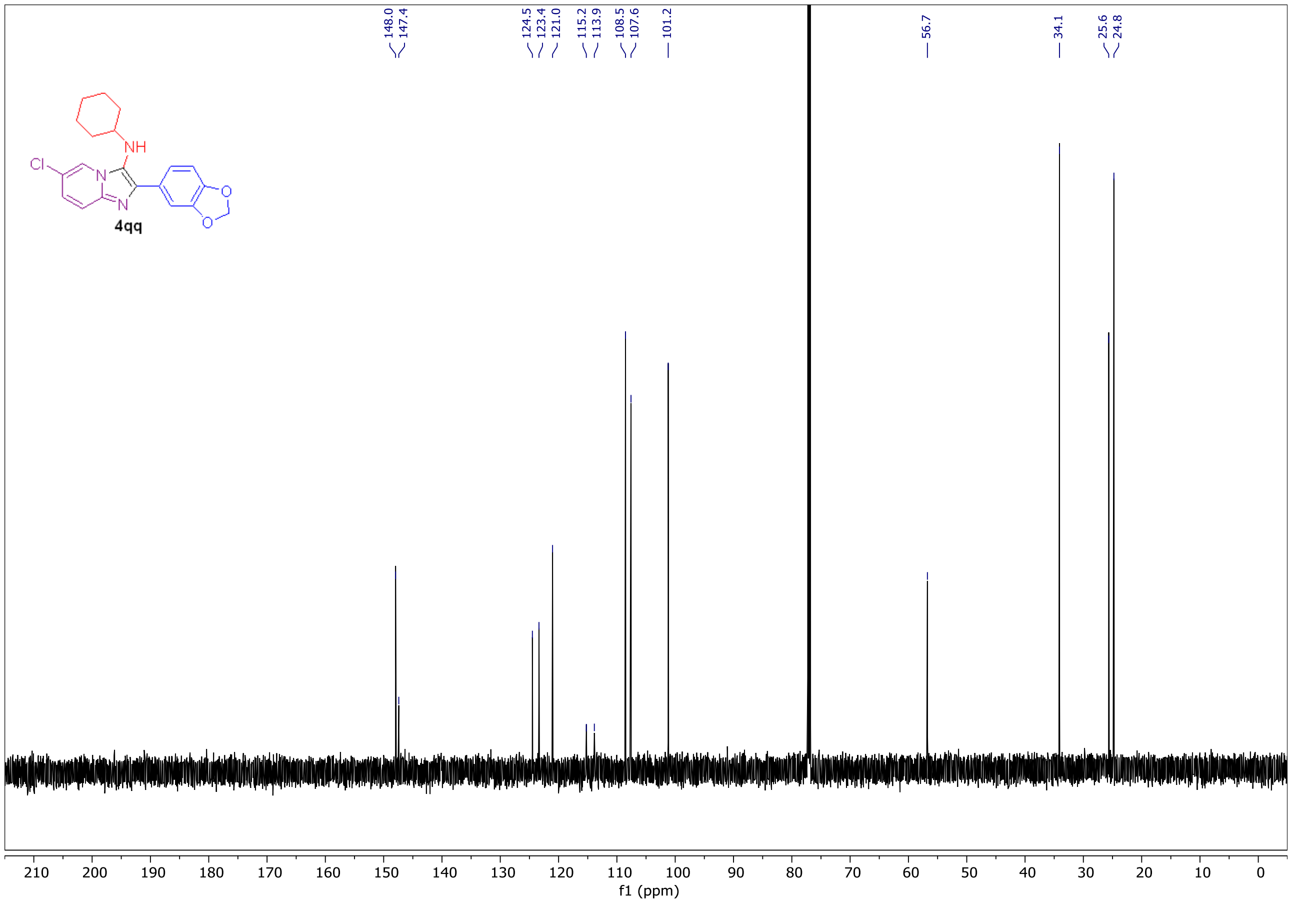

S 171. ^{13}C NMR spectrum (151 MHz, $CDCl_3$) of compound **4qq**.

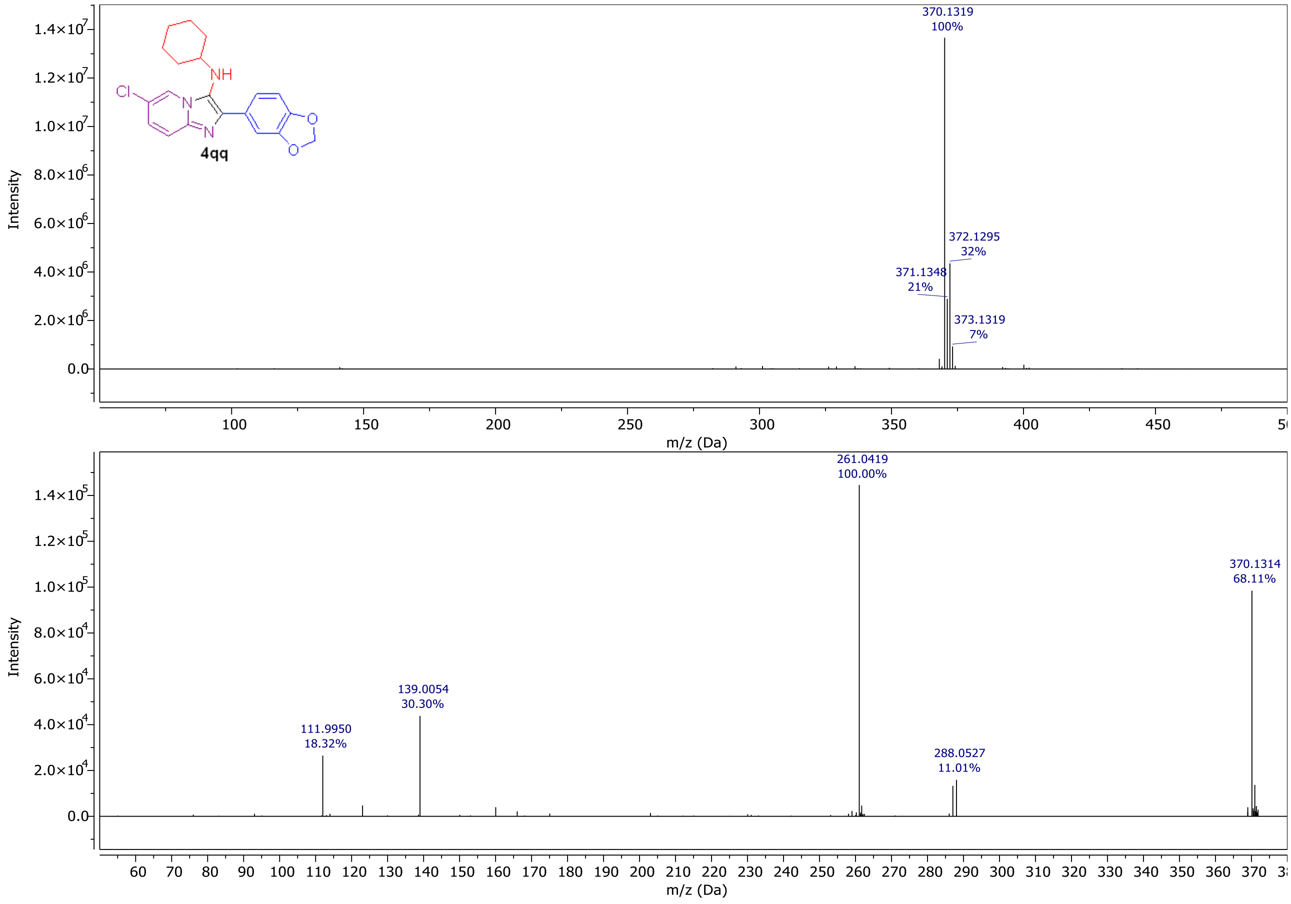

S 172. HRMS (ESI-QTOF) of compound **4qq** and HRMS/MS for [M+H]$^+$.

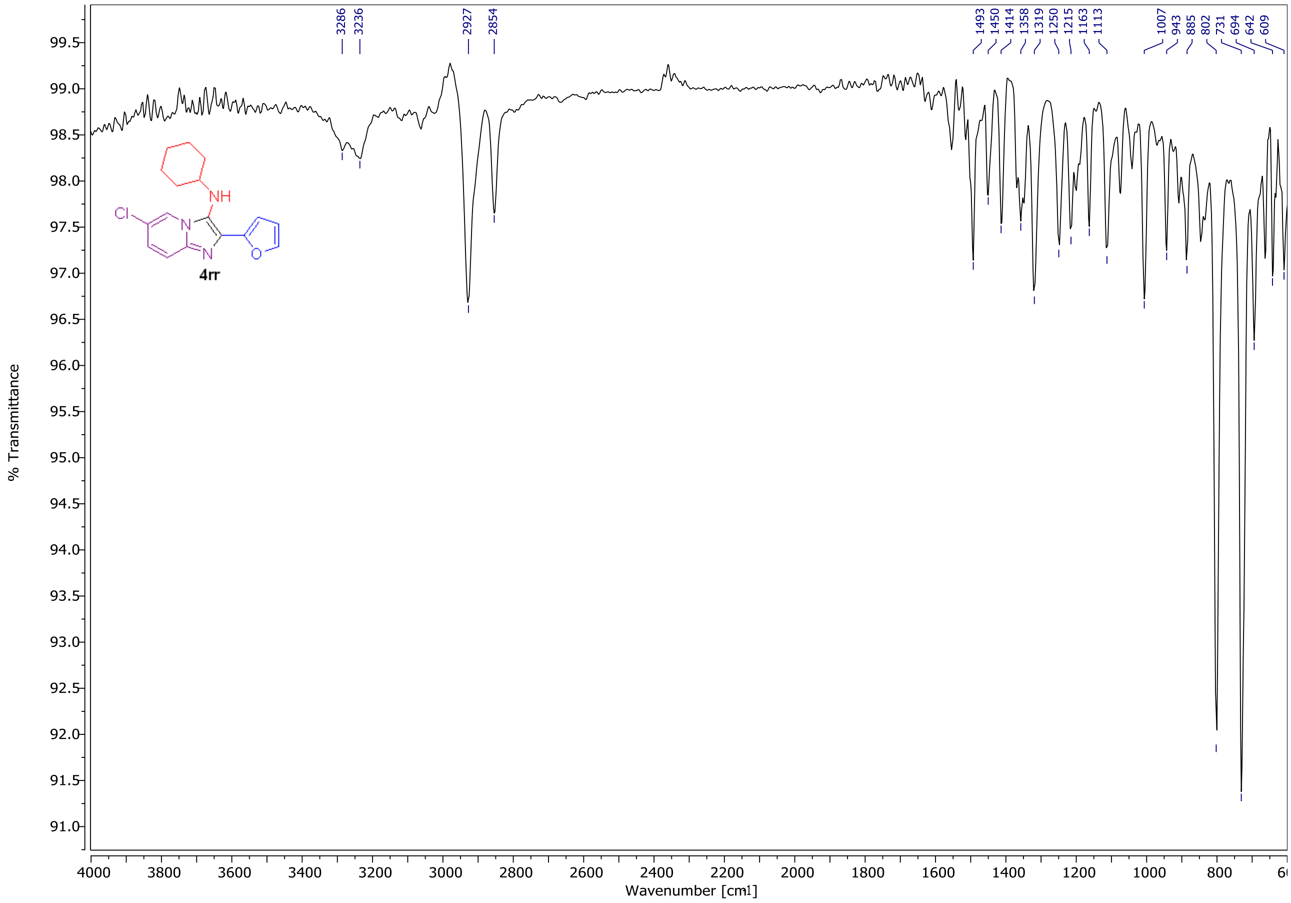

S 173. FT-IR (ATR) of compound **4rr**.

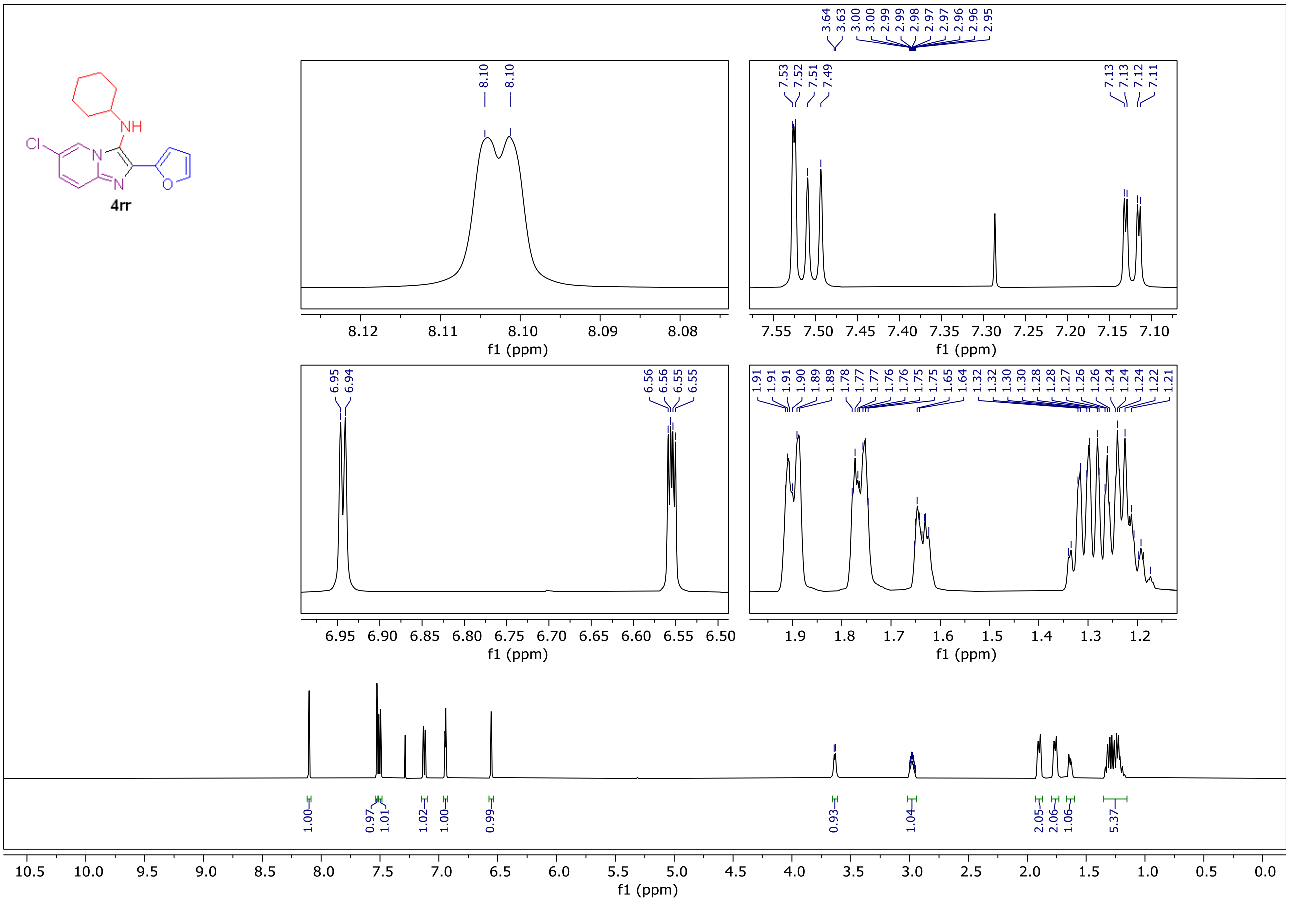

S 174. ^{1}H NMR spectrum (600 MHz, $CDCl_3$) of compound **4rr**.

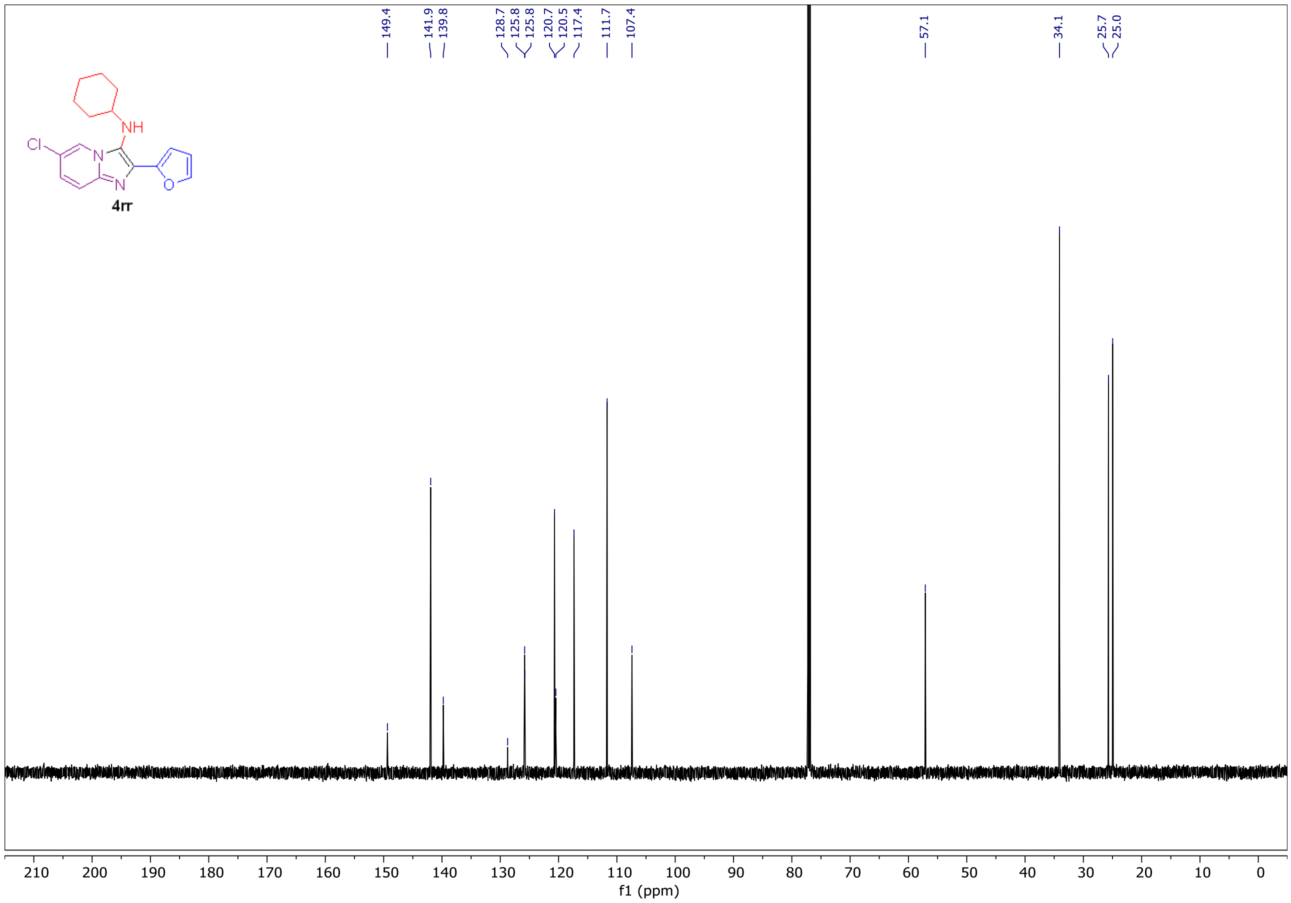

S 175. ^{13}C NMR spectrum (151 MHz, $CDCl_3$) of compound **4rr**.

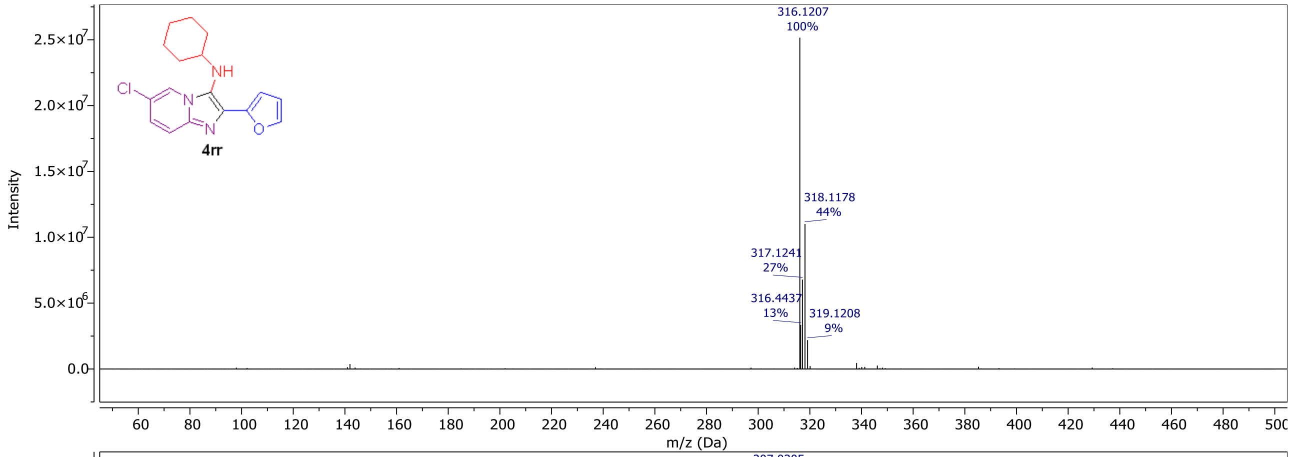

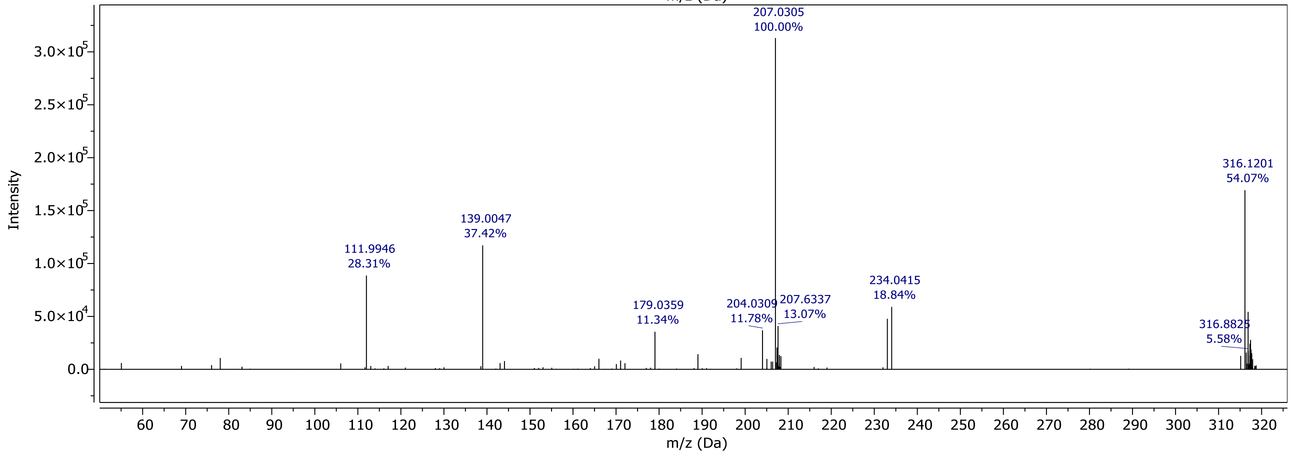

S 176. HRMS (ESI-QTOF) of compound **4rr** and HRMS/MS for $[M+H]^+$.

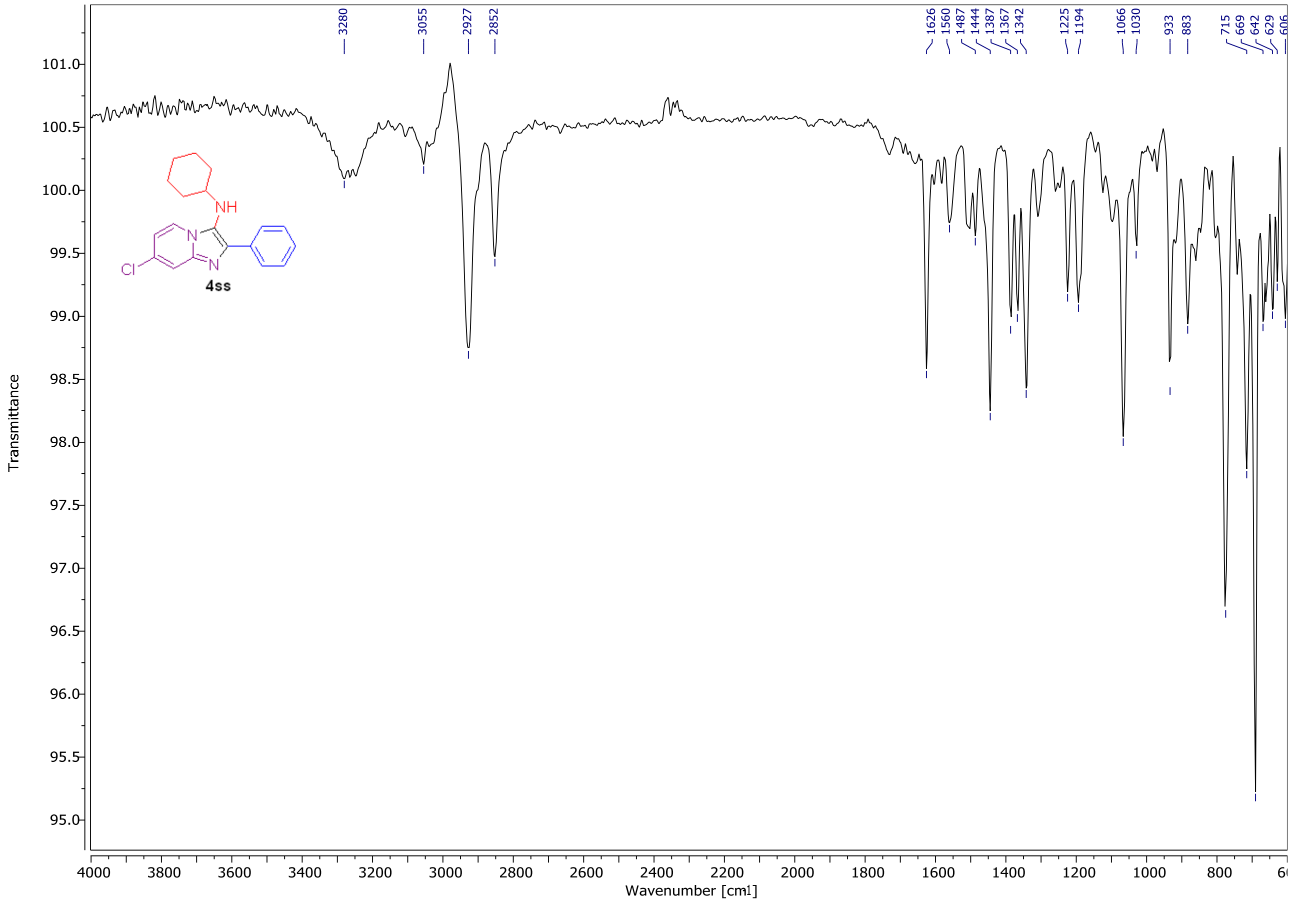

S 177. FT-IR (ATR) of compound **4ss**.

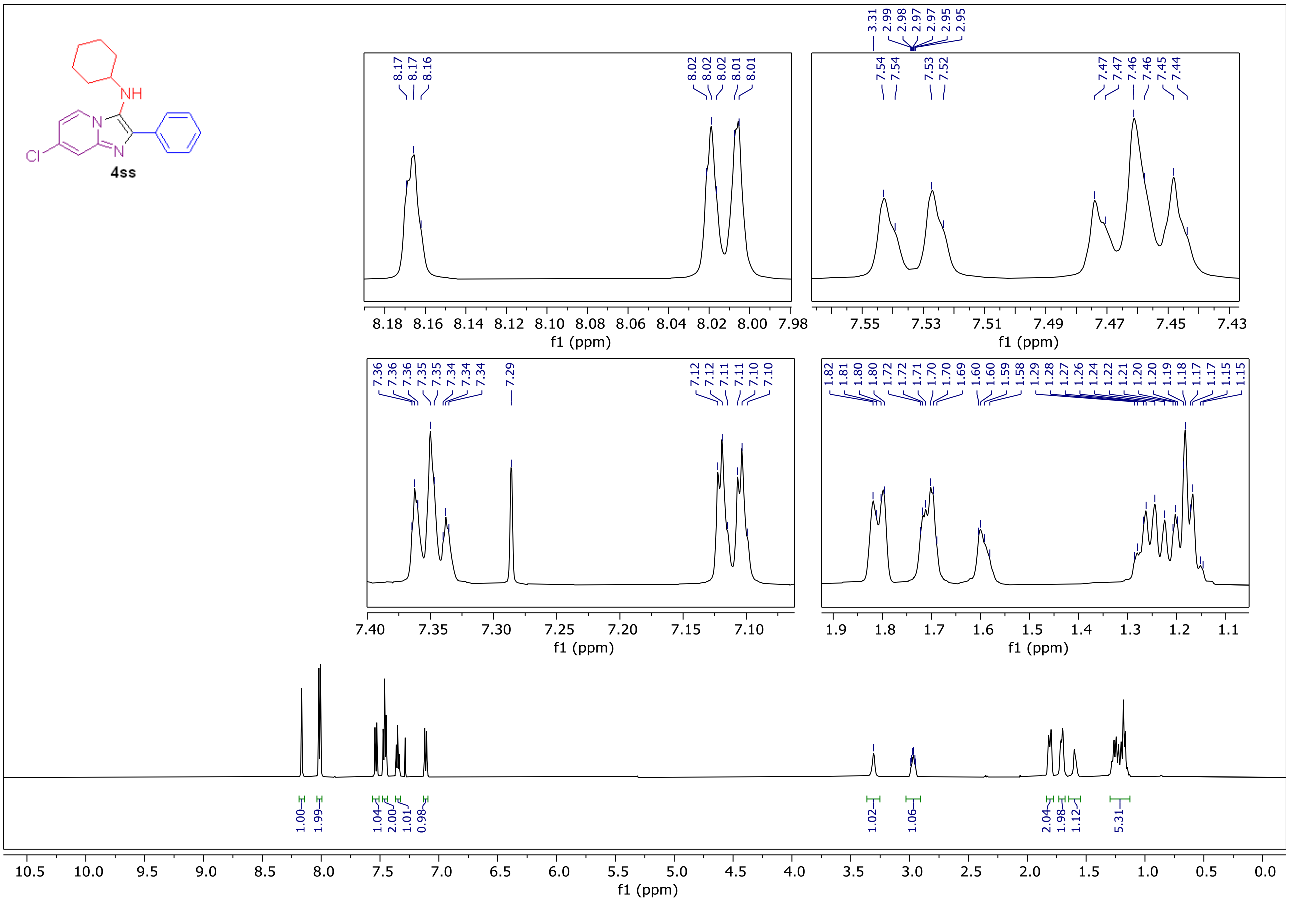

S 178. ^{1}H NMR spectrum (600 MHz, $CDCl_3$) of compound **4ss**.

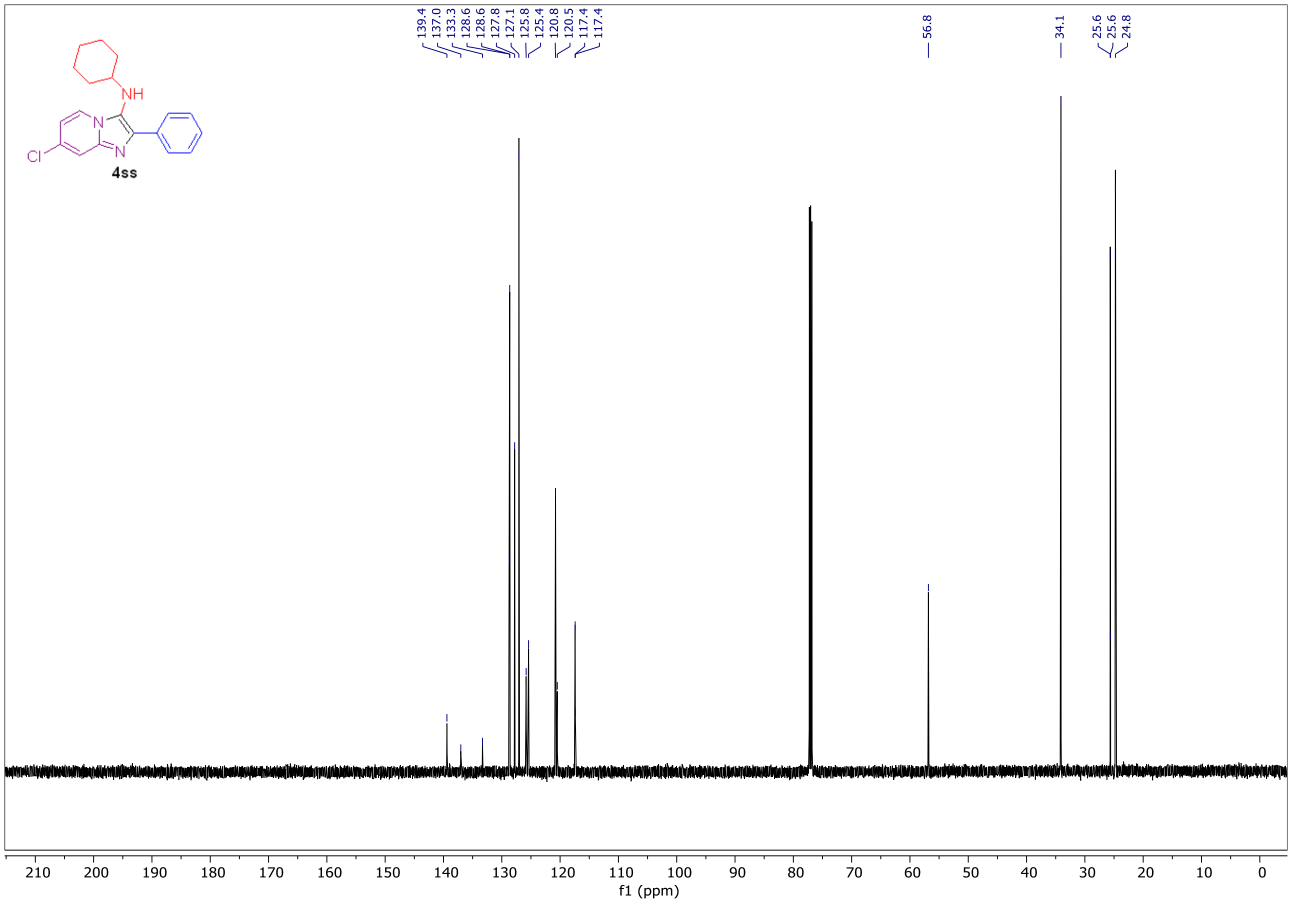

S 179. ^{13}C NMR spectrum (151 MHz, $CDCl_3$) of compound **4ss**.

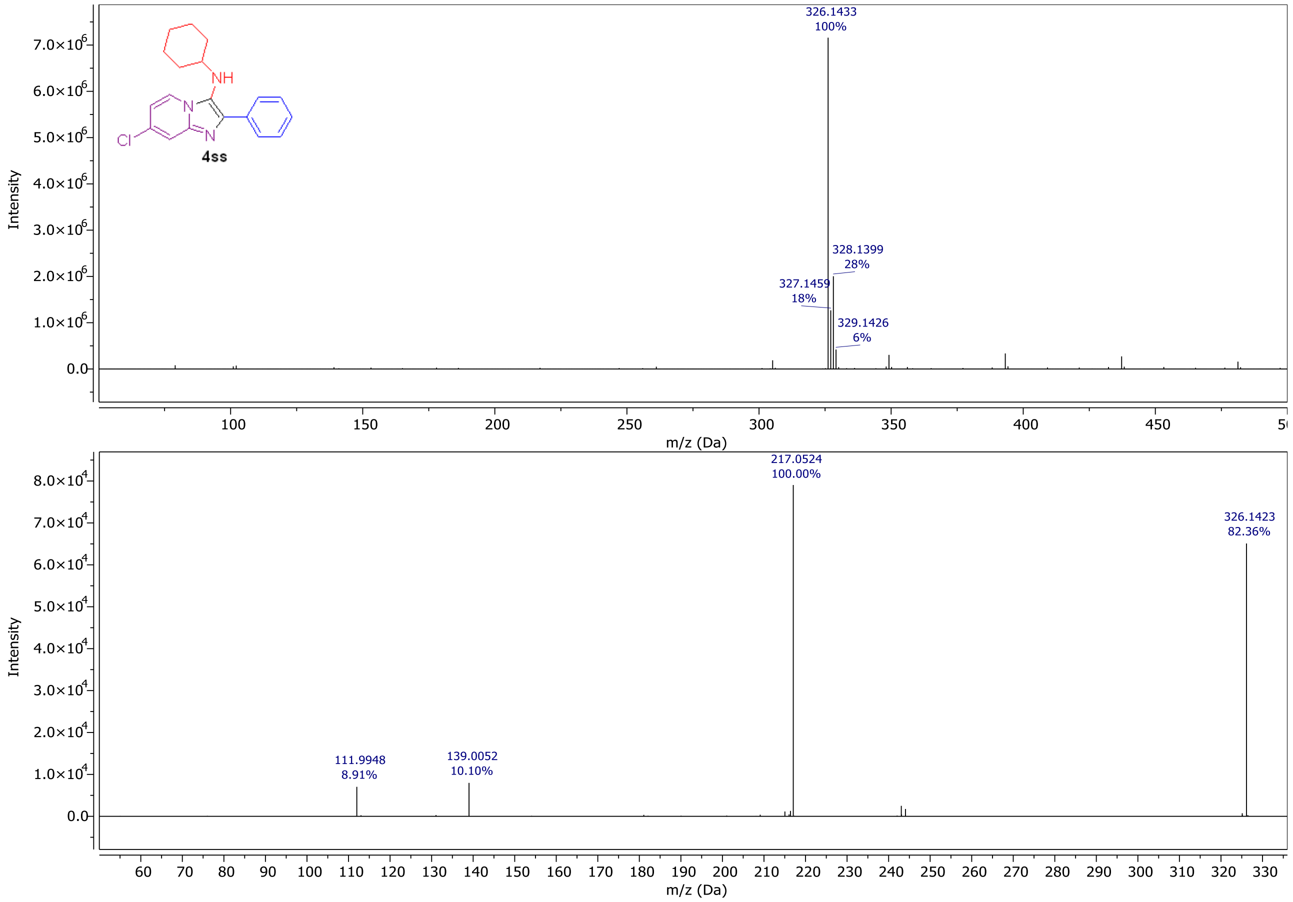

S 180. HRMS (ESI-QTOF) of compound **4ss** and HRMS/MS for $[M+H]^+$.

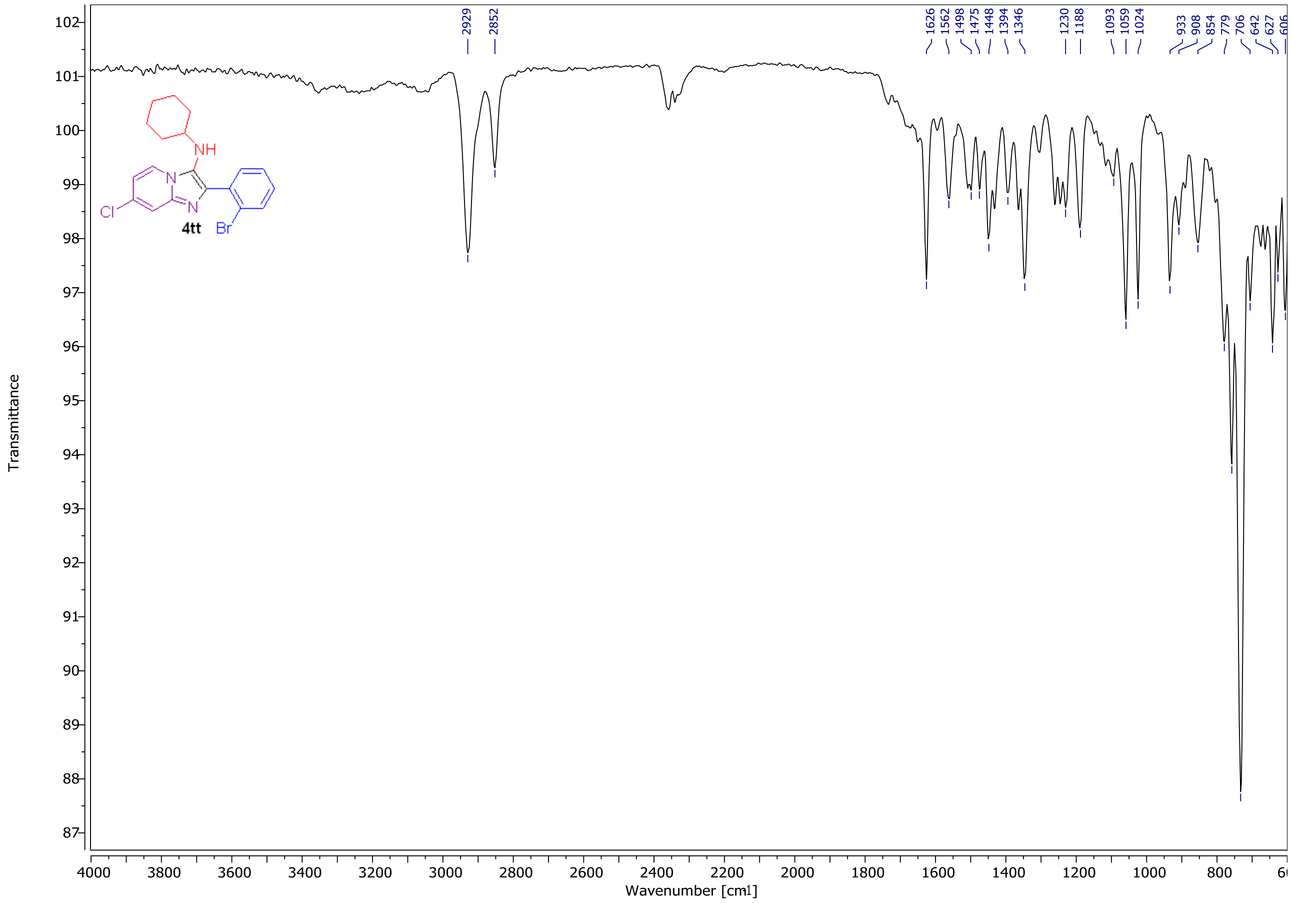

S 181. FT-IR (ATR) of compound **4tt**.

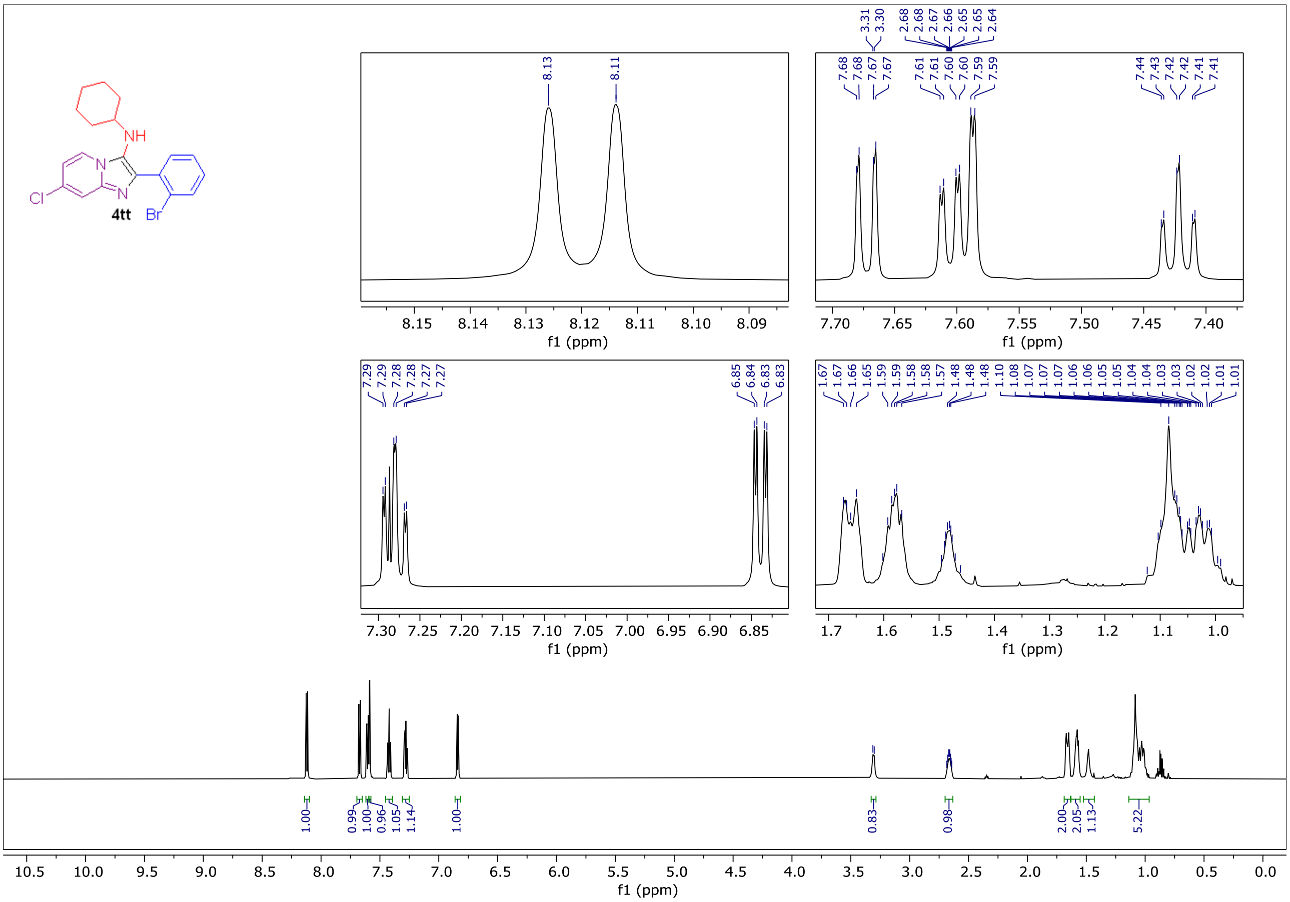

S 182. ^{1}H NMR spectrum (600 MHz, $CDCl_3$) of compound **4tt**.

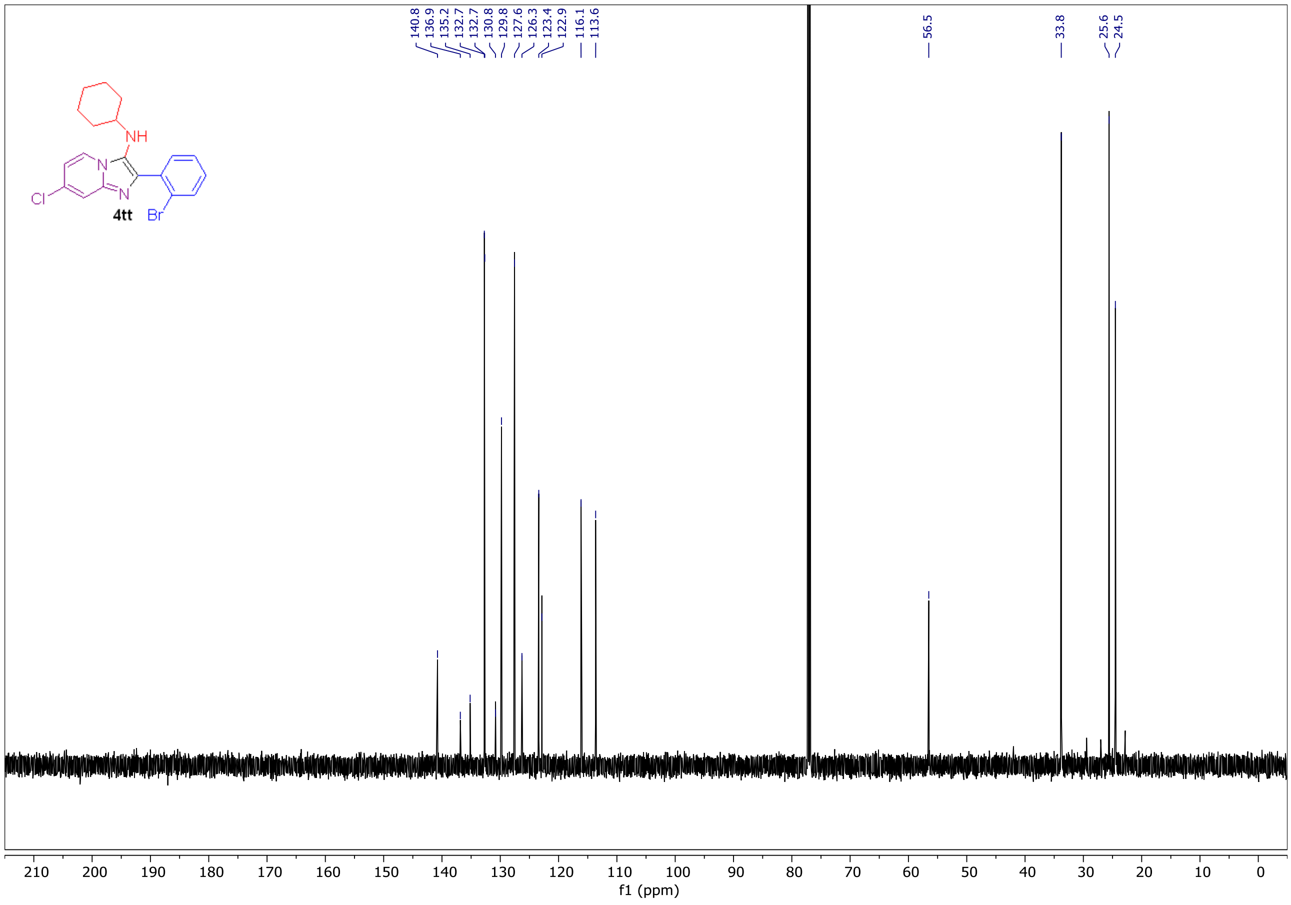

S 183. ^{13}C NMR spectrum (151 MHz, $CDCl_3$) of compound **4tt**.

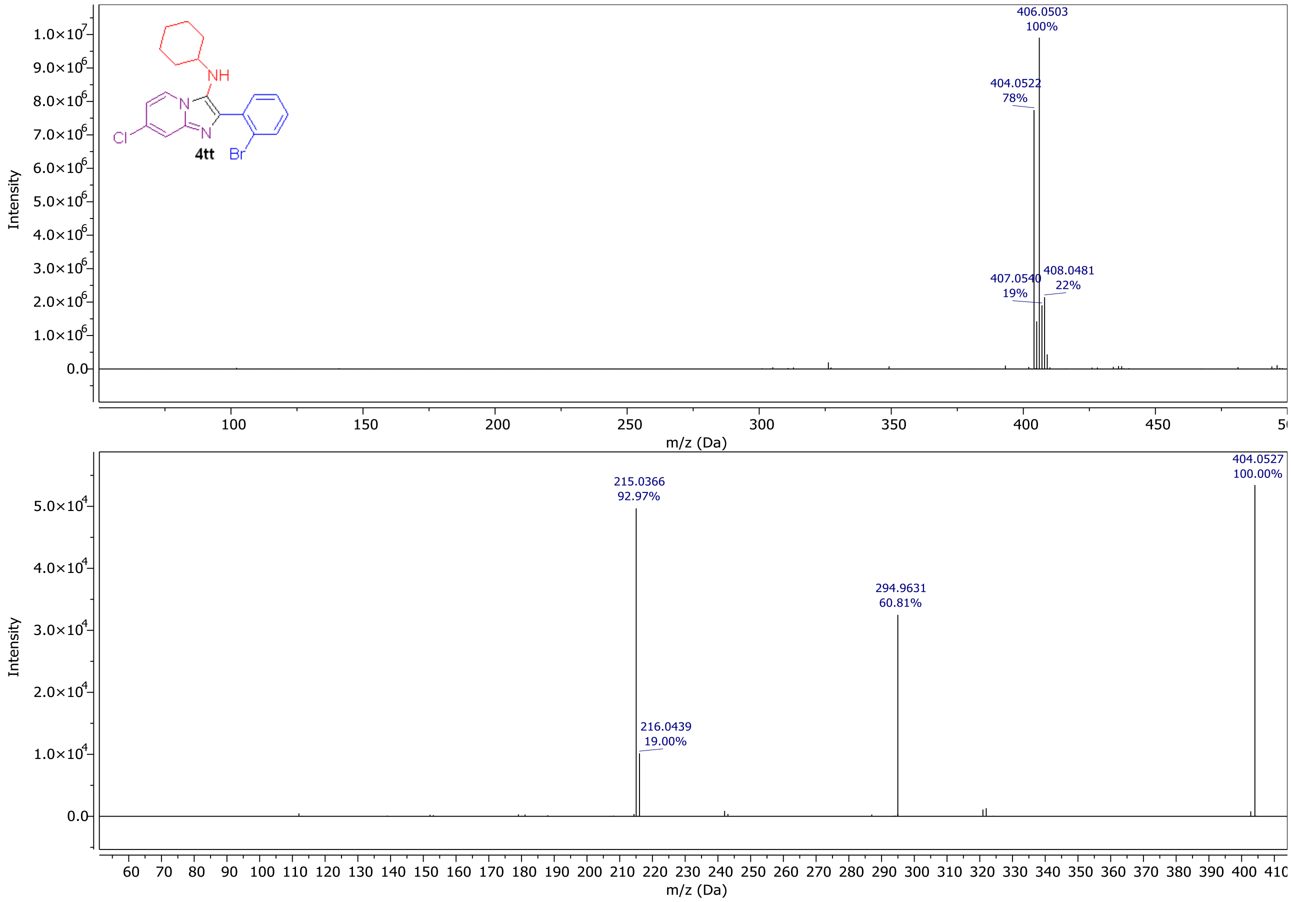

S 184. HRMS (ESI-QTOF) of compound **4tt** and HRMS/MS for $[M+H]^+$.

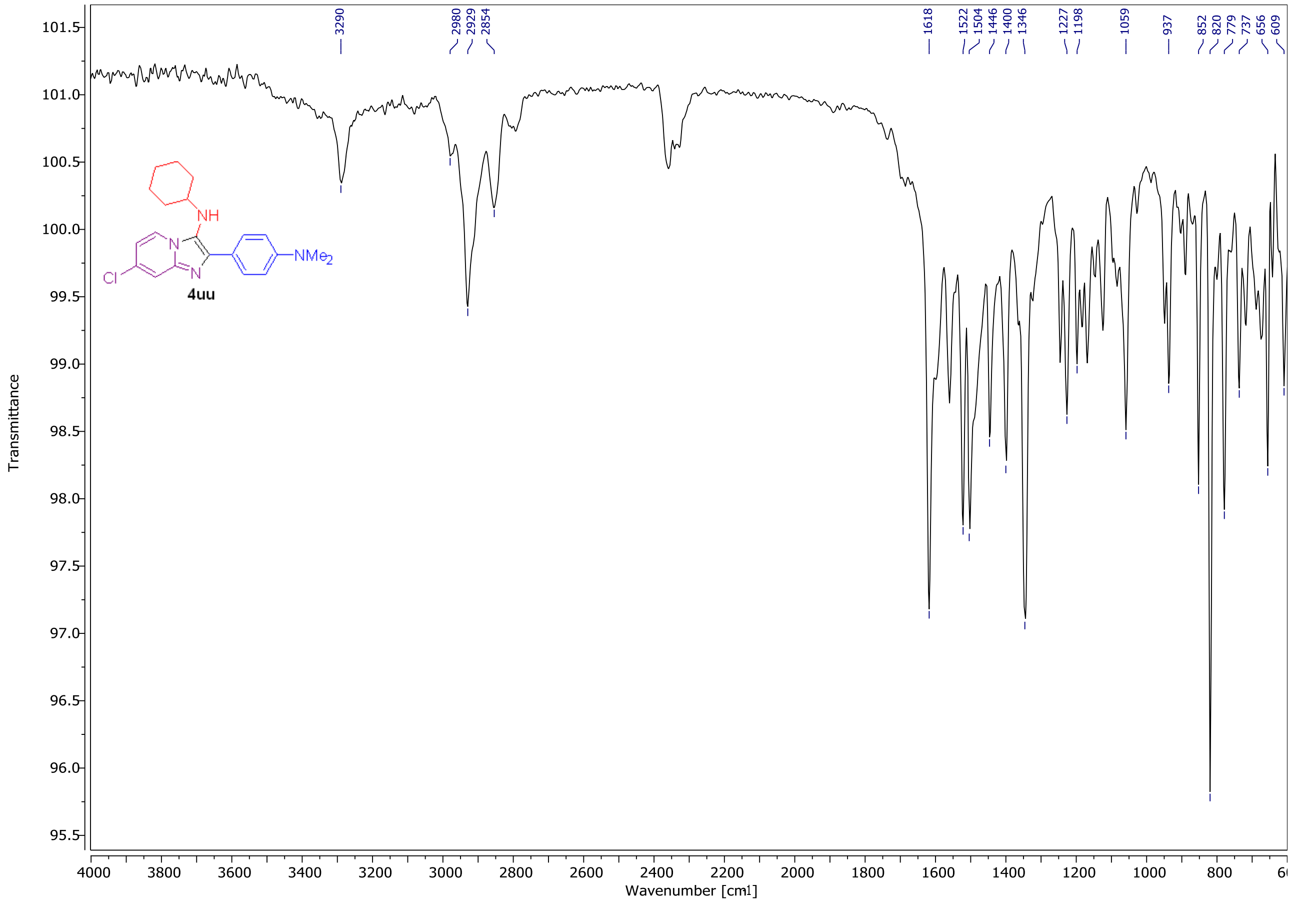

S 185. FT-IR (ATR) of compound **4uu**.

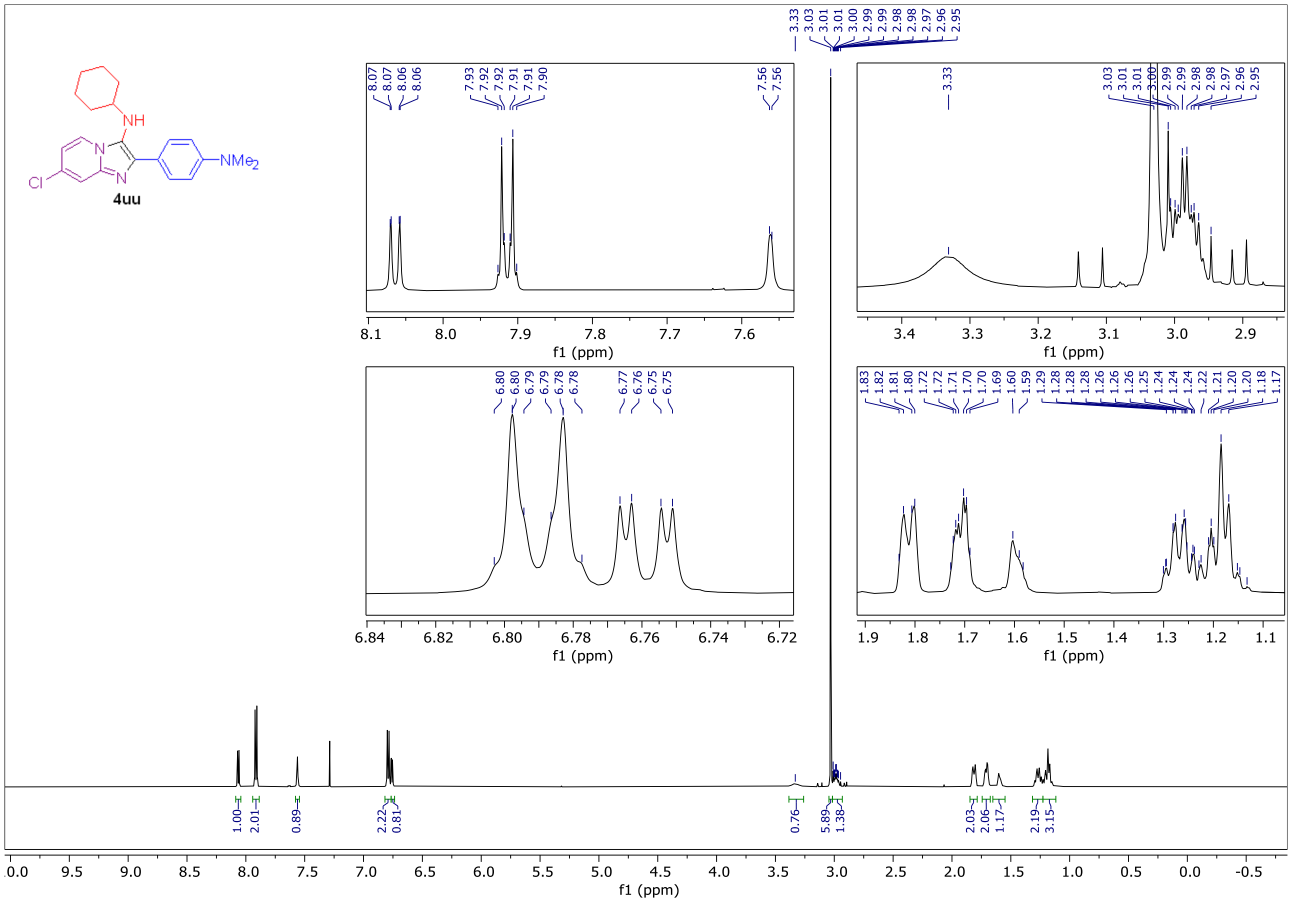

S 186. ^{1}H NMR spectrum (600 MHz, CDCl3) of compound **4uu**.

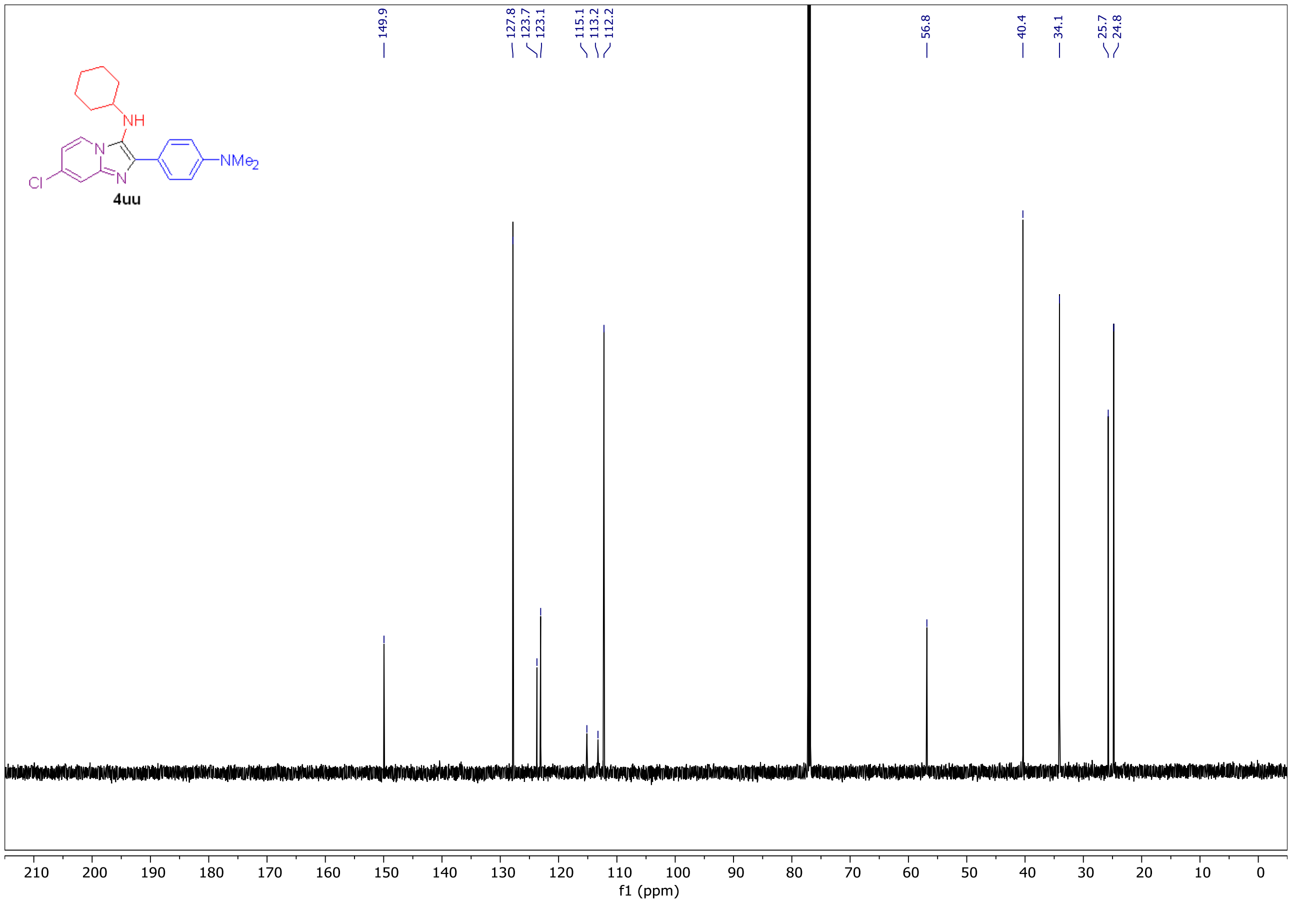

S 187. ^{13}C NMR spectrum (151 MHz, $CDCl_3$) of compound **4uu**.

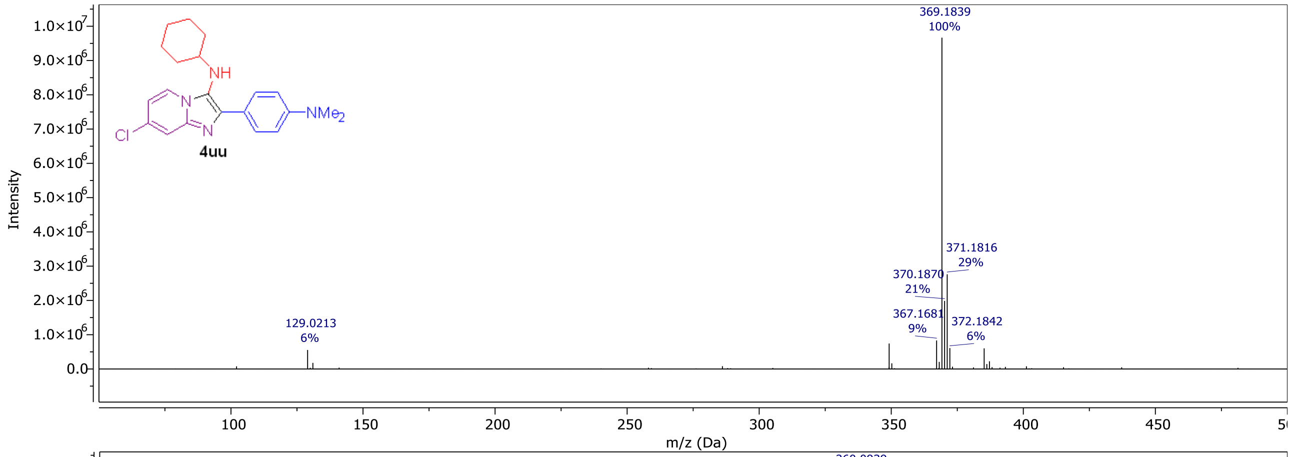

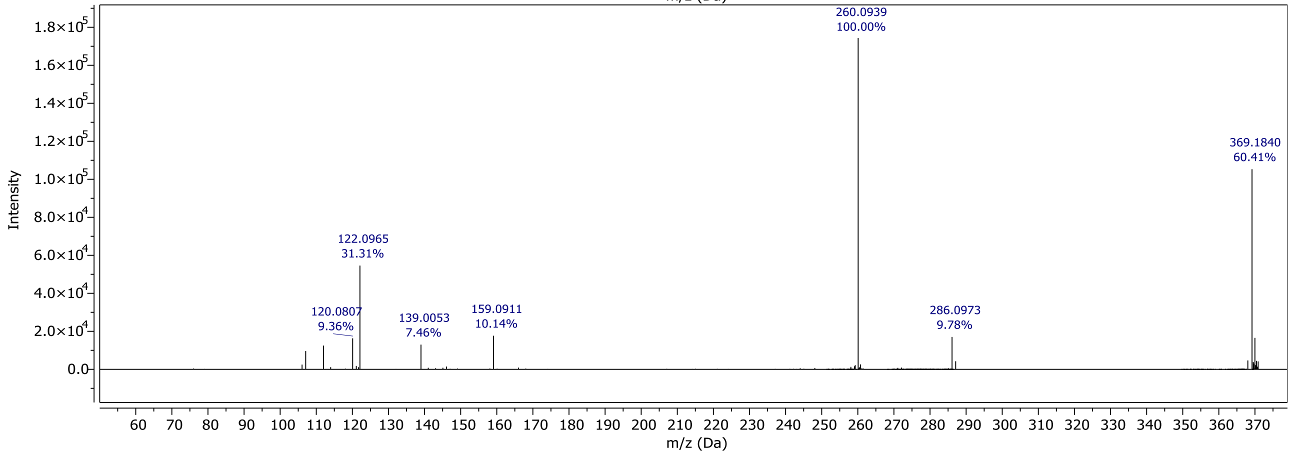

S 188. HRMS (ESI-QTOF) of compound **4uu** and HRMS/MS for $[M+H]^+$.

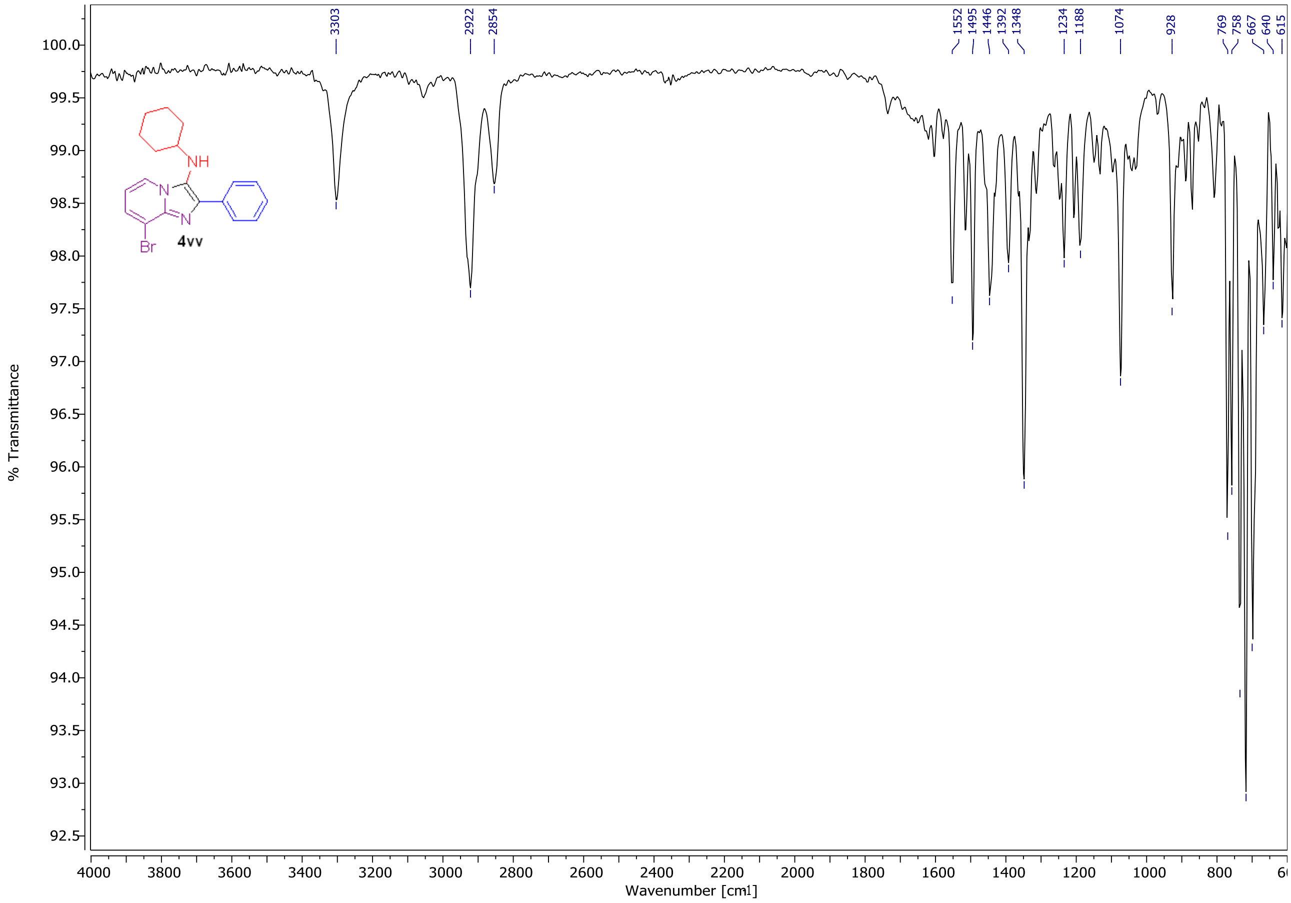

S 189. FT-IR (ATR) of compound **4vv**.

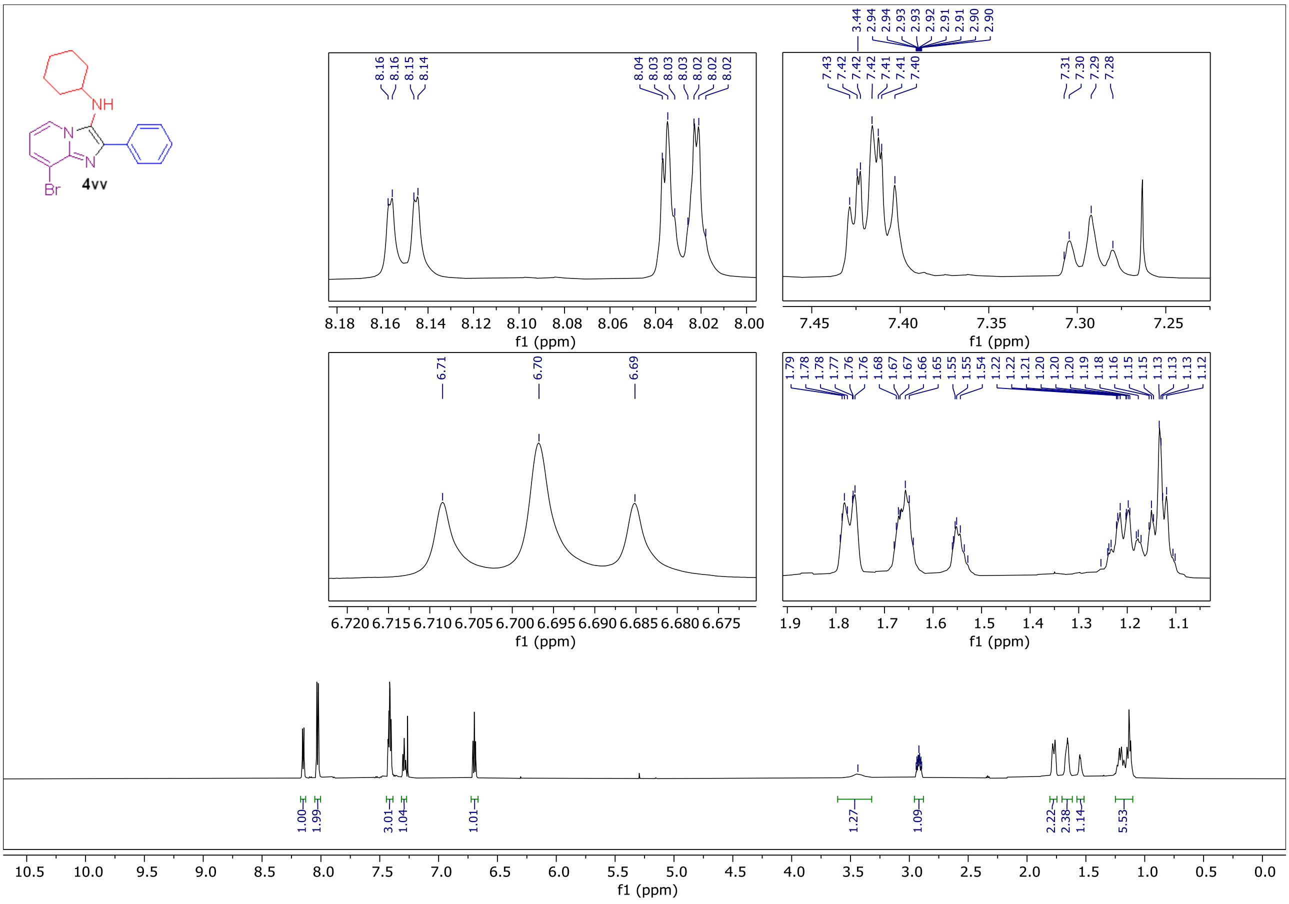

S 190. ^{1}H NMR spectrum (600 MHz, $CDCl_3$) of compound **4vv**.

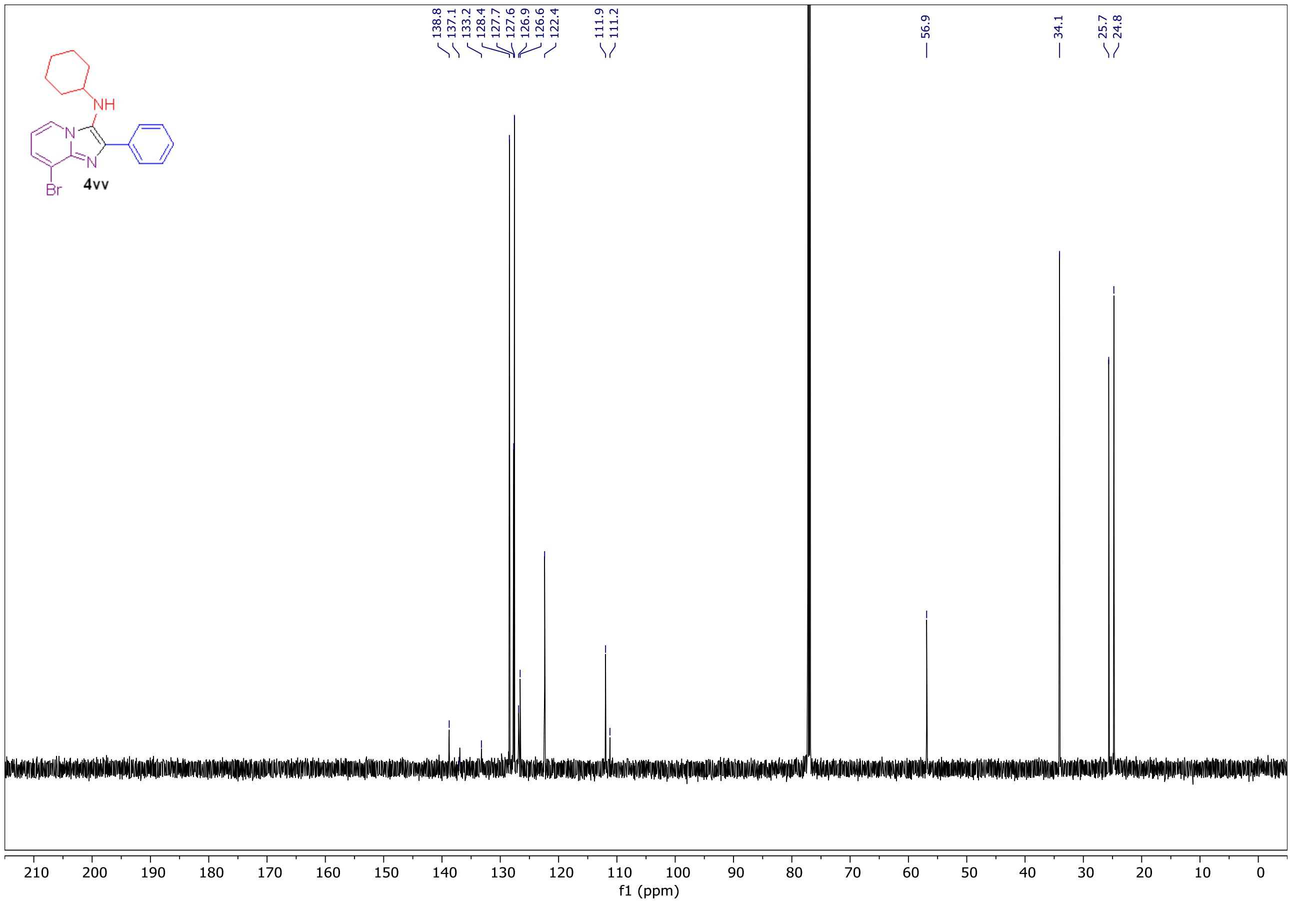

S 191. ^{13}C NMR spectrum (151 MHz, $CDCl_3$) of compound **4vv**.

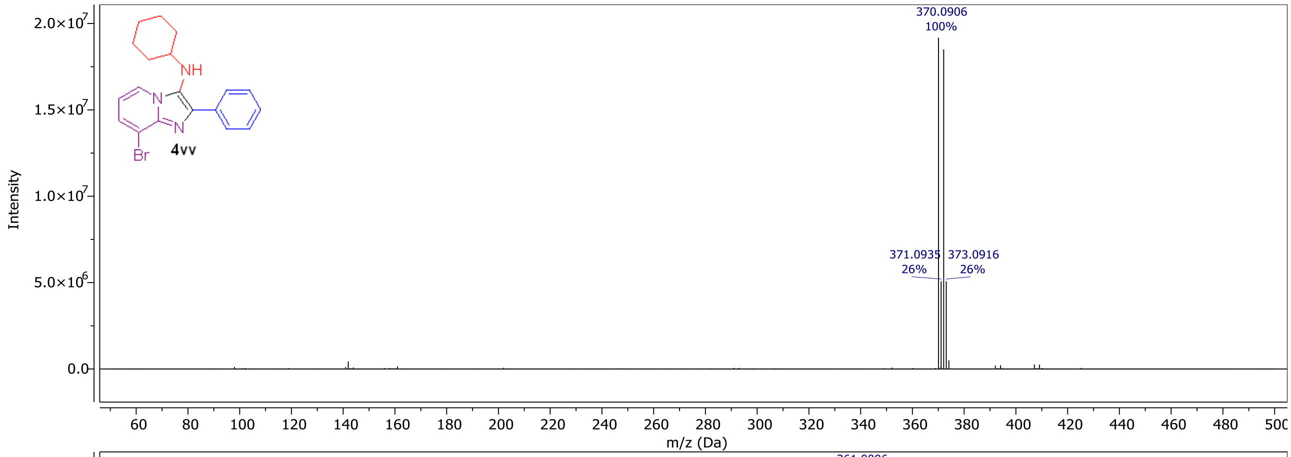

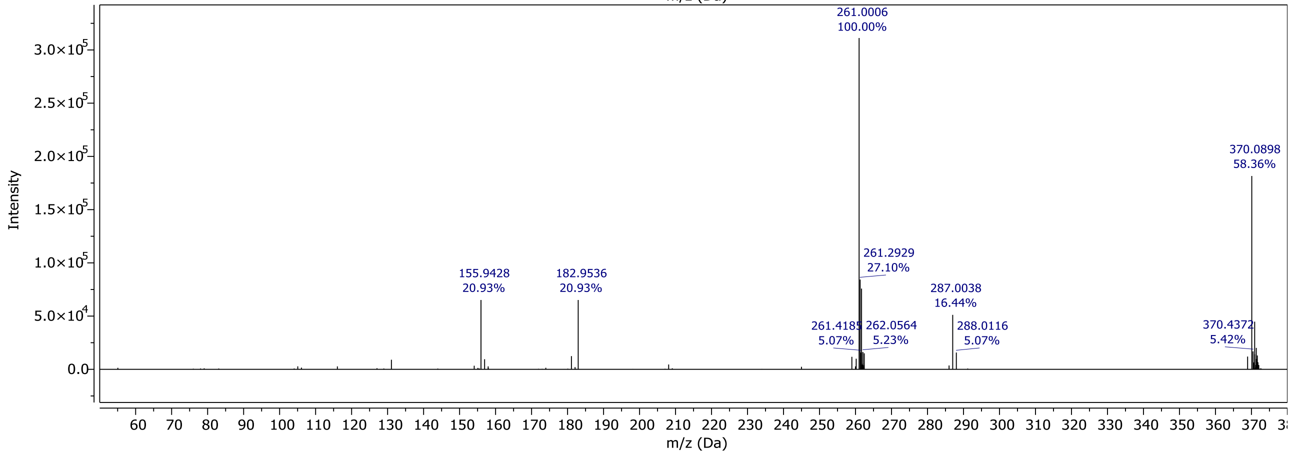

S 192. HRMS (ESI-QTOF) of compound **4vv** and HRMS/MS for $[M+H]^+$.

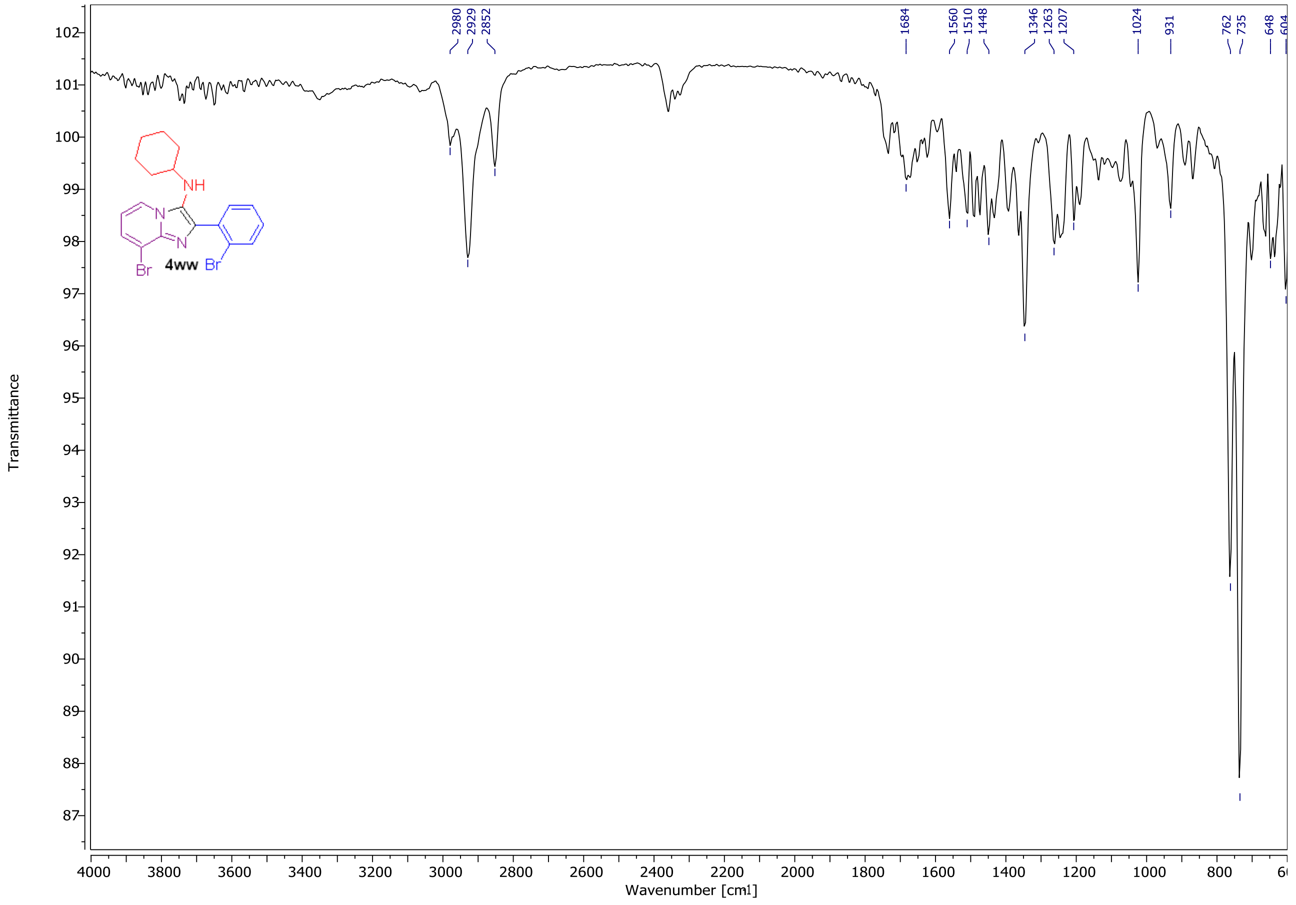

S 193. FT-IR (ATR) of compound **4ww**.

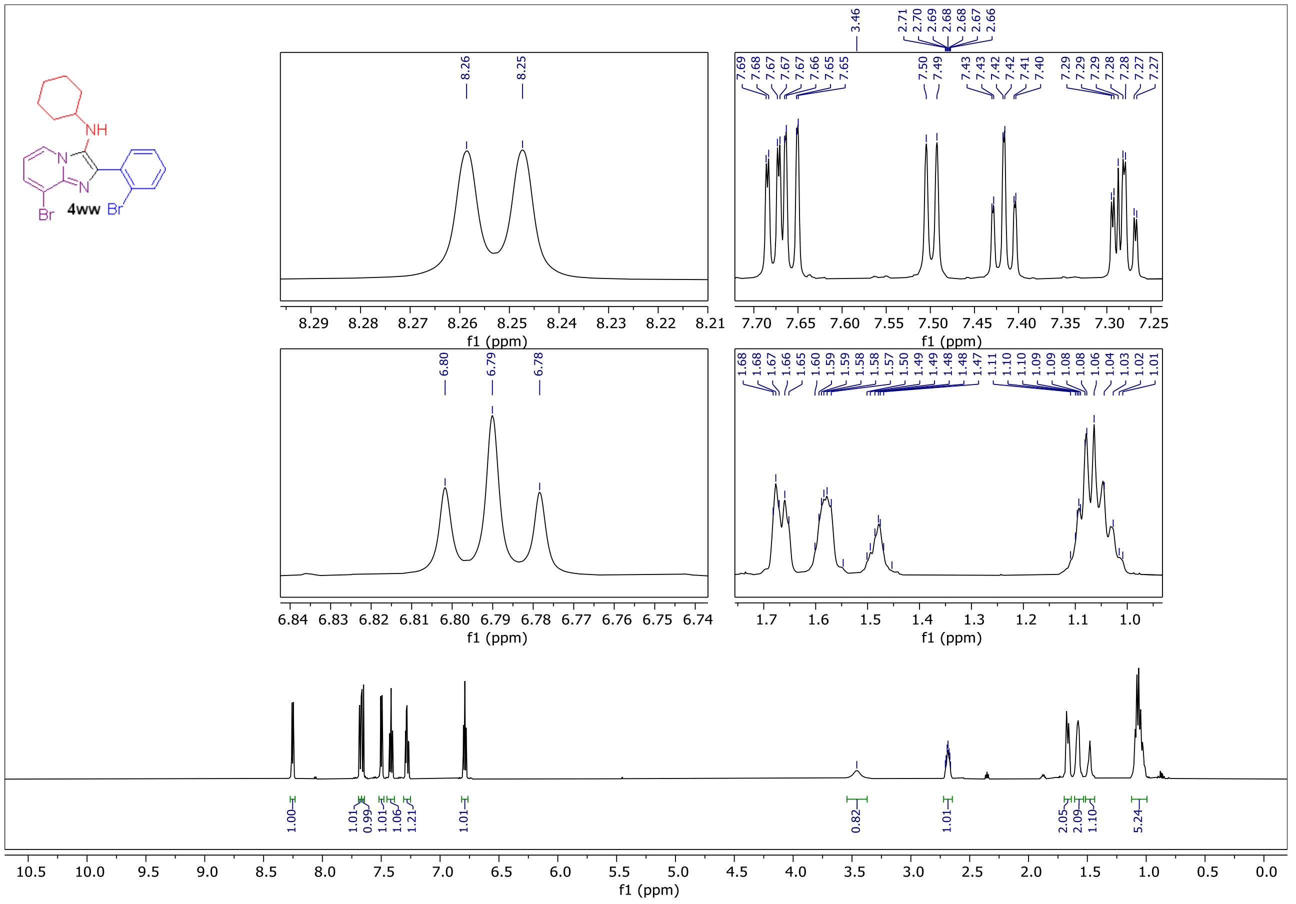

S 194. ^{1}H NMR spectrum (600 MHz, $CDCl_3$) of compound **4ww**.

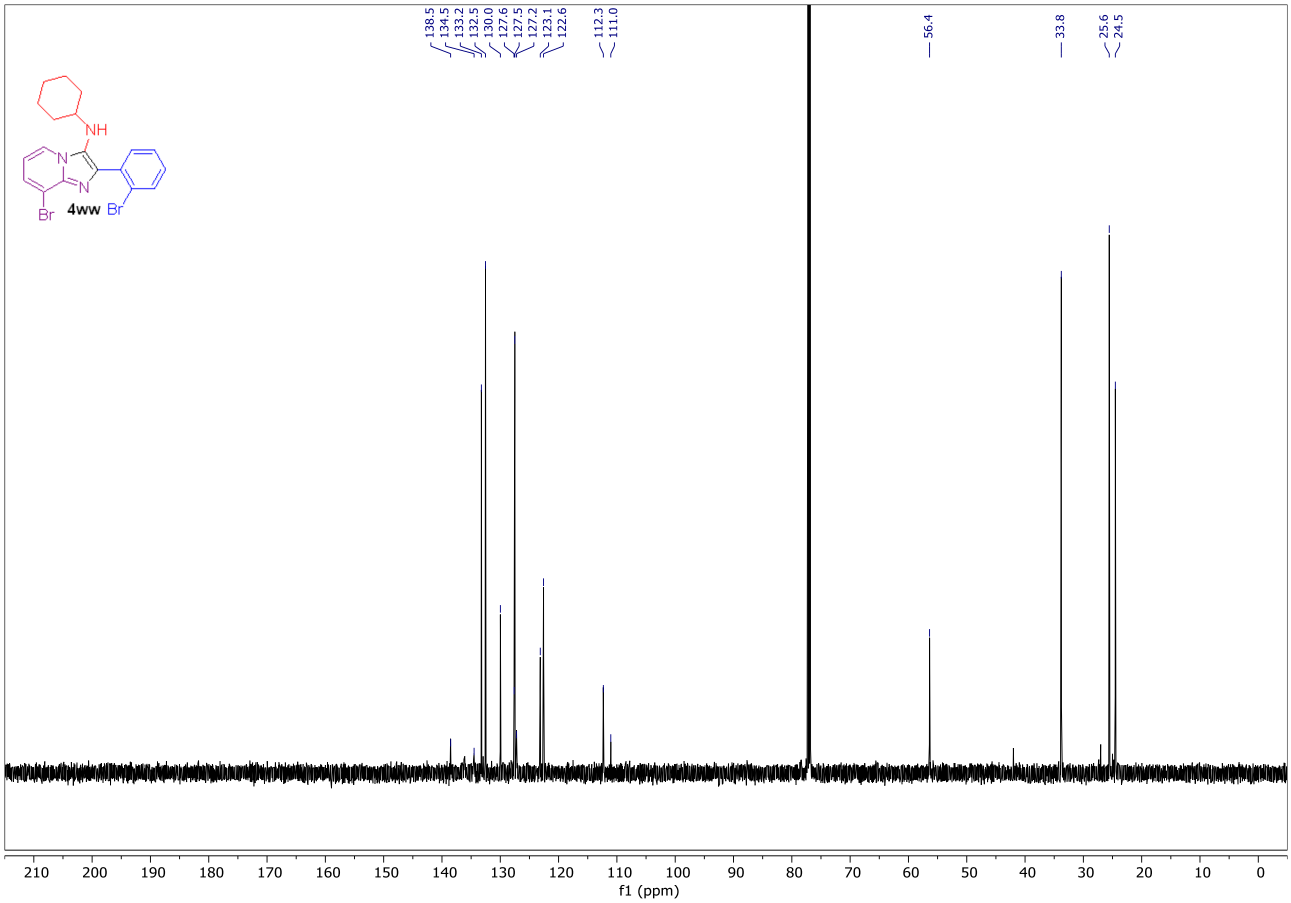

S 195. ^{13}C NMR spectrum (151 MHz, CDCl$_3$) of compound **4ww**.

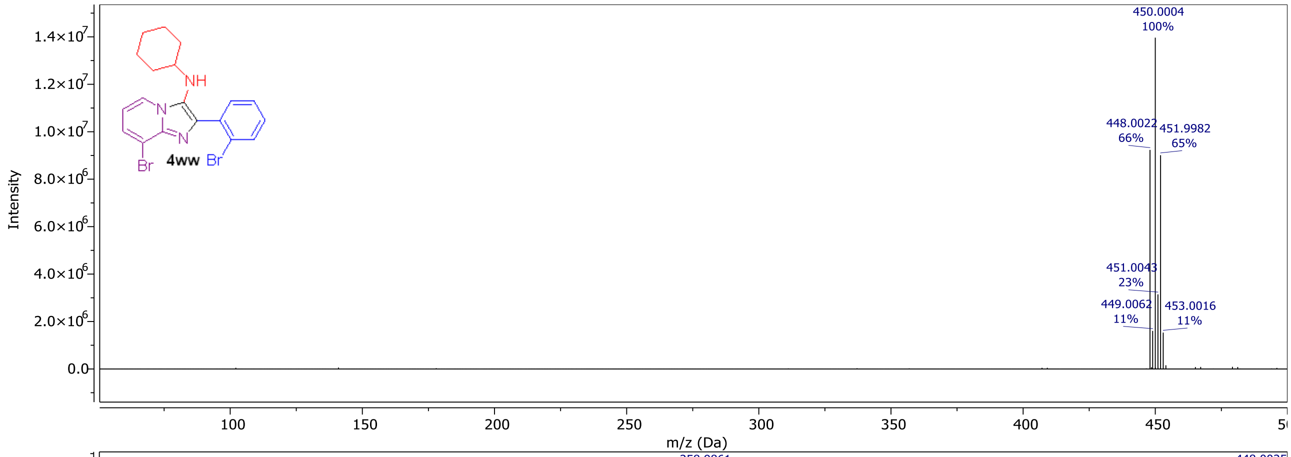

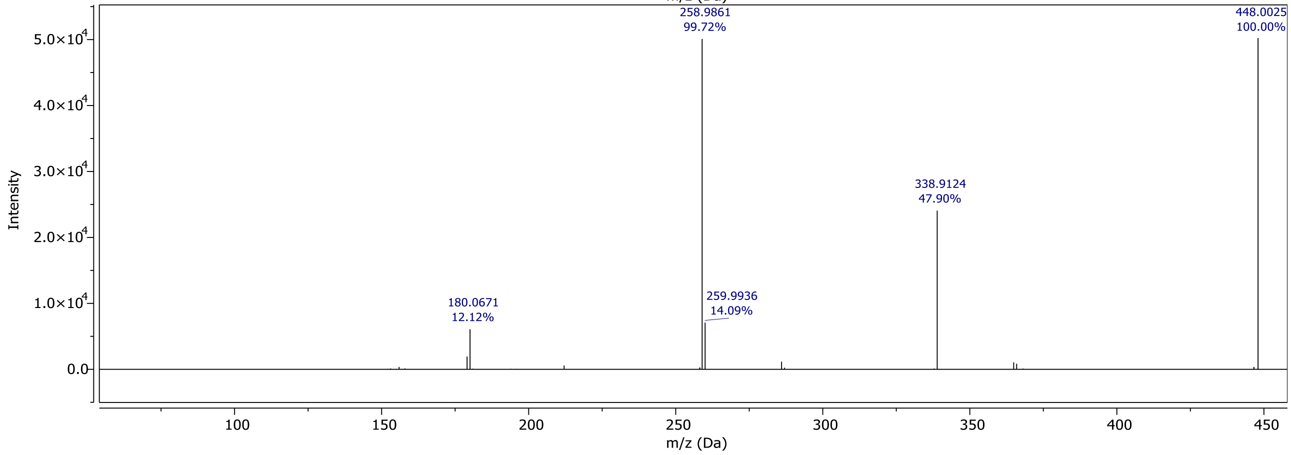

S 196. HRMS (ESI-QTOF) of compound **4ww** and HRMS/MS for [M+H]$^+$.

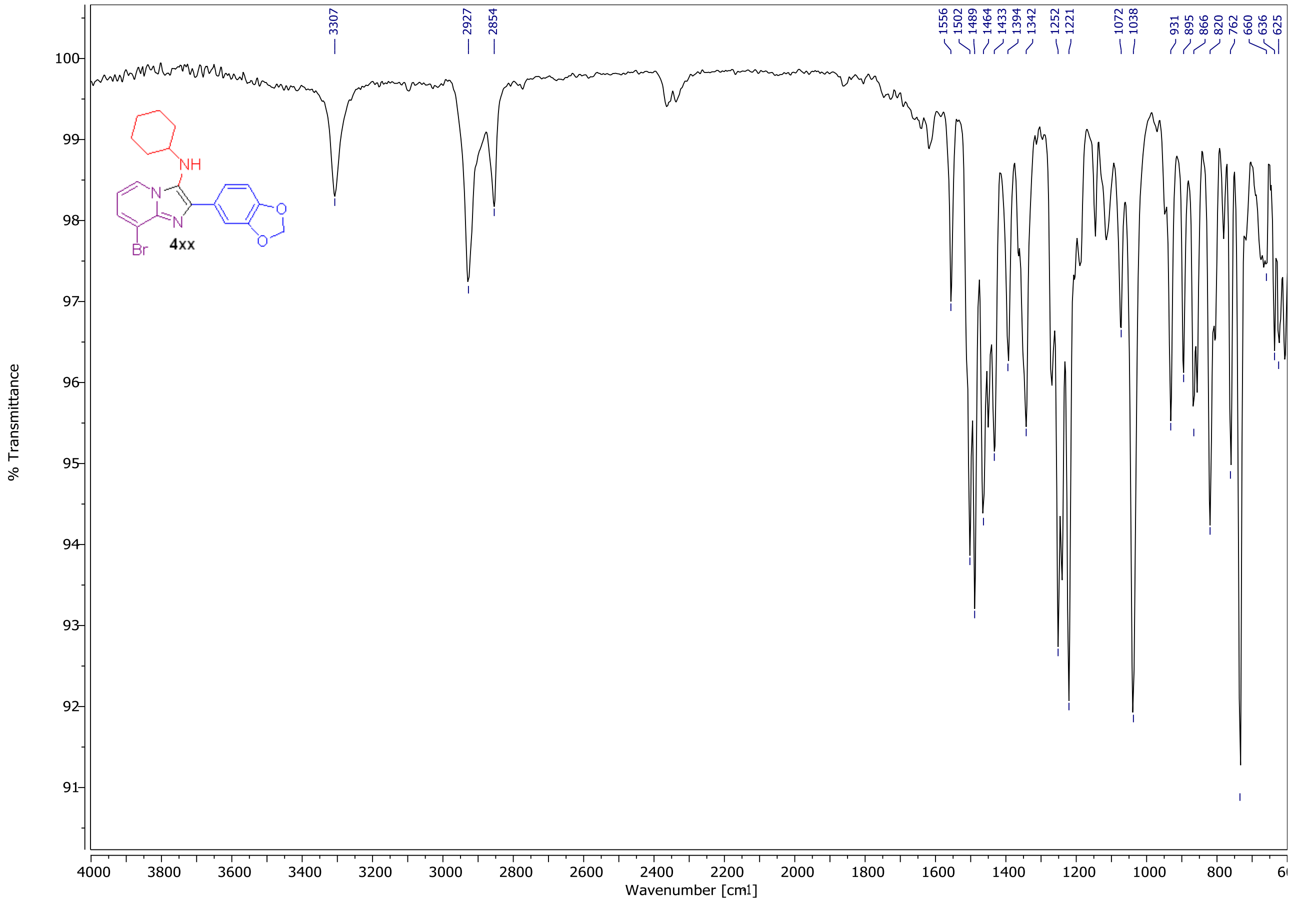

S 197. FT-IR (ATR) of compound **4xx**.

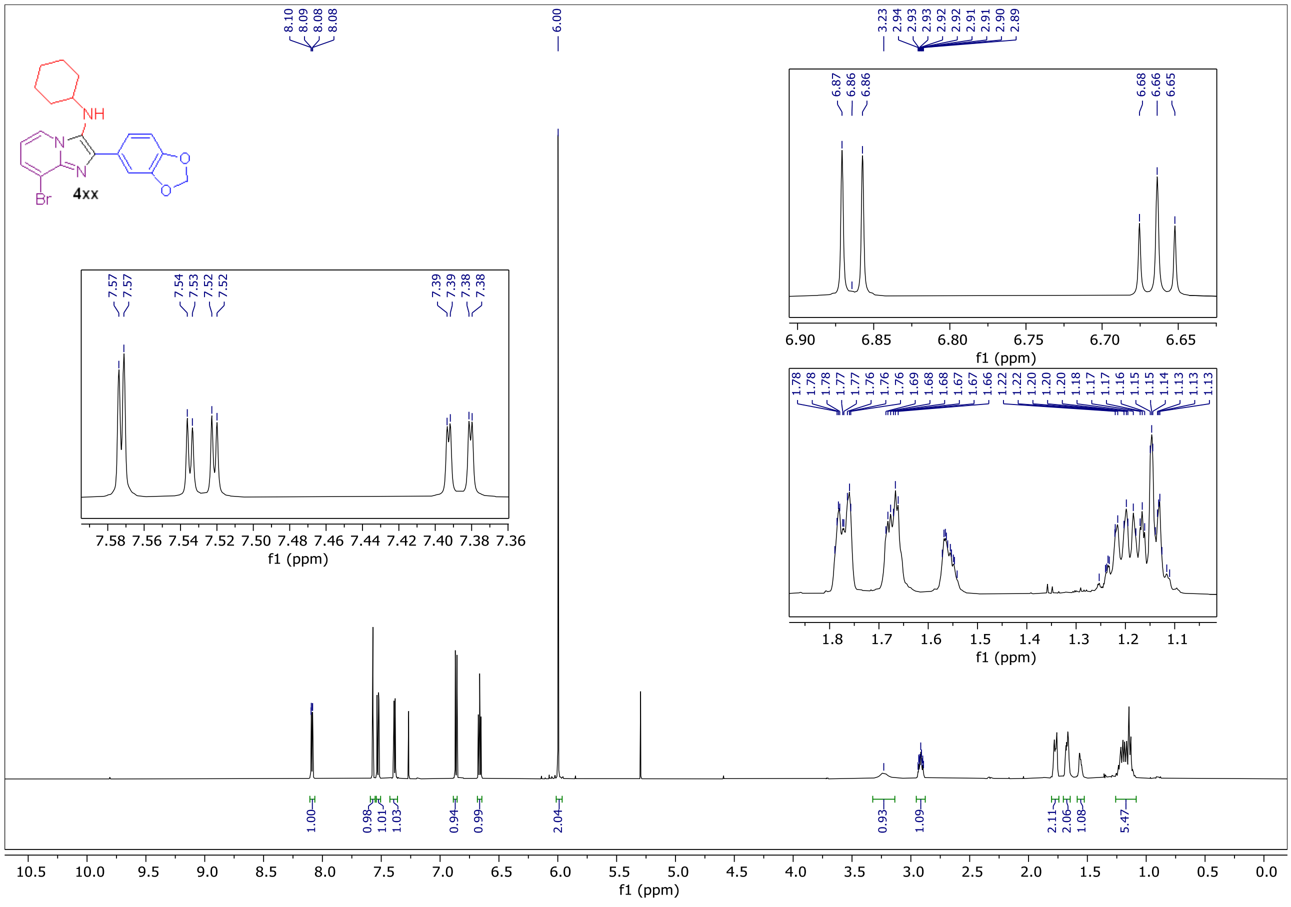

S 198. ^{1}H NMR spectrum (600 MHz, $CDCl_3$) of compound **4xx**.

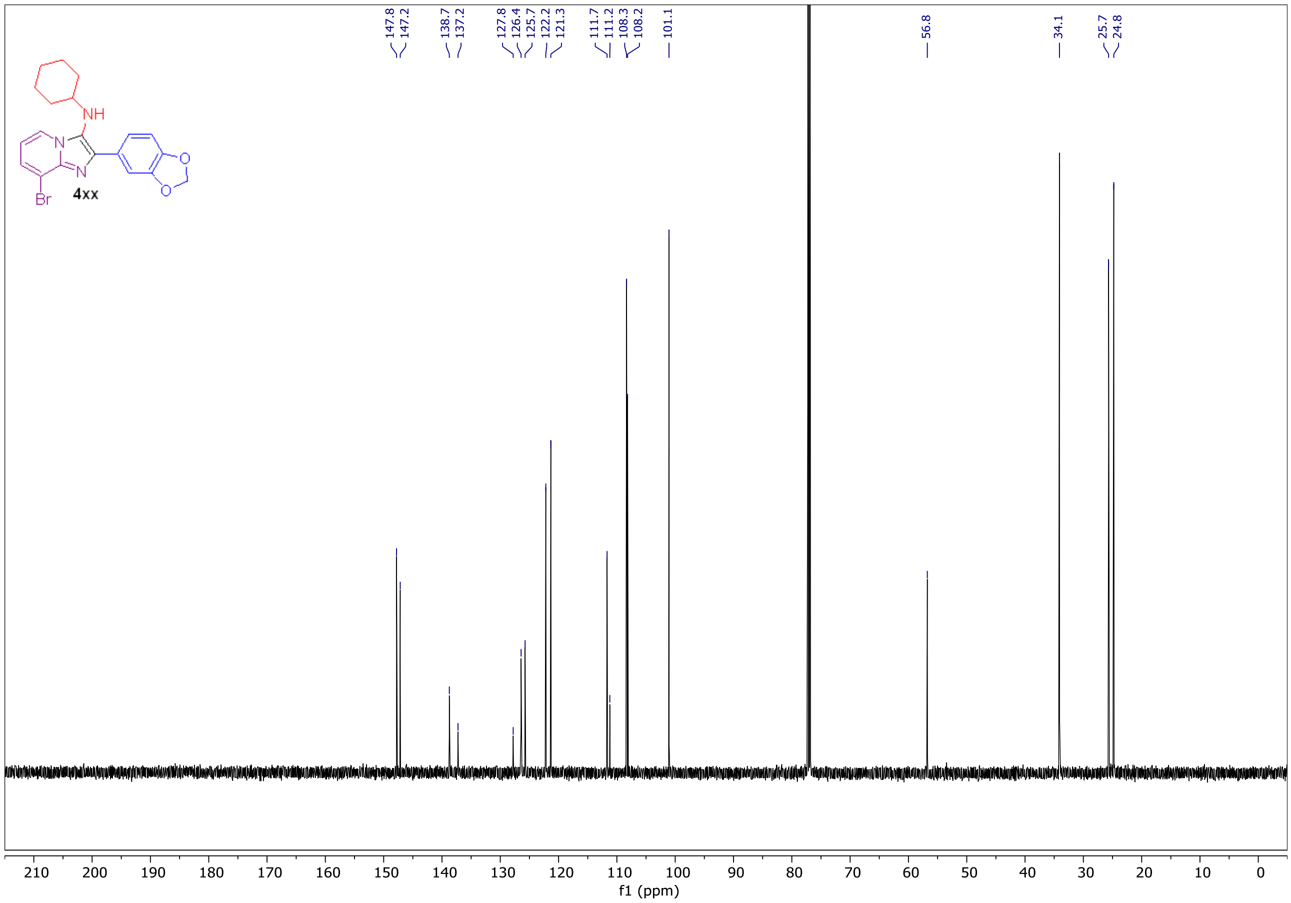

S 199. ^{13}C NMR spectrum (151 MHz, $CDCl_3$) of compound **4xx**.

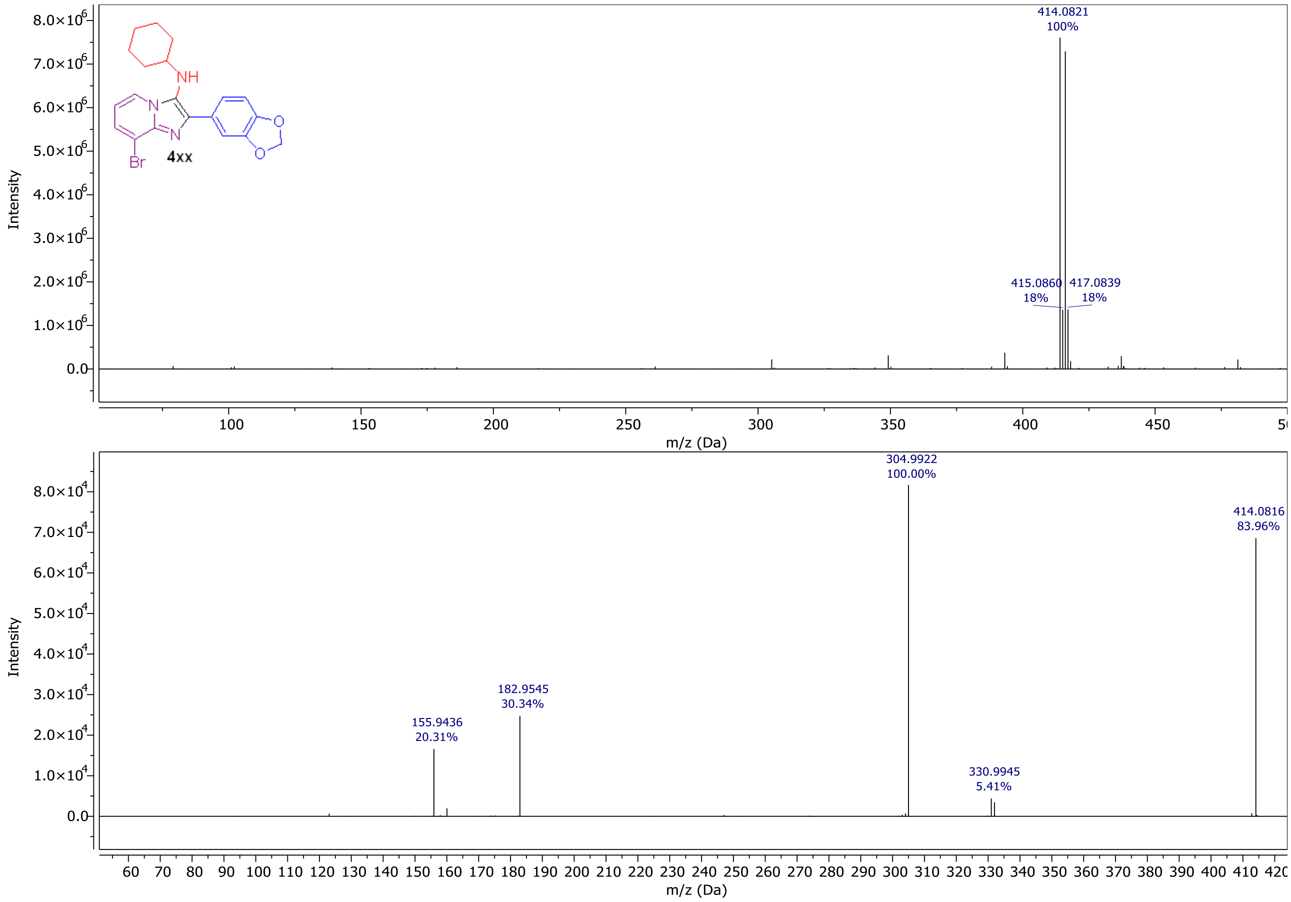

S 200. HRMS (ESI-QTOF) of compound **4xx** and HRMS/MS for $[M+H]^+$.

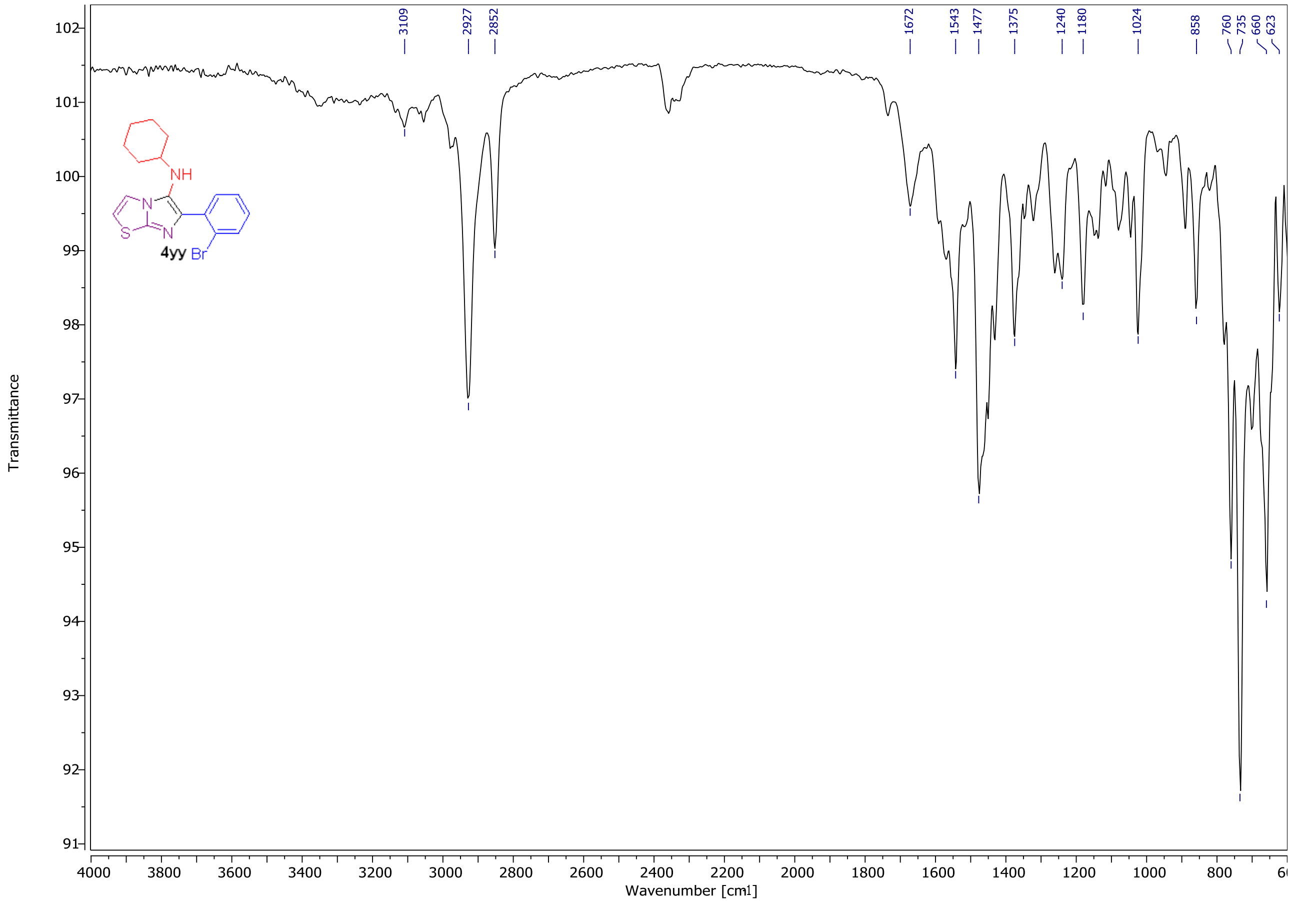

S 201. FT-IR (ATR) of compound **4yy**.

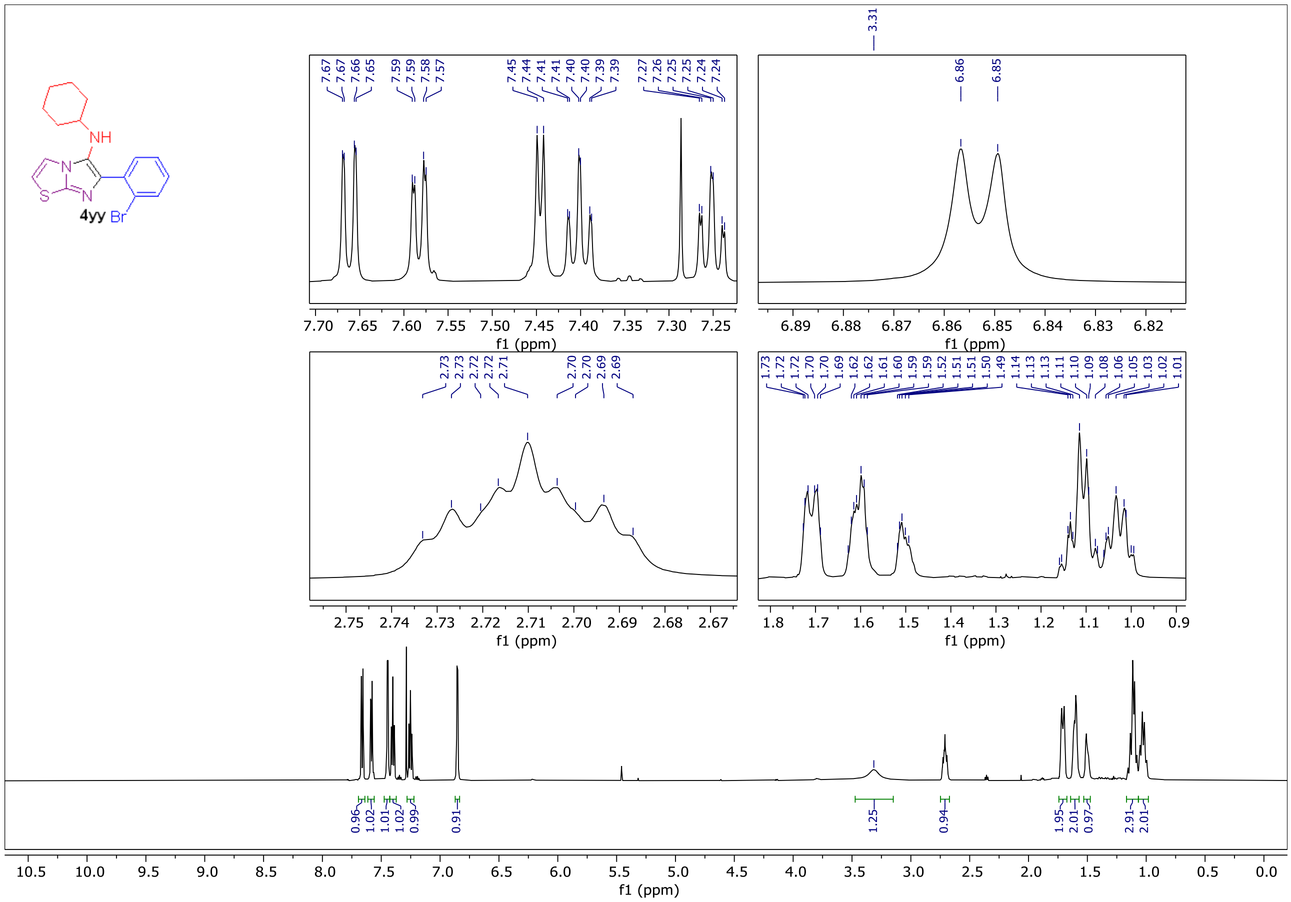

S 202. ^{1}H NMR spectrum (600 MHz, $CDCl_3$) of compound **4yy**.

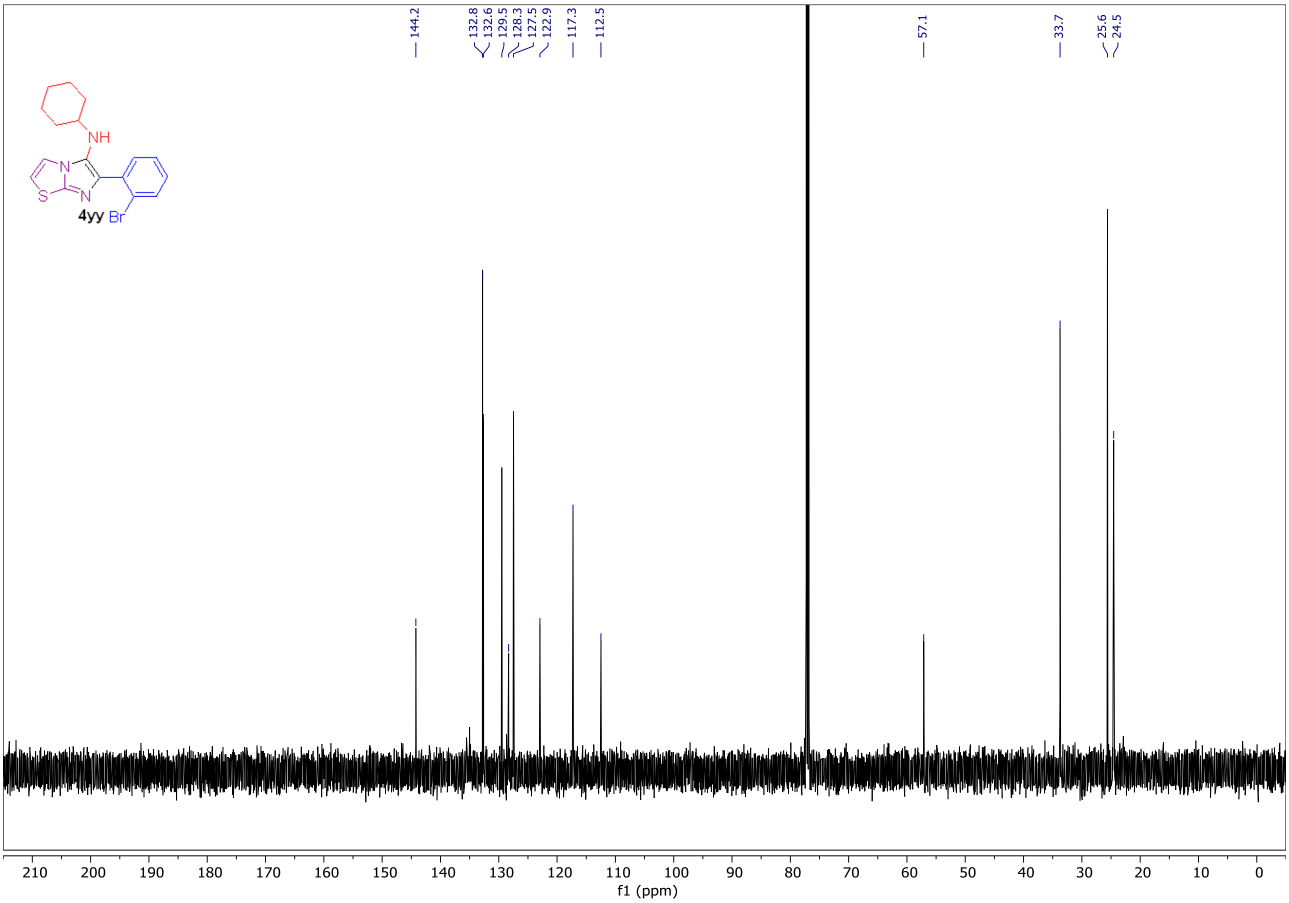

S 203. ^{13}C NMR spectrum (151 MHz, $CDCl_3$) of compound **4yy**.

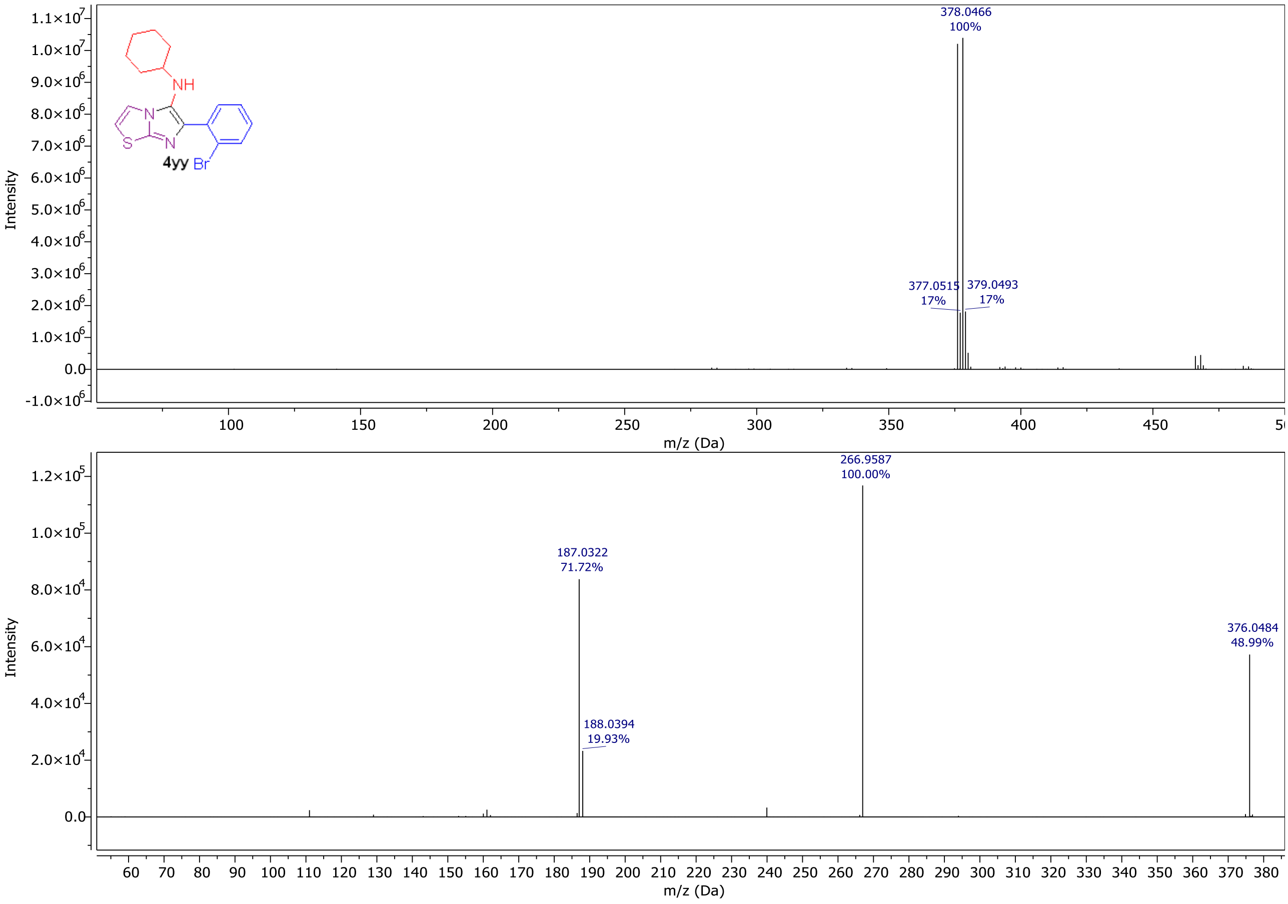

S 204. HRMS (ESI-QTOF) of compound **4yy** and HRMS/MS for $[M+H]^+$.

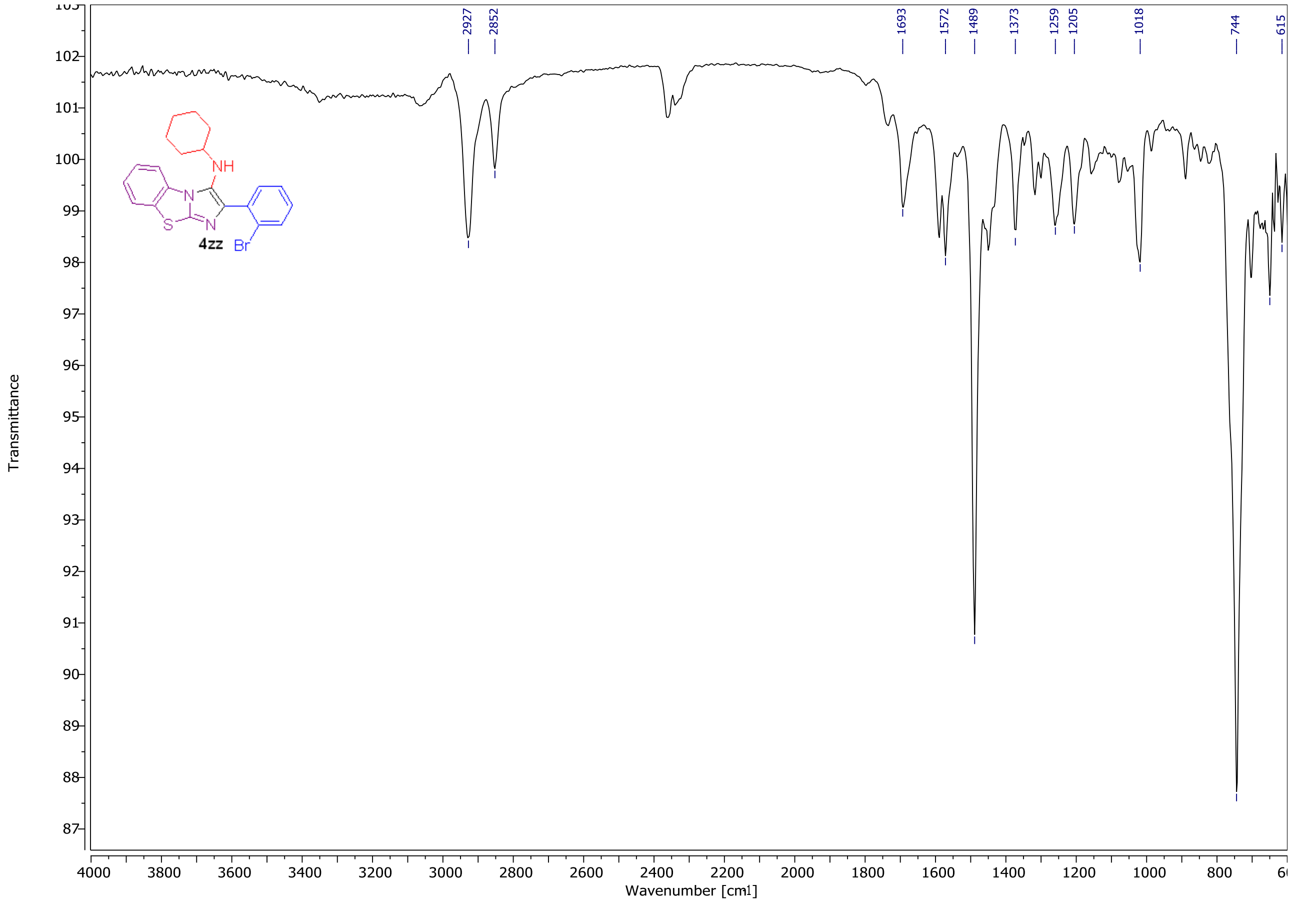

S 205. FT-IR (ATR) of compound **4zz**.

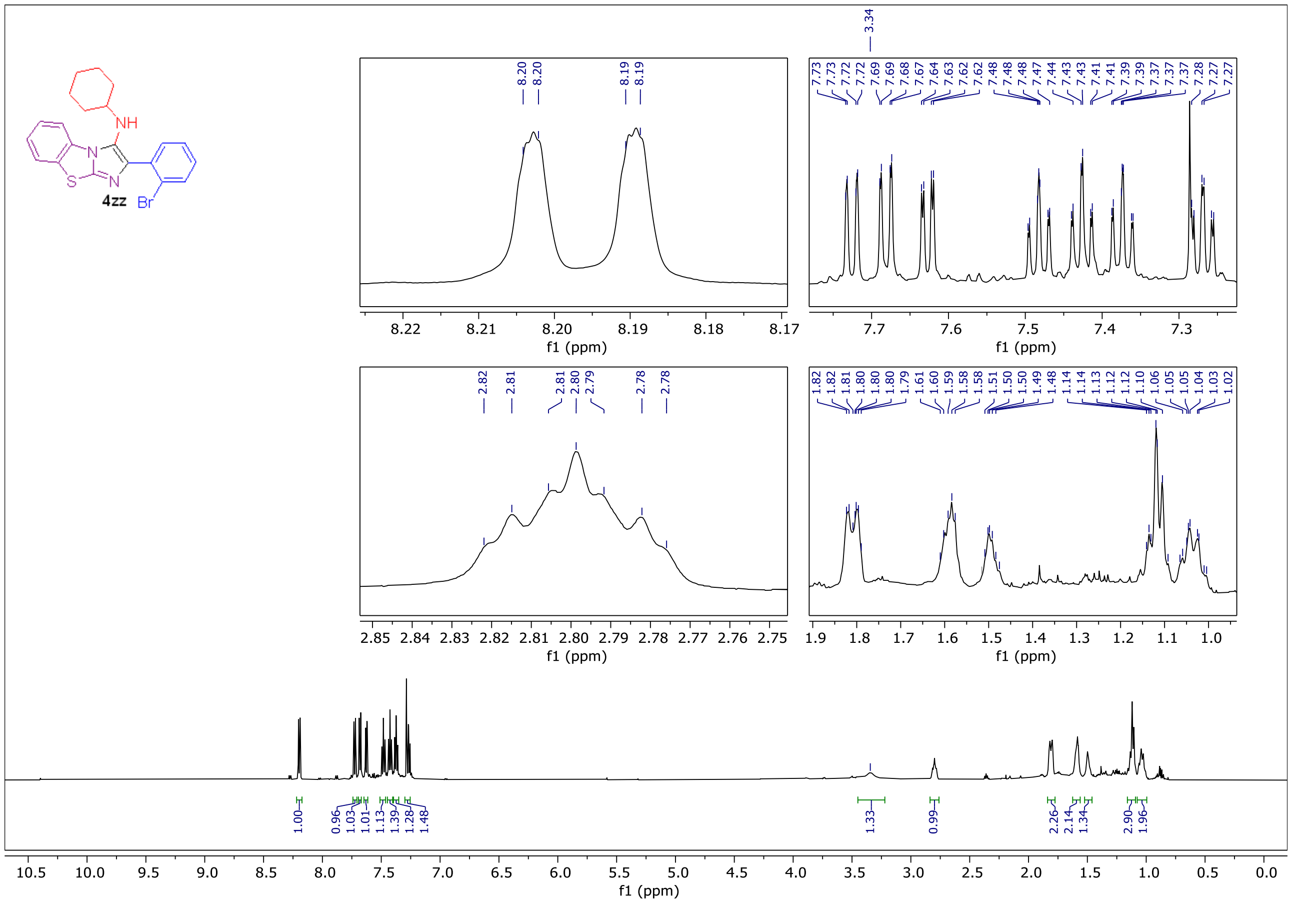

S 206. ^{1}H NMR spectrum (600 MHz, $CDCl_3$) of compound **4zz**.

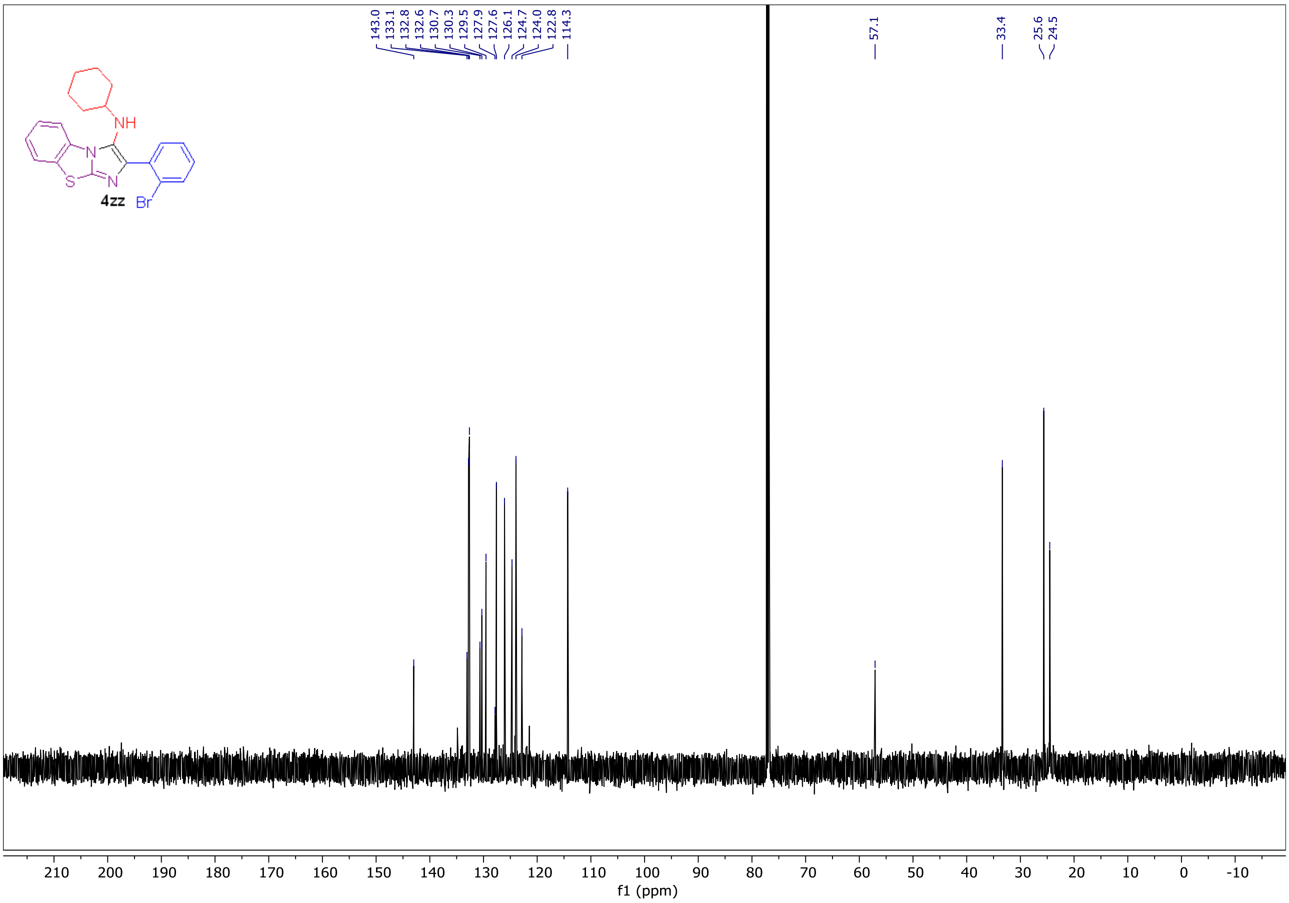

S 207. ^{13}C NMR spectrum (151 MHz, $CDCl_3$) of compound **4zz**.

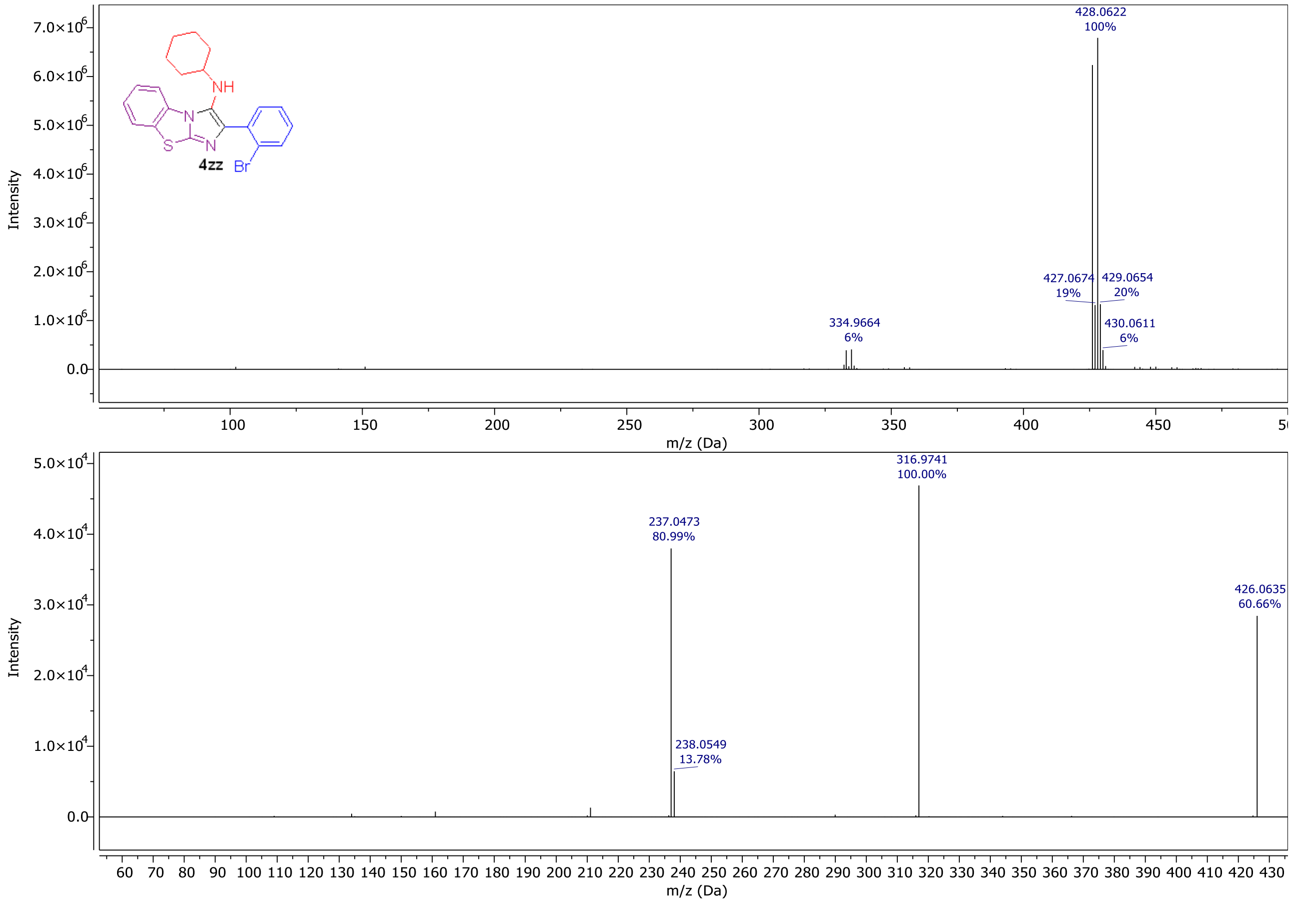

S 208. HRMS (ESI-QTOF) of compound **4zz** and HRMS/MS for $[M+H]^+$.

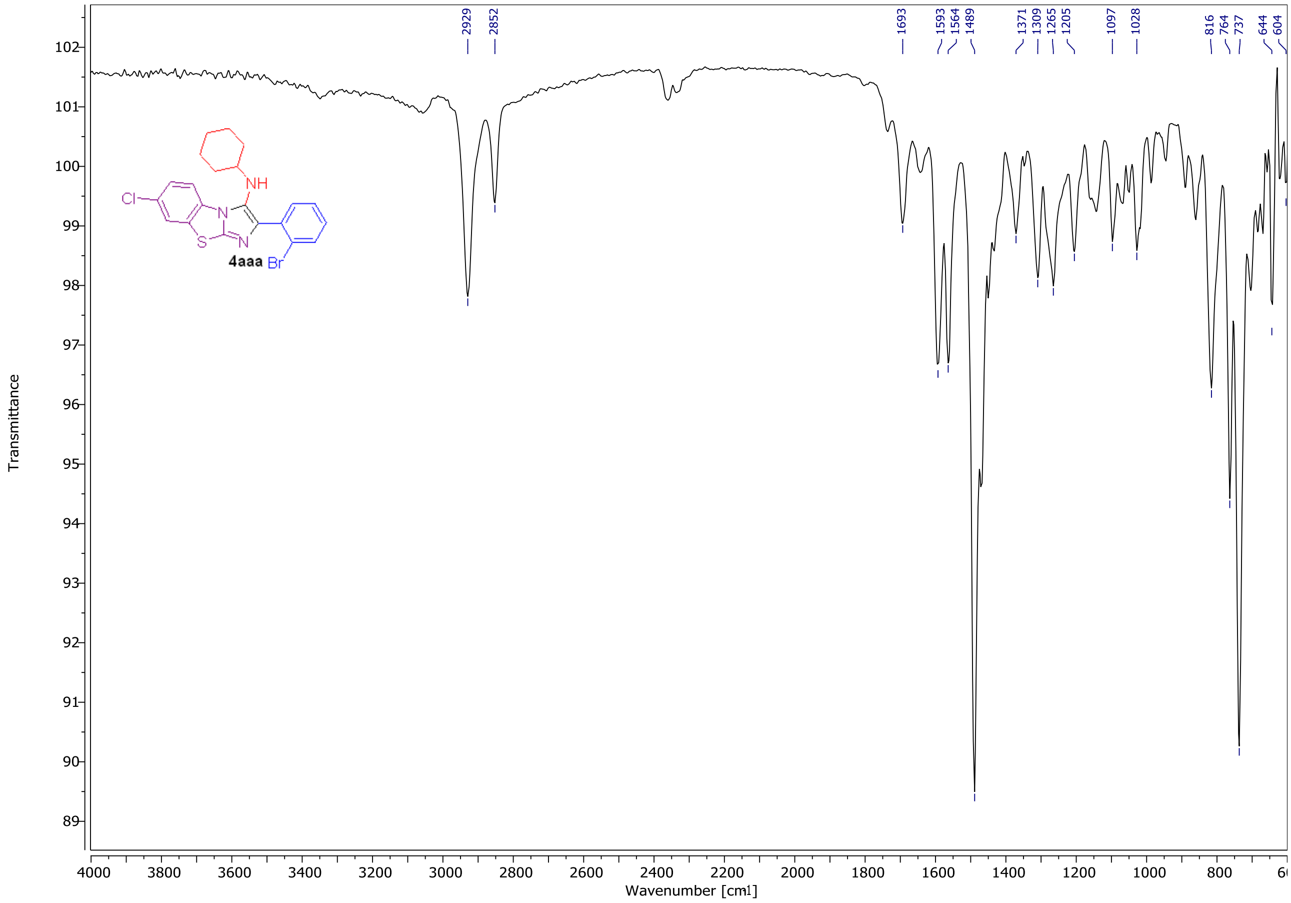

S 209. FT-IR (ATR) of compound **4aaa**.

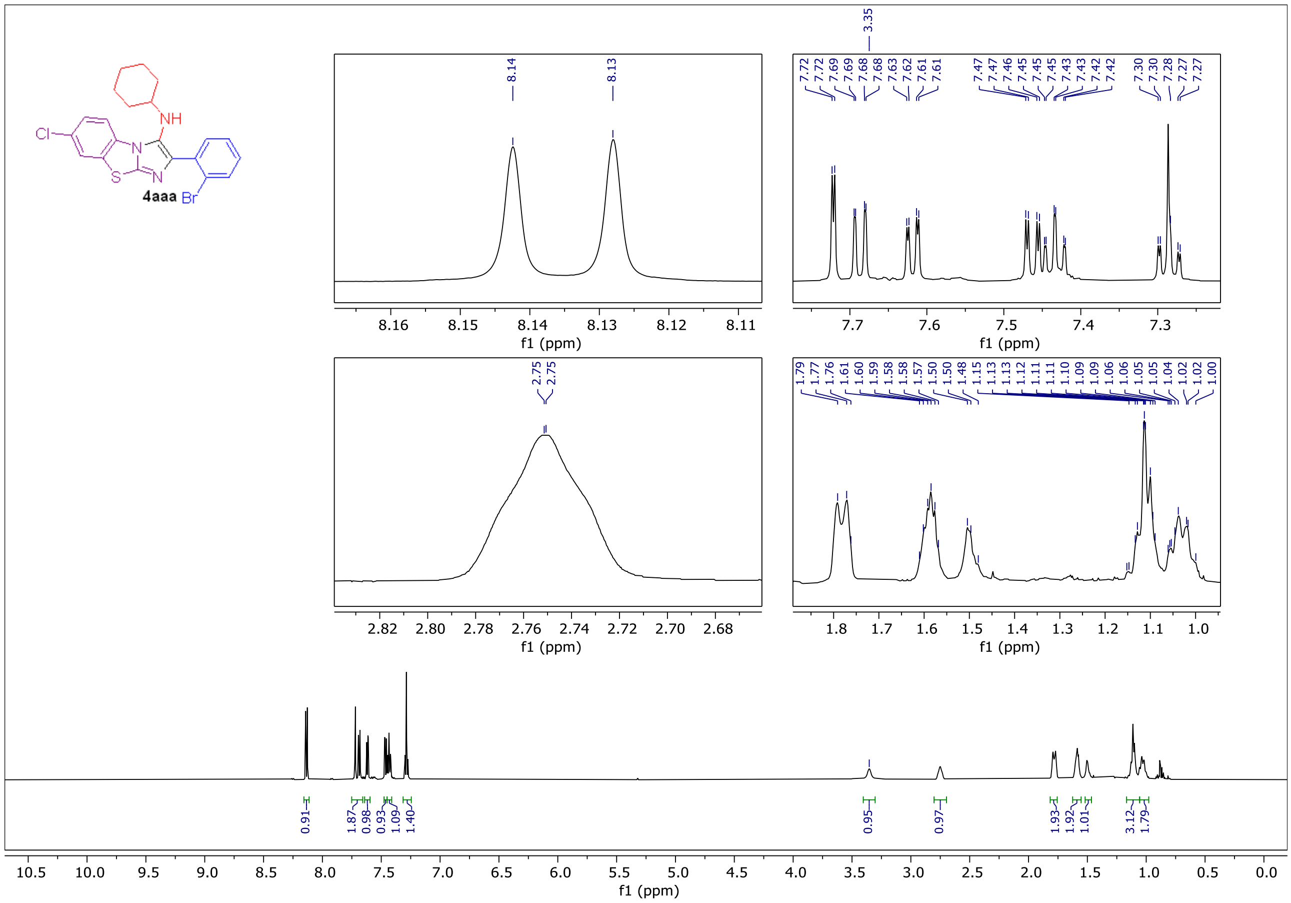

S 210. ^{1}H NMR spectrum (600 MHz, $CDCl_3$) of compound **4aaa**.

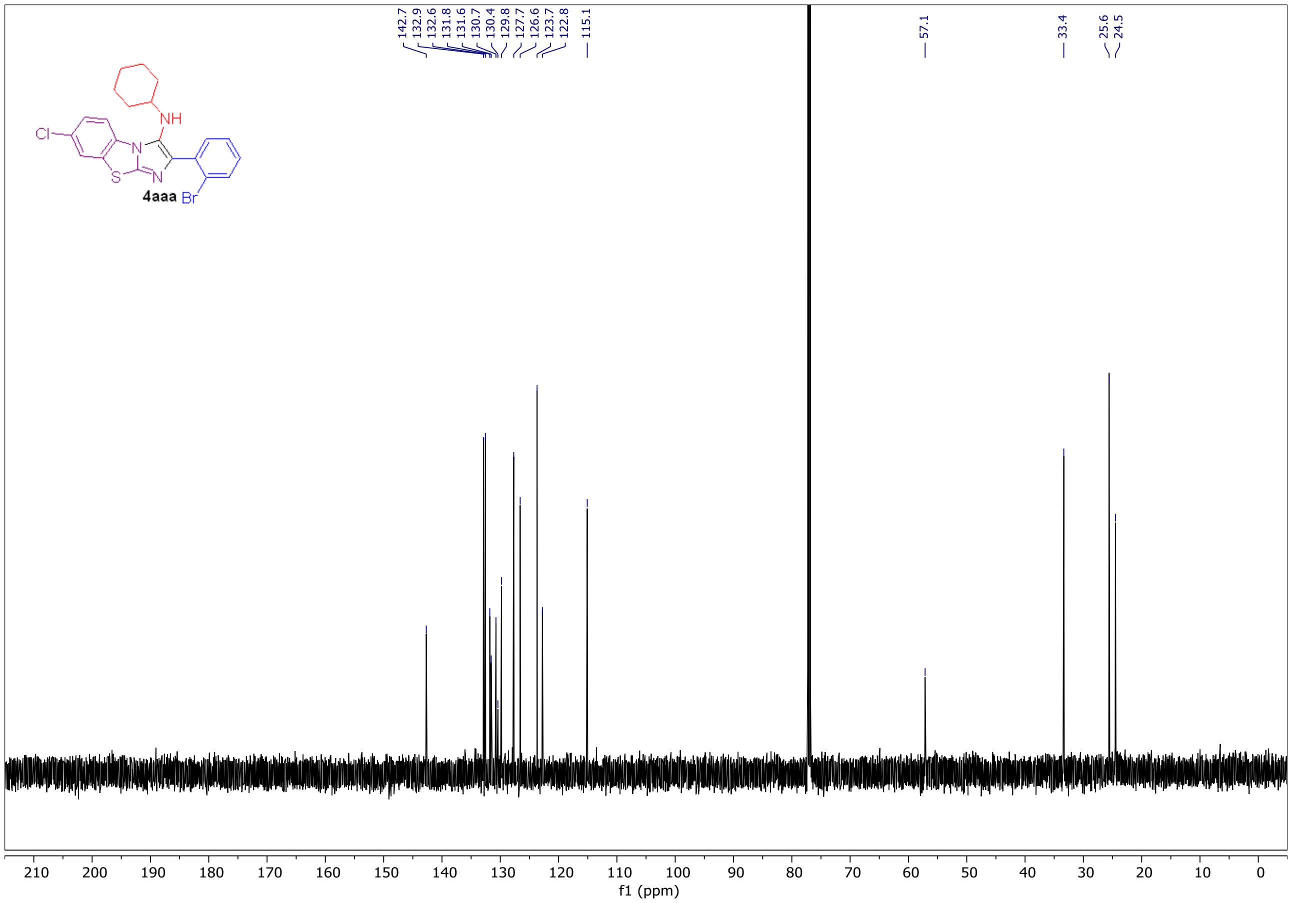

S 211. ^{13}C NMR spectrum (151 MHz, $CDCl_3$) of compound **4aaa**.

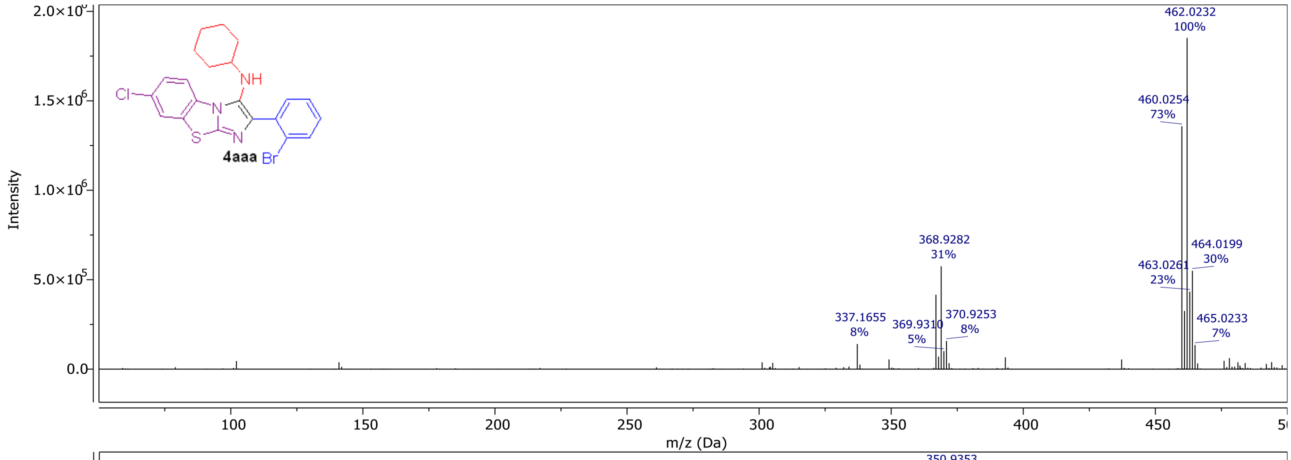

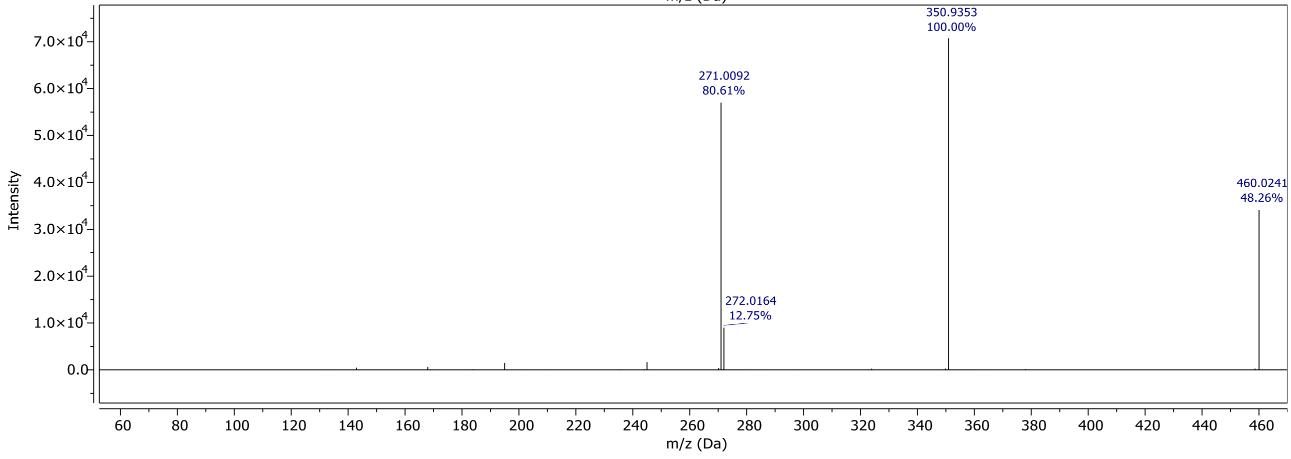

S 212. HRMS (ESI-QTOF) of compound **4aaa** and HRMS/MS for $[M+H]^+$.

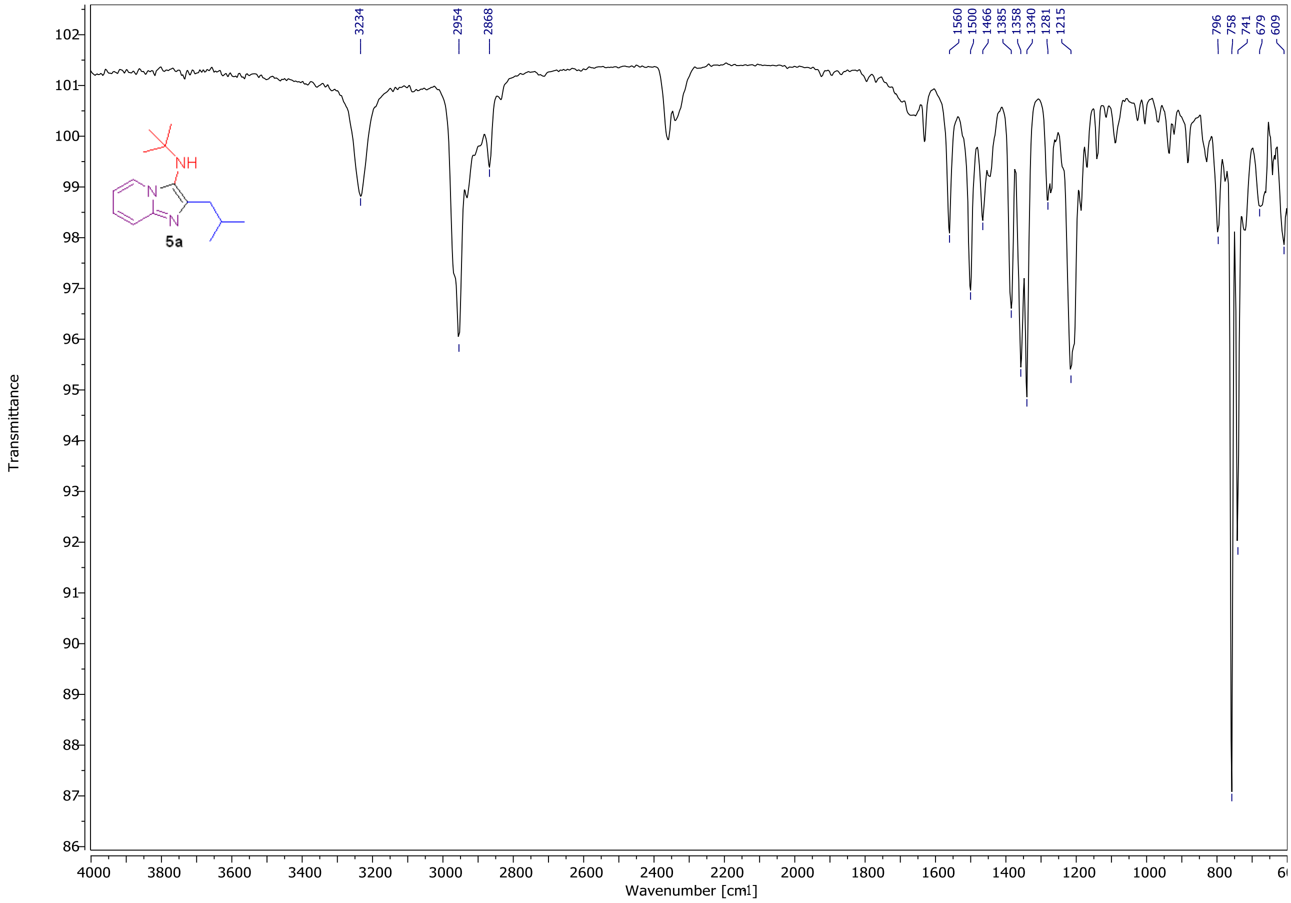

S 213. FT-IR (ATR) of compound **5a**.

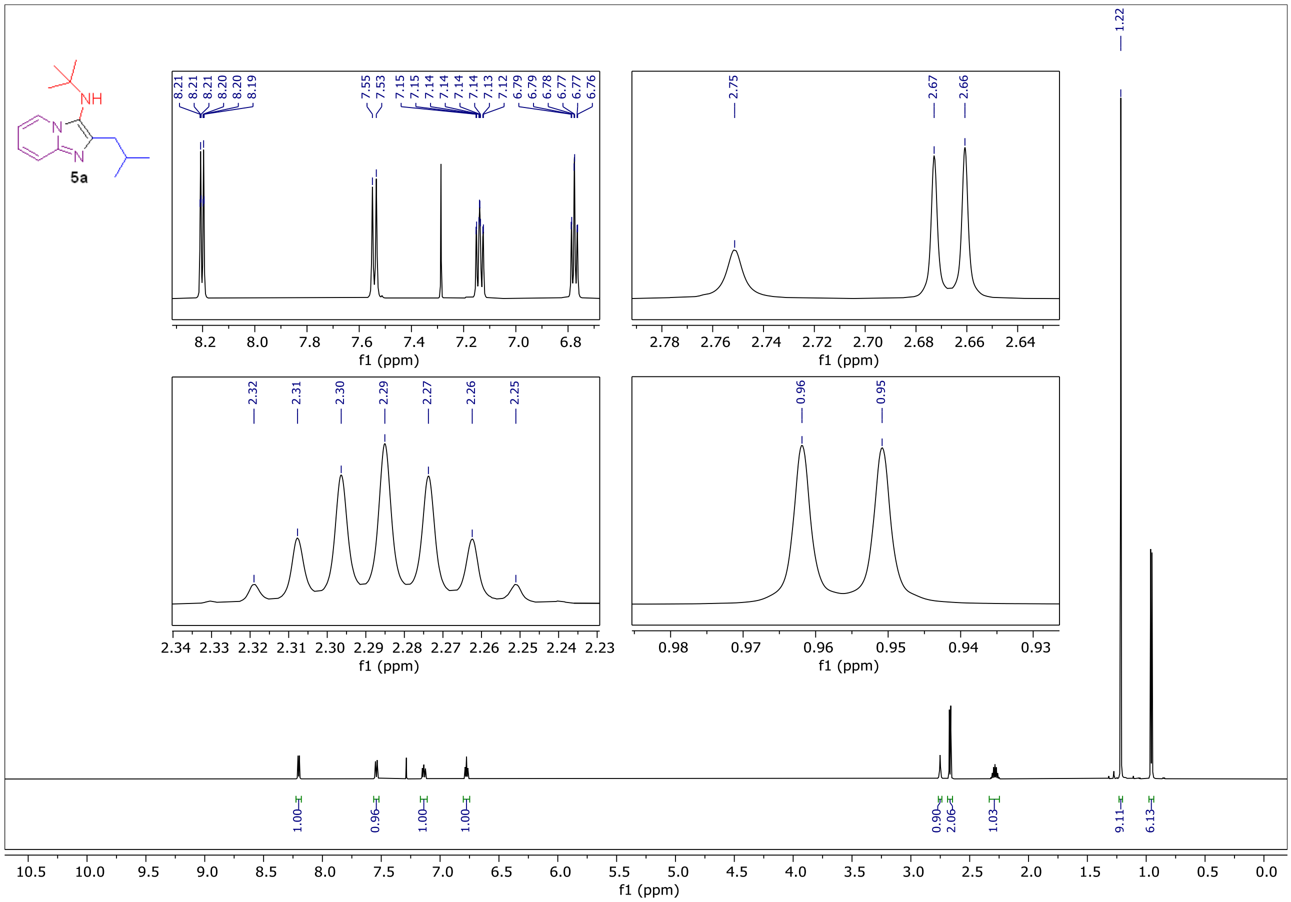

S 214. ^{1}H NMR spectrum (600 MHz, $CDCl_3$) of compound **5a**.

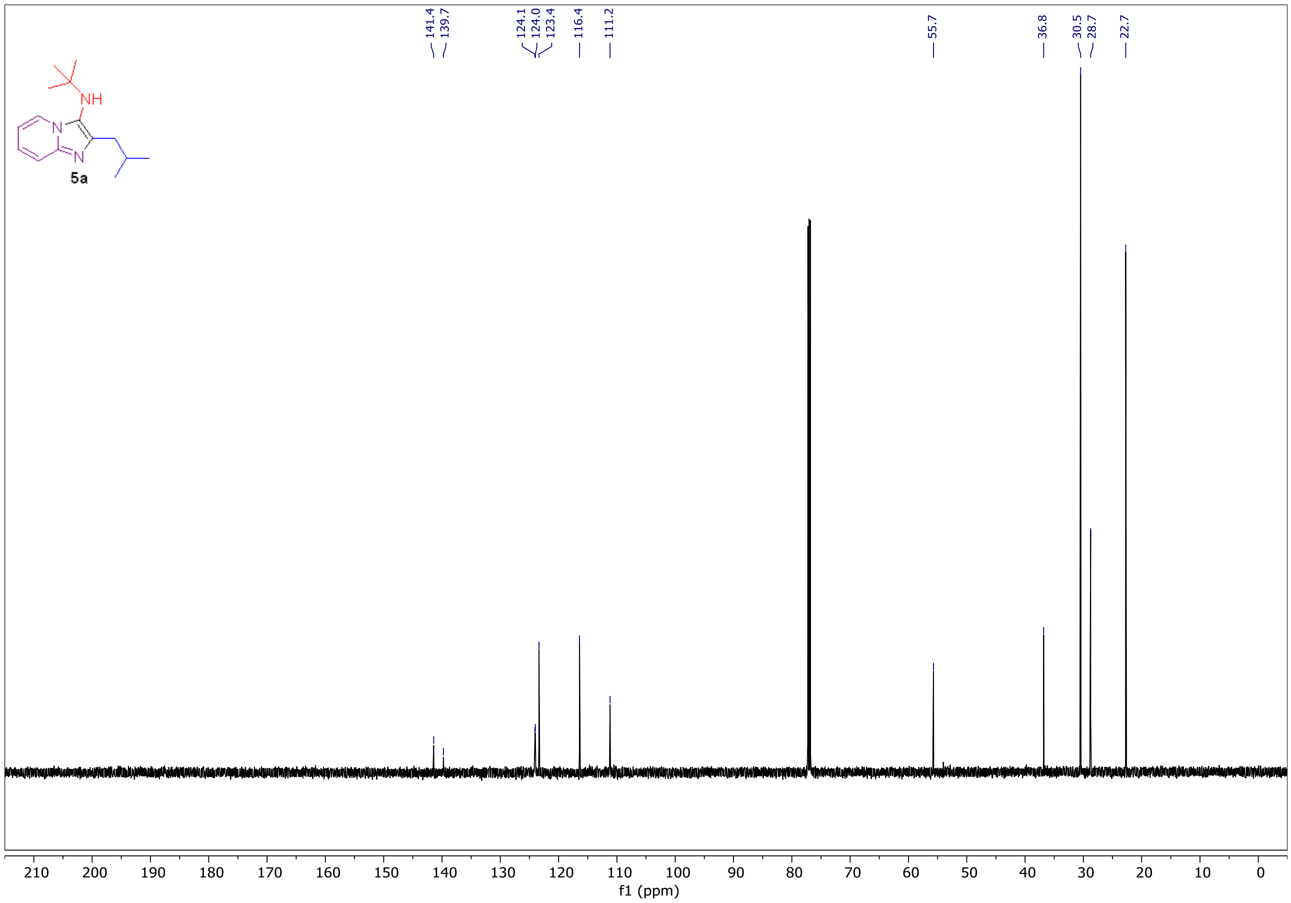

S 215. ^{13}C NMR spectrum (151 MHz, $CDCl_3$) of compound **5a**.

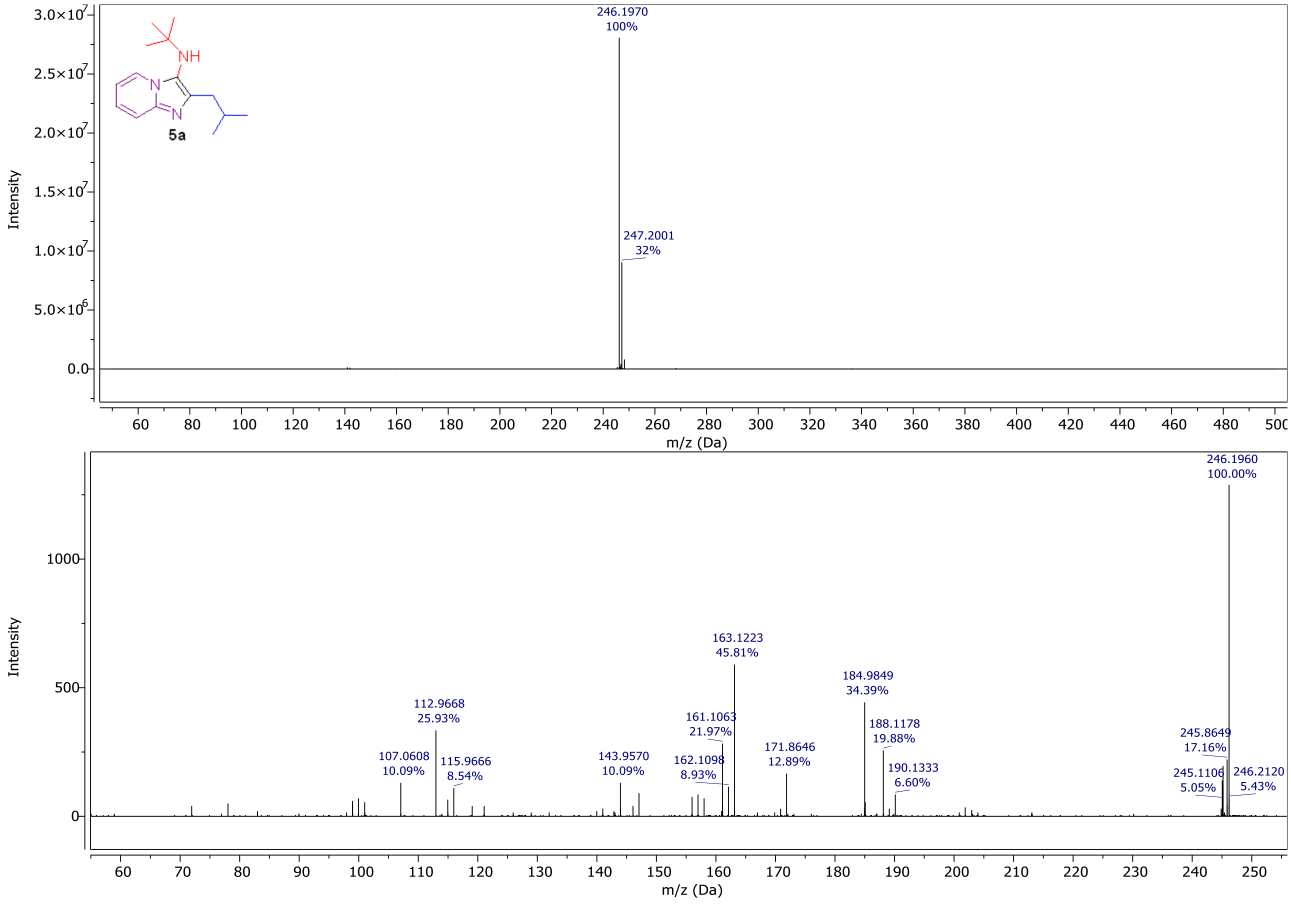

S 216. HRMS (ESI-QTOF) of compound **5a** and HRMS/MS for $[M+H]^+$.

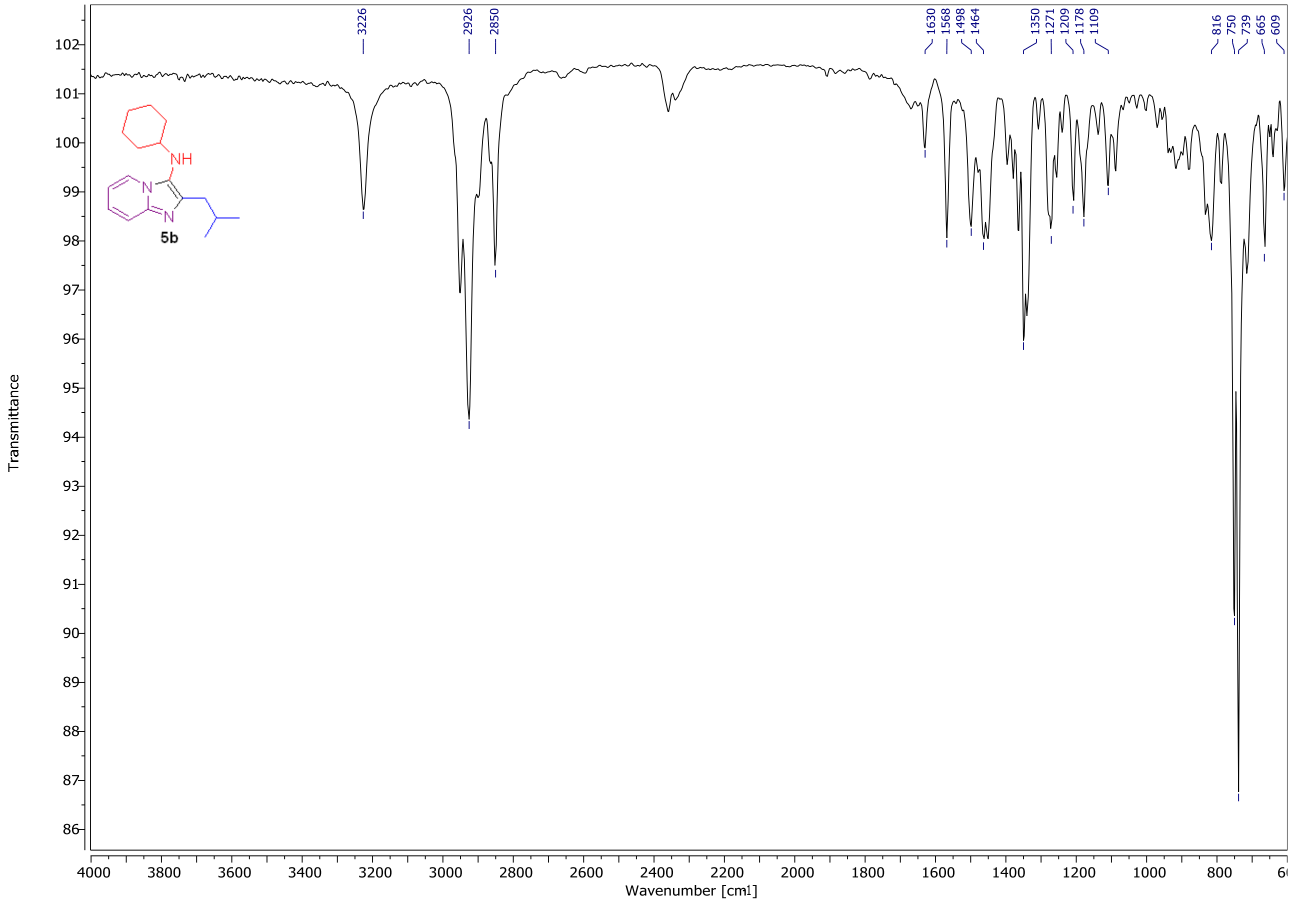

S 217. FT-IR (ATR) of compound **5b**.

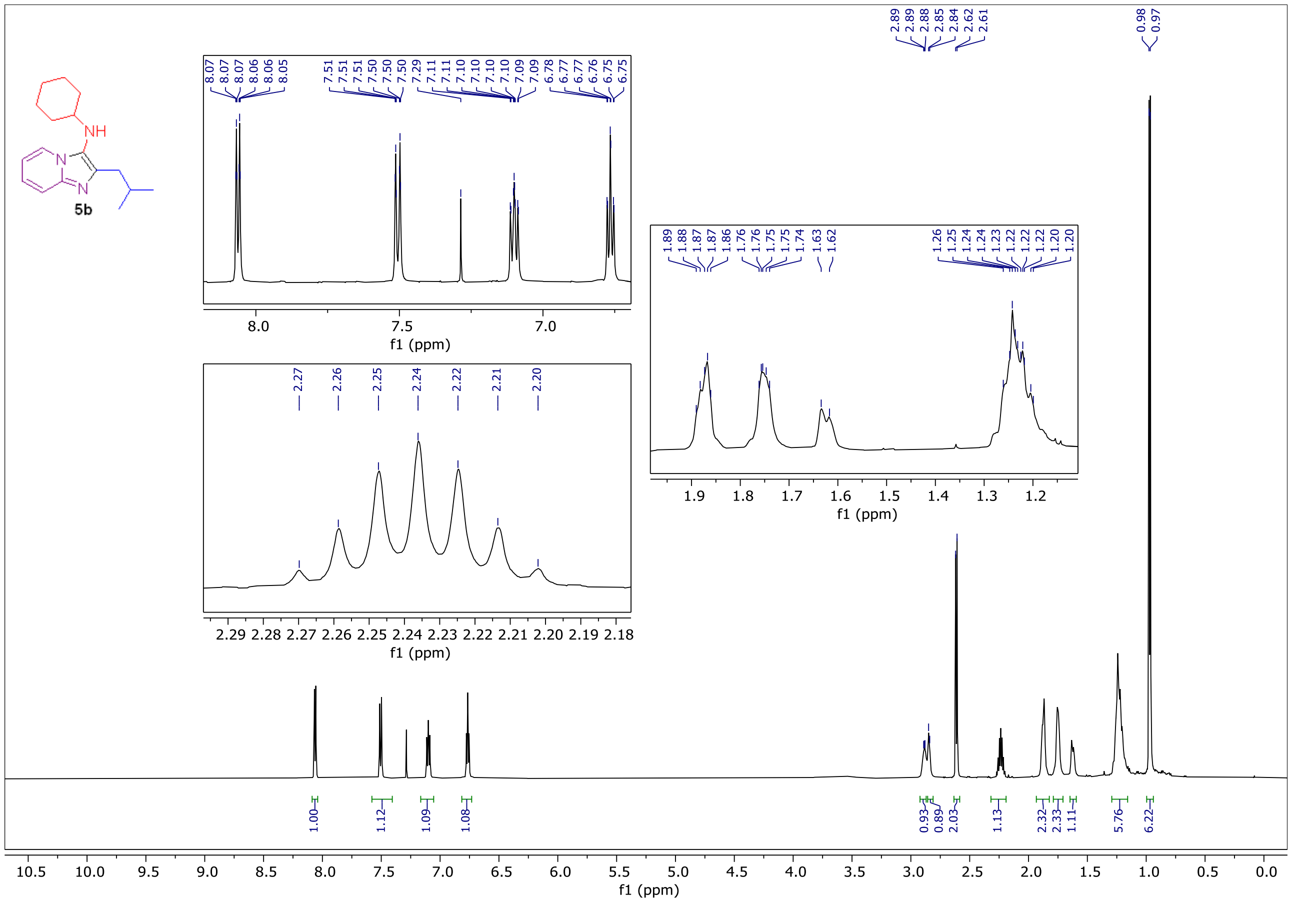

S 218. ^{1}H NMR spectrum (600 MHz, $CDCl_3$) of compound **5b**.

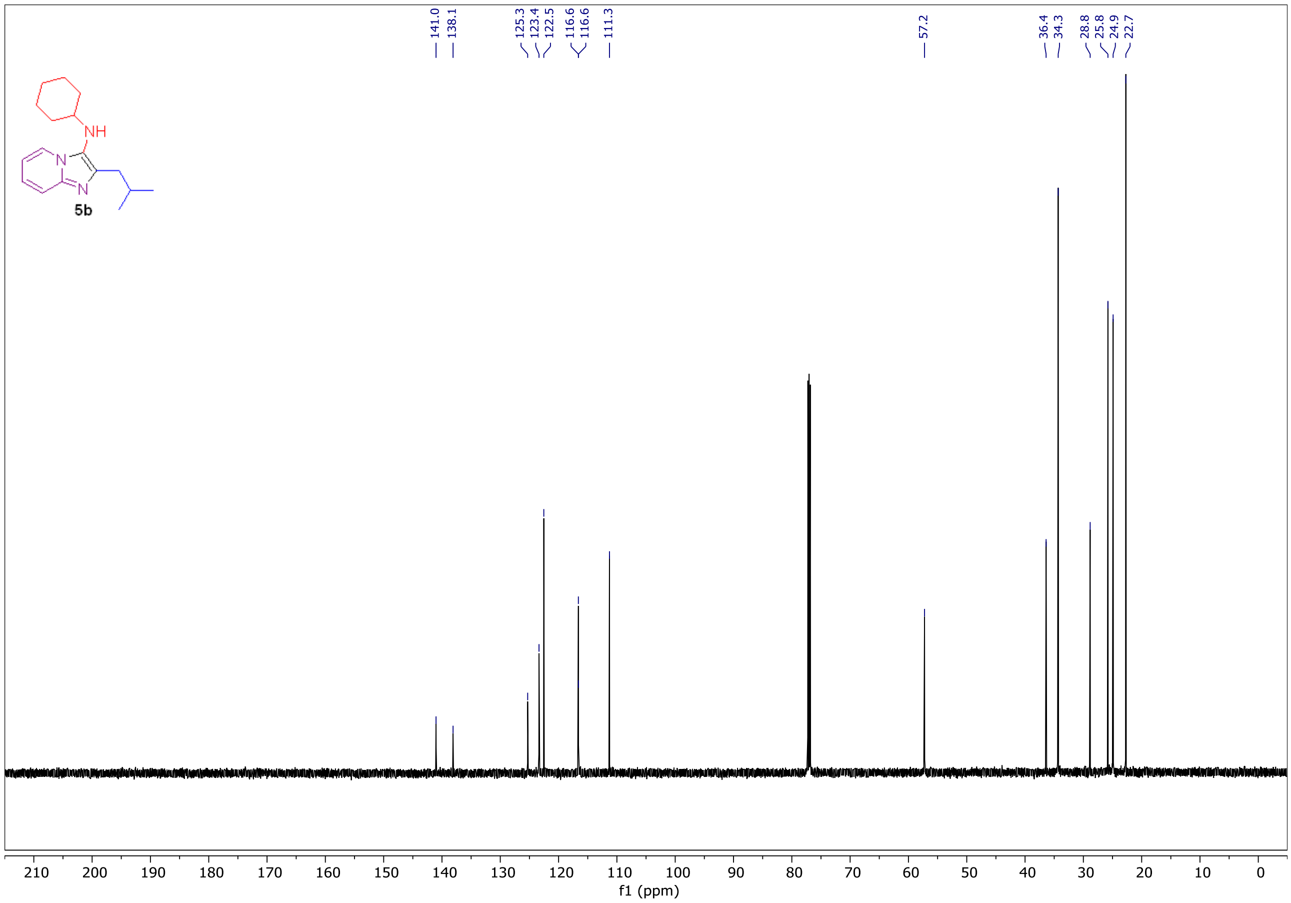

S 219. ^{13}C NMR spectrum (151 MHz, $CDCl_3$) of compound **5b**.

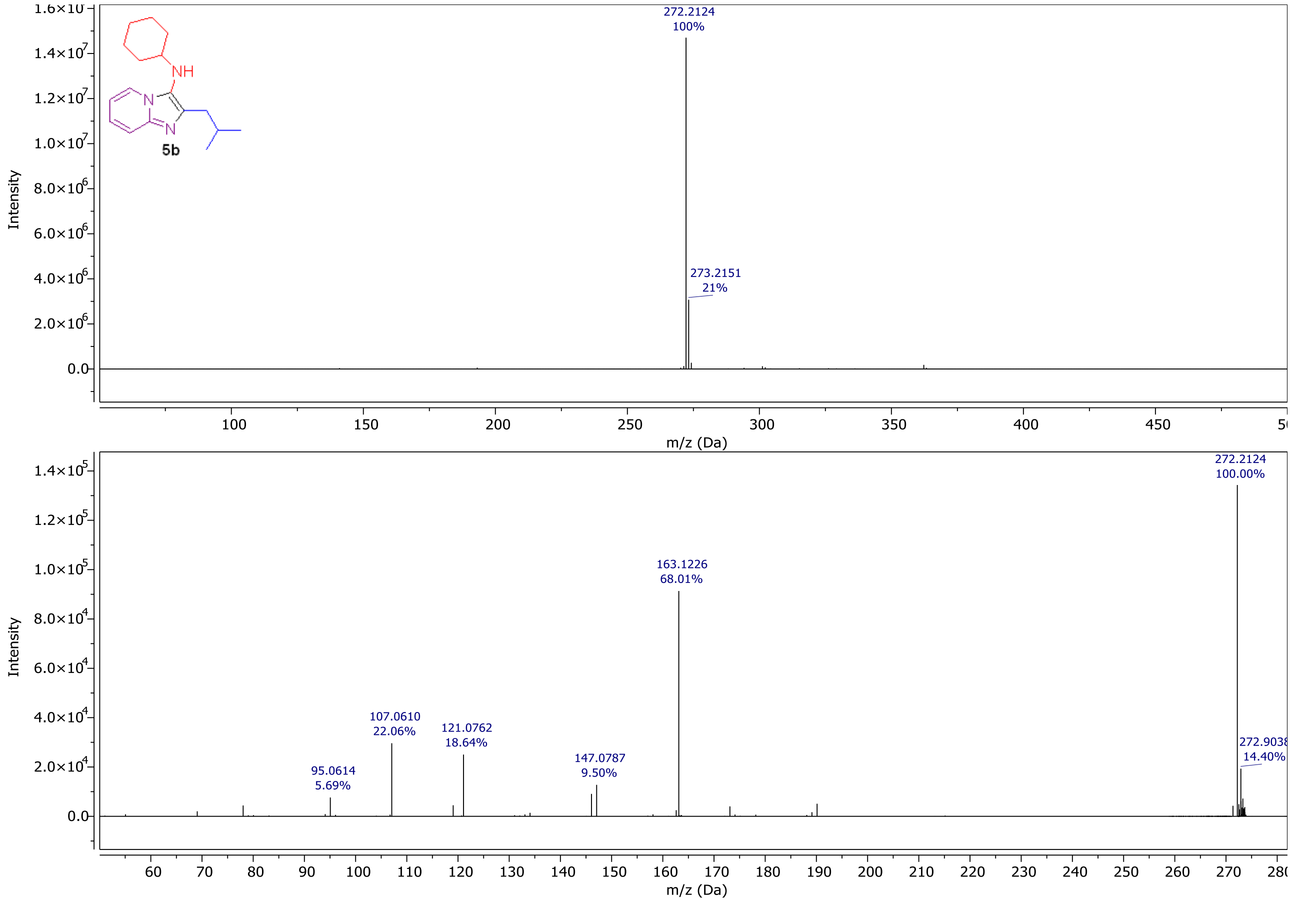

S 220. HRMS (ESI-QTOF) of compound **5b** and HRMS/MS for $[M+H]^+$.

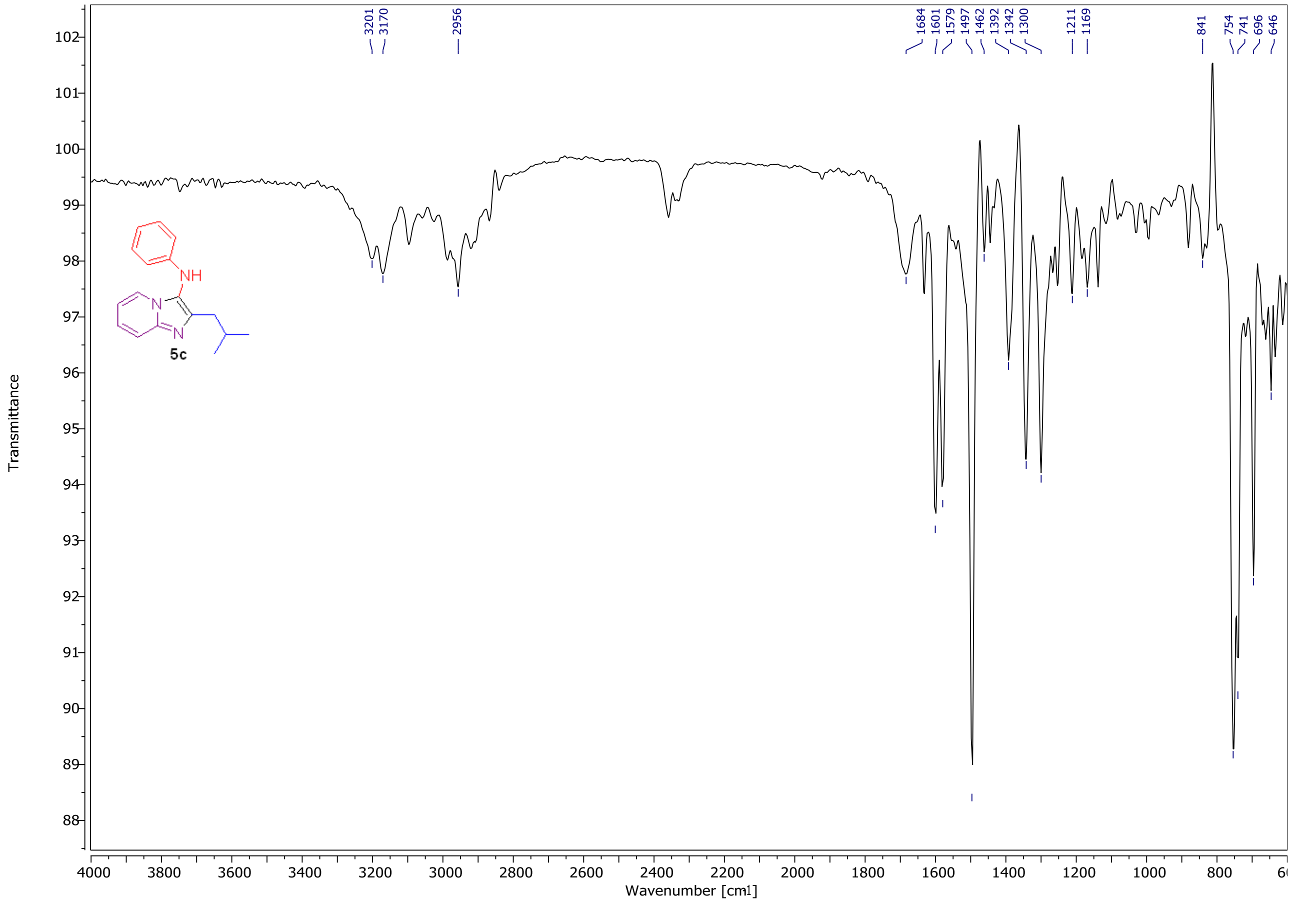

S 221. FT-IR (ATR) of compound **5c**.

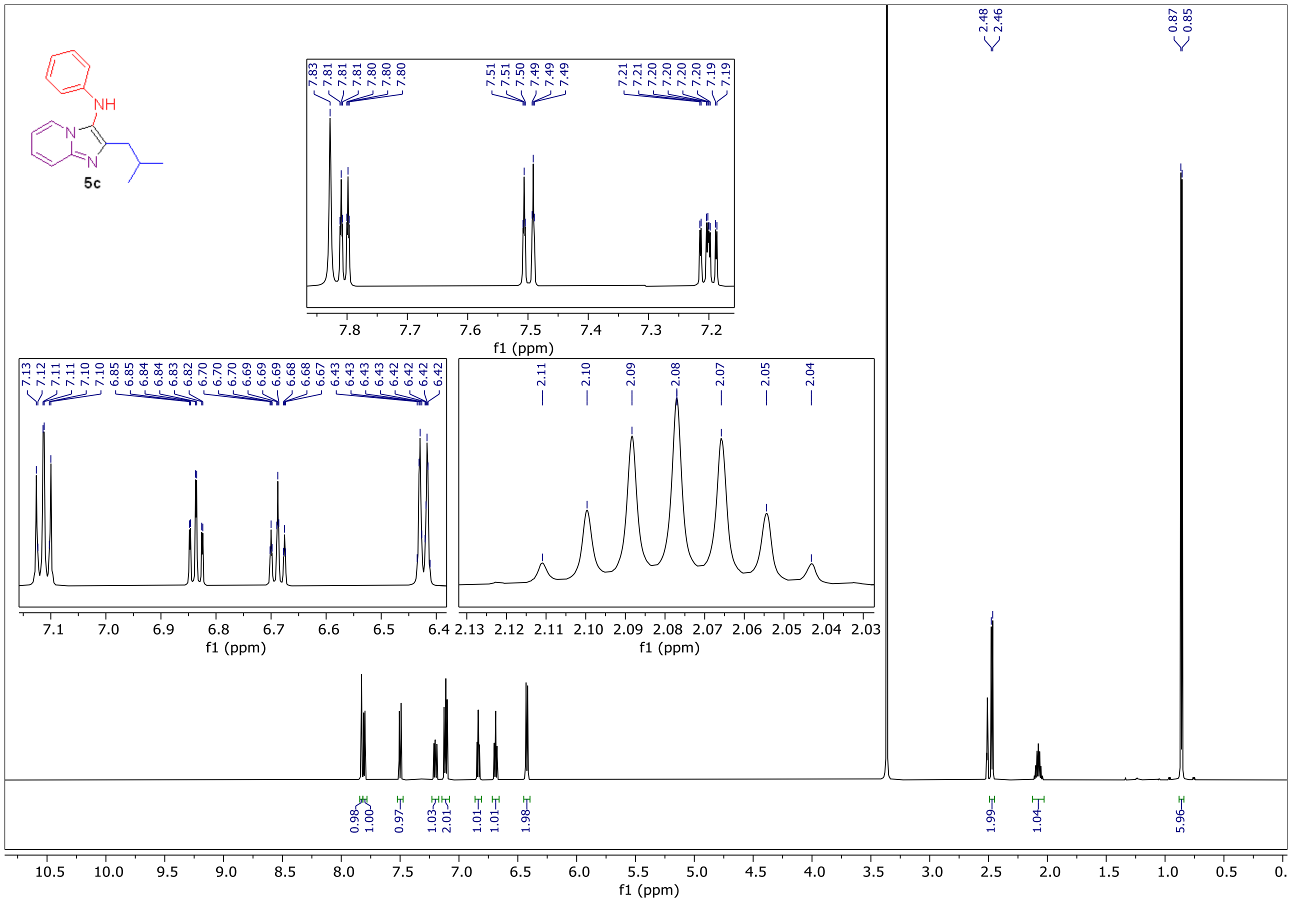

S 222. ^{1}H NMR spectrum (600 MHz, DMSO-d_6) of compound **5c**.

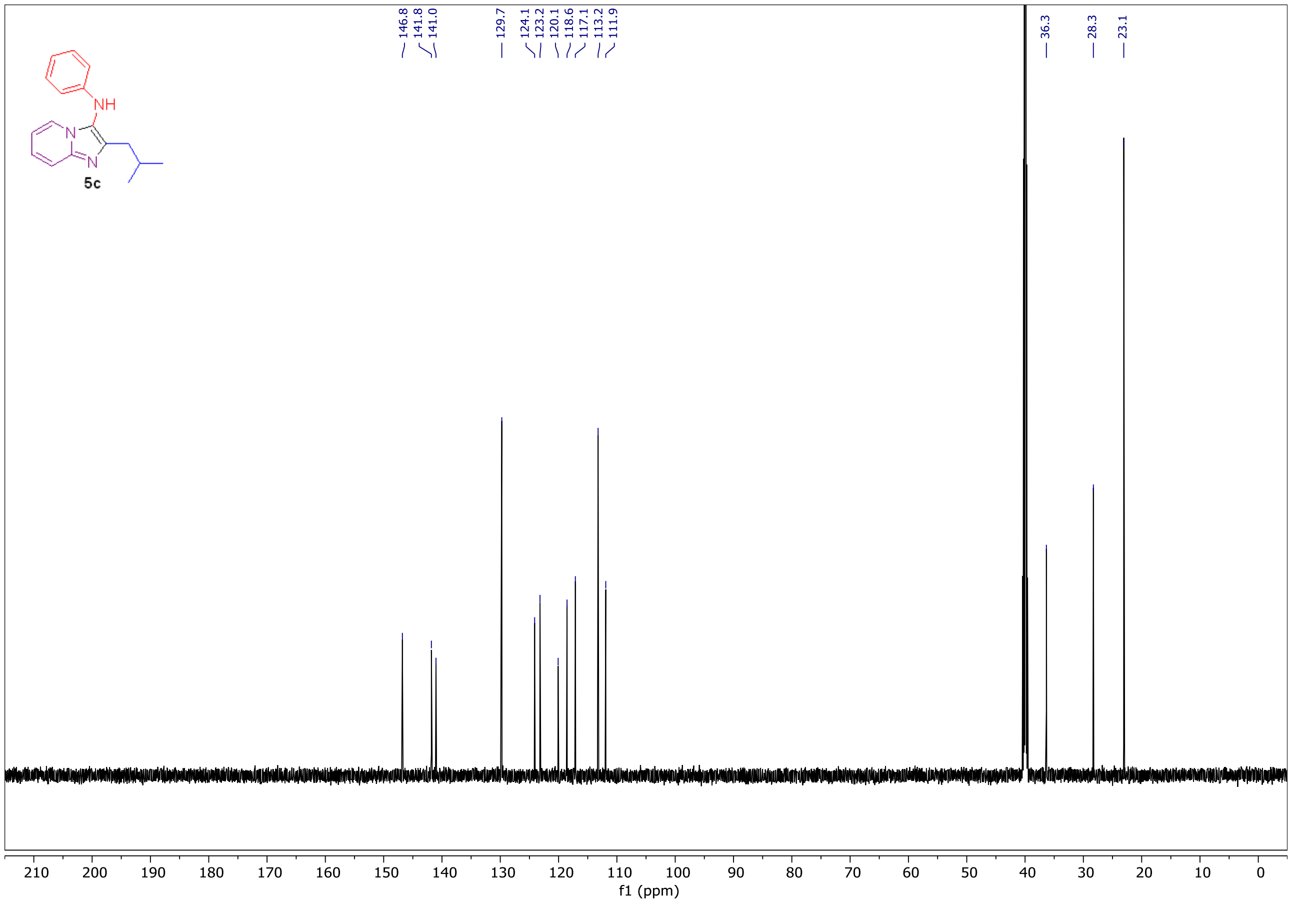

S 223. ^{13}C NMR spectrum (151 MHz, DMSO-d_6) of compound **5c**.

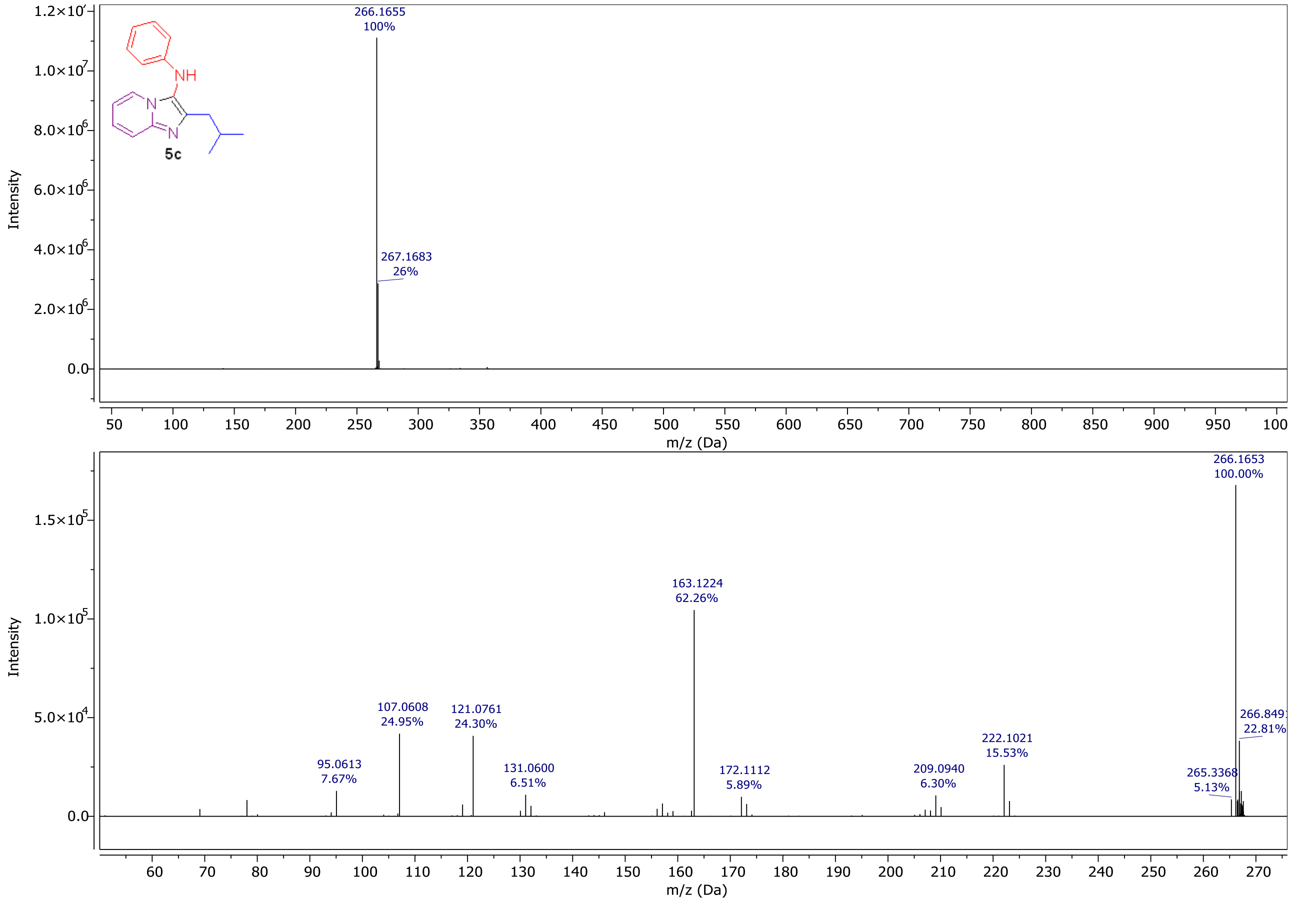

S 224. HRMS (ESI-QTOF) of compound **5c** and HRMS/MS for $[M+H]^+$.

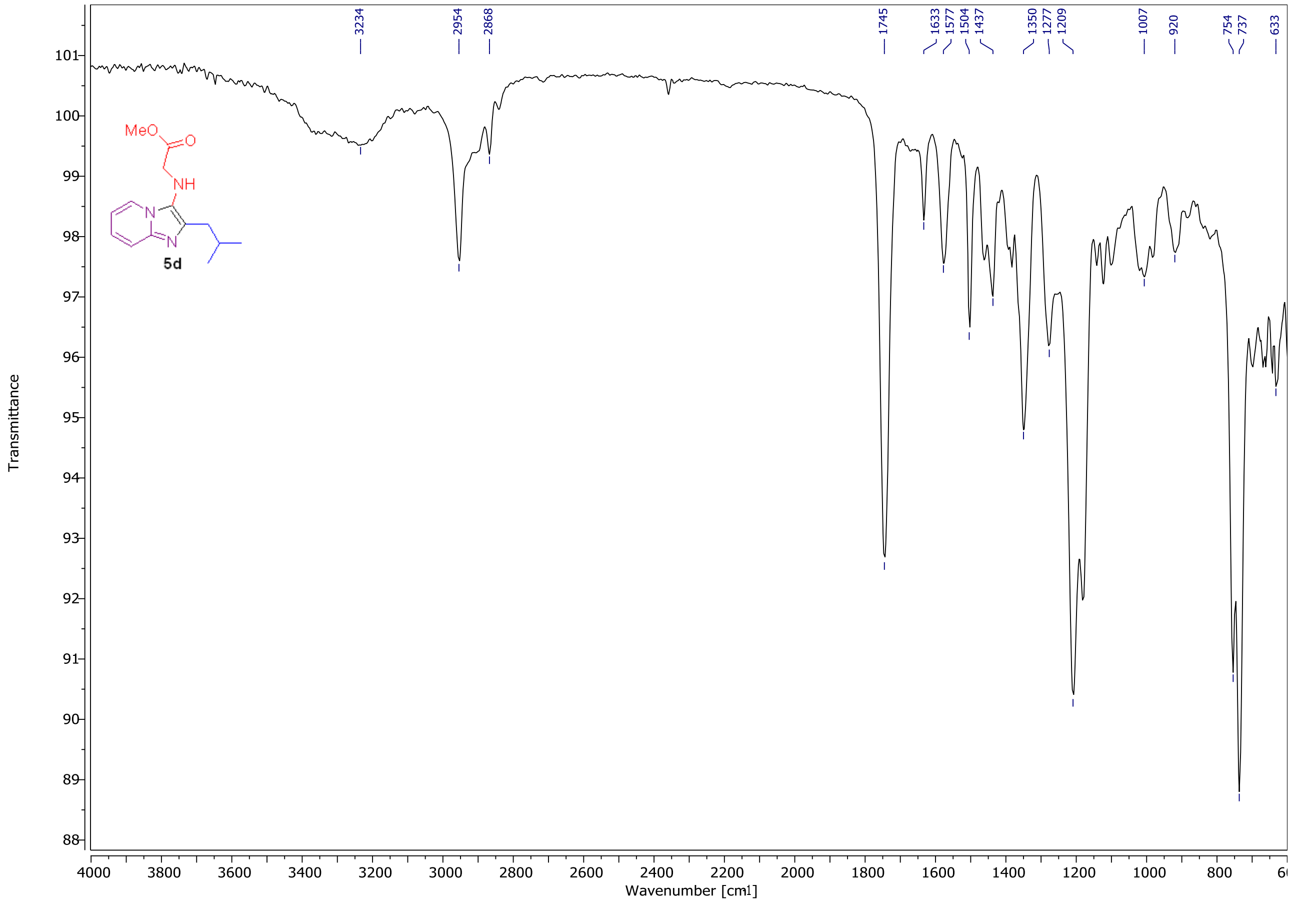

S 225. FT-IR (ATR) of compound **5d**.

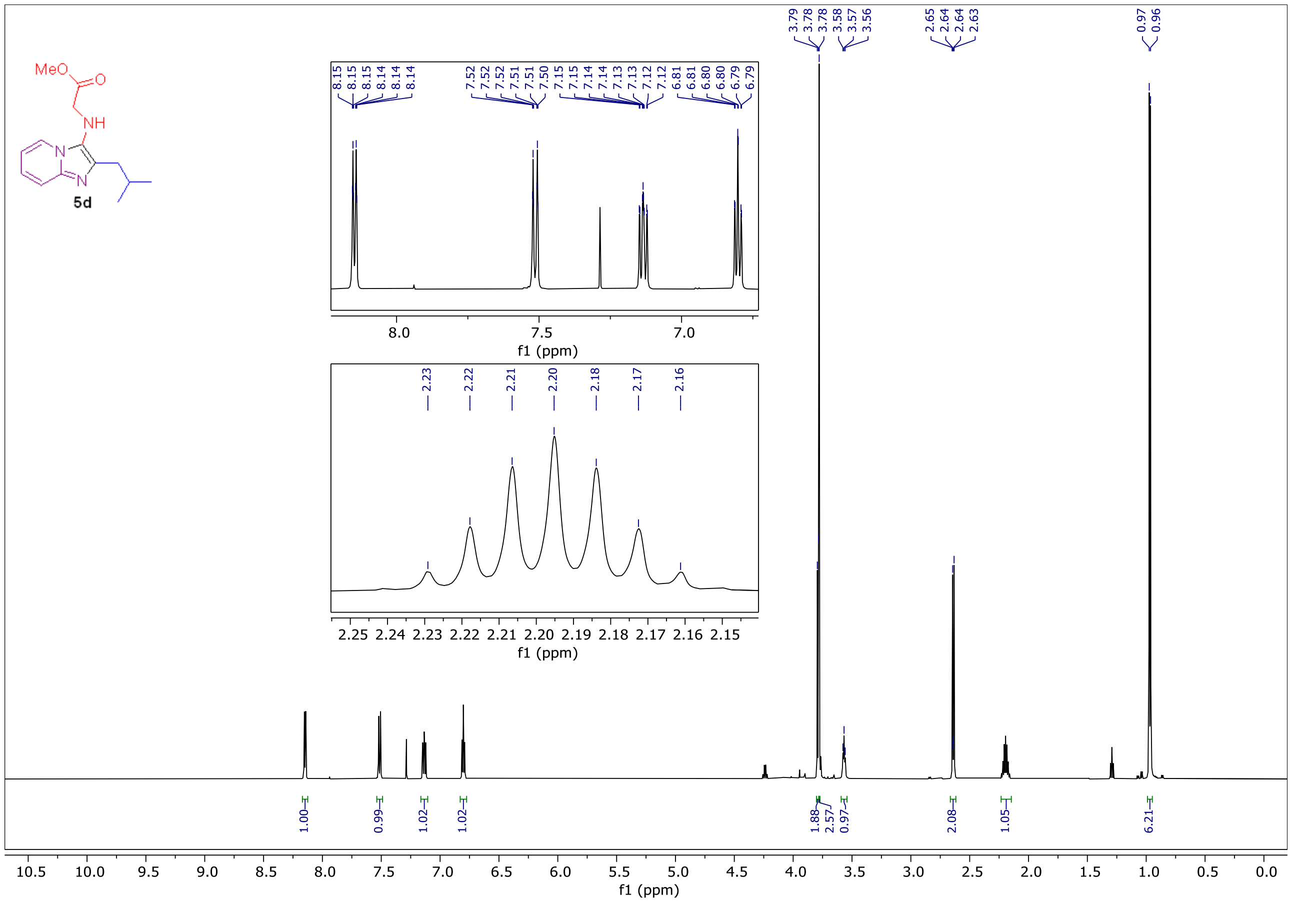

S 226. ^{1}H NMR spectrum (600 MHz, $CDCl_3$) of compound **5d**.

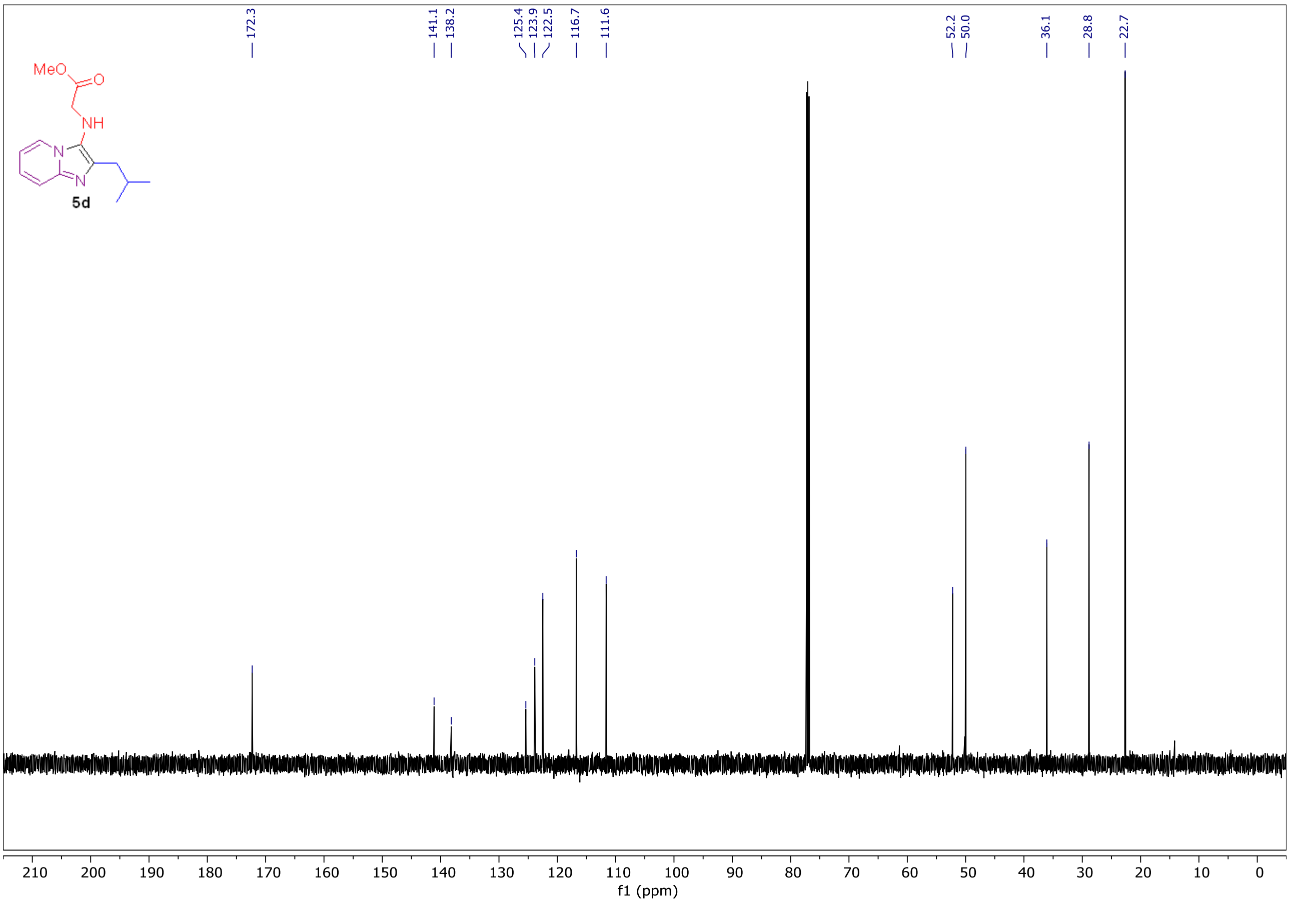

S 227. ^{13}C NMR spectrum (151 MHz, $CDCl_3$) of compound **5d**.

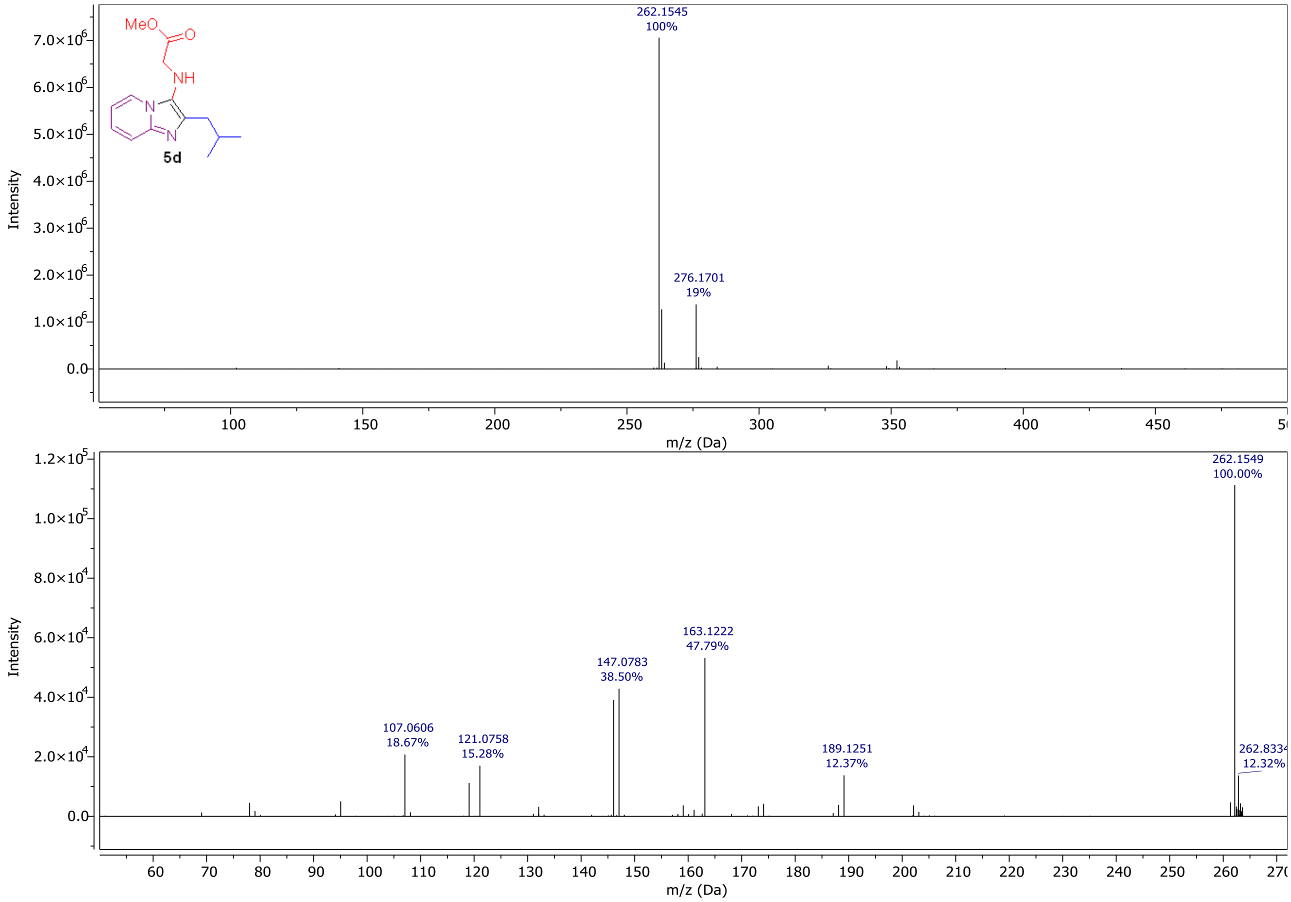

S 228. HRMS (ESI-QTOF) of compound **5d** and HRMS/MS for $[M+H]^+$.

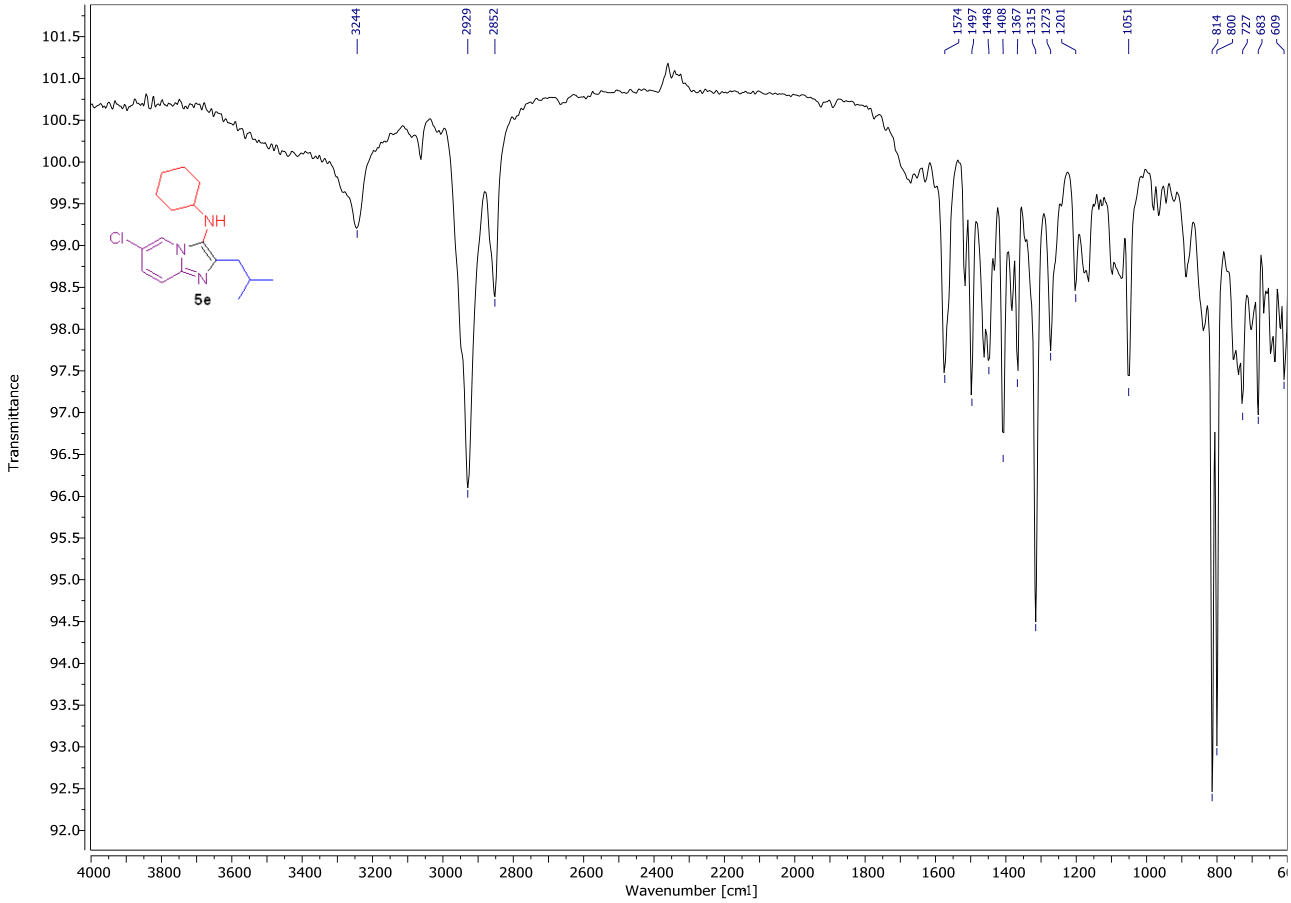

S 229. FT-IR (ATR) of compound **5e**.

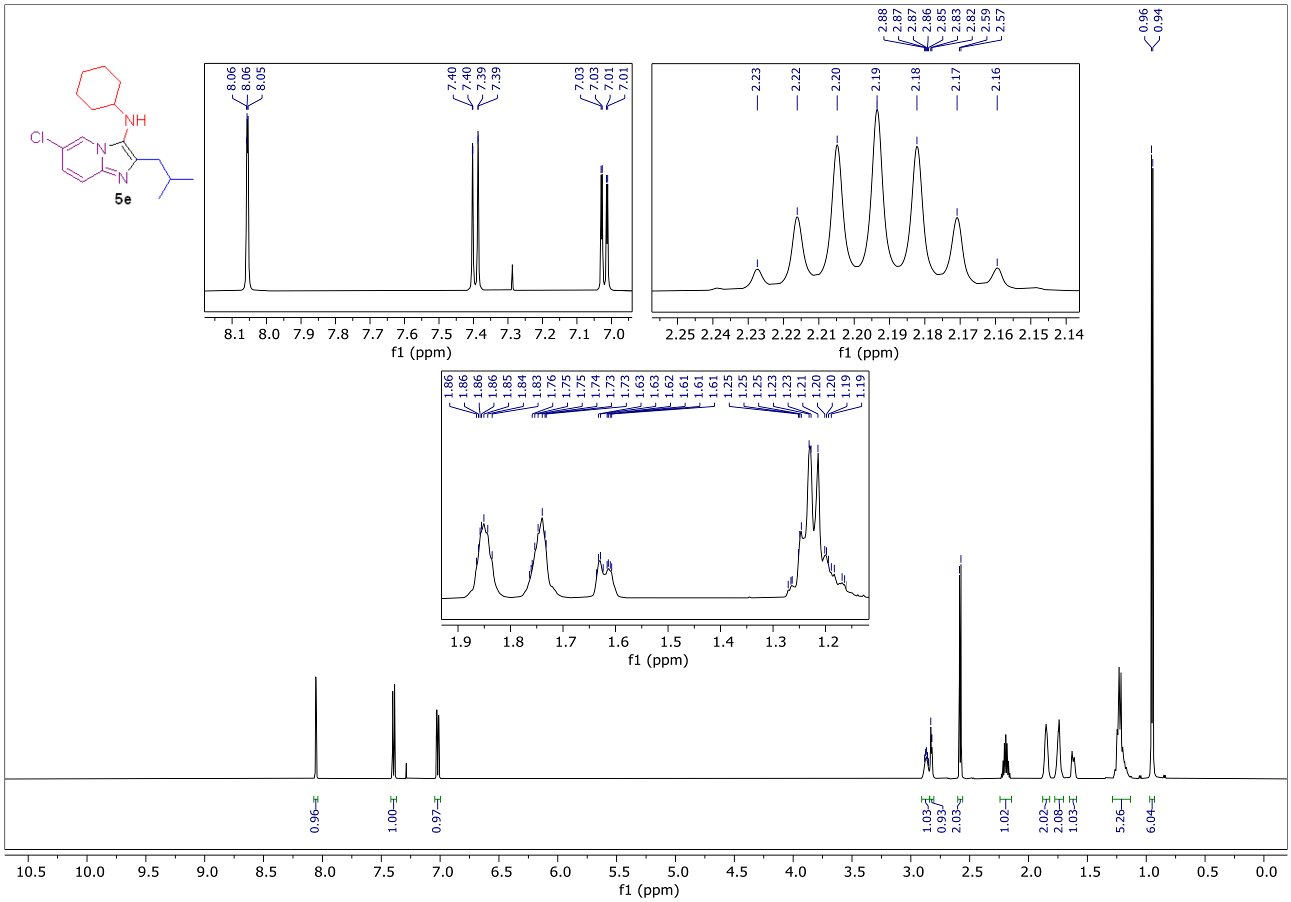

S 230. ^{1}H NMR spectrum (600 MHz, $CDCl_3$) of compound **5e**.

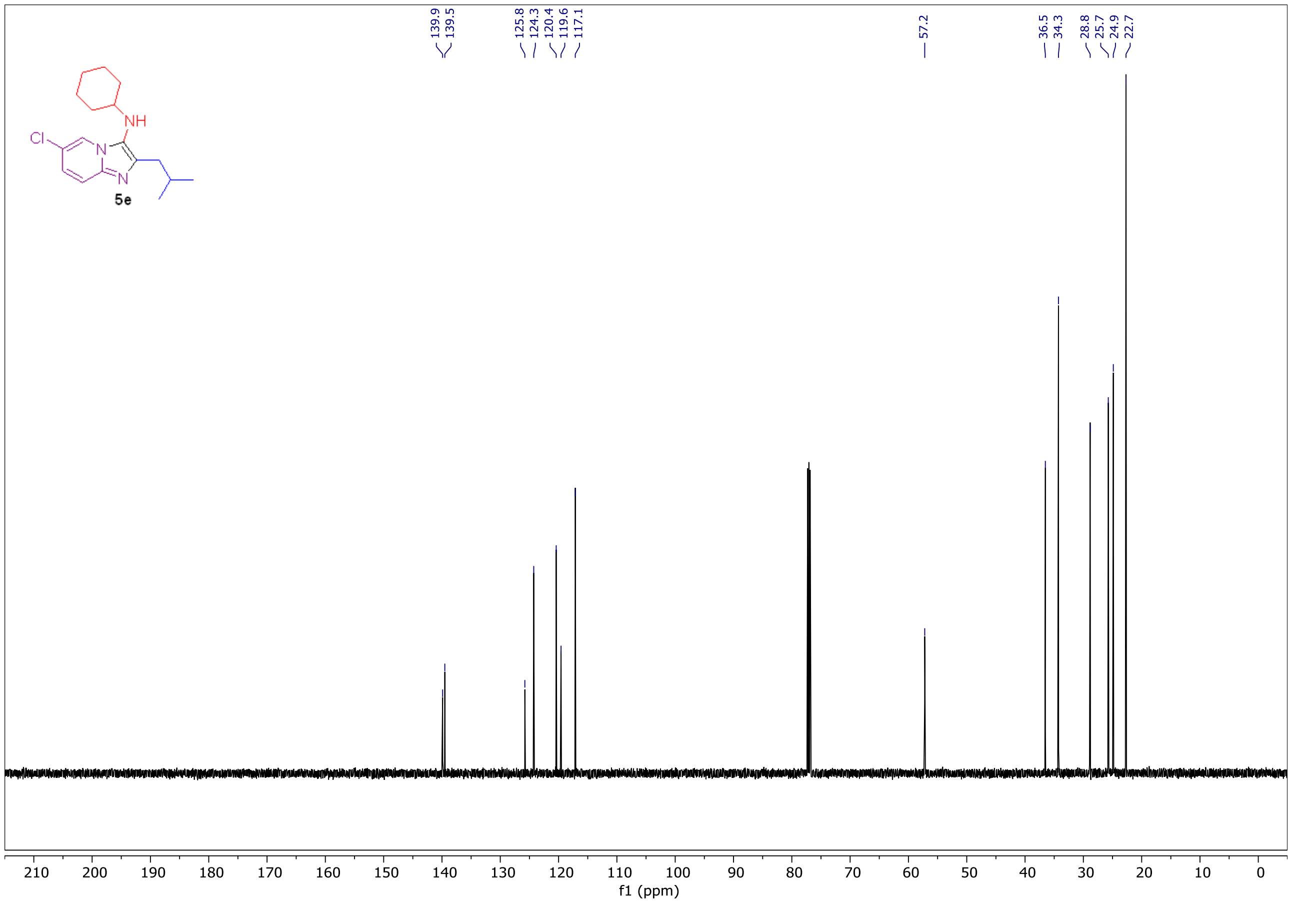

S 231. ^{13}C NMR spectrum (151 MHz, $CDCl_3$) of compound **5e**.

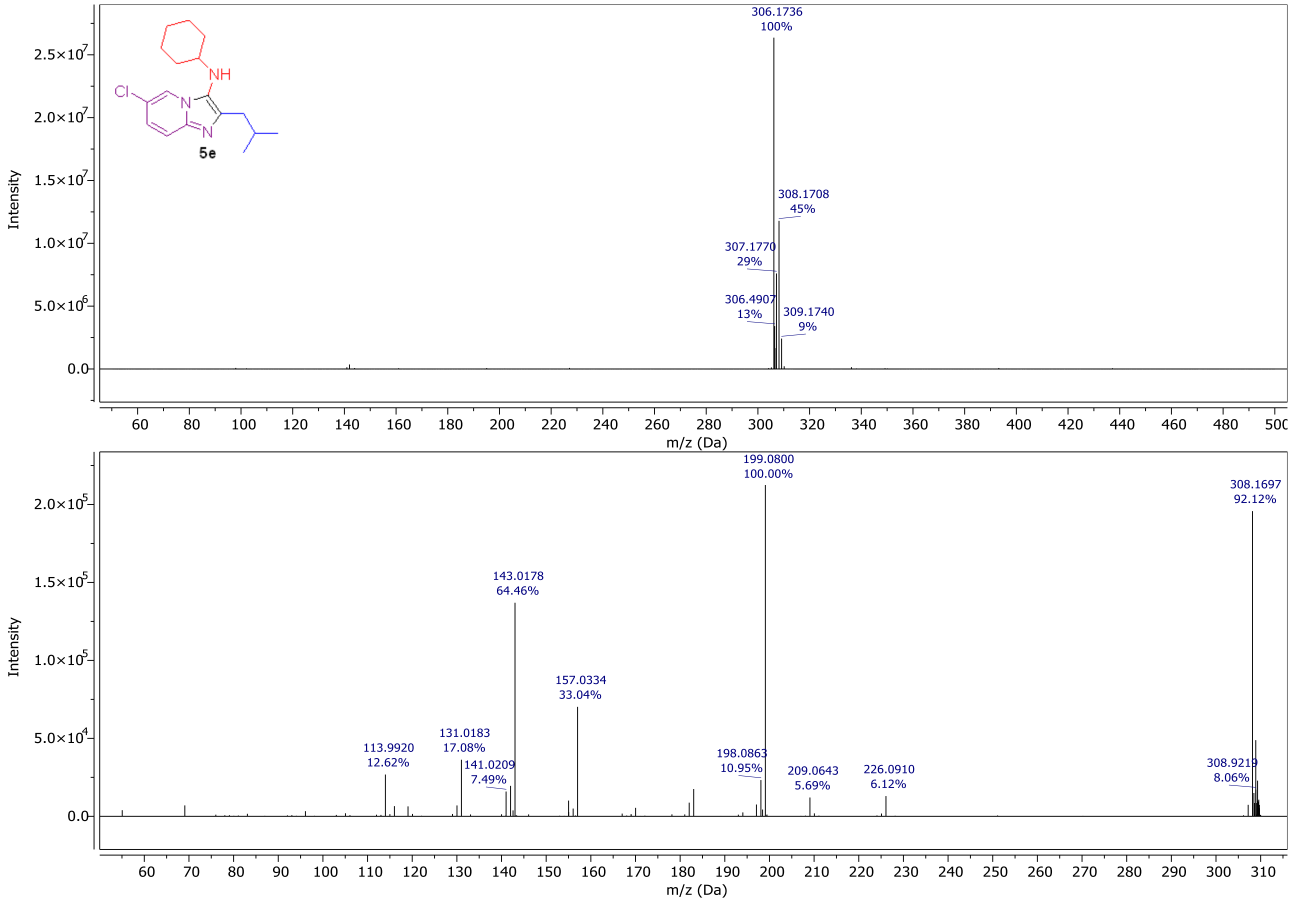

S 232. HRMS (ESI-QTOF) of compound **5e** and HRMS/MS for [M+H]$^+$.

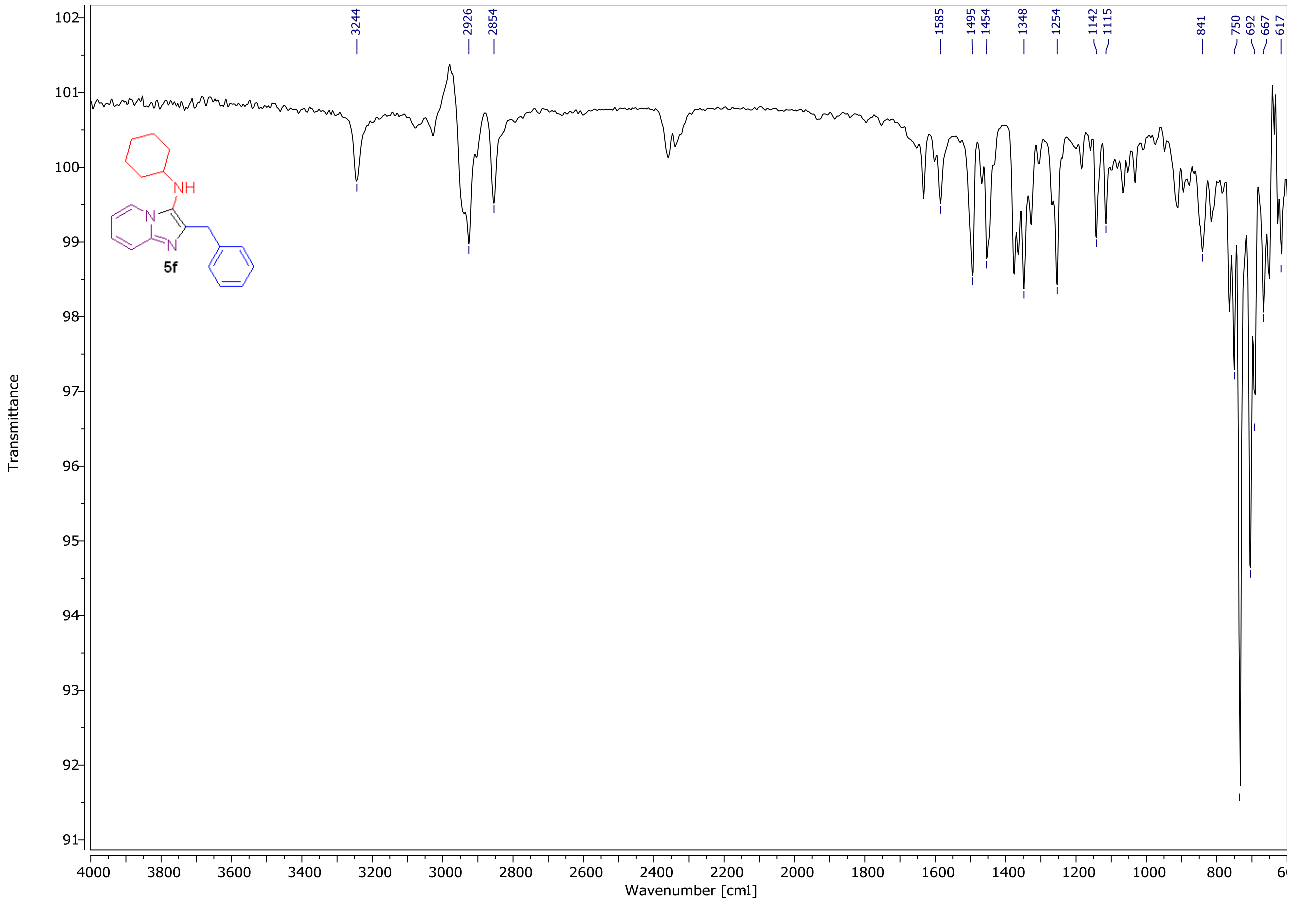

S 233. FT-IR (ATR) of compound **5f**.

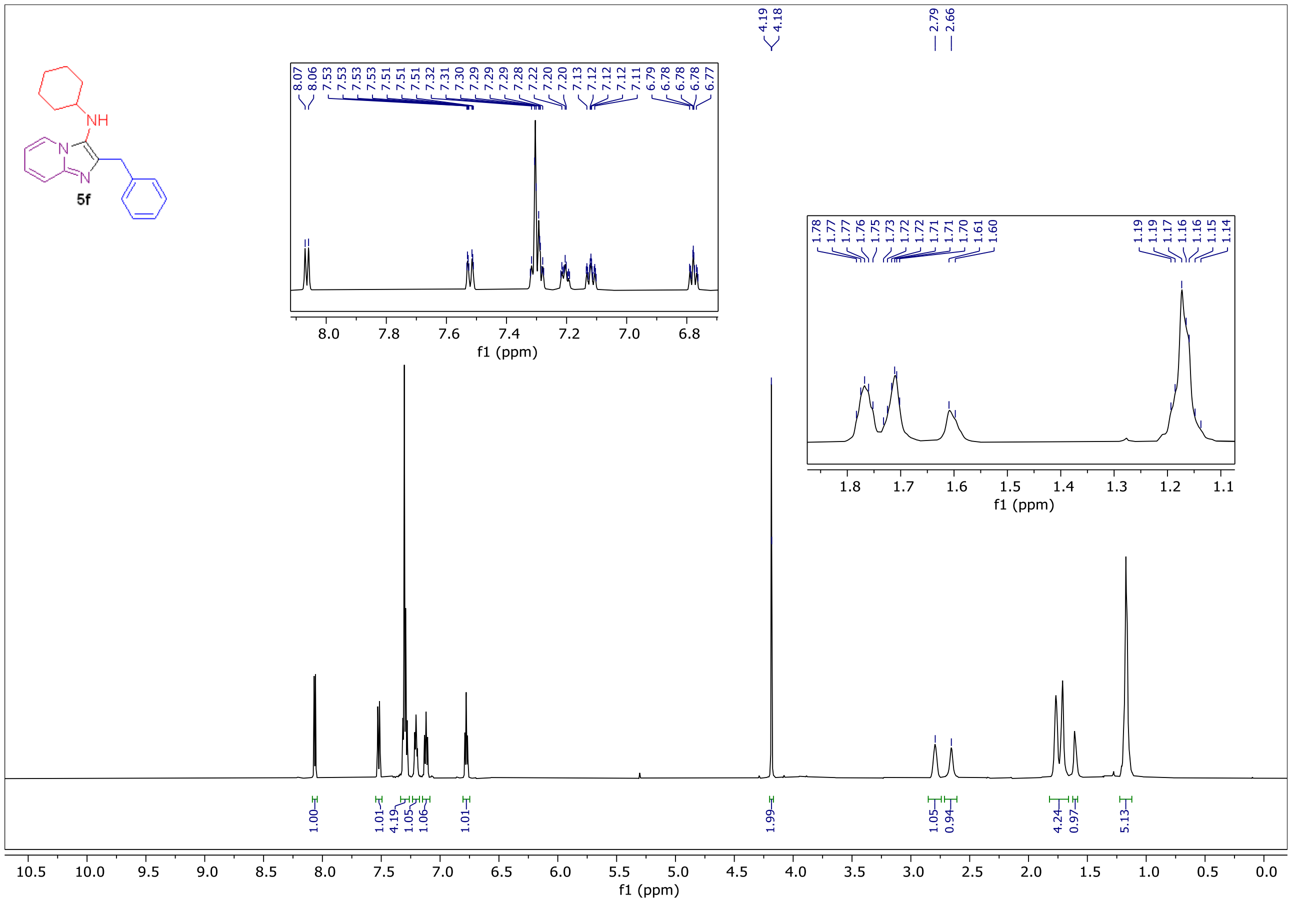

S 234. ^{1}H NMR spectrum (600 MHz, $CDCl_3$) of compound **5f**.

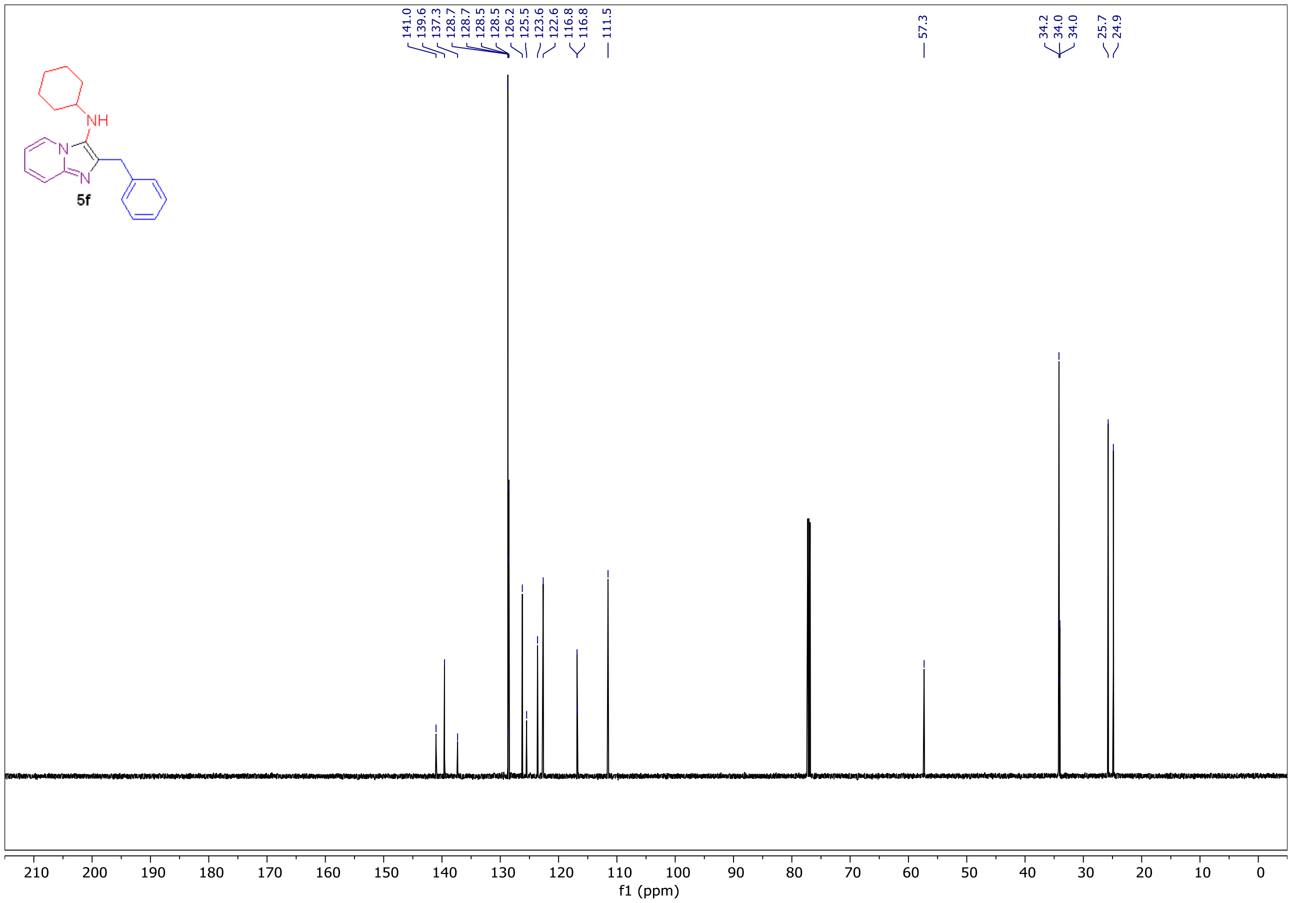

S 235. ^{13}C NMR spectrum (151 MHz, $CDCl_3$) of compound **5f**.

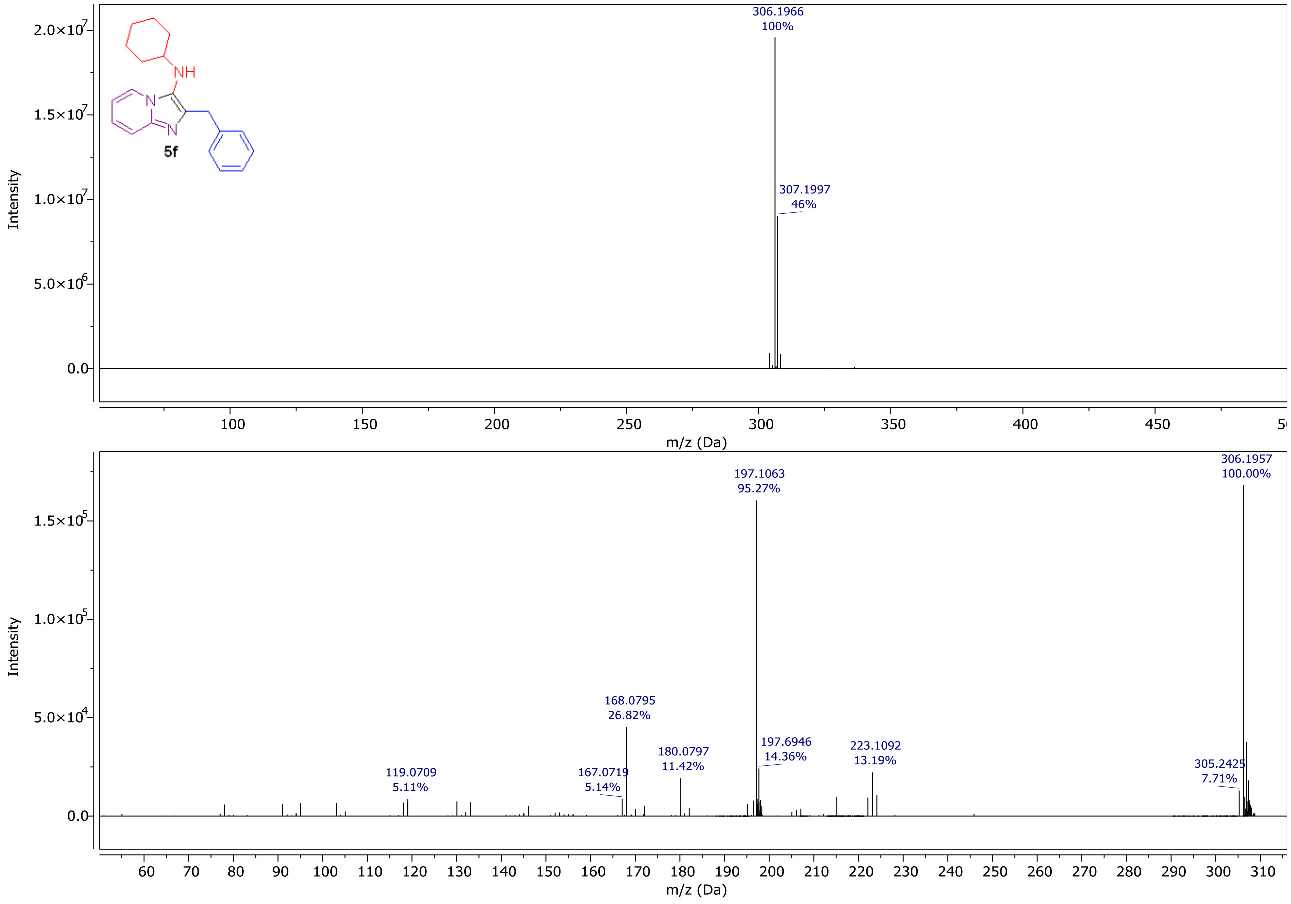

S 236. HRMS (ESI-QTOF) of compound **5f** and HRMS/MS for $[M+H]^+$.

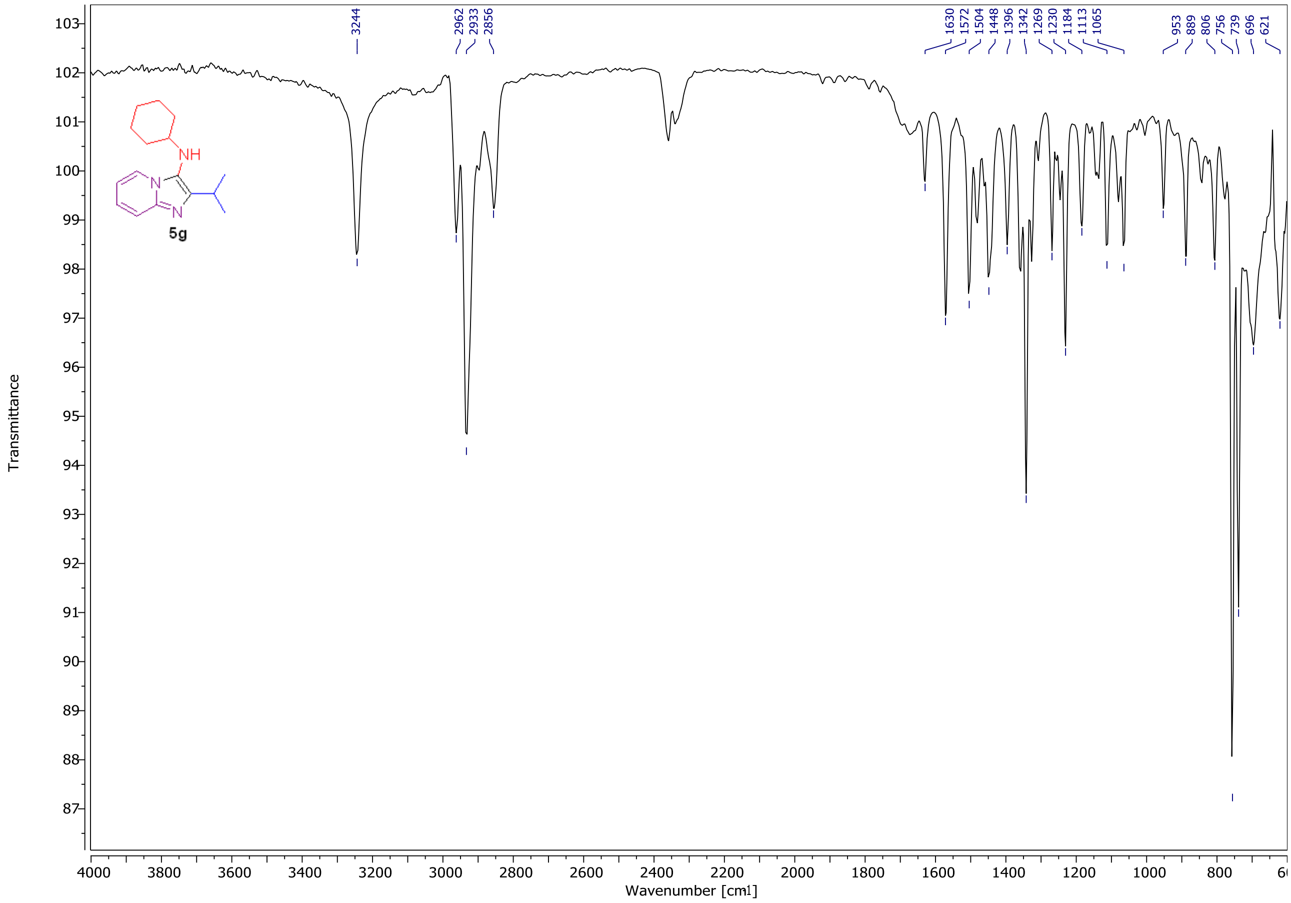

S 237. FT-IR (ATR) of compound **5g**.

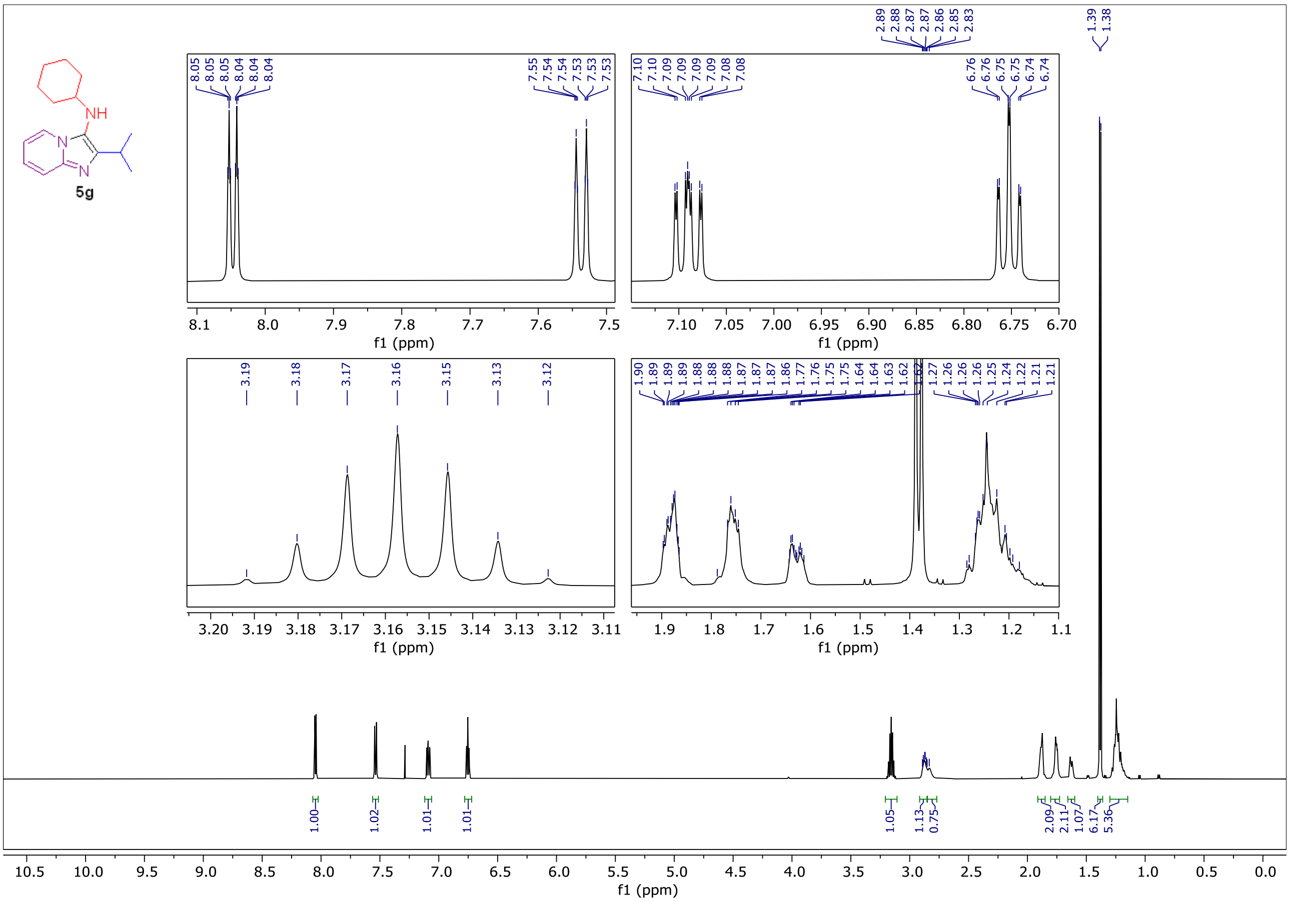

S 238. ^{1}H NMR spectrum (600 MHz, $CDCl_3$) of compound **5g**.

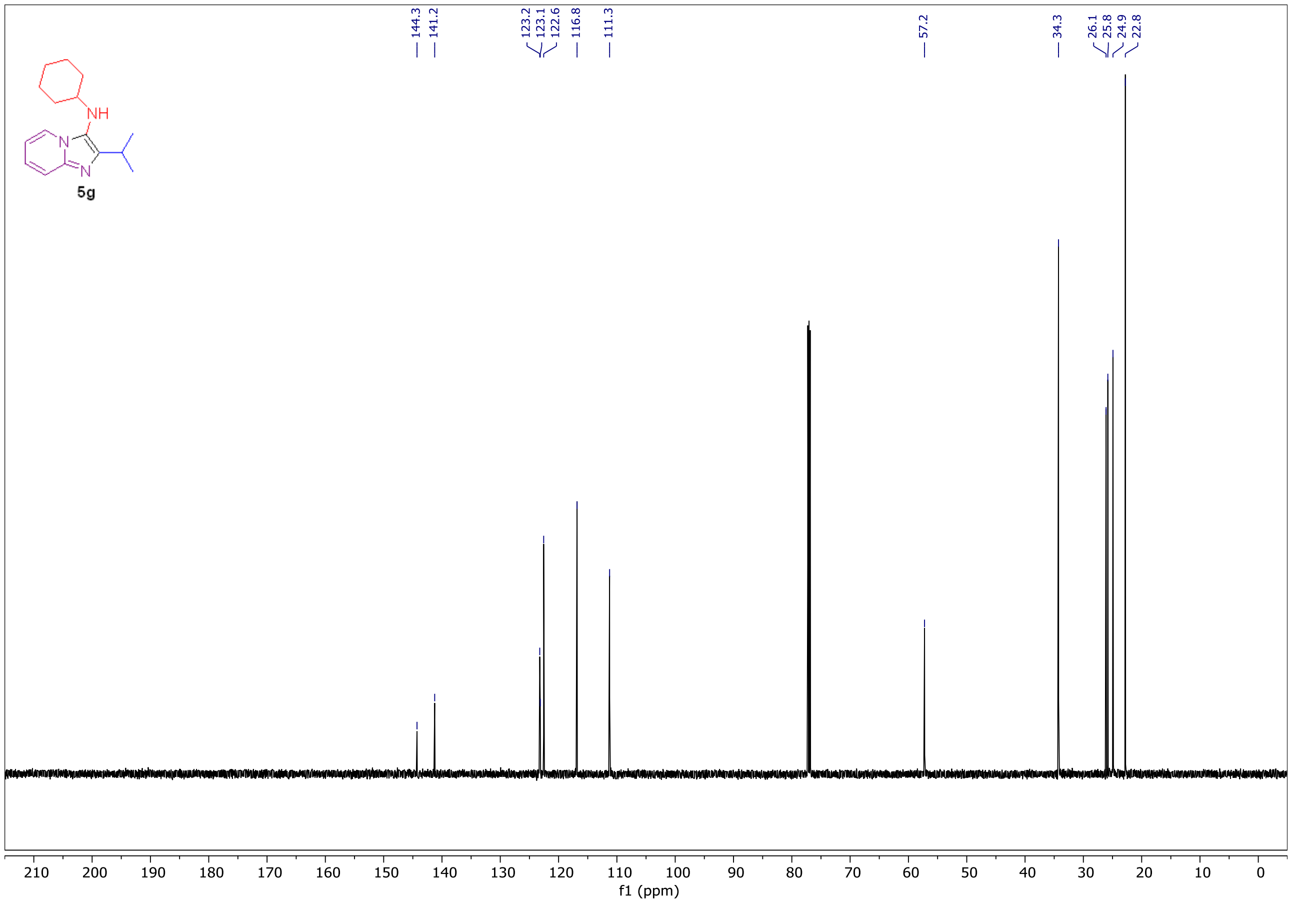

S 239. ^{13}C NMR spectrum (151 MHz, $CDCl_3$) of compound **5g**.

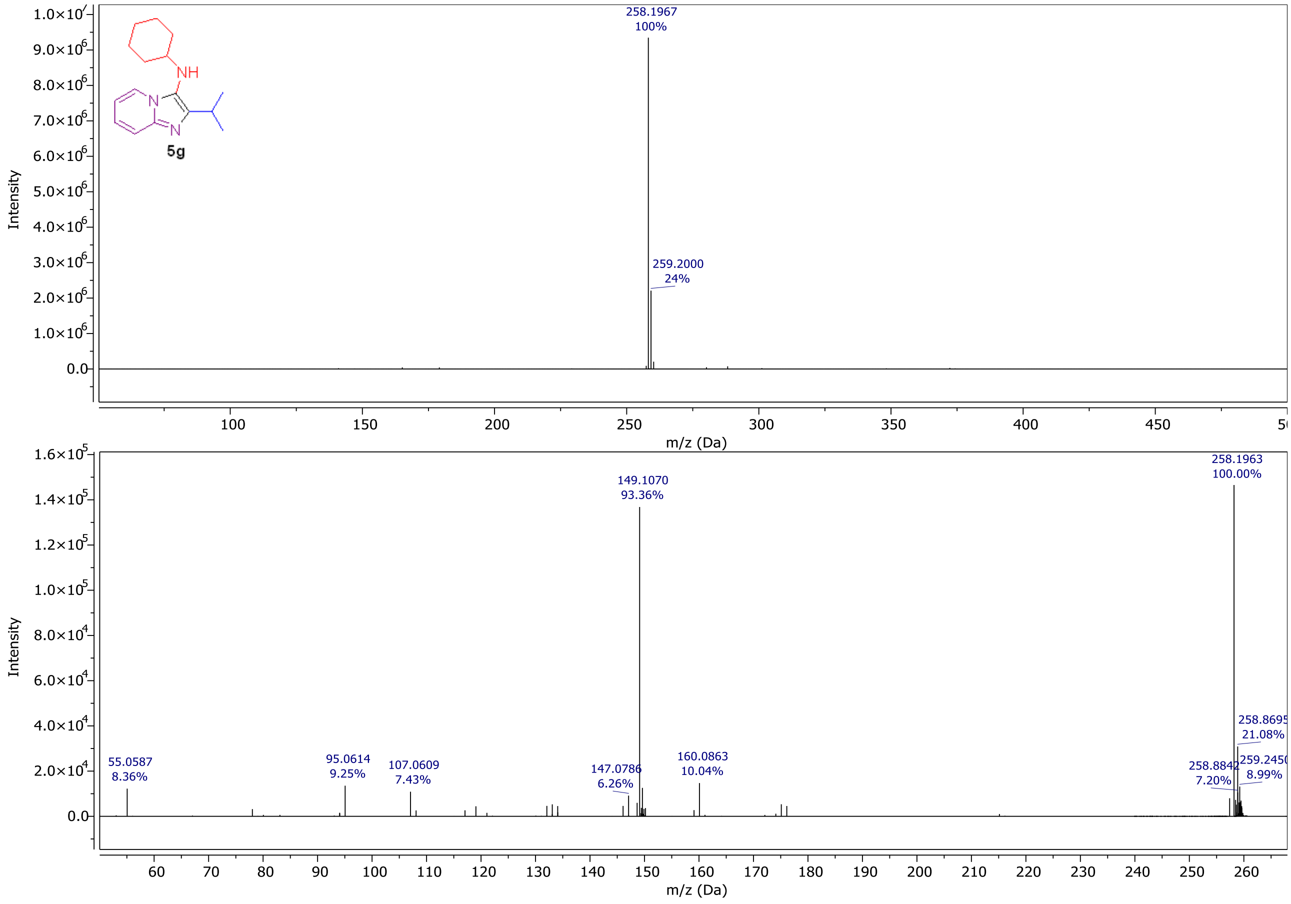

S 240. HRMS (ESI-QTOF) of compound **5g** and HRMS/MS for $[M+H]^+$.

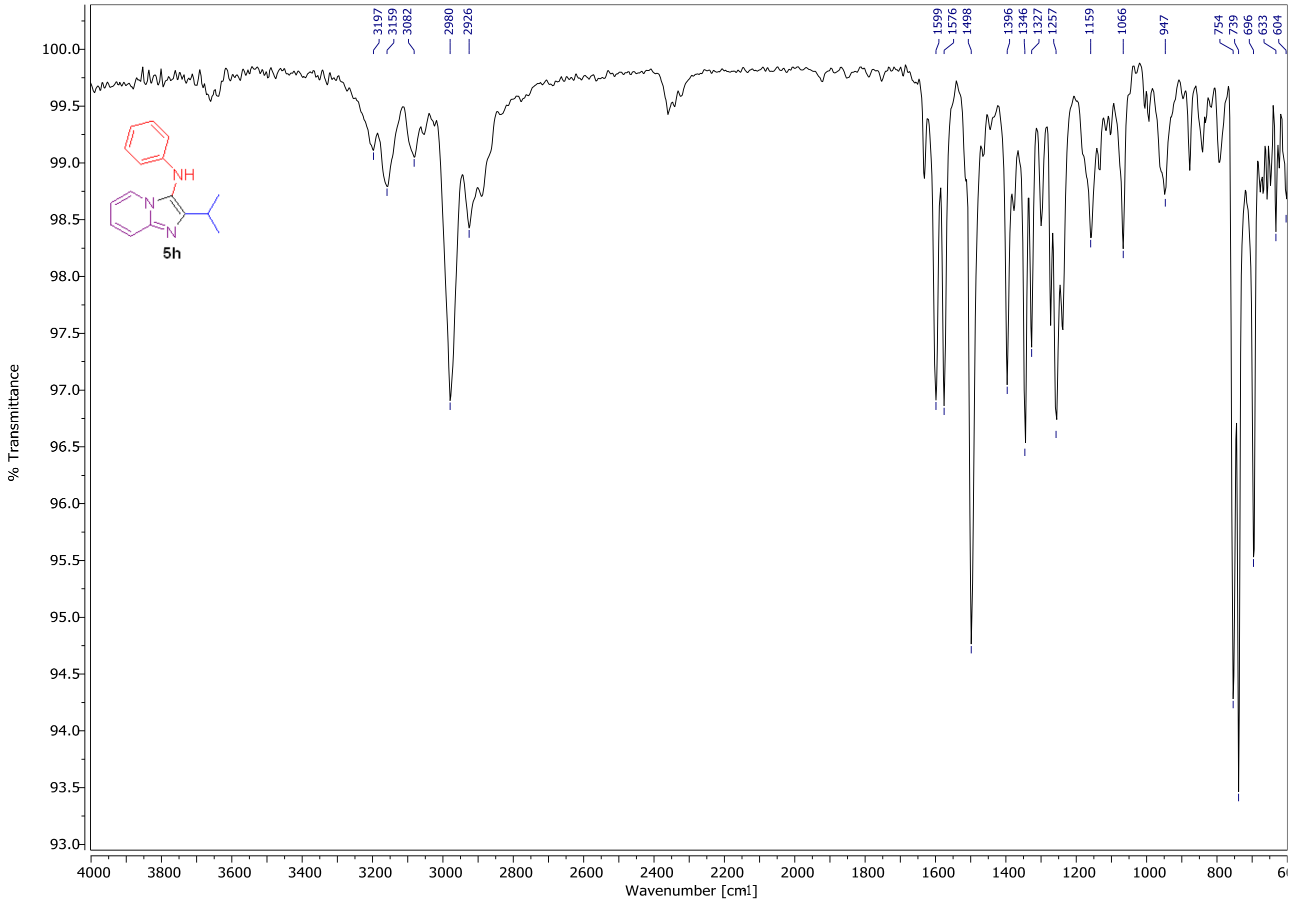

S 241. FT-IR (ATR) of compound **5h**.

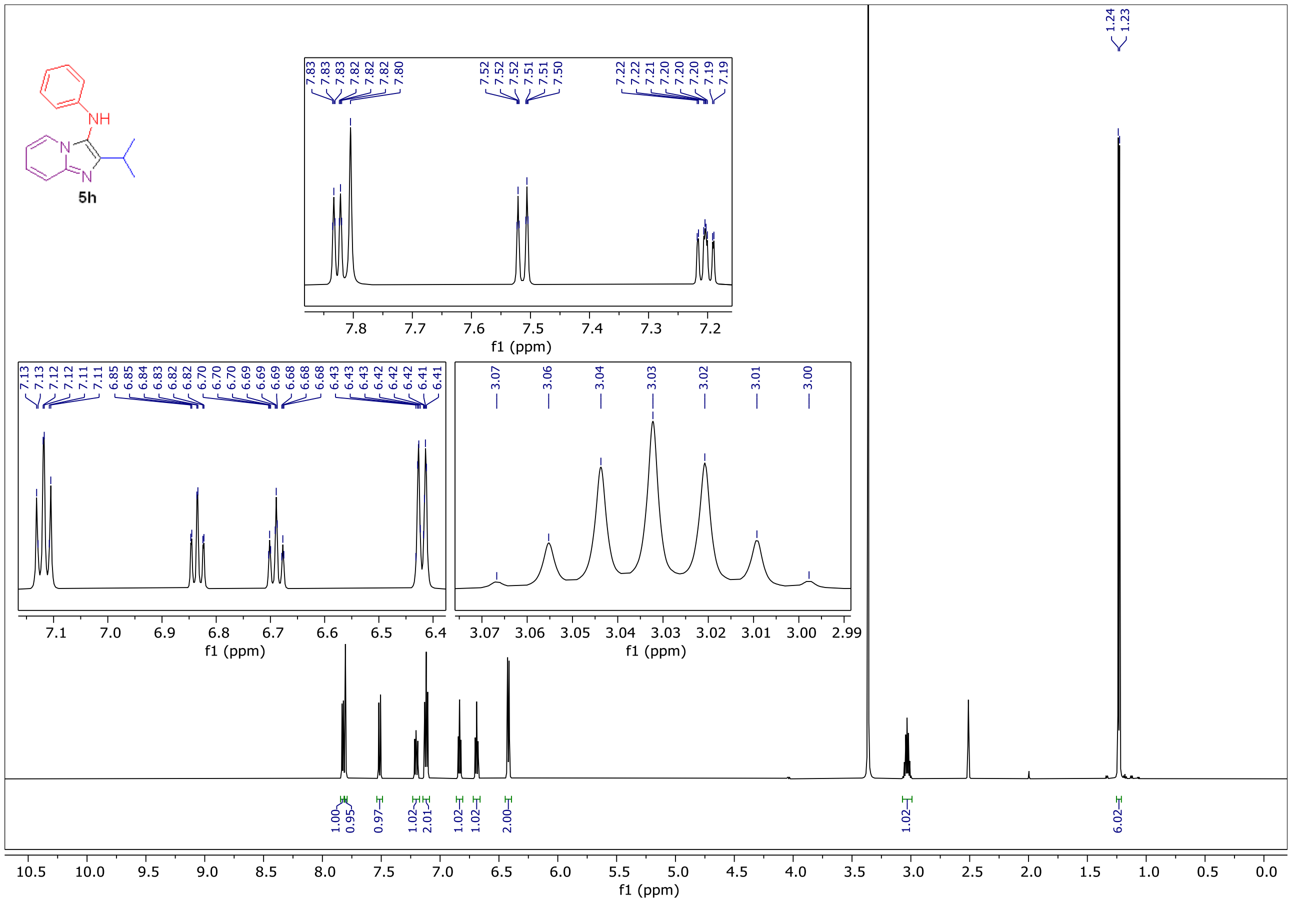

S 242. ^{1}H NMR spectrum (600 MHz, DMSO-d_6) of compound **5h**.

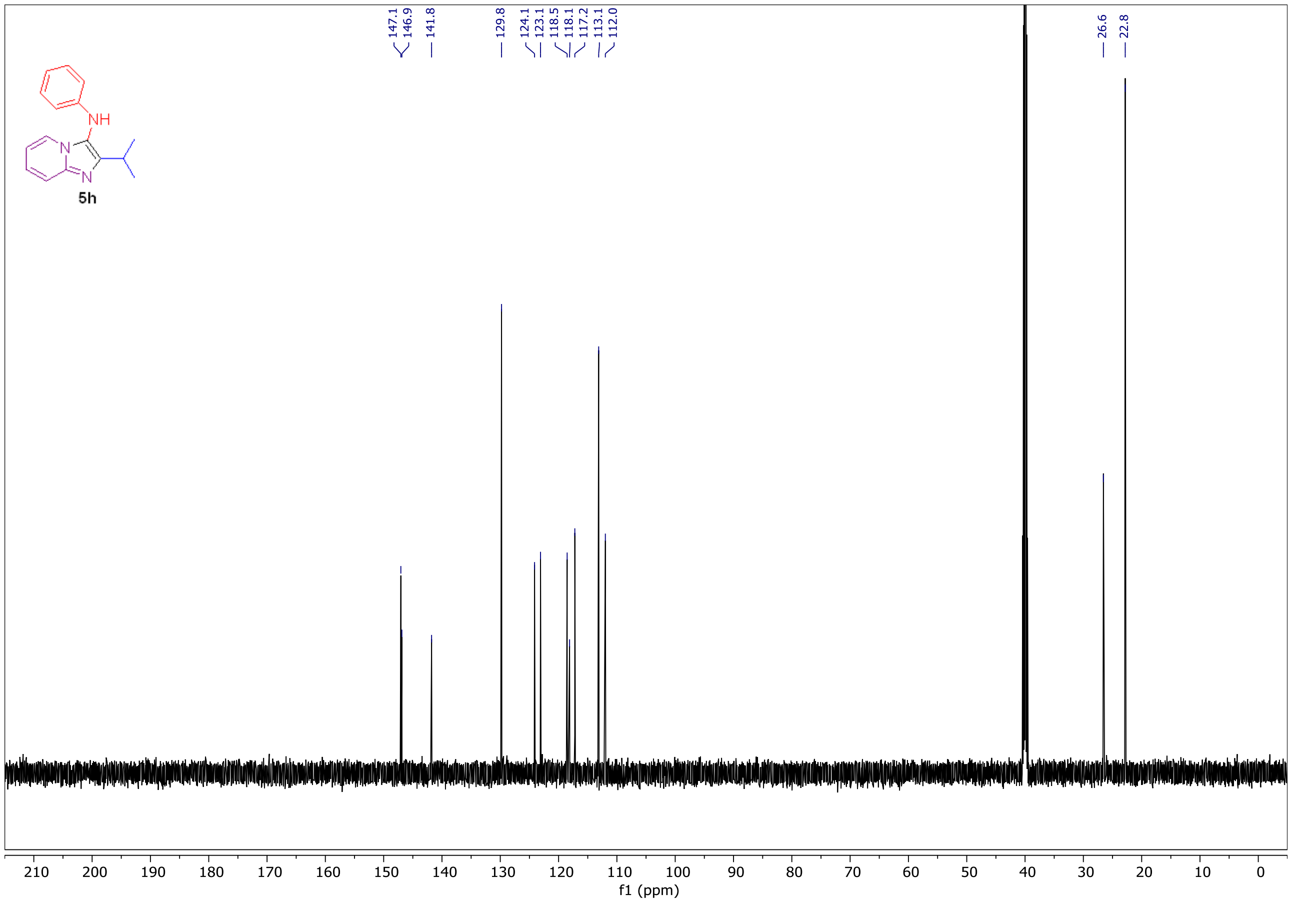

S 243. ^{13}C NMR spectrum (151 MHz, DMSO-d_6) of compound **5h**.

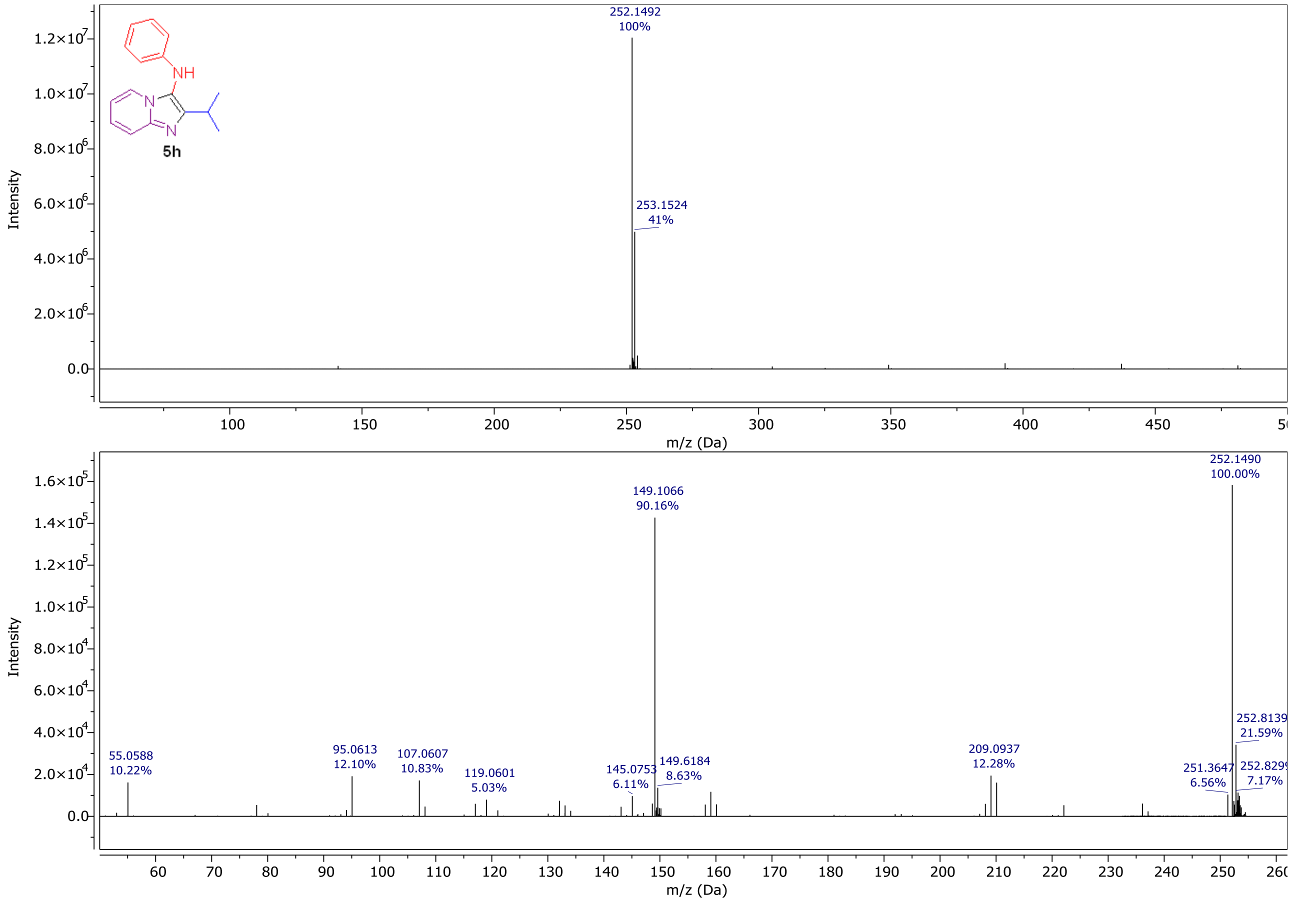

S 244. HRMS (ESI-QTOF) of compound **5h** and HRMS/MS for [M+H]$^+$.

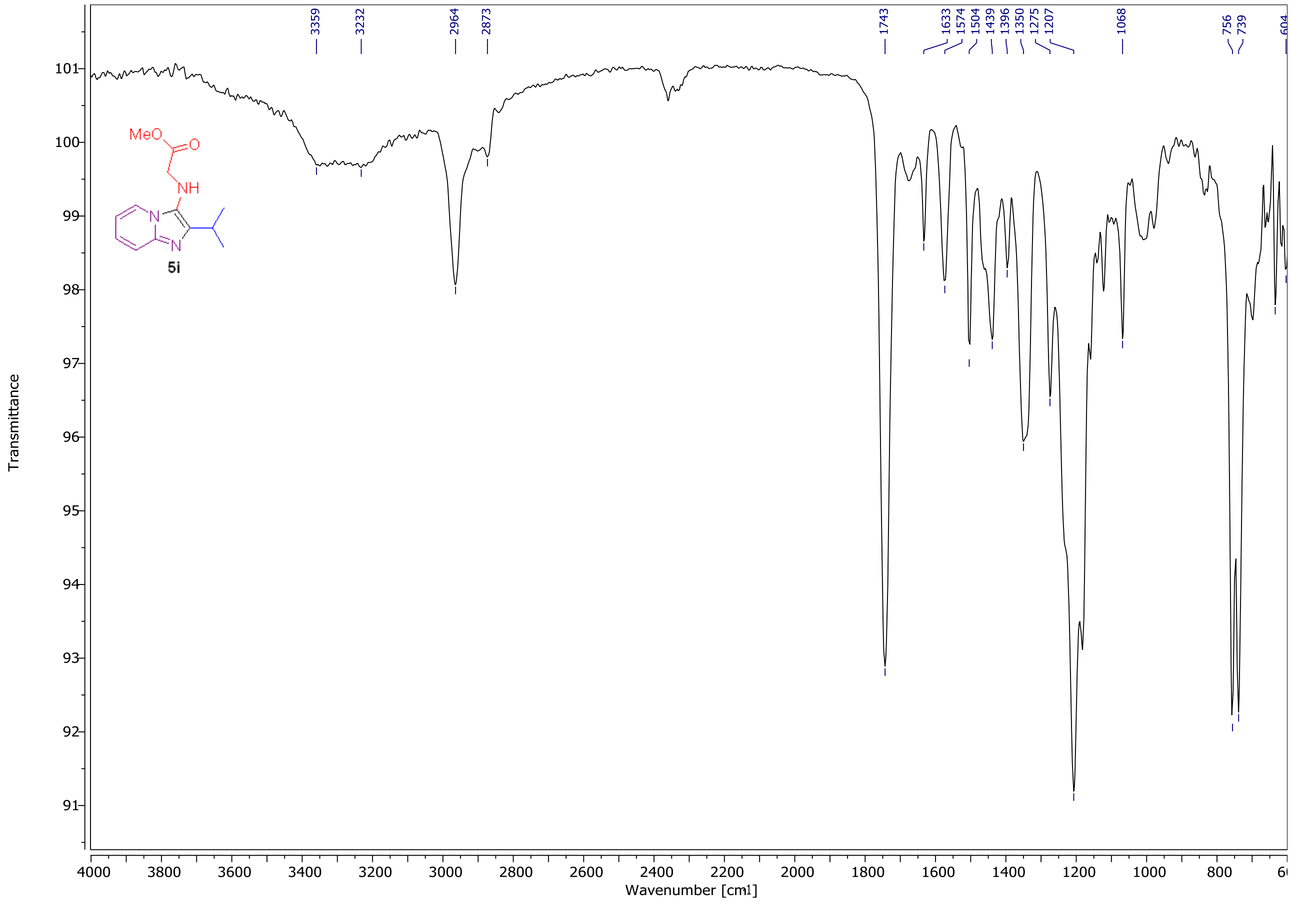

S 245. FT-IR (ATR) of compound **5i**.

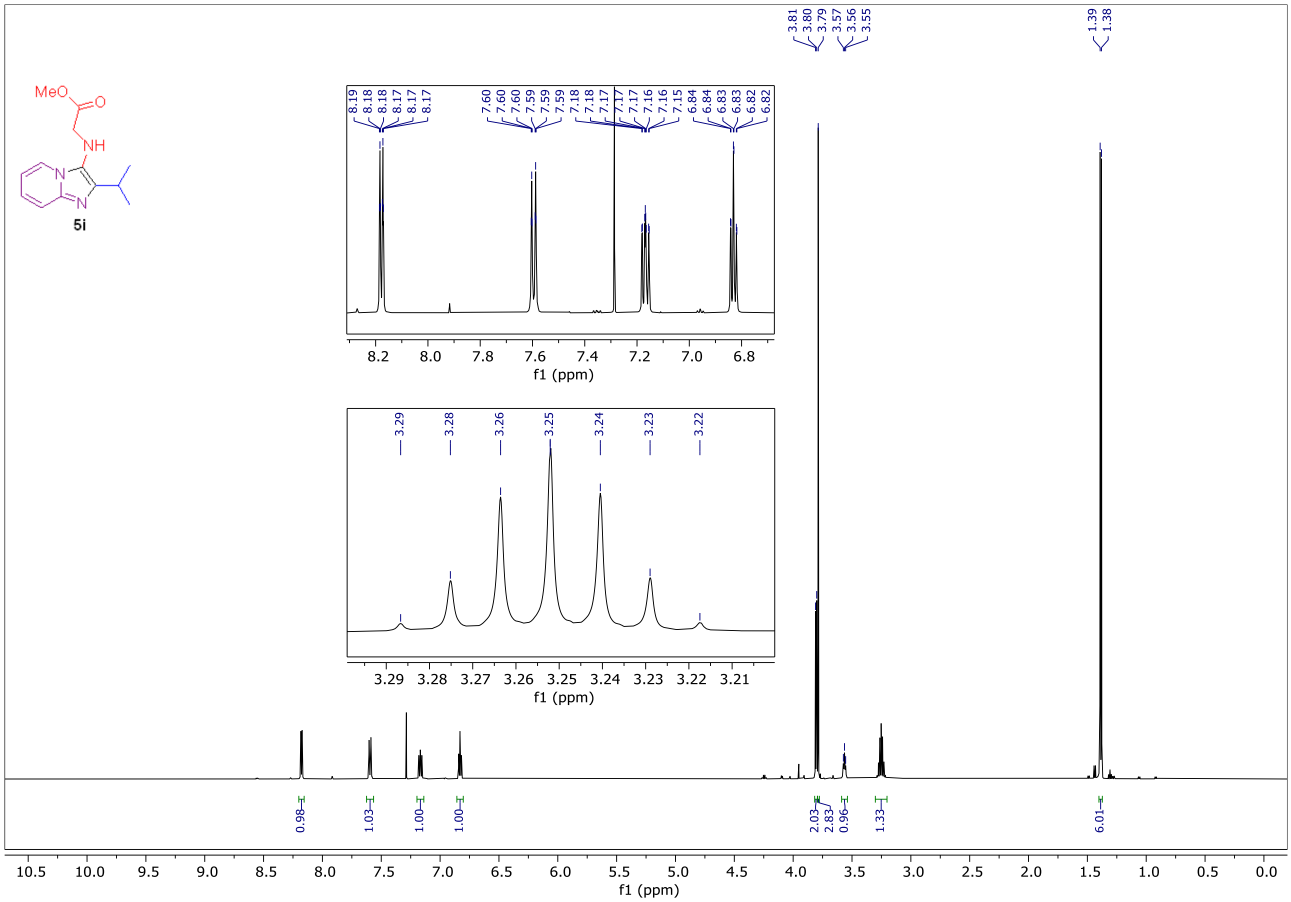

S 246. ^{1}H NMR spectrum (600 MHz, $CDCl_3$) of compound **5i**.

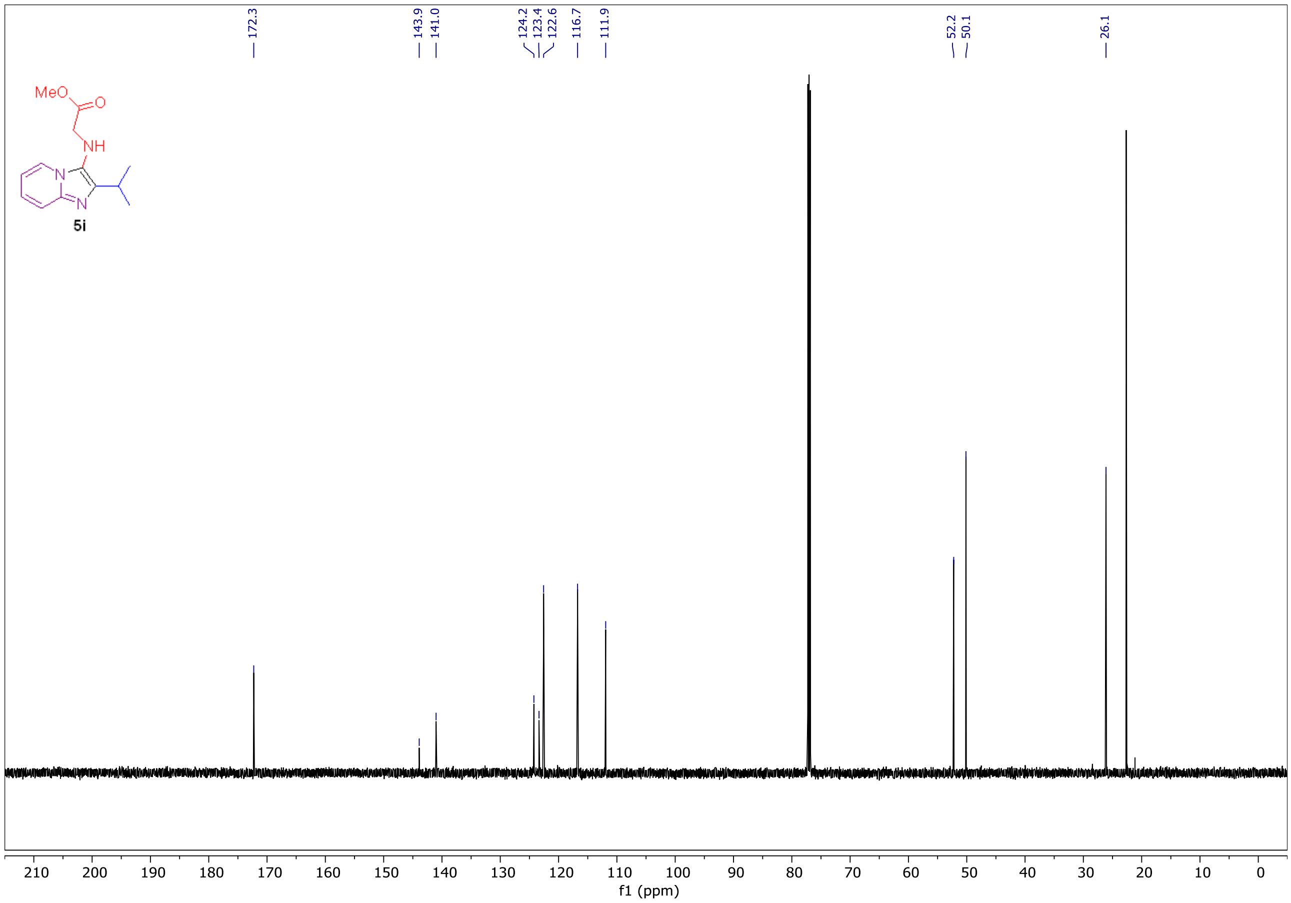

S 247. ^{13}C NMR spectrum (151 MHz, $CDCl_3$) of compound **5i**.

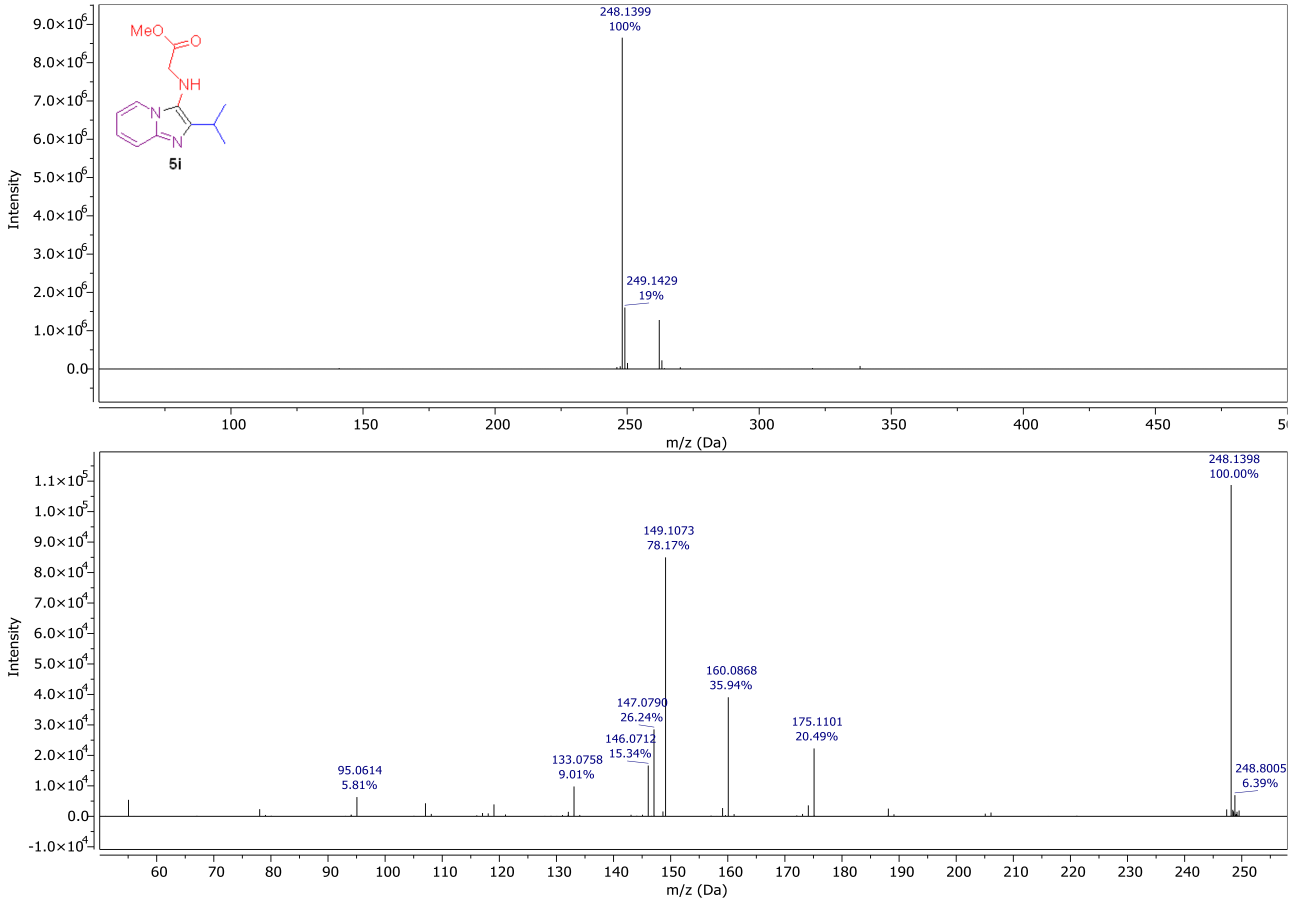

S 248. HRMS (ESI-QTOF) of compound **5i** and HRMS/MS for $[M+H]^+$.

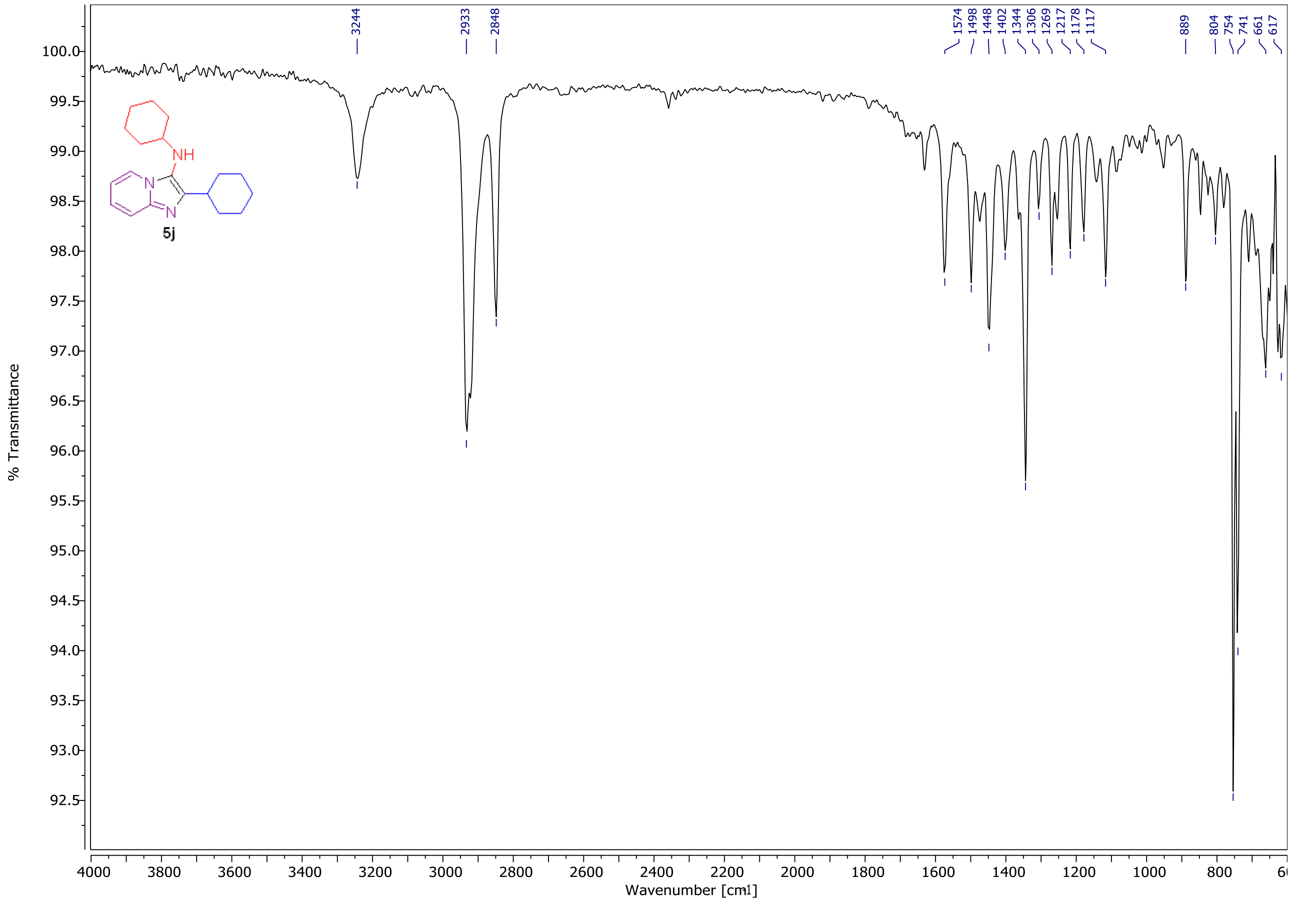

S 249. FT-IR (ATR) of compound **5j**.

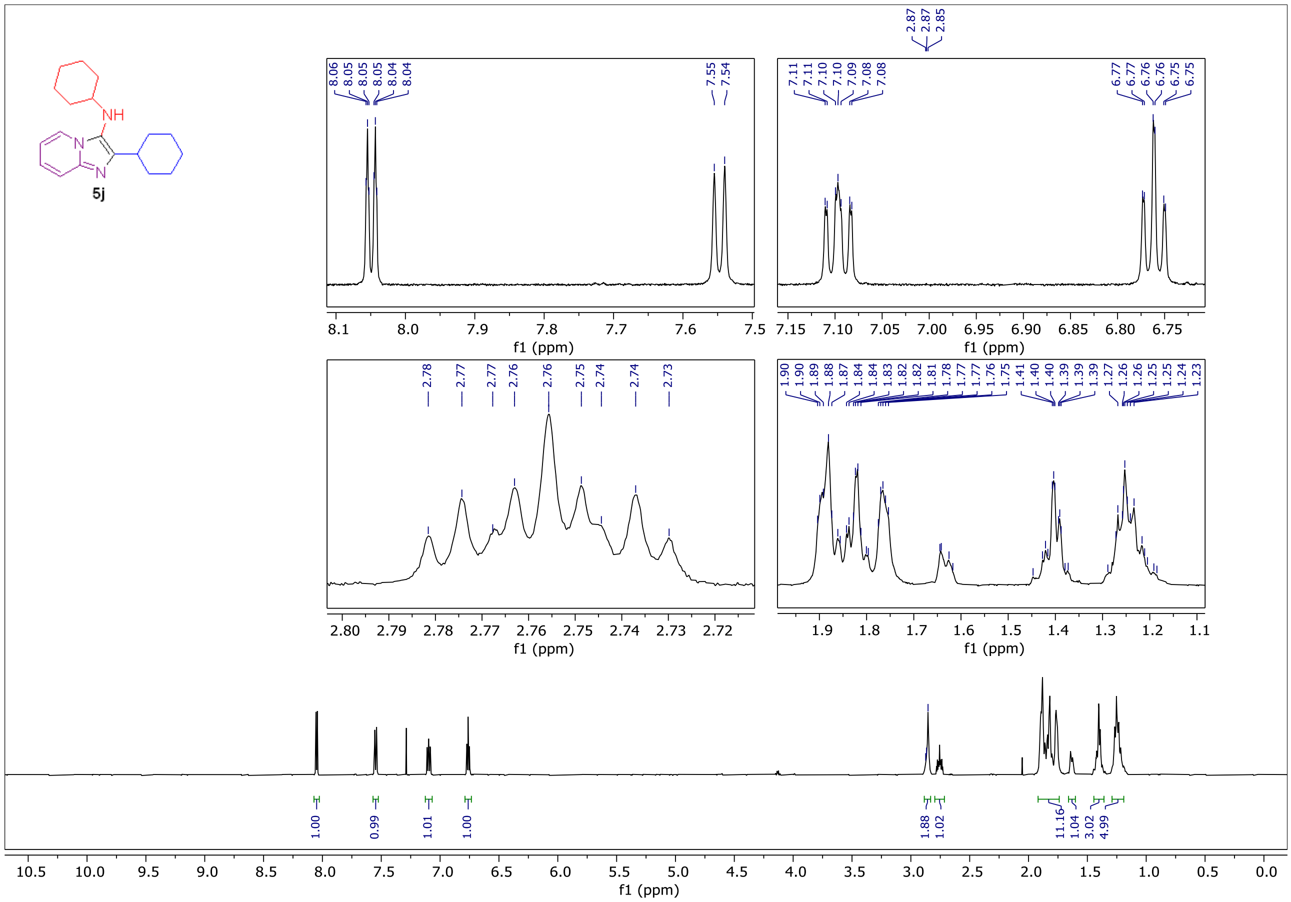

S 250. ^{1}H NMR spectrum (600 MHz, $CDCl_3$) of compound **5j**.

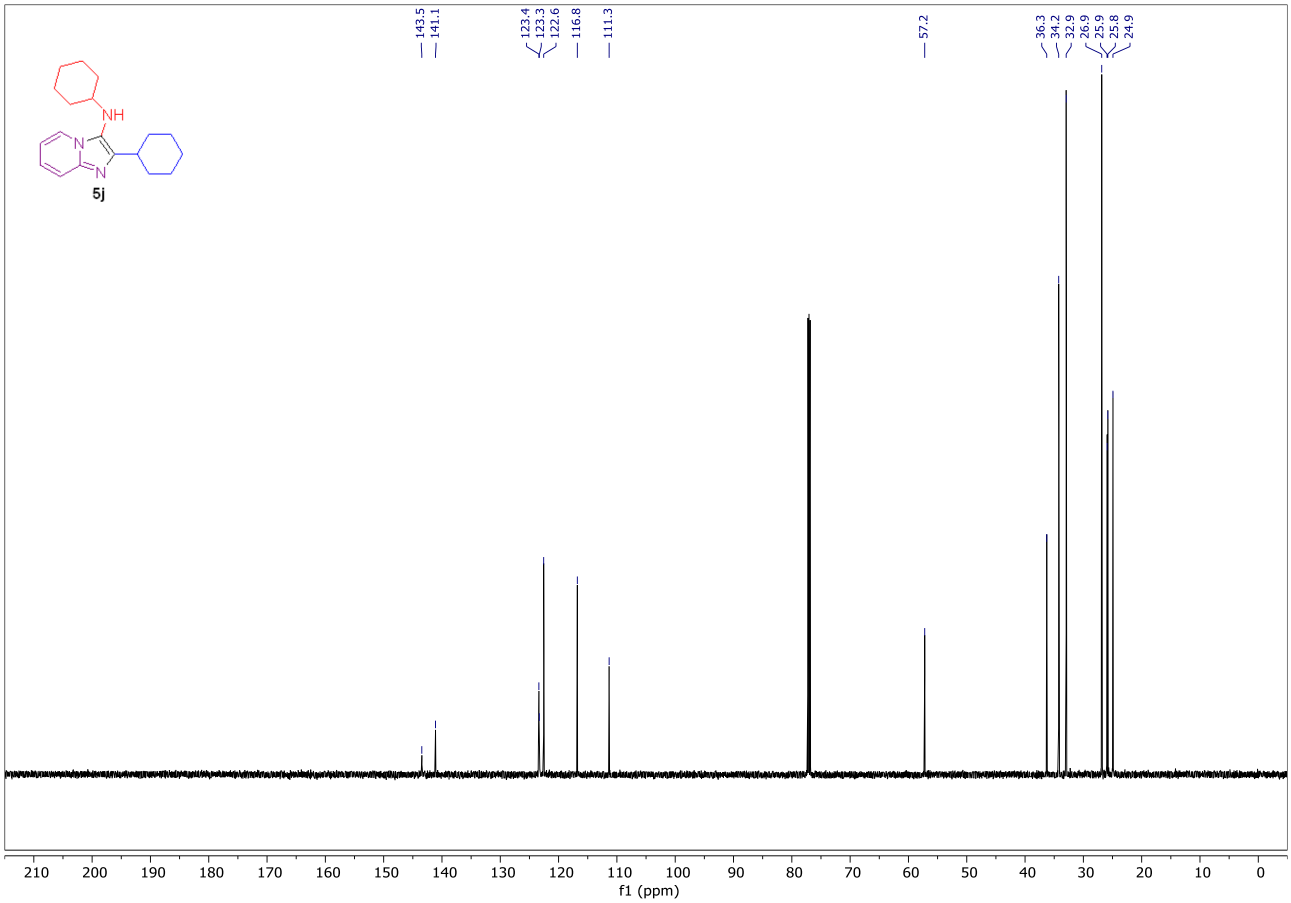

S 251. ^{13}C NMR spectrum (151 MHz, $CDCl_3$) of compound **5j**.

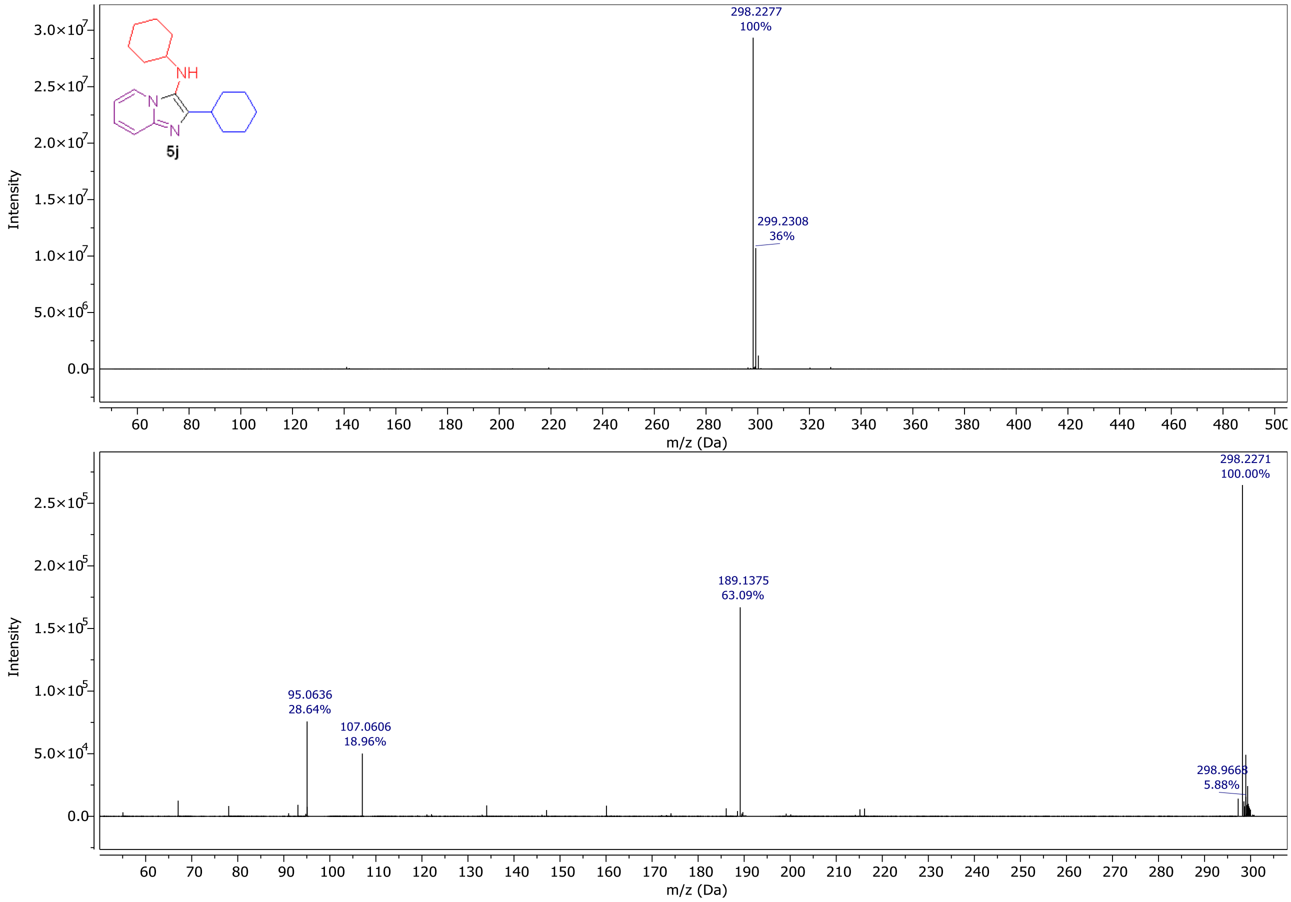

S 252. HRMS (ESI-QTOF) of compound **5j** and HRMS/MS for $[M+H]^+$.

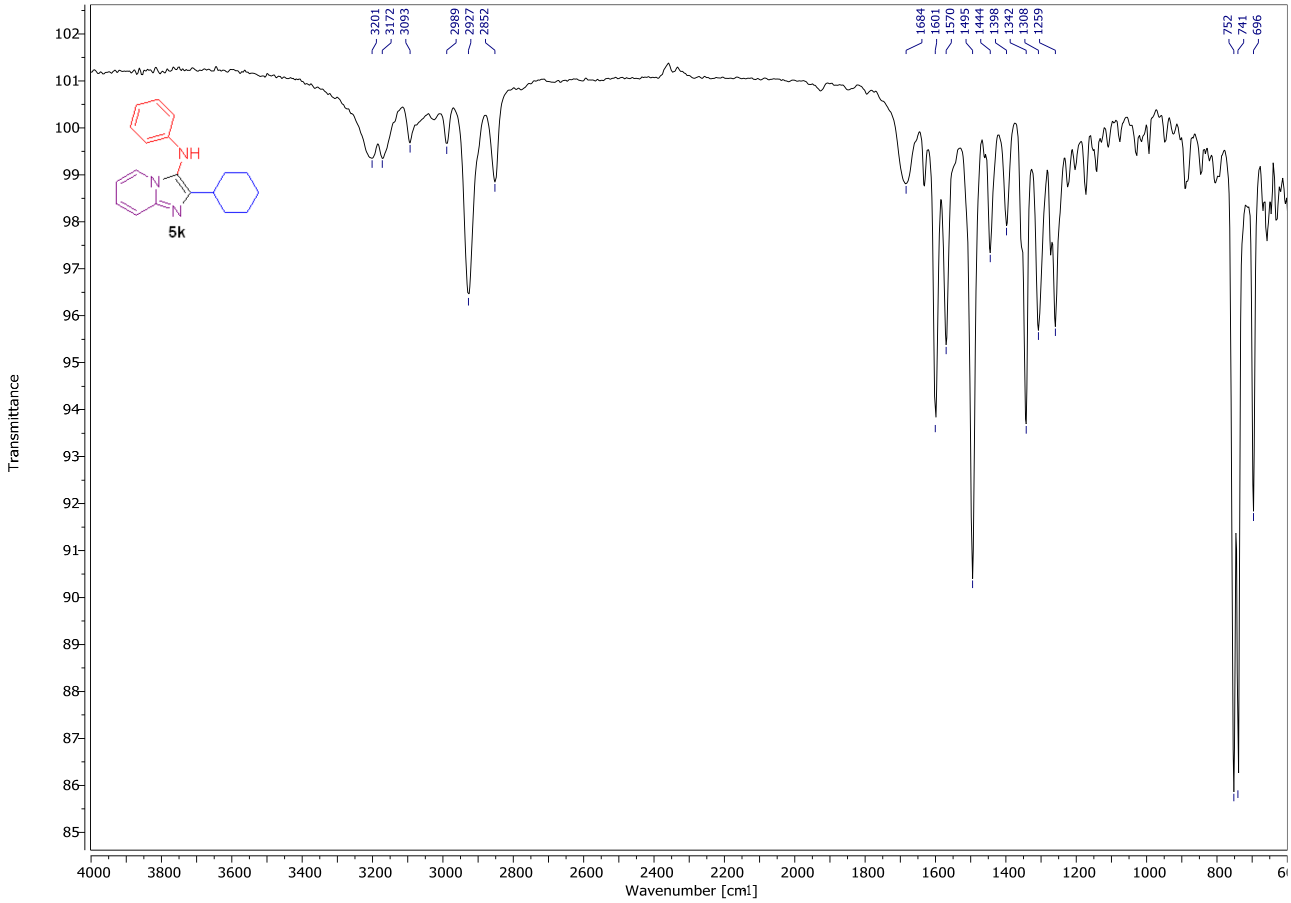

S 253. FT-IR (ATR) of compound **5k**.

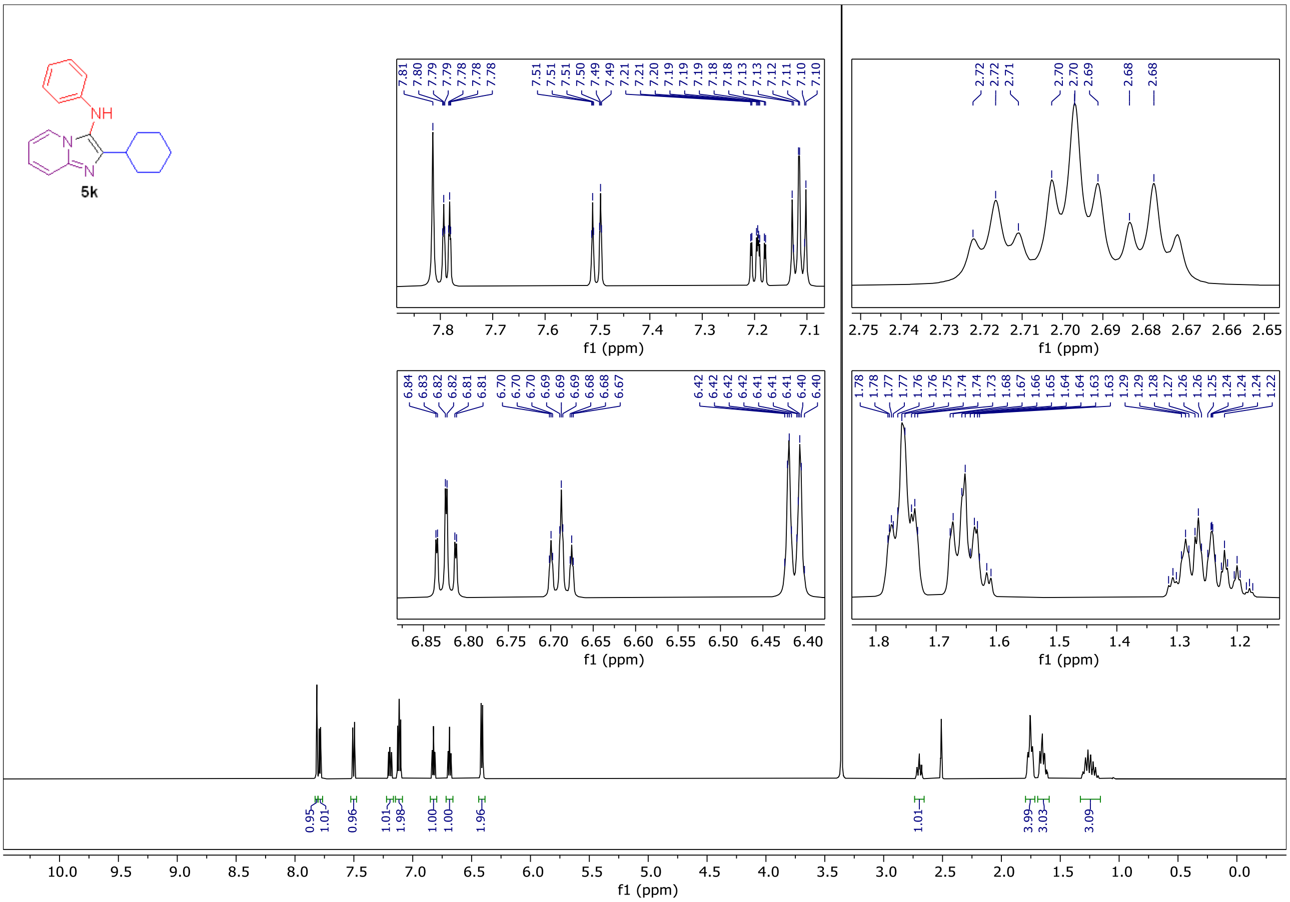

S 254. ^{1}H NMR spectrum (600 MHz, DMSO-d_6) of compound **5k**.

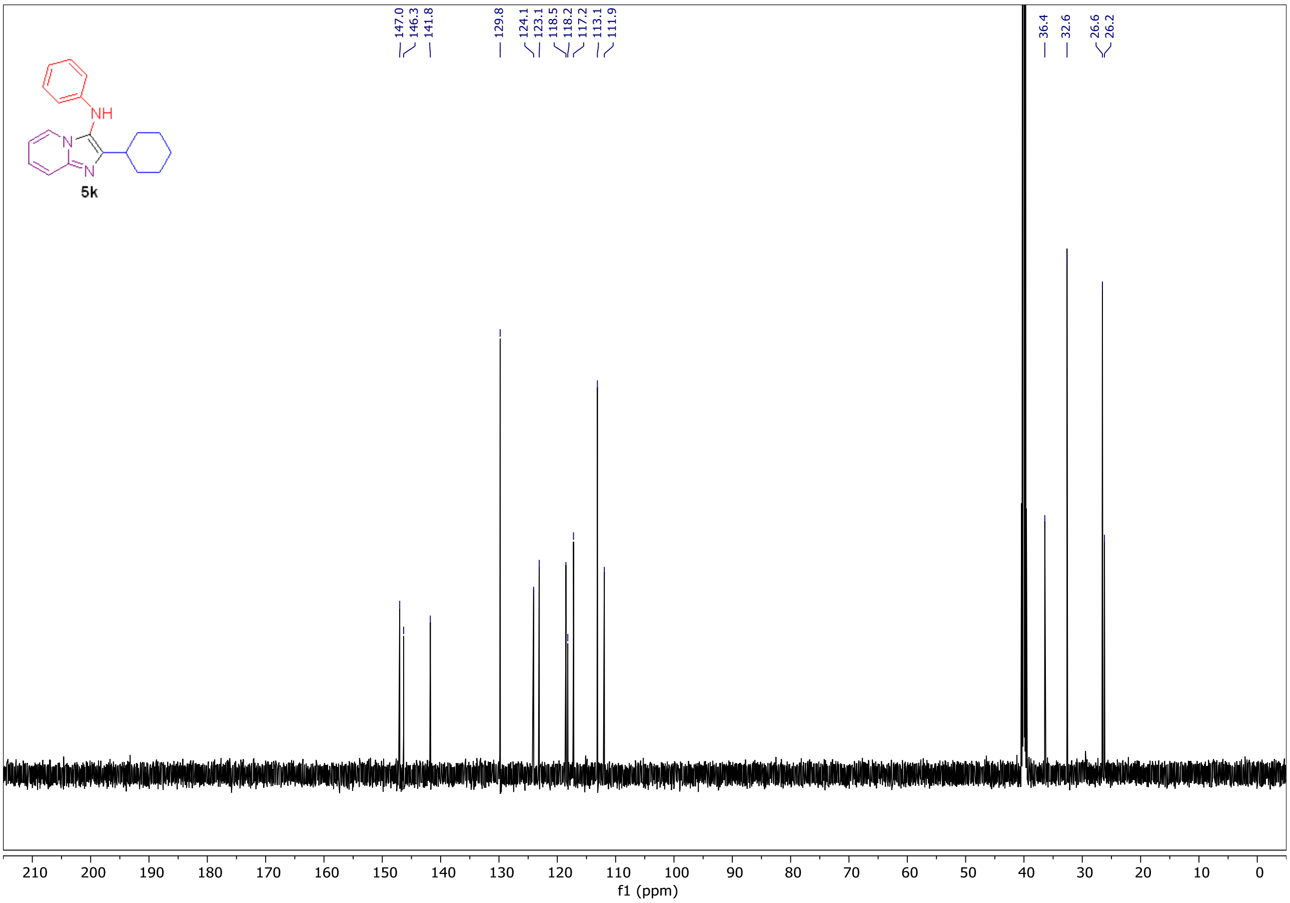

S 255. ^{13}C NMR spectrum (151 MHz, DMSO-d_6) of compound **5k**.

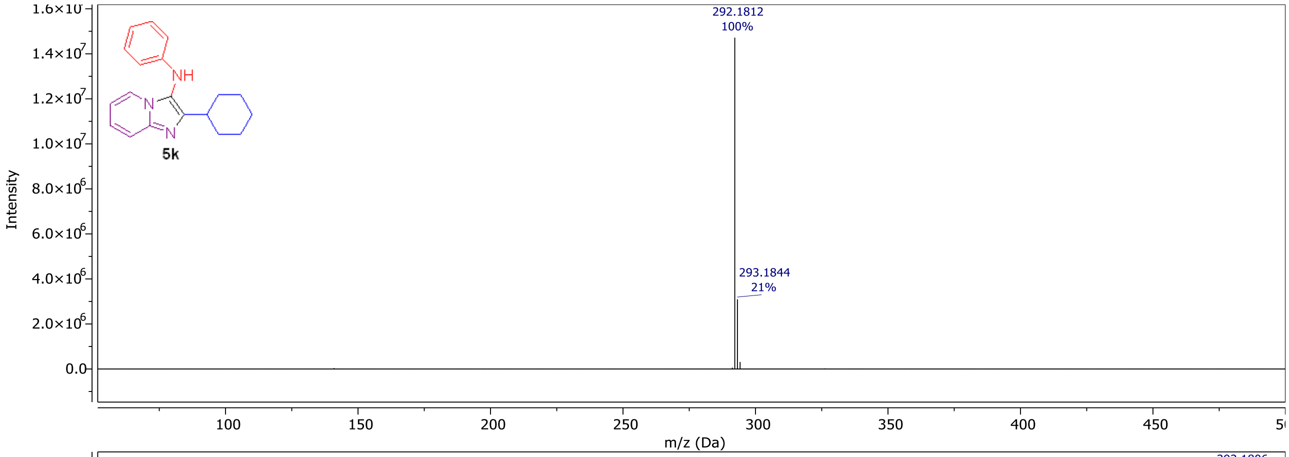

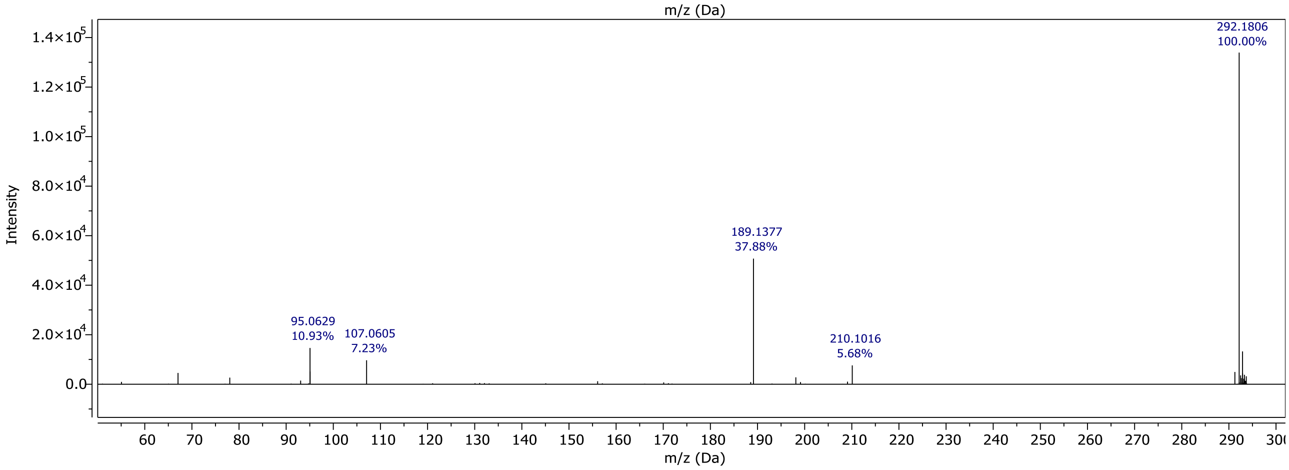

S 256. HRMS (ESI-QTOF) of compound **5k** and HRMS/MS for $[M+H]^+$.

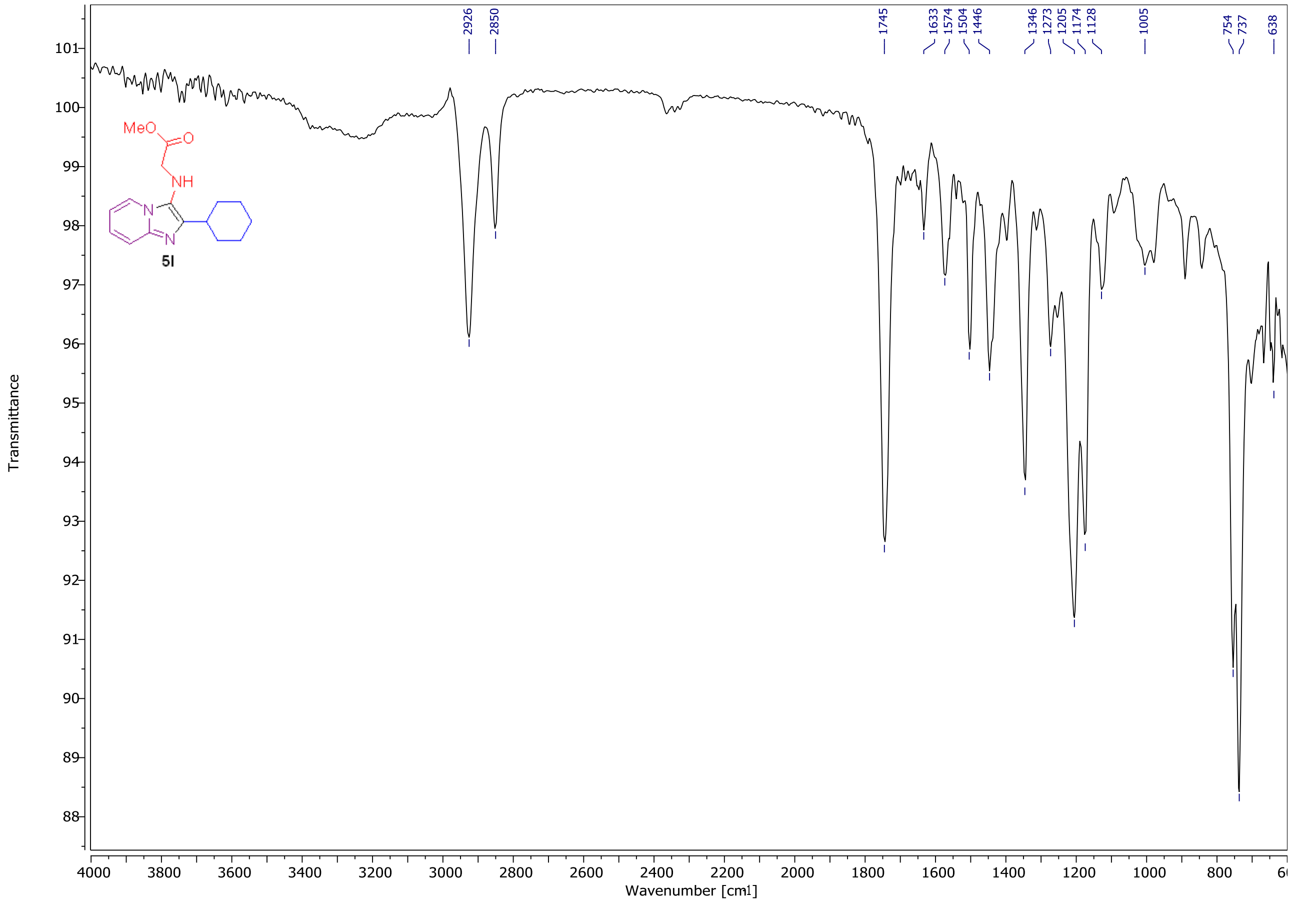

S 257. FT-IR (ATR) of compound **5l**.

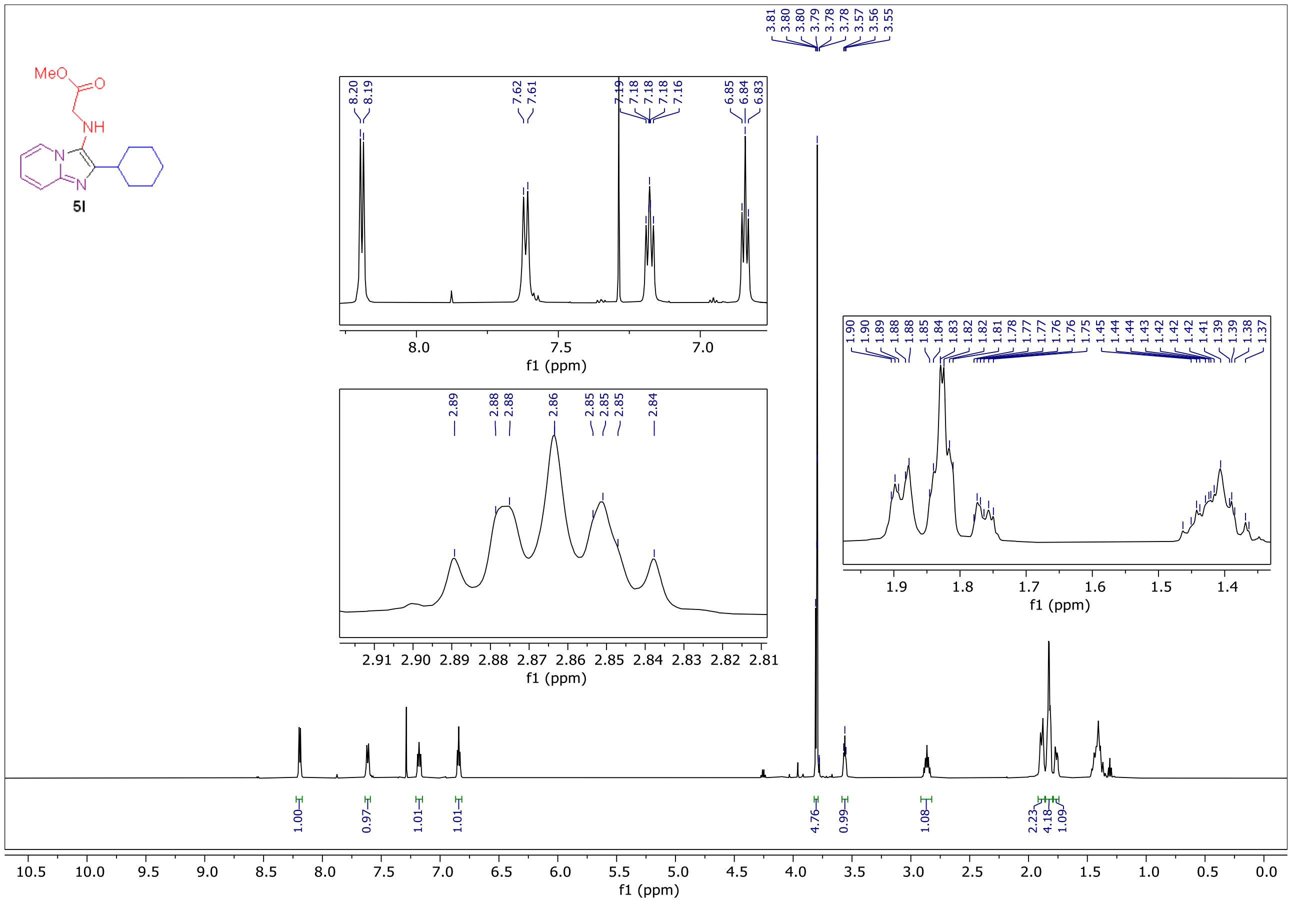

S 258. ^{1}H NMR spectrum (600 MHz, $CDCl_3$) of compound **5l**.

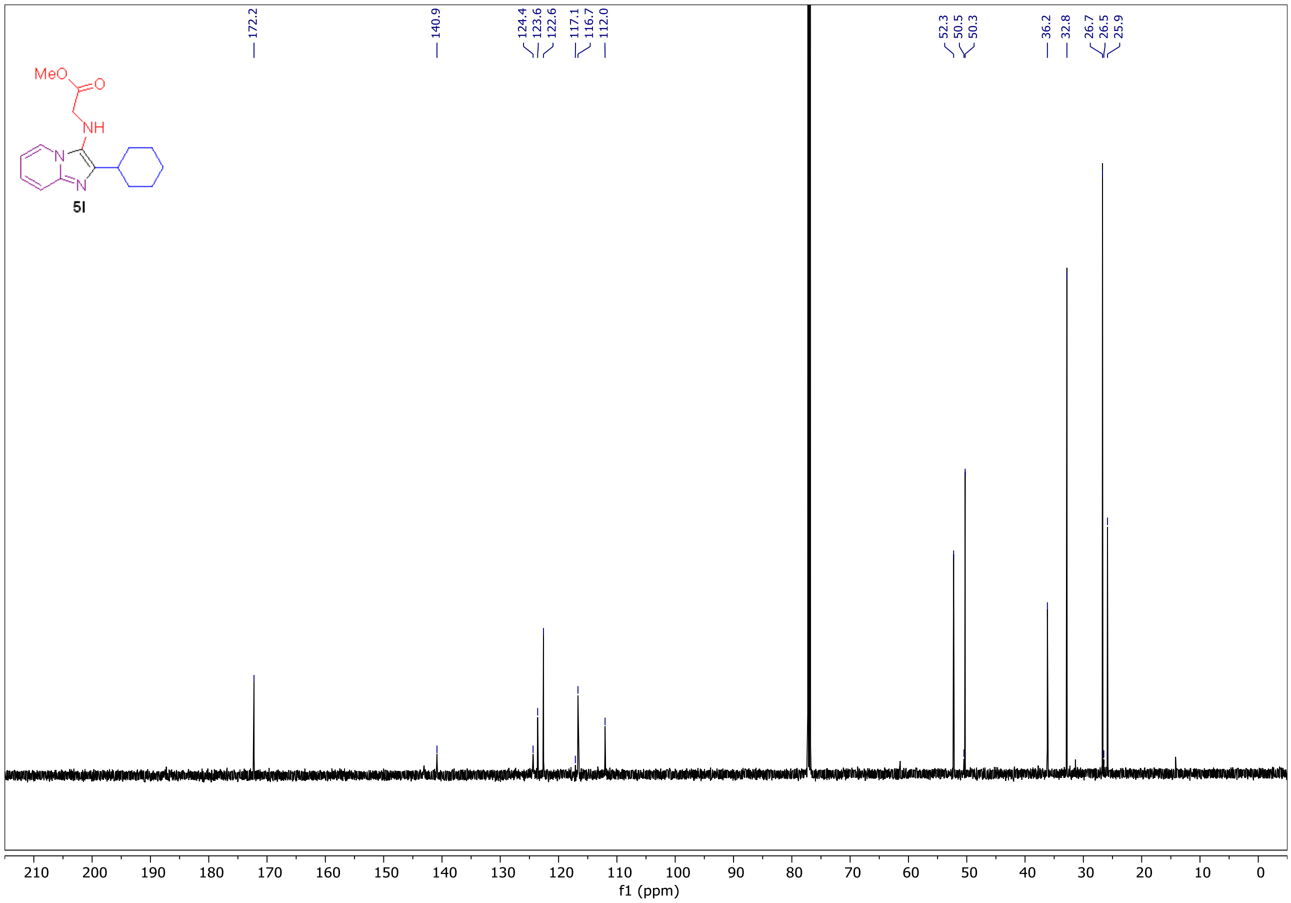

S 259. ^{13}C NMR spectrum (151 MHz, $CDCl_3$) of compound **5l**.

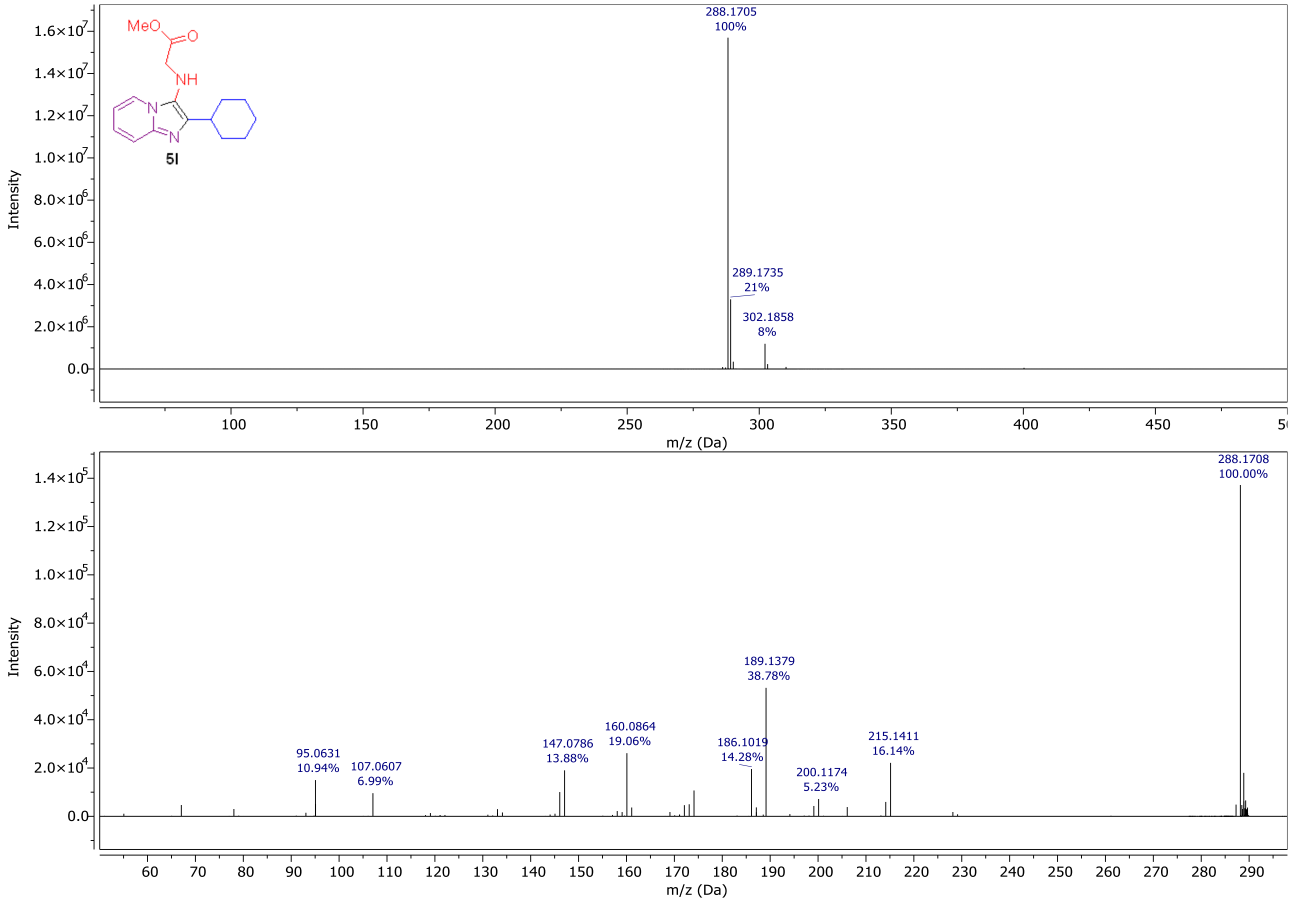

S 260. HRMS (ESI-QTOF) of compound **5l** and HRMS/MS for $[M+H]^+$.

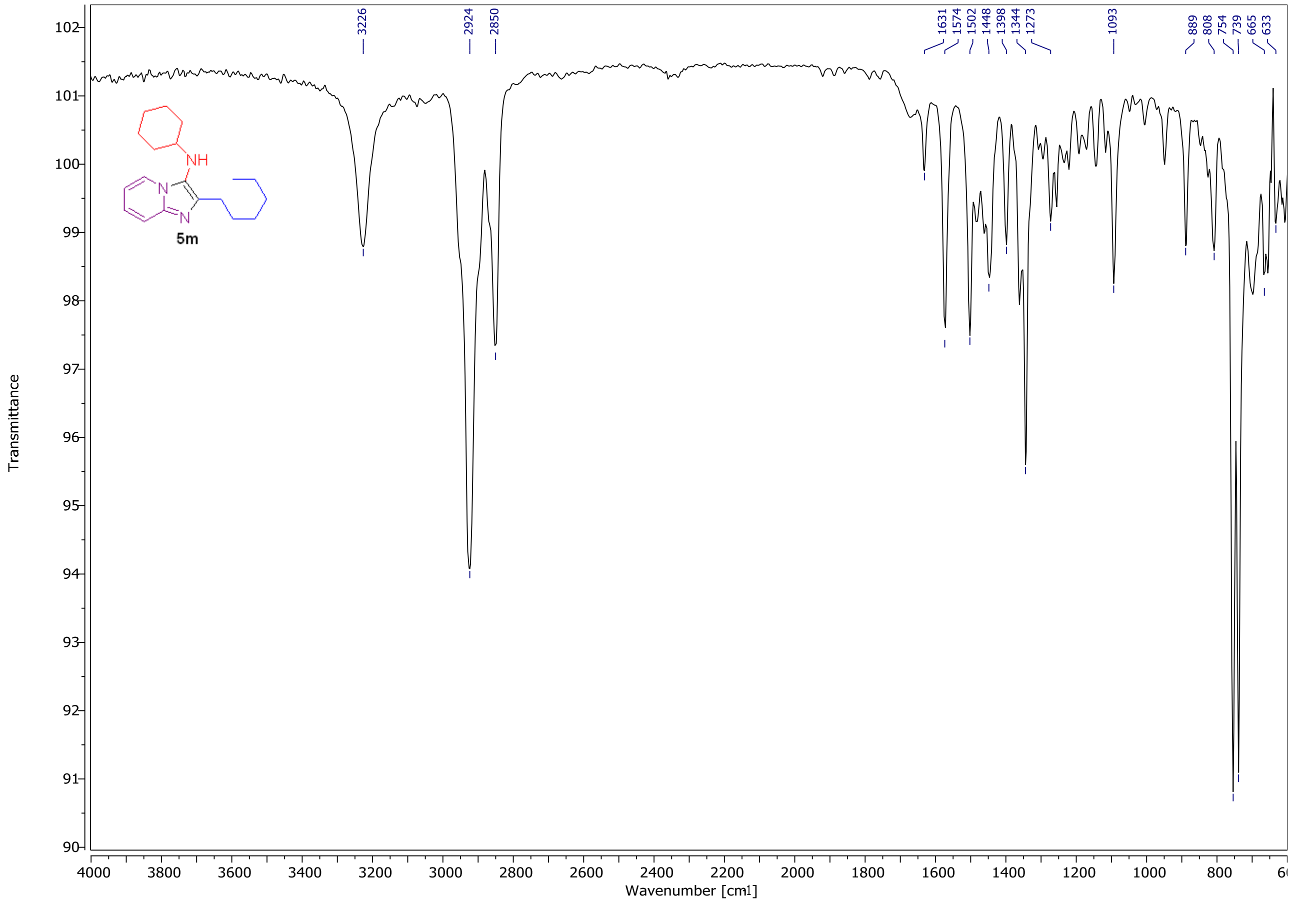

S 261. FT-IR (ATR) of compound **5m**.

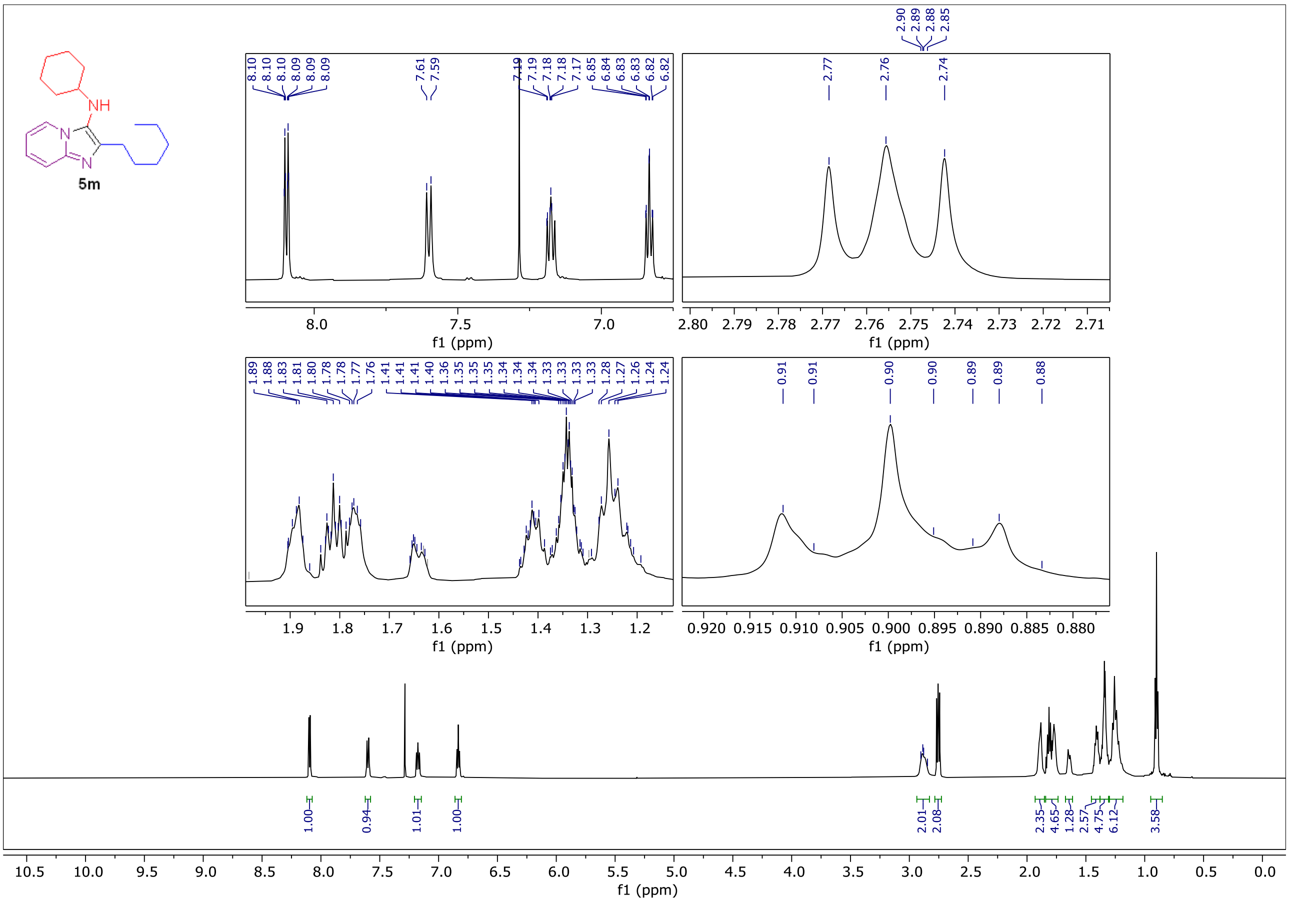

S 262. ^{1}H NMR spectrum (600 MHz, $CDCl_3$) of compound **5m**.

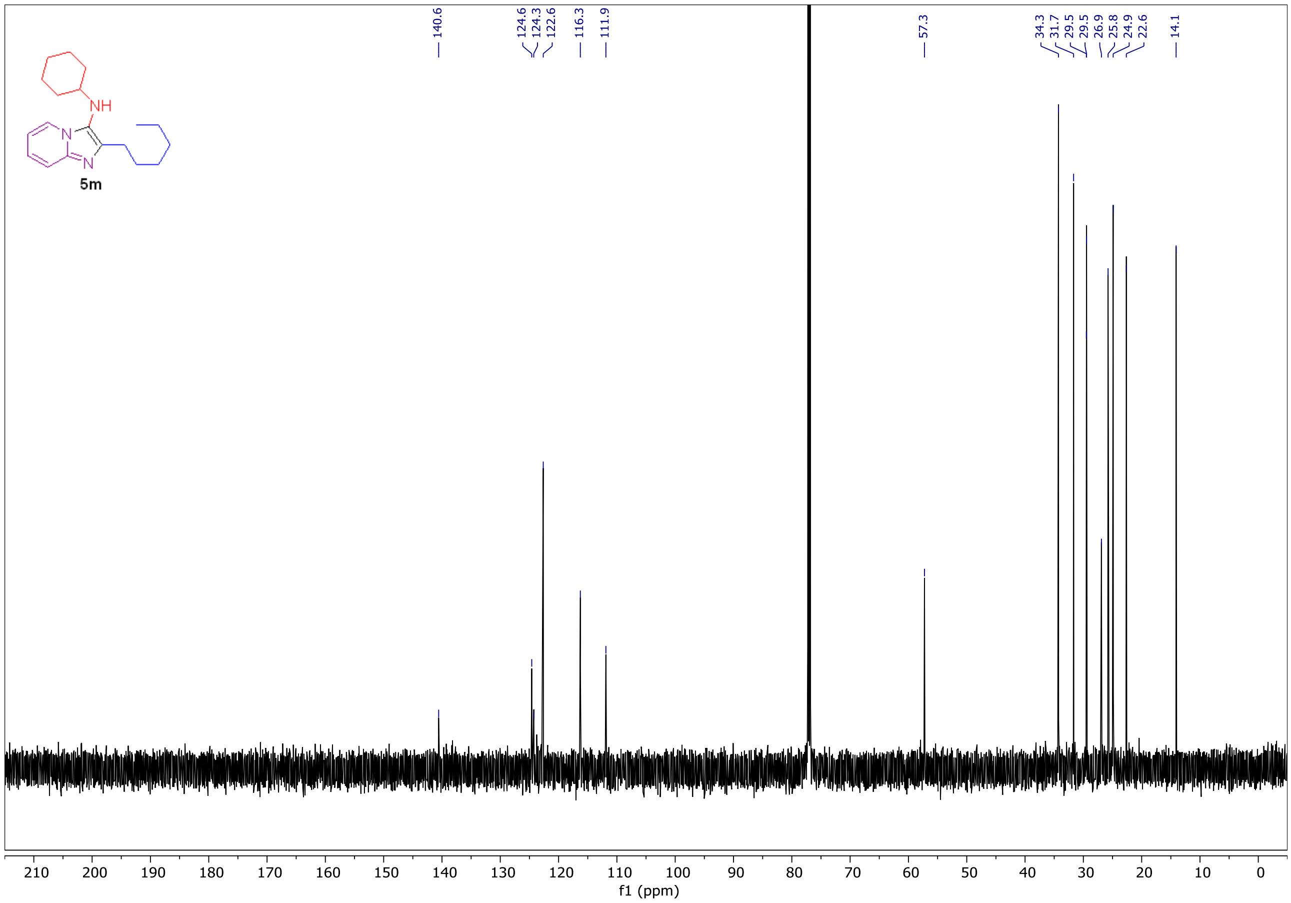

S 263. ^{13}C NMR spectrum (151 MHz, $CDCl_3$) of compound **5m**.

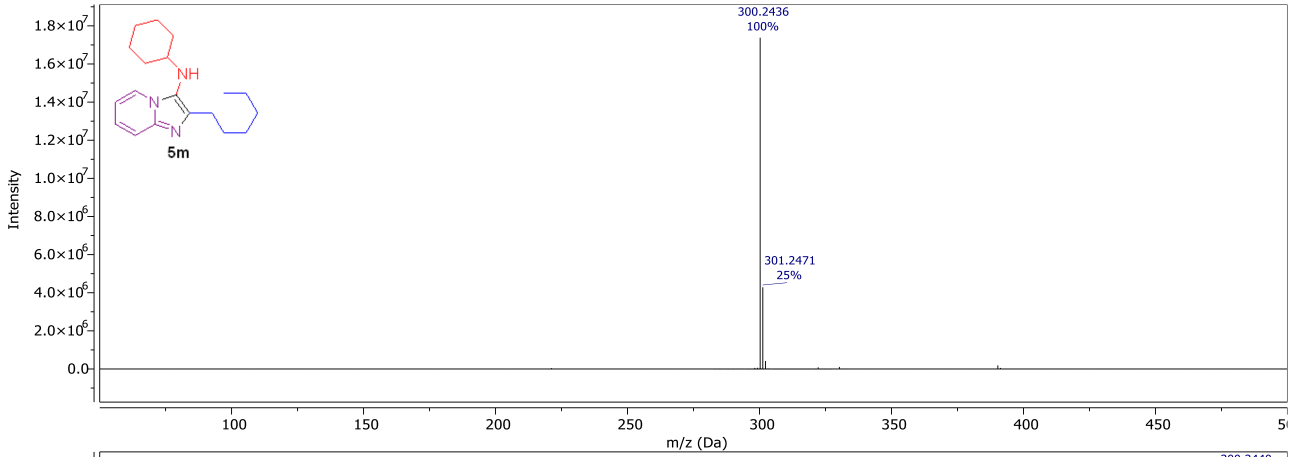

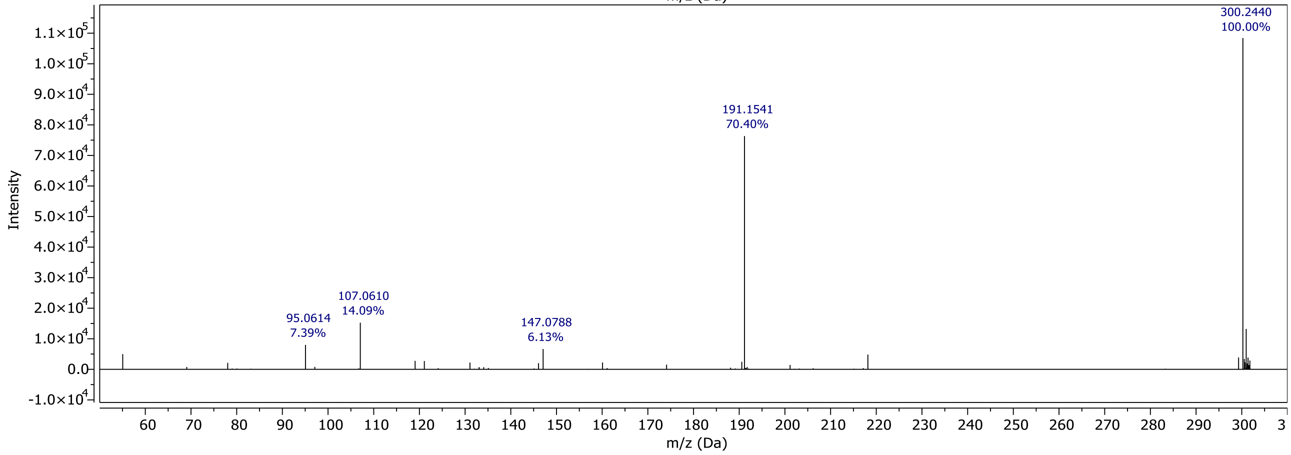

S 264. HRMS (ESI-QTOF) of compound **5m** and HRMS/MS for $[M+H]^+$.

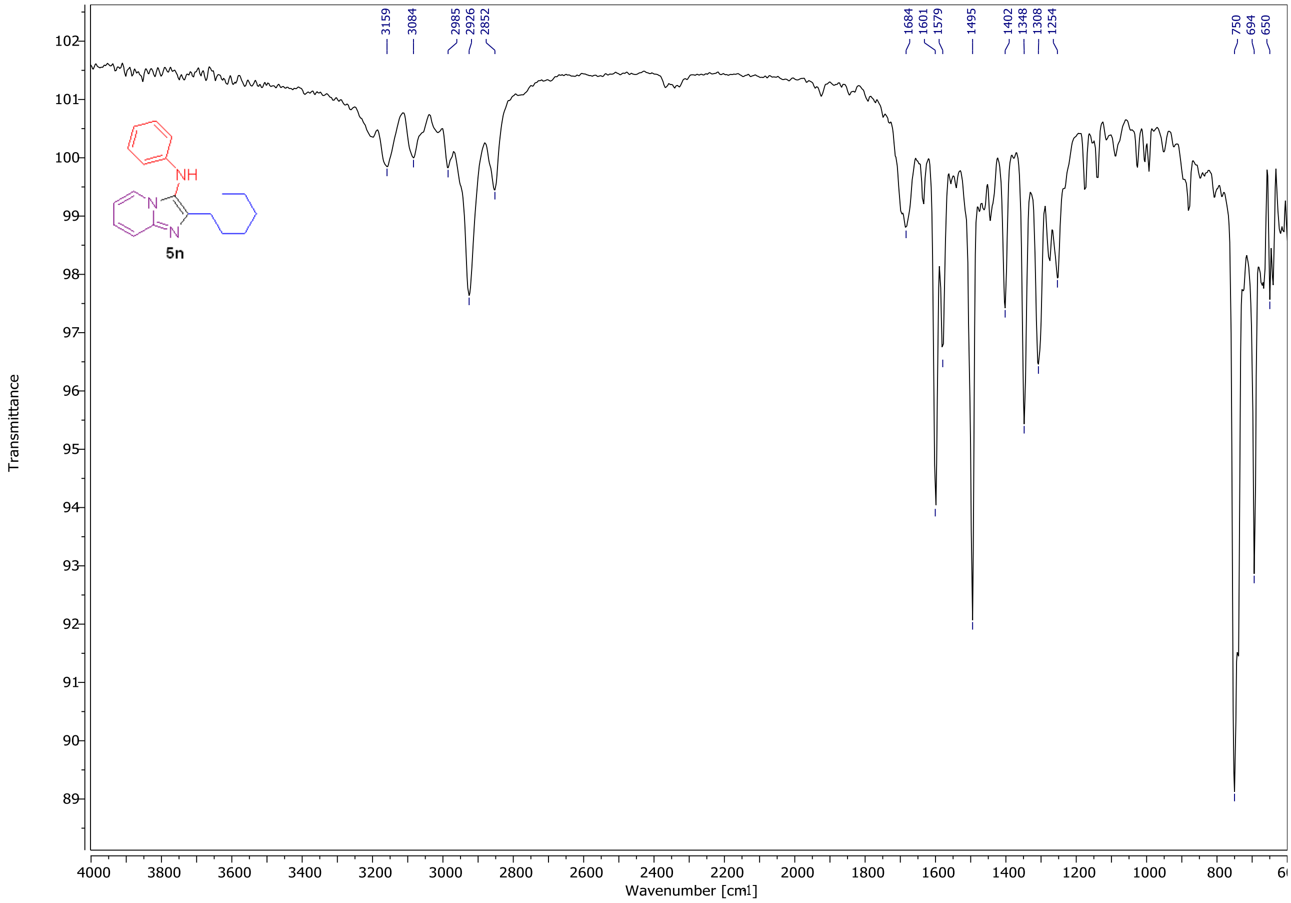

S 265. FT-IR (ATR) of compound **5n**.

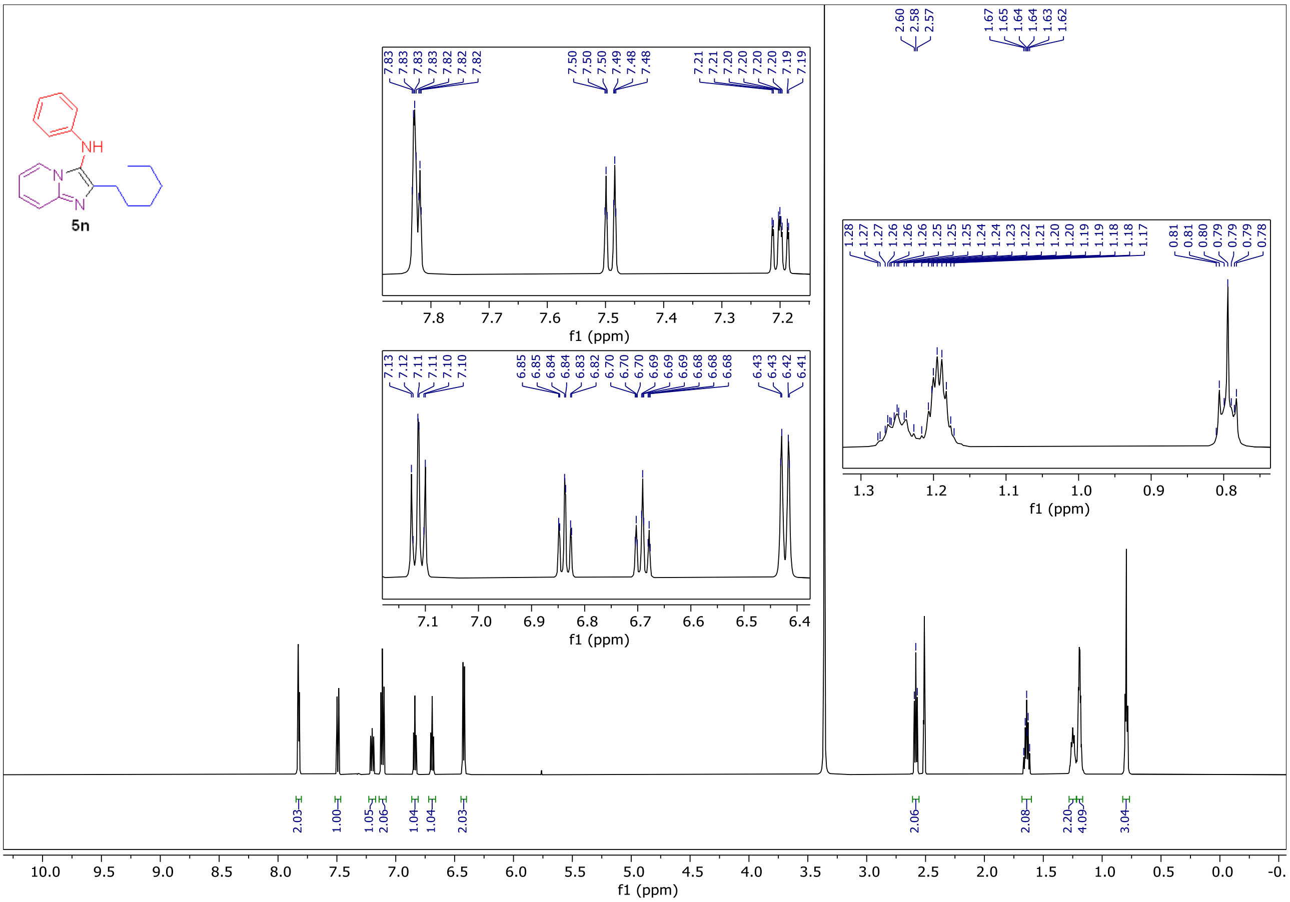

S 266. ^{1}H NMR spectrum (600 MHz, DMSO-d_6) of compound **5n**.

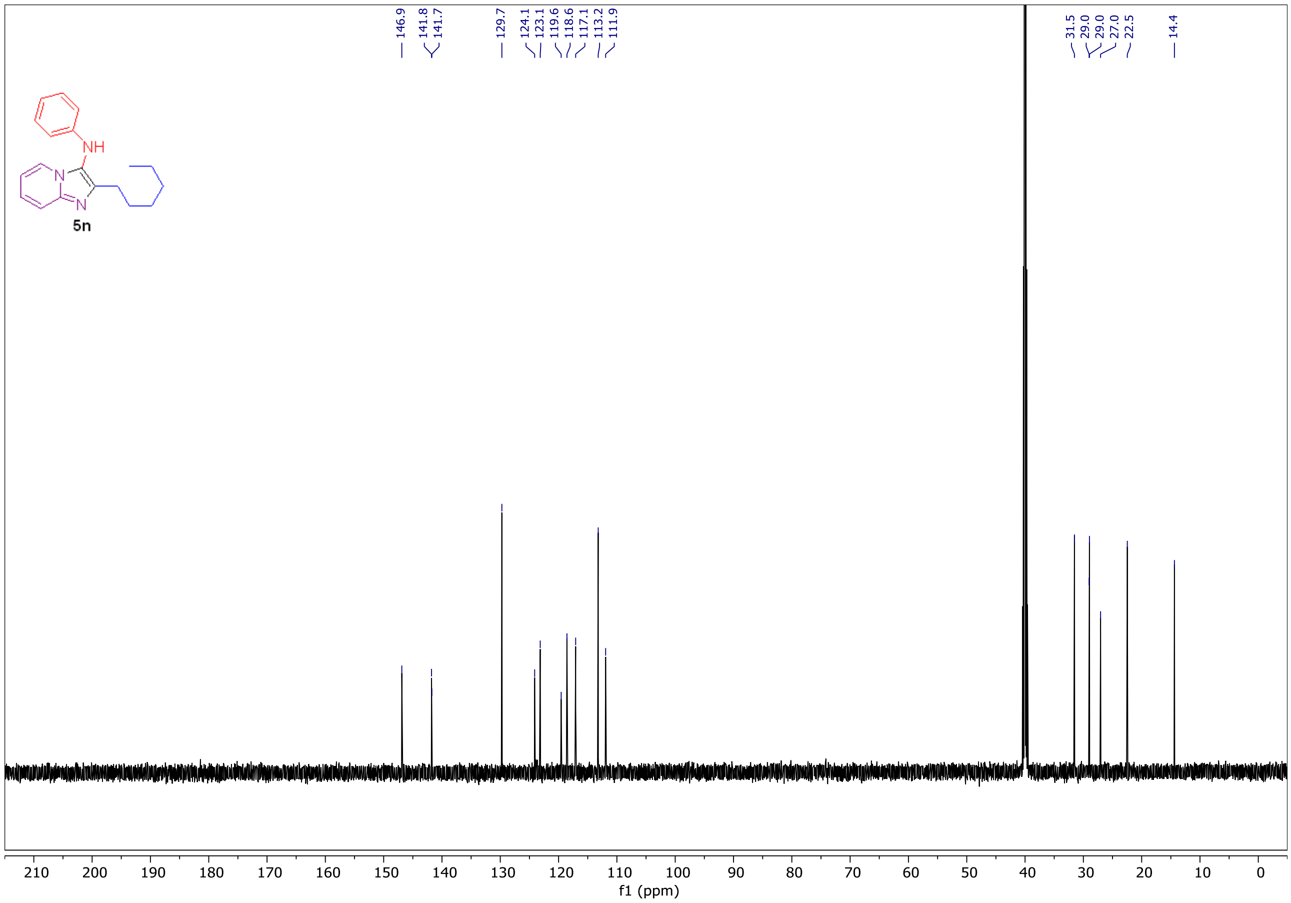

S 267. ^{13}C NMR spectrum (151 MHz, DMSO-d_6) of compound **5n**.

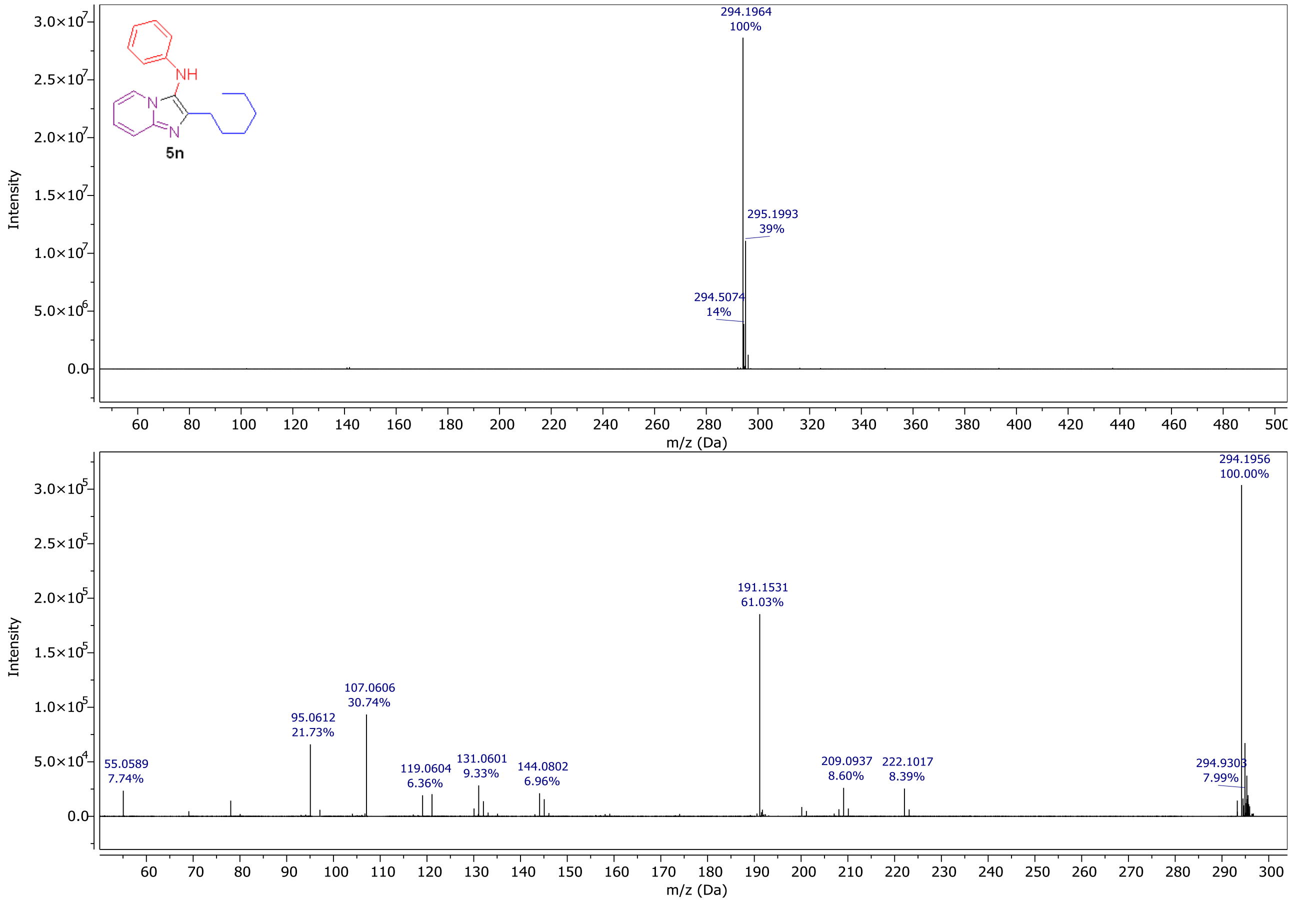

S 268. HRMS (ESI-QTOF) of compound **5n** and HRMS/MS for [M+H]$^+$.

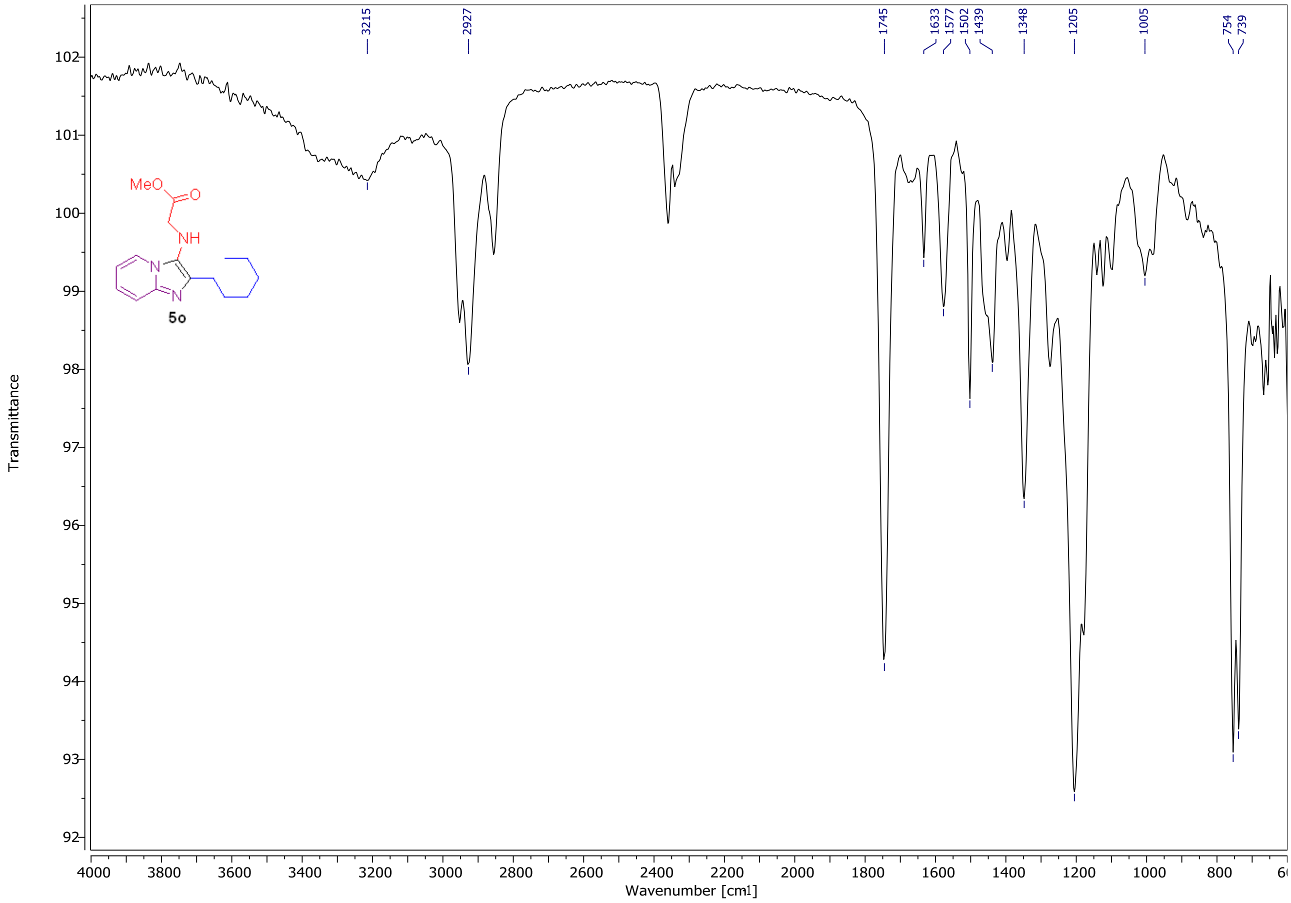

S 269. FT-IR (ATR) of compound **5o**.

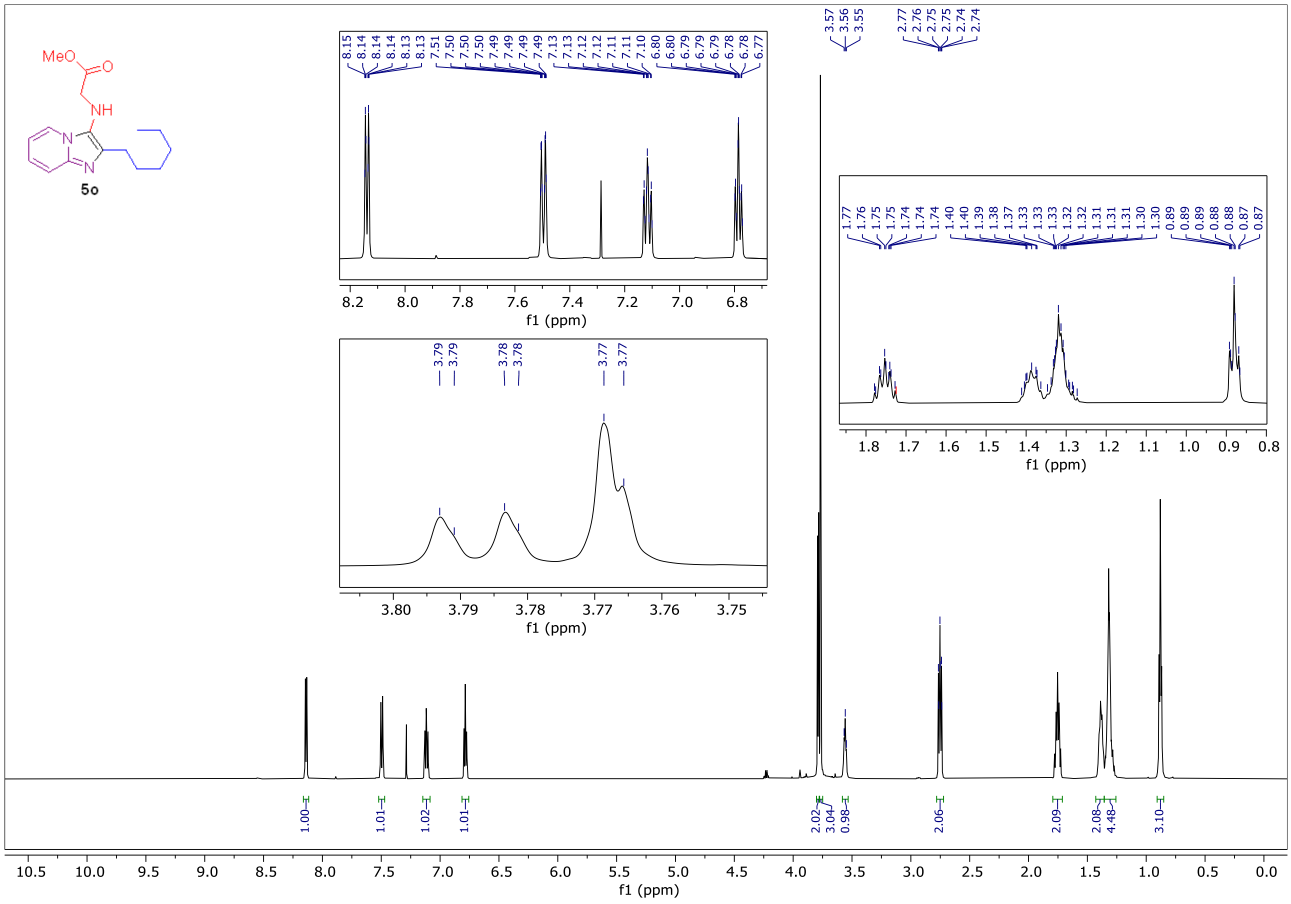

S 270. ^{1}H NMR spectrum (600 MHz, $CDCl_3$) of compound **5o**.

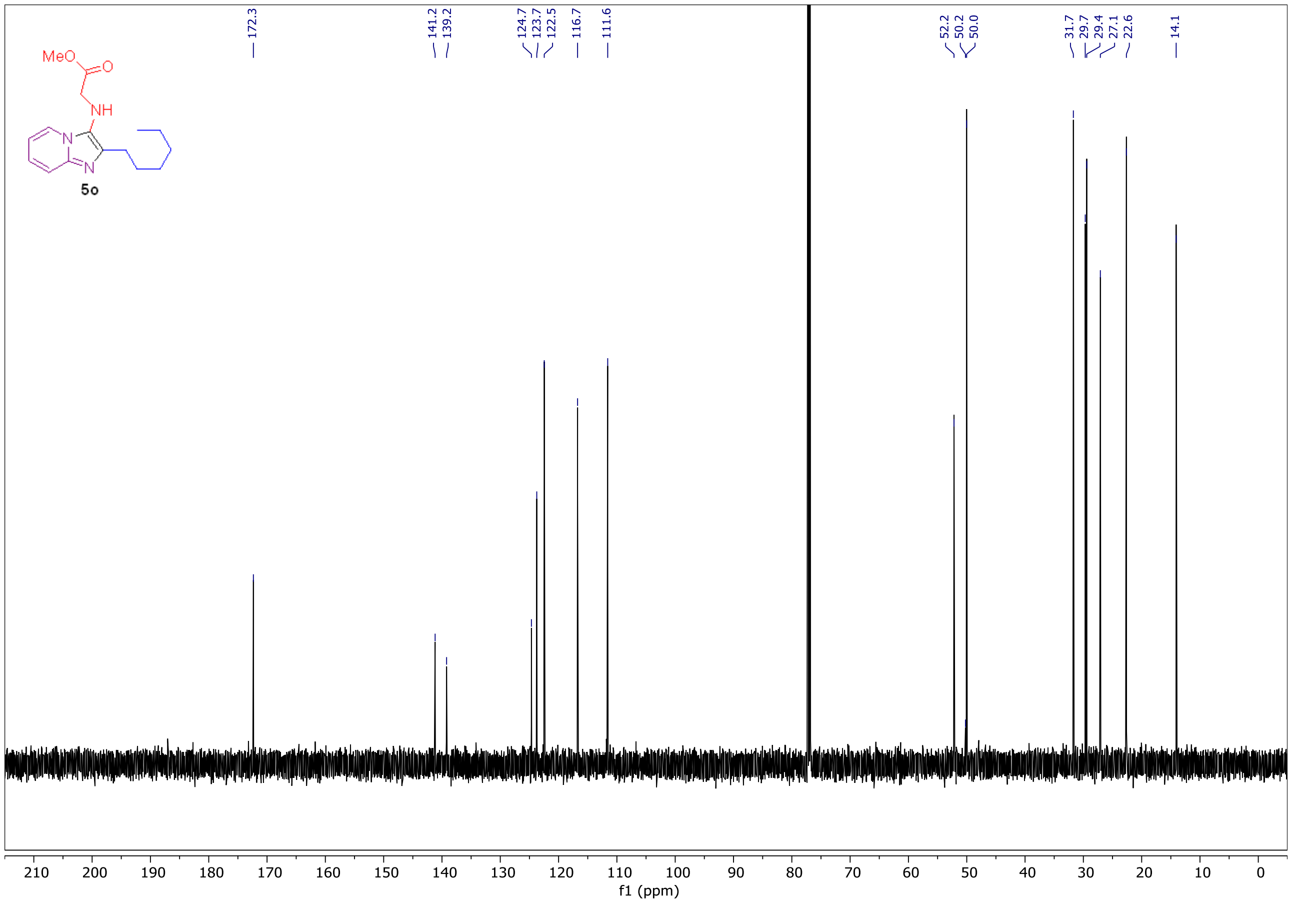

S 271. ^{13}C NMR spectrum (151 MHz, $CDCl_3$) of compound **5o**.

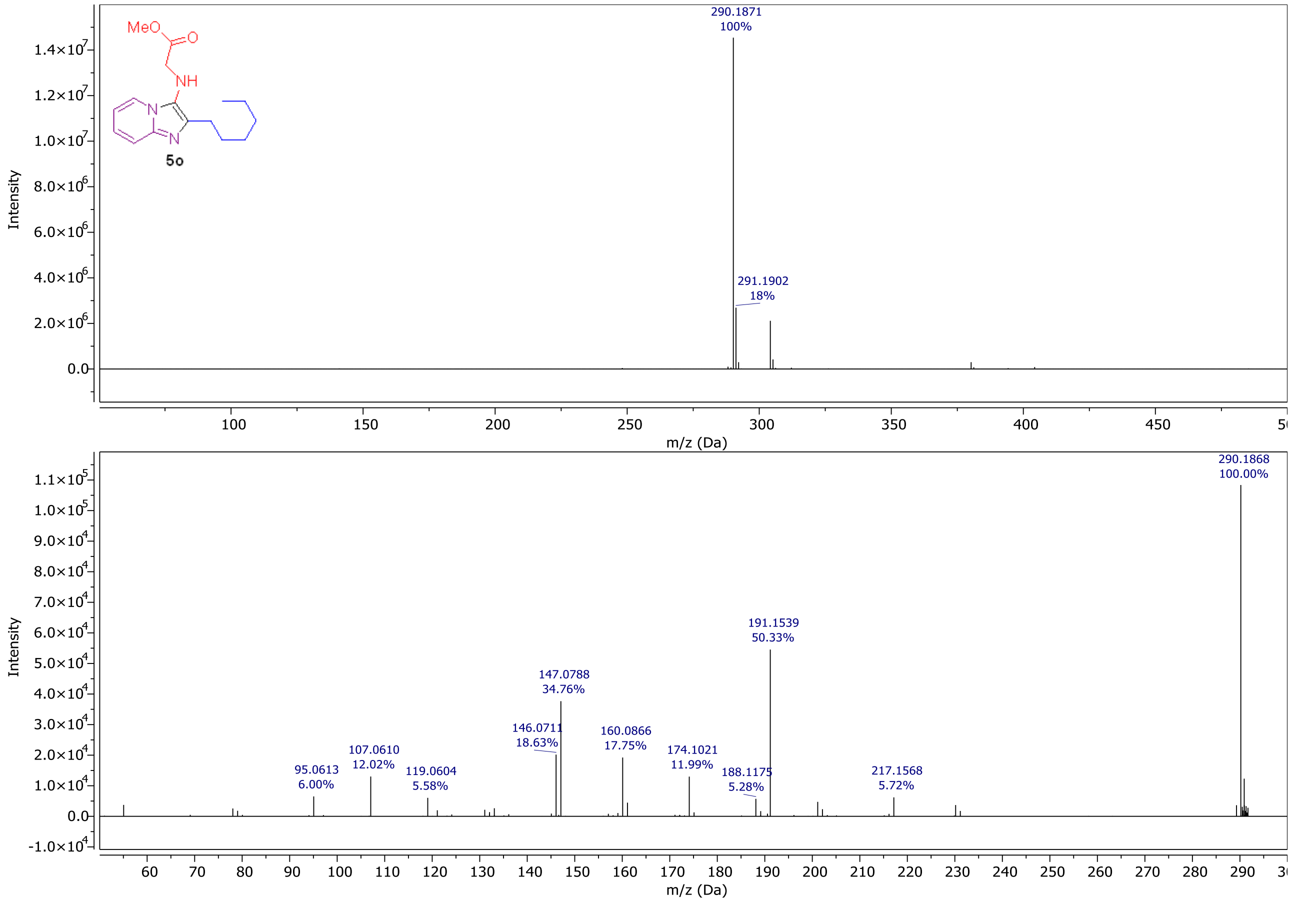

S 272. HRMS (ESI-QTOF) of compound **5o** and HRMS/MS for $[M+H]^+$.
